# Construction of a medicinal leech transcriptome database and its application to the identification of leech homologs of neural and innate immune genes

**DOI:** 10.1186/1471-2164-11-407

**Published:** 2010-06-25

**Authors:** Eduardo R Macagno, Terry Gaasterland, Lee Edsall, Vineet Bafna, Marcelo B Soares, Todd Scheetz, Thomas Casavant, Corinne Da Silva, Patrick Wincker, Aurélie Tasiemski, Michel Salzet

**Affiliations:** 1 Division of Biological Sciences, University of California, San Diego, CA, USA; 2 Scripps Institution of Oceanography, University of California, San Diego, CA, USA; 3 Department of Computer Science, University of California, San Diego, CA, USA; 4 Cancer Biology and Epigenomics Program, Children's Memorial Research Center, and Department of Pediatrics, Northwestern University's Feinberg School of Medicine, Chicago, IL, USA; 5 Department of Biomedical Engineering, Center for Bioinformatics and Computational Biology, University of Iowa, Iowa, USA; 6 CEA, DSV, IG, Genoscope, 2 rue Gaston Crémieux CP5706, 91057 Evry Cedex France; 7 Université Nord de France, CNRS, Laboratoire de Neuroimmunologie et Neurochimie Evolutives, FRE 3249, Université de Lille 1, 59655 Villeneuve d'Ascq, France

## Abstract

**Background:**

The medicinal leech, *Hirudo medicinalis*, is an important model system for the study of nervous system structure, function, development, regeneration and repair. It is also a unique species in being presently approved for use in medical procedures, such as clearing of pooled blood following certain surgical procedures. It is a current, and potentially also future, source of medically useful molecular factors, such as anticoagulants and antibacterial peptides, which may have evolved as a result of its parasitizing large mammals, including humans. Despite the broad focus of research on this system, little has been done at the genomic or transcriptomic levels and there is a paucity of openly available sequence data. To begin to address this problem, we constructed whole embryo and adult central nervous system (CNS) EST libraries and created a clustered sequence database of the *Hirudo *transcriptome that is available to the scientific community.

**Results:**

A total of ~133,000 EST clones from two directionally-cloned cDNA libraries, one constructed from mRNA derived from whole embryos at several developmental stages and the other from adult CNS cords, were sequenced in one or both directions by three different groups: Genoscope (French National Sequencing Center), the University of Iowa Sequencing Facility and the DOE Joint Genome Institute. These were assembled using the phrap software package into 31,232 unique contigs and singletons, with an average length of 827 nt. The assembled transcripts were then translated in all six frames and compared to proteins in NCBI's non-redundant (NR) and to the Gene Ontology (GO) protein sequence databases, resulting in 15,565 matches to 11,236 proteins in NR and 13,935 matches to 8,073 proteins in GO. Searching the database for transcripts of genes homologous to those thought to be involved in the innate immune responses of vertebrates and other invertebrates yielded a set of nearly one hundred evolutionarily conserved sequences, representing all known pathways involved in these important functions.

**Conclusions:**

The sequences obtained for *Hirudo *transcripts represent the first major database of genes expressed in this important model system. Comparison of translated open reading frames (ORFs) with the other openly available leech datasets, the genome and transcriptome of *Helobdella robusta*, shows an average identity at the amino acid level of 58% in matched sequences. Interestingly, comparison with other available Lophotrochozoans shows similar high levels of amino acid identity, where sequences match, for example, 64% with *Capitella capitata *(a polychaete) and 56% with *Aplysia californica *(a mollusk), as well as 58% with *Schistosoma mansoni *(a platyhelminth). Phylogenetic comparisons of putative *Hirudo *innate immune response genes present within the *Hirudo *transcriptome database herein described show a strong resemblance to the corresponding mammalian genes, indicating that this important physiological response may have older origins than what has been previously proposed.

## Background

Contemporary studies of biological systems are increasingly dependent upon detailed knowledge of genomic sequences, as well as spatiotemporal data on gene expression in cells and tissues. This need is being met in part by a growing but limited number of published complete genomic sequences that are now available for many of the most studied model organisms, but for many important and useful species this is not currently the case, though the ever-decreasing cost of large scale sequencing leads to some optimism that this will change in the near future. For functional genomic studies, however, the significantly more modest investment required for creating transcript databases of expressed sequence tags derived from cDNA libraries has provided the opportunity to pursue gene discovery and functional genetic studies in the absence of a fully sequenced genome. We report here the creation of a transcriptome resource for the medicinal leech, an organism with a long history of contributions in neuroscience.

The medicinal leech, *Hirudo medicinalis*, is an important model system for the study of nervous system structure, function, development, regeneration and repair. It is also a unique species in being presently approved for use in medical procedures, such as clearing of pooled blood following certain surgical procedures [1]. It is a current, and potentially also future, source of medically useful molecular factors, such as anticoagulants and antibacterial peptides [2-12], which may have evolved as a result of its parasitizing large mammals, including humans. Because of its relative simplicity and accessibility, the central nervous system (CNS) the medicinal leech, *Hirudo medicinalis*, has been extensively studied and analyzed. Central neurons can be identified, beginning early in embryogenesis, and most have been characterized anatomically and physiologically, their synaptic connectivities assayed, and their roles in particular behaviors determined [13]. The leech CNS has also become a focus for studies of the cellular and molecular mechanisms of development, regeneration and repair, as well as the interface of neural function and the innate immune response [14-20]. Recent advances in the application of contemporary molecular genetic and biochemical techniques to studies of the leech nervous system, including RNA interference [21] and ectopic gene expression in single identified cells [22] or groups of cells [23], as well as mass spectrometry (MS) imaging of embryonic whole mounts and adult sections [24], have opened the door to detailed studies of the mechanisms underlying fundamental biological phenomena.

Leeches are annelids with a fixed number of segments (32 metameres), in contrast to other annelid groups (i.e., oligochaetes and polychaetes), which have variable numbers. The CNS of the leech consists of 32 bilateral neuromeres, of which the 4 anterior-most fuse to form the sub-esophageal ganglion and the 7 posterior-most fuse to form the tail ganglion. Individual ganglia in mid-body segments are comprised of single bilateral neuromeres connected to each other by a bilateral pair of nerves (the lateral "connectives") and a single small medial nerve (Faivre's), and to the periphery by two or three bilateral pairs of nerves ("roots") that branch in stereotypic patterns. In addition, many sensory neurons in the body wall and other internal organs comprise the peripheral nervous system (PNS), providing a variety of sensory information to the CNS.

In hirudinid leeches, each segmental ganglionic primordium gives rise to ~400 neurons [25]. Most of these occur as bilateral pairs (~180-190 pairs), but 5-8% are unpaired, with at least some becoming unpaired through cell death [25,26]. Thus, understanding how a leech segmental ganglion functions requires, in principle, detailed knowledge of the function and connectivity of only ~200-220 individual neurons. Moreover, since each segmental ganglion is a variation on a theme (with the exception of the "sex" ganglia of body segments 5 and 6, which have additional complements of neurosecretory cells [27]), the leech has one of the most accessible nervous systems from a systems analysis point of view. Current knowledge of which neurons contribute to the activity of neuronal circuits responsible for generating specific behavioral responses (see review by [28]) is becoming much more complete as a result of the recent and successful application of multi-neuronal functional imaging to leech ganglia [29].

Lacking within this constellation of detailed knowledge about the nervous system of the medicinal leech are genomic and transcriptomic sequence databases of sufficient size to enable detailed genetic functional studies. This paucity led us to undertake the construction of EST libraries, particularly from neural tissue, the sequencing of over 130,000 clones, and the generation and analysis of a representative transcriptomic database reported herein. The generation of these resources paves the way for an in-depth examination of genetic pathways involved in development and regeneration of the nervous system as well as mechanisms of neuroimmunity.

## Results and Discussion

### Generation and assembly of total embryonic and adult CNS ESTs

Our general goal was to define a large (but not necessarily complete) representative set of the genes expressed in the embryonic and adult central nervous systems of the medicinal leech that would be useful in future analyses of neural development, neural regeneration and repair, neural stress responses and neural innate immune responses.

To this end, we generated expressed sequence tags (ESTs) from two cDNA libraries derived from (a) multiple embryonic stages and (b) adult central nervous system (CNS). This approach has been successfully used in the past to identify the set of transcribed genes specific to an organ or tissue of a particular organism (see e.g., [30]).

After subtraction of the most abundant clones, the libraries were sequenced using different strategies at three separate sites. Sequencing at the University of Iowa included embryonic clones and adult CNS clones, mostly from the 3' end; at the Joint Genome Institute sequences were generated starting at both 5' and 3' ends of clones from the embryonic cDNA library; and at Genoscope, sequences were generated only from the 5' ends of adult nervous system library clones exclusively. A total of 133,161 reads were generated, 41,928 (31%) representing embryonic transcripts and 91,233 (69%) adult transcripts; the contributions from each source are enumerated in Table 1. Of these, 76% were sequenced 5' to 3', and 24% 3' to 5'.

**Table 1:** Numbers of raw sequences used to build the transcriptome database

Source	Whole Embryo 5' to 3'	Whole Embryo 3' to 5'	Adult CNS 5' to 3'	Adult CNS 3' to 5'	Total
**JGI**	13,492	13,354			26,846
**Genoscope**			87,763		87,763
**Iowa**	87	14,995		3,470	18,552

****Total****	13,579	28.349	87,763	3,470	**133,161**

The ~133,000 raw sequences represent ~86.2 × 10^6 ^nucleotides. Raw sequences were trimmed of vector and repetitive as well as low-quality sequences, yielding 133,161 high-quality masked ESTs with an average read length of 648 nt. Some of the sequences were paired (clones sequenced from both ends) and if they overlapped, were merged. These operations removed about 5% of the raw sequence data, leaving ~81.6 × 10^6 ^nucleotides and an average sequence length of 656 nt.

The sequences were assembled using the phrap program, which yielded 31,232 contiguous sequences (contigs - see Table 2). About 20% of these were sufficiently similar to others that they might be considered repeats. They might also represent genetic variants since our libraries likely contain transcripts from two very closely related species of European medicinal leeches, *Hirudo medicinalis *and *Hirudo verbana *[31]. Or, they may represent splice variants. Since splice variation is abundant in the human CNS [32,33], our assembly was tuned to preserve small variations as separate transcripts (See Methods for detail). Thus, we estimate that these complete and partial transcripts may represent ~25,000 unique gene structures. 3% of the transcripts were assembled from only embryonic sequences, 23% from only adult CNS sequences, and 74% from clones derived from both sources. Of the total 133,161 ESTs, 78,288 contributed non-redundant information to the assembly. The remaining ESTs matched subsequences of contributing ESTs. Table 2 shows the numbers of contiguous sequences that were assembled from different numbers of non-redundant ESTs; 16,710 transcripts are assembled from two or more ESTs, and 14,522 are singletons, of which 8,612 matched no assembled transcript at any percent identity.

**Table 2:** The numbers of contigs comprised of data from 1 to 15 ESTs

Number of ESTs	Number of Contigs
1	14522
2	5778
3	3541
4	2413
5	1852
6	1283
7	826
8	469
9	280
10	138
11	68
12	30
13	19
14	7
15	6
16+	0

**Total**	**31232**

Contigs resulting from the clustering of 1 to 15 ESTs

Computational analysis of the sequence data was performed to evaluate sequence redundancy within and across datasets, sequencing quality, and transcript paralogy (see Methods for details).

### Annotation of the assembled sequences: Interspecies comparisons

The 31,232 sequences from the merged input sets were pairwise aligned with BLASTX using default parameters with the non-redundant set of public protein sequences, NR, downloaded from GenBank, maintained at NCBI (http://www.ncbi.nlm.nih.gov, May 14, 2009). For each query sequence, its suite of pairwise alignments was evaluated to select a well-supported description line and to build a weighted index of descriptive words. Using a relevance scoring algorithm based on alignment qualities (see Methods for details), the most relevant description was selected as representative for the query sequence.

Table 3 presents a summary of the number of matches of putative *Hirudo *peptides/proteins with those of a selected set of species representative of three major metazoan phyla, the Lophotrochozoa (which includes leeches), the Ecdysozoa and the Chordates, for which complete genomic sequence data is openly available. For each of seven species, the number of transcripts which rank best through seventh-best match is presented. As might be expected, the closest relation, with 13,047 best matches and 16,732 total among the comparison group of seven genomes, is to *Helobdella robusta*, a species belonging to a distantly related family of non-blood sucking leeches [31]. Another annelid, the polychaete *Capitella*, is next with 2,905 best matches and a total of 15,153. The next best matches are to chordates, with Branchiostoma slightly ahead of the zebrafish and human. Sequence homology is significantly lower when the comparison is with the two representatives of the Ecdysozoa, the fruit fly and the small nematode *C. elegans *(see Table 3). The sequences obtained for *Hirudo *transcripts represent the first major database of genes expressed in this important model system.

**Table 3:** Best matches between *Hirudo *proteins and those of selected species

Organism				Rank				
	**1**	**2**	**3**	**4**	**5**	**6**	**7**	**TOTAL**

***Helobdella***	**13047**	1472	622	552	509	362	168	16732
***Capitella***	2905	**7301**	1854	1339	1012	533	209	15153
***Branchiostoma***	1029	2704	**3445**	2502	2436	1222	501	13839
***Danio***	722	2022	**3435**	**3622**	**2721**	1082	278	13882
***Homo***	637	2005	**3401**	**3689**	**2774**	1171	319	13996
***Drosophila***	421	837	1667	1566	2143	**3657**	961	11251
***Caenorhabditis***	121	323	641	676	940	2090	**3711**	8502

Numbers of amino acid sequence top matches of translated *Hirudo *clustered ESTs to protein sequences available in public databases of seven organisms with sequenced genomes. The order of the species reflects the descending number of first rank matches, i.e., the number of instances where the best match was to a particular species. Numbers in bold are maxima for each rank

To construct the seven-proteome comparison to the *Hirudo *transcripts, six-frame translations of transcripts were compared with complete proteomes from seven organisms selected to range in phylogenetic distance from *Hirudo*. For each transcript, for each proteome, the rank from one to seven of the best match was counted. Of note, no proteome consistently had the best match or even the top three matches for *Hirudo*, and every proteome contributed at least a few best matching proteins, with *C. elegans *lowest at 121 proteins with a match rank of 1.

**Figure 1:**
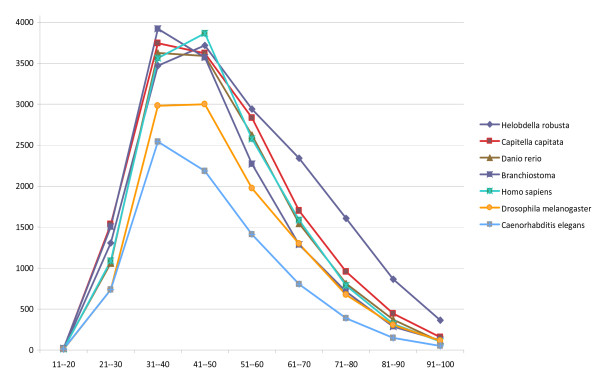
**Graph of the number of translated sequence matches (ordinate) as a function of average percent identity (abscissa) at the amino acid level for Hirudo versus the same species used to construct Table 3**.

Another way to compare the *Hirudo *data to those of the same set of species is shown in Figure 1, where the number of matches is plotted versus average percent identity at the amino acid level for the same comparison group. Again, the closest species is *H. robusta *and the most distant is *C. elegans*. In the middle range of 30-50% identity, the number of matches of translated *Hirudo *transcripts is approximately the same to the vertebrates as it is to the two other annelids, and significantly higher than to either *Drosophila *or *Caenorhabditis*. Of general interest is the small cohort of protein domains that remain perfectly or nearly perfectly conserved across all seven organisms, represented by the alignments in the 91-100% range.

Comparison of translated open reading frames (ORFs) with the other openly available leech datasets, the genome and transcriptome of *Helobdella robusta*, shows an average identity at the amino acid level of 58% in matched sequences. Interestingly, comparison with other available Lophotrochozoans shows similar high levels of amino acid identity where sequences match, for example, 64% with *Capitella capitata *(a polychaete) [34] and 56% with *Aplysia californica *(a mollusk) [35-37], as well as 58% with *Schistosoma mansoni *(a platyhelminth) [38]

These results support the idea that, evolutionarily, the annelids have diverged less from the chordates than have the more highly derived arthropods and nematodes [39-44]. This is further supported by the analysis of a specific group of functionally related proteins, those involved in the innate immune response, as discussed below.

### Gene Ontology analysis of neural proteins represented in the Hirudo transcript database

To test the potential representation of genes in the *Hirudo *transcriptome database that might be involved in nervous system structure, function or development, we aligned six-frame translations of the ~31,000 unique transcripts (assembled sequences + singletons) against the Gene Ontology data in all relevant categories. Overall, we obtained 3,955 matches against 157 of the 166 categories that include the defining term "neuro" and have representative sequences in the Gene Ontology Database. The number of matches for any one term ranged from 1 to a maximum of 1,206. Table 4 presents the number of Hirudo matches for 30 GO categories that are relevant to neuronal development and have the largest numbers of matching transcripts. Numbers of transcripts assigned to each neural process are shown, including numbers assembled from embryo library sequences only, from CNS libraries only, and from a mix of ESTs from both types of libraries. Embryo-only, CNS-only, and "mixed" were determined based on the full set of 133K ESTs, not just the non-redundant contributing ESTs. The total number of unique leech transcripts that matched these 30 categories is 3,003.

**Table 4:** GO categories with highest number of corresponding leech transcripts

GO Index	GO Term	Total	Embryo	Adult CNS	Both
GO:0007409	axonogenesis	1206	209	260	737
GO:0001764	neuron migration	1136	218	238	680
GO:0030182	neuron differentiation	741	140	176	425
GO:0043005	neuron projection	719	136	131	452
GO:0007413	axonal fasciculation	605	102	165	338
GO:0043524	negative regulation of neuron apoptosis	565	81	115	369
GO:0007528	neuromuscular junction development	552	91	133	328
GO:0050885	neuromuscular process controlling balance	494	112	103	279
GO:0048666	neuron development	450	75	156	219
GO:0010001	glial cell differentiation	441	63	142	236
GO:0050767	regulation of neurogenesis	397	56	109	232
GO:0045665	negative regulation of neuron differentiation	389	84	90	215
GO:0055059	asymmetric neuroblast division	377	63	64	250
GO:0051124	synaptic growth at neuromuscular junction	347	60	64	223
GO:0045664	regulation of neuron differentiation	343	48	86	209
GO:0043525	positive regulation of neuron apoptosis	341	53	67	221
GO:0048663	neuron fate commitment	330	54	104	172
GO:0007405	neuroblast proliferation	324	56	68	200
GO:0050768	negative regulation of neurogenesis	288	52	106	130
GO:0021952	CNS projection neuron axonogenesis	255	41	54	160
GO:0051402	neuron apoptosis	252	38	56	158
GO:0021523	somatic motor neuron differentiation	237	36	85	116
GO:0045200	establishment of neuroblast polarity	234	52	36	146
GO:0019838	growth factor binding	227	35	40	152
GO:0007400	neuroblast fate determination	227	30	83	114
GO:0021522	spinal cord motor neuron differentiation	221	32	67	122
GO:0045666	positive regulation of neuron differentiation	217	39	48	130
GO:0021954	central nervous system neuron development	216	38	45	133
GO:0022008	Neurogenesis	187	27	78	82
GO:0043523	regulation of neuron apoptosis	182	30	37	115

Partial *Hirudo *protein sequences were extracted from the alignment data and annotated with their corresponding GO categories. (For the list of the GO identifiers matched by each of the *Hirudo *transcripts, as well as to obtain the *Hirudo *transcript sequences, please see additional files 1 and 2). Our results indicate that the *Hirudo *transcript database contains a significant number of neural sequences, and that it will provide a useful resource for exploring various aspects of nervous system function and development.

### Innate immunity response genes in Hirudo

The innate immune response is an evolutionarily ancient defense strategy against pathogens that has been documented widely in living organisms, including plants and fungi as well as invertebrate and vertebrate animals. In vertebrates, its major functions include: (1) recruiting immune system cells to infection sites though the production of chemokines and cytokines; (2) activating the complement cascade in order to identify pathogens, activate cells to promote pathogen clearance and stimulate the adaptative immune response; (3) interacting specifically with pathogens through membrane or cytosolic receptors in leukocytes in order to remove pathogens from organs and tissues; (4) activating the adaptative immune response though antigen presentation processes [45-51].

To search for the major players for these four functions, we screened the *Hirudo *transcriptome database for possible homologs of vertebrate genes in these categories. The results of this analysis are shown in Table 5. The 92 different transcripts identified fell into eight groups relevant to the immune system, including: pattern recognition receptors (PRR), PRR pathways, cytokine-related molecules, complement system factors, clotting and fibrinolytic cascades, cluster of differentiation related genes, effector genes and adaptive immune response factors.

**Table 5:** *Hirudo *transcripts identified as putative homologs of immune genes

Groups of Immune Factors	TOTAL TRANSCRIPTS	EMBRYO ONLY	ADULT CNS ONLY	MIXED
PRR pathway proteins	22	1	2	19
Pattern recognition Receptors (PRRs)	12	1	4	7
Antimicrobial response factors (Effectors)	11	0	2	9
Complement system	19	0	4	15
Clotting and fibrinolytic cascades	4	1	2	1
Cytokines	5	0	0	5
Cluster of differentiation related molecules	8	0	1	7
Related to vertebrate adaptive immune system	11	1	2	8

TOTAL	92	4	17	71

Numbers of *Hirudo *transcripts identified as putative homologs of genes belonging to seven major groups implicated in the innate immune response, plus a group of potential representatives of the adaptive immune response. Most transcripts are assembled from both whole embryo and adult CNS EST clones, with ~3% present exclusively in the embryonic clones and ~18% exclusively in the Adult CNS. Identification of individual transcripts and the corresponding names of their putative homologs can be found in additional file 3.

As can be seen in Table 5, of the 92 transcripts identified in the database as putatively related to immunity, 88 were derived from EST clones obtained from adult CNS tissues, suggesting that the nervous system is deeply involved in and capable of mounting a fully capable innate immune response. A caveat that needs to be considered is that the leech CNS normally resides within a blood sinus and is therefore continuously in contact with cells of the blood and circulatory system, including fibroblasts, macrophages and microglia, which have been implicated in immune responses in various systems. Some of these may have been carried with the dissected adult nervous systems that provided the mRNA from which the EST libraries were made. Further work will be essential in order to determine whether neurons and neuroglia do express all of the factors we have identified as neuronal or not.

We also determined the numbers of individual EST clones in the raw data that showed high sequence overlap (90% and 96% sequence identity; see additional file 3) as a way to gain some measure of the relative abundances of the putative immune system transcripts present in the EST libraries. As can be seen in this table, with a 90% nucleotide match criterion, the numbers of clones matching the assembled transcripts range from a single one to over several hundred. The significance of this variability in the frequency with which clones corresponding to these transcripts were picked needs to be explored in detail, but it does suggest that some components or some pathways are actively and continuously expressed at higher levels in the adult nervous system.

#### Pattern recognition receptors (PRRs)

Danger signaling receptors are well conserved in leeches. The innate immune system uses different molecules that sense pathogen-associated molecular patterns. These include Toll-like receptors (TLRs), retinoic-acid-inducible gene-1 (RIG-1-like) receptors (RLRs), and the NOD-like receptors (NLRs), all of which contain Leucine Rich Repeat domains (LRRs). Some immunoglobulin superfamily members also contain LRR domains and are known as ISLRs (immunoglobulin superfamily containing Leucine-rich repeats), as for example the Trk neurotrophin receptor protein [52].

#### TOLL-like receptors (TLRs)

In the *Hirudo *EST libraries reported here, our analysis led to the identification of 4 TLRs (Table 5; additional file 3). The complete sequence of one of these, *Hm*TLR1, has been obtained and shows particular homology to the mouse TLR13 [17] Interestingly, in the leech nervous system HmTLR1 appears to be associated with the expression of a cytokine, EMAPII, following exposure to bacterial toxins or in response to a nerve crush [17], but not with the expression of antimicrobial peptides known to be expressed by the central nervous system [16]. It is worth pointing out that these antimicrobial peptides (neuromacin, lumbricin) appear to exert neurotrophic effects after a nerve crush [16] in line with recent data obtained in mammals [53].

#### TLR pathways

Analysis of the *Hirudo *transcriptome database reveals the presence of putative homologs of nearly all factors reported to play critical roles in human TLR pathways, with the exception of homologs of TRIF, TAB1/2 and the Endosome receptor (Table 5; for EST identification and to obtain sequences, see additional file 3). This stands in sharp contrast to other invertebrates, such as insects and nematodes, for which the PRR pathways thus far appear to be much simpler, with many components missing. Whether all the identified leech putative homologs indeed play similar functional roles remains to be shown by further analysis, but their presence in the transcriptome database adds support to the hypothesis that Lophotrochozoan genetic programs are more closely related to those of vertebrates than are those of *Drosophila *and *Caenorhabditis*, two highly-derived members of the other major protostomian group, the Ecdysozoans

#### Immune Effectors: Antimicrobial peptides

Several antimicrobial peptides (lumbricin, theromacin, theromyzin) and destabilases sharing activities against Gram+ and/or Gram- bacteria have been detected and cloned from the *Hirudo *CNS [16] (see additional file 3). We have also identified putative leech homologs of serpins (eglin c) and a tryptase inhibitor (LDTI), which in other systems are known to be active against viruses like HIV or Hepatitis C Virus NS3 protease [54,55].

#### Cytokines

Several cytokines have been identified recently in leech (Table 5; additional file 3), *e.g*., one is related to human p43/Endothelial monocyte-activating polypeptide 2 (EMAP2) [17]. Interestingly, in the leech nervous system HmTLR1 appears to be associated with the expression of the cytokine EMAP2 following exposure to bacterial toxins or in response to a nerve crush [17] but not with the expression of antimicrobial peptides also present in the nervous system [16].

Once activated, danger sensing receptors can promote the production of numerous molecular effectors like antimicrobial peptides (AMPs), chemokines and cytokines. These factors participate in the recruitment of immune cells, development of the inflammatory response and finally, in mammals, the adapative immune response. Among the effectors already discovered in leeches, HmTLR1 is linked to the cytokine related to EMAP2 in the context on the brain immune response after [17]. EMAP2 is the first cytokine-related molecule characterized in invertebrate nervous systems. In mammals, EMAP2 is known to participate in the recruitment of polymorphonuclear leukocytes and mononuclear phagocytes, to promote endothelial apoptosis, and to enhance the expression of some other cytokines [56].

#### Complement

The *Hirudo *database contains putative homologs of the majority of elements thought to participate in pathogen recognition in vertebrates through cell-membrane carbohydrate detection, opsonization and phagocytosis through C3-related protein and α2 macroglobulin-related protein (Table 5, additional file 3). However, the first element already characterized in the leech brain, related to C1q, has been shown to be involved in microglial chemotaxis [18].

#### Cluster of Differentiation (CD) proteins

The CD system is commonly used as cell markers, allowing cells to be defined based on what molecules are present on their surface. In particular, CD proteins are often used to associate cells with certain immune functions. While using one CD molecule to define populations is uncommon (though a few examples exist), combining markers has allowed for cell types with very specific definitions within the immune system [57,58]. We detected several putative leech CDs (Table 5; additional file 3) that are similar to mammalian CDs, e.g., *Hm*CD45, sharing 55% identity with human CD45, and *Hm*CD20, HmCD19 and HmCD61 sharing 32%, 31% and 31% identity with mouse CD20, CD19 and CD61. These data are in line with the results obtained by de Eguileor et al., [57,58], who used human monoclonal antibodies to detect different leech hemocytic cell types, *e.g*., Macrophage-like cells positive for CD25, CD14, CD61, CD68, CD11b and CD11c, NK-like cells positive for CD25, CD56, CD57 and CD16, and granulocytes positive for CD11b and CD11c.

#### Adaptive Immune Response elements

The data discussed above indicate that the majority of proteins known to participate in the vertebrate *innate *immune response are present in the medicinal leech transcriptome. This raises an interesting question: are orthologs of factors implicated in the vertebrate *adaptive *immune response also present in the leech transcriptome? Indeed, several are, including genes related to Rag-1 (Recombination activating gene) as well as calnexin, calreticulin, cathepsins and several others (additional file 3). For example, the *Hm*Rag-1 transcript in the *Hirudo *database displays high sequence homology with vertebrate Rag-1, particularly in two MtN3/saliva domains (average 48% homology). The presence of a Rag-1 related gene in leech is suggestive of the presence of an adaptative response in these long-lived span animals (around 30 years) and opens the door to a reconsideration of the evolution of immune response. Determining whether molecules sharing recombination signal sequence (RSSs) [59,60] homology are present in the leech will be an important step towards establishing the presence of a real adaptive immune response in the medicinal leech, but the presence of Rag-1 related genes in leech is consistent with recent data obtained in the *Aplysia *genome, in which *a N-RAG-TP *transposon encodes a protein similar to the N-terminal part of Rag-1 in vertebrates has been discovered [61]. Similarly, a Rag1/2-like cluster has been found in the sea urchin genome [62-65]. These data are consistent with the theory that V(D)J recombination reaction in jawed vertebrates catalyzed by the Rag-1 and Rag-2 proteins could have emerged approximately 500 million years ago from transposon-encoded proteins. Interestingly, the "core" region of Rag-1 required for its catalytic activity is significantly similar to the transposase encoded by DNA transposons that belong to the Transib superfamily. This superfamily was discovered recently based on computational analysis of the fruit fly, the African malaria mosquito, yellow fever mosquito, silkworm, dog hookworm, hydra, soybean rust and sea urchin genomes [66]. The leech Rag-1-related molecule also aligns with the core part of the Transib transposase from *Helicoverpa zea *[67] further supporting the hypothesis. The complete gene sequence will allow us, in the future, to confirm definitively its homology.

While these observations need to be supplemented by functional studies that confirm the tentative identifications, they do raise a question that needs to be fully explored: did the adaptive immune response evolve earlier than presently thought?

## Conclusions

We expect that the open availability of the *Hirudo *transcript database described herein will help researchers interested in pursuing both functional and comparative studies of proteins and peptides involved in many important biological phenomena. Transcript sequence information is an essential complement to other on-going studies of the leech nervous system, making it possible to explore systemically the genetic programs and the molecular mechanisms that specify individual CNS cells and their ensemble properties in this important model organism. Since gene expression and function can now be assayed and modulated in individual leech neurons or groups of neurons, a systems level approach, focused on relating the neural expression of genetic programs to physiological programs, would be timely and perhaps uniquely feasible in the leech.

Considering the immune response, our data suggest that a well conserved innate immune response, very similar to that found in vertebrates as well as other invertebrate species, including *Biomphalaria glabrata *[68-71], *Daphnia pulex *[72], *Crassostrea gigas *[73], Aplysia [61], and *Chlamys farreri *[74], is present in *Hirudo*, but many details need to be explored further. The medicinal leech is a very important model for exploring interactions between danger sensing receptors in and anti-microbial responses to both bacteria and viruses, given its interactions over time with its human hosts. Perhaps the most important aspect of the observations we are reporting here is that most if not all the immune factors and mechanisms we have identified appear to be present in the *Hirudo *nervous system. Thus, we have preliminary evidence that an essentially complete innate immune response occurs in the leech CNS, especially after mechanical damage, such as a nerve crush, during the complex processes that underlie regeneration. Thus, this model system will allow dissecting the cross-talk between neurons, macroglia and microglia cells, as well as cells and other factors found in the haemolymph. The leech ventral nerve cord is covered by a semi permeable protective capsule and resides within the ventral sinus of the circulatory system, thus being continuously bathed by haemolymph. This capsule and the interaction with blood resemble the mammalian hematological blood-brain barrier. *Hirudo*, therefore, is an excellent model system for exploring fundamental questions about the interaction of the nervous and innate immune systems, including (a) What is the range of functions of microglia? (b) What are the interactions among neurons, glia and blood-borne cells in the responses to pathogens, mechanical damage and other stresses? And (c) What is the nature of the innate immune response mounted by the nervous system?

## Methods

### Animal maintenance and tissue preparation

Leech embryos and adults used in these experiments were obtained from a *Hirudo medicinalis *colony maintained in our laboratory. Prior to use, embryos were removed from their cocoons and kept in artificial spring water (0.5 g/l Instant Ocean, Aquarium Systems) at 22°C, and staged according to the criteria of Fernandez and Stent [75]. At this temperature, day 0 (E0) is defined as the day of cocoon deposition and day 30 (E30) as the day of emergence of the juvenile animal from the cocoon.

### RNA isolation

To construct whole embryo and adult CNS libraries, total RNA was extracted in RNALater (Ambion) using Trizol reagent (Gibco BRL, Rockville, MD). Total RNA was quantitated by spectrophotometry and the quality was determined by 2% formaldehyde-agarose gel electrophoresis. Poly(A)+ RNA was isolated from total RNA samples using oligo-(dT)-cellulose chromatography.

The method used for the construction of directionally cloned cDNA libraries [76] includes a column chromatography step that is aimed at eliminating unwanted DNA fragments (primers and adaptors) and short cDNAs (e.g., those consisting exclusively of poly(A) tail). Although aware of the possibility of excluding cDNAs derived from genuine short transcripts, this step has proven important to minimize generation of nuisance ESTs in large-scale sequencing projects [77].

### Construction of cDNA libraries

DNase-treated poly-(A)+ samples were used to create start (non-normalized) cDNA libraries from which normalized and subtracted cDNA libraries were developed. For each library, cDNA was primed with the following oligo (dT) primer, [TGTTACCATTCTGATGTTGGAGCGGCCGC-N[6-10]-T [76]]. Each primer contained a NotI restriction site for directional cloning and a unique oligonucleotide library tag, which identifies the condition of origin (embryo or adult CNS; Gavin et al. 2002). Double-stranded cDNA was ligated to EcoRI adaptors [5'-AATTGGCACGAGG-3', 3'-GCCGTGCTCC-5'], digested with NotI, and directionally cloned into the phagemid vector pT7T3-Pac as described in [76]. Each library comprised several times more recombinants than the expected number of transcripts from the RNA populations utilized, and thus can be treated as if they had the same number of primary recombinants.

### Sequencing, analysis and clustering

Di-deoxy terminator sequencing was performed from the 3' end of the cDNA clones using M13 forward (5'-GTTTTCCCAGTCAC-3') primers in a 96-well format via cycle sequencing with dRhodamine dye terminator chemistry (Applied Biosystems, Foster City, CA). After thermal cycling, sequencing reactions were processed and analyzed on ABI 3730xl, ABI-377 or ABI-3700 capillary sequencer as described in [78]. Nucleotide sequences and per-base quality values were extracted from the ABI-generated chromatograph files (SCF and AB1 files) using the phred base-calling program and evaluated for 3 features: 1) overall sequence quality (phred q-score >25); 2) percent of sequence (in nt) over q20 > 50%, and 3) the quality-trimmed EST insert length of more than 100 bp [79].

ESTprep [80] and RepeatMasker [81] programs were used to assess the presence of the following EST features: vector cloning site, restriction site, polyadenylation tail and signal sequence, library tag, and potential contaminating sequences from *Escherichia coli *as described in [78]. Local clustering of the ESTs was performed using the sequence-based clustering program UIcluster [82], allowing matches based on both the forward and reverse complements.

ESTs were screened for spurious ribosomal RNA sequences as follows. A database was constructed of 18 S, 28 S, and 16 S rRNA gene sequences from closely related organisms with rRNA sequences in NR, including *Aeolosoma, Aliolimnatis, Aporrectodea, Diestecostoma, Eisenia, Enchytraeus, Haemadipsa, Haementeria, Haemopis, Helobdella, Hirudo, Inanidrilus, Lumbricus, Smithsonidrilus, Stylaria, Tubifex*, and Tubificoides species. ESTs aligning partially or completely with rRNAs at 90% identity or higher were flagged as rRNA suspects or rRNA genes, respectively.

### Sequence analysis and clustering

Computational analysis of the sequence data was performed to evaluate sequence redundancy within and across datasets, sequencing quality, and transcript paralogy. The analysis assembled overlapping reads into contiguous sequences (contigs). Each input sequence was assigned an identifier that indicated its source library, sequencing center, and sequencing strategy (e.g., 3'-end or 5'-end). Each assembled contig received a new identifier that reflected the identifiers of its contributing input sequences. Contigs were produced by assembling the 133,161 sequences from the three source datasets. For the combined input set, the following computation was executed: all sequences were pairwise aligned with all other sequences using BLASTN with default parameters [83]. If two sequences participated in a pairwise alignment above a pre-selected threshold quality, they were put into a "bin"; additional sequences were added to the bin if they had a sufficient pairwise alignment to a sequence already in the bin. When no further sequences could be added to the bin, a new bin was started with a remaining pairwise alignment, until no further sequences remained with sufficient quality alignments. The sequences within each bin were subjected to assembly using phrap with default parameters http://www.phrap.org. For each bin, phrap produced one or more contigs from the input sequences and labeled unassembled sequences as "singlets" or "problems", depending on the nature of their differences from the contigs. phrap output was evaluated to obtain contigs, singleton sequences, and "problem" sequences for each bin. The most common type of so-called "problem" sequences had a high number of ambiguous nucleotides, denoted by 'N' instead of A, C, G or T. The binning step was intended to facilitate efficiency of assembly. Binning was constrained using a minimum threshold on percent identity for alignments that met the default maximum E-value. Bins were computed with percent identity thresholds of 95%, 90%, 85% and 80%. Similar numbers of contigs were obtained from bins constructed at or below 90% identity. From the 90% identical bins, the 133,161 sequencing reads assembled into 16,710 contigs with two or more contributing sequences plus 8,612 singletons and 5,910 "problem" sequences for a total of 31,232 output sequences.

To facilitate efficiency of assembly and to ensure sensitivity to splice variation and genetic variation, input sequences were binned as described in the previous paragraph. Our binning algorithm accomplished the following: for every sequence, if it had a pairwise alignment with any subsequence of any other input sequence, the sequences were placed in the same bin. Each bin of two or more sequences was subjected to assembly with phrap using default settings to produce consensus contigs, singletons and problem sequences (e.g., sequences with too many ambiguities for phrap's default thresholds). The original, unassembled 3' and 5' sequences generated as a part of this research were submitted to dbEST division of GenBank at National Center for Biotechnology Information (NCBI) under accession numbers ranging from GenBank: EY478949 to GenBank: EY505781 Assembled sequences were annotated through alignments with the NR database of proteins and loaded into a local database available for queries at http://genomes.ucsd.edu/leechmaster/transcriptome-paper/.

Transcripts were annotated with protein functional descriptions based on aggregate alignments with proteins from the non-redundant protein database maintained at NCBI (protein NR, downloaded from http://www.ncbi.nih.gov). Proteins putatively involved in neural processes were further annotated based on alignments to sequences maintained and curated as part of the Gene Ontology project (Gene Ontology Consortium, 2000; April 2009 Release). To assign functional descriptions based on NR alignments, a relevance score was computed for each description and each transcript as follows. For each Hirudo transcript, for its complement of aligned proteins, each word from each description was assigned a weight based on sequence alignment quality. Any word in more than one description received the sum of its alignment qualities. Low information content words like "protein" or "similar" were flagged by their high frequency of occurrence and filtered from contributing to description selection. The resulting list of weighted words for each query was ordered by word weight, and mean and standard deviation (STD) were computed. Words with weights above two times STD were considered significant and used for indexing; others were discarded. Each description received a score that was the sum of its word weights, and the highest scoring description was selected for the query transcript.

## Authors' contributions

ERM, TG and MS conceived the study, participated in its design and coordination and drafted the manuscript. MBS prepared the libraries used in this study and, along with TS and TC, carried out the initial sequencing and clustering. VB contributed to the analysis of transcript domain structure and detection of translated and UTR domains. TG and LE performed the final clustering and created the database and website. CDA and PW contributed extensive sequencing of the adult nervous system transcripts. AT and MS carried out the analysis of sequences related to the immune response genes. All authors read and approved the final manuscript.

## Supplementary Material

Additional file 1 **Supplementary Table S1: Neural Transcripts in Top 30 Gene Ontology Categories**. Transcripts listed for the thirty most highly represented categories. Transcript IDs are linked to protein sequence alignment summaries provided via the Leechmaster Database http://genomes.ucsd.edu/leechmaster. Click here for file

Additional file 2 **Supplementary Table S2: Complete Set of Neural Transcripts Identified by Gene Ontology Analysis**. All transcripts encoding *Hirudo *proteins with homology to Gene Ontology proteins in neural categories. Transcript IDs are linked to protein sequence alignment summaries provided via the Leechmaster Database http://genomes.ucsd.edu/leechmaster. Click here for file

Additional file 3 **Supplementary Table S3: Immune System Transcripts**. Transcripts encoding *Hirudo *proteins with homology to immune factors, listed by functional groups. Transcript IDs are linked to protein sequence alignment summaries provided via the Leechmaster Database http://genomes.ucsd.edu/leechmaster. Click here for file
