# Supplementary material for: Construction of a medicinal leech transcriptome database and its application to the identification of leech homologs of neural and innate immune genes
Source: BMC Genomics. 2010 Jun 25;11:407. doi: 10.1186/1471-2164-11-407 (PMC2996935; doi:10.1186/1471-2164-11-407)
Supplement: Additional file 1 — Supplementary Table S1: Neural Transcripts in Top 30 Gene Ontology Categories. Transcripts listed for the thirty most highly represented categories. Transcript IDs are linked to protein sequence alignment summaries provided via the Leechmaster Database http://genomes.ucsd.edu/leechmaster. [file 1471-2164-11-407-S1.HTM]

| Medicinal Leech Transcriptome Database  Supplementary Table 1 | |  |  |  |  |  |  |  |  |  |  |  |  |  |  |  |  |  |  |  |
|  |  |  |  |  |  |  |  |  |  |  |  |  |  |  |  |  |  |  |  |  |
| Embryo |  |  |  |  |  |  |  |  |  |  |  |  |  |  |  |  |  |  |  |  |
| Adult CNS | GO ID | GO Category (neural - top 30) | Transcript ID | |  |  |  |  |  |  |  |  |  |  |  |  |  |  |  |  |
| Mixed |  |  |  |  |  |  |  |  |  |  |  |  |  |  |  |  |  |  |  |  |
| E | 0055059 | asymmetric neuroblast division | EN-124k-90-group343.jgi\_contig\_JGI\_CBBP19299\_fwd | | | | | | | | | | | | | | | | | |
| E | 0055059 | asymmetric neuroblast division | EN-124k-90-group725.jgi\_paired\_JGI\_CBBP10785\_fwd | | | | | | | | | | | | | | | | | |
| A | 0055059 | asymmetric neuroblast division | EN-124k-90-group807.gs\_71281 | | | | | | | | | | | | | | |  |  |  |
| E | 0055059 | asymmetric neuroblast division | EN-124k-90-group1680.jgi\_contig\_JGI\_CBBP17232\_fwd | | | | | | | | | | | | | | | | | |
| E | 0055059 | asymmetric neuroblast division | EN-124k-90-group1852.jgi\_paired\_JGI\_CBBP10940\_fwd | | | | | | | | | | | | | | | | | |
| E | 0055059 | asymmetric neuroblast division | EN-124k-90-group2530.jgi\_paired\_JGI\_CBBP5537\_fwd | | | | | | | | | | | | | | | | | |
| E | 0055059 | asymmetric neuroblast division | EN-124k-90-group3448.jgi\_paired\_JGI\_CBBP19729\_fwd | | | | | | | | | | | | | | | | | |
| A | 0055059 | asymmetric neuroblast division | EN-124k-90-group3564.gs\_15152 | | | | | | | | | | | | | | |  |  |  |
| A | 0055059 | asymmetric neuroblast division | EN-124k-90-group4315.gs\_20609 | | | | | | | | | | | | | | |  |  |  |
| E | 0055059 | asymmetric neuroblast division | EN-124k-90-group5117.jgi\_paired\_JGI\_CBBP19153\_fwd | | | | | | | | | | | | | | | | | |
| A | 0055059 | asymmetric neuroblast division | EN-124k-90-group5938.gs\_54662 | | | | | | | | | | | | | | |  |  |  |
| E | 0055059 | asymmetric neuroblast division | EN-124k-90-group7594.jgi\_paired\_JGI\_CBBP17768\_fwd | | | | | | | | | | | | | | | | | |
| E | 0055059 | asymmetric neuroblast division | EN-124k-90-group7598.jgi\_unpaired\_JGI\_CBBP18343\_fwd | | | | | | | | | | | | | | | | | |
| A | 0055059 | asymmetric neuroblast division | EN-124k-90-group8310.gs\_24913 | | | | | | | | | | | | | | |  |  |  |
| E | 0055059 | asymmetric neuroblast division | EN-124k-90-group8370.jgi\_paired\_JGI\_CBBP17511\_fwd | | | | | | | | | | | | | | | | | |
| A | 0055059 | asymmetric neuroblast division | EN-124k-90-group8977.gs\_49487 | | | | | | | | | | | | | | |  |  |  |
| E | 0055059 | asymmetric neuroblast division | EN-124k-90-group9209.jgi\_paired\_JGI\_CBBP19605\_fwd | | | | | | | | | | | | | | | | | |
| A | 0055059 | asymmetric neuroblast division | EN-124k-90-group9467.gs\_16039 | | | | | | | | | | | | | | |  |  |  |
| A | 0055059 | asymmetric neuroblast division | EN-124k-90-group9714.gs\_64750 | | | | | | | | | | | | | | |  |  |  |
| E | 0055059 | asymmetric neuroblast division | EN-124k-90-group9773.jgi\_paired\_JGI\_CBBP6585\_rev | | | | | | | | | | | | | | | | | |
| A | 0055059 | asymmetric neuroblast division | EN-124k-90-group10312.gs\_22624 | | | | | | | | | | | | | | |  |  |  |
| E | 0055059 | asymmetric neuroblast division | EN-124k-90-group10852.jgi\_paired\_JGI\_CBBP12445\_fwd | | | | | | | | | | | | | | | | | |
| E | 0055059 | asymmetric neuroblast division | EN-124k-90-group11382.jgi\_unpaired\_JGI\_CBBP12542\_fwd | | | | | | | | | | | | | | | | | |
| A | 0055059 | asymmetric neuroblast division | EN-124k-90-group11387.gs\_16543 | | | | | | | | | | | | | | |  |  |  |
| E | 0055059 | asymmetric neuroblast division | EN-124k-90-group11559.jgi\_unpaired\_JGI\_CBBP12231\_fwd | | | | | | | | | | | | | | | | | |
| E | 0055059 | asymmetric neuroblast division | EN-124k-90-group11653.jgi\_paired\_JGI\_CBBP15394\_fwd | | | | | | | | | | | | | | | | | |
| A | 0055059 | asymmetric neuroblast division | EN-124k-90-group11738.gs\_53113 | | | | | | | | | | | | | | |  |  |  |
| E | 0055059 | asymmetric neuroblast division | EN-124k-90-group12452.EN\_iowa\_9722 | | | | | | | | | | | | | | | |  |  |
| E | 0055059 | asymmetric neuroblast division | EN-124k-90-group12603.jgi\_paired\_JGI\_CBBP6814\_fwd | | | | | | | | | | | | | | | | | |
| E | 0055059 | asymmetric neuroblast division | EN-124k-90-group12657.jgi\_contig\_JGI\_CBBP14885\_fwd | | | | | | | | | | | | | | | | | |
| E | 0055059 | asymmetric neuroblast division | EN-124k-90-group12676.jgi\_paired\_JGI\_CBBP17511\_rev | | | | | | | | | | | | | | | | | |
| E | 0055059 | asymmetric neuroblast division | EN-124k-90-group12744.EN\_iowa\_4256 | | | | | | | | | | | | | | | |  |  |
| E | 0055059 | asymmetric neuroblast division | EN-124k-90-group13261.jgi\_contig\_JGI\_CBBP9652\_fwd | | | | | | | | | | | | | | | | | |
| E | 0055059 | asymmetric neuroblast division | EN-124k-90-group13515.EN\_iowa\_10440 | | | | | | | | | | | | | | | |  |  |
| E | 0055059 | asymmetric neuroblast division | EN-124k-90-group13793.jgi\_paired\_JGI\_CBBP2343\_fwd | | | | | | | | | | | | | | | | | |
| E | 0055059 | asymmetric neuroblast division | EN-124k-90-group13916.EN\_iowa\_14815 | | | | | | | | | | | | | | | |  |  |
| A | 0055059 | asymmetric neuroblast division | EN-124k-90-group13937.gs\_79016 | | | | | | | | | | | | | | |  |  |  |
| A | 0055059 | asymmetric neuroblast division | EN-124k-90-group14128.gs\_38861 | | | | | | | | | | | | | | |  |  |  |
| E | 0055059 | asymmetric neuroblast division | EN-124k-90-group14306.jgi\_paired\_JGI\_CBBP12364\_fwd | | | | | | | | | | | | | | | | | |
| A | 0055059 | asymmetric neuroblast division | EN-124k-90-group14320.gs\_86221 | | | | | | | | | | | | | | |  |  |  |
| A | 0055059 | asymmetric neuroblast division | EN-124k-90-group14351.gs\_85401 | | | | | | | | | | | | | | |  |  |  |
| E | 0055059 | asymmetric neuroblast division | EN-124k-90-group14509.jgi\_paired\_JGI\_CBBP933\_fwd | | | | | | | | | | | | | | | | | |
| A | 0055059 | asymmetric neuroblast division | EN-124k-90-group14558.gs\_28342 | | | | | | | | | | | | | | |  |  |  |
| A | 0055059 | asymmetric neuroblast division | EN-124k-90-group14563.gs\_48486 | | | | | | | | | | | | | | |  |  |  |
| E | 0055059 | asymmetric neuroblast division | EN-124k-90-group14573.jgi\_paired\_JGI\_CBBP20040\_fwd | | | | | | | | | | | | | | | | | |
| A | 0055059 | asymmetric neuroblast division | EN-124k-90-group14609.gs\_33389 | | | | | | | | | | | | | | |  |  |  |
| E | 0055059 | asymmetric neuroblast division | EN-124k-90-group14931.jgi\_paired\_JGI\_CBBP10444\_fwd | | | | | | | | | | | | | | | | | |
| A | 0055059 | asymmetric neuroblast division | EN-124k-90-group15056.gs\_73097 | | | | | | | | | | | | | | |  |  |  |
| A | 0055059 | asymmetric neuroblast division | EN-124k-90-group15229.gs\_75551 | | | | | | | | | | | | | | |  |  |  |
| E | 0055059 | asymmetric neuroblast division | EN-124k-90-group15290.EN\_iowa\_9903 | | | | | | | | | | | | | | | |  |  |
| E | 0055059 | asymmetric neuroblast division | EN-124k-90-group15546.EN\_iowa\_13502 | | | | | | | | | | | | | | | |  |  |
| A | 0055059 | asymmetric neuroblast division | EN-124k-90-group15770.gs\_45569 | | | | | | | | | | | | | | |  |  |  |
| E | 0055059 | asymmetric neuroblast division | EN-124k-90-group15944.jgi\_paired\_JGI\_CBBP5778\_fwd | | | | | | | | | | | | | | | | | |
| E | 0055059 | asymmetric neuroblast division | EN-124k-90-group16200.jgi\_paired\_JGI\_CBBP10444\_rev | | | | | | | | | | | | | | | | | |
| A | 0055059 | asymmetric neuroblast division | EN-124k-90-group16267.gs\_28098 | | | | | | | | | | | | | | |  |  |  |
| A | 0055059 | asymmetric neuroblast division | EN-124k-90-group16416.gs\_28586 | | | | | | | | | | | | | | |  |  |  |
| A | 0055059 | asymmetric neuroblast division | EN-124k-90-group16523.gs\_75670 | | | | | | | | | | | | | | |  |  |  |
| A | 0055059 | asymmetric neuroblast division | EN-124k-90-group16830.gs\_82512 | | | | | | | | | | | | | | |  |  |  |
| A | 0055059 | asymmetric neuroblast division | EN-124k-90-group16971.gs\_60466 | | | | | | | | | | | | | | |  |  |  |
| E | 0055059 | asymmetric neuroblast division | EN-124k-90-group17086.jgi\_paired\_JGI\_CBBP6417\_rev | | | | | | | | | | | | | | | | | |
| E | 0055059 | asymmetric neuroblast division | EN-124k-90-group17091.jgi\_paired\_JGI\_CBBP10179\_rev | | | | | | | | | | | | | | | | | |
| A | 0055059 | asymmetric neuroblast division | EN-124k-90-group17193.gs\_13341 | | | | | | | | | | | | | | |  |  |  |
| A | 0055059 | asymmetric neuroblast division | EN-124k-90-group17442.gs\_63748 | | | | | | | | | | | | | | |  |  |  |
| A | 0055059 | asymmetric neuroblast division | EN-124k-90-group17445.gs\_83255 | | | | | | | | | | | | | | |  |  |  |
| A | 0055059 | asymmetric neuroblast division | EN-124k-90-group17563.gs\_80410 | | | | | | | | | | | | | | |  |  |  |
| A | 0055059 | asymmetric neuroblast division | EN-124k-90-group17582.gs\_70636 | | | | | | | | | | | | | | |  |  |  |
| A | 0055059 | asymmetric neuroblast division | EN-124k-90-group17638.gs\_37176 | | | | | | | | | | | | | | |  |  |  |
| A | 0055059 | asymmetric neuroblast division | EN-124k-90-group17744.gs\_30255 | | | | | | | | | | | | | | |  |  |  |
| A | 0055059 | asymmetric neuroblast division | EN-124k-90-group17864.gs\_35131 | | | | | | | | | | | | | | |  |  |  |
| A | 0055059 | asymmetric neuroblast division | EN-124k-90-group18140.gs\_87168 | | | | | | | | | | | | | | |  |  |  |
| A | 0055059 | asymmetric neuroblast division | EN-124k-90-group18148.gs\_80788 | | | | | | | | | | | | | | |  |  |  |
| A | 0055059 | asymmetric neuroblast division | EN-124k-90-group18480.gs\_449 | | | | | | | | | | | | | | |  |  |  |
| M | 0055059 | asymmetric neuroblast division | EN-124k-90-group10.Contig1 | | | | | | | | | | | | | |  |  |  |  |
| M | 0055059 | asymmetric neuroblast division | EN-124k-90-group21.Contig1 | | | | | | | | | | | | | |  |  |  |  |
| M | 0055059 | asymmetric neuroblast division | EN-124k-90-group163.Contig2 | | | | | | | | | | | | | |  |  |  |  |
| M | 0055059 | asymmetric neuroblast division | EN-124k-90-group171.Contig2 | | | | | | | | | | | | | |  |  |  |  |
| M | 0055059 | asymmetric neuroblast division | EN-124k-90-group202.Contig2 | | | | | | | | | | | | | |  |  |  |  |
| M | 0055059 | asymmetric neuroblast division | EN-124k-90-group229.Contig1 | | | | | | | | | | | | | |  |  |  |  |
| M | 0055059 | asymmetric neuroblast division | EN-124k-90-group230.Contig1 | | | | | | | | | | | | | |  |  |  |  |
| M | 0055059 | asymmetric neuroblast division | EN-124k-90-group265.Contig1 | | | | | | | | | | | | | |  |  |  |  |
| M | 0055059 | asymmetric neuroblast division | EN-124k-90-group324.Contig1 | | | | | | | | | | | | | |  |  |  |  |
| M | 0055059 | asymmetric neuroblast division | EN-124k-90-group328.Contig2 | | | | | | | | | | | | | |  |  |  |  |
| M | 0055059 | asymmetric neuroblast division | EN-124k-90-group335.Contig1 | | | | | | | | | | | | | |  |  |  |  |
| M | 0055059 | asymmetric neuroblast division | EN-124k-90-group335.Contig2 | | | | | | | | | | | | | |  |  |  |  |
| A | 0055059 | asymmetric neuroblast division | EN-124k-90-group358.Contig1 | | | | | | | | | | | | | |  |  |  |  |
| M | 0055059 | asymmetric neuroblast division | EN-124k-90-group446.Contig4 | | | | | | | | | | | | | |  |  |  |  |
| M | 0055059 | asymmetric neuroblast division | EN-124k-90-group462.Contig1 | | | | | | | | | | | | | |  |  |  |  |
| M | 0055059 | asymmetric neuroblast division | EN-124k-90-group481.Contig4 | | | | | | | | | | | | | |  |  |  |  |
| M | 0055059 | asymmetric neuroblast division | EN-124k-90-group550.Contig1 | | | | | | | | | | | | | |  |  |  |  |
| M | 0055059 | asymmetric neuroblast division | EN-124k-90-group550.Contig2 | | | | | | | | | | | | | |  |  |  |  |
| M | 0055059 | asymmetric neuroblast division | EN-124k-90-group593.Contig1 | | | | | | | | | | | | | |  |  |  |  |
| M | 0055059 | asymmetric neuroblast division | EN-124k-90-group658.Contig1 | | | | | | | | | | | | | |  |  |  |  |
| M | 0055059 | asymmetric neuroblast division | EN-124k-90-group658.Contig3 | | | | | | | | | | | | | |  |  |  |  |
| M | 0055059 | asymmetric neuroblast division | EN-124k-90-group682.Contig1 | | | | | | | | | | | | | |  |  |  |  |
| M | 0055059 | asymmetric neuroblast division | EN-124k-90-group701.Contig1 | | | | | | | | | | | | | |  |  |  |  |
| M | 0055059 | asymmetric neuroblast division | EN-124k-90-group701.Contig2 | | | | | | | | | | | | | |  |  |  |  |
| M | 0055059 | asymmetric neuroblast division | EN-124k-90-group702.Contig2 | | | | | | | | | | | | | |  |  |  |  |
| M | 0055059 | asymmetric neuroblast division | EN-124k-90-group716.Contig1 | | | | | | | | | | | | | |  |  |  |  |
| M | 0055059 | asymmetric neuroblast division | EN-124k-90-group820.Contig1 | | | | | | | | | | | | | |  |  |  |  |
| M | 0055059 | asymmetric neuroblast division | EN-124k-90-group881.Contig3 | | | | | | | | | | | | | |  |  |  |  |
| M | 0055059 | asymmetric neuroblast division | EN-124k-90-group900.Contig1 | | | | | | | | | | | | | |  |  |  |  |
| M | 0055059 | asymmetric neuroblast division | EN-124k-90-group945.Contig1 | | | | | | | | | | | | | |  |  |  |  |
| M | 0055059 | asymmetric neuroblast division | EN-124k-90-group998.Contig1 | | | | | | | | | | | | | |  |  |  |  |
| M | 0055059 | asymmetric neuroblast division | EN-124k-90-group1069.Contig1 | | | | | | | | | | | | | |  |  |  |  |
| M | 0055059 | asymmetric neuroblast division | EN-124k-90-group1153.Contig1 | | | | | | | | | | | | | |  |  |  |  |
| E | 0055059 | asymmetric neuroblast division | EN-124k-90-group1160.Contig2 | | | | | | | | | | | | | |  |  |  |  |
| M | 0055059 | asymmetric neuroblast division | EN-124k-90-group1160.Contig5 | | | | | | | | | | | | | |  |  |  |  |
| M | 0055059 | asymmetric neuroblast division | EN-124k-90-group1160.Contig6 | | | | | | | | | | | | | |  |  |  |  |
| M | 0055059 | asymmetric neuroblast division | EN-124k-90-group1161.Contig1 | | | | | | | | | | | | | |  |  |  |  |
| M | 0055059 | asymmetric neuroblast division | EN-124k-90-group1161.Contig2 | | | | | | | | | | | | | |  |  |  |  |
| M | 0055059 | asymmetric neuroblast division | EN-124k-90-group1167.Contig2 | | | | | | | | | | | | | |  |  |  |  |
| M | 0055059 | asymmetric neuroblast division | EN-124k-90-group1167.Contig3 | | | | | | | | | | | | | |  |  |  |  |
| M | 0055059 | asymmetric neuroblast division | EN-124k-90-group1203.Contig1 | | | | | | | | | | | | | |  |  |  |  |
| M | 0055059 | asymmetric neuroblast division | EN-124k-90-group1203.Contig2 | | | | | | | | | | | | | |  |  |  |  |
| M | 0055059 | asymmetric neuroblast division | EN-124k-90-group1212.Contig1 | | | | | | | | | | | | | |  |  |  |  |
| E | 0055059 | asymmetric neuroblast division | EN-124k-90-group1255.Contig1 | | | | | | | | | | | | | |  |  |  |  |
| M | 0055059 | asymmetric neuroblast division | EN-124k-90-group1272.Contig1 | | | | | | | | | | | | | |  |  |  |  |
| M | 0055059 | asymmetric neuroblast division | EN-124k-90-group1302.Contig4 | | | | | | | | | | | | | |  |  |  |  |
| M | 0055059 | asymmetric neuroblast division | EN-124k-90-group1304.Contig2 | | | | | | | | | | | | | |  |  |  |  |
| M | 0055059 | asymmetric neuroblast division | EN-124k-90-group1304.Contig4 | | | | | | | | | | | | | |  |  |  |  |
| M | 0055059 | asymmetric neuroblast division | EN-124k-90-group1304.Contig5 | | | | | | | | | | | | | |  |  |  |  |
| M | 0055059 | asymmetric neuroblast division | EN-124k-90-group1304.Contig8 | | | | | | | | | | | | | |  |  |  |  |
| M | 0055059 | asymmetric neuroblast division | EN-124k-90-group1351.Contig1 | | | | | | | | | | | | | |  |  |  |  |
| M | 0055059 | asymmetric neuroblast division | EN-124k-90-group1456.Contig4 | | | | | | | | | | | | | |  |  |  |  |
| M | 0055059 | asymmetric neuroblast division | EN-124k-90-group1456.Contig8 | | | | | | | | | | | | | |  |  |  |  |
| M | 0055059 | asymmetric neuroblast division | EN-124k-90-group1493.Contig2 | | | | | | | | | | | | | |  |  |  |  |
| M | 0055059 | asymmetric neuroblast division | EN-124k-90-group1560.Contig1 | | | | | | | | | | | | | |  |  |  |  |
| M | 0055059 | asymmetric neuroblast division | EN-124k-90-group1728.Contig2 | | | | | | | | | | | | | |  |  |  |  |
| M | 0055059 | asymmetric neuroblast division | EN-124k-90-group1735.Contig4 | | | | | | | | | | | | | |  |  |  |  |
| M | 0055059 | asymmetric neuroblast division | EN-124k-90-group1735.Contig6 | | | | | | | | | | | | | |  |  |  |  |
| M | 0055059 | asymmetric neuroblast division | EN-124k-90-group1754.Contig1 | | | | | | | | | | | | | |  |  |  |  |
| M | 0055059 | asymmetric neuroblast division | EN-124k-90-group1754.Contig2 | | | | | | | | | | | | | |  |  |  |  |
| M | 0055059 | asymmetric neuroblast division | EN-124k-90-group1782.Contig1 | | | | | | | | | | | | | |  |  |  |  |
| M | 0055059 | asymmetric neuroblast division | EN-124k-90-group1915.Contig1 | | | | | | | | | | | | | |  |  |  |  |
| M | 0055059 | asymmetric neuroblast division | EN-124k-90-group1960.Contig1 | | | | | | | | | | | | | |  |  |  |  |
| M | 0055059 | asymmetric neuroblast division | EN-124k-90-group2069.Contig3 | | | | | | | | | | | | | |  |  |  |  |
| M | 0055059 | asymmetric neuroblast division | EN-124k-90-group2074.Contig1 | | | | | | | | | | | | | |  |  |  |  |
| M | 0055059 | asymmetric neuroblast division | EN-124k-90-group2077.Contig3 | | | | | | | | | | | | | |  |  |  |  |
| M | 0055059 | asymmetric neuroblast division | EN-124k-90-group2081.Contig1 | | | | | | | | | | | | | |  |  |  |  |
| M | 0055059 | asymmetric neuroblast division | EN-124k-90-group2107.Contig1 | | | | | | | | | | | | | |  |  |  |  |
| M | 0055059 | asymmetric neuroblast division | EN-124k-90-group2107.Contig2 | | | | | | | | | | | | | |  |  |  |  |
| M | 0055059 | asymmetric neuroblast division | EN-124k-90-group2107.Contig3 | | | | | | | | | | | | | |  |  |  |  |
| M | 0055059 | asymmetric neuroblast division | EN-124k-90-group2107.Contig4 | | | | | | | | | | | | | |  |  |  |  |
| M | 0055059 | asymmetric neuroblast division | EN-124k-90-group2192.Contig1 | | | | | | | | | | | | | |  |  |  |  |
| M | 0055059 | asymmetric neuroblast division | EN-124k-90-group2192.Contig2 | | | | | | | | | | | | | |  |  |  |  |
| M | 0055059 | asymmetric neuroblast division | EN-124k-90-group2199.Contig1 | | | | | | | | | | | | | |  |  |  |  |
| M | 0055059 | asymmetric neuroblast division | EN-124k-90-group2200.Contig1 | | | | | | | | | | | | | |  |  |  |  |
| M | 0055059 | asymmetric neuroblast division | EN-124k-90-group2260.Contig2 | | | | | | | | | | | | | |  |  |  |  |
| M | 0055059 | asymmetric neuroblast division | EN-124k-90-group2268.Contig2 | | | | | | | | | | | | | |  |  |  |  |
| M | 0055059 | asymmetric neuroblast division | EN-124k-90-group2289.Contig1 | | | | | | | | | | | | | |  |  |  |  |
| M | 0055059 | asymmetric neuroblast division | EN-124k-90-group2367.Contig2 | | | | | | | | | | | | | |  |  |  |  |
| M | 0055059 | asymmetric neuroblast division | EN-124k-90-group2409.Contig1 | | | | | | | | | | | | | |  |  |  |  |
| M | 0055059 | asymmetric neuroblast division | EN-124k-90-group2409.Contig3 | | | | | | | | | | | | | |  |  |  |  |
| M | 0055059 | asymmetric neuroblast division | EN-124k-90-group2409.Contig4 | | | | | | | | | | | | | |  |  |  |  |
| M | 0055059 | asymmetric neuroblast division | EN-124k-90-group2428.Contig1 | | | | | | | | | | | | | |  |  |  |  |
| M | 0055059 | asymmetric neuroblast division | EN-124k-90-group2449.Contig1 | | | | | | | | | | | | | |  |  |  |  |
| M | 0055059 | asymmetric neuroblast division | EN-124k-90-group2449.Contig2 | | | | | | | | | | | | | |  |  |  |  |
| M | 0055059 | asymmetric neuroblast division | EN-124k-90-group2498.Contig5 | | | | | | | | | | | | | |  |  |  |  |
| M | 0055059 | asymmetric neuroblast division | EN-124k-90-group2498.Contig7 | | | | | | | | | | | | | |  |  |  |  |
| M | 0055059 | asymmetric neuroblast division | EN-124k-90-group2509.Contig2 | | | | | | | | | | | | | |  |  |  |  |
| M | 0055059 | asymmetric neuroblast division | EN-124k-90-group2537.Contig1 | | | | | | | | | | | | | |  |  |  |  |
| M | 0055059 | asymmetric neuroblast division | EN-124k-90-group2662.Contig1 | | | | | | | | | | | | | |  |  |  |  |
| M | 0055059 | asymmetric neuroblast division | EN-124k-90-group2664.Contig1 | | | | | | | | | | | | | |  |  |  |  |
| M | 0055059 | asymmetric neuroblast division | EN-124k-90-group2664.Contig2 | | | | | | | | | | | | | |  |  |  |  |
| M | 0055059 | asymmetric neuroblast division | EN-124k-90-group2825.Contig1 | | | | | | | | | | | | | |  |  |  |  |
| M | 0055059 | asymmetric neuroblast division | EN-124k-90-group2841.Contig1 | | | | | | | | | | | | | |  |  |  |  |
| M | 0055059 | asymmetric neuroblast division | EN-124k-90-group2846.Contig1 | | | | | | | | | | | | | |  |  |  |  |
| M | 0055059 | asymmetric neuroblast division | EN-124k-90-group2940.Contig2 | | | | | | | | | | | | | |  |  |  |  |
| M | 0055059 | asymmetric neuroblast division | EN-124k-90-group3158.Contig2 | | | | | | | | | | | | | |  |  |  |  |
| E | 0055059 | asymmetric neuroblast division | EN-124k-90-group3241.Contig1 | | | | | | | | | | | | | |  |  |  |  |
| M | 0055059 | asymmetric neuroblast division | EN-124k-90-group3313.Contig1 | | | | | | | | | | | | | |  |  |  |  |
| M | 0055059 | asymmetric neuroblast division | EN-124k-90-group3313.Contig2 | | | | | | | | | | | | | |  |  |  |  |
| M | 0055059 | asymmetric neuroblast division | EN-124k-90-group3313.Contig3 | | | | | | | | | | | | | |  |  |  |  |
| M | 0055059 | asymmetric neuroblast division | EN-124k-90-group3315.Contig6 | | | | | | | | | | | | | |  |  |  |  |
| M | 0055059 | asymmetric neuroblast division | EN-124k-90-group3316.Contig1 | | | | | | | | | | | | | |  |  |  |  |
| M | 0055059 | asymmetric neuroblast division | EN-124k-90-group3365.Contig1 | | | | | | | | | | | | | |  |  |  |  |
| M | 0055059 | asymmetric neuroblast division | EN-124k-90-group3408.Contig1 | | | | | | | | | | | | | |  |  |  |  |
| M | 0055059 | asymmetric neuroblast division | EN-124k-90-group3408.Contig2 | | | | | | | | | | | | | |  |  |  |  |
| M | 0055059 | asymmetric neuroblast division | EN-124k-90-group3520.Contig1 | | | | | | | | | | | | | |  |  |  |  |
| M | 0055059 | asymmetric neuroblast division | EN-124k-90-group3522.Contig1 | | | | | | | | | | | | | |  |  |  |  |
| M | 0055059 | asymmetric neuroblast division | EN-124k-90-group3606.Contig2 | | | | | | | | | | | | | |  |  |  |  |
| M | 0055059 | asymmetric neuroblast division | EN-124k-90-group3707.Contig1 | | | | | | | | | | | | | |  |  |  |  |
| M | 0055059 | asymmetric neuroblast division | EN-124k-90-group3713.Contig1 | | | | | | | | | | | | | |  |  |  |  |
| M | 0055059 | asymmetric neuroblast division | EN-124k-90-group3753.Contig1 | | | | | | | | | | | | | |  |  |  |  |
| M | 0055059 | asymmetric neuroblast division | EN-124k-90-group3753.Contig2 | | | | | | | | | | | | | |  |  |  |  |
| M | 0055059 | asymmetric neuroblast division | EN-124k-90-group3830.Contig1 | | | | | | | | | | | | | |  |  |  |  |
| M | 0055059 | asymmetric neuroblast division | EN-124k-90-group3892.Contig1 | | | | | | | | | | | | | |  |  |  |  |
| M | 0055059 | asymmetric neuroblast division | EN-124k-90-group3997.Contig2 | | | | | | | | | | | | | |  |  |  |  |
| M | 0055059 | asymmetric neuroblast division | EN-124k-90-group4130.Contig1 | | | | | | | | | | | | | |  |  |  |  |
| M | 0055059 | asymmetric neuroblast division | EN-124k-90-group4168.Contig1 | | | | | | | | | | | | | |  |  |  |  |
| M | 0055059 | asymmetric neuroblast division | EN-124k-90-group4168.Contig3 | | | | | | | | | | | | | |  |  |  |  |
| M | 0055059 | asymmetric neuroblast division | EN-124k-90-group4183.Contig1 | | | | | | | | | | | | | |  |  |  |  |
| M | 0055059 | asymmetric neuroblast division | EN-124k-90-group4194.Contig1 | | | | | | | | | | | | | |  |  |  |  |
| M | 0055059 | asymmetric neuroblast division | EN-124k-90-group4349.Contig2 | | | | | | | | | | | | | |  |  |  |  |
| M | 0055059 | asymmetric neuroblast division | EN-124k-90-group4349.Contig3 | | | | | | | | | | | | | |  |  |  |  |
| M | 0055059 | asymmetric neuroblast division | EN-124k-90-group4372.Contig1 | | | | | | | | | | | | | |  |  |  |  |
| M | 0055059 | asymmetric neuroblast division | EN-124k-90-group4410.Contig1 | | | | | | | | | | | | | |  |  |  |  |
| M | 0055059 | asymmetric neuroblast division | EN-124k-90-group4454.Contig1 | | | | | | | | | | | | | |  |  |  |  |
| M | 0055059 | asymmetric neuroblast division | EN-124k-90-group4560.Contig1 | | | | | | | | | | | | | |  |  |  |  |
| M | 0055059 | asymmetric neuroblast division | EN-124k-90-group4599.Contig1 | | | | | | | | | | | | | |  |  |  |  |
| M | 0055059 | asymmetric neuroblast division | EN-124k-90-group4668.Contig1 | | | | | | | | | | | | | |  |  |  |  |
| M | 0055059 | asymmetric neuroblast division | EN-124k-90-group4709.Contig1 | | | | | | | | | | | | | |  |  |  |  |
| E | 0055059 | asymmetric neuroblast division | EN-124k-90-group4723.Contig2 | | | | | | | | | | | | | |  |  |  |  |
| M | 0055059 | asymmetric neuroblast division | EN-124k-90-group4757.Contig1 | | | | | | | | | | | | | |  |  |  |  |
| M | 0055059 | asymmetric neuroblast division | EN-124k-90-group4757.Contig2 | | | | | | | | | | | | | |  |  |  |  |
| M | 0055059 | asymmetric neuroblast division | EN-124k-90-group4763.Contig1 | | | | | | | | | | | | | |  |  |  |  |
| M | 0055059 | asymmetric neuroblast division | EN-124k-90-group4804.Contig1 | | | | | | | | | | | | | |  |  |  |  |
| E | 0055059 | asymmetric neuroblast division | EN-124k-90-group4884.Contig1 | | | | | | | | | | | | | |  |  |  |  |
| M | 0055059 | asymmetric neuroblast division | EN-124k-90-group4913.Contig1 | | | | | | | | | | | | | |  |  |  |  |
| M | 0055059 | asymmetric neuroblast division | EN-124k-90-group4913.Contig2 | | | | | | | | | | | | | |  |  |  |  |
| M | 0055059 | asymmetric neuroblast division | EN-124k-90-group4929.Contig2 | | | | | | | | | | | | | |  |  |  |  |
| M | 0055059 | asymmetric neuroblast division | EN-124k-90-group4933.Contig1 | | | | | | | | | | | | | |  |  |  |  |
| M | 0055059 | asymmetric neuroblast division | EN-124k-90-group4933.Contig2 | | | | | | | | | | | | | |  |  |  |  |
| M | 0055059 | asymmetric neuroblast division | EN-124k-90-group4945.Contig2 | | | | | | | | | | | | | |  |  |  |  |
| M | 0055059 | asymmetric neuroblast division | EN-124k-90-group5063.Contig1 | | | | | | | | | | | | | |  |  |  |  |
| M | 0055059 | asymmetric neuroblast division | EN-124k-90-group5071.Contig3 | | | | | | | | | | | | | |  |  |  |  |
| M | 0055059 | asymmetric neuroblast division | EN-124k-90-group5093.Contig2 | | | | | | | | | | | | | |  |  |  |  |
| M | 0055059 | asymmetric neuroblast division | EN-124k-90-group5298.Contig1 | | | | | | | | | | | | | |  |  |  |  |
| M | 0055059 | asymmetric neuroblast division | EN-124k-90-group5359.Contig3 | | | | | | | | | | | | | |  |  |  |  |
| M | 0055059 | asymmetric neuroblast division | EN-124k-90-group5411.Contig1 | | | | | | | | | | | | | |  |  |  |  |
| M | 0055059 | asymmetric neuroblast division | EN-124k-90-group5525.Contig1 | | | | | | | | | | | | | |  |  |  |  |
| M | 0055059 | asymmetric neuroblast division | EN-124k-90-group5542.Contig1 | | | | | | | | | | | | | |  |  |  |  |
| M | 0055059 | asymmetric neuroblast division | EN-124k-90-group5607.Contig1 | | | | | | | | | | | | | |  |  |  |  |
| M | 0055059 | asymmetric neuroblast division | EN-124k-90-group5616.Contig1 | | | | | | | | | | | | | |  |  |  |  |
| M | 0055059 | asymmetric neuroblast division | EN-124k-90-group5668.Contig1 | | | | | | | | | | | | | |  |  |  |  |
| M | 0055059 | asymmetric neuroblast division | EN-124k-90-group5717.Contig2 | | | | | | | | | | | | | |  |  |  |  |
| M | 0055059 | asymmetric neuroblast division | EN-124k-90-group5742.Contig1 | | | | | | | | | | | | | |  |  |  |  |
| M | 0055059 | asymmetric neuroblast division | EN-124k-90-group5910.Contig1 | | | | | | | | | | | | | |  |  |  |  |
| M | 0055059 | asymmetric neuroblast division | EN-124k-90-group5968.Contig1 | | | | | | | | | | | | | |  |  |  |  |
| M | 0055059 | asymmetric neuroblast division | EN-124k-90-group6033.Contig1 | | | | | | | | | | | | | |  |  |  |  |
| M | 0055059 | asymmetric neuroblast division | EN-124k-90-group6033.Contig2 | | | | | | | | | | | | | |  |  |  |  |
| M | 0055059 | asymmetric neuroblast division | EN-124k-90-group6033.Contig3 | | | | | | | | | | | | | |  |  |  |  |
| M | 0055059 | asymmetric neuroblast division | EN-124k-90-group6195.Contig1 | | | | | | | | | | | | | |  |  |  |  |
| M | 0055059 | asymmetric neuroblast division | EN-124k-90-group6204.Contig1 | | | | | | | | | | | | | |  |  |  |  |
| M | 0055059 | asymmetric neuroblast division | EN-124k-90-group6204.Contig2 | | | | | | | | | | | | | |  |  |  |  |
| M | 0055059 | asymmetric neuroblast division | EN-124k-90-group6400.Contig1 | | | | | | | | | | | | | |  |  |  |  |
| M | 0055059 | asymmetric neuroblast division | EN-124k-90-group6638.Contig1 | | | | | | | | | | | | | |  |  |  |  |
| M | 0055059 | asymmetric neuroblast division | EN-124k-90-group6666.Contig1 | | | | | | | | | | | | | |  |  |  |  |
| M | 0055059 | asymmetric neuroblast division | EN-124k-90-group6742.Contig2 | | | | | | | | | | | | | |  |  |  |  |
| M | 0055059 | asymmetric neuroblast division | EN-124k-90-group6809.Contig1 | | | | | | | | | | | | | |  |  |  |  |
| E | 0055059 | asymmetric neuroblast division | EN-124k-90-group6858.Contig1 | | | | | | | | | | | | | |  |  |  |  |
| M | 0055059 | asymmetric neuroblast division | EN-124k-90-group6923.Contig1 | | | | | | | | | | | | | |  |  |  |  |
| M | 0055059 | asymmetric neuroblast division | EN-124k-90-group6923.Contig2 | | | | | | | | | | | | | |  |  |  |  |
| M | 0055059 | asymmetric neuroblast division | EN-124k-90-group7046.Contig1 | | | | | | | | | | | | | |  |  |  |  |
| M | 0055059 | asymmetric neuroblast division | EN-124k-90-group7149.Contig2 | | | | | | | | | | | | | |  |  |  |  |
| M | 0055059 | asymmetric neuroblast division | EN-124k-90-group7165.Contig1 | | | | | | | | | | | | | |  |  |  |  |
| M | 0055059 | asymmetric neuroblast division | EN-124k-90-group7207.Contig1 | | | | | | | | | | | | | |  |  |  |  |
| A | 0055059 | asymmetric neuroblast division | EN-124k-90-group7213.Contig1 | | | | | | | | | | | | | |  |  |  |  |
| M | 0055059 | asymmetric neuroblast division | EN-124k-90-group7234.Contig1 | | | | | | | | | | | | | |  |  |  |  |
| M | 0055059 | asymmetric neuroblast division | EN-124k-90-group7281.Contig1 | | | | | | | | | | | | | |  |  |  |  |
| M | 0055059 | asymmetric neuroblast division | EN-124k-90-group7281.Contig2 | | | | | | | | | | | | | |  |  |  |  |
| M | 0055059 | asymmetric neuroblast division | EN-124k-90-group7311.Contig1 | | | | | | | | | | | | | |  |  |  |  |
| M | 0055059 | asymmetric neuroblast division | EN-124k-90-group7316.Contig1 | | | | | | | | | | | | | |  |  |  |  |
| M | 0055059 | asymmetric neuroblast division | EN-124k-90-group7316.Contig3 | | | | | | | | | | | | | |  |  |  |  |
| M | 0055059 | asymmetric neuroblast division | EN-124k-90-group7343.Contig1 | | | | | | | | | | | | | |  |  |  |  |
| M | 0055059 | asymmetric neuroblast division | EN-124k-90-group7343.Contig2 | | | | | | | | | | | | | |  |  |  |  |
| M | 0055059 | asymmetric neuroblast division | EN-124k-90-group7420.Contig1 | | | | | | | | | | | | | |  |  |  |  |
| M | 0055059 | asymmetric neuroblast division | EN-124k-90-group7440.Contig1 | | | | | | | | | | | | | |  |  |  |  |
| M | 0055059 | asymmetric neuroblast division | EN-124k-90-group7457.Contig1 | | | | | | | | | | | | | |  |  |  |  |
| M | 0055059 | asymmetric neuroblast division | EN-124k-90-group7528.Contig1 | | | | | | | | | | | | | |  |  |  |  |
| M | 0055059 | asymmetric neuroblast division | EN-124k-90-group7866.Contig1 | | | | | | | | | | | | | |  |  |  |  |
| M | 0055059 | asymmetric neuroblast division | EN-124k-90-group7874.Contig2 | | | | | | | | | | | | | |  |  |  |  |
| M | 0055059 | asymmetric neuroblast division | EN-124k-90-group7875.Contig1 | | | | | | | | | | | | | |  |  |  |  |
| M | 0055059 | asymmetric neuroblast division | EN-124k-90-group7875.Contig2 | | | | | | | | | | | | | |  |  |  |  |
| M | 0055059 | asymmetric neuroblast division | EN-124k-90-group7875.Contig3 | | | | | | | | | | | | | |  |  |  |  |
| M | 0055059 | asymmetric neuroblast division | EN-124k-90-group7876.Contig1 | | | | | | | | | | | | | |  |  |  |  |
| M | 0055059 | asymmetric neuroblast division | EN-124k-90-group7878.Contig2 | | | | | | | | | | | | | |  |  |  |  |
| M | 0055059 | asymmetric neuroblast division | EN-124k-90-group7893.Contig1 | | | | | | | | | | | | | |  |  |  |  |
| M | 0055059 | asymmetric neuroblast division | EN-124k-90-group8010.Contig1 | | | | | | | | | | | | | |  |  |  |  |
| M | 0055059 | asymmetric neuroblast division | EN-124k-90-group8010.Contig2 | | | | | | | | | | | | | |  |  |  |  |
| M | 0055059 | asymmetric neuroblast division | EN-124k-90-group8079.Contig1 | | | | | | | | | | | | | |  |  |  |  |
| M | 0055059 | asymmetric neuroblast division | EN-124k-90-group8079.Contig2 | | | | | | | | | | | | | |  |  |  |  |
| M | 0055059 | asymmetric neuroblast division | EN-124k-90-group8211.Contig1 | | | | | | | | | | | | | |  |  |  |  |
| M | 0055059 | asymmetric neuroblast division | EN-124k-90-group8221.Contig1 | | | | | | | | | | | | | |  |  |  |  |
| A | 0055059 | asymmetric neuroblast division | EN-124k-90-group8281.Contig1 | | | | | | | | | | | | | |  |  |  |  |
| M | 0055059 | asymmetric neuroblast division | EN-124k-90-group8306.Contig3 | | | | | | | | | | | | | |  |  |  |  |
| M | 0055059 | asymmetric neuroblast division | EN-124k-90-group8306.Contig4 | | | | | | | | | | | | | |  |  |  |  |
| M | 0055059 | asymmetric neuroblast division | EN-124k-90-group8325.Contig1 | | | | | | | | | | | | | |  |  |  |  |
| M | 0055059 | asymmetric neuroblast division | EN-124k-90-group8375.Contig1 | | | | | | | | | | | | | |  |  |  |  |
| M | 0055059 | asymmetric neuroblast division | EN-124k-90-group8492.Contig1 | | | | | | | | | | | | | |  |  |  |  |
| A | 0055059 | asymmetric neuroblast division | EN-124k-90-group8519.Contig1 | | | | | | | | | | | | | |  |  |  |  |
| M | 0055059 | asymmetric neuroblast division | EN-124k-90-group8568.Contig1 | | | | | | | | | | | | | |  |  |  |  |
| M | 0055059 | asymmetric neuroblast division | EN-124k-90-group8645.Contig1 | | | | | | | | | | | | | |  |  |  |  |
| A | 0055059 | asymmetric neuroblast division | EN-124k-90-group8668.Contig1 | | | | | | | | | | | | | |  |  |  |  |
| M | 0055059 | asymmetric neuroblast division | EN-124k-90-group8799.Contig2 | | | | | | | | | | | | | |  |  |  |  |
| M | 0055059 | asymmetric neuroblast division | EN-124k-90-group8850.Contig1 | | | | | | | | | | | | | |  |  |  |  |
| A | 0055059 | asymmetric neuroblast division | EN-124k-90-group8957.Contig1 | | | | | | | | | | | | | |  |  |  |  |
| M | 0055059 | asymmetric neuroblast division | EN-124k-90-group9045.Contig1 | | | | | | | | | | | | | |  |  |  |  |
| M | 0055059 | asymmetric neuroblast division | EN-124k-90-group9079.Contig1 | | | | | | | | | | | | | |  |  |  |  |
| A | 0055059 | asymmetric neuroblast division | EN-124k-90-group9245.Contig1 | | | | | | | | | | | | | |  |  |  |  |
| M | 0055059 | asymmetric neuroblast division | EN-124k-90-group9299.Contig2 | | | | | | | | | | | | | |  |  |  |  |
| M | 0055059 | asymmetric neuroblast division | EN-124k-90-group9299.Contig3 | | | | | | | | | | | | | |  |  |  |  |
| M | 0055059 | asymmetric neuroblast division | EN-124k-90-group9317.Contig1 | | | | | | | | | | | | | |  |  |  |  |
| M | 0055059 | asymmetric neuroblast division | EN-124k-90-group9388.Contig2 | | | | | | | | | | | | | |  |  |  |  |
| M | 0055059 | asymmetric neuroblast division | EN-124k-90-group9446.Contig1 | | | | | | | | | | | | | |  |  |  |  |
| M | 0055059 | asymmetric neuroblast division | EN-124k-90-group9479.Contig1 | | | | | | | | | | | | | |  |  |  |  |
| M | 0055059 | asymmetric neuroblast division | EN-124k-90-group9650.Contig1 | | | | | | | | | | | | | |  |  |  |  |
| M | 0055059 | asymmetric neuroblast division | EN-124k-90-group9695.Contig1 | | | | | | | | | | | | | |  |  |  |  |
| M | 0055059 | asymmetric neuroblast division | EN-124k-90-group9819.Contig1 | | | | | | | | | | | | | |  |  |  |  |
| M | 0055059 | asymmetric neuroblast division | EN-124k-90-group9843.Contig1 | | | | | | | | | | | | | |  |  |  |  |
| M | 0055059 | asymmetric neuroblast division | EN-124k-90-group10032.Contig1 | | | | | | | | | | | | | | |  |  |  |
| M | 0055059 | asymmetric neuroblast division | EN-124k-90-group10085.Contig1 | | | | | | | | | | | | | | |  |  |  |
| M | 0055059 | asymmetric neuroblast division | EN-124k-90-group10085.Contig2 | | | | | | | | | | | | | | |  |  |  |
| M | 0055059 | asymmetric neuroblast division | EN-124k-90-group10090.Contig2 | | | | | | | | | | | | | | |  |  |  |
| M | 0055059 | asymmetric neuroblast division | EN-124k-90-group10221.Contig1 | | | | | | | | | | | | | | |  |  |  |
| M | 0055059 | asymmetric neuroblast division | EN-124k-90-group10276.Contig4 | | | | | | | | | | | | | | |  |  |  |
| M | 0055059 | asymmetric neuroblast division | EN-124k-90-group10598.Contig1 | | | | | | | | | | | | | | |  |  |  |
| M | 0055059 | asymmetric neuroblast division | EN-124k-90-group10652.Contig1 | | | | | | | | | | | | | | |  |  |  |
| M | 0055059 | asymmetric neuroblast division | EN-124k-90-group10667.Contig1 | | | | | | | | | | | | | | |  |  |  |
| M | 0055059 | asymmetric neuroblast division | EN-124k-90-group10717.Contig1 | | | | | | | | | | | | | | |  |  |  |
| M | 0055059 | asymmetric neuroblast division | EN-124k-90-group10790.Contig1 | | | | | | | | | | | | | | |  |  |  |
| M | 0055059 | asymmetric neuroblast division | EN-124k-90-group10824.Contig1 | | | | | | | | | | | | | | |  |  |  |
| M | 0055059 | asymmetric neuroblast division | EN-124k-90-group10838.Contig2 | | | | | | | | | | | | | | |  |  |  |
| M | 0055059 | asymmetric neuroblast division | EN-124k-90-group10881.Contig1 | | | | | | | | | | | | | | |  |  |  |
| M | 0055059 | asymmetric neuroblast division | EN-124k-90-group10891.Contig1 | | | | | | | | | | | | | | |  |  |  |
| M | 0055059 | asymmetric neuroblast division | EN-124k-90-group10891.Contig2 | | | | | | | | | | | | | | |  |  |  |
| M | 0055059 | asymmetric neuroblast division | EN-124k-90-group10912.Contig1 | | | | | | | | | | | | | | |  |  |  |
| M | 0055059 | asymmetric neuroblast division | EN-124k-90-group10928.Contig1 | | | | | | | | | | | | | | |  |  |  |
| M | 0055059 | asymmetric neuroblast division | EN-124k-90-group11235.Contig1 | | | | | | | | | | | | | | |  |  |  |
| M | 0055059 | asymmetric neuroblast division | EN-124k-90-group11250.Contig1 | | | | | | | | | | | | | | |  |  |  |
| M | 0055059 | asymmetric neuroblast division | EN-124k-90-group11408.Contig1 | | | | | | | | | | | | | | |  |  |  |
| M | 0055059 | asymmetric neuroblast division | EN-124k-90-group11686.Contig1 | | | | | | | | | | | | | | |  |  |  |
| M | 0055059 | asymmetric neuroblast division | EN-124k-90-group11808.Contig1 | | | | | | | | | | | | | | |  |  |  |
| M | 0055059 | asymmetric neuroblast division | EN-124k-90-group11987.Contig1 | | | | | | | | | | | | | | |  |  |  |
| E | 0055059 | asymmetric neuroblast division | EN-124k-90-group12010.Contig1 | | | | | | | | | | | | | | |  |  |  |
| E | 0055059 | asymmetric neuroblast division | EN-124k-90-group12010.Contig2 | | | | | | | | | | | | | | |  |  |  |
| M | 0055059 | asymmetric neuroblast division | EN-124k-90-group12099.Contig1 | | | | | | | | | | | | | | |  |  |  |
| M | 0055059 | asymmetric neuroblast division | EN-124k-90-group12519.Contig1 | | | | | | | | | | | | | | |  |  |  |
| M | 0055059 | asymmetric neuroblast division | EN-124k-90-group12678.Contig1 | | | | | | | | | | | | | | |  |  |  |
| M | 0055059 | asymmetric neuroblast division | EN-124k-90-group12973.Contig1 | | | | | | | | | | | | | | |  |  |  |
| M | 0055059 | asymmetric neuroblast division | EN-124k-90-group13121.Contig1 | | | | | | | | | | | | | | |  |  |  |
| M | 0055059 | asymmetric neuroblast division | EN-124k-90-group13235.Contig1 | | | | | | | | | | | | | | |  |  |  |
| M | 0055059 | asymmetric neuroblast division | EN-124k-90-group13502.Contig1 | | | | | | | | | | | | | | |  |  |  |
| M | 0055059 | asymmetric neuroblast division | EN-124k-90-group13502.Contig2 | | | | | | | | | | | | | | |  |  |  |
| M | 0055059 | asymmetric neuroblast division | EN-124k-90-group13754.Contig1 | | | | | | | | | | | | | | |  |  |  |
| E | 0055059 | asymmetric neuroblast division | EN-124k-90-group14464.Contig1 | | | | | | | | | | | | | | |  |  |  |
| M | 0055059 | asymmetric neuroblast division | EN-124k-90-group14708.Contig1 | | | | | | | | | | | | | | |  |  |  |
| M | 0055059 | asymmetric neuroblast division | EN-124k-90-group15300.Contig1 | | | | | | | | | | | | | | |  |  |  |
| A | 0055059 | asymmetric neuroblast division | EN-124k-90-group230.gs\_25885 | | | | | | | | | | | | | | |  |  |  |
| A | 0055059 | asymmetric neuroblast division | EN-124k-90-group574.gs\_59096 | | | | | | | | | | | | | | |  |  |  |
| E | 0055059 | asymmetric neuroblast division | EN-124k-90-group701.jgi\_contig\_JGI\_CBBP10955\_fwd | | | | | | | | | | | | | | | | | |
| E | 0055059 | asymmetric neuroblast division | EN-124k-90-group701.jgi\_contig\_JGI\_CBBP5477\_fwd | | | | | | | | | | | | | | | | | |
| E | 0055059 | asymmetric neuroblast division | EN-124k-90-group701.EN\_iowa\_8242 | | | | | | | | | | | | | | |  |  |  |
| A | 0055059 | asymmetric neuroblast division | EN-124k-90-group701.gs\_14861 | | | | | | | | | | | | | | |  |  |  |
| E | 0055059 | asymmetric neuroblast division | EN-124k-90-group900.EN\_iowa\_15436 | | | | | | | | | | | | | | |  |  |  |
| A | 0055059 | asymmetric neuroblast division | EN-124k-90-group900.gs\_31783 | | | | | | | | | | | | | | |  |  |  |
| A | 0055059 | asymmetric neuroblast division | EN-124k-90-group900.gs\_71447 | | | | | | | | | | | | | | |  |  |  |
| A | 0055059 | asymmetric neuroblast division | EN-124k-90-group1153.gs\_635 | | | | | | | | | | | | | |  |  |  |  |
| A | 0055059 | asymmetric neuroblast division | EN-124k-90-group1153.gs\_33239 | | | | | | | | | | | | | | |  |  |  |
| E | 0055059 | asymmetric neuroblast division | EN-124k-90-group1493.jgi\_contig\_JGI\_CBBP6493\_fwd | | | | | | | | | | | | | | | | | |
| E | 0055059 | asymmetric neuroblast division | EN-124k-90-group1560.jgi\_contig\_JGI\_CBBP5129\_fwd | | | | | | | | | | | | | | | | | |
| A | 0055059 | asymmetric neuroblast division | EN-124k-90-group1782.gs\_16663 | | | | | | | | | | | | | | |  |  |  |
| A | 0055059 | asymmetric neuroblast division | EN-124k-90-group2069.gs\_85543 | | | | | | | | | | | | | | |  |  |  |
| A | 0055059 | asymmetric neuroblast division | EN-124k-90-group2069.gs\_63060 | | | | | | | | | | | | | | |  |  |  |
| A | 0055059 | asymmetric neuroblast division | EN-124k-90-group2069.gs\_69693 | | | | | | | | | | | | | | |  |  |  |
| E | 0055059 | asymmetric neuroblast division | EN-124k-90-group2077.jgi\_paired\_JGI\_CBBP13793\_rev | | | | | | | | | | | | | | | | | |
| E | 0055059 | asymmetric neuroblast division | EN-124k-90-group2409.EN\_iowa\_9419 | | | | | | | | | | | | | | |  |  |  |
| E | 0055059 | asymmetric neuroblast division | EN-124k-90-group2409.EN\_iowa\_12940 | | | | | | | | | | | | | | | |  |  |
| A | 0055059 | asymmetric neuroblast division | EN-124k-90-group3522.gs\_9768 | | | | | | | | | | | | | | |  |  |  |
| A | 0055059 | asymmetric neuroblast division | EN-124k-90-group3713.gs\_7861 | | | | | | | | | | | | | | |  |  |  |
| A | 0055059 | asymmetric neuroblast division | EN-124k-90-group4349.gs\_42524 | | | | | | | | | | | | | | |  |  |  |
| E | 0055059 | asymmetric neuroblast division | EN-124k-90-group4804.jgi\_contig\_JGI\_CBBP4923\_fwd | | | | | | | | | | | | | | | | | |
| A | 0055059 | asymmetric neuroblast division | EN-124k-90-group4804.gs\_72028 | | | | | | | | | | | | | | |  |  |  |
| E | 0055059 | asymmetric neuroblast division | EN-124k-90-group4945.jgi\_contig\_JGI\_CBBP14472\_fwd | | | | | | | | | | | | | | | | | |
| E | 0055059 | asymmetric neuroblast division | EN-124k-90-group4945.jgi\_contig\_JGI\_CBBP13066\_fwd | | | | | | | | | | | | | | | | | |
| A | 0055059 | asymmetric neuroblast division | EN-124k-90-group4945.gs\_26726 | | | | | | | | | | | | | | |  |  |  |
| E | 0055059 | asymmetric neuroblast division | EN-124k-90-group4945.jgi\_contig\_JGI\_CBBP19558\_fwd | | | | | | | | | | | | | | | | | |
| A | 0055059 | asymmetric neuroblast division | EN-124k-90-group4945.gs\_49527 | | | | | | | | | | | | | | |  |  |  |
| E | 0055059 | asymmetric neuroblast division | EN-124k-90-group5607.jgi\_contig\_JGI\_CBBP445\_fwd | | | | | | | | | | | | | | | | | |
| E | 0055059 | asymmetric neuroblast division | EN-124k-90-group6204.EN\_iowa\_1799 | | | | | | | | | | | | | | |  |  |  |
| E | 0055059 | asymmetric neuroblast division | EN-124k-90-group6204.EN\_iowa\_4716 | | | | | | | | | | | | | | |  |  |  |
| E | 0055059 | asymmetric neuroblast division | EN-124k-90-group6923.jgi\_contig\_JGI\_CBBP17789\_fwd | | | | | | | | | | | | | | | | | |
| A | 0055059 | asymmetric neuroblast division | EN-124k-90-group7440.gs\_75782 | | | | | | | | | | | | | | |  |  |  |
| E | 0055059 | asymmetric neuroblast division | EN-124k-90-group7875.jgi\_paired\_JGI\_CBBP10776\_rev | | | | | | | | | | | | | | | | | |
| E | 0055059 | asymmetric neuroblast division | EN-124k-90-group8221.jgi\_contig\_JGI\_CBBP18338\_fwd | | | | | | | | | | | | | | | | | |
| A | 0055059 | asymmetric neuroblast division | EN-124k-90-group8306.gs\_61906 | | | | | | | | | | | | | | |  |  |  |
| A | 0055059 | asymmetric neuroblast division | EN-124k-90-group9299.gs\_87064 | | | | | | | | | | | | | | |  |  |  |
| A | 0007413 | axonal fasciculation | EN-124k-90-group10674.gs\_45324 | | | | | | | | | | | | | | |  |  |  |
| A | 0007413 | axonal fasciculation | EN-124k-90-group807.gs\_71281 | | | | | | | | | | | | | | |  |  |  |
| A | 0007413 | axonal fasciculation | EN-124k-90-group1020.gs\_69762 | | | | | | | | | | | | | | |  |  |  |
| E | 0007413 | axonal fasciculation | EN-124k-90-group1177.jgi\_unpaired\_JGI\_CBBP6661\_fwd | | | | | | | | | | | | | | | | | |
| E | 0007413 | axonal fasciculation | EN-124k-90-group1306.EN\_iowa\_2354 | | | | | | | | | | | | | | |  |  |  |
| E | 0007413 | axonal fasciculation | EN-124k-90-group1825.jgi\_contig\_JGI\_CBBP14395\_fwd | | | | | | | | | | | | | | | | | |
| E | 0007413 | axonal fasciculation | EN-124k-90-group2916.jgi\_paired\_JGI\_CBBP2065\_fwd | | | | | | | | | | | | | | | | | |
| A | 0007413 | axonal fasciculation | EN-124k-90-group2932.gs\_32728 | | | | | | | | | | | | | | |  |  |  |
| A | 0007413 | axonal fasciculation | EN-124k-90-group3420.gs\_43284 | | | | | | | | | | | | | | |  |  |  |
| E | 0007413 | axonal fasciculation | EN-124k-90-group3448.jgi\_paired\_JGI\_CBBP19729\_fwd | | | | | | | | | | | | | | | | | |
| A | 0007413 | axonal fasciculation | EN-124k-90-group3807.gs\_52991 | | | | | | | | | | | | | | |  |  |  |
| E | 0007413 | axonal fasciculation | EN-124k-90-group5117.jgi\_paired\_JGI\_CBBP19153\_fwd | | | | | | | | | | | | | | | | | |
| A | 0007413 | axonal fasciculation | EN-124k-90-group5423.gs\_20381 | | | | | | | | | | | | | | |  |  |  |
| E | 0007413 | axonal fasciculation | EN-124k-90-group5834.jgi\_contig\_JGI\_CBBP12701\_fwd | | | | | | | | | | | | | | | | | |
| E | 0007413 | axonal fasciculation | EN-124k-90-group6116.EN\_iowa\_7333 | | | | | | | | | | | | | | |  |  |  |
| E | 0007413 | axonal fasciculation | EN-124k-90-group6144.EN\_iowa\_5856 | | | | | | | | | | | | | | |  |  |  |
| E | 0007413 | axonal fasciculation | EN-124k-90-group6737.jgi\_contig\_JGI\_CBBP17820\_fwd | | | | | | | | | | | | | | | | | |
| E | 0007413 | axonal fasciculation | EN-124k-90-group6849.EN\_iowa\_8480 | | | | | | | | | | | | | | |  |  |  |
| A | 0007413 | axonal fasciculation | EN-124k-90-group7595.gs\_36261 | | | | | | | | | | | | | | |  |  |  |
| A | 0007413 | axonal fasciculation | EN-124k-90-group7953.gs\_11284 | | | | | | | | | | | | | | |  |  |  |
| E | 0007413 | axonal fasciculation | EN-124k-90-group8104.jgi\_paired\_JGI\_CBBP9893\_fwd | | | | | | | | | | | | | | | | | |
| E | 0007413 | axonal fasciculation | EN-124k-90-group8107.jgi\_paired\_JGI\_CBBP6197\_fwd | | | | | | | | | | | | | | | | | |
| A | 0007413 | axonal fasciculation | EN-124k-90-group8310.gs\_24913 | | | | | | | | | | | | | | |  |  |  |
| A | 0007413 | axonal fasciculation | EN-124k-90-group8658.gs\_22315 | | | | | | | | | | | | | | |  |  |  |
| E | 0007413 | axonal fasciculation | EN-124k-90-group8721.jgi\_paired\_JGI\_CBBP11443\_rev | | | | | | | | | | | | | | | | | |
| E | 0007413 | axonal fasciculation | EN-124k-90-group9286.jgi\_paired\_JGI\_CBBP4063\_fwd | | | | | | | | | | | | | | | | | |
| A | 0007413 | axonal fasciculation | EN-124k-90-group9442.gs\_10851 | | | | | | | | | | | | | | |  |  |  |
| E | 0007413 | axonal fasciculation | EN-124k-90-group9448.jgi\_paired\_JGI\_CBBP4366\_rev | | | | | | | | | | | | | | | | | |
| A | 0007413 | axonal fasciculation | EN-124k-90-group9467.gs\_16039 | | | | | | | | | | | | | | |  |  |  |
| A | 0007413 | axonal fasciculation | EN-124k-90-group9657.gs\_43696 | | | | | | | | | | | | | | |  |  |  |
| E | 0007413 | axonal fasciculation | EN-124k-90-group9773.jgi\_paired\_JGI\_CBBP6585\_rev | | | | | | | | | | | | | | | | | |
| E | 0007413 | axonal fasciculation | EN-124k-90-group9900.jgi\_paired\_JGI\_CBBP17759\_fwd | | | | | | | | | | | | | | | | | |
| E | 0007413 | axonal fasciculation | EN-124k-90-group10230.jgi\_paired\_JGI\_CBBP3918\_fwd | | | | | | | | | | | | | | | | | |
| A | 0007413 | axonal fasciculation | EN-124k-90-group10312.gs\_22624 | | | | | | | | | | | | | | |  |  |  |
| E | 0007413 | axonal fasciculation | EN-124k-90-group10416.jgi\_contig\_JGI\_CBBP3274\_fwd | | | | | | | | | | | | | | | | | |
| A | 0007413 | axonal fasciculation | EN-124k-90-group10635.gs\_47150 | | | | | | | | | | | | | | |  |  |  |
| E | 0007413 | axonal fasciculation | EN-124k-90-group10878.jgi\_paired\_JGI\_CBBP952\_rev | | | | | | | | | | | | | | | | | |
| A | 0007413 | axonal fasciculation | EN-124k-90-group10935.gs\_64995 | | | | | | | | | | | | | | |  |  |  |
| E | 0007413 | axonal fasciculation | EN-124k-90-group11108.jgi\_contig\_JGI\_CBBP10361\_fwd | | | | | | | | | | | | | | | | | |
| E | 0007413 | axonal fasciculation | EN-124k-90-group11156.EN\_iowa\_9243 | | | | | | | | | | | | | | | |  |  |
| A | 0007413 | axonal fasciculation | EN-124k-90-group11354.gs\_78099 | | | | | | | | | | | | | | |  |  |  |
| A | 0007413 | axonal fasciculation | EN-124k-90-group11387.gs\_16543 | | | | | | | | | | | | | | |  |  |  |
| E | 0007413 | axonal fasciculation | EN-124k-90-group11469.jgi\_paired\_JGI\_CBBP7559\_fwd | | | | | | | | | | | | | | | | | |
| E | 0007413 | axonal fasciculation | EN-124k-90-group11545.jgi\_paired\_JGI\_CBBP2808\_fwd | | | | | | | | | | | | | | | | | |
| E | 0007413 | axonal fasciculation | EN-124k-90-group11647.EN\_iowa\_13292 | | | | | | | | | | | | | | | |  |  |
| A | 0007413 | axonal fasciculation | EN-124k-90-group11738.gs\_53113 | | | | | | | | | | | | | | |  |  |  |
| E | 0007413 | axonal fasciculation | EN-124k-90-group11780.jgi\_paired\_JGI\_CBBP12332\_fwd | | | | | | | | | | | | | | | | | |
| E | 0007413 | axonal fasciculation | EN-124k-90-group12012.jgi\_contig\_JGI\_CBBP10480\_fwd | | | | | | | | | | | | | | | | | |
| E | 0007413 | axonal fasciculation | EN-124k-90-group12452.EN\_iowa\_9722 | | | | | | | | | | | | | | | |  |  |
| E | 0007413 | axonal fasciculation | EN-124k-90-group12523.jgi\_unpaired\_JGI\_CBBP6872\_fwd | | | | | | | | | | | | | | | | | |
| E | 0007413 | axonal fasciculation | EN-124k-90-group12537.jgi\_contig\_JGI\_CBBP17605\_fwd | | | | | | | | | | | | | | | | | |
| E | 0007413 | axonal fasciculation | EN-124k-90-group12595.jgi\_contig\_JGI\_CBBP15955\_fwd | | | | | | | | | | | | | | | | | |
| A | 0007413 | axonal fasciculation | EN-124k-90-group12740.gs\_56121 | | | | | | | | | | | | | | |  |  |  |
| A | 0007413 | axonal fasciculation | EN-124k-90-group12946.gs\_86900 | | | | | | | | | | | | | | |  |  |  |
| A | 0007413 | axonal fasciculation | EN-124k-90-group13006.gs\_33814 | | | | | | | | | | | | | | |  |  |  |
| E | 0007413 | axonal fasciculation | EN-124k-90-group13146.jgi\_paired\_JGI\_CBBP17160\_fwd | | | | | | | | | | | | | | | | | |
| A | 0007413 | axonal fasciculation | EN-124k-90-group13187.gs\_23330 | | | | | | | | | | | | | | |  |  |  |
| E | 0007413 | axonal fasciculation | EN-124k-90-group13466.jgi\_paired\_JGI\_CBBP5821\_fwd | | | | | | | | | | | | | | | | | |
| A | 0007413 | axonal fasciculation | EN-124k-90-group13506.gs\_86402 | | | | | | | | | | | | | | |  |  |  |
| A | 0007413 | axonal fasciculation | EN-124k-90-group13537.gs\_73717 | | | | | | | | | | | | | | |  |  |  |
| E | 0007413 | axonal fasciculation | EN-124k-90-group13736.jgi\_paired\_JGI\_CBBP15489\_rev | | | | | | | | | | | | | | | | | |
| E | 0007413 | axonal fasciculation | EN-124k-90-group13770.jgi\_paired\_JGI\_CBBP10036\_fwd | | | | | | | | | | | | | | | | | |
| A | 0007413 | axonal fasciculation | EN-124k-90-group13777.gs\_40847 | | | | | | | | | | | | | | |  |  |  |
| A | 0007413 | axonal fasciculation | EN-124k-90-group13823.gs\_32410 | | | | | | | | | | | | | | |  |  |  |
| A | 0007413 | axonal fasciculation | EN-124k-90-group13900.gs\_16949 | | | | | | | | | | | | | | |  |  |  |
| A | 0007413 | axonal fasciculation | EN-124k-90-group14128.gs\_38861 | | | | | | | | | | | | | | |  |  |  |
| E | 0007413 | axonal fasciculation | EN-124k-90-group14335.jgi\_paired\_JGI\_CBBP12938\_fwd | | | | | | | | | | | | | | | | | |
| A | 0007413 | axonal fasciculation | EN-124k-90-group14336.gs\_71984 | | | | | | | | | | | | | | |  |  |  |
| A | 0007413 | axonal fasciculation | EN-124k-90-group14498.gs\_52406 | | | | | | | | | | | | | | |  |  |  |
| A | 0007413 | axonal fasciculation | EN-124k-90-group14558.gs\_28342 | | | | | | | | | | | | | | |  |  |  |
| A | 0007413 | axonal fasciculation | EN-124k-90-group14563.gs\_48486 | | | | | | | | | | | | | | |  |  |  |
| A | 0007413 | axonal fasciculation | EN-124k-90-group14582.gs\_17139 | | | | | | | | | | | | | | |  |  |  |
| E | 0007413 | axonal fasciculation | EN-124k-90-group14826.jgi\_paired\_JGI\_CBBP20200\_fwd | | | | | | | | | | | | | | | | | |
| A | 0007413 | axonal fasciculation | EN-124k-90-group14891.gs\_33740 | | | | | | | | | | | | | | |  |  |  |
| A | 0007413 | axonal fasciculation | EN-124k-90-group14896.gs\_55224 | | | | | | | | | | | | | | |  |  |  |
| A | 0007413 | axonal fasciculation | EN-124k-90-group14948.gs\_26769 | | | | | | | | | | | | | | |  |  |  |
| E | 0007413 | axonal fasciculation | EN-124k-90-group14953.EN\_iowa\_3925 | | | | | | | | | | | | | | | |  |  |
| E | 0007413 | axonal fasciculation | EN-124k-90-group14960.jgi\_paired\_JGI\_CBBP2642\_fwd | | | | | | | | | | | | | | | | | |
| A | 0007413 | axonal fasciculation | EN-124k-90-group15062.gs\_40000 | | | | | | | | | | | | | | |  |  |  |
| A | 0007413 | axonal fasciculation | EN-124k-90-group15124.gs\_82644 | | | | | | | | | | | | | | |  |  |  |
| A | 0007413 | axonal fasciculation | EN-124k-90-group15268.gs\_21540 | | | | | | | | | | | | | | |  |  |  |
| E | 0007413 | axonal fasciculation | EN-124k-90-group15315.EN\_iowa\_6410 | | | | | | | | | | | | | | | |  |  |
| E | 0007413 | axonal fasciculation | EN-124k-90-group15320.jgi\_paired\_JGI\_CBBP9817\_fwd | | | | | | | | | | | | | | | | | |
| E | 0007413 | axonal fasciculation | EN-124k-90-group15486.jgi\_paired\_JGI\_CBBP15354\_fwd | | | | | | | | | | | | | | | | | |
| E | 0007413 | axonal fasciculation | EN-124k-90-group15532.EN\_iowa\_5883 | | | | | | | | | | | | | | | |  |  |
| A | 0007413 | axonal fasciculation | EN-124k-90-group15589.gs\_17337 | | | | | | | | | | | | | | |  |  |  |
| E | 0007413 | axonal fasciculation | EN-124k-90-group15692.jgi\_contig\_JGI\_CBBP12510\_fwd | | | | | | | | | | | | | | | | | |
| E | 0007413 | axonal fasciculation | EN-124k-90-group15723.jgi\_paired\_JGI\_CBBP16981\_fwd | | | | | | | | | | | | | | | | | |
| A | 0007413 | axonal fasciculation | EN-124k-90-group15770.gs\_45569 | | | | | | | | | | | | | | |  |  |  |
| A | 0007413 | axonal fasciculation | EN-124k-90-group15856.gs\_15241 | | | | | | | | | | | | | | |  |  |  |
| E | 0007413 | axonal fasciculation | EN-124k-90-group15940.jgi\_contig\_JGI\_CBBP11433\_fwd | | | | | | | | | | | | | | | | | |
| A | 0007413 | axonal fasciculation | EN-124k-90-group16026.gs\_15943 | | | | | | | | | | | | | | |  |  |  |
| A | 0007413 | axonal fasciculation | EN-124k-90-group16087.gs\_81197 | | | | | | | | | | | | | | |  |  |  |
| E | 0007413 | axonal fasciculation | EN-124k-90-group16134.jgi\_contig\_JGI\_CBBP14519\_fwd | | | | | | | | | | | | | | | | | |
| A | 0007413 | axonal fasciculation | EN-124k-90-group16149.gs\_13439 | | | | | | | | | | | | | | |  |  |  |
| A | 0007413 | axonal fasciculation | EN-124k-90-group16189.gs\_70159 | | | | | | | | | | | | | | |  |  |  |
| E | 0007413 | axonal fasciculation | EN-124k-90-group16200.jgi\_paired\_JGI\_CBBP10444\_rev | | | | | | | | | | | | | | | | | |
| E | 0007413 | axonal fasciculation | EN-124k-90-group16264.jgi\_paired\_JGI\_CBBP10036\_rev | | | | | | | | | | | | | | | | | |
| A | 0007413 | axonal fasciculation | EN-124k-90-group16285.gs\_47129 | | | | | | | | | | | | | | |  |  |  |
| A | 0007413 | axonal fasciculation | EN-124k-90-group16291.gs\_65588 | | | | | | | | | | | | | | |  |  |  |
| E | 0007413 | axonal fasciculation | EN-124k-90-group16346.jgi\_contig\_JGI\_CBBP6214\_fwd | | | | | | | | | | | | | | | | | |
| A | 0007413 | axonal fasciculation | EN-124k-90-group16416.gs\_28586 | | | | | | | | | | | | | | |  |  |  |
| A | 0007413 | axonal fasciculation | EN-124k-90-group16423.gs\_20626 | | | | | | | | | | | | | | |  |  |  |
| A | 0007413 | axonal fasciculation | EN-124k-90-group16443.gs\_57863 | | | | | | | | | | | | | | |  |  |  |
| A | 0007413 | axonal fasciculation | EN-124k-90-group16489.gs\_80464 | | | | | | | | | | | | | | |  |  |  |
| A | 0007413 | axonal fasciculation | EN-124k-90-group16520.gs\_62867 | | | | | | | | | | | | | | |  |  |  |
| A | 0007413 | axonal fasciculation | EN-124k-90-group16523.gs\_75670 | | | | | | | | | | | | | | |  |  |  |
| A | 0007413 | axonal fasciculation | EN-124k-90-group16636.gs\_429 | | | | | | | | | | | | | | |  |  |  |
| E | 0007413 | axonal fasciculation | EN-124k-90-group16800.EN\_iowa\_4640 | | | | | | | | | | | | | | | |  |  |
| A | 0007413 | axonal fasciculation | EN-124k-90-group16830.gs\_82512 | | | | | | | | | | | | | | |  |  |  |
| A | 0007413 | axonal fasciculation | EN-124k-90-group16837.gs\_15971 | | | | | | | | | | | | | | |  |  |  |
| A | 0007413 | axonal fasciculation | EN-124k-90-group16910.gs\_22871 | | | | | | | | | | | | | | |  |  |  |
| A | 0007413 | axonal fasciculation | EN-124k-90-group17193.gs\_13341 | | | | | | | | | | | | | | |  |  |  |
| A | 0007413 | axonal fasciculation | EN-124k-90-group17338.gs\_21850 | | | | | | | | | | | | | | |  |  |  |
| A | 0007413 | axonal fasciculation | EN-124k-90-group17386.gs\_68411 | | | | | | | | | | | | | | |  |  |  |
| A | 0007413 | axonal fasciculation | EN-124k-90-group17411.gs\_13940 | | | | | | | | | | | | | | |  |  |  |
| A | 0007413 | axonal fasciculation | EN-124k-90-group17442.gs\_63748 | | | | | | | | | | | | | | |  |  |  |
| A | 0007413 | axonal fasciculation | EN-124k-90-group17445.gs\_83255 | | | | | | | | | | | | | | |  |  |  |
| A | 0007413 | axonal fasciculation | EN-124k-90-group17487.gs\_82041 | | | | | | | | | | | | | | |  |  |  |
| A | 0007413 | axonal fasciculation | EN-124k-90-group17524.gs\_80802 | | | | | | | | | | | | | | |  |  |  |
| A | 0007413 | axonal fasciculation | EN-124k-90-group17563.gs\_80410 | | | | | | | | | | | | | | |  |  |  |
| A | 0007413 | axonal fasciculation | EN-124k-90-group17572.gs\_7187 | | | | | | | | | | | | | | |  |  |  |
| A | 0007413 | axonal fasciculation | EN-124k-90-group17582.gs\_70636 | | | | | | | | | | | | | | |  |  |  |
| A | 0007413 | axonal fasciculation | EN-124k-90-group17650.gs\_59359 | | | | | | | | | | | | | | |  |  |  |
| A | 0007413 | axonal fasciculation | EN-124k-90-group17702.gs\_18827 | | | | | | | | | | | | | | |  |  |  |
| A | 0007413 | axonal fasciculation | EN-124k-90-group17706.gs\_80669 | | | | | | | | | | | | | | |  |  |  |
| A | 0007413 | axonal fasciculation | EN-124k-90-group17930.gs\_83513 | | | | | | | | | | | | | | |  |  |  |
| E | 0007413 | axonal fasciculation | EN-124k-90-group17979.jgi\_contig\_JGI\_CBBP2421\_fwd | | | | | | | | | | | | | | | | | |
| A | 0007413 | axonal fasciculation | EN-124k-90-group18038.gs\_30262 | | | | | | | | | | | | | | |  |  |  |
| A | 0007413 | axonal fasciculation | EN-124k-90-group18140.gs\_87168 | | | | | | | | | | | | | | |  |  |  |
| A | 0007413 | axonal fasciculation | EN-124k-90-group18238.gs\_39782 | | | | | | | | | | | | | | |  |  |  |
| A | 0007413 | axonal fasciculation | EN-124k-90-group18289.gs\_45417 | | | | | | | | | | | | | | |  |  |  |
| A | 0007413 | axonal fasciculation | EN-124k-90-group18324.gs\_57299 | | | | | | | | | | | | | | |  |  |  |
| A | 0007413 | axonal fasciculation | EN-124k-90-group18342.gs\_65612 | | | | | | | | | | | | | | |  |  |  |
| E | 0007413 | axonal fasciculation | EN-124k-90-group18538.jgi\_paired\_JGI\_CBBP19133\_rev | | | | | | | | | | | | | | | | | |
| A | 0007413 | axonal fasciculation | EN-124k-90-group18592.gs\_31083 | | | | | | | | | | | | | | |  |  |  |
| A | 0007413 | axonal fasciculation | EN-124k-90-group18618.gs\_55759 | | | | | | | | | | | | | | |  |  |  |
| A | 0007413 | axonal fasciculation | EN-124k-90-group18774.gs\_57540 | | | | | | | | | | | | | | |  |  |  |
| A | 0007413 | axonal fasciculation | EN-124k-90-group18929.gs\_13816 | | | | | | | | | | | | | | |  |  |  |
| M | 0007413 | axonal fasciculation | EN-124k-90-group59.Contig1 | | | | | | | | | | | | | |  |  |  |  |
| M | 0007413 | axonal fasciculation | EN-124k-90-group115.Contig1 | | | | | | | | | | | | | |  |  |  |  |
| M | 0007413 | axonal fasciculation | EN-124k-90-group115.Contig2 | | | | | | | | | | | | | |  |  |  |  |
| M | 0007413 | axonal fasciculation | EN-124k-90-group138.Contig1 | | | | | | | | | | | | | |  |  |  |  |
| M | 0007413 | axonal fasciculation | EN-124k-90-group138.Contig62 | | | | | | | | | | | | | |  |  |  |  |
| M | 0007413 | axonal fasciculation | EN-124k-90-group229.Contig1 | | | | | | | | | | | | | |  |  |  |  |
| M | 0007413 | axonal fasciculation | EN-124k-90-group230.Contig1 | | | | | | | | | | | | | |  |  |  |  |
| M | 0007413 | axonal fasciculation | EN-124k-90-group232.Contig1 | | | | | | | | | | | | | |  |  |  |  |
| M | 0007413 | axonal fasciculation | EN-124k-90-group232.Contig2 | | | | | | | | | | | | | |  |  |  |  |
| M | 0007413 | axonal fasciculation | EN-124k-90-group232.Contig3 | | | | | | | | | | | | | |  |  |  |  |
| M | 0007413 | axonal fasciculation | EN-124k-90-group232.Contig5 | | | | | | | | | | | | | |  |  |  |  |
| M | 0007413 | axonal fasciculation | EN-124k-90-group253.Contig1 | | | | | | | | | | | | | |  |  |  |  |
| M | 0007413 | axonal fasciculation | EN-124k-90-group270.Contig2 | | | | | | | | | | | | | |  |  |  |  |
| M | 0007413 | axonal fasciculation | EN-124k-90-group290.Contig5 | | | | | | | | | | | | | |  |  |  |  |
| M | 0007413 | axonal fasciculation | EN-124k-90-group290.Contig6 | | | | | | | | | | | | | |  |  |  |  |
| M | 0007413 | axonal fasciculation | EN-124k-90-group414.Contig1 | | | | | | | | | | | | | |  |  |  |  |
| M | 0007413 | axonal fasciculation | EN-124k-90-group414.Contig2 | | | | | | | | | | | | | |  |  |  |  |
| M | 0007413 | axonal fasciculation | EN-124k-90-group414.Contig3 | | | | | | | | | | | | | |  |  |  |  |
| M | 0007413 | axonal fasciculation | EN-124k-90-group430.Contig1 | | | | | | | | | | | | | |  |  |  |  |
| M | 0007413 | axonal fasciculation | EN-124k-90-group506.Contig1 | | | | | | | | | | | | | |  |  |  |  |
| M | 0007413 | axonal fasciculation | EN-124k-90-group506.Contig3 | | | | | | | | | | | | | |  |  |  |  |
| M | 0007413 | axonal fasciculation | EN-124k-90-group506.Contig4 | | | | | | | | | | | | | |  |  |  |  |
| M | 0007413 | axonal fasciculation | EN-124k-90-group518.Contig2 | | | | | | | | | | | | | |  |  |  |  |
| M | 0007413 | axonal fasciculation | EN-124k-90-group524.Contig3 | | | | | | | | | | | | | |  |  |  |  |
| M | 0007413 | axonal fasciculation | EN-124k-90-group529.Contig2 | | | | | | | | | | | | | |  |  |  |  |
| M | 0007413 | axonal fasciculation | EN-124k-90-group550.Contig2 | | | | | | | | | | | | | |  |  |  |  |
| M | 0007413 | axonal fasciculation | EN-124k-90-group564.Contig5 | | | | | | | | | | | | | |  |  |  |  |
| M | 0007413 | axonal fasciculation | EN-124k-90-group586.Contig2 | | | | | | | | | | | | | |  |  |  |  |
| M | 0007413 | axonal fasciculation | EN-124k-90-group630.Contig2 | | | | | | | | | | | | | |  |  |  |  |
| M | 0007413 | axonal fasciculation | EN-124k-90-group682.Contig1 | | | | | | | | | | | | | |  |  |  |  |
| M | 0007413 | axonal fasciculation | EN-124k-90-group689.Contig4 | | | | | | | | | | | | | |  |  |  |  |
| M | 0007413 | axonal fasciculation | EN-124k-90-group727.Contig5 | | | | | | | | | | | | | |  |  |  |  |
| M | 0007413 | axonal fasciculation | EN-124k-90-group735.Contig1 | | | | | | | | | | | | | |  |  |  |  |
| M | 0007413 | axonal fasciculation | EN-124k-90-group750.Contig1 | | | | | | | | | | | | | |  |  |  |  |
| M | 0007413 | axonal fasciculation | EN-124k-90-group750.Contig4 | | | | | | | | | | | | | |  |  |  |  |
| M | 0007413 | axonal fasciculation | EN-124k-90-group750.Contig6 | | | | | | | | | | | | | |  |  |  |  |
| M | 0007413 | axonal fasciculation | EN-124k-90-group750.Contig7 | | | | | | | | | | | | | |  |  |  |  |
| M | 0007413 | axonal fasciculation | EN-124k-90-group850.Contig1 | | | | | | | | | | | | | |  |  |  |  |
| M | 0007413 | axonal fasciculation | EN-124k-90-group881.Contig2 | | | | | | | | | | | | | |  |  |  |  |
| M | 0007413 | axonal fasciculation | EN-124k-90-group881.Contig3 | | | | | | | | | | | | | |  |  |  |  |
| M | 0007413 | axonal fasciculation | EN-124k-90-group882.Contig2 | | | | | | | | | | | | | |  |  |  |  |
| M | 0007413 | axonal fasciculation | EN-124k-90-group900.Contig1 | | | | | | | | | | | | | |  |  |  |  |
| M | 0007413 | axonal fasciculation | EN-124k-90-group908.Contig6 | | | | | | | | | | | | | |  |  |  |  |
| M | 0007413 | axonal fasciculation | EN-124k-90-group944.Contig1 | | | | | | | | | | | | | |  |  |  |  |
| M | 0007413 | axonal fasciculation | EN-124k-90-group944.Contig2 | | | | | | | | | | | | | |  |  |  |  |
| M | 0007413 | axonal fasciculation | EN-124k-90-group944.Contig4 | | | | | | | | | | | | | |  |  |  |  |
| M | 0007413 | axonal fasciculation | EN-124k-90-group944.Contig5 | | | | | | | | | | | | | |  |  |  |  |
| M | 0007413 | axonal fasciculation | EN-124k-90-group944.Contig6 | | | | | | | | | | | | | |  |  |  |  |
| M | 0007413 | axonal fasciculation | EN-124k-90-group944.Contig7 | | | | | | | | | | | | | |  |  |  |  |
| M | 0007413 | axonal fasciculation | EN-124k-90-group944.Contig8 | | | | | | | | | | | | | |  |  |  |  |
| M | 0007413 | axonal fasciculation | EN-124k-90-group944.Contig9 | | | | | | | | | | | | | |  |  |  |  |
| M | 0007413 | axonal fasciculation | EN-124k-90-group944.Contig11 | | | | | | | | | | | | | |  |  |  |  |
| M | 0007413 | axonal fasciculation | EN-124k-90-group944.Contig13 | | | | | | | | | | | | | |  |  |  |  |
| M | 0007413 | axonal fasciculation | EN-124k-90-group944.Contig14 | | | | | | | | | | | | | |  |  |  |  |
| M | 0007413 | axonal fasciculation | EN-124k-90-group944.Contig15 | | | | | | | | | | | | | |  |  |  |  |
| M | 0007413 | axonal fasciculation | EN-124k-90-group945.Contig1 | | | | | | | | | | | | | |  |  |  |  |
| M | 0007413 | axonal fasciculation | EN-124k-90-group994.Contig1 | | | | | | | | | | | | | |  |  |  |  |
| M | 0007413 | axonal fasciculation | EN-124k-90-group998.Contig1 | | | | | | | | | | | | | |  |  |  |  |
| M | 0007413 | axonal fasciculation | EN-124k-90-group1106.Contig1 | | | | | | | | | | | | | |  |  |  |  |
| M | 0007413 | axonal fasciculation | EN-124k-90-group1135.Contig1 | | | | | | | | | | | | | |  |  |  |  |
| M | 0007413 | axonal fasciculation | EN-124k-90-group1135.Contig4 | | | | | | | | | | | | | |  |  |  |  |
| M | 0007413 | axonal fasciculation | EN-124k-90-group1135.Contig5 | | | | | | | | | | | | | |  |  |  |  |
| M | 0007413 | axonal fasciculation | EN-124k-90-group1135.Contig7 | | | | | | | | | | | | | |  |  |  |  |
| M | 0007413 | axonal fasciculation | EN-124k-90-group1161.Contig1 | | | | | | | | | | | | | |  |  |  |  |
| M | 0007413 | axonal fasciculation | EN-124k-90-group1161.Contig2 | | | | | | | | | | | | | |  |  |  |  |
| M | 0007413 | axonal fasciculation | EN-124k-90-group1272.Contig1 | | | | | | | | | | | | | |  |  |  |  |
| M | 0007413 | axonal fasciculation | EN-124k-90-group1294.Contig1 | | | | | | | | | | | | | |  |  |  |  |
| M | 0007413 | axonal fasciculation | EN-124k-90-group1302.Contig5 | | | | | | | | | | | | | |  |  |  |  |
| M | 0007413 | axonal fasciculation | EN-124k-90-group1304.Contig2 | | | | | | | | | | | | | |  |  |  |  |
| M | 0007413 | axonal fasciculation | EN-124k-90-group1304.Contig4 | | | | | | | | | | | | | |  |  |  |  |
| M | 0007413 | axonal fasciculation | EN-124k-90-group1304.Contig8 | | | | | | | | | | | | | |  |  |  |  |
| M | 0007413 | axonal fasciculation | EN-124k-90-group1351.Contig1 | | | | | | | | | | | | | |  |  |  |  |
| M | 0007413 | axonal fasciculation | EN-124k-90-group1423.Contig14 | | | | | | | | | | | | | | |  |  |  |
| M | 0007413 | axonal fasciculation | EN-124k-90-group1423.Contig22 | | | | | | | | | | | | | | |  |  |  |
| M | 0007413 | axonal fasciculation | EN-124k-90-group1456.Contig7 | | | | | | | | | | | | | |  |  |  |  |
| M | 0007413 | axonal fasciculation | EN-124k-90-group1462.Contig1 | | | | | | | | | | | | | |  |  |  |  |
| A | 0007413 | axonal fasciculation | EN-124k-90-group1482.Contig1 | | | | | | | | | | | | | |  |  |  |  |
| A | 0007413 | axonal fasciculation | EN-124k-90-group1521.Contig1 | | | | | | | | | | | | | |  |  |  |  |
| A | 0007413 | axonal fasciculation | EN-124k-90-group1521.Contig3 | | | | | | | | | | | | | |  |  |  |  |
| M | 0007413 | axonal fasciculation | EN-124k-90-group1567.Contig1 | | | | | | | | | | | | | |  |  |  |  |
| M | 0007413 | axonal fasciculation | EN-124k-90-group1578.Contig1 | | | | | | | | | | | | | |  |  |  |  |
| M | 0007413 | axonal fasciculation | EN-124k-90-group1578.Contig2 | | | | | | | | | | | | | |  |  |  |  |
| M | 0007413 | axonal fasciculation | EN-124k-90-group1578.Contig3 | | | | | | | | | | | | | |  |  |  |  |
| M | 0007413 | axonal fasciculation | EN-124k-90-group1626.Contig1 | | | | | | | | | | | | | |  |  |  |  |
| M | 0007413 | axonal fasciculation | EN-124k-90-group1654.Contig1 | | | | | | | | | | | | | |  |  |  |  |
| M | 0007413 | axonal fasciculation | EN-124k-90-group1654.Contig2 | | | | | | | | | | | | | |  |  |  |  |
| M | 0007413 | axonal fasciculation | EN-124k-90-group1657.Contig1 | | | | | | | | | | | | | |  |  |  |  |
| M | 0007413 | axonal fasciculation | EN-124k-90-group1735.Contig6 | | | | | | | | | | | | | |  |  |  |  |
| M | 0007413 | axonal fasciculation | EN-124k-90-group1762.Contig1 | | | | | | | | | | | | | |  |  |  |  |
| M | 0007413 | axonal fasciculation | EN-124k-90-group1770.Contig2 | | | | | | | | | | | | | |  |  |  |  |
| M | 0007413 | axonal fasciculation | EN-124k-90-group1782.Contig1 | | | | | | | | | | | | | |  |  |  |  |
| M | 0007413 | axonal fasciculation | EN-124k-90-group1798.Contig2 | | | | | | | | | | | | | |  |  |  |  |
| M | 0007413 | axonal fasciculation | EN-124k-90-group1798.Contig3 | | | | | | | | | | | | | |  |  |  |  |
| M | 0007413 | axonal fasciculation | EN-124k-90-group1799.Contig1 | | | | | | | | | | | | | |  |  |  |  |
| M | 0007413 | axonal fasciculation | EN-124k-90-group1799.Contig2 | | | | | | | | | | | | | |  |  |  |  |
| M | 0007413 | axonal fasciculation | EN-124k-90-group1799.Contig3 | | | | | | | | | | | | | |  |  |  |  |
| M | 0007413 | axonal fasciculation | EN-124k-90-group1799.Contig5 | | | | | | | | | | | | | |  |  |  |  |
| M | 0007413 | axonal fasciculation | EN-124k-90-group1799.Contig7 | | | | | | | | | | | | | |  |  |  |  |
| M | 0007413 | axonal fasciculation | EN-124k-90-group1799.Contig8 | | | | | | | | | | | | | |  |  |  |  |
| M | 0007413 | axonal fasciculation | EN-124k-90-group1799.Contig9 | | | | | | | | | | | | | |  |  |  |  |
| M | 0007413 | axonal fasciculation | EN-124k-90-group1819.Contig3 | | | | | | | | | | | | | |  |  |  |  |
| M | 0007413 | axonal fasciculation | EN-124k-90-group1834.Contig1 | | | | | | | | | | | | | |  |  |  |  |
| M | 0007413 | axonal fasciculation | EN-124k-90-group1861.Contig1 | | | | | | | | | | | | | |  |  |  |  |
| M | 0007413 | axonal fasciculation | EN-124k-90-group1861.Contig2 | | | | | | | | | | | | | |  |  |  |  |
| M | 0007413 | axonal fasciculation | EN-124k-90-group1867.Contig1 | | | | | | | | | | | | | |  |  |  |  |
| M | 0007413 | axonal fasciculation | EN-124k-90-group1907.Contig1 | | | | | | | | | | | | | |  |  |  |  |
| M | 0007413 | axonal fasciculation | EN-124k-90-group1909.Contig3 | | | | | | | | | | | | | |  |  |  |  |
| M | 0007413 | axonal fasciculation | EN-124k-90-group1915.Contig1 | | | | | | | | | | | | | |  |  |  |  |
| M | 0007413 | axonal fasciculation | EN-124k-90-group1958.Contig1 | | | | | | | | | | | | | |  |  |  |  |
| M | 0007413 | axonal fasciculation | EN-124k-90-group1960.Contig1 | | | | | | | | | | | | | |  |  |  |  |
| M | 0007413 | axonal fasciculation | EN-124k-90-group2021.Contig2 | | | | | | | | | | | | | |  |  |  |  |
| E | 0007413 | axonal fasciculation | EN-124k-90-group2041.Contig1 | | | | | | | | | | | | | |  |  |  |  |
| M | 0007413 | axonal fasciculation | EN-124k-90-group2076.Contig1 | | | | | | | | | | | | | |  |  |  |  |
| M | 0007413 | axonal fasciculation | EN-124k-90-group2081.Contig1 | | | | | | | | | | | | | |  |  |  |  |
| M | 0007413 | axonal fasciculation | EN-124k-90-group2081.Contig3 | | | | | | | | | | | | | |  |  |  |  |
| M | 0007413 | axonal fasciculation | EN-124k-90-group2097.Contig1 | | | | | | | | | | | | | |  |  |  |  |
| M | 0007413 | axonal fasciculation | EN-124k-90-group2200.Contig1 | | | | | | | | | | | | | |  |  |  |  |
| M | 0007413 | axonal fasciculation | EN-124k-90-group2428.Contig1 | | | | | | | | | | | | | |  |  |  |  |
| M | 0007413 | axonal fasciculation | EN-124k-90-group2449.Contig1 | | | | | | | | | | | | | |  |  |  |  |
| M | 0007413 | axonal fasciculation | EN-124k-90-group2449.Contig2 | | | | | | | | | | | | | |  |  |  |  |
| M | 0007413 | axonal fasciculation | EN-124k-90-group2451.Contig1 | | | | | | | | | | | | | |  |  |  |  |
| M | 0007413 | axonal fasciculation | EN-124k-90-group2455.Contig1 | | | | | | | | | | | | | |  |  |  |  |
| M | 0007413 | axonal fasciculation | EN-124k-90-group2472.Contig2 | | | | | | | | | | | | | |  |  |  |  |
| M | 0007413 | axonal fasciculation | EN-124k-90-group2472.Contig3 | | | | | | | | | | | | | |  |  |  |  |
| M | 0007413 | axonal fasciculation | EN-124k-90-group2495.Contig1 | | | | | | | | | | | | | |  |  |  |  |
| M | 0007413 | axonal fasciculation | EN-124k-90-group2495.Contig2 | | | | | | | | | | | | | |  |  |  |  |
| M | 0007413 | axonal fasciculation | EN-124k-90-group2498.Contig5 | | | | | | | | | | | | | |  |  |  |  |
| M | 0007413 | axonal fasciculation | EN-124k-90-group2537.Contig1 | | | | | | | | | | | | | |  |  |  |  |
| M | 0007413 | axonal fasciculation | EN-124k-90-group2540.Contig1 | | | | | | | | | | | | | |  |  |  |  |
| M | 0007413 | axonal fasciculation | EN-124k-90-group2540.Contig2 | | | | | | | | | | | | | |  |  |  |  |
| M | 0007413 | axonal fasciculation | EN-124k-90-group2582.Contig1 | | | | | | | | | | | | | |  |  |  |  |
| M | 0007413 | axonal fasciculation | EN-124k-90-group2584.Contig2 | | | | | | | | | | | | | |  |  |  |  |
| A | 0007413 | axonal fasciculation | EN-124k-90-group2585.Contig1 | | | | | | | | | | | | | |  |  |  |  |
| M | 0007413 | axonal fasciculation | EN-124k-90-group2606.Contig1 | | | | | | | | | | | | | |  |  |  |  |
| M | 0007413 | axonal fasciculation | EN-124k-90-group2681.Contig1 | | | | | | | | | | | | | |  |  |  |  |
| M | 0007413 | axonal fasciculation | EN-124k-90-group2686.Contig1 | | | | | | | | | | | | | |  |  |  |  |
| M | 0007413 | axonal fasciculation | EN-124k-90-group2834.Contig2 | | | | | | | | | | | | | |  |  |  |  |
| M | 0007413 | axonal fasciculation | EN-124k-90-group2835.Contig2 | | | | | | | | | | | | | |  |  |  |  |
| M | 0007413 | axonal fasciculation | EN-124k-90-group2852.Contig1 | | | | | | | | | | | | | |  |  |  |  |
| M | 0007413 | axonal fasciculation | EN-124k-90-group2903.Contig1 | | | | | | | | | | | | | |  |  |  |  |
| M | 0007413 | axonal fasciculation | EN-124k-90-group2917.Contig1 | | | | | | | | | | | | | |  |  |  |  |
| M | 0007413 | axonal fasciculation | EN-124k-90-group2917.Contig2 | | | | | | | | | | | | | |  |  |  |  |
| M | 0007413 | axonal fasciculation | EN-124k-90-group2917.Contig3 | | | | | | | | | | | | | |  |  |  |  |
| M | 0007413 | axonal fasciculation | EN-124k-90-group2917.Contig4 | | | | | | | | | | | | | |  |  |  |  |
| M | 0007413 | axonal fasciculation | EN-124k-90-group2940.Contig2 | | | | | | | | | | | | | |  |  |  |  |
| M | 0007413 | axonal fasciculation | EN-124k-90-group2959.Contig1 | | | | | | | | | | | | | |  |  |  |  |
| M | 0007413 | axonal fasciculation | EN-124k-90-group2959.Contig3 | | | | | | | | | | | | | |  |  |  |  |
| M | 0007413 | axonal fasciculation | EN-124k-90-group2975.Contig1 | | | | | | | | | | | | | |  |  |  |  |
| M | 0007413 | axonal fasciculation | EN-124k-90-group3082.Contig1 | | | | | | | | | | | | | |  |  |  |  |
| M | 0007413 | axonal fasciculation | EN-124k-90-group3082.Contig2 | | | | | | | | | | | | | |  |  |  |  |
| M | 0007413 | axonal fasciculation | EN-124k-90-group3135.Contig1 | | | | | | | | | | | | | |  |  |  |  |
| M | 0007413 | axonal fasciculation | EN-124k-90-group3135.Contig2 | | | | | | | | | | | | | |  |  |  |  |
| M | 0007413 | axonal fasciculation | EN-124k-90-group3223.Contig2 | | | | | | | | | | | | | |  |  |  |  |
| M | 0007413 | axonal fasciculation | EN-124k-90-group3224.Contig4 | | | | | | | | | | | | | |  |  |  |  |
| M | 0007413 | axonal fasciculation | EN-124k-90-group3224.Contig5 | | | | | | | | | | | | | |  |  |  |  |
| M | 0007413 | axonal fasciculation | EN-124k-90-group3292.Contig1 | | | | | | | | | | | | | |  |  |  |  |
| M | 0007413 | axonal fasciculation | EN-124k-90-group3299.Contig1 | | | | | | | | | | | | | |  |  |  |  |
| M | 0007413 | axonal fasciculation | EN-124k-90-group3313.Contig1 | | | | | | | | | | | | | |  |  |  |  |
| M | 0007413 | axonal fasciculation | EN-124k-90-group3313.Contig3 | | | | | | | | | | | | | |  |  |  |  |
| M | 0007413 | axonal fasciculation | EN-124k-90-group3315.Contig6 | | | | | | | | | | | | | |  |  |  |  |
| M | 0007413 | axonal fasciculation | EN-124k-90-group3343.Contig1 | | | | | | | | | | | | | |  |  |  |  |
| M | 0007413 | axonal fasciculation | EN-124k-90-group3394.Contig1 | | | | | | | | | | | | | |  |  |  |  |
| M | 0007413 | axonal fasciculation | EN-124k-90-group3394.Contig2 | | | | | | | | | | | | | |  |  |  |  |
| M | 0007413 | axonal fasciculation | EN-124k-90-group3394.Contig3 | | | | | | | | | | | | | |  |  |  |  |
| M | 0007413 | axonal fasciculation | EN-124k-90-group3394.Contig4 | | | | | | | | | | | | | |  |  |  |  |
| M | 0007413 | axonal fasciculation | EN-124k-90-group3401.Contig2 | | | | | | | | | | | | | |  |  |  |  |
| M | 0007413 | axonal fasciculation | EN-124k-90-group3477.Contig1 | | | | | | | | | | | | | |  |  |  |  |
| M | 0007413 | axonal fasciculation | EN-124k-90-group3479.Contig1 | | | | | | | | | | | | | |  |  |  |  |
| M | 0007413 | axonal fasciculation | EN-124k-90-group3511.Contig1 | | | | | | | | | | | | | |  |  |  |  |
| M | 0007413 | axonal fasciculation | EN-124k-90-group3511.Contig2 | | | | | | | | | | | | | |  |  |  |  |
| M | 0007413 | axonal fasciculation | EN-124k-90-group3522.Contig1 | | | | | | | | | | | | | |  |  |  |  |
| M | 0007413 | axonal fasciculation | EN-124k-90-group3567.Contig1 | | | | | | | | | | | | | |  |  |  |  |
| M | 0007413 | axonal fasciculation | EN-124k-90-group3567.Contig2 | | | | | | | | | | | | | |  |  |  |  |
| M | 0007413 | axonal fasciculation | EN-124k-90-group3567.Contig3 | | | | | | | | | | | | | |  |  |  |  |
| M | 0007413 | axonal fasciculation | EN-124k-90-group3582.Contig2 | | | | | | | | | | | | | |  |  |  |  |
| M | 0007413 | axonal fasciculation | EN-124k-90-group3586.Contig1 | | | | | | | | | | | | | |  |  |  |  |
| M | 0007413 | axonal fasciculation | EN-124k-90-group3594.Contig4 | | | | | | | | | | | | | |  |  |  |  |
| E | 0007413 | axonal fasciculation | EN-124k-90-group3611.Contig1 | | | | | | | | | | | | | |  |  |  |  |
| M | 0007413 | axonal fasciculation | EN-124k-90-group3630.Contig2 | | | | | | | | | | | | | |  |  |  |  |
| M | 0007413 | axonal fasciculation | EN-124k-90-group3700.Contig1 | | | | | | | | | | | | | |  |  |  |  |
| M | 0007413 | axonal fasciculation | EN-124k-90-group3713.Contig1 | | | | | | | | | | | | | |  |  |  |  |
| A | 0007413 | axonal fasciculation | EN-124k-90-group3737.Contig1 | | | | | | | | | | | | | |  |  |  |  |
| M | 0007413 | axonal fasciculation | EN-124k-90-group3816.Contig1 | | | | | | | | | | | | | |  |  |  |  |
| M | 0007413 | axonal fasciculation | EN-124k-90-group3882.Contig1 | | | | | | | | | | | | | |  |  |  |  |
| M | 0007413 | axonal fasciculation | EN-124k-90-group3897.Contig1 | | | | | | | | | | | | | |  |  |  |  |
| M | 0007413 | axonal fasciculation | EN-124k-90-group3916.Contig1 | | | | | | | | | | | | | |  |  |  |  |
| M | 0007413 | axonal fasciculation | EN-124k-90-group3933.Contig1 | | | | | | | | | | | | | |  |  |  |  |
| M | 0007413 | axonal fasciculation | EN-124k-90-group4009.Contig1 | | | | | | | | | | | | | |  |  |  |  |
| M | 0007413 | axonal fasciculation | EN-124k-90-group4045.Contig1 | | | | | | | | | | | | | |  |  |  |  |
| M | 0007413 | axonal fasciculation | EN-124k-90-group4046.Contig1 | | | | | | | | | | | | | |  |  |  |  |
| M | 0007413 | axonal fasciculation | EN-124k-90-group4049.Contig1 | | | | | | | | | | | | | |  |  |  |  |
| M | 0007413 | axonal fasciculation | EN-124k-90-group4168.Contig1 | | | | | | | | | | | | | |  |  |  |  |
| M | 0007413 | axonal fasciculation | EN-124k-90-group4168.Contig3 | | | | | | | | | | | | | |  |  |  |  |
| M | 0007413 | axonal fasciculation | EN-124k-90-group4183.Contig1 | | | | | | | | | | | | | |  |  |  |  |
| M | 0007413 | axonal fasciculation | EN-124k-90-group4194.Contig1 | | | | | | | | | | | | | |  |  |  |  |
| M | 0007413 | axonal fasciculation | EN-124k-90-group4216.Contig1 | | | | | | | | | | | | | |  |  |  |  |
| M | 0007413 | axonal fasciculation | EN-124k-90-group4349.Contig4 | | | | | | | | | | | | | |  |  |  |  |
| M | 0007413 | axonal fasciculation | EN-124k-90-group4351.Contig1 | | | | | | | | | | | | | |  |  |  |  |
| M | 0007413 | axonal fasciculation | EN-124k-90-group4372.Contig1 | | | | | | | | | | | | | |  |  |  |  |
| M | 0007413 | axonal fasciculation | EN-124k-90-group4410.Contig1 | | | | | | | | | | | | | |  |  |  |  |
| M | 0007413 | axonal fasciculation | EN-124k-90-group4432.Contig4 | | | | | | | | | | | | | |  |  |  |  |
| M | 0007413 | axonal fasciculation | EN-124k-90-group4477.Contig4 | | | | | | | | | | | | | |  |  |  |  |
| M | 0007413 | axonal fasciculation | EN-124k-90-group4562.Contig1 | | | | | | | | | | | | | |  |  |  |  |
| M | 0007413 | axonal fasciculation | EN-124k-90-group4600.Contig1 | | | | | | | | | | | | | |  |  |  |  |
| M | 0007413 | axonal fasciculation | EN-124k-90-group4616.Contig1 | | | | | | | | | | | | | |  |  |  |  |
| M | 0007413 | axonal fasciculation | EN-124k-90-group4753.Contig1 | | | | | | | | | | | | | |  |  |  |  |
| M | 0007413 | axonal fasciculation | EN-124k-90-group4759.Contig3 | | | | | | | | | | | | | |  |  |  |  |
| M | 0007413 | axonal fasciculation | EN-124k-90-group4763.Contig1 | | | | | | | | | | | | | |  |  |  |  |
| M | 0007413 | axonal fasciculation | EN-124k-90-group4827.Contig2 | | | | | | | | | | | | | |  |  |  |  |
| M | 0007413 | axonal fasciculation | EN-124k-90-group4929.Contig2 | | | | | | | | | | | | | |  |  |  |  |
| M | 0007413 | axonal fasciculation | EN-124k-90-group4944.Contig2 | | | | | | | | | | | | | |  |  |  |  |
| M | 0007413 | axonal fasciculation | EN-124k-90-group5036.Contig2 | | | | | | | | | | | | | |  |  |  |  |
| M | 0007413 | axonal fasciculation | EN-124k-90-group5040.Contig1 | | | | | | | | | | | | | |  |  |  |  |
| M | 0007413 | axonal fasciculation | EN-124k-90-group5040.Contig2 | | | | | | | | | | | | | |  |  |  |  |
| M | 0007413 | axonal fasciculation | EN-124k-90-group5055.Contig1 | | | | | | | | | | | | | |  |  |  |  |
| M | 0007413 | axonal fasciculation | EN-124k-90-group5055.Contig3 | | | | | | | | | | | | | |  |  |  |  |
| M | 0007413 | axonal fasciculation | EN-124k-90-group5063.Contig1 | | | | | | | | | | | | | |  |  |  |  |
| A | 0007413 | axonal fasciculation | EN-124k-90-group5155.Contig1 | | | | | | | | | | | | | |  |  |  |  |
| M | 0007413 | axonal fasciculation | EN-124k-90-group5170.Contig1 | | | | | | | | | | | | | |  |  |  |  |
| M | 0007413 | axonal fasciculation | EN-124k-90-group5170.Contig2 | | | | | | | | | | | | | |  |  |  |  |
| M | 0007413 | axonal fasciculation | EN-124k-90-group5170.Contig3 | | | | | | | | | | | | | |  |  |  |  |
| M | 0007413 | axonal fasciculation | EN-124k-90-group5172.Contig1 | | | | | | | | | | | | | |  |  |  |  |
| M | 0007413 | axonal fasciculation | EN-124k-90-group5173.Contig1 | | | | | | | | | | | | | |  |  |  |  |
| M | 0007413 | axonal fasciculation | EN-124k-90-group5203.Contig2 | | | | | | | | | | | | | |  |  |  |  |
| M | 0007413 | axonal fasciculation | EN-124k-90-group5215.Contig3 | | | | | | | | | | | | | |  |  |  |  |
| M | 0007413 | axonal fasciculation | EN-124k-90-group5252.Contig1 | | | | | | | | | | | | | |  |  |  |  |
| M | 0007413 | axonal fasciculation | EN-124k-90-group5273.Contig1 | | | | | | | | | | | | | |  |  |  |  |
| M | 0007413 | axonal fasciculation | EN-124k-90-group5273.Contig3 | | | | | | | | | | | | | |  |  |  |  |
| M | 0007413 | axonal fasciculation | EN-124k-90-group5298.Contig1 | | | | | | | | | | | | | |  |  |  |  |
| M | 0007413 | axonal fasciculation | EN-124k-90-group5329.Contig1 | | | | | | | | | | | | | |  |  |  |  |
| M | 0007413 | axonal fasciculation | EN-124k-90-group5398.Contig1 | | | | | | | | | | | | | |  |  |  |  |
| M | 0007413 | axonal fasciculation | EN-124k-90-group5479.Contig1 | | | | | | | | | | | | | |  |  |  |  |
| M | 0007413 | axonal fasciculation | EN-124k-90-group5491.Contig1 | | | | | | | | | | | | | |  |  |  |  |
| M | 0007413 | axonal fasciculation | EN-124k-90-group5491.Contig2 | | | | | | | | | | | | | |  |  |  |  |
| M | 0007413 | axonal fasciculation | EN-124k-90-group5532.Contig1 | | | | | | | | | | | | | |  |  |  |  |
| M | 0007413 | axonal fasciculation | EN-124k-90-group5532.Contig2 | | | | | | | | | | | | | |  |  |  |  |
| M | 0007413 | axonal fasciculation | EN-124k-90-group5541.Contig1 | | | | | | | | | | | | | |  |  |  |  |
| M | 0007413 | axonal fasciculation | EN-124k-90-group5567.Contig2 | | | | | | | | | | | | | |  |  |  |  |
| M | 0007413 | axonal fasciculation | EN-124k-90-group5567.Contig3 | | | | | | | | | | | | | |  |  |  |  |
| M | 0007413 | axonal fasciculation | EN-124k-90-group5607.Contig1 | | | | | | | | | | | | | |  |  |  |  |
| M | 0007413 | axonal fasciculation | EN-124k-90-group5616.Contig2 | | | | | | | | | | | | | |  |  |  |  |
| M | 0007413 | axonal fasciculation | EN-124k-90-group5616.Contig3 | | | | | | | | | | | | | |  |  |  |  |
| M | 0007413 | axonal fasciculation | EN-124k-90-group5709.Contig1 | | | | | | | | | | | | | |  |  |  |  |
| M | 0007413 | axonal fasciculation | EN-124k-90-group5719.Contig1 | | | | | | | | | | | | | |  |  |  |  |
| M | 0007413 | axonal fasciculation | EN-124k-90-group5817.Contig1 | | | | | | | | | | | | | |  |  |  |  |
| M | 0007413 | axonal fasciculation | EN-124k-90-group5968.Contig1 | | | | | | | | | | | | | |  |  |  |  |
| M | 0007413 | axonal fasciculation | EN-124k-90-group6033.Contig1 | | | | | | | | | | | | | |  |  |  |  |
| M | 0007413 | axonal fasciculation | EN-124k-90-group6033.Contig3 | | | | | | | | | | | | | |  |  |  |  |
| M | 0007413 | axonal fasciculation | EN-124k-90-group6038.Contig1 | | | | | | | | | | | | | |  |  |  |  |
| M | 0007413 | axonal fasciculation | EN-124k-90-group6104.Contig1 | | | | | | | | | | | | | |  |  |  |  |
| M | 0007413 | axonal fasciculation | EN-124k-90-group6104.Contig2 | | | | | | | | | | | | | |  |  |  |  |
| M | 0007413 | axonal fasciculation | EN-124k-90-group6147.Contig1 | | | | | | | | | | | | | |  |  |  |  |
| E | 0007413 | axonal fasciculation | EN-124k-90-group6238.Contig1 | | | | | | | | | | | | | |  |  |  |  |
| E | 0007413 | axonal fasciculation | EN-124k-90-group6326.Contig1 | | | | | | | | | | | | | |  |  |  |  |
| M | 0007413 | axonal fasciculation | EN-124k-90-group6358.Contig1 | | | | | | | | | | | | | |  |  |  |  |
| M | 0007413 | axonal fasciculation | EN-124k-90-group6358.Contig2 | | | | | | | | | | | | | |  |  |  |  |
| M | 0007413 | axonal fasciculation | EN-124k-90-group6400.Contig1 | | | | | | | | | | | | | |  |  |  |  |
| M | 0007413 | axonal fasciculation | EN-124k-90-group6472.Contig1 | | | | | | | | | | | | | |  |  |  |  |
| M | 0007413 | axonal fasciculation | EN-124k-90-group6472.Contig2 | | | | | | | | | | | | | |  |  |  |  |
| M | 0007413 | axonal fasciculation | EN-124k-90-group6591.Contig1 | | | | | | | | | | | | | |  |  |  |  |
| M | 0007413 | axonal fasciculation | EN-124k-90-group6647.Contig1 | | | | | | | | | | | | | |  |  |  |  |
| M | 0007413 | axonal fasciculation | EN-124k-90-group6809.Contig1 | | | | | | | | | | | | | |  |  |  |  |
| E | 0007413 | axonal fasciculation | EN-124k-90-group6858.Contig1 | | | | | | | | | | | | | |  |  |  |  |
| M | 0007413 | axonal fasciculation | EN-124k-90-group6923.Contig1 | | | | | | | | | | | | | |  |  |  |  |
| M | 0007413 | axonal fasciculation | EN-124k-90-group6923.Contig2 | | | | | | | | | | | | | |  |  |  |  |
| M | 0007413 | axonal fasciculation | EN-124k-90-group7010.Contig1 | | | | | | | | | | | | | |  |  |  |  |
| M | 0007413 | axonal fasciculation | EN-124k-90-group7013.Contig1 | | | | | | | | | | | | | |  |  |  |  |
| M | 0007413 | axonal fasciculation | EN-124k-90-group7017.Contig1 | | | | | | | | | | | | | |  |  |  |  |
| M | 0007413 | axonal fasciculation | EN-124k-90-group7107.Contig2 | | | | | | | | | | | | | |  |  |  |  |
| M | 0007413 | axonal fasciculation | EN-124k-90-group7149.Contig2 | | | | | | | | | | | | | |  |  |  |  |
| M | 0007413 | axonal fasciculation | EN-124k-90-group7157.Contig2 | | | | | | | | | | | | | |  |  |  |  |
| M | 0007413 | axonal fasciculation | EN-124k-90-group7165.Contig1 | | | | | | | | | | | | | |  |  |  |  |
| M | 0007413 | axonal fasciculation | EN-124k-90-group7231.Contig1 | | | | | | | | | | | | | |  |  |  |  |
| M | 0007413 | axonal fasciculation | EN-124k-90-group7234.Contig1 | | | | | | | | | | | | | |  |  |  |  |
| M | 0007413 | axonal fasciculation | EN-124k-90-group7343.Contig1 | | | | | | | | | | | | | |  |  |  |  |
| M | 0007413 | axonal fasciculation | EN-124k-90-group7440.Contig1 | | | | | | | | | | | | | |  |  |  |  |
| M | 0007413 | axonal fasciculation | EN-124k-90-group7457.Contig1 | | | | | | | | | | | | | |  |  |  |  |
| M | 0007413 | axonal fasciculation | EN-124k-90-group7460.Contig2 | | | | | | | | | | | | | |  |  |  |  |
| M | 0007413 | axonal fasciculation | EN-124k-90-group7463.Contig2 | | | | | | | | | | | | | |  |  |  |  |
| A | 0007413 | axonal fasciculation | EN-124k-90-group7650.Contig1 | | | | | | | | | | | | | |  |  |  |  |
| M | 0007413 | axonal fasciculation | EN-124k-90-group7740.Contig1 | | | | | | | | | | | | | |  |  |  |  |
| M | 0007413 | axonal fasciculation | EN-124k-90-group7740.Contig3 | | | | | | | | | | | | | |  |  |  |  |
| M | 0007413 | axonal fasciculation | EN-124k-90-group7871.Contig1 | | | | | | | | | | | | | |  |  |  |  |
| M | 0007413 | axonal fasciculation | EN-124k-90-group7964.Contig1 | | | | | | | | | | | | | |  |  |  |  |
| M | 0007413 | axonal fasciculation | EN-124k-90-group8079.Contig1 | | | | | | | | | | | | | |  |  |  |  |
| M | 0007413 | axonal fasciculation | EN-124k-90-group8079.Contig2 | | | | | | | | | | | | | |  |  |  |  |
| E | 0007413 | axonal fasciculation | EN-124k-90-group8101.Contig1 | | | | | | | | | | | | | |  |  |  |  |
| M | 0007413 | axonal fasciculation | EN-124k-90-group8131.Contig1 | | | | | | | | | | | | | |  |  |  |  |
| M | 0007413 | axonal fasciculation | EN-124k-90-group8174.Contig1 | | | | | | | | | | | | | |  |  |  |  |
| M | 0007413 | axonal fasciculation | EN-124k-90-group8174.Contig2 | | | | | | | | | | | | | |  |  |  |  |
| M | 0007413 | axonal fasciculation | EN-124k-90-group8211.Contig1 | | | | | | | | | | | | | |  |  |  |  |
| M | 0007413 | axonal fasciculation | EN-124k-90-group8253.Contig1 | | | | | | | | | | | | | |  |  |  |  |
| A | 0007413 | axonal fasciculation | EN-124k-90-group8519.Contig1 | | | | | | | | | | | | | |  |  |  |  |
| M | 0007413 | axonal fasciculation | EN-124k-90-group8524.Contig1 | | | | | | | | | | | | | |  |  |  |  |
| M | 0007413 | axonal fasciculation | EN-124k-90-group8568.Contig1 | | | | | | | | | | | | | |  |  |  |  |
| M | 0007413 | axonal fasciculation | EN-124k-90-group8645.Contig1 | | | | | | | | | | | | | |  |  |  |  |
| M | 0007413 | axonal fasciculation | EN-124k-90-group8696.Contig1 | | | | | | | | | | | | | |  |  |  |  |
| M | 0007413 | axonal fasciculation | EN-124k-90-group8799.Contig2 | | | | | | | | | | | | | |  |  |  |  |
| M | 0007413 | axonal fasciculation | EN-124k-90-group8850.Contig1 | | | | | | | | | | | | | |  |  |  |  |
| M | 0007413 | axonal fasciculation | EN-124k-90-group8897.Contig1 | | | | | | | | | | | | | |  |  |  |  |
| M | 0007413 | axonal fasciculation | EN-124k-90-group8937.Contig1 | | | | | | | | | | | | | |  |  |  |  |
| M | 0007413 | axonal fasciculation | EN-124k-90-group8937.Contig2 | | | | | | | | | | | | | |  |  |  |  |
| A | 0007413 | axonal fasciculation | EN-124k-90-group8957.Contig1 | | | | | | | | | | | | | |  |  |  |  |
| M | 0007413 | axonal fasciculation | EN-124k-90-group9165.Contig1 | | | | | | | | | | | | | |  |  |  |  |
| M | 0007413 | axonal fasciculation | EN-124k-90-group9168.Contig1 | | | | | | | | | | | | | |  |  |  |  |
| A | 0007413 | axonal fasciculation | EN-124k-90-group9245.Contig1 | | | | | | | | | | | | | |  |  |  |  |
| M | 0007413 | axonal fasciculation | EN-124k-90-group9278.Contig2 | | | | | | | | | | | | | |  |  |  |  |
| M | 0007413 | axonal fasciculation | EN-124k-90-group9299.Contig3 | | | | | | | | | | | | | |  |  |  |  |
| M | 0007413 | axonal fasciculation | EN-124k-90-group9317.Contig1 | | | | | | | | | | | | | |  |  |  |  |
| M | 0007413 | axonal fasciculation | EN-124k-90-group9388.Contig2 | | | | | | | | | | | | | |  |  |  |  |
| M | 0007413 | axonal fasciculation | EN-124k-90-group9446.Contig1 | | | | | | | | | | | | | |  |  |  |  |
| M | 0007413 | axonal fasciculation | EN-124k-90-group9485.Contig4 | | | | | | | | | | | | | |  |  |  |  |
| M | 0007413 | axonal fasciculation | EN-124k-90-group9506.Contig1 | | | | | | | | | | | | | |  |  |  |  |
| M | 0007413 | axonal fasciculation | EN-124k-90-group9517.Contig2 | | | | | | | | | | | | | |  |  |  |  |
| M | 0007413 | axonal fasciculation | EN-124k-90-group9771.Contig1 | | | | | | | | | | | | | |  |  |  |  |
| M | 0007413 | axonal fasciculation | EN-124k-90-group9771.Contig2 | | | | | | | | | | | | | |  |  |  |  |
| M | 0007413 | axonal fasciculation | EN-124k-90-group9819.Contig1 | | | | | | | | | | | | | |  |  |  |  |
| M | 0007413 | axonal fasciculation | EN-124k-90-group9887.Contig1 | | | | | | | | | | | | | |  |  |  |  |
| M | 0007413 | axonal fasciculation | EN-124k-90-group10085.Contig1 | | | | | | | | | | | | | | |  |  |  |
| M | 0007413 | axonal fasciculation | EN-124k-90-group10085.Contig2 | | | | | | | | | | | | | | |  |  |  |
| A | 0007413 | axonal fasciculation | EN-124k-90-group10370.Contig1 | | | | | | | | | | | | | | |  |  |  |
| M | 0007413 | axonal fasciculation | EN-124k-90-group10467.Contig1 | | | | | | | | | | | | | | |  |  |  |
| M | 0007413 | axonal fasciculation | EN-124k-90-group10630.Contig1 | | | | | | | | | | | | | | |  |  |  |
| M | 0007413 | axonal fasciculation | EN-124k-90-group10630.Contig2 | | | | | | | | | | | | | | |  |  |  |
| M | 0007413 | axonal fasciculation | EN-124k-90-group10652.Contig1 | | | | | | | | | | | | | | |  |  |  |
| E | 0007413 | axonal fasciculation | EN-124k-90-group10722.Contig1 | | | | | | | | | | | | | | |  |  |  |
| M | 0007413 | axonal fasciculation | EN-124k-90-group10759.Contig1 | | | | | | | | | | | | | | |  |  |  |
| M | 0007413 | axonal fasciculation | EN-124k-90-group10790.Contig1 | | | | | | | | | | | | | | |  |  |  |
| M | 0007413 | axonal fasciculation | EN-124k-90-group10838.Contig2 | | | | | | | | | | | | | | |  |  |  |
| M | 0007413 | axonal fasciculation | EN-124k-90-group10912.Contig1 | | | | | | | | | | | | | | |  |  |  |
| M | 0007413 | axonal fasciculation | EN-124k-90-group10928.Contig1 | | | | | | | | | | | | | | |  |  |  |
| M | 0007413 | axonal fasciculation | EN-124k-90-group10963.Contig1 | | | | | | | | | | | | | | |  |  |  |
| M | 0007413 | axonal fasciculation | EN-124k-90-group10990.Contig1 | | | | | | | | | | | | | | |  |  |  |
| E | 0007413 | axonal fasciculation | EN-124k-90-group11029.Contig1 | | | | | | | | | | | | | | |  |  |  |
| M | 0007413 | axonal fasciculation | EN-124k-90-group11131.Contig1 | | | | | | | | | | | | | | |  |  |  |
| M | 0007413 | axonal fasciculation | EN-124k-90-group11134.Contig1 | | | | | | | | | | | | | | |  |  |  |
| M | 0007413 | axonal fasciculation | EN-124k-90-group11235.Contig1 | | | | | | | | | | | | | | |  |  |  |
| M | 0007413 | axonal fasciculation | EN-124k-90-group11392.Contig1 | | | | | | | | | | | | | | |  |  |  |
| M | 0007413 | axonal fasciculation | EN-124k-90-group11392.Contig2 | | | | | | | | | | | | | | |  |  |  |
| M | 0007413 | axonal fasciculation | EN-124k-90-group11408.Contig1 | | | | | | | | | | | | | | |  |  |  |
| M | 0007413 | axonal fasciculation | EN-124k-90-group11707.Contig1 | | | | | | | | | | | | | | |  |  |  |
| M | 0007413 | axonal fasciculation | EN-124k-90-group11808.Contig1 | | | | | | | | | | | | | | |  |  |  |
| M | 0007413 | axonal fasciculation | EN-124k-90-group11810.Contig1 | | | | | | | | | | | | | | |  |  |  |
| M | 0007413 | axonal fasciculation | EN-124k-90-group12003.Contig1 | | | | | | | | | | | | | | |  |  |  |
| M | 0007413 | axonal fasciculation | EN-124k-90-group12099.Contig1 | | | | | | | | | | | | | | |  |  |  |
| M | 0007413 | axonal fasciculation | EN-124k-90-group12143.Contig1 | | | | | | | | | | | | | | |  |  |  |
| M | 0007413 | axonal fasciculation | EN-124k-90-group12337.Contig2 | | | | | | | | | | | | | | |  |  |  |
| M | 0007413 | axonal fasciculation | EN-124k-90-group12409.Contig1 | | | | | | | | | | | | | | |  |  |  |
| M | 0007413 | axonal fasciculation | EN-124k-90-group12409.Contig2 | | | | | | | | | | | | | | |  |  |  |
| A | 0007413 | axonal fasciculation | EN-124k-90-group12616.Contig1 | | | | | | | | | | | | | | |  |  |  |
| E | 0007413 | axonal fasciculation | EN-124k-90-group12653.Contig1 | | | | | | | | | | | | | | |  |  |  |
| M | 0007413 | axonal fasciculation | EN-124k-90-group13121.Contig1 | | | | | | | | | | | | | | |  |  |  |
| M | 0007413 | axonal fasciculation | EN-124k-90-group13235.Contig1 | | | | | | | | | | | | | | |  |  |  |
| M | 0007413 | axonal fasciculation | EN-124k-90-group13502.Contig2 | | | | | | | | | | | | | | |  |  |  |
| M | 0007413 | axonal fasciculation | EN-124k-90-group13646.Contig1 | | | | | | | | | | | | | | |  |  |  |
| M | 0007413 | axonal fasciculation | EN-124k-90-group13754.Contig1 | | | | | | | | | | | | | | |  |  |  |
| E | 0007413 | axonal fasciculation | EN-124k-90-group14241.Contig2 | | | | | | | | | | | | | | |  |  |  |
| M | 0007413 | axonal fasciculation | EN-124k-90-group14390.Contig2 | | | | | | | | | | | | | | |  |  |  |
| M | 0007413 | axonal fasciculation | EN-124k-90-group14708.Contig1 | | | | | | | | | | | | | | |  |  |  |
| E | 0007413 | axonal fasciculation | EN-124k-90-group15137.Contig1 | | | | | | | | | | | | | | |  |  |  |
| M | 0007413 | axonal fasciculation | EN-124k-90-group15757.Contig1 | | | | | | | | | | | | | | |  |  |  |
| E | 0007413 | axonal fasciculation | EN-124k-90-group15764.Contig1 | | | | | | | | | | | | | | |  |  |  |
| E | 0007413 | axonal fasciculation | EN-124k-90-group115.EN\_iowa\_11188 | | | | | | | | | | | | | | |  |  |  |
| A | 0007413 | axonal fasciculation | EN-124k-90-group230.gs\_25885 | | | | | | | | | | | | | | |  |  |  |
| E | 0007413 | axonal fasciculation | EN-124k-90-group232.jgi\_contig\_JGI\_CBBP16338\_fwd | | | | | | | | | | | | | | | | | |
| E | 0007413 | axonal fasciculation | EN-124k-90-group232.jgi\_contig\_JGI\_CBBP9823\_fwd | | | | | | | | | | | | | | | | | |
| E | 0007413 | axonal fasciculation | EN-124k-90-group232.jgi\_contig\_JGI\_CBBP10596\_fwd | | | | | | | | | | | | | | | | | |
| E | 0007413 | axonal fasciculation | EN-124k-90-group290.EN\_iowa\_14805 | | | | | | | | | | | | | | |  |  |  |
| E | 0007413 | axonal fasciculation | EN-124k-90-group414.EN\_iowa\_11198 | | | | | | | | | | | | | | |  |  |  |
| A | 0007413 | axonal fasciculation | EN-124k-90-group735.gs\_7849 | | | | | | | | | | | | | |  |  |  |  |
| E | 0007413 | axonal fasciculation | EN-124k-90-group850.EN\_iowa\_1547 | | | | | | | | | | | | | | |  |  |  |
| E | 0007413 | axonal fasciculation | EN-124k-90-group900.EN\_iowa\_15436 | | | | | | | | | | | | | | |  |  |  |
| A | 0007413 | axonal fasciculation | EN-124k-90-group900.gs\_31783 | | | | | | | | | | | | | | |  |  |  |
| A | 0007413 | axonal fasciculation | EN-124k-90-group900.gs\_71447 | | | | | | | | | | | | | | |  |  |  |
| E | 0007413 | axonal fasciculation | EN-124k-90-group944.EN\_iowa\_1330 | | | | | | | | | | | | | | |  |  |  |
| E | 0007413 | axonal fasciculation | EN-124k-90-group1135.jgi\_unpaired\_JGI\_CBBP19047\_fwd | | | | | | | | | | | | | | | | | |
| E | 0007413 | axonal fasciculation | EN-124k-90-group1135.jgi\_contig\_JGI\_CBBP19919\_fwd | | | | | | | | | | | | | | | | | |
| A | 0007413 | axonal fasciculation | EN-124k-90-group1135.gs\_2733 | | | | | | | | | | | | | | |  |  |  |
| A | 0007413 | axonal fasciculation | EN-124k-90-group1302.gs\_68057 | | | | | | | | | | | | | | |  |  |  |
| A | 0007413 | axonal fasciculation | EN-124k-90-group1351.gs\_80275 | | | | | | | | | | | | | | |  |  |  |
| A | 0007413 | axonal fasciculation | EN-124k-90-group1423.gs\_16014 | | | | | | | | | | | | | | |  |  |  |
| A | 0007413 | axonal fasciculation | EN-124k-90-group1423.gs\_11172 | | | | | | | | | | | | | | |  |  |  |
| A | 0007413 | axonal fasciculation | EN-124k-90-group1423.gs\_52362 | | | | | | | | | | | | | | |  |  |  |
| A | 0007413 | axonal fasciculation | EN-124k-90-group1423.gs\_75717 | | | | | | | | | | | | | | |  |  |  |
| E | 0007413 | axonal fasciculation | EN-124k-90-group1456.jgi\_contig\_JGI\_CBBP4193\_fwd | | | | | | | | | | | | | | | | | |
| A | 0007413 | axonal fasciculation | EN-124k-90-group1521.gs\_86350 | | | | | | | | | | | | | | |  |  |  |
| E | 0007413 | axonal fasciculation | EN-124k-90-group1626.jgi\_contig\_JGI\_CBBP20097\_fwd | | | | | | | | | | | | | | | | | |
| A | 0007413 | axonal fasciculation | EN-124k-90-group1626.gs\_84009 | | | | | | | | | | | | | | |  |  |  |
| E | 0007413 | axonal fasciculation | EN-124k-90-group1626.jgi\_contig\_JGI\_CBBP16438\_fwd | | | | | | | | | | | | | | | | | |
| A | 0007413 | axonal fasciculation | EN-124k-90-group1654.gs\_12044 | | | | | | | | | | | | | | |  |  |  |
| A | 0007413 | axonal fasciculation | EN-124k-90-group1782.gs\_16663 | | | | | | | | | | | | | | |  |  |  |
| A | 0007413 | axonal fasciculation | EN-124k-90-group1798.gs\_54312 | | | | | | | | | | | | | | |  |  |  |
| A | 0007413 | axonal fasciculation | EN-124k-90-group1798.gs\_39367 | | | | | | | | | | | | | | |  |  |  |
| E | 0007413 | axonal fasciculation | EN-124k-90-group1798.EN\_iowa\_16312 | | | | | | | | | | | | | | | |  |  |
| A | 0007413 | axonal fasciculation | EN-124k-90-group1798.gs\_29292 | | | | | | | | | | | | | | |  |  |  |
| A | 0007413 | axonal fasciculation | EN-124k-90-group1798.gs\_11661 | | | | | | | | | | | | | | |  |  |  |
| A | 0007413 | axonal fasciculation | EN-124k-90-group1798.gs\_36996 | | | | | | | | | | | | | | |  |  |  |
| A | 0007413 | axonal fasciculation | EN-124k-90-group1798.gs\_46615 | | | | | | | | | | | | | | |  |  |  |
| E | 0007413 | axonal fasciculation | EN-124k-90-group1798.EN\_iowa\_6796 | | | | | | | | | | | | | | |  |  |  |
| A | 0007413 | axonal fasciculation | EN-124k-90-group1798.gs\_56703 | | | | | | | | | | | | | | |  |  |  |
| A | 0007413 | axonal fasciculation | EN-124k-90-group1798.gs\_77511 | | | | | | | | | | | | | | |  |  |  |
| A | 0007413 | axonal fasciculation | EN-124k-90-group1798.gs\_54963 | | | | | | | | | | | | | | |  |  |  |
| A | 0007413 | axonal fasciculation | EN-124k-90-group1798.gs\_79284 | | | | | | | | | | | | | | |  |  |  |
| A | 0007413 | axonal fasciculation | EN-124k-90-group1798.gs\_4498 | | | | | | | | | | | | | | |  |  |  |
| A | 0007413 | axonal fasciculation | EN-124k-90-group1798.gs\_7178 | | | | | | | | | | | | | | |  |  |  |
| A | 0007413 | axonal fasciculation | EN-124k-90-group1798.gs\_7576 | | | | | | | | | | | | | | |  |  |  |
| A | 0007413 | axonal fasciculation | EN-124k-90-group1798.gs\_11790 | | | | | | | | | | | | | | |  |  |  |
| A | 0007413 | axonal fasciculation | EN-124k-90-group1798.gs\_50785 | | | | | | | | | | | | | | |  |  |  |
| A | 0007413 | axonal fasciculation | EN-124k-90-group1798.gs\_55765 | | | | | | | | | | | | | | |  |  |  |
| E | 0007413 | axonal fasciculation | EN-124k-90-group1799.EN\_iowa\_15365 | | | | | | | | | | | | | | | |  |  |
| A | 0007413 | axonal fasciculation | EN-124k-90-group1799.gs\_13071 | | | | | | | | | | | | | | |  |  |  |
| A | 0007413 | axonal fasciculation | EN-124k-90-group1799.gs\_20064 | | | | | | | | | | | | | | |  |  |  |
| A | 0007413 | axonal fasciculation | EN-124k-90-group1799.gs\_76593 | | | | | | | | | | | | | | |  |  |  |
| A | 0007413 | axonal fasciculation | EN-124k-90-group1799.gs\_77643 | | | | | | | | | | | | | | |  |  |  |
| A | 0007413 | axonal fasciculation | EN-124k-90-group1799.gs\_38929 | | | | | | | | | | | | | | |  |  |  |
| E | 0007413 | axonal fasciculation | EN-124k-90-group1799.EN\_iowa\_9582 | | | | | | | | | | | | | | |  |  |  |
| A | 0007413 | axonal fasciculation | EN-124k-90-group1799.gs\_3745 | | | | | | | | | | | | | | |  |  |  |
| E | 0007413 | axonal fasciculation | EN-124k-90-group1799.EN\_iowa\_16390 | | | | | | | | | | | | | | | |  |  |
| E | 0007413 | axonal fasciculation | EN-124k-90-group1819.jgi\_contig\_JGI\_CBBP4948\_fwd | | | | | | | | | | | | | | | | | |
| A | 0007413 | axonal fasciculation | EN-124k-90-group2021.gs\_23180 | | | | | | | | | | | | | | |  |  |  |
| E | 0007413 | axonal fasciculation | EN-124k-90-group2076.jgi\_contig\_JGI\_CBBP929\_fwd | | | | | | | | | | | | | | | | | |
| A | 0007413 | axonal fasciculation | EN-124k-90-group2076.gs\_5951 | | | | | | | | | | | | | | |  |  |  |
| A | 0007413 | axonal fasciculation | EN-124k-90-group2076.gs\_13876 | | | | | | | | | | | | | | |  |  |  |
| A | 0007413 | axonal fasciculation | EN-124k-90-group2076.gs\_6163 | | | | | | | | | | | | | | |  |  |  |
| A | 0007413 | axonal fasciculation | EN-124k-90-group2449.gs\_35369 | | | | | | | | | | | | | | |  |  |  |
| E | 0007413 | axonal fasciculation | EN-124k-90-group2451.jgi\_contig\_JGI\_CBBP13284\_fwd | | | | | | | | | | | | | | | | | |
| A | 0007413 | axonal fasciculation | EN-124k-90-group2903.gs\_16952 | | | | | | | | | | | | | | |  |  |  |
| E | 0007413 | axonal fasciculation | EN-124k-90-group2959.jgi\_contig\_JGI\_CBBP3203\_fwd | | | | | | | | | | | | | | | | | |
| E | 0007413 | axonal fasciculation | EN-124k-90-group2959.jgi\_contig\_JGI\_CBBP16696\_fwd | | | | | | | | | | | | | | | | | |
| E | 0007413 | axonal fasciculation | EN-124k-90-group2959.jgi\_contig\_JGI\_CBBP5581\_fwd | | | | | | | | | | | | | | | | | |
| A | 0007413 | axonal fasciculation | EN-124k-90-group3394.gs\_42372 | | | | | | | | | | | | | | |  |  |  |
| A | 0007413 | axonal fasciculation | EN-124k-90-group3522.gs\_9768 | | | | | | | | | | | | | | |  |  |  |
| E | 0007413 | axonal fasciculation | EN-124k-90-group3582.jgi\_contig\_JGI\_CBBP13041\_fwd | | | | | | | | | | | | | | | | | |
| A | 0007413 | axonal fasciculation | EN-124k-90-group3594.gs\_29852 | | | | | | | | | | | | | | |  |  |  |
| E | 0007413 | axonal fasciculation | EN-124k-90-group3611.EN\_iowa\_4463 | | | | | | | | | | | | | | |  |  |  |
| A | 0007413 | axonal fasciculation | EN-124k-90-group3630.gs\_19342 | | | | | | | | | | | | | | |  |  |  |
| A | 0007413 | axonal fasciculation | EN-124k-90-group3630.gs\_19317 | | | | | | | | | | | | | | |  |  |  |
| A | 0007413 | axonal fasciculation | EN-124k-90-group3713.gs\_7861 | | | | | | | | | | | | | | |  |  |  |
| E | 0007413 | axonal fasciculation | EN-124k-90-group4049.EN\_iowa\_15523 | | | | | | | | | | | | | | | |  |  |
| A | 0007413 | axonal fasciculation | EN-124k-90-group4349.gs\_17809 | | | | | | | | | | | | | | |  |  |  |
| E | 0007413 | axonal fasciculation | EN-124k-90-group4600.jgi\_contig\_JGI\_CBBP12800\_fwd | | | | | | | | | | | | | | | | | |
| A | 0007413 | axonal fasciculation | EN-124k-90-group5203.gs\_53868 | | | | | | | | | | | | | | |  |  |  |
| A | 0007413 | axonal fasciculation | EN-124k-90-group5203.gs\_41715 | | | | | | | | | | | | | | |  |  |  |
| A | 0007413 | axonal fasciculation | EN-124k-90-group5203.gs\_19246 | | | | | | | | | | | | | | |  |  |  |
| A | 0007413 | axonal fasciculation | EN-124k-90-group5215.gs\_74365 | | | | | | | | | | | | | | |  |  |  |
| A | 0007413 | axonal fasciculation | EN-124k-90-group5491.gs\_7631 | | | | | | | | | | | | | | |  |  |  |
| A | 0007413 | axonal fasciculation | EN-124k-90-group5491.gs\_75129 | | | | | | | | | | | | | | |  |  |  |
| A | 0007413 | axonal fasciculation | EN-124k-90-group5532.gs\_21595 | | | | | | | | | | | | | | |  |  |  |
| A | 0007413 | axonal fasciculation | EN-124k-90-group5532.gs\_71380 | | | | | | | | | | | | | | |  |  |  |
| A | 0007413 | axonal fasciculation | EN-124k-90-group5532.gs\_38926 | | | | | | | | | | | | | | |  |  |  |
| A | 0007413 | axonal fasciculation | EN-124k-90-group5532.gs\_31235 | | | | | | | | | | | | | | |  |  |  |
| E | 0007413 | axonal fasciculation | EN-124k-90-group5532.EN\_iowa\_9665 | | | | | | | | | | | | | | |  |  |  |
| A | 0007413 | axonal fasciculation | EN-124k-90-group5532.gs\_59967 | | | | | | | | | | | | | | |  |  |  |
| E | 0007413 | axonal fasciculation | EN-124k-90-group5532.EN\_iowa\_13749 | | | | | | | | | | | | | | | |  |  |
| A | 0007413 | axonal fasciculation | EN-124k-90-group5532.gs\_23346 | | | | | | | | | | | | | | |  |  |  |
| E | 0007413 | axonal fasciculation | EN-124k-90-group5532.EN\_iowa\_12774 | | | | | | | | | | | | | | | |  |  |
| A | 0007413 | axonal fasciculation | EN-124k-90-group5532.gs\_2852 | | | | | | | | | | | | | | |  |  |  |
| A | 0007413 | axonal fasciculation | EN-124k-90-group5532.gs\_25188 | | | | | | | | | | | | | | |  |  |  |
| A | 0007413 | axonal fasciculation | EN-124k-90-group5532.gs\_35715 | | | | | | | | | | | | | | |  |  |  |
| A | 0007413 | axonal fasciculation | EN-124k-90-group5532.gs\_7280 | | | | | | | | | | | | | | |  |  |  |
| E | 0007413 | axonal fasciculation | EN-124k-90-group5607.jgi\_contig\_JGI\_CBBP445\_fwd | | | | | | | | | | | | | | | | | |
| E | 0007413 | axonal fasciculation | EN-124k-90-group6472.jgi\_contig\_JGI\_CBBP2402\_fwd | | | | | | | | | | | | | | | | | |
| E | 0007413 | axonal fasciculation | EN-124k-90-group6923.jgi\_contig\_JGI\_CBBP17789\_fwd | | | | | | | | | | | | | | | | | |
| A | 0007413 | axonal fasciculation | EN-124k-90-group7440.gs\_75782 | | | | | | | | | | | | | | |  |  |  |
| A | 0007413 | axonal fasciculation | EN-124k-90-group10963.gs\_69412 | | | | | | | | | | | | | | |  |  |  |
| E | 0007413 | axonal fasciculation | EN-124k-90-group12653.EN\_iowa\_13251 | | | | | | | | | | | | | | | |  |  |
| A | 0007409 | axonogenesis | EN-124k-90-group10674.gs\_45324 | | | | | | | | | | | | | | |  |  |  |
| E | 0007409 | axonogenesis | EN-124k-90-group343.jgi\_contig\_JGI\_CBBP19299\_fwd | | | | | | | | | | | | | | | | | |
| A | 0007409 | axonogenesis | EN-124k-90-group807.gs\_71281 | | | | | | | | | | | | | | |  |  |  |
| E | 0007409 | axonogenesis | EN-124k-90-group1177.jgi\_unpaired\_JGI\_CBBP6661\_fwd | | | | | | | | | | | | | | | | | |
| E | 0007409 | axonogenesis | EN-124k-90-group1306.EN\_iowa\_2354 | | | | | | | | | | | | | | |  |  |  |
| E | 0007409 | axonogenesis | EN-124k-90-group1680.jgi\_contig\_JGI\_CBBP17232\_fwd | | | | | | | | | | | | | | | | | |
| E | 0007409 | axonogenesis | EN-124k-90-group1825.jgi\_contig\_JGI\_CBBP14395\_fwd | | | | | | | | | | | | | | | | | |
| E | 0007409 | axonogenesis | EN-124k-90-group1852.jgi\_paired\_JGI\_CBBP10940\_fwd | | | | | | | | | | | | | | | | | |
| A | 0007409 | axonogenesis | EN-124k-90-group1944.gs\_55531 | | | | | | | | | | | | | | |  |  |  |
| E | 0007409 | axonogenesis | EN-124k-90-group2106.jgi\_paired\_JGI\_CBBP3443\_fwd | | | | | | | | | | | | | | | | | |
| E | 0007409 | axonogenesis | EN-124k-90-group2530.jgi\_paired\_JGI\_CBBP5537\_fwd | | | | | | | | | | | | | | | | | |
| E | 0007409 | axonogenesis | EN-124k-90-group2916.jgi\_paired\_JGI\_CBBP2065\_fwd | | | | | | | | | | | | | | | | | |
| A | 0007409 | axonogenesis | EN-124k-90-group2932.gs\_32728 | | | | | | | | | | | | | | |  |  |  |
| E | 0007409 | axonogenesis | EN-124k-90-group3265.jgi\_paired\_JGI\_CBBP17018\_rev | | | | | | | | | | | | | | | | | |
| A | 0007409 | axonogenesis | EN-124k-90-group3420.gs\_43284 | | | | | | | | | | | | | | |  |  |  |
| E | 0007409 | axonogenesis | EN-124k-90-group3448.jgi\_paired\_JGI\_CBBP19729\_fwd | | | | | | | | | | | | | | | | | |
| A | 0007409 | axonogenesis | EN-124k-90-group3564.gs\_15152 | | | | | | | | | | | | | | |  |  |  |
| E | 0007409 | axonogenesis | EN-124k-90-group3588.jgi\_paired\_JGI\_CBBP13551\_rev | | | | | | | | | | | | | | | | | |
| A | 0007409 | axonogenesis | EN-124k-90-group3807.gs\_52991 | | | | | | | | | | | | | | |  |  |  |
| E | 0007409 | axonogenesis | EN-124k-90-group4197.jgi\_paired\_JGI\_CBBP4981\_fwd | | | | | | | | | | | | | | | | | |
| E | 0007409 | axonogenesis | EN-124k-90-group4248.jgi\_contig\_JGI\_CBBP19155\_fwd | | | | | | | | | | | | | | | | | |
| E | 0007409 | axonogenesis | EN-124k-90-group5117.jgi\_paired\_JGI\_CBBP19153\_fwd | | | | | | | | | | | | | | | | | |
| A | 0007409 | axonogenesis | EN-124k-90-group5330.gs\_86287 | | | | | | | | | | | | | | |  |  |  |
| A | 0007409 | axonogenesis | EN-124k-90-group5346.gs\_72008 | | | | | | | | | | | | | | |  |  |  |
| A | 0007409 | axonogenesis | EN-124k-90-group5384.gs\_17980 | | | | | | | | | | | | | | |  |  |  |
| A | 0007409 | axonogenesis | EN-124k-90-group5423.gs\_20381 | | | | | | | | | | | | | | |  |  |  |
| A | 0007409 | axonogenesis | EN-124k-90-group5604.gs\_26346 | | | | | | | | | | | | | | |  |  |  |
| E | 0007409 | axonogenesis | EN-124k-90-group5741.jgi\_paired\_JGI\_CBBP19133\_fwd | | | | | | | | | | | | | | | | | |
| E | 0007409 | axonogenesis | EN-124k-90-group5833.EN\_iowa\_5392 | | | | | | | | | | | | | | |  |  |  |
| E | 0007409 | axonogenesis | EN-124k-90-group5834.jgi\_contig\_JGI\_CBBP12701\_fwd | | | | | | | | | | | | | | | | | |
| E | 0007409 | axonogenesis | EN-124k-90-group6144.EN\_iowa\_5856 | | | | | | | | | | | | | | |  |  |  |
| E | 0007409 | axonogenesis | EN-124k-90-group6152.jgi\_paired\_JGI\_CBBP3620\_fwd | | | | | | | | | | | | | | | | | |
| E | 0007409 | axonogenesis | EN-124k-90-group6737.jgi\_contig\_JGI\_CBBP17820\_fwd | | | | | | | | | | | | | | | | | |
| E | 0007409 | axonogenesis | EN-124k-90-group7523.jgi\_paired\_JGI\_CBBP3627\_rev | | | | | | | | | | | | | | | | | |
| E | 0007409 | axonogenesis | EN-124k-90-group7594.jgi\_paired\_JGI\_CBBP17768\_fwd | | | | | | | | | | | | | | | | | |
| A | 0007409 | axonogenesis | EN-124k-90-group7595.gs\_36261 | | | | | | | | | | | | | | |  |  |  |
| E | 0007409 | axonogenesis | EN-124k-90-group7598.jgi\_unpaired\_JGI\_CBBP18343\_fwd | | | | | | | | | | | | | | | | | |
| E | 0007409 | axonogenesis | EN-124k-90-group7869.jgi\_paired\_JGI\_CBBP11345\_fwd | | | | | | | | | | | | | | | | | |
| E | 0007409 | axonogenesis | EN-124k-90-group7940.jgi\_contig\_JGI\_CBBP6088\_fwd | | | | | | | | | | | | | | | | | |
| A | 0007409 | axonogenesis | EN-124k-90-group7953.gs\_11284 | | | | | | | | | | | | | | |  |  |  |
| E | 0007409 | axonogenesis | EN-124k-90-group8037.jgi\_paired\_JGI\_CBBP11201\_fwd | | | | | | | | | | | | | | | | | |
| E | 0007409 | axonogenesis | EN-124k-90-group8104.jgi\_paired\_JGI\_CBBP9893\_fwd | | | | | | | | | | | | | | | | | |
| E | 0007409 | axonogenesis | EN-124k-90-group8107.jgi\_paired\_JGI\_CBBP6197\_fwd | | | | | | | | | | | | | | | | | |
| A | 0007409 | axonogenesis | EN-124k-90-group8144.gs\_73566 | | | | | | | | | | | | | | |  |  |  |
| A | 0007409 | axonogenesis | EN-124k-90-group8310.gs\_24913 | | | | | | | | | | | | | | |  |  |  |
| E | 0007409 | axonogenesis | EN-124k-90-group8370.jgi\_paired\_JGI\_CBBP17511\_fwd | | | | | | | | | | | | | | | | | |
| E | 0007409 | axonogenesis | EN-124k-90-group8443.jgi\_paired\_JGI\_CBBP6445\_fwd | | | | | | | | | | | | | | | | | |
| A | 0007409 | axonogenesis | EN-124k-90-group8567.gs\_80390 | | | | | | | | | | | | | | |  |  |  |
| A | 0007409 | axonogenesis | EN-124k-90-group8658.gs\_22315 | | | | | | | | | | | | | | |  |  |  |
| E | 0007409 | axonogenesis | EN-124k-90-group8721.jgi\_paired\_JGI\_CBBP11443\_rev | | | | | | | | | | | | | | | | | |
| E | 0007409 | axonogenesis | EN-124k-90-group9037.jgi\_paired\_JGI\_CBBP9664\_rev | | | | | | | | | | | | | | | | | |
| E | 0007409 | axonogenesis | EN-124k-90-group9064.EN\_iowa\_5147 | | | | | | | | | | | | | | |  |  |  |
| E | 0007409 | axonogenesis | EN-124k-90-group9263.jgi\_paired\_JGI\_CBBP3642\_fwd | | | | | | | | | | | | | | | | | |
| E | 0007409 | axonogenesis | EN-124k-90-group9286.jgi\_paired\_JGI\_CBBP4063\_fwd | | | | | | | | | | | | | | | | | |
| A | 0007409 | axonogenesis | EN-124k-90-group9467.gs\_16039 | | | | | | | | | | | | | | |  |  |  |
| A | 0007409 | axonogenesis | EN-124k-90-group9534.gs\_76749 | | | | | | | | | | | | | | |  |  |  |
| A | 0007409 | axonogenesis | EN-124k-90-group9629.gs\_48808 | | | | | | | | | | | | | | |  |  |  |
| A | 0007409 | axonogenesis | EN-124k-90-group9714.gs\_64750 | | | | | | | | | | | | | | |  |  |  |
| A | 0007409 | axonogenesis | EN-124k-90-group9739.gs\_17794 | | | | | | | | | | | | | | |  |  |  |
| E | 0007409 | axonogenesis | EN-124k-90-group9773.jgi\_paired\_JGI\_CBBP6585\_rev | | | | | | | | | | | | | | | | | |
| A | 0007409 | axonogenesis | EN-124k-90-group9881.gs\_79019 | | | | | | | | | | | | | | |  |  |  |
| A | 0007409 | axonogenesis | EN-124k-90-group9910.gs\_20549 | | | | | | | | | | | | | | |  |  |  |
| A | 0007409 | axonogenesis | EN-124k-90-group9923.gs\_69535 | | | | | | | | | | | | | | |  |  |  |
| E | 0007409 | axonogenesis | EN-124k-90-group9989.EN\_iowa\_3399 | | | | | | | | | | | | | | |  |  |  |
| E | 0007409 | axonogenesis | EN-124k-90-group10110.jgi\_paired\_JGI\_CBBP10219\_fwd | | | | | | | | | | | | | | | | | |
| E | 0007409 | axonogenesis | EN-124k-90-group10277.jgi\_paired\_JGI\_CBBP15154\_rev | | | | | | | | | | | | | | | | | |
| E | 0007409 | axonogenesis | EN-124k-90-group10304.jgi\_paired\_JGI\_CBBP11045\_fwd | | | | | | | | | | | | | | | | | |
| A | 0007409 | axonogenesis | EN-124k-90-group10312.gs\_22624 | | | | | | | | | | | | | | |  |  |  |
| E | 0007409 | axonogenesis | EN-124k-90-group10416.jgi\_contig\_JGI\_CBBP3274\_fwd | | | | | | | | | | | | | | | | | |
| E | 0007409 | axonogenesis | EN-124k-90-group10458.jgi\_contig\_JGI\_CBBP9840\_fwd | | | | | | | | | | | | | | | | | |
| E | 0007409 | axonogenesis | EN-124k-90-group10538.jgi\_contig\_JGI\_CBBP12785\_fwd | | | | | | | | | | | | | | | | | |
| A | 0007409 | axonogenesis | EN-124k-90-group10635.gs\_47150 | | | | | | | | | | | | | | |  |  |  |
| A | 0007409 | axonogenesis | EN-124k-90-group10841.gs\_43135 | | | | | | | | | | | | | | |  |  |  |
| E | 0007409 | axonogenesis | EN-124k-90-group10878.jgi\_paired\_JGI\_CBBP952\_rev | | | | | | | | | | | | | | | | | |
| E | 0007409 | axonogenesis | EN-124k-90-group10888.jgi\_paired\_JGI\_CBBP10968\_rev | | | | | | | | | | | | | | | | | |
| A | 0007409 | axonogenesis | EN-124k-90-group10935.gs\_64995 | | | | | | | | | | | | | | |  |  |  |
| E | 0007409 | axonogenesis | EN-124k-90-group10968.EN\_iowa\_5471 | | | | | | | | | | | | | | | |  |  |
| A | 0007409 | axonogenesis | EN-124k-90-group11067.gs\_59652 | | | | | | | | | | | | | | |  |  |  |
| A | 0007409 | axonogenesis | EN-124k-90-group11147.gs\_84591 | | | | | | | | | | | | | | |  |  |  |
| E | 0007409 | axonogenesis | EN-124k-90-group11156.EN\_iowa\_9243 | | | | | | | | | | | | | | | |  |  |
| E | 0007409 | axonogenesis | EN-124k-90-group11184.EN\_iowa\_18337 | | | | | | | | | | | | | | | |  |  |
| E | 0007409 | axonogenesis | EN-124k-90-group11237.jgi\_paired\_JGI\_CBBP10968\_fwd | | | | | | | | | | | | | | | | | |
| A | 0007409 | axonogenesis | EN-124k-90-group11354.gs\_78099 | | | | | | | | | | | | | | |  |  |  |
| A | 0007409 | axonogenesis | EN-124k-90-group11434.gs\_43503 | | | | | | | | | | | | | | |  |  |  |
| E | 0007409 | axonogenesis | EN-124k-90-group11438.jgi\_paired\_JGI\_CBBP9709\_fwd | | | | | | | | | | | | | | | | | |
| A | 0007409 | axonogenesis | EN-124k-90-group11441.gs\_30577 | | | | | | | | | | | | | | |  |  |  |
| E | 0007409 | axonogenesis | EN-124k-90-group11503.jgi\_paired\_JGI\_CBBP14517\_fwd | | | | | | | | | | | | | | | | | |
| E | 0007409 | axonogenesis | EN-124k-90-group11545.jgi\_paired\_JGI\_CBBP2808\_fwd | | | | | | | | | | | | | | | | | |
| E | 0007409 | axonogenesis | EN-124k-90-group11559.jgi\_unpaired\_JGI\_CBBP12231\_fwd | | | | | | | | | | | | | | | | | |
| E | 0007409 | axonogenesis | EN-124k-90-group11586.jgi\_paired\_JGI\_CBBP13551\_fwd | | | | | | | | | | | | | | | | | |
| E | 0007409 | axonogenesis | EN-124k-90-group11708.jgi\_contig\_JGI\_CBBP516\_fwd | | | | | | | | | | | | | | | | | |
| A | 0007409 | axonogenesis | EN-124k-90-group11738.gs\_53113 | | | | | | | | | | | | | | |  |  |  |
| E | 0007409 | axonogenesis | EN-124k-90-group11780.jgi\_paired\_JGI\_CBBP12332\_fwd | | | | | | | | | | | | | | | | | |
| A | 0007409 | axonogenesis | EN-124k-90-group11821.gs\_86440 | | | | | | | | | | | | | | |  |  |  |
| A | 0007409 | axonogenesis | EN-124k-90-group11865.gs\_82011 | | | | | | | | | | | | | | |  |  |  |
| E | 0007409 | axonogenesis | EN-124k-90-group11878.EN\_iowa\_18095 | | | | | | | | | | | | | | | |  |  |
| E | 0007409 | axonogenesis | EN-124k-90-group11882.EN\_iowa\_9262 | | | | | | | | | | | | | | | |  |  |
| E | 0007409 | axonogenesis | EN-124k-90-group11917.jgi\_paired\_JGI\_CBBP3416\_fwd | | | | | | | | | | | | | | | | | |
| E | 0007409 | axonogenesis | EN-124k-90-group12007.jgi\_paired\_JGI\_CBBP14791\_fwd | | | | | | | | | | | | | | | | | |
| E | 0007409 | axonogenesis | EN-124k-90-group12012.jgi\_contig\_JGI\_CBBP10480\_fwd | | | | | | | | | | | | | | | | | |
| A | 0007409 | axonogenesis | EN-124k-90-group12032.gs\_85627 | | | | | | | | | | | | | | |  |  |  |
| A | 0007409 | axonogenesis | EN-124k-90-group12066.gs\_43214 | | | | | | | | | | | | | | |  |  |  |
| A | 0007409 | axonogenesis | EN-124k-90-group12082.gs\_71967 | | | | | | | | | | | | | | |  |  |  |
| E | 0007409 | axonogenesis | EN-124k-90-group12191.jgi\_paired\_JGI\_CBBP10887\_fwd | | | | | | | | | | | | | | | | | |
| A | 0007409 | axonogenesis | EN-124k-90-group12204.gs\_9947 | | | | | | | | | | | | | | |  |  |  |
| E | 0007409 | axonogenesis | EN-124k-90-group12452.EN\_iowa\_9722 | | | | | | | | | | | | | | | |  |  |
| E | 0007409 | axonogenesis | EN-124k-90-group12477.jgi\_paired\_JGI\_CBBP10794\_fwd | | | | | | | | | | | | | | | | | |
| E | 0007409 | axonogenesis | EN-124k-90-group12537.jgi\_contig\_JGI\_CBBP17605\_fwd | | | | | | | | | | | | | | | | | |
| E | 0007409 | axonogenesis | EN-124k-90-group12563.EN\_iowa\_9742 | | | | | | | | | | | | | | | |  |  |
| E | 0007409 | axonogenesis | EN-124k-90-group12595.jgi\_contig\_JGI\_CBBP15955\_fwd | | | | | | | | | | | | | | | | | |
| E | 0007409 | axonogenesis | EN-124k-90-group12676.jgi\_paired\_JGI\_CBBP17511\_rev | | | | | | | | | | | | | | | | | |
| E | 0007409 | axonogenesis | EN-124k-90-group12683.jgi\_paired\_JGI\_CBBP11245\_fwd | | | | | | | | | | | | | | | | | |
| E | 0007409 | axonogenesis | EN-124k-90-group12721.EN\_iowa\_5331 | | | | | | | | | | | | | | | |  |  |
| A | 0007409 | axonogenesis | EN-124k-90-group12740.gs\_56121 | | | | | | | | | | | | | | |  |  |  |
| E | 0007409 | axonogenesis | EN-124k-90-group12744.EN\_iowa\_4256 | | | | | | | | | | | | | | | |  |  |
| A | 0007409 | axonogenesis | EN-124k-90-group12821.gs\_58803 | | | | | | | | | | | | | | |  |  |  |
| A | 0007409 | axonogenesis | EN-124k-90-group12946.gs\_86900 | | | | | | | | | | | | | | |  |  |  |
| A | 0007409 | axonogenesis | EN-124k-90-group13006.gs\_33814 | | | | | | | | | | | | | | |  |  |  |
| E | 0007409 | axonogenesis | EN-124k-90-group13011.jgi\_paired\_JGI\_CBBP19322\_fwd | | | | | | | | | | | | | | | | | |
| E | 0007409 | axonogenesis | EN-124k-90-group13023.EN\_iowa\_251 | | | | | | | | | | | | | | |  |  |  |
| E | 0007409 | axonogenesis | EN-124k-90-group13146.jgi\_paired\_JGI\_CBBP17160\_fwd | | | | | | | | | | | | | | | | | |
| E | 0007409 | axonogenesis | EN-124k-90-group13346.jgi\_unpaired\_JGI\_CBBP5460\_rev | | | | | | | | | | | | | | | | | |
| E | 0007409 | axonogenesis | EN-124k-90-group13368.jgi\_unpaired\_JGI\_CBBP5492\_rev | | | | | | | | | | | | | | | | | |
| E | 0007409 | axonogenesis | EN-124k-90-group13382.jgi\_contig\_JGI\_CBBP6648\_fwd | | | | | | | | | | | | | | | | | |
| A | 0007409 | axonogenesis | EN-124k-90-group13390.gs\_82388 | | | | | | | | | | | | | | |  |  |  |
| E | 0007409 | axonogenesis | EN-124k-90-group13505.EN\_iowa\_4386 | | | | | | | | | | | | | | | |  |  |
| A | 0007409 | axonogenesis | EN-124k-90-group13506.gs\_86402 | | | | | | | | | | | | | | |  |  |  |
| E | 0007409 | axonogenesis | EN-124k-90-group13508.EN\_iowa\_12581 | | | | | | | | | | | | | | | |  |  |
| E | 0007409 | axonogenesis | EN-124k-90-group13515.EN\_iowa\_10440 | | | | | | | | | | | | | | | |  |  |
| E | 0007409 | axonogenesis | EN-124k-90-group13547.jgi\_contig\_JGI\_CBBP6263\_fwd | | | | | | | | | | | | | | | | | |
| A | 0007409 | axonogenesis | EN-124k-90-group13721.gs\_13610 | | | | | | | | | | | | | | |  |  |  |
| A | 0007409 | axonogenesis | EN-124k-90-group13771.gs\_28668 | | | | | | | | | | | | | | |  |  |  |
| A | 0007409 | axonogenesis | EN-124k-90-group13823.gs\_32410 | | | | | | | | | | | | | | |  |  |  |
| E | 0007409 | axonogenesis | EN-124k-90-group13906.jgi\_paired\_JGI\_CBBP12965\_fwd | | | | | | | | | | | | | | | | | |
| E | 0007409 | axonogenesis | EN-124k-90-group13916.EN\_iowa\_14815 | | | | | | | | | | | | | | | |  |  |
| A | 0007409 | axonogenesis | EN-124k-90-group13938.gs\_32571 | | | | | | | | | | | | | | |  |  |  |
| E | 0007409 | axonogenesis | EN-124k-90-group13951.jgi\_paired\_JGI\_CBBP12336\_fwd | | | | | | | | | | | | | | | | | |
| A | 0007409 | axonogenesis | EN-124k-90-group14040.gs\_63341 | | | | | | | | | | | | | | |  |  |  |
| E | 0007409 | axonogenesis | EN-124k-90-group14050.jgi\_paired\_JGI\_CBBP18955\_fwd | | | | | | | | | | | | | | | | | |
| E | 0007409 | axonogenesis | EN-124k-90-group14114.jgi\_paired\_JGI\_CBBP9776\_fwd | | | | | | | | | | | | | | | | | |
| A | 0007409 | axonogenesis | EN-124k-90-group14128.gs\_38861 | | | | | | | | | | | | | | |  |  |  |
| E | 0007409 | axonogenesis | EN-124k-90-group14311.jgi\_paired\_JGI\_CBBP682\_fwd | | | | | | | | | | | | | | | | | |
| A | 0007409 | axonogenesis | EN-124k-90-group14320.gs\_86221 | | | | | | | | | | | | | | |  |  |  |
| E | 0007409 | axonogenesis | EN-124k-90-group14335.jgi\_paired\_JGI\_CBBP12938\_fwd | | | | | | | | | | | | | | | | | |
| A | 0007409 | axonogenesis | EN-124k-90-group14351.gs\_85401 | | | | | | | | | | | | | | |  |  |  |
| E | 0007409 | axonogenesis | EN-124k-90-group14452.jgi\_paired\_JGI\_CBBP10500\_fwd | | | | | | | | | | | | | | | | | |
| E | 0007409 | axonogenesis | EN-124k-90-group14475.jgi\_paired\_JGI\_CBBP15870\_rev | | | | | | | | | | | | | | | | | |
| E | 0007409 | axonogenesis | EN-124k-90-group14509.jgi\_paired\_JGI\_CBBP933\_fwd | | | | | | | | | | | | | | | | | |
| E | 0007409 | axonogenesis | EN-124k-90-group14544.jgi\_contig\_JGI\_CBBP14322\_fwd | | | | | | | | | | | | | | | | | |
| A | 0007409 | axonogenesis | EN-124k-90-group14563.gs\_48486 | | | | | | | | | | | | | | |  |  |  |
| E | 0007409 | axonogenesis | EN-124k-90-group14573.jgi\_paired\_JGI\_CBBP20040\_fwd | | | | | | | | | | | | | | | | | |
| A | 0007409 | axonogenesis | EN-124k-90-group14582.gs\_17139 | | | | | | | | | | | | | | |  |  |  |
| A | 0007409 | axonogenesis | EN-124k-90-group14609.gs\_33389 | | | | | | | | | | | | | | |  |  |  |
| A | 0007409 | axonogenesis | EN-124k-90-group14734.gs\_78905 | | | | | | | | | | | | | | |  |  |  |
| A | 0007409 | axonogenesis | EN-124k-90-group14813.gs\_84560 | | | | | | | | | | | | | | |  |  |  |
| E | 0007409 | axonogenesis | EN-124k-90-group14826.jgi\_paired\_JGI\_CBBP20200\_fwd | | | | | | | | | | | | | | | | | |
| A | 0007409 | axonogenesis | EN-124k-90-group14887.gs\_61232 | | | | | | | | | | | | | | |  |  |  |
| E | 0007409 | axonogenesis | EN-124k-90-group14931.jgi\_paired\_JGI\_CBBP10444\_fwd | | | | | | | | | | | | | | | | | |
| E | 0007409 | axonogenesis | EN-124k-90-group14935.jgi\_contig\_JGI\_CBBP17959\_fwd | | | | | | | | | | | | | | | | | |
| A | 0007409 | axonogenesis | EN-124k-90-group14948.gs\_26769 | | | | | | | | | | | | | | |  |  |  |
| A | 0007409 | axonogenesis | EN-124k-90-group15056.gs\_73097 | | | | | | | | | | | | | | |  |  |  |
| A | 0007409 | axonogenesis | EN-124k-90-group15062.gs\_40000 | | | | | | | | | | | | | | |  |  |  |
| A | 0007409 | axonogenesis | EN-124k-90-group15124.gs\_82644 | | | | | | | | | | | | | | |  |  |  |
| E | 0007409 | axonogenesis | EN-124k-90-group15223.EN\_iowa\_18438 | | | | | | | | | | | | | | | |  |  |
| A | 0007409 | axonogenesis | EN-124k-90-group15229.gs\_75551 | | | | | | | | | | | | | | |  |  |  |
| A | 0007409 | axonogenesis | EN-124k-90-group15268.gs\_21540 | | | | | | | | | | | | | | |  |  |  |
| E | 0007409 | axonogenesis | EN-124k-90-group15289.jgi\_paired\_JGI\_CBBP6226\_fwd | | | | | | | | | | | | | | | | | |
| E | 0007409 | axonogenesis | EN-124k-90-group15290.EN\_iowa\_9903 | | | | | | | | | | | | | | | |  |  |
| E | 0007409 | axonogenesis | EN-124k-90-group15320.jgi\_paired\_JGI\_CBBP9817\_fwd | | | | | | | | | | | | | | | | | |
| E | 0007409 | axonogenesis | EN-124k-90-group15378.jgi\_contig\_JGI\_CBBP10913\_fwd | | | | | | | | | | | | | | | | | |
| A | 0007409 | axonogenesis | EN-124k-90-group15397.gs\_22489 | | | | | | | | | | | | | | |  |  |  |
| A | 0007409 | axonogenesis | EN-124k-90-group15399.gs\_80457 | | | | | | | | | | | | | | |  |  |  |
| A | 0007409 | axonogenesis | EN-124k-90-group15413.gs\_80915 | | | | | | | | | | | | | | |  |  |  |
| E | 0007409 | axonogenesis | EN-124k-90-group15532.EN\_iowa\_5883 | | | | | | | | | | | | | | | |  |  |
| E | 0007409 | axonogenesis | EN-124k-90-group15546.EN\_iowa\_13502 | | | | | | | | | | | | | | | |  |  |
| E | 0007409 | axonogenesis | EN-124k-90-group15553.EN\_iowa\_14509 | | | | | | | | | | | | | | | |  |  |
| A | 0007409 | axonogenesis | EN-124k-90-group15556.gs\_43579 | | | | | | | | | | | | | | |  |  |  |
| E | 0007409 | axonogenesis | EN-124k-90-group15588.jgi\_paired\_JGI\_CBBP15154\_fwd | | | | | | | | | | | | | | | | | |
| E | 0007409 | axonogenesis | EN-124k-90-group15601.jgi\_paired\_JGI\_CBBP13413\_fwd | | | | | | | | | | | | | | | | | |
| E | 0007409 | axonogenesis | EN-124k-90-group15671.jgi\_paired\_JGI\_CBBP10266\_fwd | | | | | | | | | | | | | | | | | |
| E | 0007409 | axonogenesis | EN-124k-90-group15692.jgi\_contig\_JGI\_CBBP12510\_fwd | | | | | | | | | | | | | | | | | |
| E | 0007409 | axonogenesis | EN-124k-90-group15723.jgi\_paired\_JGI\_CBBP16981\_fwd | | | | | | | | | | | | | | | | | |
| A | 0007409 | axonogenesis | EN-124k-90-group15749.gs\_84109 | | | | | | | | | | | | | | |  |  |  |
| A | 0007409 | axonogenesis | EN-124k-90-group15770.gs\_45569 | | | | | | | | | | | | | | |  |  |  |
| E | 0007409 | axonogenesis | EN-124k-90-group15776.EN\_iowa\_1971 | | | | | | | | | | | | | | | |  |  |
| E | 0007409 | axonogenesis | EN-124k-90-group15789.jgi\_paired\_JGI\_CBBP2907\_fwd | | | | | | | | | | | | | | | | | |
| A | 0007409 | axonogenesis | EN-124k-90-group15856.gs\_15241 | | | | | | | | | | | | | | |  |  |  |
| E | 0007409 | axonogenesis | EN-124k-90-group15877.EN\_iowa\_3358 | | | | | | | | | | | | | | | |  |  |
| E | 0007409 | axonogenesis | EN-124k-90-group15927.EN\_iowa\_7602 | | | | | | | | | | | | | | | |  |  |
| E | 0007409 | axonogenesis | EN-124k-90-group15944.jgi\_paired\_JGI\_CBBP5778\_fwd | | | | | | | | | | | | | | | | | |
| E | 0007409 | axonogenesis | EN-124k-90-group15994.jgi\_paired\_JGI\_CBBP14428\_fwd | | | | | | | | | | | | | | | | | |
| A | 0007409 | axonogenesis | EN-124k-90-group16026.gs\_15943 | | | | | | | | | | | | | | |  |  |  |
| A | 0007409 | axonogenesis | EN-124k-90-group16067.gs\_19008 | | | | | | | | | | | | | | |  |  |  |
| E | 0007409 | axonogenesis | EN-124k-90-group16134.jgi\_contig\_JGI\_CBBP14519\_fwd | | | | | | | | | | | | | | | | | |
| E | 0007409 | axonogenesis | EN-124k-90-group16143.jgi\_paired\_JGI\_CBBP4408\_fwd | | | | | | | | | | | | | | | | | |
| A | 0007409 | axonogenesis | EN-124k-90-group16149.gs\_13439 | | | | | | | | | | | | | | |  |  |  |
| A | 0007409 | axonogenesis | EN-124k-90-group16189.gs\_70159 | | | | | | | | | | | | | | |  |  |  |
| E | 0007409 | axonogenesis | EN-124k-90-group16200.jgi\_paired\_JGI\_CBBP10444\_rev | | | | | | | | | | | | | | | | | |
| A | 0007409 | axonogenesis | EN-124k-90-group16285.gs\_47129 | | | | | | | | | | | | | | |  |  |  |
| A | 0007409 | axonogenesis | EN-124k-90-group16291.gs\_65588 | | | | | | | | | | | | | | |  |  |  |
| A | 0007409 | axonogenesis | EN-124k-90-group16323.gs\_48303 | | | | | | | | | | | | | | |  |  |  |
| E | 0007409 | axonogenesis | EN-124k-90-group16346.jgi\_contig\_JGI\_CBBP6214\_fwd | | | | | | | | | | | | | | | | | |
| A | 0007409 | axonogenesis | EN-124k-90-group16399.gs\_80314 | | | | | | | | | | | | | | |  |  |  |
| A | 0007409 | axonogenesis | EN-124k-90-group16412.gs\_64548 | | | | | | | | | | | | | | |  |  |  |
| A | 0007409 | axonogenesis | EN-124k-90-group16416.gs\_28586 | | | | | | | | | | | | | | |  |  |  |
| A | 0007409 | axonogenesis | EN-124k-90-group16439.gs\_54729 | | | | | | | | | | | | | | |  |  |  |
| A | 0007409 | axonogenesis | EN-124k-90-group16443.gs\_57863 | | | | | | | | | | | | | | |  |  |  |
| A | 0007409 | axonogenesis | EN-124k-90-group16489.gs\_80464 | | | | | | | | | | | | | | |  |  |  |
| A | 0007409 | axonogenesis | EN-124k-90-group16520.gs\_62867 | | | | | | | | | | | | | | |  |  |  |
| A | 0007409 | axonogenesis | EN-124k-90-group16522.gs\_78634 | | | | | | | | | | | | | | |  |  |  |
| A | 0007409 | axonogenesis | EN-124k-90-group16523.gs\_75670 | | | | | | | | | | | | | | |  |  |  |
| A | 0007409 | axonogenesis | EN-124k-90-group16598.gs\_3233 | | | | | | | | | | | | | | |  |  |  |
| A | 0007409 | axonogenesis | EN-124k-90-group16636.gs\_429 | | | | | | | | | | | | | | |  |  |  |
| A | 0007409 | axonogenesis | EN-124k-90-group16714.gs\_320 | | | | | | | | | | | | | | |  |  |  |
| A | 0007409 | axonogenesis | EN-124k-90-group16826.gs\_5886 | | | | | | | | | | | | | | |  |  |  |
| A | 0007409 | axonogenesis | EN-124k-90-group16830.gs\_82512 | | | | | | | | | | | | | | |  |  |  |
| A | 0007409 | axonogenesis | EN-124k-90-group16845.gs\_50498 | | | | | | | | | | | | | | |  |  |  |
| A | 0007409 | axonogenesis | EN-124k-90-group16851.gs\_64557 | | | | | | | | | | | | | | |  |  |  |
| A | 0007409 | axonogenesis | EN-124k-90-group16852.gs\_27372 | | | | | | | | | | | | | | |  |  |  |
| A | 0007409 | axonogenesis | EN-124k-90-group16857.gs\_13788 | | | | | | | | | | | | | | |  |  |  |
| A | 0007409 | axonogenesis | EN-124k-90-group16872.gs\_213 | | | | | | | | | | | | | | |  |  |  |
| A | 0007409 | axonogenesis | EN-124k-90-group16910.gs\_22871 | | | | | | | | | | | | | | |  |  |  |
| A | 0007409 | axonogenesis | EN-124k-90-group16929.gs\_43290 | | | | | | | | | | | | | | |  |  |  |
| A | 0007409 | axonogenesis | EN-124k-90-group16932.gs\_197 | | | | | | | | | | | | | | |  |  |  |
| A | 0007409 | axonogenesis | EN-124k-90-group17012.gs\_52263 | | | | | | | | | | | | | | |  |  |  |
| A | 0007409 | axonogenesis | EN-124k-90-group17022.gs\_80073 | | | | | | | | | | | | | | |  |  |  |
| E | 0007409 | axonogenesis | EN-124k-90-group17086.jgi\_paired\_JGI\_CBBP6417\_rev | | | | | | | | | | | | | | | | | |
| E | 0007409 | axonogenesis | EN-124k-90-group17091.jgi\_paired\_JGI\_CBBP10179\_rev | | | | | | | | | | | | | | | | | |
| A | 0007409 | axonogenesis | EN-124k-90-group17117.gs\_84807 | | | | | | | | | | | | | | |  |  |  |
| A | 0007409 | axonogenesis | EN-124k-90-group17129.gs\_61289 | | | | | | | | | | | | | | |  |  |  |
| A | 0007409 | axonogenesis | EN-124k-90-group17140.gs\_14565 | | | | | | | | | | | | | | |  |  |  |
| A | 0007409 | axonogenesis | EN-124k-90-group17161.gs\_56414 | | | | | | | | | | | | | | |  |  |  |
| A | 0007409 | axonogenesis | EN-124k-90-group17210.gs\_64350 | | | | | | | | | | | | | | |  |  |  |
| A | 0007409 | axonogenesis | EN-124k-90-group17273.gs\_68617 | | | | | | | | | | | | | | |  |  |  |
| A | 0007409 | axonogenesis | EN-124k-90-group17276.gs\_80679 | | | | | | | | | | | | | | |  |  |  |
| A | 0007409 | axonogenesis | EN-124k-90-group17301.gs\_75053 | | | | | | | | | | | | | | |  |  |  |
| A | 0007409 | axonogenesis | EN-124k-90-group17321.gs\_60379 | | | | | | | | | | | | | | |  |  |  |
| A | 0007409 | axonogenesis | EN-124k-90-group17360.gs\_72469 | | | | | | | | | | | | | | |  |  |  |
| A | 0007409 | axonogenesis | EN-124k-90-group17386.gs\_68411 | | | | | | | | | | | | | | |  |  |  |
| A | 0007409 | axonogenesis | EN-124k-90-group17411.gs\_13940 | | | | | | | | | | | | | | |  |  |  |
| A | 0007409 | axonogenesis | EN-124k-90-group17433.gs\_222 | | | | | | | | | | | | | | |  |  |  |
| A | 0007409 | axonogenesis | EN-124k-90-group17442.gs\_63748 | | | | | | | | | | | | | | |  |  |  |
| A | 0007409 | axonogenesis | EN-124k-90-group17445.gs\_83255 | | | | | | | | | | | | | | |  |  |  |
| A | 0007409 | axonogenesis | EN-124k-90-group17471.gs\_85794 | | | | | | | | | | | | | | |  |  |  |
| A | 0007409 | axonogenesis | EN-124k-90-group17487.gs\_82041 | | | | | | | | | | | | | | |  |  |  |
| A | 0007409 | axonogenesis | EN-124k-90-group17524.gs\_80802 | | | | | | | | | | | | | | |  |  |  |
| A | 0007409 | axonogenesis | EN-124k-90-group17556.gs\_87114 | | | | | | | | | | | | | | |  |  |  |
| A | 0007409 | axonogenesis | EN-124k-90-group17563.gs\_80410 | | | | | | | | | | | | | | |  |  |  |
| A | 0007409 | axonogenesis | EN-124k-90-group17567.gs\_58610 | | | | | | | | | | | | | | |  |  |  |
| A | 0007409 | axonogenesis | EN-124k-90-group17572.gs\_7187 | | | | | | | | | | | | | | |  |  |  |
| A | 0007409 | axonogenesis | EN-124k-90-group17582.gs\_70636 | | | | | | | | | | | | | | |  |  |  |
| A | 0007409 | axonogenesis | EN-124k-90-group17650.gs\_59359 | | | | | | | | | | | | | | |  |  |  |
| A | 0007409 | axonogenesis | EN-124k-90-group17657.gs\_68076 | | | | | | | | | | | | | | |  |  |  |
| A | 0007409 | axonogenesis | EN-124k-90-group17679.gs\_71618 | | | | | | | | | | | | | | |  |  |  |
| A | 0007409 | axonogenesis | EN-124k-90-group17702.gs\_18827 | | | | | | | | | | | | | | |  |  |  |
| A | 0007409 | axonogenesis | EN-124k-90-group17743.gs\_53689 | | | | | | | | | | | | | | |  |  |  |
| A | 0007409 | axonogenesis | EN-124k-90-group17744.gs\_30255 | | | | | | | | | | | | | | |  |  |  |
| A | 0007409 | axonogenesis | EN-124k-90-group17776.gs\_56285 | | | | | | | | | | | | | | |  |  |  |
| A | 0007409 | axonogenesis | EN-124k-90-group17819.gs\_51352 | | | | | | | | | | | | | | |  |  |  |
| A | 0007409 | axonogenesis | EN-124k-90-group17864.gs\_35131 | | | | | | | | | | | | | | |  |  |  |
| A | 0007409 | axonogenesis | EN-124k-90-group17887.gs\_80072 | | | | | | | | | | | | | | |  |  |  |
| A | 0007409 | axonogenesis | EN-124k-90-group17892.gs\_49260 | | | | | | | | | | | | | | |  |  |  |
| A | 0007409 | axonogenesis | EN-124k-90-group17930.gs\_83513 | | | | | | | | | | | | | | |  |  |  |
| E | 0007409 | axonogenesis | EN-124k-90-group17943.EN\_iowa\_18374 | | | | | | | | | | | | | | | |  |  |
| A | 0007409 | axonogenesis | EN-124k-90-group17951.gs\_43129 | | | | | | | | | | | | | | |  |  |  |
| E | 0007409 | axonogenesis | EN-124k-90-group17979.jgi\_contig\_JGI\_CBBP2421\_fwd | | | | | | | | | | | | | | | | | |
| A | 0007409 | axonogenesis | EN-124k-90-group18006.gs\_18909 | | | | | | | | | | | | | | |  |  |  |
| A | 0007409 | axonogenesis | EN-124k-90-group18033.gs\_11467 | | | | | | | | | | | | | | |  |  |  |
| A | 0007409 | axonogenesis | EN-124k-90-group18038.gs\_30262 | | | | | | | | | | | | | | |  |  |  |
| A | 0007409 | axonogenesis | EN-124k-90-group18140.gs\_87168 | | | | | | | | | | | | | | |  |  |  |
| A | 0007409 | axonogenesis | EN-124k-90-group18148.gs\_80788 | | | | | | | | | | | | | | |  |  |  |
| A | 0007409 | axonogenesis | EN-124k-90-group18205.gs\_57120 | | | | | | | | | | | | | | |  |  |  |
| A | 0007409 | axonogenesis | EN-124k-90-group18238.gs\_39782 | | | | | | | | | | | | | | |  |  |  |
| A | 0007409 | axonogenesis | EN-124k-90-group18245.gs\_78006 | | | | | | | | | | | | | | |  |  |  |
| A | 0007409 | axonogenesis | EN-124k-90-group18289.gs\_45417 | | | | | | | | | | | | | | |  |  |  |
| A | 0007409 | axonogenesis | EN-124k-90-group18372.gs\_75667 | | | | | | | | | | | | | | |  |  |  |
| E | 0007409 | axonogenesis | EN-124k-90-group18375.EN\_iowa\_3673 | | | | | | | | | | | | | | | |  |  |
| A | 0007409 | axonogenesis | EN-124k-90-group18409.gs\_84438 | | | | | | | | | | | | | | |  |  |  |
| A | 0007409 | axonogenesis | EN-124k-90-group18447.gs\_40187 | | | | | | | | | | | | | | |  |  |  |
| A | 0007409 | axonogenesis | EN-124k-90-group18480.gs\_449 | | | | | | | | | | | | | | |  |  |  |
| A | 0007409 | axonogenesis | EN-124k-90-group18526.gs\_58285 | | | | | | | | | | | | | | |  |  |  |
| E | 0007409 | axonogenesis | EN-124k-90-group18538.jgi\_paired\_JGI\_CBBP19133\_rev | | | | | | | | | | | | | | | | | |
| A | 0007409 | axonogenesis | EN-124k-90-group18592.gs\_31083 | | | | | | | | | | | | | | |  |  |  |
| A | 0007409 | axonogenesis | EN-124k-90-group18634.gs\_80302 | | | | | | | | | | | | | | |  |  |  |
| A | 0007409 | axonogenesis | EN-124k-90-group18774.gs\_57540 | | | | | | | | | | | | | | |  |  |  |
| A | 0007409 | axonogenesis | EN-124k-90-group18840.gs\_32731 | | | | | | | | | | | | | | |  |  |  |
| A | 0007409 | axonogenesis | EN-124k-90-group18887.gs\_36301 | | | | | | | | | | | | | | |  |  |  |
| A | 0007409 | axonogenesis | EN-124k-90-group18929.gs\_13816 | | | | | | | | | | | | | | |  |  |  |
| A | 0007409 | axonogenesis | EN-124k-90-group18948.gs\_56541 | | | | | | | | | | | | | | |  |  |  |
| M | 0007409 | axonogenesis | EN-124k-90-group10.Contig1 | | | | | | | | | | | | | |  |  |  |  |
| M | 0007409 | axonogenesis | EN-124k-90-group29.Contig2 | | | | | | | | | | | | | |  |  |  |  |
| M | 0007409 | axonogenesis | EN-124k-90-group29.Contig3 | | | | | | | | | | | | | |  |  |  |  |
| M | 0007409 | axonogenesis | EN-124k-90-group29.Contig7 | | | | | | | | | | | | | |  |  |  |  |
| M | 0007409 | axonogenesis | EN-124k-90-group29.Contig9 | | | | | | | | | | | | | |  |  |  |  |
| M | 0007409 | axonogenesis | EN-124k-90-group29.Contig13 | | | | | | | | | | | | | |  |  |  |  |
| M | 0007409 | axonogenesis | EN-124k-90-group29.Contig15 | | | | | | | | | | | | | |  |  |  |  |
| M | 0007409 | axonogenesis | EN-124k-90-group43.Contig1 | | | | | | | | | | | | | |  |  |  |  |
| M | 0007409 | axonogenesis | EN-124k-90-group63.Contig2 | | | | | | | | | | | | | |  |  |  |  |
| M | 0007409 | axonogenesis | EN-124k-90-group70.Contig3 | | | | | | | | | | | | | |  |  |  |  |
| M | 0007409 | axonogenesis | EN-124k-90-group70.Contig17 | | | | | | | | | | | | | |  |  |  |  |
| M | 0007409 | axonogenesis | EN-124k-90-group75.Contig2 | | | | | | | | | | | | | |  |  |  |  |
| M | 0007409 | axonogenesis | EN-124k-90-group138.Contig1 | | | | | | | | | | | | | |  |  |  |  |
| M | 0007409 | axonogenesis | EN-124k-90-group138.Contig35 | | | | | | | | | | | | | |  |  |  |  |
| M | 0007409 | axonogenesis | EN-124k-90-group138.Contig62 | | | | | | | | | | | | | |  |  |  |  |
| M | 0007409 | axonogenesis | EN-124k-90-group186.Contig2 | | | | | | | | | | | | | |  |  |  |  |
| M | 0007409 | axonogenesis | EN-124k-90-group229.Contig1 | | | | | | | | | | | | | |  |  |  |  |
| M | 0007409 | axonogenesis | EN-124k-90-group230.Contig1 | | | | | | | | | | | | | |  |  |  |  |
| M | 0007409 | axonogenesis | EN-124k-90-group232.Contig1 | | | | | | | | | | | | | |  |  |  |  |
| M | 0007409 | axonogenesis | EN-124k-90-group232.Contig2 | | | | | | | | | | | | | |  |  |  |  |
| M | 0007409 | axonogenesis | EN-124k-90-group232.Contig3 | | | | | | | | | | | | | |  |  |  |  |
| M | 0007409 | axonogenesis | EN-124k-90-group232.Contig5 | | | | | | | | | | | | | |  |  |  |  |
| M | 0007409 | axonogenesis | EN-124k-90-group245.Contig3 | | | | | | | | | | | | | |  |  |  |  |
| M | 0007409 | axonogenesis | EN-124k-90-group245.Contig28 | | | | | | | | | | | | | |  |  |  |  |
| M | 0007409 | axonogenesis | EN-124k-90-group253.Contig1 | | | | | | | | | | | | | |  |  |  |  |
| M | 0007409 | axonogenesis | EN-124k-90-group265.Contig1 | | | | | | | | | | | | | |  |  |  |  |
| M | 0007409 | axonogenesis | EN-124k-90-group270.Contig2 | | | | | | | | | | | | | |  |  |  |  |
| M | 0007409 | axonogenesis | EN-124k-90-group294.Contig1 | | | | | | | | | | | | | |  |  |  |  |
| M | 0007409 | axonogenesis | EN-124k-90-group294.Contig3 | | | | | | | | | | | | | |  |  |  |  |
| M | 0007409 | axonogenesis | EN-124k-90-group304.Contig1 | | | | | | | | | | | | | |  |  |  |  |
| M | 0007409 | axonogenesis | EN-124k-90-group324.Contig1 | | | | | | | | | | | | | |  |  |  |  |
| M | 0007409 | axonogenesis | EN-124k-90-group328.Contig2 | | | | | | | | | | | | | |  |  |  |  |
| M | 0007409 | axonogenesis | EN-124k-90-group382.Contig1 | | | | | | | | | | | | | |  |  |  |  |
| M | 0007409 | axonogenesis | EN-124k-90-group382.Contig2 | | | | | | | | | | | | | |  |  |  |  |
| M | 0007409 | axonogenesis | EN-124k-90-group430.Contig1 | | | | | | | | | | | | | |  |  |  |  |
| M | 0007409 | axonogenesis | EN-124k-90-group462.Contig1 | | | | | | | | | | | | | |  |  |  |  |
| M | 0007409 | axonogenesis | EN-124k-90-group481.Contig4 | | | | | | | | | | | | | |  |  |  |  |
| M | 0007409 | axonogenesis | EN-124k-90-group506.Contig1 | | | | | | | | | | | | | |  |  |  |  |
| M | 0007409 | axonogenesis | EN-124k-90-group506.Contig3 | | | | | | | | | | | | | |  |  |  |  |
| M | 0007409 | axonogenesis | EN-124k-90-group506.Contig4 | | | | | | | | | | | | | |  |  |  |  |
| M | 0007409 | axonogenesis | EN-124k-90-group509.Contig1 | | | | | | | | | | | | | |  |  |  |  |
| M | 0007409 | axonogenesis | EN-124k-90-group509.Contig3 | | | | | | | | | | | | | |  |  |  |  |
| M | 0007409 | axonogenesis | EN-124k-90-group513.Contig1 | | | | | | | | | | | | | |  |  |  |  |
| M | 0007409 | axonogenesis | EN-124k-90-group513.Contig2 | | | | | | | | | | | | | |  |  |  |  |
| M | 0007409 | axonogenesis | EN-124k-90-group516.Contig2 | | | | | | | | | | | | | |  |  |  |  |
| M | 0007409 | axonogenesis | EN-124k-90-group518.Contig2 | | | | | | | | | | | | | |  |  |  |  |
| M | 0007409 | axonogenesis | EN-124k-90-group524.Contig1 | | | | | | | | | | | | | |  |  |  |  |
| M | 0007409 | axonogenesis | EN-124k-90-group524.Contig2 | | | | | | | | | | | | | |  |  |  |  |
| M | 0007409 | axonogenesis | EN-124k-90-group524.Contig3 | | | | | | | | | | | | | |  |  |  |  |
| M | 0007409 | axonogenesis | EN-124k-90-group530.Contig2 | | | | | | | | | | | | | |  |  |  |  |
| M | 0007409 | axonogenesis | EN-124k-90-group543.Contig1 | | | | | | | | | | | | | |  |  |  |  |
| M | 0007409 | axonogenesis | EN-124k-90-group550.Contig1 | | | | | | | | | | | | | |  |  |  |  |
| M | 0007409 | axonogenesis | EN-124k-90-group550.Contig2 | | | | | | | | | | | | | |  |  |  |  |
| M | 0007409 | axonogenesis | EN-124k-90-group564.Contig5 | | | | | | | | | | | | | |  |  |  |  |
| M | 0007409 | axonogenesis | EN-124k-90-group583.Contig1 | | | | | | | | | | | | | |  |  |  |  |
| M | 0007409 | axonogenesis | EN-124k-90-group586.Contig1 | | | | | | | | | | | | | |  |  |  |  |
| M | 0007409 | axonogenesis | EN-124k-90-group586.Contig2 | | | | | | | | | | | | | |  |  |  |  |
| M | 0007409 | axonogenesis | EN-124k-90-group593.Contig1 | | | | | | | | | | | | | |  |  |  |  |
| M | 0007409 | axonogenesis | EN-124k-90-group630.Contig1 | | | | | | | | | | | | | |  |  |  |  |
| M | 0007409 | axonogenesis | EN-124k-90-group630.Contig2 | | | | | | | | | | | | | |  |  |  |  |
| M | 0007409 | axonogenesis | EN-124k-90-group658.Contig1 | | | | | | | | | | | | | |  |  |  |  |
| M | 0007409 | axonogenesis | EN-124k-90-group658.Contig3 | | | | | | | | | | | | | |  |  |  |  |
| M | 0007409 | axonogenesis | EN-124k-90-group674.Contig1 | | | | | | | | | | | | | |  |  |  |  |
| M | 0007409 | axonogenesis | EN-124k-90-group674.Contig2 | | | | | | | | | | | | | |  |  |  |  |
| M | 0007409 | axonogenesis | EN-124k-90-group678.Contig2 | | | | | | | | | | | | | |  |  |  |  |
| M | 0007409 | axonogenesis | EN-124k-90-group682.Contig1 | | | | | | | | | | | | | |  |  |  |  |
| M | 0007409 | axonogenesis | EN-124k-90-group689.Contig4 | | | | | | | | | | | | | |  |  |  |  |
| M | 0007409 | axonogenesis | EN-124k-90-group702.Contig2 | | | | | | | | | | | | | |  |  |  |  |
| M | 0007409 | axonogenesis | EN-124k-90-group716.Contig1 | | | | | | | | | | | | | |  |  |  |  |
| M | 0007409 | axonogenesis | EN-124k-90-group735.Contig1 | | | | | | | | | | | | | |  |  |  |  |
| M | 0007409 | axonogenesis | EN-124k-90-group745.Contig1 | | | | | | | | | | | | | |  |  |  |  |
| M | 0007409 | axonogenesis | EN-124k-90-group745.Contig2 | | | | | | | | | | | | | |  |  |  |  |
| M | 0007409 | axonogenesis | EN-124k-90-group760.Contig4 | | | | | | | | | | | | | |  |  |  |  |
| E | 0007409 | axonogenesis | EN-124k-90-group792.Contig1 | | | | | | | | | | | | | |  |  |  |  |
| M | 0007409 | axonogenesis | EN-124k-90-group849.Contig2 | | | | | | | | | | | | | |  |  |  |  |
| M | 0007409 | axonogenesis | EN-124k-90-group849.Contig3 | | | | | | | | | | | | | |  |  |  |  |
| M | 0007409 | axonogenesis | EN-124k-90-group850.Contig1 | | | | | | | | | | | | | |  |  |  |  |
| M | 0007409 | axonogenesis | EN-124k-90-group868.Contig1 | | | | | | | | | | | | | |  |  |  |  |
| M | 0007409 | axonogenesis | EN-124k-90-group881.Contig1 | | | | | | | | | | | | | |  |  |  |  |
| M | 0007409 | axonogenesis | EN-124k-90-group881.Contig2 | | | | | | | | | | | | | |  |  |  |  |
| M | 0007409 | axonogenesis | EN-124k-90-group881.Contig3 | | | | | | | | | | | | | |  |  |  |  |
| M | 0007409 | axonogenesis | EN-124k-90-group882.Contig2 | | | | | | | | | | | | | |  |  |  |  |
| M | 0007409 | axonogenesis | EN-124k-90-group900.Contig1 | | | | | | | | | | | | | |  |  |  |  |
| M | 0007409 | axonogenesis | EN-124k-90-group908.Contig6 | | | | | | | | | | | | | |  |  |  |  |
| M | 0007409 | axonogenesis | EN-124k-90-group925.Contig1 | | | | | | | | | | | | | |  |  |  |  |
| M | 0007409 | axonogenesis | EN-124k-90-group925.Contig2 | | | | | | | | | | | | | |  |  |  |  |
| M | 0007409 | axonogenesis | EN-124k-90-group925.Contig3 | | | | | | | | | | | | | |  |  |  |  |
| M | 0007409 | axonogenesis | EN-124k-90-group939.Contig1 | | | | | | | | | | | | | |  |  |  |  |
| M | 0007409 | axonogenesis | EN-124k-90-group944.Contig4 | | | | | | | | | | | | | |  |  |  |  |
| M | 0007409 | axonogenesis | EN-124k-90-group945.Contig1 | | | | | | | | | | | | | |  |  |  |  |
| M | 0007409 | axonogenesis | EN-124k-90-group986.Contig2 | | | | | | | | | | | | | |  |  |  |  |
| M | 0007409 | axonogenesis | EN-124k-90-group994.Contig1 | | | | | | | | | | | | | |  |  |  |  |
| M | 0007409 | axonogenesis | EN-124k-90-group998.Contig1 | | | | | | | | | | | | | |  |  |  |  |
| M | 0007409 | axonogenesis | EN-124k-90-group1001.Contig1 | | | | | | | | | | | | | |  |  |  |  |
| M | 0007409 | axonogenesis | EN-124k-90-group1001.Contig4 | | | | | | | | | | | | | |  |  |  |  |
| M | 0007409 | axonogenesis | EN-124k-90-group1007.Contig1 | | | | | | | | | | | | | |  |  |  |  |
| M | 0007409 | axonogenesis | EN-124k-90-group1021.Contig1 | | | | | | | | | | | | | |  |  |  |  |
| M | 0007409 | axonogenesis | EN-124k-90-group1021.Contig2 | | | | | | | | | | | | | |  |  |  |  |
| M | 0007409 | axonogenesis | EN-124k-90-group1071.Contig1 | | | | | | | | | | | | | |  |  |  |  |
| M | 0007409 | axonogenesis | EN-124k-90-group1071.Contig2 | | | | | | | | | | | | | |  |  |  |  |
| M | 0007409 | axonogenesis | EN-124k-90-group1071.Contig3 | | | | | | | | | | | | | |  |  |  |  |
| M | 0007409 | axonogenesis | EN-124k-90-group1076.Contig1 | | | | | | | | | | | | | |  |  |  |  |
| M | 0007409 | axonogenesis | EN-124k-90-group1091.Contig1 | | | | | | | | | | | | | |  |  |  |  |
| M | 0007409 | axonogenesis | EN-124k-90-group1091.Contig3 | | | | | | | | | | | | | |  |  |  |  |
| E | 0007409 | axonogenesis | EN-124k-90-group1092.Contig1 | | | | | | | | | | | | | |  |  |  |  |
| M | 0007409 | axonogenesis | EN-124k-90-group1106.Contig1 | | | | | | | | | | | | | |  |  |  |  |
| M | 0007409 | axonogenesis | EN-124k-90-group1135.Contig1 | | | | | | | | | | | | | |  |  |  |  |
| M | 0007409 | axonogenesis | EN-124k-90-group1135.Contig3 | | | | | | | | | | | | | |  |  |  |  |
| M | 0007409 | axonogenesis | EN-124k-90-group1135.Contig4 | | | | | | | | | | | | | |  |  |  |  |
| M | 0007409 | axonogenesis | EN-124k-90-group1135.Contig5 | | | | | | | | | | | | | |  |  |  |  |
| M | 0007409 | axonogenesis | EN-124k-90-group1135.Contig7 | | | | | | | | | | | | | |  |  |  |  |
| M | 0007409 | axonogenesis | EN-124k-90-group1142.Contig8 | | | | | | | | | | | | | |  |  |  |  |
| M | 0007409 | axonogenesis | EN-124k-90-group1142.Contig10 | | | | | | | | | | | | | | |  |  |  |
| M | 0007409 | axonogenesis | EN-124k-90-group1153.Contig1 | | | | | | | | | | | | | |  |  |  |  |
| M | 0007409 | axonogenesis | EN-124k-90-group1161.Contig1 | | | | | | | | | | | | | |  |  |  |  |
| M | 0007409 | axonogenesis | EN-124k-90-group1161.Contig2 | | | | | | | | | | | | | |  |  |  |  |
| M | 0007409 | axonogenesis | EN-124k-90-group1167.Contig2 | | | | | | | | | | | | | |  |  |  |  |
| M | 0007409 | axonogenesis | EN-124k-90-group1167.Contig3 | | | | | | | | | | | | | |  |  |  |  |
| M | 0007409 | axonogenesis | EN-124k-90-group1182.Contig1 | | | | | | | | | | | | | |  |  |  |  |
| M | 0007409 | axonogenesis | EN-124k-90-group1182.Contig2 | | | | | | | | | | | | | |  |  |  |  |
| M | 0007409 | axonogenesis | EN-124k-90-group1182.Contig3 | | | | | | | | | | | | | |  |  |  |  |
| M | 0007409 | axonogenesis | EN-124k-90-group1182.Contig4 | | | | | | | | | | | | | |  |  |  |  |
| M | 0007409 | axonogenesis | EN-124k-90-group1212.Contig1 | | | | | | | | | | | | | |  |  |  |  |
| M | 0007409 | axonogenesis | EN-124k-90-group1215.Contig3 | | | | | | | | | | | | | |  |  |  |  |
| M | 0007409 | axonogenesis | EN-124k-90-group1215.Contig4 | | | | | | | | | | | | | |  |  |  |  |
| M | 0007409 | axonogenesis | EN-124k-90-group1272.Contig1 | | | | | | | | | | | | | |  |  |  |  |
| M | 0007409 | axonogenesis | EN-124k-90-group1290.Contig1 | | | | | | | | | | | | | |  |  |  |  |
| M | 0007409 | axonogenesis | EN-124k-90-group1290.Contig2 | | | | | | | | | | | | | |  |  |  |  |
| M | 0007409 | axonogenesis | EN-124k-90-group1290.Contig3 | | | | | | | | | | | | | |  |  |  |  |
| M | 0007409 | axonogenesis | EN-124k-90-group1290.Contig5 | | | | | | | | | | | | | |  |  |  |  |
| M | 0007409 | axonogenesis | EN-124k-90-group1290.Contig6 | | | | | | | | | | | | | |  |  |  |  |
| M | 0007409 | axonogenesis | EN-124k-90-group1290.Contig8 | | | | | | | | | | | | | |  |  |  |  |
| M | 0007409 | axonogenesis | EN-124k-90-group1290.Contig10 | | | | | | | | | | | | | | |  |  |  |
| M | 0007409 | axonogenesis | EN-124k-90-group1294.Contig1 | | | | | | | | | | | | | |  |  |  |  |
| M | 0007409 | axonogenesis | EN-124k-90-group1302.Contig2 | | | | | | | | | | | | | |  |  |  |  |
| M | 0007409 | axonogenesis | EN-124k-90-group1302.Contig3 | | | | | | | | | | | | | |  |  |  |  |
| M | 0007409 | axonogenesis | EN-124k-90-group1302.Contig4 | | | | | | | | | | | | | |  |  |  |  |
| M | 0007409 | axonogenesis | EN-124k-90-group1302.Contig6 | | | | | | | | | | | | | |  |  |  |  |
| M | 0007409 | axonogenesis | EN-124k-90-group1304.Contig2 | | | | | | | | | | | | | |  |  |  |  |
| M | 0007409 | axonogenesis | EN-124k-90-group1304.Contig4 | | | | | | | | | | | | | |  |  |  |  |
| M | 0007409 | axonogenesis | EN-124k-90-group1304.Contig8 | | | | | | | | | | | | | |  |  |  |  |
| E | 0007409 | axonogenesis | EN-124k-90-group1344.Contig1 | | | | | | | | | | | | | |  |  |  |  |
| M | 0007409 | axonogenesis | EN-124k-90-group1351.Contig1 | | | | | | | | | | | | | |  |  |  |  |
| M | 0007409 | axonogenesis | EN-124k-90-group1373.Contig1 | | | | | | | | | | | | | |  |  |  |  |
| M | 0007409 | axonogenesis | EN-124k-90-group1408.Contig1 | | | | | | | | | | | | | |  |  |  |  |
| M | 0007409 | axonogenesis | EN-124k-90-group1408.Contig2 | | | | | | | | | | | | | |  |  |  |  |
| M | 0007409 | axonogenesis | EN-124k-90-group1423.Contig14 | | | | | | | | | | | | | | |  |  |  |
| M | 0007409 | axonogenesis | EN-124k-90-group1423.Contig20 | | | | | | | | | | | | | | |  |  |  |
| M | 0007409 | axonogenesis | EN-124k-90-group1423.Contig22 | | | | | | | | | | | | | | |  |  |  |
| M | 0007409 | axonogenesis | EN-124k-90-group1456.Contig4 | | | | | | | | | | | | | |  |  |  |  |
| M | 0007409 | axonogenesis | EN-124k-90-group1456.Contig7 | | | | | | | | | | | | | |  |  |  |  |
| M | 0007409 | axonogenesis | EN-124k-90-group1456.Contig8 | | | | | | | | | | | | | |  |  |  |  |
| M | 0007409 | axonogenesis | EN-124k-90-group1462.Contig1 | | | | | | | | | | | | | |  |  |  |  |
| M | 0007409 | axonogenesis | EN-124k-90-group1465.Contig1 | | | | | | | | | | | | | |  |  |  |  |
| M | 0007409 | axonogenesis | EN-124k-90-group1465.Contig2 | | | | | | | | | | | | | |  |  |  |  |
| A | 0007409 | axonogenesis | EN-124k-90-group1482.Contig1 | | | | | | | | | | | | | |  |  |  |  |
| M | 0007409 | axonogenesis | EN-124k-90-group1493.Contig2 | | | | | | | | | | | | | |  |  |  |  |
| A | 0007409 | axonogenesis | EN-124k-90-group1521.Contig1 | | | | | | | | | | | | | |  |  |  |  |
| A | 0007409 | axonogenesis | EN-124k-90-group1521.Contig2 | | | | | | | | | | | | | |  |  |  |  |
| A | 0007409 | axonogenesis | EN-124k-90-group1521.Contig3 | | | | | | | | | | | | | |  |  |  |  |
| M | 0007409 | axonogenesis | EN-124k-90-group1542.Contig1 | | | | | | | | | | | | | |  |  |  |  |
| M | 0007409 | axonogenesis | EN-124k-90-group1549.Contig1 | | | | | | | | | | | | | |  |  |  |  |
| M | 0007409 | axonogenesis | EN-124k-90-group1554.Contig1 | | | | | | | | | | | | | |  |  |  |  |
| M | 0007409 | axonogenesis | EN-124k-90-group1585.Contig2 | | | | | | | | | | | | | |  |  |  |  |
| M | 0007409 | axonogenesis | EN-124k-90-group1642.Contig5 | | | | | | | | | | | | | |  |  |  |  |
| M | 0007409 | axonogenesis | EN-124k-90-group1654.Contig1 | | | | | | | | | | | | | |  |  |  |  |
| M | 0007409 | axonogenesis | EN-124k-90-group1654.Contig2 | | | | | | | | | | | | | |  |  |  |  |
| M | 0007409 | axonogenesis | EN-124k-90-group1699.Contig1 | | | | | | | | | | | | | |  |  |  |  |
| M | 0007409 | axonogenesis | EN-124k-90-group1699.Contig3 | | | | | | | | | | | | | |  |  |  |  |
| M | 0007409 | axonogenesis | EN-124k-90-group1726.Contig2 | | | | | | | | | | | | | |  |  |  |  |
| M | 0007409 | axonogenesis | EN-124k-90-group1728.Contig2 | | | | | | | | | | | | | |  |  |  |  |
| M | 0007409 | axonogenesis | EN-124k-90-group1735.Contig4 | | | | | | | | | | | | | |  |  |  |  |
| M | 0007409 | axonogenesis | EN-124k-90-group1735.Contig6 | | | | | | | | | | | | | |  |  |  |  |
| M | 0007409 | axonogenesis | EN-124k-90-group1752.Contig1 | | | | | | | | | | | | | |  |  |  |  |
| M | 0007409 | axonogenesis | EN-124k-90-group1752.Contig2 | | | | | | | | | | | | | |  |  |  |  |
| M | 0007409 | axonogenesis | EN-124k-90-group1752.Contig3 | | | | | | | | | | | | | |  |  |  |  |
| M | 0007409 | axonogenesis | EN-124k-90-group1752.Contig4 | | | | | | | | | | | | | |  |  |  |  |
| M | 0007409 | axonogenesis | EN-124k-90-group1752.Contig5 | | | | | | | | | | | | | |  |  |  |  |
| M | 0007409 | axonogenesis | EN-124k-90-group1752.Contig6 | | | | | | | | | | | | | |  |  |  |  |
| M | 0007409 | axonogenesis | EN-124k-90-group1757.Contig1 | | | | | | | | | | | | | |  |  |  |  |
| M | 0007409 | axonogenesis | EN-124k-90-group1770.Contig2 | | | | | | | | | | | | | |  |  |  |  |
| M | 0007409 | axonogenesis | EN-124k-90-group1772.Contig1 | | | | | | | | | | | | | |  |  |  |  |
| M | 0007409 | axonogenesis | EN-124k-90-group1781.Contig1 | | | | | | | | | | | | | |  |  |  |  |
| M | 0007409 | axonogenesis | EN-124k-90-group1782.Contig1 | | | | | | | | | | | | | |  |  |  |  |
| M | 0007409 | axonogenesis | EN-124k-90-group1798.Contig2 | | | | | | | | | | | | | |  |  |  |  |
| M | 0007409 | axonogenesis | EN-124k-90-group1798.Contig3 | | | | | | | | | | | | | |  |  |  |  |
| M | 0007409 | axonogenesis | EN-124k-90-group1819.Contig1 | | | | | | | | | | | | | |  |  |  |  |
| M | 0007409 | axonogenesis | EN-124k-90-group1819.Contig3 | | | | | | | | | | | | | |  |  |  |  |
| M | 0007409 | axonogenesis | EN-124k-90-group1834.Contig1 | | | | | | | | | | | | | |  |  |  |  |
| M | 0007409 | axonogenesis | EN-124k-90-group1861.Contig1 | | | | | | | | | | | | | |  |  |  |  |
| M | 0007409 | axonogenesis | EN-124k-90-group1861.Contig2 | | | | | | | | | | | | | |  |  |  |  |
| M | 0007409 | axonogenesis | EN-124k-90-group1867.Contig1 | | | | | | | | | | | | | |  |  |  |  |
| M | 0007409 | axonogenesis | EN-124k-90-group1869.Contig3 | | | | | | | | | | | | | |  |  |  |  |
| M | 0007409 | axonogenesis | EN-124k-90-group1873.Contig1 | | | | | | | | | | | | | |  |  |  |  |
| M | 0007409 | axonogenesis | EN-124k-90-group1907.Contig1 | | | | | | | | | | | | | |  |  |  |  |
| M | 0007409 | axonogenesis | EN-124k-90-group1909.Contig1 | | | | | | | | | | | | | |  |  |  |  |
| M | 0007409 | axonogenesis | EN-124k-90-group1909.Contig2 | | | | | | | | | | | | | |  |  |  |  |
| M | 0007409 | axonogenesis | EN-124k-90-group1915.Contig1 | | | | | | | | | | | | | |  |  |  |  |
| M | 0007409 | axonogenesis | EN-124k-90-group1933.Contig1 | | | | | | | | | | | | | |  |  |  |  |
| M | 0007409 | axonogenesis | EN-124k-90-group1933.Contig2 | | | | | | | | | | | | | |  |  |  |  |
| M | 0007409 | axonogenesis | EN-124k-90-group1938.Contig1 | | | | | | | | | | | | | |  |  |  |  |
| M | 0007409 | axonogenesis | EN-124k-90-group1958.Contig1 | | | | | | | | | | | | | |  |  |  |  |
| M | 0007409 | axonogenesis | EN-124k-90-group1960.Contig1 | | | | | | | | | | | | | |  |  |  |  |
| M | 0007409 | axonogenesis | EN-124k-90-group1980.Contig1 | | | | | | | | | | | | | |  |  |  |  |
| A | 0007409 | axonogenesis | EN-124k-90-group1993.Contig1 | | | | | | | | | | | | | |  |  |  |  |
| M | 0007409 | axonogenesis | EN-124k-90-group2035.Contig1 | | | | | | | | | | | | | |  |  |  |  |
| E | 0007409 | axonogenesis | EN-124k-90-group2041.Contig1 | | | | | | | | | | | | | |  |  |  |  |
| M | 0007409 | axonogenesis | EN-124k-90-group2069.Contig3 | | | | | | | | | | | | | |  |  |  |  |
| M | 0007409 | axonogenesis | EN-124k-90-group2074.Contig1 | | | | | | | | | | | | | |  |  |  |  |
| M | 0007409 | axonogenesis | EN-124k-90-group2075.Contig2 | | | | | | | | | | | | | |  |  |  |  |
| M | 0007409 | axonogenesis | EN-124k-90-group2077.Contig2 | | | | | | | | | | | | | |  |  |  |  |
| M | 0007409 | axonogenesis | EN-124k-90-group2077.Contig3 | | | | | | | | | | | | | |  |  |  |  |
| M | 0007409 | axonogenesis | EN-124k-90-group2081.Contig1 | | | | | | | | | | | | | |  |  |  |  |
| M | 0007409 | axonogenesis | EN-124k-90-group2081.Contig3 | | | | | | | | | | | | | |  |  |  |  |
| M | 0007409 | axonogenesis | EN-124k-90-group2097.Contig1 | | | | | | | | | | | | | |  |  |  |  |
| M | 0007409 | axonogenesis | EN-124k-90-group2099.Contig1 | | | | | | | | | | | | | |  |  |  |  |
| M | 0007409 | axonogenesis | EN-124k-90-group2127.Contig1 | | | | | | | | | | | | | |  |  |  |  |
| M | 0007409 | axonogenesis | EN-124k-90-group2132.Contig1 | | | | | | | | | | | | | |  |  |  |  |
| M | 0007409 | axonogenesis | EN-124k-90-group2145.Contig1 | | | | | | | | | | | | | |  |  |  |  |
| M | 0007409 | axonogenesis | EN-124k-90-group2163.Contig1 | | | | | | | | | | | | | |  |  |  |  |
| M | 0007409 | axonogenesis | EN-124k-90-group2200.Contig1 | | | | | | | | | | | | | |  |  |  |  |
| M | 0007409 | axonogenesis | EN-124k-90-group2221.Contig2 | | | | | | | | | | | | | |  |  |  |  |
| M | 0007409 | axonogenesis | EN-124k-90-group2223.Contig1 | | | | | | | | | | | | | |  |  |  |  |
| M | 0007409 | axonogenesis | EN-124k-90-group2239.Contig1 | | | | | | | | | | | | | |  |  |  |  |
| M | 0007409 | axonogenesis | EN-124k-90-group2264.Contig1 | | | | | | | | | | | | | |  |  |  |  |
| M | 0007409 | axonogenesis | EN-124k-90-group2264.Contig2 | | | | | | | | | | | | | |  |  |  |  |
| M | 0007409 | axonogenesis | EN-124k-90-group2264.Contig3 | | | | | | | | | | | | | |  |  |  |  |
| M | 0007409 | axonogenesis | EN-124k-90-group2268.Contig2 | | | | | | | | | | | | | |  |  |  |  |
| M | 0007409 | axonogenesis | EN-124k-90-group2287.Contig2 | | | | | | | | | | | | | |  |  |  |  |
| M | 0007409 | axonogenesis | EN-124k-90-group2289.Contig1 | | | | | | | | | | | | | |  |  |  |  |
| A | 0007409 | axonogenesis | EN-124k-90-group2305.Contig1 | | | | | | | | | | | | | |  |  |  |  |
| M | 0007409 | axonogenesis | EN-124k-90-group2327.Contig2 | | | | | | | | | | | | | |  |  |  |  |
| M | 0007409 | axonogenesis | EN-124k-90-group2397.Contig1 | | | | | | | | | | | | | |  |  |  |  |
| M | 0007409 | axonogenesis | EN-124k-90-group2397.Contig2 | | | | | | | | | | | | | |  |  |  |  |
| M | 0007409 | axonogenesis | EN-124k-90-group2409.Contig1 | | | | | | | | | | | | | |  |  |  |  |
| M | 0007409 | axonogenesis | EN-124k-90-group2409.Contig3 | | | | | | | | | | | | | |  |  |  |  |
| M | 0007409 | axonogenesis | EN-124k-90-group2409.Contig4 | | | | | | | | | | | | | |  |  |  |  |
| M | 0007409 | axonogenesis | EN-124k-90-group2425.Contig1 | | | | | | | | | | | | | |  |  |  |  |
| M | 0007409 | axonogenesis | EN-124k-90-group2425.Contig7 | | | | | | | | | | | | | |  |  |  |  |
| M | 0007409 | axonogenesis | EN-124k-90-group2425.Contig8 | | | | | | | | | | | | | |  |  |  |  |
| M | 0007409 | axonogenesis | EN-124k-90-group2428.Contig1 | | | | | | | | | | | | | |  |  |  |  |
| M | 0007409 | axonogenesis | EN-124k-90-group2430.Contig1 | | | | | | | | | | | | | |  |  |  |  |
| M | 0007409 | axonogenesis | EN-124k-90-group2430.Contig2 | | | | | | | | | | | | | |  |  |  |  |
| M | 0007409 | axonogenesis | EN-124k-90-group2438.Contig1 | | | | | | | | | | | | | |  |  |  |  |
| M | 0007409 | axonogenesis | EN-124k-90-group2443.Contig1 | | | | | | | | | | | | | |  |  |  |  |
| M | 0007409 | axonogenesis | EN-124k-90-group2449.Contig1 | | | | | | | | | | | | | |  |  |  |  |
| M | 0007409 | axonogenesis | EN-124k-90-group2449.Contig2 | | | | | | | | | | | | | |  |  |  |  |
| M | 0007409 | axonogenesis | EN-124k-90-group2451.Contig1 | | | | | | | | | | | | | |  |  |  |  |
| M | 0007409 | axonogenesis | EN-124k-90-group2455.Contig1 | | | | | | | | | | | | | |  |  |  |  |
| M | 0007409 | axonogenesis | EN-124k-90-group2472.Contig2 | | | | | | | | | | | | | |  |  |  |  |
| M | 0007409 | axonogenesis | EN-124k-90-group2472.Contig3 | | | | | | | | | | | | | |  |  |  |  |
| M | 0007409 | axonogenesis | EN-124k-90-group2489.Contig1 | | | | | | | | | | | | | |  |  |  |  |
| M | 0007409 | axonogenesis | EN-124k-90-group2491.Contig1 | | | | | | | | | | | | | |  |  |  |  |
| M | 0007409 | axonogenesis | EN-124k-90-group2495.Contig1 | | | | | | | | | | | | | |  |  |  |  |
| M | 0007409 | axonogenesis | EN-124k-90-group2495.Contig2 | | | | | | | | | | | | | |  |  |  |  |
| M | 0007409 | axonogenesis | EN-124k-90-group2498.Contig5 | | | | | | | | | | | | | |  |  |  |  |
| M | 0007409 | axonogenesis | EN-124k-90-group2498.Contig7 | | | | | | | | | | | | | |  |  |  |  |
| M | 0007409 | axonogenesis | EN-124k-90-group2509.Contig2 | | | | | | | | | | | | | |  |  |  |  |
| M | 0007409 | axonogenesis | EN-124k-90-group2525.Contig1 | | | | | | | | | | | | | |  |  |  |  |
| M | 0007409 | axonogenesis | EN-124k-90-group2525.Contig2 | | | | | | | | | | | | | |  |  |  |  |
| M | 0007409 | axonogenesis | EN-124k-90-group2525.Contig3 | | | | | | | | | | | | | |  |  |  |  |
| M | 0007409 | axonogenesis | EN-124k-90-group2525.Contig4 | | | | | | | | | | | | | |  |  |  |  |
| M | 0007409 | axonogenesis | EN-124k-90-group2537.Contig1 | | | | | | | | | | | | | |  |  |  |  |
| M | 0007409 | axonogenesis | EN-124k-90-group2582.Contig1 | | | | | | | | | | | | | |  |  |  |  |
| M | 0007409 | axonogenesis | EN-124k-90-group2584.Contig2 | | | | | | | | | | | | | |  |  |  |  |
| M | 0007409 | axonogenesis | EN-124k-90-group2600.Contig2 | | | | | | | | | | | | | |  |  |  |  |
| M | 0007409 | axonogenesis | EN-124k-90-group2606.Contig1 | | | | | | | | | | | | | |  |  |  |  |
| M | 0007409 | axonogenesis | EN-124k-90-group2619.Contig1 | | | | | | | | | | | | | |  |  |  |  |
| M | 0007409 | axonogenesis | EN-124k-90-group2619.Contig2 | | | | | | | | | | | | | |  |  |  |  |
| M | 0007409 | axonogenesis | EN-124k-90-group2651.Contig1 | | | | | | | | | | | | | |  |  |  |  |
| M | 0007409 | axonogenesis | EN-124k-90-group2681.Contig1 | | | | | | | | | | | | | |  |  |  |  |
| M | 0007409 | axonogenesis | EN-124k-90-group2686.Contig1 | | | | | | | | | | | | | |  |  |  |  |
| M | 0007409 | axonogenesis | EN-124k-90-group2806.Contig1 | | | | | | | | | | | | | |  |  |  |  |
| M | 0007409 | axonogenesis | EN-124k-90-group2811.Contig1 | | | | | | | | | | | | | |  |  |  |  |
| M | 0007409 | axonogenesis | EN-124k-90-group2811.Contig2 | | | | | | | | | | | | | |  |  |  |  |
| M | 0007409 | axonogenesis | EN-124k-90-group2830.Contig1 | | | | | | | | | | | | | |  |  |  |  |
| M | 0007409 | axonogenesis | EN-124k-90-group2834.Contig2 | | | | | | | | | | | | | |  |  |  |  |
| M | 0007409 | axonogenesis | EN-124k-90-group2835.Contig1 | | | | | | | | | | | | | |  |  |  |  |
| M | 0007409 | axonogenesis | EN-124k-90-group2835.Contig2 | | | | | | | | | | | | | |  |  |  |  |
| M | 0007409 | axonogenesis | EN-124k-90-group2841.Contig1 | | | | | | | | | | | | | |  |  |  |  |
| M | 0007409 | axonogenesis | EN-124k-90-group2846.Contig1 | | | | | | | | | | | | | |  |  |  |  |
| M | 0007409 | axonogenesis | EN-124k-90-group2850.Contig2 | | | | | | | | | | | | | |  |  |  |  |
| M | 0007409 | axonogenesis | EN-124k-90-group2903.Contig1 | | | | | | | | | | | | | |  |  |  |  |
| M | 0007409 | axonogenesis | EN-124k-90-group2917.Contig1 | | | | | | | | | | | | | |  |  |  |  |
| M | 0007409 | axonogenesis | EN-124k-90-group2917.Contig2 | | | | | | | | | | | | | |  |  |  |  |
| M | 0007409 | axonogenesis | EN-124k-90-group2917.Contig3 | | | | | | | | | | | | | |  |  |  |  |
| M | 0007409 | axonogenesis | EN-124k-90-group2917.Contig4 | | | | | | | | | | | | | |  |  |  |  |
| M | 0007409 | axonogenesis | EN-124k-90-group2930.Contig1 | | | | | | | | | | | | | |  |  |  |  |
| M | 0007409 | axonogenesis | EN-124k-90-group2940.Contig2 | | | | | | | | | | | | | |  |  |  |  |
| M | 0007409 | axonogenesis | EN-124k-90-group3018.Contig1 | | | | | | | | | | | | | |  |  |  |  |
| M | 0007409 | axonogenesis | EN-124k-90-group3018.Contig2 | | | | | | | | | | | | | |  |  |  |  |
| M | 0007409 | axonogenesis | EN-124k-90-group3033.Contig1 | | | | | | | | | | | | | |  |  |  |  |
| M | 0007409 | axonogenesis | EN-124k-90-group3037.Contig1 | | | | | | | | | | | | | |  |  |  |  |
| M | 0007409 | axonogenesis | EN-124k-90-group3037.Contig2 | | | | | | | | | | | | | |  |  |  |  |
| M | 0007409 | axonogenesis | EN-124k-90-group3067.Contig1 | | | | | | | | | | | | | |  |  |  |  |
| M | 0007409 | axonogenesis | EN-124k-90-group3082.Contig2 | | | | | | | | | | | | | |  |  |  |  |
| M | 0007409 | axonogenesis | EN-124k-90-group3092.Contig2 | | | | | | | | | | | | | |  |  |  |  |
| M | 0007409 | axonogenesis | EN-124k-90-group3114.Contig1 | | | | | | | | | | | | | |  |  |  |  |
| M | 0007409 | axonogenesis | EN-124k-90-group3114.Contig3 | | | | | | | | | | | | | |  |  |  |  |
| M | 0007409 | axonogenesis | EN-124k-90-group3157.Contig1 | | | | | | | | | | | | | |  |  |  |  |
| M | 0007409 | axonogenesis | EN-124k-90-group3157.Contig5 | | | | | | | | | | | | | |  |  |  |  |
| M | 0007409 | axonogenesis | EN-124k-90-group3158.Contig2 | | | | | | | | | | | | | |  |  |  |  |
| M | 0007409 | axonogenesis | EN-124k-90-group3202.Contig1 | | | | | | | | | | | | | |  |  |  |  |
| M | 0007409 | axonogenesis | EN-124k-90-group3202.Contig2 | | | | | | | | | | | | | |  |  |  |  |
| M | 0007409 | axonogenesis | EN-124k-90-group3202.Contig3 | | | | | | | | | | | | | |  |  |  |  |
| M | 0007409 | axonogenesis | EN-124k-90-group3222.Contig1 | | | | | | | | | | | | | |  |  |  |  |
| M | 0007409 | axonogenesis | EN-124k-90-group3223.Contig2 | | | | | | | | | | | | | |  |  |  |  |
| M | 0007409 | axonogenesis | EN-124k-90-group3238.Contig1 | | | | | | | | | | | | | |  |  |  |  |
| E | 0007409 | axonogenesis | EN-124k-90-group3241.Contig1 | | | | | | | | | | | | | |  |  |  |  |
| M | 0007409 | axonogenesis | EN-124k-90-group3242.Contig1 | | | | | | | | | | | | | |  |  |  |  |
| M | 0007409 | axonogenesis | EN-124k-90-group3245.Contig2 | | | | | | | | | | | | | |  |  |  |  |
| M | 0007409 | axonogenesis | EN-124k-90-group3257.Contig1 | | | | | | | | | | | | | |  |  |  |  |
| M | 0007409 | axonogenesis | EN-124k-90-group3292.Contig1 | | | | | | | | | | | | | |  |  |  |  |
| M | 0007409 | axonogenesis | EN-124k-90-group3299.Contig1 | | | | | | | | | | | | | |  |  |  |  |
| M | 0007409 | axonogenesis | EN-124k-90-group3313.Contig1 | | | | | | | | | | | | | |  |  |  |  |
| M | 0007409 | axonogenesis | EN-124k-90-group3313.Contig2 | | | | | | | | | | | | | |  |  |  |  |
| M | 0007409 | axonogenesis | EN-124k-90-group3313.Contig3 | | | | | | | | | | | | | |  |  |  |  |
| M | 0007409 | axonogenesis | EN-124k-90-group3315.Contig2 | | | | | | | | | | | | | |  |  |  |  |
| M | 0007409 | axonogenesis | EN-124k-90-group3315.Contig5 | | | | | | | | | | | | | |  |  |  |  |
| M | 0007409 | axonogenesis | EN-124k-90-group3315.Contig6 | | | | | | | | | | | | | |  |  |  |  |
| M | 0007409 | axonogenesis | EN-124k-90-group3316.Contig1 | | | | | | | | | | | | | |  |  |  |  |
| M | 0007409 | axonogenesis | EN-124k-90-group3343.Contig1 | | | | | | | | | | | | | |  |  |  |  |
| E | 0007409 | axonogenesis | EN-124k-90-group3343.Contig2 | | | | | | | | | | | | | |  |  |  |  |
| M | 0007409 | axonogenesis | EN-124k-90-group3357.Contig1 | | | | | | | | | | | | | |  |  |  |  |
| M | 0007409 | axonogenesis | EN-124k-90-group3365.Contig1 | | | | | | | | | | | | | |  |  |  |  |
| M | 0007409 | axonogenesis | EN-124k-90-group3394.Contig1 | | | | | | | | | | | | | |  |  |  |  |
| M | 0007409 | axonogenesis | EN-124k-90-group3394.Contig2 | | | | | | | | | | | | | |  |  |  |  |
| M | 0007409 | axonogenesis | EN-124k-90-group3394.Contig3 | | | | | | | | | | | | | |  |  |  |  |
| M | 0007409 | axonogenesis | EN-124k-90-group3394.Contig4 | | | | | | | | | | | | | |  |  |  |  |
| M | 0007409 | axonogenesis | EN-124k-90-group3398.Contig1 | | | | | | | | | | | | | |  |  |  |  |
| M | 0007409 | axonogenesis | EN-124k-90-group3408.Contig1 | | | | | | | | | | | | | |  |  |  |  |
| M | 0007409 | axonogenesis | EN-124k-90-group3408.Contig2 | | | | | | | | | | | | | |  |  |  |  |
| M | 0007409 | axonogenesis | EN-124k-90-group3477.Contig1 | | | | | | | | | | | | | |  |  |  |  |
| M | 0007409 | axonogenesis | EN-124k-90-group3479.Contig1 | | | | | | | | | | | | | |  |  |  |  |
| M | 0007409 | axonogenesis | EN-124k-90-group3509.Contig1 | | | | | | | | | | | | | |  |  |  |  |
| M | 0007409 | axonogenesis | EN-124k-90-group3520.Contig1 | | | | | | | | | | | | | |  |  |  |  |
| M | 0007409 | axonogenesis | EN-124k-90-group3522.Contig1 | | | | | | | | | | | | | |  |  |  |  |
| M | 0007409 | axonogenesis | EN-124k-90-group3537.Contig1 | | | | | | | | | | | | | |  |  |  |  |
| M | 0007409 | axonogenesis | EN-124k-90-group3577.Contig1 | | | | | | | | | | | | | |  |  |  |  |
| M | 0007409 | axonogenesis | EN-124k-90-group3577.Contig2 | | | | | | | | | | | | | |  |  |  |  |
| M | 0007409 | axonogenesis | EN-124k-90-group3584.Contig1 | | | | | | | | | | | | | |  |  |  |  |
| M | 0007409 | axonogenesis | EN-124k-90-group3586.Contig1 | | | | | | | | | | | | | |  |  |  |  |
| M | 0007409 | axonogenesis | EN-124k-90-group3590.Contig1 | | | | | | | | | | | | | |  |  |  |  |
| M | 0007409 | axonogenesis | EN-124k-90-group3594.Contig1 | | | | | | | | | | | | | |  |  |  |  |
| M | 0007409 | axonogenesis | EN-124k-90-group3594.Contig4 | | | | | | | | | | | | | |  |  |  |  |
| M | 0007409 | axonogenesis | EN-124k-90-group3606.Contig2 | | | | | | | | | | | | | |  |  |  |  |
| E | 0007409 | axonogenesis | EN-124k-90-group3611.Contig1 | | | | | | | | | | | | | |  |  |  |  |
| M | 0007409 | axonogenesis | EN-124k-90-group3660.Contig1 | | | | | | | | | | | | | |  |  |  |  |
| M | 0007409 | axonogenesis | EN-124k-90-group3670.Contig1 | | | | | | | | | | | | | |  |  |  |  |
| M | 0007409 | axonogenesis | EN-124k-90-group3700.Contig1 | | | | | | | | | | | | | |  |  |  |  |
| E | 0007409 | axonogenesis | EN-124k-90-group3702.Contig1 | | | | | | | | | | | | | |  |  |  |  |
| M | 0007409 | axonogenesis | EN-124k-90-group3713.Contig1 | | | | | | | | | | | | | |  |  |  |  |
| M | 0007409 | axonogenesis | EN-124k-90-group3753.Contig1 | | | | | | | | | | | | | |  |  |  |  |
| M | 0007409 | axonogenesis | EN-124k-90-group3753.Contig2 | | | | | | | | | | | | | |  |  |  |  |
| M | 0007409 | axonogenesis | EN-124k-90-group3810.Contig1 | | | | | | | | | | | | | |  |  |  |  |
| M | 0007409 | axonogenesis | EN-124k-90-group3816.Contig1 | | | | | | | | | | | | | |  |  |  |  |
| M | 0007409 | axonogenesis | EN-124k-90-group3830.Contig1 | | | | | | | | | | | | | |  |  |  |  |
| M | 0007409 | axonogenesis | EN-124k-90-group3849.Contig3 | | | | | | | | | | | | | |  |  |  |  |
| M | 0007409 | axonogenesis | EN-124k-90-group3872.Contig3 | | | | | | | | | | | | | |  |  |  |  |
| M | 0007409 | axonogenesis | EN-124k-90-group3872.Contig4 | | | | | | | | | | | | | |  |  |  |  |
| M | 0007409 | axonogenesis | EN-124k-90-group3876.Contig1 | | | | | | | | | | | | | |  |  |  |  |
| M | 0007409 | axonogenesis | EN-124k-90-group3882.Contig1 | | | | | | | | | | | | | |  |  |  |  |
| M | 0007409 | axonogenesis | EN-124k-90-group3882.Contig2 | | | | | | | | | | | | | |  |  |  |  |
| M | 0007409 | axonogenesis | EN-124k-90-group3883.Contig1 | | | | | | | | | | | | | |  |  |  |  |
| M | 0007409 | axonogenesis | EN-124k-90-group3883.Contig2 | | | | | | | | | | | | | |  |  |  |  |
| M | 0007409 | axonogenesis | EN-124k-90-group3883.Contig3 | | | | | | | | | | | | | |  |  |  |  |
| M | 0007409 | axonogenesis | EN-124k-90-group3883.Contig4 | | | | | | | | | | | | | |  |  |  |  |
| M | 0007409 | axonogenesis | EN-124k-90-group3892.Contig1 | | | | | | | | | | | | | |  |  |  |  |
| M | 0007409 | axonogenesis | EN-124k-90-group3911.Contig2 | | | | | | | | | | | | | |  |  |  |  |
| M | 0007409 | axonogenesis | EN-124k-90-group3916.Contig1 | | | | | | | | | | | | | |  |  |  |  |
| M | 0007409 | axonogenesis | EN-124k-90-group3933.Contig1 | | | | | | | | | | | | | |  |  |  |  |
| M | 0007409 | axonogenesis | EN-124k-90-group3947.Contig1 | | | | | | | | | | | | | |  |  |  |  |
| M | 0007409 | axonogenesis | EN-124k-90-group3947.Contig2 | | | | | | | | | | | | | |  |  |  |  |
| M | 0007409 | axonogenesis | EN-124k-90-group3966.Contig1 | | | | | | | | | | | | | |  |  |  |  |
| M | 0007409 | axonogenesis | EN-124k-90-group3966.Contig2 | | | | | | | | | | | | | |  |  |  |  |
| M | 0007409 | axonogenesis | EN-124k-90-group3991.Contig1 | | | | | | | | | | | | | |  |  |  |  |
| M | 0007409 | axonogenesis | EN-124k-90-group3997.Contig2 | | | | | | | | | | | | | |  |  |  |  |
| M | 0007409 | axonogenesis | EN-124k-90-group4009.Contig1 | | | | | | | | | | | | | |  |  |  |  |
| E | 0007409 | axonogenesis | EN-124k-90-group4039.Contig1 | | | | | | | | | | | | | |  |  |  |  |
| M | 0007409 | axonogenesis | EN-124k-90-group4045.Contig1 | | | | | | | | | | | | | |  |  |  |  |
| M | 0007409 | axonogenesis | EN-124k-90-group4046.Contig1 | | | | | | | | | | | | | |  |  |  |  |
| M | 0007409 | axonogenesis | EN-124k-90-group4049.Contig1 | | | | | | | | | | | | | |  |  |  |  |
| M | 0007409 | axonogenesis | EN-124k-90-group4086.Contig1 | | | | | | | | | | | | | |  |  |  |  |
| M | 0007409 | axonogenesis | EN-124k-90-group4090.Contig1 | | | | | | | | | | | | | |  |  |  |  |
| M | 0007409 | axonogenesis | EN-124k-90-group4106.Contig1 | | | | | | | | | | | | | |  |  |  |  |
| M | 0007409 | axonogenesis | EN-124k-90-group4115.Contig2 | | | | | | | | | | | | | |  |  |  |  |
| M | 0007409 | axonogenesis | EN-124k-90-group4128.Contig1 | | | | | | | | | | | | | |  |  |  |  |
| M | 0007409 | axonogenesis | EN-124k-90-group4154.Contig1 | | | | | | | | | | | | | |  |  |  |  |
| M | 0007409 | axonogenesis | EN-124k-90-group4168.Contig1 | | | | | | | | | | | | | |  |  |  |  |
| M | 0007409 | axonogenesis | EN-124k-90-group4168.Contig2 | | | | | | | | | | | | | |  |  |  |  |
| M | 0007409 | axonogenesis | EN-124k-90-group4168.Contig3 | | | | | | | | | | | | | |  |  |  |  |
| M | 0007409 | axonogenesis | EN-124k-90-group4183.Contig1 | | | | | | | | | | | | | |  |  |  |  |
| M | 0007409 | axonogenesis | EN-124k-90-group4216.Contig1 | | | | | | | | | | | | | |  |  |  |  |
| M | 0007409 | axonogenesis | EN-124k-90-group4225.Contig2 | | | | | | | | | | | | | |  |  |  |  |
| M | 0007409 | axonogenesis | EN-124k-90-group4290.Contig3 | | | | | | | | | | | | | |  |  |  |  |
| M | 0007409 | axonogenesis | EN-124k-90-group4339.Contig1 | | | | | | | | | | | | | |  |  |  |  |
| M | 0007409 | axonogenesis | EN-124k-90-group4349.Contig1 | | | | | | | | | | | | | |  |  |  |  |
| M | 0007409 | axonogenesis | EN-124k-90-group4349.Contig3 | | | | | | | | | | | | | |  |  |  |  |
| M | 0007409 | axonogenesis | EN-124k-90-group4349.Contig4 | | | | | | | | | | | | | |  |  |  |  |
| M | 0007409 | axonogenesis | EN-124k-90-group4350.Contig2 | | | | | | | | | | | | | |  |  |  |  |
| M | 0007409 | axonogenesis | EN-124k-90-group4372.Contig1 | | | | | | | | | | | | | |  |  |  |  |
| M | 0007409 | axonogenesis | EN-124k-90-group4410.Contig1 | | | | | | | | | | | | | |  |  |  |  |
| M | 0007409 | axonogenesis | EN-124k-90-group4432.Contig4 | | | | | | | | | | | | | |  |  |  |  |
| M | 0007409 | axonogenesis | EN-124k-90-group4440.Contig1 | | | | | | | | | | | | | |  |  |  |  |
| M | 0007409 | axonogenesis | EN-124k-90-group4440.Contig2 | | | | | | | | | | | | | |  |  |  |  |
| M | 0007409 | axonogenesis | EN-124k-90-group4440.Contig3 | | | | | | | | | | | | | |  |  |  |  |
| M | 0007409 | axonogenesis | EN-124k-90-group4440.Contig4 | | | | | | | | | | | | | |  |  |  |  |
| M | 0007409 | axonogenesis | EN-124k-90-group4529.Contig1 | | | | | | | | | | | | | |  |  |  |  |
| M | 0007409 | axonogenesis | EN-124k-90-group4558.Contig2 | | | | | | | | | | | | | |  |  |  |  |
| M | 0007409 | axonogenesis | EN-124k-90-group4560.Contig1 | | | | | | | | | | | | | |  |  |  |  |
| M | 0007409 | axonogenesis | EN-124k-90-group4562.Contig1 | | | | | | | | | | | | | |  |  |  |  |
| M | 0007409 | axonogenesis | EN-124k-90-group4599.Contig1 | | | | | | | | | | | | | |  |  |  |  |
| M | 0007409 | axonogenesis | EN-124k-90-group4600.Contig1 | | | | | | | | | | | | | |  |  |  |  |
| M | 0007409 | axonogenesis | EN-124k-90-group4616.Contig1 | | | | | | | | | | | | | |  |  |  |  |
| M | 0007409 | axonogenesis | EN-124k-90-group4621.Contig2 | | | | | | | | | | | | | |  |  |  |  |
| M | 0007409 | axonogenesis | EN-124k-90-group4668.Contig1 | | | | | | | | | | | | | |  |  |  |  |
| M | 0007409 | axonogenesis | EN-124k-90-group4701.Contig1 | | | | | | | | | | | | | |  |  |  |  |
| M | 0007409 | axonogenesis | EN-124k-90-group4701.Contig2 | | | | | | | | | | | | | |  |  |  |  |
| M | 0007409 | axonogenesis | EN-124k-90-group4701.Contig3 | | | | | | | | | | | | | |  |  |  |  |
| M | 0007409 | axonogenesis | EN-124k-90-group4716.Contig1 | | | | | | | | | | | | | |  |  |  |  |
| M | 0007409 | axonogenesis | EN-124k-90-group4753.Contig1 | | | | | | | | | | | | | |  |  |  |  |
| M | 0007409 | axonogenesis | EN-124k-90-group4759.Contig2 | | | | | | | | | | | | | |  |  |  |  |
| M | 0007409 | axonogenesis | EN-124k-90-group4759.Contig3 | | | | | | | | | | | | | |  |  |  |  |
| M | 0007409 | axonogenesis | EN-124k-90-group4763.Contig1 | | | | | | | | | | | | | |  |  |  |  |
| M | 0007409 | axonogenesis | EN-124k-90-group4781.Contig1 | | | | | | | | | | | | | |  |  |  |  |
| M | 0007409 | axonogenesis | EN-124k-90-group4781.Contig2 | | | | | | | | | | | | | |  |  |  |  |
| M | 0007409 | axonogenesis | EN-124k-90-group4784.Contig1 | | | | | | | | | | | | | |  |  |  |  |
| M | 0007409 | axonogenesis | EN-124k-90-group4788.Contig1 | | | | | | | | | | | | | |  |  |  |  |
| M | 0007409 | axonogenesis | EN-124k-90-group4793.Contig2 | | | | | | | | | | | | | |  |  |  |  |
| M | 0007409 | axonogenesis | EN-124k-90-group4803.Contig1 | | | | | | | | | | | | | |  |  |  |  |
| M | 0007409 | axonogenesis | EN-124k-90-group4803.Contig2 | | | | | | | | | | | | | |  |  |  |  |
| M | 0007409 | axonogenesis | EN-124k-90-group4808.Contig1 | | | | | | | | | | | | | |  |  |  |  |
| M | 0007409 | axonogenesis | EN-124k-90-group4808.Contig2 | | | | | | | | | | | | | |  |  |  |  |
| M | 0007409 | axonogenesis | EN-124k-90-group4824.Contig3 | | | | | | | | | | | | | |  |  |  |  |
| M | 0007409 | axonogenesis | EN-124k-90-group4824.Contig4 | | | | | | | | | | | | | |  |  |  |  |
| M | 0007409 | axonogenesis | EN-124k-90-group4827.Contig2 | | | | | | | | | | | | | |  |  |  |  |
| M | 0007409 | axonogenesis | EN-124k-90-group4827.Contig3 | | | | | | | | | | | | | |  |  |  |  |
| M | 0007409 | axonogenesis | EN-124k-90-group4829.Contig3 | | | | | | | | | | | | | |  |  |  |  |
| E | 0007409 | axonogenesis | EN-124k-90-group4884.Contig1 | | | | | | | | | | | | | |  |  |  |  |
| M | 0007409 | axonogenesis | EN-124k-90-group4890.Contig1 | | | | | | | | | | | | | |  |  |  |  |
| M | 0007409 | axonogenesis | EN-124k-90-group4929.Contig1 | | | | | | | | | | | | | |  |  |  |  |
| M | 0007409 | axonogenesis | EN-124k-90-group4929.Contig2 | | | | | | | | | | | | | |  |  |  |  |
| A | 0007409 | axonogenesis | EN-124k-90-group4960.Contig1 | | | | | | | | | | | | | |  |  |  |  |
| M | 0007409 | axonogenesis | EN-124k-90-group4989.Contig2 | | | | | | | | | | | | | |  |  |  |  |
| M | 0007409 | axonogenesis | EN-124k-90-group5022.Contig2 | | | | | | | | | | | | | |  |  |  |  |
| M | 0007409 | axonogenesis | EN-124k-90-group5036.Contig2 | | | | | | | | | | | | | |  |  |  |  |
| M | 0007409 | axonogenesis | EN-124k-90-group5040.Contig1 | | | | | | | | | | | | | |  |  |  |  |
| M | 0007409 | axonogenesis | EN-124k-90-group5040.Contig2 | | | | | | | | | | | | | |  |  |  |  |
| M | 0007409 | axonogenesis | EN-124k-90-group5051.Contig2 | | | | | | | | | | | | | |  |  |  |  |
| M | 0007409 | axonogenesis | EN-124k-90-group5054.Contig1 | | | | | | | | | | | | | |  |  |  |  |
| A | 0007409 | axonogenesis | EN-124k-90-group5054.Contig2 | | | | | | | | | | | | | |  |  |  |  |
| M | 0007409 | axonogenesis | EN-124k-90-group5054.Contig3 | | | | | | | | | | | | | |  |  |  |  |
| M | 0007409 | axonogenesis | EN-124k-90-group5054.Contig4 | | | | | | | | | | | | | |  |  |  |  |
| M | 0007409 | axonogenesis | EN-124k-90-group5055.Contig1 | | | | | | | | | | | | | |  |  |  |  |
| M | 0007409 | axonogenesis | EN-124k-90-group5055.Contig3 | | | | | | | | | | | | | |  |  |  |  |
| M | 0007409 | axonogenesis | EN-124k-90-group5063.Contig1 | | | | | | | | | | | | | |  |  |  |  |
| M | 0007409 | axonogenesis | EN-124k-90-group5071.Contig3 | | | | | | | | | | | | | |  |  |  |  |
| M | 0007409 | axonogenesis | EN-124k-90-group5093.Contig2 | | | | | | | | | | | | | |  |  |  |  |
| M | 0007409 | axonogenesis | EN-124k-90-group5134.Contig1 | | | | | | | | | | | | | |  |  |  |  |
| M | 0007409 | axonogenesis | EN-124k-90-group5148.Contig1 | | | | | | | | | | | | | |  |  |  |  |
| M | 0007409 | axonogenesis | EN-124k-90-group5148.Contig3 | | | | | | | | | | | | | |  |  |  |  |
| M | 0007409 | axonogenesis | EN-124k-90-group5170.Contig1 | | | | | | | | | | | | | |  |  |  |  |
| M | 0007409 | axonogenesis | EN-124k-90-group5170.Contig2 | | | | | | | | | | | | | |  |  |  |  |
| M | 0007409 | axonogenesis | EN-124k-90-group5170.Contig3 | | | | | | | | | | | | | |  |  |  |  |
| M | 0007409 | axonogenesis | EN-124k-90-group5172.Contig1 | | | | | | | | | | | | | |  |  |  |  |
| M | 0007409 | axonogenesis | EN-124k-90-group5173.Contig1 | | | | | | | | | | | | | |  |  |  |  |
| M | 0007409 | axonogenesis | EN-124k-90-group5184.Contig1 | | | | | | | | | | | | | |  |  |  |  |
| M | 0007409 | axonogenesis | EN-124k-90-group5215.Contig3 | | | | | | | | | | | | | |  |  |  |  |
| M | 0007409 | axonogenesis | EN-124k-90-group5252.Contig1 | | | | | | | | | | | | | |  |  |  |  |
| M | 0007409 | axonogenesis | EN-124k-90-group5273.Contig2 | | | | | | | | | | | | | |  |  |  |  |
| M | 0007409 | axonogenesis | EN-124k-90-group5273.Contig3 | | | | | | | | | | | | | |  |  |  |  |
| M | 0007409 | axonogenesis | EN-124k-90-group5282.Contig1 | | | | | | | | | | | | | |  |  |  |  |
| M | 0007409 | axonogenesis | EN-124k-90-group5293.Contig1 | | | | | | | | | | | | | |  |  |  |  |
| M | 0007409 | axonogenesis | EN-124k-90-group5298.Contig1 | | | | | | | | | | | | | |  |  |  |  |
| M | 0007409 | axonogenesis | EN-124k-90-group5300.Contig5 | | | | | | | | | | | | | |  |  |  |  |
| M | 0007409 | axonogenesis | EN-124k-90-group5329.Contig1 | | | | | | | | | | | | | |  |  |  |  |
| M | 0007409 | axonogenesis | EN-124k-90-group5341.Contig4 | | | | | | | | | | | | | |  |  |  |  |
| M | 0007409 | axonogenesis | EN-124k-90-group5359.Contig3 | | | | | | | | | | | | | |  |  |  |  |
| M | 0007409 | axonogenesis | EN-124k-90-group5365.Contig1 | | | | | | | | | | | | | |  |  |  |  |
| M | 0007409 | axonogenesis | EN-124k-90-group5380.Contig1 | | | | | | | | | | | | | |  |  |  |  |
| M | 0007409 | axonogenesis | EN-124k-90-group5383.Contig1 | | | | | | | | | | | | | |  |  |  |  |
| M | 0007409 | axonogenesis | EN-124k-90-group5398.Contig1 | | | | | | | | | | | | | |  |  |  |  |
| M | 0007409 | axonogenesis | EN-124k-90-group5411.Contig1 | | | | | | | | | | | | | |  |  |  |  |
| M | 0007409 | axonogenesis | EN-124k-90-group5459.Contig2 | | | | | | | | | | | | | |  |  |  |  |
| M | 0007409 | axonogenesis | EN-124k-90-group5471.Contig1 | | | | | | | | | | | | | |  |  |  |  |
| M | 0007409 | axonogenesis | EN-124k-90-group5471.Contig3 | | | | | | | | | | | | | |  |  |  |  |
| M | 0007409 | axonogenesis | EN-124k-90-group5479.Contig1 | | | | | | | | | | | | | |  |  |  |  |
| M | 0007409 | axonogenesis | EN-124k-90-group5491.Contig1 | | | | | | | | | | | | | |  |  |  |  |
| M | 0007409 | axonogenesis | EN-124k-90-group5491.Contig2 | | | | | | | | | | | | | |  |  |  |  |
| M | 0007409 | axonogenesis | EN-124k-90-group5527.Contig2 | | | | | | | | | | | | | |  |  |  |  |
| M | 0007409 | axonogenesis | EN-124k-90-group5541.Contig1 | | | | | | | | | | | | | |  |  |  |  |
| M | 0007409 | axonogenesis | EN-124k-90-group5542.Contig1 | | | | | | | | | | | | | |  |  |  |  |
| A | 0007409 | axonogenesis | EN-124k-90-group5543.Contig1 | | | | | | | | | | | | | |  |  |  |  |
| M | 0007409 | axonogenesis | EN-124k-90-group5563.Contig1 | | | | | | | | | | | | | |  |  |  |  |
| M | 0007409 | axonogenesis | EN-124k-90-group5567.Contig2 | | | | | | | | | | | | | |  |  |  |  |
| M | 0007409 | axonogenesis | EN-124k-90-group5567.Contig3 | | | | | | | | | | | | | |  |  |  |  |
| M | 0007409 | axonogenesis | EN-124k-90-group5597.Contig2 | | | | | | | | | | | | | |  |  |  |  |
| M | 0007409 | axonogenesis | EN-124k-90-group5607.Contig1 | | | | | | | | | | | | | |  |  |  |  |
| M | 0007409 | axonogenesis | EN-124k-90-group5616.Contig1 | | | | | | | | | | | | | |  |  |  |  |
| M | 0007409 | axonogenesis | EN-124k-90-group5633.Contig1 | | | | | | | | | | | | | |  |  |  |  |
| M | 0007409 | axonogenesis | EN-124k-90-group5633.Contig2 | | | | | | | | | | | | | |  |  |  |  |
| M | 0007409 | axonogenesis | EN-124k-90-group5709.Contig1 | | | | | | | | | | | | | |  |  |  |  |
| M | 0007409 | axonogenesis | EN-124k-90-group5710.Contig2 | | | | | | | | | | | | | |  |  |  |  |
| M | 0007409 | axonogenesis | EN-124k-90-group5717.Contig2 | | | | | | | | | | | | | |  |  |  |  |
| M | 0007409 | axonogenesis | EN-124k-90-group5729.Contig1 | | | | | | | | | | | | | |  |  |  |  |
| M | 0007409 | axonogenesis | EN-124k-90-group5729.Contig2 | | | | | | | | | | | | | |  |  |  |  |
| M | 0007409 | axonogenesis | EN-124k-90-group5746.Contig1 | | | | | | | | | | | | | |  |  |  |  |
| M | 0007409 | axonogenesis | EN-124k-90-group5910.Contig1 | | | | | | | | | | | | | |  |  |  |  |
| M | 0007409 | axonogenesis | EN-124k-90-group5967.Contig1 | | | | | | | | | | | | | |  |  |  |  |
| M | 0007409 | axonogenesis | EN-124k-90-group5968.Contig1 | | | | | | | | | | | | | |  |  |  |  |
| M | 0007409 | axonogenesis | EN-124k-90-group5996.Contig1 | | | | | | | | | | | | | |  |  |  |  |
| M | 0007409 | axonogenesis | EN-124k-90-group5996.Contig2 | | | | | | | | | | | | | |  |  |  |  |
| M | 0007409 | axonogenesis | EN-124k-90-group6033.Contig1 | | | | | | | | | | | | | |  |  |  |  |
| M | 0007409 | axonogenesis | EN-124k-90-group6033.Contig2 | | | | | | | | | | | | | |  |  |  |  |
| M | 0007409 | axonogenesis | EN-124k-90-group6033.Contig3 | | | | | | | | | | | | | |  |  |  |  |
| M | 0007409 | axonogenesis | EN-124k-90-group6039.Contig2 | | | | | | | | | | | | | |  |  |  |  |
| M | 0007409 | axonogenesis | EN-124k-90-group6039.Contig3 | | | | | | | | | | | | | |  |  |  |  |
| M | 0007409 | axonogenesis | EN-124k-90-group6045.Contig1 | | | | | | | | | | | | | |  |  |  |  |
| M | 0007409 | axonogenesis | EN-124k-90-group6109.Contig1 | | | | | | | | | | | | | |  |  |  |  |
| M | 0007409 | axonogenesis | EN-124k-90-group6112.Contig1 | | | | | | | | | | | | | |  |  |  |  |
| M | 0007409 | axonogenesis | EN-124k-90-group6157.Contig1 | | | | | | | | | | | | | |  |  |  |  |
| M | 0007409 | axonogenesis | EN-124k-90-group6172.Contig1 | | | | | | | | | | | | | |  |  |  |  |
| M | 0007409 | axonogenesis | EN-124k-90-group6172.Contig2 | | | | | | | | | | | | | |  |  |  |  |
| M | 0007409 | axonogenesis | EN-124k-90-group6172.Contig3 | | | | | | | | | | | | | |  |  |  |  |
| M | 0007409 | axonogenesis | EN-124k-90-group6183.Contig1 | | | | | | | | | | | | | |  |  |  |  |
| M | 0007409 | axonogenesis | EN-124k-90-group6195.Contig1 | | | | | | | | | | | | | |  |  |  |  |
| M | 0007409 | axonogenesis | EN-124k-90-group6195.Contig2 | | | | | | | | | | | | | |  |  |  |  |
| M | 0007409 | axonogenesis | EN-124k-90-group6203.Contig1 | | | | | | | | | | | | | |  |  |  |  |
| M | 0007409 | axonogenesis | EN-124k-90-group6204.Contig1 | | | | | | | | | | | | | |  |  |  |  |
| M | 0007409 | axonogenesis | EN-124k-90-group6204.Contig2 | | | | | | | | | | | | | |  |  |  |  |
| E | 0007409 | axonogenesis | EN-124k-90-group6238.Contig1 | | | | | | | | | | | | | |  |  |  |  |
| M | 0007409 | axonogenesis | EN-124k-90-group6259.Contig1 | | | | | | | | | | | | | |  |  |  |  |
| M | 0007409 | axonogenesis | EN-124k-90-group6259.Contig2 | | | | | | | | | | | | | |  |  |  |  |
| M | 0007409 | axonogenesis | EN-124k-90-group6291.Contig2 | | | | | | | | | | | | | |  |  |  |  |
| E | 0007409 | axonogenesis | EN-124k-90-group6326.Contig1 | | | | | | | | | | | | | |  |  |  |  |
| M | 0007409 | axonogenesis | EN-124k-90-group6358.Contig2 | | | | | | | | | | | | | |  |  |  |  |
| M | 0007409 | axonogenesis | EN-124k-90-group6360.Contig1 | | | | | | | | | | | | | |  |  |  |  |
| M | 0007409 | axonogenesis | EN-124k-90-group6400.Contig1 | | | | | | | | | | | | | |  |  |  |  |
| M | 0007409 | axonogenesis | EN-124k-90-group6457.Contig1 | | | | | | | | | | | | | |  |  |  |  |
| M | 0007409 | axonogenesis | EN-124k-90-group6591.Contig1 | | | | | | | | | | | | | |  |  |  |  |
| M | 0007409 | axonogenesis | EN-124k-90-group6614.Contig1 | | | | | | | | | | | | | |  |  |  |  |
| M | 0007409 | axonogenesis | EN-124k-90-group6620.Contig1 | | | | | | | | | | | | | |  |  |  |  |
| M | 0007409 | axonogenesis | EN-124k-90-group6644.Contig1 | | | | | | | | | | | | | |  |  |  |  |
| M | 0007409 | axonogenesis | EN-124k-90-group6647.Contig1 | | | | | | | | | | | | | |  |  |  |  |
| M | 0007409 | axonogenesis | EN-124k-90-group6649.Contig2 | | | | | | | | | | | | | |  |  |  |  |
| M | 0007409 | axonogenesis | EN-124k-90-group6649.Contig3 | | | | | | | | | | | | | |  |  |  |  |
| M | 0007409 | axonogenesis | EN-124k-90-group6652.Contig1 | | | | | | | | | | | | | |  |  |  |  |
| M | 0007409 | axonogenesis | EN-124k-90-group6666.Contig1 | | | | | | | | | | | | | |  |  |  |  |
| M | 0007409 | axonogenesis | EN-124k-90-group6667.Contig1 | | | | | | | | | | | | | |  |  |  |  |
| M | 0007409 | axonogenesis | EN-124k-90-group6680.Contig1 | | | | | | | | | | | | | |  |  |  |  |
| M | 0007409 | axonogenesis | EN-124k-90-group6687.Contig2 | | | | | | | | | | | | | |  |  |  |  |
| M | 0007409 | axonogenesis | EN-124k-90-group6694.Contig1 | | | | | | | | | | | | | |  |  |  |  |
| E | 0007409 | axonogenesis | EN-124k-90-group6714.Contig1 | | | | | | | | | | | | | |  |  |  |  |
| M | 0007409 | axonogenesis | EN-124k-90-group6719.Contig2 | | | | | | | | | | | | | |  |  |  |  |
| M | 0007409 | axonogenesis | EN-124k-90-group6742.Contig2 | | | | | | | | | | | | | |  |  |  |  |
| M | 0007409 | axonogenesis | EN-124k-90-group6809.Contig1 | | | | | | | | | | | | | |  |  |  |  |
| M | 0007409 | axonogenesis | EN-124k-90-group6883.Contig2 | | | | | | | | | | | | | |  |  |  |  |
| M | 0007409 | axonogenesis | EN-124k-90-group6895.Contig1 | | | | | | | | | | | | | |  |  |  |  |
| M | 0007409 | axonogenesis | EN-124k-90-group6904.Contig1 | | | | | | | | | | | | | |  |  |  |  |
| M | 0007409 | axonogenesis | EN-124k-90-group6923.Contig1 | | | | | | | | | | | | | |  |  |  |  |
| M | 0007409 | axonogenesis | EN-124k-90-group6923.Contig2 | | | | | | | | | | | | | |  |  |  |  |
| A | 0007409 | axonogenesis | EN-124k-90-group6966.Contig1 | | | | | | | | | | | | | |  |  |  |  |
| M | 0007409 | axonogenesis | EN-124k-90-group6983.Contig2 | | | | | | | | | | | | | |  |  |  |  |
| M | 0007409 | axonogenesis | EN-124k-90-group7017.Contig1 | | | | | | | | | | | | | |  |  |  |  |
| M | 0007409 | axonogenesis | EN-124k-90-group7018.Contig1 | | | | | | | | | | | | | |  |  |  |  |
| M | 0007409 | axonogenesis | EN-124k-90-group7046.Contig1 | | | | | | | | | | | | | |  |  |  |  |
| M | 0007409 | axonogenesis | EN-124k-90-group7065.Contig2 | | | | | | | | | | | | | |  |  |  |  |
| M | 0007409 | axonogenesis | EN-124k-90-group7102.Contig1 | | | | | | | | | | | | | |  |  |  |  |
| M | 0007409 | axonogenesis | EN-124k-90-group7107.Contig2 | | | | | | | | | | | | | |  |  |  |  |
| M | 0007409 | axonogenesis | EN-124k-90-group7145.Contig1 | | | | | | | | | | | | | |  |  |  |  |
| M | 0007409 | axonogenesis | EN-124k-90-group7149.Contig2 | | | | | | | | | | | | | |  |  |  |  |
| M | 0007409 | axonogenesis | EN-124k-90-group7157.Contig1 | | | | | | | | | | | | | |  |  |  |  |
| M | 0007409 | axonogenesis | EN-124k-90-group7157.Contig2 | | | | | | | | | | | | | |  |  |  |  |
| M | 0007409 | axonogenesis | EN-124k-90-group7165.Contig1 | | | | | | | | | | | | | |  |  |  |  |
| M | 0007409 | axonogenesis | EN-124k-90-group7214.Contig1 | | | | | | | | | | | | | |  |  |  |  |
| M | 0007409 | axonogenesis | EN-124k-90-group7214.Contig2 | | | | | | | | | | | | | |  |  |  |  |
| M | 0007409 | axonogenesis | EN-124k-90-group7231.Contig1 | | | | | | | | | | | | | |  |  |  |  |
| M | 0007409 | axonogenesis | EN-124k-90-group7234.Contig1 | | | | | | | | | | | | | |  |  |  |  |
| M | 0007409 | axonogenesis | EN-124k-90-group7280.Contig1 | | | | | | | | | | | | | |  |  |  |  |
| M | 0007409 | axonogenesis | EN-124k-90-group7281.Contig1 | | | | | | | | | | | | | |  |  |  |  |
| M | 0007409 | axonogenesis | EN-124k-90-group7281.Contig2 | | | | | | | | | | | | | |  |  |  |  |
| M | 0007409 | axonogenesis | EN-124k-90-group7332.Contig1 | | | | | | | | | | | | | |  |  |  |  |
| M | 0007409 | axonogenesis | EN-124k-90-group7343.Contig1 | | | | | | | | | | | | | |  |  |  |  |
| M | 0007409 | axonogenesis | EN-124k-90-group7382.Contig1 | | | | | | | | | | | | | |  |  |  |  |
| M | 0007409 | axonogenesis | EN-124k-90-group7382.Contig2 | | | | | | | | | | | | | |  |  |  |  |
| M | 0007409 | axonogenesis | EN-124k-90-group7440.Contig1 | | | | | | | | | | | | | |  |  |  |  |
| M | 0007409 | axonogenesis | EN-124k-90-group7457.Contig1 | | | | | | | | | | | | | |  |  |  |  |
| M | 0007409 | axonogenesis | EN-124k-90-group7489.Contig1 | | | | | | | | | | | | | |  |  |  |  |
| M | 0007409 | axonogenesis | EN-124k-90-group7514.Contig1 | | | | | | | | | | | | | |  |  |  |  |
| M | 0007409 | axonogenesis | EN-124k-90-group7528.Contig1 | | | | | | | | | | | | | |  |  |  |  |
| M | 0007409 | axonogenesis | EN-124k-90-group7579.Contig1 | | | | | | | | | | | | | |  |  |  |  |
| M | 0007409 | axonogenesis | EN-124k-90-group7612.Contig2 | | | | | | | | | | | | | |  |  |  |  |
| A | 0007409 | axonogenesis | EN-124k-90-group7650.Contig1 | | | | | | | | | | | | | |  |  |  |  |
| M | 0007409 | axonogenesis | EN-124k-90-group7660.Contig1 | | | | | | | | | | | | | |  |  |  |  |
| M | 0007409 | axonogenesis | EN-124k-90-group7666.Contig2 | | | | | | | | | | | | | |  |  |  |  |
| M | 0007409 | axonogenesis | EN-124k-90-group7743.Contig1 | | | | | | | | | | | | | |  |  |  |  |
| M | 0007409 | axonogenesis | EN-124k-90-group7749.Contig1 | | | | | | | | | | | | | |  |  |  |  |
| M | 0007409 | axonogenesis | EN-124k-90-group7770.Contig1 | | | | | | | | | | | | | |  |  |  |  |
| M | 0007409 | axonogenesis | EN-124k-90-group7770.Contig2 | | | | | | | | | | | | | |  |  |  |  |
| M | 0007409 | axonogenesis | EN-124k-90-group7770.Contig3 | | | | | | | | | | | | | |  |  |  |  |
| M | 0007409 | axonogenesis | EN-124k-90-group7785.Contig1 | | | | | | | | | | | | | |  |  |  |  |
| M | 0007409 | axonogenesis | EN-124k-90-group7786.Contig1 | | | | | | | | | | | | | |  |  |  |  |
| M | 0007409 | axonogenesis | EN-124k-90-group7859.Contig3 | | | | | | | | | | | | | |  |  |  |  |
| M | 0007409 | axonogenesis | EN-124k-90-group7866.Contig1 | | | | | | | | | | | | | |  |  |  |  |
| M | 0007409 | axonogenesis | EN-124k-90-group7876.Contig1 | | | | | | | | | | | | | |  |  |  |  |
| M | 0007409 | axonogenesis | EN-124k-90-group7883.Contig1 | | | | | | | | | | | | | |  |  |  |  |
| E | 0007409 | axonogenesis | EN-124k-90-group7888.Contig1 | | | | | | | | | | | | | |  |  |  |  |
| M | 0007409 | axonogenesis | EN-124k-90-group7915.Contig1 | | | | | | | | | | | | | |  |  |  |  |
| M | 0007409 | axonogenesis | EN-124k-90-group7948.Contig1 | | | | | | | | | | | | | |  |  |  |  |
| M | 0007409 | axonogenesis | EN-124k-90-group8079.Contig1 | | | | | | | | | | | | | |  |  |  |  |
| M | 0007409 | axonogenesis | EN-124k-90-group8079.Contig2 | | | | | | | | | | | | | |  |  |  |  |
| E | 0007409 | axonogenesis | EN-124k-90-group8101.Contig1 | | | | | | | | | | | | | |  |  |  |  |
| M | 0007409 | axonogenesis | EN-124k-90-group8103.Contig4 | | | | | | | | | | | | | |  |  |  |  |
| M | 0007409 | axonogenesis | EN-124k-90-group8174.Contig1 | | | | | | | | | | | | | |  |  |  |  |
| M | 0007409 | axonogenesis | EN-124k-90-group8174.Contig2 | | | | | | | | | | | | | |  |  |  |  |
| M | 0007409 | axonogenesis | EN-124k-90-group8193.Contig1 | | | | | | | | | | | | | |  |  |  |  |
| M | 0007409 | axonogenesis | EN-124k-90-group8211.Contig1 | | | | | | | | | | | | | |  |  |  |  |
| M | 0007409 | axonogenesis | EN-124k-90-group8221.Contig1 | | | | | | | | | | | | | |  |  |  |  |
| M | 0007409 | axonogenesis | EN-124k-90-group8245.Contig2 | | | | | | | | | | | | | |  |  |  |  |
| M | 0007409 | axonogenesis | EN-124k-90-group8245.Contig3 | | | | | | | | | | | | | |  |  |  |  |
| M | 0007409 | axonogenesis | EN-124k-90-group8253.Contig1 | | | | | | | | | | | | | |  |  |  |  |
| M | 0007409 | axonogenesis | EN-124k-90-group8297.Contig1 | | | | | | | | | | | | | |  |  |  |  |
| M | 0007409 | axonogenesis | EN-124k-90-group8306.Contig1 | | | | | | | | | | | | | |  |  |  |  |
| M | 0007409 | axonogenesis | EN-124k-90-group8306.Contig4 | | | | | | | | | | | | | |  |  |  |  |
| M | 0007409 | axonogenesis | EN-124k-90-group8325.Contig1 | | | | | | | | | | | | | |  |  |  |  |
| M | 0007409 | axonogenesis | EN-124k-90-group8351.Contig2 | | | | | | | | | | | | | |  |  |  |  |
| M | 0007409 | axonogenesis | EN-124k-90-group8375.Contig1 | | | | | | | | | | | | | |  |  |  |  |
| E | 0007409 | axonogenesis | EN-124k-90-group8412.Contig1 | | | | | | | | | | | | | |  |  |  |  |
| M | 0007409 | axonogenesis | EN-124k-90-group8418.Contig1 | | | | | | | | | | | | | |  |  |  |  |
| M | 0007409 | axonogenesis | EN-124k-90-group8434.Contig2 | | | | | | | | | | | | | |  |  |  |  |
| M | 0007409 | axonogenesis | EN-124k-90-group8440.Contig1 | | | | | | | | | | | | | |  |  |  |  |
| M | 0007409 | axonogenesis | EN-124k-90-group8452.Contig1 | | | | | | | | | | | | | |  |  |  |  |
| M | 0007409 | axonogenesis | EN-124k-90-group8484.Contig3 | | | | | | | | | | | | | |  |  |  |  |
| M | 0007409 | axonogenesis | EN-124k-90-group8492.Contig1 | | | | | | | | | | | | | |  |  |  |  |
| E | 0007409 | axonogenesis | EN-124k-90-group8513.Contig1 | | | | | | | | | | | | | |  |  |  |  |
| A | 0007409 | axonogenesis | EN-124k-90-group8519.Contig1 | | | | | | | | | | | | | |  |  |  |  |
| M | 0007409 | axonogenesis | EN-124k-90-group8568.Contig1 | | | | | | | | | | | | | |  |  |  |  |
| M | 0007409 | axonogenesis | EN-124k-90-group8589.Contig2 | | | | | | | | | | | | | |  |  |  |  |
| M | 0007409 | axonogenesis | EN-124k-90-group8645.Contig1 | | | | | | | | | | | | | |  |  |  |  |
| M | 0007409 | axonogenesis | EN-124k-90-group8685.Contig1 | | | | | | | | | | | | | |  |  |  |  |
| M | 0007409 | axonogenesis | EN-124k-90-group8696.Contig1 | | | | | | | | | | | | | |  |  |  |  |
| A | 0007409 | axonogenesis | EN-124k-90-group8725.Contig1 | | | | | | | | | | | | | |  |  |  |  |
| A | 0007409 | axonogenesis | EN-124k-90-group8766.Contig1 | | | | | | | | | | | | | |  |  |  |  |
| M | 0007409 | axonogenesis | EN-124k-90-group8775.Contig1 | | | | | | | | | | | | | |  |  |  |  |
| M | 0007409 | axonogenesis | EN-124k-90-group8796.Contig2 | | | | | | | | | | | | | |  |  |  |  |
| M | 0007409 | axonogenesis | EN-124k-90-group8799.Contig2 | | | | | | | | | | | | | |  |  |  |  |
| M | 0007409 | axonogenesis | EN-124k-90-group8850.Contig1 | | | | | | | | | | | | | |  |  |  |  |
| M | 0007409 | axonogenesis | EN-124k-90-group8897.Contig1 | | | | | | | | | | | | | |  |  |  |  |
| M | 0007409 | axonogenesis | EN-124k-90-group8905.Contig1 | | | | | | | | | | | | | |  |  |  |  |
| M | 0007409 | axonogenesis | EN-124k-90-group8905.Contig2 | | | | | | | | | | | | | |  |  |  |  |
| M | 0007409 | axonogenesis | EN-124k-90-group8908.Contig2 | | | | | | | | | | | | | |  |  |  |  |
| M | 0007409 | axonogenesis | EN-124k-90-group8937.Contig1 | | | | | | | | | | | | | |  |  |  |  |
| M | 0007409 | axonogenesis | EN-124k-90-group8937.Contig2 | | | | | | | | | | | | | |  |  |  |  |
| A | 0007409 | axonogenesis | EN-124k-90-group8957.Contig1 | | | | | | | | | | | | | |  |  |  |  |
| M | 0007409 | axonogenesis | EN-124k-90-group8988.Contig1 | | | | | | | | | | | | | |  |  |  |  |
| A | 0007409 | axonogenesis | EN-124k-90-group9012.Contig1 | | | | | | | | | | | | | |  |  |  |  |
| M | 0007409 | axonogenesis | EN-124k-90-group9079.Contig1 | | | | | | | | | | | | | |  |  |  |  |
| M | 0007409 | axonogenesis | EN-124k-90-group9165.Contig1 | | | | | | | | | | | | | |  |  |  |  |
| M | 0007409 | axonogenesis | EN-124k-90-group9168.Contig1 | | | | | | | | | | | | | |  |  |  |  |
| M | 0007409 | axonogenesis | EN-124k-90-group9238.Contig1 | | | | | | | | | | | | | |  |  |  |  |
| A | 0007409 | axonogenesis | EN-124k-90-group9245.Contig1 | | | | | | | | | | | | | |  |  |  |  |
| M | 0007409 | axonogenesis | EN-124k-90-group9249.Contig1 | | | | | | | | | | | | | |  |  |  |  |
| M | 0007409 | axonogenesis | EN-124k-90-group9278.Contig2 | | | | | | | | | | | | | |  |  |  |  |
| M | 0007409 | axonogenesis | EN-124k-90-group9299.Contig3 | | | | | | | | | | | | | |  |  |  |  |
| M | 0007409 | axonogenesis | EN-124k-90-group9317.Contig1 | | | | | | | | | | | | | |  |  |  |  |
| M | 0007409 | axonogenesis | EN-124k-90-group9341.Contig1 | | | | | | | | | | | | | |  |  |  |  |
| M | 0007409 | axonogenesis | EN-124k-90-group9388.Contig1 | | | | | | | | | | | | | |  |  |  |  |
| M | 0007409 | axonogenesis | EN-124k-90-group9388.Contig2 | | | | | | | | | | | | | |  |  |  |  |
| M | 0007409 | axonogenesis | EN-124k-90-group9420.Contig1 | | | | | | | | | | | | | |  |  |  |  |
| M | 0007409 | axonogenesis | EN-124k-90-group9446.Contig1 | | | | | | | | | | | | | |  |  |  |  |
| M | 0007409 | axonogenesis | EN-124k-90-group9479.Contig1 | | | | | | | | | | | | | |  |  |  |  |
| M | 0007409 | axonogenesis | EN-124k-90-group9487.Contig2 | | | | | | | | | | | | | |  |  |  |  |
| M | 0007409 | axonogenesis | EN-124k-90-group9541.Contig1 | | | | | | | | | | | | | |  |  |  |  |
| M | 0007409 | axonogenesis | EN-124k-90-group9586.Contig1 | | | | | | | | | | | | | |  |  |  |  |
| E | 0007409 | axonogenesis | EN-124k-90-group9586.Contig2 | | | | | | | | | | | | | |  |  |  |  |
| M | 0007409 | axonogenesis | EN-124k-90-group9620.Contig1 | | | | | | | | | | | | | |  |  |  |  |
| M | 0007409 | axonogenesis | EN-124k-90-group9650.Contig1 | | | | | | | | | | | | | |  |  |  |  |
| M | 0007409 | axonogenesis | EN-124k-90-group9692.Contig1 | | | | | | | | | | | | | |  |  |  |  |
| A | 0007409 | axonogenesis | EN-124k-90-group9767.Contig1 | | | | | | | | | | | | | |  |  |  |  |
| M | 0007409 | axonogenesis | EN-124k-90-group9771.Contig1 | | | | | | | | | | | | | |  |  |  |  |
| M | 0007409 | axonogenesis | EN-124k-90-group9771.Contig2 | | | | | | | | | | | | | |  |  |  |  |
| M | 0007409 | axonogenesis | EN-124k-90-group9794.Contig1 | | | | | | | | | | | | | |  |  |  |  |
| M | 0007409 | axonogenesis | EN-124k-90-group9794.Contig2 | | | | | | | | | | | | | |  |  |  |  |
| M | 0007409 | axonogenesis | EN-124k-90-group9819.Contig1 | | | | | | | | | | | | | |  |  |  |  |
| M | 0007409 | axonogenesis | EN-124k-90-group9843.Contig1 | | | | | | | | | | | | | |  |  |  |  |
| M | 0007409 | axonogenesis | EN-124k-90-group9849.Contig1 | | | | | | | | | | | | | |  |  |  |  |
| M | 0007409 | axonogenesis | EN-124k-90-group9953.Contig1 | | | | | | | | | | | | | |  |  |  |  |
| M | 0007409 | axonogenesis | EN-124k-90-group10046.Contig1 | | | | | | | | | | | | | | |  |  |  |
| M | 0007409 | axonogenesis | EN-124k-90-group10053.Contig1 | | | | | | | | | | | | | | |  |  |  |
| M | 0007409 | axonogenesis | EN-124k-90-group10056.Contig1 | | | | | | | | | | | | | | |  |  |  |
| A | 0007409 | axonogenesis | EN-124k-90-group10065.Contig1 | | | | | | | | | | | | | | |  |  |  |
| M | 0007409 | axonogenesis | EN-124k-90-group10085.Contig1 | | | | | | | | | | | | | | |  |  |  |
| M | 0007409 | axonogenesis | EN-124k-90-group10085.Contig2 | | | | | | | | | | | | | | |  |  |  |
| M | 0007409 | axonogenesis | EN-124k-90-group10221.Contig1 | | | | | | | | | | | | | | |  |  |  |
| M | 0007409 | axonogenesis | EN-124k-90-group10290.Contig2 | | | | | | | | | | | | | | |  |  |  |
| A | 0007409 | axonogenesis | EN-124k-90-group10370.Contig1 | | | | | | | | | | | | | | |  |  |  |
| M | 0007409 | axonogenesis | EN-124k-90-group10394.Contig1 | | | | | | | | | | | | | | |  |  |  |
| M | 0007409 | axonogenesis | EN-124k-90-group10450.Contig1 | | | | | | | | | | | | | | |  |  |  |
| M | 0007409 | axonogenesis | EN-124k-90-group10467.Contig1 | | | | | | | | | | | | | | |  |  |  |
| M | 0007409 | axonogenesis | EN-124k-90-group10486.Contig1 | | | | | | | | | | | | | | |  |  |  |
| M | 0007409 | axonogenesis | EN-124k-90-group10528.Contig1 | | | | | | | | | | | | | | |  |  |  |
| E | 0007409 | axonogenesis | EN-124k-90-group10565.Contig1 | | | | | | | | | | | | | | |  |  |  |
| M | 0007409 | axonogenesis | EN-124k-90-group10598.Contig1 | | | | | | | | | | | | | | |  |  |  |
| M | 0007409 | axonogenesis | EN-124k-90-group10630.Contig2 | | | | | | | | | | | | | | |  |  |  |
| M | 0007409 | axonogenesis | EN-124k-90-group10637.Contig1 | | | | | | | | | | | | | | |  |  |  |
| M | 0007409 | axonogenesis | EN-124k-90-group10650.Contig1 | | | | | | | | | | | | | | |  |  |  |
| M | 0007409 | axonogenesis | EN-124k-90-group10652.Contig1 | | | | | | | | | | | | | | |  |  |  |
| M | 0007409 | axonogenesis | EN-124k-90-group10717.Contig1 | | | | | | | | | | | | | | |  |  |  |
| E | 0007409 | axonogenesis | EN-124k-90-group10742.Contig1 | | | | | | | | | | | | | | |  |  |  |
| M | 0007409 | axonogenesis | EN-124k-90-group10750.Contig1 | | | | | | | | | | | | | | |  |  |  |
| M | 0007409 | axonogenesis | EN-124k-90-group10790.Contig1 | | | | | | | | | | | | | | |  |  |  |
| A | 0007409 | axonogenesis | EN-124k-90-group10802.Contig1 | | | | | | | | | | | | | | |  |  |  |
| M | 0007409 | axonogenesis | EN-124k-90-group10823.Contig1 | | | | | | | | | | | | | | |  |  |  |
| M | 0007409 | axonogenesis | EN-124k-90-group10824.Contig1 | | | | | | | | | | | | | | |  |  |  |
| M | 0007409 | axonogenesis | EN-124k-90-group10838.Contig1 | | | | | | | | | | | | | | |  |  |  |
| M | 0007409 | axonogenesis | EN-124k-90-group10838.Contig2 | | | | | | | | | | | | | | |  |  |  |
| M | 0007409 | axonogenesis | EN-124k-90-group10843.Contig1 | | | | | | | | | | | | | | |  |  |  |
| A | 0007409 | axonogenesis | EN-124k-90-group10847.Contig1 | | | | | | | | | | | | | | |  |  |  |
| M | 0007409 | axonogenesis | EN-124k-90-group10875.Contig1 | | | | | | | | | | | | | | |  |  |  |
| M | 0007409 | axonogenesis | EN-124k-90-group10877.Contig1 | | | | | | | | | | | | | | |  |  |  |
| M | 0007409 | axonogenesis | EN-124k-90-group10881.Contig1 | | | | | | | | | | | | | | |  |  |  |
| M | 0007409 | axonogenesis | EN-124k-90-group10907.Contig1 | | | | | | | | | | | | | | |  |  |  |
| M | 0007409 | axonogenesis | EN-124k-90-group10912.Contig1 | | | | | | | | | | | | | | |  |  |  |
| M | 0007409 | axonogenesis | EN-124k-90-group10928.Contig1 | | | | | | | | | | | | | | |  |  |  |
| M | 0007409 | axonogenesis | EN-124k-90-group10950.Contig1 | | | | | | | | | | | | | | |  |  |  |
| E | 0007409 | axonogenesis | EN-124k-90-group10984.Contig2 | | | | | | | | | | | | | | |  |  |  |
| M | 0007409 | axonogenesis | EN-124k-90-group10990.Contig1 | | | | | | | | | | | | | | |  |  |  |
| M | 0007409 | axonogenesis | EN-124k-90-group11082.Contig1 | | | | | | | | | | | | | | |  |  |  |
| M | 0007409 | axonogenesis | EN-124k-90-group11100.Contig1 | | | | | | | | | | | | | | |  |  |  |
| M | 0007409 | axonogenesis | EN-124k-90-group11223.Contig1 | | | | | | | | | | | | | | |  |  |  |
| M | 0007409 | axonogenesis | EN-124k-90-group11228.Contig1 | | | | | | | | | | | | | | |  |  |  |
| M | 0007409 | axonogenesis | EN-124k-90-group11235.Contig1 | | | | | | | | | | | | | | |  |  |  |
| M | 0007409 | axonogenesis | EN-124k-90-group11250.Contig1 | | | | | | | | | | | | | | |  |  |  |
| M | 0007409 | axonogenesis | EN-124k-90-group11317.Contig1 | | | | | | | | | | | | | | |  |  |  |
| M | 0007409 | axonogenesis | EN-124k-90-group11317.Contig2 | | | | | | | | | | | | | | |  |  |  |
| M | 0007409 | axonogenesis | EN-124k-90-group11399.Contig2 | | | | | | | | | | | | | | |  |  |  |
| M | 0007409 | axonogenesis | EN-124k-90-group11408.Contig1 | | | | | | | | | | | | | | |  |  |  |
| M | 0007409 | axonogenesis | EN-124k-90-group11413.Contig1 | | | | | | | | | | | | | | |  |  |  |
| M | 0007409 | axonogenesis | EN-124k-90-group11508.Contig1 | | | | | | | | | | | | | | |  |  |  |
| M | 0007409 | axonogenesis | EN-124k-90-group11519.Contig1 | | | | | | | | | | | | | | |  |  |  |
| M | 0007409 | axonogenesis | EN-124k-90-group11686.Contig1 | | | | | | | | | | | | | | |  |  |  |
| M | 0007409 | axonogenesis | EN-124k-90-group11723.Contig1 | | | | | | | | | | | | | | |  |  |  |
| M | 0007409 | axonogenesis | EN-124k-90-group11737.Contig1 | | | | | | | | | | | | | | |  |  |  |
| M | 0007409 | axonogenesis | EN-124k-90-group11808.Contig1 | | | | | | | | | | | | | | |  |  |  |
| M | 0007409 | axonogenesis | EN-124k-90-group12003.Contig1 | | | | | | | | | | | | | | |  |  |  |
| M | 0007409 | axonogenesis | EN-124k-90-group12008.Contig1 | | | | | | | | | | | | | | |  |  |  |
| M | 0007409 | axonogenesis | EN-124k-90-group12099.Contig1 | | | | | | | | | | | | | | |  |  |  |
| M | 0007409 | axonogenesis | EN-124k-90-group12337.Contig1 | | | | | | | | | | | | | | |  |  |  |
| M | 0007409 | axonogenesis | EN-124k-90-group12337.Contig2 | | | | | | | | | | | | | | |  |  |  |
| M | 0007409 | axonogenesis | EN-124k-90-group12470.Contig1 | | | | | | | | | | | | | | |  |  |  |
| M | 0007409 | axonogenesis | EN-124k-90-group12519.Contig1 | | | | | | | | | | | | | | |  |  |  |
| E | 0007409 | axonogenesis | EN-124k-90-group12524.Contig1 | | | | | | | | | | | | | | |  |  |  |
| M | 0007409 | axonogenesis | EN-124k-90-group12530.Contig1 | | | | | | | | | | | | | | |  |  |  |
| M | 0007409 | axonogenesis | EN-124k-90-group12530.Contig2 | | | | | | | | | | | | | | |  |  |  |
| E | 0007409 | axonogenesis | EN-124k-90-group12535.Contig1 | | | | | | | | | | | | | | |  |  |  |
| M | 0007409 | axonogenesis | EN-124k-90-group12594.Contig1 | | | | | | | | | | | | | | |  |  |  |
| A | 0007409 | axonogenesis | EN-124k-90-group12616.Contig1 | | | | | | | | | | | | | | |  |  |  |
| M | 0007409 | axonogenesis | EN-124k-90-group12638.Contig1 | | | | | | | | | | | | | | |  |  |  |
| E | 0007409 | axonogenesis | EN-124k-90-group12653.Contig1 | | | | | | | | | | | | | | |  |  |  |
| M | 0007409 | axonogenesis | EN-124k-90-group12654.Contig1 | | | | | | | | | | | | | | |  |  |  |
| M | 0007409 | axonogenesis | EN-124k-90-group12751.Contig1 | | | | | | | | | | | | | | |  |  |  |
| M | 0007409 | axonogenesis | EN-124k-90-group12751.Contig2 | | | | | | | | | | | | | | |  |  |  |
| M | 0007409 | axonogenesis | EN-124k-90-group12751.Contig3 | | | | | | | | | | | | | | |  |  |  |
| M | 0007409 | axonogenesis | EN-124k-90-group12755.Contig1 | | | | | | | | | | | | | | |  |  |  |
| A | 0007409 | axonogenesis | EN-124k-90-group12774.Contig1 | | | | | | | | | | | | | | |  |  |  |
| A | 0007409 | axonogenesis | EN-124k-90-group12832.Contig1 | | | | | | | | | | | | | | |  |  |  |
| M | 0007409 | axonogenesis | EN-124k-90-group12836.Contig1 | | | | | | | | | | | | | | |  |  |  |
| M | 0007409 | axonogenesis | EN-124k-90-group12881.Contig1 | | | | | | | | | | | | | | |  |  |  |
| M | 0007409 | axonogenesis | EN-124k-90-group12960.Contig1 | | | | | | | | | | | | | | |  |  |  |
| M | 0007409 | axonogenesis | EN-124k-90-group12973.Contig1 | | | | | | | | | | | | | | |  |  |  |
| M | 0007409 | axonogenesis | EN-124k-90-group13025.Contig1 | | | | | | | | | | | | | | |  |  |  |
| M | 0007409 | axonogenesis | EN-124k-90-group13095.Contig1 | | | | | | | | | | | | | | |  |  |  |
| M | 0007409 | axonogenesis | EN-124k-90-group13107.Contig1 | | | | | | | | | | | | | | |  |  |  |
| M | 0007409 | axonogenesis | EN-124k-90-group13121.Contig1 | | | | | | | | | | | | | | |  |  |  |
| M | 0007409 | axonogenesis | EN-124k-90-group13235.Contig1 | | | | | | | | | | | | | | |  |  |  |
| M | 0007409 | axonogenesis | EN-124k-90-group13235.Contig2 | | | | | | | | | | | | | | |  |  |  |
| E | 0007409 | axonogenesis | EN-124k-90-group13342.Contig1 | | | | | | | | | | | | | | |  |  |  |
| M | 0007409 | axonogenesis | EN-124k-90-group13502.Contig2 | | | | | | | | | | | | | | |  |  |  |
| M | 0007409 | axonogenesis | EN-124k-90-group13579.Contig3 | | | | | | | | | | | | | | |  |  |  |
| M | 0007409 | axonogenesis | EN-124k-90-group13646.Contig1 | | | | | | | | | | | | | | |  |  |  |
| M | 0007409 | axonogenesis | EN-124k-90-group14027.Contig1 | | | | | | | | | | | | | | |  |  |  |
| E | 0007409 | axonogenesis | EN-124k-90-group14073.Contig1 | | | | | | | | | | | | | | |  |  |  |
| A | 0007409 | axonogenesis | EN-124k-90-group14199.Contig1 | | | | | | | | | | | | | | |  |  |  |
| M | 0007409 | axonogenesis | EN-124k-90-group14390.Contig2 | | | | | | | | | | | | | | |  |  |  |
| M | 0007409 | axonogenesis | EN-124k-90-group14577.Contig1 | | | | | | | | | | | | | | |  |  |  |
| M | 0007409 | axonogenesis | EN-124k-90-group14580.Contig1 | | | | | | | | | | | | | | |  |  |  |
| M | 0007409 | axonogenesis | EN-124k-90-group14708.Contig1 | | | | | | | | | | | | | | |  |  |  |
| M | 0007409 | axonogenesis | EN-124k-90-group14794.Contig1 | | | | | | | | | | | | | | |  |  |  |
| M | 0007409 | axonogenesis | EN-124k-90-group15757.Contig1 | | | | | | | | | | | | | | |  |  |  |
| A | 0007409 | axonogenesis | EN-124k-90-group29.gs\_82128 | | | | | | | | | | | | | |  |  |  |  |
| A | 0007409 | axonogenesis | EN-124k-90-group65.gs\_37667 | | | | | | | | | | | | | |  |  |  |  |
| E | 0007409 | axonogenesis | EN-124k-90-group70.jgi\_contig\_JGI\_CBBP17523\_fwd | | | | | | | | | | | | | | | | | |
| A | 0007409 | axonogenesis | EN-124k-90-group230.gs\_25885 | | | | | | | | | | | | | | |  |  |  |
| E | 0007409 | axonogenesis | EN-124k-90-group232.jgi\_contig\_JGI\_CBBP16338\_fwd | | | | | | | | | | | | | | | | | |
| E | 0007409 | axonogenesis | EN-124k-90-group232.jgi\_contig\_JGI\_CBBP9823\_fwd | | | | | | | | | | | | | | | | | |
| E | 0007409 | axonogenesis | EN-124k-90-group232.jgi\_contig\_JGI\_CBBP10596\_fwd | | | | | | | | | | | | | | | | | |
| A | 0007409 | axonogenesis | EN-124k-90-group294.gs\_52577 | | | | | | | | | | | | | | |  |  |  |
| A | 0007409 | axonogenesis | EN-124k-90-group735.gs\_7849 | | | | | | | | | | | | | |  |  |  |  |
| E | 0007409 | axonogenesis | EN-124k-90-group760.jgi\_contig\_JGI\_CBBP4536\_fwd | | | | | | | | | | | | | | | | | |
| E | 0007409 | axonogenesis | EN-124k-90-group850.EN\_iowa\_1547 | | | | | | | | | | | | | | |  |  |  |
| E | 0007409 | axonogenesis | EN-124k-90-group868.jgi\_contig\_JGI\_CBBP6965\_fwd | | | | | | | | | | | | | | | | | |
| A | 0007409 | axonogenesis | EN-124k-90-group868.gs\_12961 | | | | | | | | | | | | | | |  |  |  |
| E | 0007409 | axonogenesis | EN-124k-90-group900.EN\_iowa\_15436 | | | | | | | | | | | | | | |  |  |  |
| A | 0007409 | axonogenesis | EN-124k-90-group900.gs\_31783 | | | | | | | | | | | | | | |  |  |  |
| A | 0007409 | axonogenesis | EN-124k-90-group900.gs\_71447 | | | | | | | | | | | | | | |  |  |  |
| E | 0007409 | axonogenesis | EN-124k-90-group939.jgi\_contig\_JGI\_CBBP15138\_fwd | | | | | | | | | | | | | | | | | |
| A | 0007409 | axonogenesis | EN-124k-90-group986.gs\_75970 | | | | | | | | | | | | | | |  |  |  |
| A | 0007409 | axonogenesis | EN-124k-90-group986.gs\_7329 | | | | | | | | | | | | | |  |  |  |  |
| A | 0007409 | axonogenesis | EN-124k-90-group986.gs\_9698 | | | | | | | | | | | | | |  |  |  |  |
| E | 0007409 | axonogenesis | EN-124k-90-group1135.jgi\_unpaired\_JGI\_CBBP19047\_fwd | | | | | | | | | | | | | | | | | |
| E | 0007409 | axonogenesis | EN-124k-90-group1135.jgi\_contig\_JGI\_CBBP19919\_fwd | | | | | | | | | | | | | | | | | |
| A | 0007409 | axonogenesis | EN-124k-90-group1135.gs\_2733 | | | | | | | | | | | | | | |  |  |  |
| E | 0007409 | axonogenesis | EN-124k-90-group1135.EN\_iowa\_9861 | | | | | | | | | | | | | | |  |  |  |
| A | 0007409 | axonogenesis | EN-124k-90-group1135.gs\_17825 | | | | | | | | | | | | | | |  |  |  |
| A | 0007409 | axonogenesis | EN-124k-90-group1153.gs\_635 | | | | | | | | | | | | | |  |  |  |  |
| A | 0007409 | axonogenesis | EN-124k-90-group1153.gs\_33239 | | | | | | | | | | | | | | |  |  |  |
| A | 0007409 | axonogenesis | EN-124k-90-group1290.gs\_26142 | | | | | | | | | | | | | | |  |  |  |
| A | 0007409 | axonogenesis | EN-124k-90-group1290.gs\_22118 | | | | | | | | | | | | | | |  |  |  |
| A | 0007409 | axonogenesis | EN-124k-90-group1290.gs\_35147 | | | | | | | | | | | | | | |  |  |  |
| A | 0007409 | axonogenesis | EN-124k-90-group1290.gs\_13448 | | | | | | | | | | | | | | |  |  |  |
| A | 0007409 | axonogenesis | EN-124k-90-group1290.gs\_16770 | | | | | | | | | | | | | | |  |  |  |
| E | 0007409 | axonogenesis | EN-124k-90-group1290.EN\_iowa\_15637 | | | | | | | | | | | | | | | |  |  |
| A | 0007409 | axonogenesis | EN-124k-90-group1290.gs\_28522 | | | | | | | | | | | | | | |  |  |  |
| A | 0007409 | axonogenesis | EN-124k-90-group1290.gs\_84561 | | | | | | | | | | | | | | |  |  |  |
| A | 0007409 | axonogenesis | EN-124k-90-group1408.gs\_20773 | | | | | | | | | | | | | | |  |  |  |
| A | 0007409 | axonogenesis | EN-124k-90-group1423.gs\_16014 | | | | | | | | | | | | | | |  |  |  |
| A | 0007409 | axonogenesis | EN-124k-90-group1423.gs\_11172 | | | | | | | | | | | | | | |  |  |  |
| A | 0007409 | axonogenesis | EN-124k-90-group1423.gs\_52362 | | | | | | | | | | | | | | |  |  |  |
| A | 0007409 | axonogenesis | EN-124k-90-group1423.gs\_75717 | | | | | | | | | | | | | | |  |  |  |
| E | 0007409 | axonogenesis | EN-124k-90-group1456.jgi\_contig\_JGI\_CBBP4193\_fwd | | | | | | | | | | | | | | | | | |
| E | 0007409 | axonogenesis | EN-124k-90-group1493.jgi\_contig\_JGI\_CBBP6493\_fwd | | | | | | | | | | | | | | | | | |
| A | 0007409 | axonogenesis | EN-124k-90-group1521.gs\_86350 | | | | | | | | | | | | | | |  |  |  |
| E | 0007409 | axonogenesis | EN-124k-90-group1554.jgi\_contig\_JGI\_CBBP18583\_fwd | | | | | | | | | | | | | | | | | |
| A | 0007409 | axonogenesis | EN-124k-90-group1654.gs\_12044 | | | | | | | | | | | | | | |  |  |  |
| E | 0007409 | axonogenesis | EN-124k-90-group1699.jgi\_contig\_JGI\_CBBP7344\_fwd | | | | | | | | | | | | | | | | | |
| A | 0007409 | axonogenesis | EN-124k-90-group1699.gs\_33563 | | | | | | | | | | | | | | |  |  |  |
| A | 0007409 | axonogenesis | EN-124k-90-group1782.gs\_16663 | | | | | | | | | | | | | | |  |  |  |
| A | 0007409 | axonogenesis | EN-124k-90-group1798.gs\_54312 | | | | | | | | | | | | | | |  |  |  |
| A | 0007409 | axonogenesis | EN-124k-90-group1798.gs\_39367 | | | | | | | | | | | | | | |  |  |  |
| E | 0007409 | axonogenesis | EN-124k-90-group1798.EN\_iowa\_16312 | | | | | | | | | | | | | | | |  |  |
| A | 0007409 | axonogenesis | EN-124k-90-group1798.gs\_29292 | | | | | | | | | | | | | | |  |  |  |
| A | 0007409 | axonogenesis | EN-124k-90-group1798.gs\_11661 | | | | | | | | | | | | | | |  |  |  |
| A | 0007409 | axonogenesis | EN-124k-90-group1798.gs\_36996 | | | | | | | | | | | | | | |  |  |  |
| A | 0007409 | axonogenesis | EN-124k-90-group1798.gs\_46615 | | | | | | | | | | | | | | |  |  |  |
| A | 0007409 | axonogenesis | EN-124k-90-group1798.gs\_54963 | | | | | | | | | | | | | | |  |  |  |
| A | 0007409 | axonogenesis | EN-124k-90-group1798.gs\_4498 | | | | | | | | | | | | | | |  |  |  |
| A | 0007409 | axonogenesis | EN-124k-90-group1798.gs\_7178 | | | | | | | | | | | | | | |  |  |  |
| A | 0007409 | axonogenesis | EN-124k-90-group1798.gs\_7576 | | | | | | | | | | | | | | |  |  |  |
| A | 0007409 | axonogenesis | EN-124k-90-group1798.gs\_11790 | | | | | | | | | | | | | | |  |  |  |
| E | 0007409 | axonogenesis | EN-124k-90-group1819.jgi\_contig\_JGI\_CBBP4948\_fwd | | | | | | | | | | | | | | | | | |
| E | 0007409 | axonogenesis | EN-124k-90-group2077.jgi\_paired\_JGI\_CBBP13793\_rev | | | | | | | | | | | | | | | | | |
| E | 0007409 | axonogenesis | EN-124k-90-group2163.jgi\_contig\_JGI\_CBBP7370\_fwd | | | | | | | | | | | | | | | | | |
| E | 0007409 | axonogenesis | EN-124k-90-group2221.jgi\_contig\_JGI\_CBBP11225\_fwd | | | | | | | | | | | | | | | | | |
| E | 0007409 | axonogenesis | EN-124k-90-group2409.EN\_iowa\_9419 | | | | | | | | | | | | | | |  |  |  |
| E | 0007409 | axonogenesis | EN-124k-90-group2409.EN\_iowa\_12940 | | | | | | | | | | | | | | | |  |  |
| E | 0007409 | axonogenesis | EN-124k-90-group2443.EN\_iowa\_8559 | | | | | | | | | | | | | | |  |  |  |
| A | 0007409 | axonogenesis | EN-124k-90-group2449.gs\_35369 | | | | | | | | | | | | | | |  |  |  |
| E | 0007409 | axonogenesis | EN-124k-90-group2451.jgi\_contig\_JGI\_CBBP13284\_fwd | | | | | | | | | | | | | | | | | |
| E | 0007409 | axonogenesis | EN-124k-90-group2525.jgi\_contig\_JGI\_CBBP2745\_fwd | | | | | | | | | | | | | | | | | |
| E | 0007409 | axonogenesis | EN-124k-90-group2525.jgi\_contig\_JGI\_CBBP12002\_fwd | | | | | | | | | | | | | | | | | |
| A | 0007409 | axonogenesis | EN-124k-90-group2525.gs\_26865 | | | | | | | | | | | | | | |  |  |  |
| A | 0007409 | axonogenesis | EN-124k-90-group2525.gs\_28080 | | | | | | | | | | | | | | |  |  |  |
| E | 0007409 | axonogenesis | EN-124k-90-group2525.jgi\_contig\_JGI\_CBBP7217\_fwd | | | | | | | | | | | | | | | | | |
| E | 0007409 | axonogenesis | EN-124k-90-group2600.jgi\_contig\_JGI\_CBBP7087\_fwd | | | | | | | | | | | | | | | | | |
| E | 0007409 | axonogenesis | EN-124k-90-group2600.jgi\_contig\_JGI\_CBBP2795\_fwd | | | | | | | | | | | | | | | | | |
| A | 0007409 | axonogenesis | EN-124k-90-group2619.gs\_45204 | | | | | | | | | | | | | | |  |  |  |
| A | 0007409 | axonogenesis | EN-124k-90-group2811.gs\_23030 | | | | | | | | | | | | | | |  |  |  |
| A | 0007409 | axonogenesis | EN-124k-90-group2811.gs\_38224 | | | | | | | | | | | | | | |  |  |  |
| A | 0007409 | axonogenesis | EN-124k-90-group2811.gs\_15146 | | | | | | | | | | | | | | |  |  |  |
| A | 0007409 | axonogenesis | EN-124k-90-group2903.gs\_16952 | | | | | | | | | | | | | | |  |  |  |
| A | 0007409 | axonogenesis | EN-124k-90-group3067.gs\_19498 | | | | | | | | | | | | | | |  |  |  |
| E | 0007409 | axonogenesis | EN-124k-90-group3114.jgi\_paired\_JGI\_CBBP6377\_fwd | | | | | | | | | | | | | | | | | |
| A | 0007409 | axonogenesis | EN-124k-90-group3202.gs\_15566 | | | | | | | | | | | | | | |  |  |  |
| A | 0007409 | axonogenesis | EN-124k-90-group3238.gs\_87325 | | | | | | | | | | | | | | |  |  |  |
| A | 0007409 | axonogenesis | EN-124k-90-group3357.gs\_11871 | | | | | | | | | | | | | | |  |  |  |
| A | 0007409 | axonogenesis | EN-124k-90-group3522.gs\_9768 | | | | | | | | | | | | | | |  |  |  |
| E | 0007409 | axonogenesis | EN-124k-90-group3584.jgi\_paired\_JGI\_CBBP15905\_fwd | | | | | | | | | | | | | | | | | |
| A | 0007409 | axonogenesis | EN-124k-90-group3594.gs\_29852 | | | | | | | | | | | | | | |  |  |  |
| E | 0007409 | axonogenesis | EN-124k-90-group3611.EN\_iowa\_4463 | | | | | | | | | | | | | | |  |  |  |
| A | 0007409 | axonogenesis | EN-124k-90-group3713.gs\_7861 | | | | | | | | | | | | | | |  |  |  |
| E | 0007409 | axonogenesis | EN-124k-90-group3872.jgi\_contig\_JGI\_CBBP10436\_fwd | | | | | | | | | | | | | | | | | |
| E | 0007409 | axonogenesis | EN-124k-90-group3872.EN\_iowa\_18089 | | | | | | | | | | | | | | | |  |  |
| A | 0007409 | axonogenesis | EN-124k-90-group3883.gs\_85360 | | | | | | | | | | | | | | |  |  |  |
| E | 0007409 | axonogenesis | EN-124k-90-group3905.jgi\_contig\_JGI\_CBBP12601\_fwd | | | | | | | | | | | | | | | | | |
| E | 0007409 | axonogenesis | EN-124k-90-group3947.EN\_iowa\_3123 | | | | | | | | | | | | | | |  |  |  |
| E | 0007409 | axonogenesis | EN-124k-90-group3966.jgi\_contig\_JGI\_CBBP12693\_fwd | | | | | | | | | | | | | | | | | |
| E | 0007409 | axonogenesis | EN-124k-90-group3966.jgi\_contig\_JGI\_CBBP7455\_fwd | | | | | | | | | | | | | | | | | |
| E | 0007409 | axonogenesis | EN-124k-90-group3966.EN\_iowa\_15266 | | | | | | | | | | | | | | | |  |  |
| E | 0007409 | axonogenesis | EN-124k-90-group4049.EN\_iowa\_15523 | | | | | | | | | | | | | | | |  |  |
| E | 0007409 | axonogenesis | EN-124k-90-group4154.EN\_iowa\_7753 | | | | | | | | | | | | | | |  |  |  |
| E | 0007409 | axonogenesis | EN-124k-90-group4154.EN\_iowa\_1478 | | | | | | | | | | | | | | |  |  |  |
| A | 0007409 | axonogenesis | EN-124k-90-group4349.gs\_17809 | | | | | | | | | | | | | | |  |  |  |
| E | 0007409 | axonogenesis | EN-124k-90-group4600.jgi\_contig\_JGI\_CBBP12800\_fwd | | | | | | | | | | | | | | | | | |
| E | 0007409 | axonogenesis | EN-124k-90-group4808.jgi\_contig\_JGI\_CBBP12941\_fwd | | | | | | | | | | | | | | | | | |
| E | 0007409 | axonogenesis | EN-124k-90-group4808.jgi\_contig\_JGI\_CBBP16155\_fwd | | | | | | | | | | | | | | | | | |
| A | 0007409 | axonogenesis | EN-124k-90-group4824.gs\_12585 | | | | | | | | | | | | | | |  |  |  |
| E | 0007409 | axonogenesis | EN-124k-90-group5184.EN\_iowa\_8658 | | | | | | | | | | | | | | |  |  |  |
| A | 0007409 | axonogenesis | EN-124k-90-group5215.gs\_74365 | | | | | | | | | | | | | | |  |  |  |
| A | 0007409 | axonogenesis | EN-124k-90-group5300.gs\_35256 | | | | | | | | | | | | | | |  |  |  |
| A | 0007409 | axonogenesis | EN-124k-90-group5491.gs\_7631 | | | | | | | | | | | | | | |  |  |  |
| A | 0007409 | axonogenesis | EN-124k-90-group5491.gs\_75129 | | | | | | | | | | | | | | |  |  |  |
| A | 0007409 | axonogenesis | EN-124k-90-group5563.gs\_3008 | | | | | | | | | | | | | | |  |  |  |
| E | 0007409 | axonogenesis | EN-124k-90-group5567.jgi\_paired\_JGI\_CBBP16996\_fwd | | | | | | | | | | | | | | | | | |
| E | 0007409 | axonogenesis | EN-124k-90-group5607.jgi\_contig\_JGI\_CBBP445\_fwd | | | | | | | | | | | | | | | | | |
| E | 0007409 | axonogenesis | EN-124k-90-group6204.EN\_iowa\_1799 | | | | | | | | | | | | | | |  |  |  |
| E | 0007409 | axonogenesis | EN-124k-90-group6204.EN\_iowa\_4716 | | | | | | | | | | | | | | |  |  |  |
| E | 0007409 | axonogenesis | EN-124k-90-group6687.EN\_iowa\_2719 | | | | | | | | | | | | | | |  |  |  |
| A | 0007409 | axonogenesis | EN-124k-90-group6883.gs\_22524 | | | | | | | | | | | | | | |  |  |  |
| E | 0007409 | axonogenesis | EN-124k-90-group6923.jgi\_contig\_JGI\_CBBP17789\_fwd | | | | | | | | | | | | | | | | | |
| A | 0007409 | axonogenesis | EN-124k-90-group7440.gs\_75782 | | | | | | | | | | | | | | |  |  |  |
| A | 0007409 | axonogenesis | EN-124k-90-group7650.gs\_30549 | | | | | | | | | | | | | | |  |  |  |
| A | 0007409 | axonogenesis | EN-124k-90-group7650.gs\_59944 | | | | | | | | | | | | | | |  |  |  |
| E | 0007409 | axonogenesis | EN-124k-90-group8221.jgi\_contig\_JGI\_CBBP18338\_fwd | | | | | | | | | | | | | | | | | |
| A | 0007409 | axonogenesis | EN-124k-90-group8484.gs\_85230 | | | | | | | | | | | | | | |  |  |  |
| A | 0007409 | axonogenesis | EN-124k-90-group9299.gs\_87064 | | | | | | | | | | | | | | |  |  |  |
| E | 0007409 | axonogenesis | EN-124k-90-group11408.jgi\_contig\_JGI\_CBBP16915\_fwd | | | | | | | | | | | | | | | | | |
| E | 0007409 | axonogenesis | EN-124k-90-group12653.EN\_iowa\_13251 | | | | | | | | | | | | | | | |  |  |
| E | 0021954 | central nervous system neuron development | EN-124k-90-group343.jgi\_contig\_JGI\_CBBP19299\_fwd | | | | | | | | | | | | | | | | | |
| A | 0021954 | central nervous system neuron development | EN-124k-90-group807.gs\_71281 | | | | | | | | | | | | | | |  |  |  |
| E | 0021954 | central nervous system neuron development | EN-124k-90-group1680.jgi\_contig\_JGI\_CBBP17232\_fwd | | | | | | | | | | | | | | | | | |
| E | 0021954 | central nervous system neuron development | EN-124k-90-group1852.jgi\_paired\_JGI\_CBBP10940\_fwd | | | | | | | | | | | | | | | | | |
| E | 0021954 | central nervous system neuron development | EN-124k-90-group2530.jgi\_paired\_JGI\_CBBP5537\_fwd | | | | | | | | | | | | | | | | | |
| A | 0021954 | central nervous system neuron development | EN-124k-90-group3420.gs\_43284 | | | | | | | | | | | | | | |  |  |  |
| E | 0021954 | central nervous system neuron development | EN-124k-90-group5117.jgi\_paired\_JGI\_CBBP19153\_fwd | | | | | | | | | | | | | | | | | |
| A | 0021954 | central nervous system neuron development | EN-124k-90-group5346.gs\_72008 | | | | | | | | | | | | | | |  |  |  |
| E | 0021954 | central nervous system neuron development | EN-124k-90-group5558.jgi\_contig\_JGI\_CBBP4325\_fwd | | | | | | | | | | | | | | | | | |
| E | 0021954 | central nervous system neuron development | EN-124k-90-group5834.jgi\_contig\_JGI\_CBBP12701\_fwd | | | | | | | | | | | | | | | | | |
| E | 0021954 | central nervous system neuron development | EN-124k-90-group6048.EN\_iowa\_911 | | | | | | | | | | | | | | |  |  |  |
| E | 0021954 | central nervous system neuron development | EN-124k-90-group7594.jgi\_paired\_JGI\_CBBP17768\_fwd | | | | | | | | | | | | | | | | | |
| A | 0021954 | central nervous system neuron development | EN-124k-90-group7595.gs\_36261 | | | | | | | | | | | | | | |  |  |  |
| E | 0021954 | central nervous system neuron development | EN-124k-90-group7598.jgi\_unpaired\_JGI\_CBBP18343\_fwd | | | | | | | | | | | | | | | | | |
| A | 0021954 | central nervous system neuron development | EN-124k-90-group8023.gs\_82870 | | | | | | | | | | | | | | |  |  |  |
| E | 0021954 | central nervous system neuron development | EN-124k-90-group8107.jgi\_paired\_JGI\_CBBP6197\_fwd | | | | | | | | | | | | | | | | | |
| A | 0021954 | central nervous system neuron development | EN-124k-90-group8310.gs\_24913 | | | | | | | | | | | | | | |  |  |  |
| A | 0021954 | central nervous system neuron development | EN-124k-90-group9714.gs\_64750 | | | | | | | | | | | | | | |  |  |  |
| E | 0021954 | central nervous system neuron development | EN-124k-90-group9989.EN\_iowa\_3399 | | | | | | | | | | | | | | |  |  |  |
| E | 0021954 | central nervous system neuron development | EN-124k-90-group10304.jgi\_paired\_JGI\_CBBP11045\_fwd | | | | | | | | | | | | | | | | | |
| A | 0021954 | central nervous system neuron development | EN-124k-90-group10312.gs\_22624 | | | | | | | | | | | | | | |  |  |  |
| E | 0021954 | central nervous system neuron development | EN-124k-90-group10416.jgi\_contig\_JGI\_CBBP3274\_fwd | | | | | | | | | | | | | | | | | |
| A | 0021954 | central nervous system neuron development | EN-124k-90-group10692.gs\_44993 | | | | | | | | | | | | | | |  |  |  |
| A | 0021954 | central nervous system neuron development | EN-124k-90-group10718.gs\_26673 | | | | | | | | | | | | | | |  |  |  |
| A | 0021954 | central nervous system neuron development | EN-124k-90-group11354.gs\_78099 | | | | | | | | | | | | | | |  |  |  |
| E | 0021954 | central nervous system neuron development | EN-124k-90-group11438.jgi\_paired\_JGI\_CBBP9709\_fwd | | | | | | | | | | | | | | | | | |
| E | 0021954 | central nervous system neuron development | EN-124k-90-group11605.jgi\_paired\_JGI\_CBBP17453\_rev | | | | | | | | | | | | | | | | | |
| A | 0021954 | central nervous system neuron development | EN-124k-90-group11738.gs\_53113 | | | | | | | | | | | | | | |  |  |  |
| E | 0021954 | central nervous system neuron development | EN-124k-90-group12452.EN\_iowa\_9722 | | | | | | | | | | | | | | | |  |  |
| E | 0021954 | central nervous system neuron development | EN-124k-90-group12595.jgi\_contig\_JGI\_CBBP15955\_fwd | | | | | | | | | | | | | | | | | |
| E | 0021954 | central nervous system neuron development | EN-124k-90-group13011.jgi\_paired\_JGI\_CBBP19322\_fwd | | | | | | | | | | | | | | | | | |
| A | 0021954 | central nervous system neuron development | EN-124k-90-group13823.gs\_32410 | | | | | | | | | | | | | | |  |  |  |
| E | 0021954 | central nervous system neuron development | EN-124k-90-group13911.jgi\_contig\_JGI\_CBBP10628\_fwd | | | | | | | | | | | | | | | | | |
| A | 0021954 | central nervous system neuron development | EN-124k-90-group13938.gs\_32571 | | | | | | | | | | | | | | |  |  |  |
| A | 0021954 | central nervous system neuron development | EN-124k-90-group14563.gs\_48486 | | | | | | | | | | | | | | |  |  |  |
| E | 0021954 | central nervous system neuron development | EN-124k-90-group14931.jgi\_paired\_JGI\_CBBP10444\_fwd | | | | | | | | | | | | | | | | | |
| A | 0021954 | central nervous system neuron development | EN-124k-90-group15056.gs\_73097 | | | | | | | | | | | | | | |  |  |  |
| A | 0021954 | central nervous system neuron development | EN-124k-90-group15229.gs\_75551 | | | | | | | | | | | | | | |  |  |  |
| E | 0021954 | central nervous system neuron development | EN-124k-90-group15289.jgi\_paired\_JGI\_CBBP6226\_fwd | | | | | | | | | | | | | | | | | |
| E | 0021954 | central nervous system neuron development | EN-124k-90-group15290.EN\_iowa\_9903 | | | | | | | | | | | | | | | |  |  |
| E | 0021954 | central nervous system neuron development | EN-124k-90-group15532.EN\_iowa\_5883 | | | | | | | | | | | | | | | |  |  |
| A | 0021954 | central nervous system neuron development | EN-124k-90-group15770.gs\_45569 | | | | | | | | | | | | | | |  |  |  |
| A | 0021954 | central nervous system neuron development | EN-124k-90-group15856.gs\_15241 | | | | | | | | | | | | | | |  |  |  |
| E | 0021954 | central nervous system neuron development | EN-124k-90-group16034.jgi\_paired\_JGI\_CBBP10733\_fwd | | | | | | | | | | | | | | | | | |
| E | 0021954 | central nervous system neuron development | EN-124k-90-group16143.jgi\_paired\_JGI\_CBBP4408\_fwd | | | | | | | | | | | | | | | | | |
| E | 0021954 | central nervous system neuron development | EN-124k-90-group16262.jgi\_paired\_JGI\_CBBP4191\_fwd | | | | | | | | | | | | | | | | | |
| A | 0021954 | central nervous system neuron development | EN-124k-90-group16285.gs\_47129 | | | | | | | | | | | | | | |  |  |  |
| A | 0021954 | central nervous system neuron development | EN-124k-90-group16439.gs\_54729 | | | | | | | | | | | | | | |  |  |  |
| E | 0021954 | central nervous system neuron development | EN-124k-90-group16705.jgi\_paired\_JGI\_CBBP19935\_rev | | | | | | | | | | | | | | | | | |
| A | 0021954 | central nervous system neuron development | EN-124k-90-group16837.gs\_15971 | | | | | | | | | | | | | | |  |  |  |
| A | 0021954 | central nervous system neuron development | EN-124k-90-group16910.gs\_22871 | | | | | | | | | | | | | | |  |  |  |
| A | 0021954 | central nervous system neuron development | EN-124k-90-group16929.gs\_43290 | | | | | | | | | | | | | | |  |  |  |
| E | 0021954 | central nervous system neuron development | EN-124k-90-group17086.jgi\_paired\_JGI\_CBBP6417\_rev | | | | | | | | | | | | | | | | | |
| E | 0021954 | central nervous system neuron development | EN-124k-90-group17091.jgi\_paired\_JGI\_CBBP10179\_rev | | | | | | | | | | | | | | | | | |
| A | 0021954 | central nervous system neuron development | EN-124k-90-group17442.gs\_63748 | | | | | | | | | | | | | | |  |  |  |
| A | 0021954 | central nervous system neuron development | EN-124k-90-group17524.gs\_80802 | | | | | | | | | | | | | | |  |  |  |
| A | 0021954 | central nervous system neuron development | EN-124k-90-group17556.gs\_87114 | | | | | | | | | | | | | | |  |  |  |
| A | 0021954 | central nervous system neuron development | EN-124k-90-group17563.gs\_80410 | | | | | | | | | | | | | | |  |  |  |
| A | 0021954 | central nervous system neuron development | EN-124k-90-group17930.gs\_83513 | | | | | | | | | | | | | | |  |  |  |
| A | 0021954 | central nervous system neuron development | EN-124k-90-group18038.gs\_30262 | | | | | | | | | | | | | | |  |  |  |
| M | 0021954 | central nervous system neuron development | EN-124k-90-group13.Contig4 | | | | | | | | | | | | | |  |  |  |  |
| M | 0021954 | central nervous system neuron development | EN-124k-90-group70.Contig6 | | | | | | | | | | | | | |  |  |  |  |
| M | 0021954 | central nervous system neuron development | EN-124k-90-group138.Contig1 | | | | | | | | | | | | | |  |  |  |  |
| M | 0021954 | central nervous system neuron development | EN-124k-90-group138.Contig62 | | | | | | | | | | | | | |  |  |  |  |
| M | 0021954 | central nervous system neuron development | EN-124k-90-group229.Contig1 | | | | | | | | | | | | | |  |  |  |  |
| M | 0021954 | central nervous system neuron development | EN-124k-90-group230.Contig1 | | | | | | | | | | | | | |  |  |  |  |
| M | 0021954 | central nervous system neuron development | EN-124k-90-group324.Contig1 | | | | | | | | | | | | | |  |  |  |  |
| M | 0021954 | central nervous system neuron development | EN-124k-90-group478.Contig1 | | | | | | | | | | | | | |  |  |  |  |
| M | 0021954 | central nervous system neuron development | EN-124k-90-group481.Contig4 | | | | | | | | | | | | | |  |  |  |  |
| M | 0021954 | central nervous system neuron development | EN-124k-90-group550.Contig1 | | | | | | | | | | | | | |  |  |  |  |
| M | 0021954 | central nervous system neuron development | EN-124k-90-group550.Contig2 | | | | | | | | | | | | | |  |  |  |  |
| M | 0021954 | central nervous system neuron development | EN-124k-90-group593.Contig1 | | | | | | | | | | | | | |  |  |  |  |
| M | 0021954 | central nervous system neuron development | EN-124k-90-group630.Contig2 | | | | | | | | | | | | | |  |  |  |  |
| M | 0021954 | central nervous system neuron development | EN-124k-90-group658.Contig1 | | | | | | | | | | | | | |  |  |  |  |
| M | 0021954 | central nervous system neuron development | EN-124k-90-group658.Contig3 | | | | | | | | | | | | | |  |  |  |  |
| M | 0021954 | central nervous system neuron development | EN-124k-90-group682.Contig1 | | | | | | | | | | | | | |  |  |  |  |
| M | 0021954 | central nervous system neuron development | EN-124k-90-group900.Contig1 | | | | | | | | | | | | | |  |  |  |  |
| M | 0021954 | central nervous system neuron development | EN-124k-90-group1167.Contig3 | | | | | | | | | | | | | |  |  |  |  |
| M | 0021954 | central nervous system neuron development | EN-124k-90-group1266.Contig1 | | | | | | | | | | | | | |  |  |  |  |
| M | 0021954 | central nervous system neuron development | EN-124k-90-group1266.Contig2 | | | | | | | | | | | | | |  |  |  |  |
| M | 0021954 | central nervous system neuron development | EN-124k-90-group1266.Contig3 | | | | | | | | | | | | | |  |  |  |  |
| M | 0021954 | central nervous system neuron development | EN-124k-90-group1297.Contig1 | | | | | | | | | | | | | |  |  |  |  |
| M | 0021954 | central nervous system neuron development | EN-124k-90-group1304.Contig2 | | | | | | | | | | | | | |  |  |  |  |
| M | 0021954 | central nervous system neuron development | EN-124k-90-group1304.Contig4 | | | | | | | | | | | | | |  |  |  |  |
| M | 0021954 | central nervous system neuron development | EN-124k-90-group1304.Contig8 | | | | | | | | | | | | | |  |  |  |  |
| M | 0021954 | central nervous system neuron development | EN-124k-90-group1423.Contig14 | | | | | | | | | | | | | | |  |  |  |
| M | 0021954 | central nervous system neuron development | EN-124k-90-group1456.Contig4 | | | | | | | | | | | | | |  |  |  |  |
| M | 0021954 | central nervous system neuron development | EN-124k-90-group1456.Contig8 | | | | | | | | | | | | | |  |  |  |  |
| A | 0021954 | central nervous system neuron development | EN-124k-90-group1482.Contig1 | | | | | | | | | | | | | |  |  |  |  |
| M | 0021954 | central nervous system neuron development | EN-124k-90-group1735.Contig6 | | | | | | | | | | | | | |  |  |  |  |
| M | 0021954 | central nervous system neuron development | EN-124k-90-group1915.Contig1 | | | | | | | | | | | | | |  |  |  |  |
| M | 0021954 | central nervous system neuron development | EN-124k-90-group1960.Contig1 | | | | | | | | | | | | | |  |  |  |  |
| M | 0021954 | central nervous system neuron development | EN-124k-90-group2004.Contig3 | | | | | | | | | | | | | |  |  |  |  |
| M | 0021954 | central nervous system neuron development | EN-124k-90-group2074.Contig1 | | | | | | | | | | | | | |  |  |  |  |
| M | 0021954 | central nervous system neuron development | EN-124k-90-group2081.Contig1 | | | | | | | | | | | | | |  |  |  |  |
| M | 0021954 | central nervous system neuron development | EN-124k-90-group2081.Contig3 | | | | | | | | | | | | | |  |  |  |  |
| M | 0021954 | central nervous system neuron development | EN-124k-90-group2409.Contig3 | | | | | | | | | | | | | |  |  |  |  |
| M | 0021954 | central nervous system neuron development | EN-124k-90-group2428.Contig1 | | | | | | | | | | | | | |  |  |  |  |
| M | 0021954 | central nervous system neuron development | EN-124k-90-group2455.Contig1 | | | | | | | | | | | | | |  |  |  |  |
| M | 0021954 | central nervous system neuron development | EN-124k-90-group2582.Contig1 | | | | | | | | | | | | | |  |  |  |  |
| M | 0021954 | central nervous system neuron development | EN-124k-90-group2607.Contig1 | | | | | | | | | | | | | |  |  |  |  |
| M | 0021954 | central nervous system neuron development | EN-124k-90-group2607.Contig2 | | | | | | | | | | | | | |  |  |  |  |
| M | 0021954 | central nervous system neuron development | EN-124k-90-group2607.Contig7 | | | | | | | | | | | | | |  |  |  |  |
| M | 0021954 | central nervous system neuron development | EN-124k-90-group2681.Contig1 | | | | | | | | | | | | | |  |  |  |  |
| M | 0021954 | central nervous system neuron development | EN-124k-90-group2811.Contig1 | | | | | | | | | | | | | |  |  |  |  |
| M | 0021954 | central nervous system neuron development | EN-124k-90-group2811.Contig2 | | | | | | | | | | | | | |  |  |  |  |
| M | 0021954 | central nervous system neuron development | EN-124k-90-group2829.Contig2 | | | | | | | | | | | | | |  |  |  |  |
| M | 0021954 | central nervous system neuron development | EN-124k-90-group2846.Contig1 | | | | | | | | | | | | | |  |  |  |  |
| M | 0021954 | central nervous system neuron development | EN-124k-90-group2866.Contig1 | | | | | | | | | | | | | |  |  |  |  |
| M | 0021954 | central nervous system neuron development | EN-124k-90-group2940.Contig2 | | | | | | | | | | | | | |  |  |  |  |
| M | 0021954 | central nervous system neuron development | EN-124k-90-group3058.Contig1 | | | | | | | | | | | | | |  |  |  |  |
| M | 0021954 | central nervous system neuron development | EN-124k-90-group3082.Contig2 | | | | | | | | | | | | | |  |  |  |  |
| M | 0021954 | central nervous system neuron development | EN-124k-90-group3299.Contig1 | | | | | | | | | | | | | |  |  |  |  |
| M | 0021954 | central nervous system neuron development | EN-124k-90-group3313.Contig1 | | | | | | | | | | | | | |  |  |  |  |
| M | 0021954 | central nervous system neuron development | EN-124k-90-group3313.Contig2 | | | | | | | | | | | | | |  |  |  |  |
| M | 0021954 | central nervous system neuron development | EN-124k-90-group3313.Contig3 | | | | | | | | | | | | | |  |  |  |  |
| M | 0021954 | central nervous system neuron development | EN-124k-90-group3315.Contig6 | | | | | | | | | | | | | |  |  |  |  |
| M | 0021954 | central nervous system neuron development | EN-124k-90-group3316.Contig1 | | | | | | | | | | | | | |  |  |  |  |
| M | 0021954 | central nervous system neuron development | EN-124k-90-group3479.Contig1 | | | | | | | | | | | | | |  |  |  |  |
| M | 0021954 | central nervous system neuron development | EN-124k-90-group3586.Contig1 | | | | | | | | | | | | | |  |  |  |  |
| M | 0021954 | central nervous system neuron development | EN-124k-90-group3606.Contig2 | | | | | | | | | | | | | |  |  |  |  |
| M | 0021954 | central nervous system neuron development | EN-124k-90-group3615.Contig4 | | | | | | | | | | | | | |  |  |  |  |
| M | 0021954 | central nervous system neuron development | EN-124k-90-group3620.Contig3 | | | | | | | | | | | | | |  |  |  |  |
| M | 0021954 | central nervous system neuron development | EN-124k-90-group3661.Contig1 | | | | | | | | | | | | | |  |  |  |  |
| M | 0021954 | central nervous system neuron development | EN-124k-90-group3897.Contig1 | | | | | | | | | | | | | |  |  |  |  |
| M | 0021954 | central nervous system neuron development | EN-124k-90-group4183.Contig1 | | | | | | | | | | | | | |  |  |  |  |
| M | 0021954 | central nervous system neuron development | EN-124k-90-group4339.Contig1 | | | | | | | | | | | | | |  |  |  |  |
| M | 0021954 | central nervous system neuron development | EN-124k-90-group4374.Contig1 | | | | | | | | | | | | | |  |  |  |  |
| M | 0021954 | central nervous system neuron development | EN-124k-90-group4583.Contig4 | | | | | | | | | | | | | |  |  |  |  |
| M | 0021954 | central nervous system neuron development | EN-124k-90-group4753.Contig1 | | | | | | | | | | | | | |  |  |  |  |
| M | 0021954 | central nervous system neuron development | EN-124k-90-group4803.Contig1 | | | | | | | | | | | | | |  |  |  |  |
| M | 0021954 | central nervous system neuron development | EN-124k-90-group4803.Contig2 | | | | | | | | | | | | | |  |  |  |  |
| M | 0021954 | central nervous system neuron development | EN-124k-90-group4929.Contig2 | | | | | | | | | | | | | |  |  |  |  |
| M | 0021954 | central nervous system neuron development | EN-124k-90-group5173.Contig1 | | | | | | | | | | | | | |  |  |  |  |
| M | 0021954 | central nervous system neuron development | EN-124k-90-group5203.Contig2 | | | | | | | | | | | | | |  |  |  |  |
| M | 0021954 | central nervous system neuron development | EN-124k-90-group5252.Contig1 | | | | | | | | | | | | | |  |  |  |  |
| M | 0021954 | central nervous system neuron development | EN-124k-90-group5273.Contig2 | | | | | | | | | | | | | |  |  |  |  |
| M | 0021954 | central nervous system neuron development | EN-124k-90-group5411.Contig1 | | | | | | | | | | | | | |  |  |  |  |
| M | 0021954 | central nervous system neuron development | EN-124k-90-group5471.Contig3 | | | | | | | | | | | | | |  |  |  |  |
| M | 0021954 | central nervous system neuron development | EN-124k-90-group5491.Contig1 | | | | | | | | | | | | | |  |  |  |  |
| M | 0021954 | central nervous system neuron development | EN-124k-90-group5491.Contig2 | | | | | | | | | | | | | |  |  |  |  |
| M | 0021954 | central nervous system neuron development | EN-124k-90-group5527.Contig2 | | | | | | | | | | | | | |  |  |  |  |
| M | 0021954 | central nervous system neuron development | EN-124k-90-group5542.Contig1 | | | | | | | | | | | | | |  |  |  |  |
| A | 0021954 | central nervous system neuron development | EN-124k-90-group5543.Contig1 | | | | | | | | | | | | | |  |  |  |  |
| M | 0021954 | central nervous system neuron development | EN-124k-90-group5607.Contig1 | | | | | | | | | | | | | |  |  |  |  |
| M | 0021954 | central nervous system neuron development | EN-124k-90-group6044.Contig2 | | | | | | | | | | | | | |  |  |  |  |
| M | 0021954 | central nervous system neuron development | EN-124k-90-group6204.Contig1 | | | | | | | | | | | | | |  |  |  |  |
| M | 0021954 | central nervous system neuron development | EN-124k-90-group6204.Contig2 | | | | | | | | | | | | | |  |  |  |  |
| M | 0021954 | central nervous system neuron development | EN-124k-90-group6400.Contig1 | | | | | | | | | | | | | |  |  |  |  |
| M | 0021954 | central nervous system neuron development | EN-124k-90-group6472.Contig1 | | | | | | | | | | | | | |  |  |  |  |
| M | 0021954 | central nervous system neuron development | EN-124k-90-group6472.Contig2 | | | | | | | | | | | | | |  |  |  |  |
| M | 0021954 | central nervous system neuron development | EN-124k-90-group6666.Contig1 | | | | | | | | | | | | | |  |  |  |  |
| M | 0021954 | central nervous system neuron development | EN-124k-90-group6923.Contig1 | | | | | | | | | | | | | |  |  |  |  |
| M | 0021954 | central nervous system neuron development | EN-124k-90-group6923.Contig2 | | | | | | | | | | | | | |  |  |  |  |
| M | 0021954 | central nervous system neuron development | EN-124k-90-group7149.Contig2 | | | | | | | | | | | | | |  |  |  |  |
| M | 0021954 | central nervous system neuron development | EN-124k-90-group7231.Contig1 | | | | | | | | | | | | | |  |  |  |  |
| M | 0021954 | central nervous system neuron development | EN-124k-90-group7343.Contig1 | | | | | | | | | | | | | |  |  |  |  |
| M | 0021954 | central nervous system neuron development | EN-124k-90-group7382.Contig2 | | | | | | | | | | | | | |  |  |  |  |
| M | 0021954 | central nervous system neuron development | EN-124k-90-group7528.Contig1 | | | | | | | | | | | | | |  |  |  |  |
| M | 0021954 | central nervous system neuron development | EN-124k-90-group7787.Contig1 | | | | | | | | | | | | | |  |  |  |  |
| M | 0021954 | central nervous system neuron development | EN-124k-90-group7787.Contig2 | | | | | | | | | | | | | |  |  |  |  |
| M | 0021954 | central nervous system neuron development | EN-124k-90-group7787.Contig3 | | | | | | | | | | | | | |  |  |  |  |
| M | 0021954 | central nervous system neuron development | EN-124k-90-group7866.Contig1 | | | | | | | | | | | | | |  |  |  |  |
| M | 0021954 | central nervous system neuron development | EN-124k-90-group7915.Contig1 | | | | | | | | | | | | | |  |  |  |  |
| M | 0021954 | central nervous system neuron development | EN-124k-90-group8079.Contig1 | | | | | | | | | | | | | |  |  |  |  |
| M | 0021954 | central nervous system neuron development | EN-124k-90-group8079.Contig2 | | | | | | | | | | | | | |  |  |  |  |
| M | 0021954 | central nervous system neuron development | EN-124k-90-group8418.Contig1 | | | | | | | | | | | | | |  |  |  |  |
| M | 0021954 | central nervous system neuron development | EN-124k-90-group8492.Contig1 | | | | | | | | | | | | | |  |  |  |  |
| A | 0021954 | central nervous system neuron development | EN-124k-90-group8565.Contig1 | | | | | | | | | | | | | |  |  |  |  |
| M | 0021954 | central nervous system neuron development | EN-124k-90-group8799.Contig2 | | | | | | | | | | | | | |  |  |  |  |
| M | 0021954 | central nervous system neuron development | EN-124k-90-group8850.Contig1 | | | | | | | | | | | | | |  |  |  |  |
| M | 0021954 | central nervous system neuron development | EN-124k-90-group9079.Contig1 | | | | | | | | | | | | | |  |  |  |  |
| M | 0021954 | central nervous system neuron development | EN-124k-90-group9299.Contig3 | | | | | | | | | | | | | |  |  |  |  |
| M | 0021954 | central nervous system neuron development | EN-124k-90-group9388.Contig1 | | | | | | | | | | | | | |  |  |  |  |
| M | 0021954 | central nervous system neuron development | EN-124k-90-group9388.Contig2 | | | | | | | | | | | | | |  |  |  |  |
| M | 0021954 | central nervous system neuron development | EN-124k-90-group9446.Contig1 | | | | | | | | | | | | | |  |  |  |  |
| M | 0021954 | central nervous system neuron development | EN-124k-90-group9819.Contig1 | | | | | | | | | | | | | |  |  |  |  |
| M | 0021954 | central nervous system neuron development | EN-124k-90-group9843.Contig1 | | | | | | | | | | | | | |  |  |  |  |
| M | 0021954 | central nervous system neuron development | EN-124k-90-group10085.Contig1 | | | | | | | | | | | | | | |  |  |  |
| M | 0021954 | central nervous system neuron development | EN-124k-90-group10085.Contig2 | | | | | | | | | | | | | | |  |  |  |
| M | 0021954 | central nervous system neuron development | EN-124k-90-group10221.Contig1 | | | | | | | | | | | | | | |  |  |  |
| M | 0021954 | central nervous system neuron development | EN-124k-90-group10467.Contig1 | | | | | | | | | | | | | | |  |  |  |
| M | 0021954 | central nervous system neuron development | EN-124k-90-group10637.Contig1 | | | | | | | | | | | | | | |  |  |  |
| M | 0021954 | central nervous system neuron development | EN-124k-90-group10877.Contig1 | | | | | | | | | | | | | | |  |  |  |
| M | 0021954 | central nervous system neuron development | EN-124k-90-group10912.Contig1 | | | | | | | | | | | | | | |  |  |  |
| M | 0021954 | central nervous system neuron development | EN-124k-90-group10928.Contig1 | | | | | | | | | | | | | | |  |  |  |
| M | 0021954 | central nervous system neuron development | EN-124k-90-group11235.Contig1 | | | | | | | | | | | | | | |  |  |  |
| M | 0021954 | central nervous system neuron development | EN-124k-90-group11399.Contig2 | | | | | | | | | | | | | | |  |  |  |
| M | 0021954 | central nervous system neuron development | EN-124k-90-group12099.Contig1 | | | | | | | | | | | | | | |  |  |  |
| M | 0021954 | central nervous system neuron development | EN-124k-90-group12115.Contig1 | | | | | | | | | | | | | | |  |  |  |
| M | 0021954 | central nervous system neuron development | EN-124k-90-group12519.Contig1 | | | | | | | | | | | | | | |  |  |  |
| M | 0021954 | central nervous system neuron development | EN-124k-90-group12638.Contig1 | | | | | | | | | | | | | | |  |  |  |
| M | 0021954 | central nervous system neuron development | EN-124k-90-group12973.Contig1 | | | | | | | | | | | | | | |  |  |  |
| E | 0021954 | central nervous system neuron development | EN-124k-90-group13069.Contig2 | | | | | | | | | | | | | | |  |  |  |
| M | 0021954 | central nervous system neuron development | EN-124k-90-group13235.Contig1 | | | | | | | | | | | | | | |  |  |  |
| A | 0021954 | central nervous system neuron development | EN-124k-90-group14199.Contig1 | | | | | | | | | | | | | | |  |  |  |
| M | 0021954 | central nervous system neuron development | EN-124k-90-group14514.Contig1 | | | | | | | | | | | | | | |  |  |  |
| M | 0021954 | central nervous system neuron development | EN-124k-90-group14708.Contig1 | | | | | | | | | | | | | | |  |  |  |
| A | 0021954 | central nervous system neuron development | EN-124k-90-group230.gs\_25885 | | | | | | | | | | | | | | |  |  |  |
| E | 0021954 | central nervous system neuron development | EN-124k-90-group478.jgi\_contig\_JGI\_CBBP10824\_fwd | | | | | | | | | | | | | | | | | |
| A | 0021954 | central nervous system neuron development | EN-124k-90-group1423.gs\_11172 | | | | | | | | | | | | | | |  |  |  |
| A | 0021954 | central nervous system neuron development | EN-124k-90-group1423.gs\_52362 | | | | | | | | | | | | | | |  |  |  |
| A | 0021954 | central nervous system neuron development | EN-124k-90-group1423.gs\_75717 | | | | | | | | | | | | | | |  |  |  |
| E | 0021954 | central nervous system neuron development | EN-124k-90-group3058.jgi\_contig\_JGI\_CBBP9922\_fwd | | | | | | | | | | | | | | | | | |
| A | 0021954 | central nervous system neuron development | EN-124k-90-group3615.gs\_46165 | | | | | | | | | | | | | | |  |  |  |
| A | 0021954 | central nervous system neuron development | EN-124k-90-group3615.gs\_63719 | | | | | | | | | | | | | | |  |  |  |
| A | 0021954 | central nervous system neuron development | EN-124k-90-group5203.gs\_53868 | | | | | | | | | | | | | | |  |  |  |
| A | 0021954 | central nervous system neuron development | EN-124k-90-group5203.gs\_41715 | | | | | | | | | | | | | | |  |  |  |
| A | 0021954 | central nervous system neuron development | EN-124k-90-group5203.gs\_19246 | | | | | | | | | | | | | | |  |  |  |
| A | 0021954 | central nervous system neuron development | EN-124k-90-group5491.gs\_7631 | | | | | | | | | | | | | | |  |  |  |
| A | 0021954 | central nervous system neuron development | EN-124k-90-group5491.gs\_75129 | | | | | | | | | | | | | | |  |  |  |
| E | 0021954 | central nervous system neuron development | EN-124k-90-group5607.jgi\_contig\_JGI\_CBBP445\_fwd | | | | | | | | | | | | | | | | | |
| E | 0021954 | central nervous system neuron development | EN-124k-90-group6204.EN\_iowa\_1799 | | | | | | | | | | | | | | |  |  |  |
| E | 0021954 | central nervous system neuron development | EN-124k-90-group6204.EN\_iowa\_4716 | | | | | | | | | | | | | | |  |  |  |
| E | 0021954 | central nervous system neuron development | EN-124k-90-group6472.jgi\_contig\_JGI\_CBBP2402\_fwd | | | | | | | | | | | | | | | | | |
| E | 0021954 | central nervous system neuron development | EN-124k-90-group6923.jgi\_contig\_JGI\_CBBP17789\_fwd | | | | | | | | | | | | | | | | | |
| A | 0021952 | central nervous system projection neuron axonogenesis | EN-124k-90-group807.gs\_71281 | | | | | | | | | | | | | | |  |  |  |
| E | 0021952 | central nervous system projection neuron axonogenesis | EN-124k-90-group1306.EN\_iowa\_2354 | | | | | | | | | | | | | | |  |  |  |
| E | 0021952 | central nervous system projection neuron axonogenesis | EN-124k-90-group2530.jgi\_paired\_JGI\_CBBP5537\_fwd | | | | | | | | | | | | | | | | | |
| E | 0021952 | central nervous system projection neuron axonogenesis | EN-124k-90-group5117.jgi\_paired\_JGI\_CBBP19153\_fwd | | | | | | | | | | | | | | | | | |
| E | 0021952 | central nervous system projection neuron axonogenesis | EN-124k-90-group5741.jgi\_paired\_JGI\_CBBP19133\_fwd | | | | | | | | | | | | | | | | | |
| E | 0021952 | central nervous system projection neuron axonogenesis | EN-124k-90-group5834.jgi\_contig\_JGI\_CBBP12701\_fwd | | | | | | | | | | | | | | | | | |
| E | 0021952 | central nervous system projection neuron axonogenesis | EN-124k-90-group5954.jgi\_paired\_JGI\_CBBP7330\_fwd | | | | | | | | | | | | | | | | | |
| E | 0021952 | central nervous system projection neuron axonogenesis | EN-124k-90-group6737.jgi\_contig\_JGI\_CBBP17820\_fwd | | | | | | | | | | | | | | | | | |
| E | 0021952 | central nervous system projection neuron axonogenesis | EN-124k-90-group7594.jgi\_paired\_JGI\_CBBP17768\_fwd | | | | | | | | | | | | | | | | | |
| A | 0021952 | central nervous system projection neuron axonogenesis | EN-124k-90-group8310.gs\_24913 | | | | | | | | | | | | | | |  |  |  |
| A | 0021952 | central nervous system projection neuron axonogenesis | EN-124k-90-group8658.gs\_22315 | | | | | | | | | | | | | | |  |  |  |
| A | 0021952 | central nervous system projection neuron axonogenesis | EN-124k-90-group9467.gs\_16039 | | | | | | | | | | | | | | |  |  |  |
| A | 0021952 | central nervous system projection neuron axonogenesis | EN-124k-90-group9714.gs\_64750 | | | | | | | | | | | | | | |  |  |  |
| E | 0021952 | central nervous system projection neuron axonogenesis | EN-124k-90-group9942.jgi\_paired\_JGI\_CBBP11089\_fwd | | | | | | | | | | | | | | | | | |
| E | 0021952 | central nervous system projection neuron axonogenesis | EN-124k-90-group9989.EN\_iowa\_3399 | | | | | | | | | | | | | | |  |  |  |
| E | 0021952 | central nervous system projection neuron axonogenesis | EN-124k-90-group10304.jgi\_paired\_JGI\_CBBP11045\_fwd | | | | | | | | | | | | | | | | | |
| A | 0021952 | central nervous system projection neuron axonogenesis | EN-124k-90-group10312.gs\_22624 | | | | | | | | | | | | | | |  |  |  |
| A | 0021952 | central nervous system projection neuron axonogenesis | EN-124k-90-group10935.gs\_64995 | | | | | | | | | | | | | | |  |  |  |
| E | 0021952 | central nervous system projection neuron axonogenesis | EN-124k-90-group11438.jgi\_paired\_JGI\_CBBP9709\_fwd | | | | | | | | | | | | | | | | | |
| E | 0021952 | central nervous system projection neuron axonogenesis | EN-124k-90-group11500.EN\_iowa\_7830 | | | | | | | | | | | | | | | |  |  |
| A | 0021952 | central nervous system projection neuron axonogenesis | EN-124k-90-group11738.gs\_53113 | | | | | | | | | | | | | | |  |  |  |
| E | 0021952 | central nervous system projection neuron axonogenesis | EN-124k-90-group12012.jgi\_contig\_JGI\_CBBP10480\_fwd | | | | | | | | | | | | | | | | | |
| E | 0021952 | central nervous system projection neuron axonogenesis | EN-124k-90-group12191.jgi\_paired\_JGI\_CBBP10887\_fwd | | | | | | | | | | | | | | | | | |
| E | 0021952 | central nervous system projection neuron axonogenesis | EN-124k-90-group12452.EN\_iowa\_9722 | | | | | | | | | | | | | | | |  |  |
| E | 0021952 | central nervous system projection neuron axonogenesis | EN-124k-90-group13011.jgi\_paired\_JGI\_CBBP19322\_fwd | | | | | | | | | | | | | | | | | |
| E | 0021952 | central nervous system projection neuron axonogenesis | EN-124k-90-group13366.jgi\_paired\_JGI\_CBBP16258\_fwd | | | | | | | | | | | | | | | | | |
| E | 0021952 | central nervous system projection neuron axonogenesis | EN-124k-90-group13491.jgi\_paired\_JGI\_CBBP11089\_rev | | | | | | | | | | | | | | | | | |
| A | 0021952 | central nervous system projection neuron axonogenesis | EN-124k-90-group13938.gs\_32571 | | | | | | | | | | | | | | |  |  |  |
| E | 0021952 | central nervous system projection neuron axonogenesis | EN-124k-90-group14335.jgi\_paired\_JGI\_CBBP12938\_fwd | | | | | | | | | | | | | | | | | |
| A | 0021952 | central nervous system projection neuron axonogenesis | EN-124k-90-group14545.gs\_14842 | | | | | | | | | | | | | | |  |  |  |
| A | 0021952 | central nervous system projection neuron axonogenesis | EN-124k-90-group14563.gs\_48486 | | | | | | | | | | | | | | |  |  |  |
| A | 0021952 | central nervous system projection neuron axonogenesis | EN-124k-90-group14948.gs\_26769 | | | | | | | | | | | | | | |  |  |  |
| A | 0021952 | central nervous system projection neuron axonogenesis | EN-124k-90-group15056.gs\_73097 | | | | | | | | | | | | | | |  |  |  |
| A | 0021952 | central nervous system projection neuron axonogenesis | EN-124k-90-group15198.gs\_65271 | | | | | | | | | | | | | | |  |  |  |
| E | 0021952 | central nervous system projection neuron axonogenesis | EN-124k-90-group15223.EN\_iowa\_18438 | | | | | | | | | | | | | | | |  |  |
| E | 0021952 | central nervous system projection neuron axonogenesis | EN-124k-90-group15290.EN\_iowa\_9903 | | | | | | | | | | | | | | | |  |  |
| E | 0021952 | central nervous system projection neuron axonogenesis | EN-124k-90-group15320.jgi\_paired\_JGI\_CBBP9817\_fwd | | | | | | | | | | | | | | | | | |
| A | 0021952 | central nervous system projection neuron axonogenesis | EN-124k-90-group15399.gs\_80457 | | | | | | | | | | | | | | |  |  |  |
| A | 0021952 | central nervous system projection neuron axonogenesis | EN-124k-90-group15770.gs\_45569 | | | | | | | | | | | | | | |  |  |  |
| E | 0021952 | central nervous system projection neuron axonogenesis | EN-124k-90-group15988.jgi\_unpaired\_JGI\_CBBP2255\_fwd | | | | | | | | | | | | | | | | | |
| A | 0021952 | central nervous system projection neuron axonogenesis | EN-124k-90-group16067.gs\_19008 | | | | | | | | | | | | | | |  |  |  |
| E | 0021952 | central nervous system projection neuron axonogenesis | EN-124k-90-group16200.jgi\_paired\_JGI\_CBBP10444\_rev | | | | | | | | | | | | | | | | | |
| A | 0021952 | central nervous system projection neuron axonogenesis | EN-124k-90-group16254.gs\_29060 | | | | | | | | | | | | | | |  |  |  |
| A | 0021952 | central nervous system projection neuron axonogenesis | EN-124k-90-group16439.gs\_54729 | | | | | | | | | | | | | | |  |  |  |
| A | 0021952 | central nervous system projection neuron axonogenesis | EN-124k-90-group16489.gs\_80464 | | | | | | | | | | | | | | |  |  |  |
| A | 0021952 | central nervous system projection neuron axonogenesis | EN-124k-90-group16600.gs\_29512 | | | | | | | | | | | | | | |  |  |  |
| A | 0021952 | central nervous system projection neuron axonogenesis | EN-124k-90-group16830.gs\_82512 | | | | | | | | | | | | | | |  |  |  |
| A | 0021952 | central nervous system projection neuron axonogenesis | EN-124k-90-group16837.gs\_15971 | | | | | | | | | | | | | | |  |  |  |
| A | 0021952 | central nervous system projection neuron axonogenesis | EN-124k-90-group16872.gs\_213 | | | | | | | | | | | | | | |  |  |  |
| A | 0021952 | central nervous system projection neuron axonogenesis | EN-124k-90-group16929.gs\_43290 | | | | | | | | | | | | | | |  |  |  |
| A | 0021952 | central nervous system projection neuron axonogenesis | EN-124k-90-group17276.gs\_80679 | | | | | | | | | | | | | | |  |  |  |
| A | 0021952 | central nervous system projection neuron axonogenesis | EN-124k-90-group17360.gs\_72469 | | | | | | | | | | | | | | |  |  |  |
| A | 0021952 | central nervous system projection neuron axonogenesis | EN-124k-90-group17487.gs\_82041 | | | | | | | | | | | | | | |  |  |  |
| A | 0021952 | central nervous system projection neuron axonogenesis | EN-124k-90-group17572.gs\_7187 | | | | | | | | | | | | | | |  |  |  |
| A | 0021952 | central nervous system projection neuron axonogenesis | EN-124k-90-group17582.gs\_70636 | | | | | | | | | | | | | | |  |  |  |
| A | 0021952 | central nervous system projection neuron axonogenesis | EN-124k-90-group17706.gs\_80669 | | | | | | | | | | | | | | |  |  |  |
| A | 0021952 | central nervous system projection neuron axonogenesis | EN-124k-90-group17776.gs\_56285 | | | | | | | | | | | | | | |  |  |  |
| A | 0021952 | central nervous system projection neuron axonogenesis | EN-124k-90-group17892.gs\_49260 | | | | | | | | | | | | | | |  |  |  |
| E | 0021952 | central nervous system projection neuron axonogenesis | EN-124k-90-group17979.jgi\_contig\_JGI\_CBBP2421\_fwd | | | | | | | | | | | | | | | | | |
| A | 0021952 | central nervous system projection neuron axonogenesis | EN-124k-90-group18140.gs\_87168 | | | | | | | | | | | | | | |  |  |  |
| E | 0021952 | central nervous system projection neuron axonogenesis | EN-124k-90-group18538.jgi\_paired\_JGI\_CBBP19133\_rev | | | | | | | | | | | | | | | | | |
| A | 0021952 | central nervous system projection neuron axonogenesis | EN-124k-90-group18774.gs\_57540 | | | | | | | | | | | | | | |  |  |  |
| A | 0021952 | central nervous system projection neuron axonogenesis | EN-124k-90-group78.Contig1 | | | | | | | | | | | | | |  |  |  |  |
| A | 0021952 | central nervous system projection neuron axonogenesis | EN-124k-90-group78.Contig2 | | | | | | | | | | | | | |  |  |  |  |
| M | 0021952 | central nervous system projection neuron axonogenesis | EN-124k-90-group230.Contig1 | | | | | | | | | | | | | |  |  |  |  |
| M | 0021952 | central nervous system projection neuron axonogenesis | EN-124k-90-group328.Contig2 | | | | | | | | | | | | | |  |  |  |  |
| M | 0021952 | central nervous system projection neuron axonogenesis | EN-124k-90-group359.Contig1 | | | | | | | | | | | | | |  |  |  |  |
| M | 0021952 | central nervous system projection neuron axonogenesis | EN-124k-90-group359.Contig2 | | | | | | | | | | | | | |  |  |  |  |
| M | 0021952 | central nervous system projection neuron axonogenesis | EN-124k-90-group359.Contig3 | | | | | | | | | | | | | |  |  |  |  |
| M | 0021952 | central nervous system projection neuron axonogenesis | EN-124k-90-group359.Contig4 | | | | | | | | | | | | | |  |  |  |  |
| M | 0021952 | central nervous system projection neuron axonogenesis | EN-124k-90-group359.Contig5 | | | | | | | | | | | | | |  |  |  |  |
| M | 0021952 | central nervous system projection neuron axonogenesis | EN-124k-90-group362.Contig2 | | | | | | | | | | | | | |  |  |  |  |
| M | 0021952 | central nervous system projection neuron axonogenesis | EN-124k-90-group481.Contig4 | | | | | | | | | | | | | |  |  |  |  |
| M | 0021952 | central nervous system projection neuron axonogenesis | EN-124k-90-group518.Contig2 | | | | | | | | | | | | | |  |  |  |  |
| M | 0021952 | central nervous system projection neuron axonogenesis | EN-124k-90-group550.Contig2 | | | | | | | | | | | | | |  |  |  |  |
| M | 0021952 | central nervous system projection neuron axonogenesis | EN-124k-90-group593.Contig1 | | | | | | | | | | | | | |  |  |  |  |
| M | 0021952 | central nervous system projection neuron axonogenesis | EN-124k-90-group630.Contig2 | | | | | | | | | | | | | |  |  |  |  |
| M | 0021952 | central nervous system projection neuron axonogenesis | EN-124k-90-group658.Contig1 | | | | | | | | | | | | | |  |  |  |  |
| M | 0021952 | central nervous system projection neuron axonogenesis | EN-124k-90-group658.Contig3 | | | | | | | | | | | | | |  |  |  |  |
| M | 0021952 | central nervous system projection neuron axonogenesis | EN-124k-90-group682.Contig1 | | | | | | | | | | | | | |  |  |  |  |
| M | 0021952 | central nervous system projection neuron axonogenesis | EN-124k-90-group735.Contig1 | | | | | | | | | | | | | |  |  |  |  |
| M | 0021952 | central nervous system projection neuron axonogenesis | EN-124k-90-group881.Contig1 | | | | | | | | | | | | | |  |  |  |  |
| M | 0021952 | central nervous system projection neuron axonogenesis | EN-124k-90-group881.Contig2 | | | | | | | | | | | | | |  |  |  |  |
| M | 0021952 | central nervous system projection neuron axonogenesis | EN-124k-90-group881.Contig3 | | | | | | | | | | | | | |  |  |  |  |
| M | 0021952 | central nervous system projection neuron axonogenesis | EN-124k-90-group900.Contig1 | | | | | | | | | | | | | |  |  |  |  |
| M | 0021952 | central nervous system projection neuron axonogenesis | EN-124k-90-group1034.Contig1 | | | | | | | | | | | | | |  |  |  |  |
| M | 0021952 | central nervous system projection neuron axonogenesis | EN-124k-90-group1071.Contig1 | | | | | | | | | | | | | |  |  |  |  |
| M | 0021952 | central nervous system projection neuron axonogenesis | EN-124k-90-group1071.Contig2 | | | | | | | | | | | | | |  |  |  |  |
| M | 0021952 | central nervous system projection neuron axonogenesis | EN-124k-90-group1071.Contig3 | | | | | | | | | | | | | |  |  |  |  |
| M | 0021952 | central nervous system projection neuron axonogenesis | EN-124k-90-group1106.Contig1 | | | | | | | | | | | | | |  |  |  |  |
| M | 0021952 | central nervous system projection neuron axonogenesis | EN-124k-90-group1167.Contig2 | | | | | | | | | | | | | |  |  |  |  |
| M | 0021952 | central nervous system projection neuron axonogenesis | EN-124k-90-group1167.Contig3 | | | | | | | | | | | | | |  |  |  |  |
| M | 0021952 | central nervous system projection neuron axonogenesis | EN-124k-90-group1304.Contig2 | | | | | | | | | | | | | |  |  |  |  |
| M | 0021952 | central nervous system projection neuron axonogenesis | EN-124k-90-group1304.Contig4 | | | | | | | | | | | | | |  |  |  |  |
| M | 0021952 | central nervous system projection neuron axonogenesis | EN-124k-90-group1304.Contig8 | | | | | | | | | | | | | |  |  |  |  |
| M | 0021952 | central nervous system projection neuron axonogenesis | EN-124k-90-group1401.Contig2 | | | | | | | | | | | | | |  |  |  |  |
| M | 0021952 | central nervous system projection neuron axonogenesis | EN-124k-90-group1456.Contig4 | | | | | | | | | | | | | |  |  |  |  |
| M | 0021952 | central nervous system projection neuron axonogenesis | EN-124k-90-group1456.Contig8 | | | | | | | | | | | | | |  |  |  |  |
| M | 0021952 | central nervous system projection neuron axonogenesis | EN-124k-90-group1619.Contig1 | | | | | | | | | | | | | |  |  |  |  |
| M | 0021952 | central nervous system projection neuron axonogenesis | EN-124k-90-group1642.Contig1 | | | | | | | | | | | | | |  |  |  |  |
| M | 0021952 | central nervous system projection neuron axonogenesis | EN-124k-90-group1642.Contig2 | | | | | | | | | | | | | |  |  |  |  |
| M | 0021952 | central nervous system projection neuron axonogenesis | EN-124k-90-group1642.Contig6 | | | | | | | | | | | | | |  |  |  |  |
| M | 0021952 | central nervous system projection neuron axonogenesis | EN-124k-90-group1642.Contig7 | | | | | | | | | | | | | |  |  |  |  |
| M | 0021952 | central nervous system projection neuron axonogenesis | EN-124k-90-group1728.Contig2 | | | | | | | | | | | | | |  |  |  |  |
| M | 0021952 | central nervous system projection neuron axonogenesis | EN-124k-90-group1735.Contig4 | | | | | | | | | | | | | |  |  |  |  |
| M | 0021952 | central nervous system projection neuron axonogenesis | EN-124k-90-group1735.Contig6 | | | | | | | | | | | | | |  |  |  |  |
| M | 0021952 | central nervous system projection neuron axonogenesis | EN-124k-90-group1782.Contig1 | | | | | | | | | | | | | |  |  |  |  |
| M | 0021952 | central nervous system projection neuron axonogenesis | EN-124k-90-group1915.Contig1 | | | | | | | | | | | | | |  |  |  |  |
| M | 0021952 | central nervous system projection neuron axonogenesis | EN-124k-90-group1960.Contig1 | | | | | | | | | | | | | |  |  |  |  |
| M | 0021952 | central nervous system projection neuron axonogenesis | EN-124k-90-group2075.Contig2 | | | | | | | | | | | | | |  |  |  |  |
| M | 0021952 | central nervous system projection neuron axonogenesis | EN-124k-90-group2081.Contig1 | | | | | | | | | | | | | |  |  |  |  |
| M | 0021952 | central nervous system projection neuron axonogenesis | EN-124k-90-group2081.Contig3 | | | | | | | | | | | | | |  |  |  |  |
| M | 0021952 | central nervous system projection neuron axonogenesis | EN-124k-90-group2097.Contig1 | | | | | | | | | | | | | |  |  |  |  |
| M | 0021952 | central nervous system projection neuron axonogenesis | EN-124k-90-group2155.Contig1 | | | | | | | | | | | | | |  |  |  |  |
| M | 0021952 | central nervous system projection neuron axonogenesis | EN-124k-90-group2200.Contig1 | | | | | | | | | | | | | |  |  |  |  |
| M | 0021952 | central nervous system projection neuron axonogenesis | EN-124k-90-group2449.Contig1 | | | | | | | | | | | | | |  |  |  |  |
| M | 0021952 | central nervous system projection neuron axonogenesis | EN-124k-90-group2449.Contig2 | | | | | | | | | | | | | |  |  |  |  |
| M | 0021952 | central nervous system projection neuron axonogenesis | EN-124k-90-group2498.Contig5 | | | | | | | | | | | | | |  |  |  |  |
| M | 0021952 | central nervous system projection neuron axonogenesis | EN-124k-90-group2498.Contig7 | | | | | | | | | | | | | |  |  |  |  |
| M | 0021952 | central nervous system projection neuron axonogenesis | EN-124k-90-group2606.Contig1 | | | | | | | | | | | | | |  |  |  |  |
| M | 0021952 | central nervous system projection neuron axonogenesis | EN-124k-90-group2778.Contig1 | | | | | | | | | | | | | |  |  |  |  |
| M | 0021952 | central nervous system projection neuron axonogenesis | EN-124k-90-group2846.Contig1 | | | | | | | | | | | | | |  |  |  |  |
| M | 0021952 | central nervous system projection neuron axonogenesis | EN-124k-90-group2917.Contig1 | | | | | | | | | | | | | |  |  |  |  |
| M | 0021952 | central nervous system projection neuron axonogenesis | EN-124k-90-group2917.Contig2 | | | | | | | | | | | | | |  |  |  |  |
| M | 0021952 | central nervous system projection neuron axonogenesis | EN-124k-90-group2917.Contig3 | | | | | | | | | | | | | |  |  |  |  |
| M | 0021952 | central nervous system projection neuron axonogenesis | EN-124k-90-group2917.Contig4 | | | | | | | | | | | | | |  |  |  |  |
| M | 0021952 | central nervous system projection neuron axonogenesis | EN-124k-90-group3082.Contig1 | | | | | | | | | | | | | |  |  |  |  |
| M | 0021952 | central nervous system projection neuron axonogenesis | EN-124k-90-group3082.Contig2 | | | | | | | | | | | | | |  |  |  |  |
| M | 0021952 | central nervous system projection neuron axonogenesis | EN-124k-90-group3223.Contig2 | | | | | | | | | | | | | |  |  |  |  |
| M | 0021952 | central nervous system projection neuron axonogenesis | EN-124k-90-group3313.Contig3 | | | | | | | | | | | | | |  |  |  |  |
| M | 0021952 | central nervous system projection neuron axonogenesis | EN-124k-90-group3315.Contig2 | | | | | | | | | | | | | |  |  |  |  |
| M | 0021952 | central nervous system projection neuron axonogenesis | EN-124k-90-group3315.Contig5 | | | | | | | | | | | | | |  |  |  |  |
| M | 0021952 | central nervous system projection neuron axonogenesis | EN-124k-90-group3315.Contig6 | | | | | | | | | | | | | |  |  |  |  |
| M | 0021952 | central nervous system projection neuron axonogenesis | EN-124k-90-group3365.Contig1 | | | | | | | | | | | | | |  |  |  |  |
| M | 0021952 | central nervous system projection neuron axonogenesis | EN-124k-90-group3394.Contig1 | | | | | | | | | | | | | |  |  |  |  |
| M | 0021952 | central nervous system projection neuron axonogenesis | EN-124k-90-group3394.Contig2 | | | | | | | | | | | | | |  |  |  |  |
| M | 0021952 | central nervous system projection neuron axonogenesis | EN-124k-90-group3394.Contig3 | | | | | | | | | | | | | |  |  |  |  |
| M | 0021952 | central nervous system projection neuron axonogenesis | EN-124k-90-group3394.Contig4 | | | | | | | | | | | | | |  |  |  |  |
| M | 0021952 | central nervous system projection neuron axonogenesis | EN-124k-90-group3479.Contig1 | | | | | | | | | | | | | |  |  |  |  |
| M | 0021952 | central nervous system projection neuron axonogenesis | EN-124k-90-group3503.Contig2 | | | | | | | | | | | | | |  |  |  |  |
| M | 0021952 | central nervous system projection neuron axonogenesis | EN-124k-90-group3503.Contig5 | | | | | | | | | | | | | |  |  |  |  |
| M | 0021952 | central nervous system projection neuron axonogenesis | EN-124k-90-group3700.Contig1 | | | | | | | | | | | | | |  |  |  |  |
| M | 0021952 | central nervous system projection neuron axonogenesis | EN-124k-90-group3830.Contig1 | | | | | | | | | | | | | |  |  |  |  |
| M | 0021952 | central nervous system projection neuron axonogenesis | EN-124k-90-group3933.Contig1 | | | | | | | | | | | | | |  |  |  |  |
| M | 0021952 | central nervous system projection neuron axonogenesis | EN-124k-90-group3997.Contig2 | | | | | | | | | | | | | |  |  |  |  |
| M | 0021952 | central nervous system projection neuron axonogenesis | EN-124k-90-group4183.Contig1 | | | | | | | | | | | | | |  |  |  |  |
| M | 0021952 | central nervous system projection neuron axonogenesis | EN-124k-90-group4372.Contig1 | | | | | | | | | | | | | |  |  |  |  |
| M | 0021952 | central nervous system projection neuron axonogenesis | EN-124k-90-group4410.Contig1 | | | | | | | | | | | | | |  |  |  |  |
| M | 0021952 | central nervous system projection neuron axonogenesis | EN-124k-90-group4562.Contig1 | | | | | | | | | | | | | |  |  |  |  |
| M | 0021952 | central nervous system projection neuron axonogenesis | EN-124k-90-group4753.Contig1 | | | | | | | | | | | | | |  |  |  |  |
| M | 0021952 | central nervous system projection neuron axonogenesis | EN-124k-90-group4929.Contig2 | | | | | | | | | | | | | |  |  |  |  |
| M | 0021952 | central nervous system projection neuron axonogenesis | EN-124k-90-group5172.Contig1 | | | | | | | | | | | | | |  |  |  |  |
| M | 0021952 | central nervous system projection neuron axonogenesis | EN-124k-90-group5173.Contig1 | | | | | | | | | | | | | |  |  |  |  |
| M | 0021952 | central nervous system projection neuron axonogenesis | EN-124k-90-group5273.Contig3 | | | | | | | | | | | | | |  |  |  |  |
| M | 0021952 | central nervous system projection neuron axonogenesis | EN-124k-90-group5337.Contig1 | | | | | | | | | | | | | |  |  |  |  |
| M | 0021952 | central nervous system projection neuron axonogenesis | EN-124k-90-group5380.Contig1 | | | | | | | | | | | | | |  |  |  |  |
| M | 0021952 | central nervous system projection neuron axonogenesis | EN-124k-90-group5411.Contig1 | | | | | | | | | | | | | |  |  |  |  |
| M | 0021952 | central nervous system projection neuron axonogenesis | EN-124k-90-group5471.Contig3 | | | | | | | | | | | | | |  |  |  |  |
| M | 0021952 | central nervous system projection neuron axonogenesis | EN-124k-90-group5479.Contig1 | | | | | | | | | | | | | |  |  |  |  |
| M | 0021952 | central nervous system projection neuron axonogenesis | EN-124k-90-group5527.Contig2 | | | | | | | | | | | | | |  |  |  |  |
| M | 0021952 | central nervous system projection neuron axonogenesis | EN-124k-90-group5542.Contig1 | | | | | | | | | | | | | |  |  |  |  |
| M | 0021952 | central nervous system projection neuron axonogenesis | EN-124k-90-group5607.Contig1 | | | | | | | | | | | | | |  |  |  |  |
| M | 0021952 | central nervous system projection neuron axonogenesis | EN-124k-90-group5717.Contig2 | | | | | | | | | | | | | |  |  |  |  |
| M | 0021952 | central nervous system projection neuron axonogenesis | EN-124k-90-group5968.Contig1 | | | | | | | | | | | | | |  |  |  |  |
| E | 0021952 | central nervous system projection neuron axonogenesis | EN-124k-90-group6238.Contig1 | | | | | | | | | | | | | |  |  |  |  |
| M | 0021952 | central nervous system projection neuron axonogenesis | EN-124k-90-group6291.Contig2 | | | | | | | | | | | | | |  |  |  |  |
| M | 0021952 | central nervous system projection neuron axonogenesis | EN-124k-90-group6358.Contig1 | | | | | | | | | | | | | |  |  |  |  |
| M | 0021952 | central nervous system projection neuron axonogenesis | EN-124k-90-group6358.Contig2 | | | | | | | | | | | | | |  |  |  |  |
| M | 0021952 | central nervous system projection neuron axonogenesis | EN-124k-90-group6400.Contig1 | | | | | | | | | | | | | |  |  |  |  |
| M | 0021952 | central nervous system projection neuron axonogenesis | EN-124k-90-group6666.Contig1 | | | | | | | | | | | | | |  |  |  |  |
| M | 0021952 | central nervous system projection neuron axonogenesis | EN-124k-90-group6923.Contig1 | | | | | | | | | | | | | |  |  |  |  |
| M | 0021952 | central nervous system projection neuron axonogenesis | EN-124k-90-group6923.Contig2 | | | | | | | | | | | | | |  |  |  |  |
| M | 0021952 | central nervous system projection neuron axonogenesis | EN-124k-90-group7149.Contig2 | | | | | | | | | | | | | |  |  |  |  |
| M | 0021952 | central nervous system projection neuron axonogenesis | EN-124k-90-group7157.Contig2 | | | | | | | | | | | | | |  |  |  |  |
| M | 0021952 | central nervous system projection neuron axonogenesis | EN-124k-90-group7231.Contig1 | | | | | | | | | | | | | |  |  |  |  |
| M | 0021952 | central nervous system projection neuron axonogenesis | EN-124k-90-group7343.Contig1 | | | | | | | | | | | | | |  |  |  |  |
| M | 0021952 | central nervous system projection neuron axonogenesis | EN-124k-90-group7528.Contig1 | | | | | | | | | | | | | |  |  |  |  |
| M | 0021952 | central nervous system projection neuron axonogenesis | EN-124k-90-group7612.Contig2 | | | | | | | | | | | | | |  |  |  |  |
| M | 0021952 | central nervous system projection neuron axonogenesis | EN-124k-90-group7866.Contig1 | | | | | | | | | | | | | |  |  |  |  |
| M | 0021952 | central nervous system projection neuron axonogenesis | EN-124k-90-group8034.Contig1 | | | | | | | | | | | | | |  |  |  |  |
| M | 0021952 | central nervous system projection neuron axonogenesis | EN-124k-90-group8079.Contig1 | | | | | | | | | | | | | |  |  |  |  |
| M | 0021952 | central nervous system projection neuron axonogenesis | EN-124k-90-group8079.Contig2 | | | | | | | | | | | | | |  |  |  |  |
| E | 0021952 | central nervous system projection neuron axonogenesis | EN-124k-90-group8101.Contig1 | | | | | | | | | | | | | |  |  |  |  |
| M | 0021952 | central nervous system projection neuron axonogenesis | EN-124k-90-group8211.Contig1 | | | | | | | | | | | | | |  |  |  |  |
| M | 0021952 | central nervous system projection neuron axonogenesis | EN-124k-90-group8297.Contig1 | | | | | | | | | | | | | |  |  |  |  |
| M | 0021952 | central nervous system projection neuron axonogenesis | EN-124k-90-group8375.Contig1 | | | | | | | | | | | | | |  |  |  |  |
| A | 0021952 | central nervous system projection neuron axonogenesis | EN-124k-90-group8565.Contig1 | | | | | | | | | | | | | |  |  |  |  |
| M | 0021952 | central nervous system projection neuron axonogenesis | EN-124k-90-group8799.Contig2 | | | | | | | | | | | | | |  |  |  |  |
| M | 0021952 | central nervous system projection neuron axonogenesis | EN-124k-90-group8937.Contig1 | | | | | | | | | | | | | |  |  |  |  |
| M | 0021952 | central nervous system projection neuron axonogenesis | EN-124k-90-group8937.Contig2 | | | | | | | | | | | | | |  |  |  |  |
| M | 0021952 | central nervous system projection neuron axonogenesis | EN-124k-90-group9278.Contig2 | | | | | | | | | | | | | |  |  |  |  |
| M | 0021952 | central nervous system projection neuron axonogenesis | EN-124k-90-group9295.Contig1 | | | | | | | | | | | | | |  |  |  |  |
| A | 0021952 | central nervous system projection neuron axonogenesis | EN-124k-90-group9295.Contig2 | | | | | | | | | | | | | |  |  |  |  |
| M | 0021952 | central nervous system projection neuron axonogenesis | EN-124k-90-group9299.Contig3 | | | | | | | | | | | | | |  |  |  |  |
| M | 0021952 | central nervous system projection neuron axonogenesis | EN-124k-90-group9317.Contig1 | | | | | | | | | | | | | |  |  |  |  |
| M | 0021952 | central nervous system projection neuron axonogenesis | EN-124k-90-group9446.Contig1 | | | | | | | | | | | | | |  |  |  |  |
| M | 0021952 | central nervous system projection neuron axonogenesis | EN-124k-90-group9620.Contig1 | | | | | | | | | | | | | |  |  |  |  |
| M | 0021952 | central nervous system projection neuron axonogenesis | EN-124k-90-group9712.Contig1 | | | | | | | | | | | | | |  |  |  |  |
| M | 0021952 | central nervous system projection neuron axonogenesis | EN-124k-90-group9747.Contig1 | | | | | | | | | | | | | |  |  |  |  |
| M | 0021952 | central nervous system projection neuron axonogenesis | EN-124k-90-group9747.Contig2 | | | | | | | | | | | | | |  |  |  |  |
| M | 0021952 | central nervous system projection neuron axonogenesis | EN-124k-90-group9819.Contig1 | | | | | | | | | | | | | |  |  |  |  |
| M | 0021952 | central nervous system projection neuron axonogenesis | EN-124k-90-group10221.Contig1 | | | | | | | | | | | | | | |  |  |  |
| M | 0021952 | central nervous system projection neuron axonogenesis | EN-124k-90-group10598.Contig1 | | | | | | | | | | | | | | |  |  |  |
| M | 0021952 | central nervous system projection neuron axonogenesis | EN-124k-90-group10630.Contig1 | | | | | | | | | | | | | | |  |  |  |
| M | 0021952 | central nervous system projection neuron axonogenesis | EN-124k-90-group10630.Contig2 | | | | | | | | | | | | | | |  |  |  |
| M | 0021952 | central nervous system projection neuron axonogenesis | EN-124k-90-group10712.Contig1 | | | | | | | | | | | | | | |  |  |  |
| M | 0021952 | central nervous system projection neuron axonogenesis | EN-124k-90-group10838.Contig2 | | | | | | | | | | | | | | |  |  |  |
| M | 0021952 | central nervous system projection neuron axonogenesis | EN-124k-90-group10877.Contig1 | | | | | | | | | | | | | | |  |  |  |
| M | 0021952 | central nervous system projection neuron axonogenesis | EN-124k-90-group10912.Contig1 | | | | | | | | | | | | | | |  |  |  |
| M | 0021952 | central nervous system projection neuron axonogenesis | EN-124k-90-group10928.Contig1 | | | | | | | | | | | | | | |  |  |  |
| M | 0021952 | central nervous system projection neuron axonogenesis | EN-124k-90-group10950.Contig1 | | | | | | | | | | | | | | |  |  |  |
| M | 0021952 | central nervous system projection neuron axonogenesis | EN-124k-90-group11134.Contig1 | | | | | | | | | | | | | | |  |  |  |
| M | 0021952 | central nervous system projection neuron axonogenesis | EN-124k-90-group11228.Contig1 | | | | | | | | | | | | | | |  |  |  |
| M | 0021952 | central nervous system projection neuron axonogenesis | EN-124k-90-group11235.Contig1 | | | | | | | | | | | | | | |  |  |  |
| M | 0021952 | central nervous system projection neuron axonogenesis | EN-124k-90-group11399.Contig2 | | | | | | | | | | | | | | |  |  |  |
| M | 0021952 | central nervous system projection neuron axonogenesis | EN-124k-90-group11408.Contig1 | | | | | | | | | | | | | | |  |  |  |
| M | 0021952 | central nervous system projection neuron axonogenesis | EN-124k-90-group11516.Contig1 | | | | | | | | | | | | | | |  |  |  |
| M | 0021952 | central nervous system projection neuron axonogenesis | EN-124k-90-group11516.Contig2 | | | | | | | | | | | | | | |  |  |  |
| M | 0021952 | central nervous system projection neuron axonogenesis | EN-124k-90-group11686.Contig1 | | | | | | | | | | | | | | |  |  |  |
| M | 0021952 | central nervous system projection neuron axonogenesis | EN-124k-90-group11808.Contig1 | | | | | | | | | | | | | | |  |  |  |
| M | 0021952 | central nervous system projection neuron axonogenesis | EN-124k-90-group12099.Contig1 | | | | | | | | | | | | | | |  |  |  |
| M | 0021952 | central nervous system projection neuron axonogenesis | EN-124k-90-group12143.Contig1 | | | | | | | | | | | | | | |  |  |  |
| M | 0021952 | central nervous system projection neuron axonogenesis | EN-124k-90-group12519.Contig1 | | | | | | | | | | | | | | |  |  |  |
| E | 0021952 | central nervous system projection neuron axonogenesis | EN-124k-90-group13069.Contig2 | | | | | | | | | | | | | | |  |  |  |
| M | 0021952 | central nervous system projection neuron axonogenesis | EN-124k-90-group13235.Contig1 | | | | | | | | | | | | | | |  |  |  |
| M | 0021952 | central nervous system projection neuron axonogenesis | EN-124k-90-group14134.Contig1 | | | | | | | | | | | | | | |  |  |  |
| A | 0021952 | central nervous system projection neuron axonogenesis | EN-124k-90-group14199.Contig1 | | | | | | | | | | | | | | |  |  |  |
| M | 0021952 | central nervous system projection neuron axonogenesis | EN-124k-90-group14708.Contig1 | | | | | | | | | | | | | | |  |  |  |
| M | 0021952 | central nervous system projection neuron axonogenesis | EN-124k-90-group14975.Contig1 | | | | | | | | | | | | | | |  |  |  |
| M | 0021952 | central nervous system projection neuron axonogenesis | EN-124k-90-group14975.Contig2 | | | | | | | | | | | | | | |  |  |  |
| A | 0021952 | central nervous system projection neuron axonogenesis | EN-124k-90-group230.gs\_25885 | | | | | | | | | | | | | | |  |  |  |
| E | 0021952 | central nervous system projection neuron axonogenesis | EN-124k-90-group359.jgi\_contig\_JGI\_CBBP16242\_fwd | | | | | | | | | | | | | | | | | |
| E | 0021952 | central nervous system projection neuron axonogenesis | EN-124k-90-group359.EN\_iowa\_14996 | | | | | | | | | | | | | | |  |  |  |
| E | 0021952 | central nervous system projection neuron axonogenesis | EN-124k-90-group359.EN\_iowa\_1876 | | | | | | | | | | | | | | |  |  |  |
| E | 0021952 | central nervous system projection neuron axonogenesis | EN-124k-90-group359.EN\_iowa\_13183 | | | | | | | | | | | | | | |  |  |  |
| A | 0021952 | central nervous system projection neuron axonogenesis | EN-124k-90-group359.gs\_11387 | | | | | | | | | | | | | | |  |  |  |
| A | 0021952 | central nervous system projection neuron axonogenesis | EN-124k-90-group359.gs\_45042 | | | | | | | | | | | | | | |  |  |  |
| A | 0021952 | central nervous system projection neuron axonogenesis | EN-124k-90-group359.gs\_60113 | | | | | | | | | | | | | | |  |  |  |
| A | 0021952 | central nervous system projection neuron axonogenesis | EN-124k-90-group735.gs\_7849 | | | | | | | | | | | | | |  |  |  |  |
| E | 0021952 | central nervous system projection neuron axonogenesis | EN-124k-90-group900.EN\_iowa\_15436 | | | | | | | | | | | | | | |  |  |  |
| A | 0021952 | central nervous system projection neuron axonogenesis | EN-124k-90-group900.gs\_31783 | | | | | | | | | | | | | | |  |  |  |
| A | 0021952 | central nervous system projection neuron axonogenesis | EN-124k-90-group900.gs\_71447 | | | | | | | | | | | | | | |  |  |  |
| A | 0021952 | central nervous system projection neuron axonogenesis | EN-124k-90-group1642.gs\_68829 | | | | | | | | | | | | | | |  |  |  |
| A | 0021952 | central nervous system projection neuron axonogenesis | EN-124k-90-group1642.gs\_49728 | | | | | | | | | | | | | | |  |  |  |
| E | 0021952 | central nervous system projection neuron axonogenesis | EN-124k-90-group1642.jgi\_contig\_JGI\_CBBP16332\_fwd | | | | | | | | | | | | | | | | | |
| A | 0021952 | central nervous system projection neuron axonogenesis | EN-124k-90-group1642.gs\_63459 | | | | | | | | | | | | | | |  |  |  |
| E | 0021952 | central nervous system projection neuron axonogenesis | EN-124k-90-group1642.jgi\_contig\_JGI\_CBBP16017\_fwd | | | | | | | | | | | | | | | | | |
| A | 0021952 | central nervous system projection neuron axonogenesis | EN-124k-90-group1642.gs\_32797 | | | | | | | | | | | | | | |  |  |  |
| E | 0021952 | central nervous system projection neuron axonogenesis | EN-124k-90-group1642.EN\_iowa\_1848 | | | | | | | | | | | | | | |  |  |  |
| A | 0021952 | central nervous system projection neuron axonogenesis | EN-124k-90-group1782.gs\_16663 | | | | | | | | | | | | | | |  |  |  |
| A | 0021952 | central nervous system projection neuron axonogenesis | EN-124k-90-group2449.gs\_35369 | | | | | | | | | | | | | | |  |  |  |
| E | 0021952 | central nervous system projection neuron axonogenesis | EN-124k-90-group2778.jgi\_contig\_JGI\_CBBP17773\_fwd | | | | | | | | | | | | | | | | | |
| E | 0021952 | central nervous system projection neuron axonogenesis | EN-124k-90-group5607.jgi\_contig\_JGI\_CBBP445\_fwd | | | | | | | | | | | | | | | | | |
| E | 0021952 | central nervous system projection neuron axonogenesis | EN-124k-90-group6923.jgi\_contig\_JGI\_CBBP17789\_fwd | | | | | | | | | | | | | | | | | |
| A | 0021952 | central nervous system projection neuron axonogenesis | EN-124k-90-group9299.gs\_87064 | | | | | | | | | | | | | | |  |  |  |
| A | 0045200 | establishment of neuroblast polarity | EN-124k-90-group807.gs\_71281 | | | | | | | | | | | | | | |  |  |  |
| E | 0045200 | establishment of neuroblast polarity | EN-124k-90-group1306.EN\_iowa\_2354 | | | | | | | | | | | | | | |  |  |  |
| E | 0045200 | establishment of neuroblast polarity | EN-124k-90-group3448.jgi\_paired\_JGI\_CBBP19729\_fwd | | | | | | | | | | | | | | | | | |
| E | 0045200 | establishment of neuroblast polarity | EN-124k-90-group4248.jgi\_contig\_JGI\_CBBP19155\_fwd | | | | | | | | | | | | | | | | | |
| E | 0045200 | establishment of neuroblast polarity | EN-124k-90-group5834.jgi\_contig\_JGI\_CBBP12701\_fwd | | | | | | | | | | | | | | | | | |
| E | 0045200 | establishment of neuroblast polarity | EN-124k-90-group6737.jgi\_contig\_JGI\_CBBP17820\_fwd | | | | | | | | | | | | | | | | | |
| E | 0045200 | establishment of neuroblast polarity | EN-124k-90-group7523.jgi\_paired\_JGI\_CBBP3627\_rev | | | | | | | | | | | | | | | | | |
| E | 0045200 | establishment of neuroblast polarity | EN-124k-90-group7867.EN\_iowa\_18229 | | | | | | | | | | | | | | | |  |  |
| E | 0045200 | establishment of neuroblast polarity | EN-124k-90-group8370.jgi\_paired\_JGI\_CBBP17511\_fwd | | | | | | | | | | | | | | | | | |
| E | 0045200 | establishment of neuroblast polarity | EN-124k-90-group9263.jgi\_paired\_JGI\_CBBP3642\_fwd | | | | | | | | | | | | | | | | | |
| A | 0045200 | establishment of neuroblast polarity | EN-124k-90-group9467.gs\_16039 | | | | | | | | | | | | | | |  |  |  |
| A | 0045200 | establishment of neuroblast polarity | EN-124k-90-group9714.gs\_64750 | | | | | | | | | | | | | | |  |  |  |
| E | 0045200 | establishment of neuroblast polarity | EN-124k-90-group9839.EN\_iowa\_2226 | | | | | | | | | | | | | | |  |  |  |
| A | 0045200 | establishment of neuroblast polarity | EN-124k-90-group10312.gs\_22624 | | | | | | | | | | | | | | |  |  |  |
| E | 0045200 | establishment of neuroblast polarity | EN-124k-90-group10968.EN\_iowa\_5471 | | | | | | | | | | | | | | | |  |  |
| E | 0045200 | establishment of neuroblast polarity | EN-124k-90-group11559.jgi\_unpaired\_JGI\_CBBP12231\_fwd | | | | | | | | | | | | | | | | | |
| A | 0045200 | establishment of neuroblast polarity | EN-124k-90-group11738.gs\_53113 | | | | | | | | | | | | | | |  |  |  |
| E | 0045200 | establishment of neuroblast polarity | EN-124k-90-group12012.jgi\_contig\_JGI\_CBBP10480\_fwd | | | | | | | | | | | | | | | | | |
| E | 0045200 | establishment of neuroblast polarity | EN-124k-90-group12454.jgi\_paired\_JGI\_CBBP19087\_fwd | | | | | | | | | | | | | | | | | |
| E | 0045200 | establishment of neuroblast polarity | EN-124k-90-group12676.jgi\_paired\_JGI\_CBBP17511\_rev | | | | | | | | | | | | | | | | | |
| E | 0045200 | establishment of neuroblast polarity | EN-124k-90-group12744.EN\_iowa\_4256 | | | | | | | | | | | | | | | |  |  |
| A | 0045200 | establishment of neuroblast polarity | EN-124k-90-group12952.gs\_44961 | | | | | | | | | | | | | | |  |  |  |
| A | 0045200 | establishment of neuroblast polarity | EN-124k-90-group13391.gs\_76600 | | | | | | | | | | | | | | |  |  |  |
| A | 0045200 | establishment of neuroblast polarity | EN-124k-90-group13542.gs\_42987 | | | | | | | | | | | | | | |  |  |  |
| E | 0045200 | establishment of neuroblast polarity | EN-124k-90-group13717.jgi\_contig\_JGI\_CBBP5296\_fwd | | | | | | | | | | | | | | | | | |
| E | 0045200 | establishment of neuroblast polarity | EN-124k-90-group13906.jgi\_paired\_JGI\_CBBP12965\_fwd | | | | | | | | | | | | | | | | | |
| E | 0045200 | establishment of neuroblast polarity | EN-124k-90-group13916.EN\_iowa\_14815 | | | | | | | | | | | | | | | |  |  |
| E | 0045200 | establishment of neuroblast polarity | EN-124k-90-group14114.jgi\_paired\_JGI\_CBBP9776\_fwd | | | | | | | | | | | | | | | | | |
| E | 0045200 | establishment of neuroblast polarity | EN-124k-90-group14311.jgi\_paired\_JGI\_CBBP682\_fwd | | | | | | | | | | | | | | | | | |
| A | 0045200 | establishment of neuroblast polarity | EN-124k-90-group14320.gs\_86221 | | | | | | | | | | | | | | |  |  |  |
| E | 0045200 | establishment of neuroblast polarity | EN-124k-90-group14509.jgi\_paired\_JGI\_CBBP933\_fwd | | | | | | | | | | | | | | | | | |
| A | 0045200 | establishment of neuroblast polarity | EN-124k-90-group14513.gs\_43210 | | | | | | | | | | | | | | |  |  |  |
| A | 0045200 | establishment of neuroblast polarity | EN-124k-90-group14563.gs\_48486 | | | | | | | | | | | | | | |  |  |  |
| E | 0045200 | establishment of neuroblast polarity | EN-124k-90-group14573.jgi\_paired\_JGI\_CBBP20040\_fwd | | | | | | | | | | | | | | | | | |
| A | 0045200 | establishment of neuroblast polarity | EN-124k-90-group14609.gs\_33389 | | | | | | | | | | | | | | |  |  |  |
| A | 0045200 | establishment of neuroblast polarity | EN-124k-90-group14948.gs\_26769 | | | | | | | | | | | | | | |  |  |  |
| A | 0045200 | establishment of neuroblast polarity | EN-124k-90-group15056.gs\_73097 | | | | | | | | | | | | | | |  |  |  |
| E | 0045200 | establishment of neuroblast polarity | EN-124k-90-group15254.jgi\_paired\_JGI\_CBBP18230\_fwd | | | | | | | | | | | | | | | | | |
| E | 0045200 | establishment of neuroblast polarity | EN-124k-90-group15320.jgi\_paired\_JGI\_CBBP9817\_fwd | | | | | | | | | | | | | | | | | |
| E | 0045200 | establishment of neuroblast polarity | EN-124k-90-group15546.EN\_iowa\_13502 | | | | | | | | | | | | | | | |  |  |
| E | 0045200 | establishment of neuroblast polarity | EN-124k-90-group15570.jgi\_paired\_JGI\_CBBP13286\_rev | | | | | | | | | | | | | | | | | |
| A | 0045200 | establishment of neuroblast polarity | EN-124k-90-group15770.gs\_45569 | | | | | | | | | | | | | | |  |  |  |
| E | 0045200 | establishment of neuroblast polarity | EN-124k-90-group15776.EN\_iowa\_1971 | | | | | | | | | | | | | | | |  |  |
| A | 0045200 | establishment of neuroblast polarity | EN-124k-90-group16285.gs\_47129 | | | | | | | | | | | | | | |  |  |  |
| A | 0045200 | establishment of neuroblast polarity | EN-124k-90-group16416.gs\_28586 | | | | | | | | | | | | | | |  |  |  |
| A | 0045200 | establishment of neuroblast polarity | EN-124k-90-group17360.gs\_72469 | | | | | | | | | | | | | | |  |  |  |
| A | 0045200 | establishment of neuroblast polarity | EN-124k-90-group17386.gs\_68411 | | | | | | | | | | | | | | |  |  |  |
| A | 0045200 | establishment of neuroblast polarity | EN-124k-90-group17442.gs\_63748 | | | | | | | | | | | | | | |  |  |  |
| A | 0045200 | establishment of neuroblast polarity | EN-124k-90-group17524.gs\_80802 | | | | | | | | | | | | | | |  |  |  |
| E | 0045200 | establishment of neuroblast polarity | EN-124k-90-group17979.jgi\_contig\_JGI\_CBBP2421\_fwd | | | | | | | | | | | | | | | | | |
| E | 0045200 | establishment of neuroblast polarity | EN-124k-90-group18538.jgi\_paired\_JGI\_CBBP19133\_rev | | | | | | | | | | | | | | | | | |
| A | 0045200 | establishment of neuroblast polarity | EN-124k-90-group18929.gs\_13816 | | | | | | | | | | | | | | |  |  |  |
| M | 0045200 | establishment of neuroblast polarity | EN-124k-90-group10.Contig1 | | | | | | | | | | | | | |  |  |  |  |
| M | 0045200 | establishment of neuroblast polarity | EN-124k-90-group124.Contig1 | | | | | | | | | | | | | |  |  |  |  |
| M | 0045200 | establishment of neuroblast polarity | EN-124k-90-group150.Contig1 | | | | | | | | | | | | | |  |  |  |  |
| M | 0045200 | establishment of neuroblast polarity | EN-124k-90-group229.Contig1 | | | | | | | | | | | | | |  |  |  |  |
| M | 0045200 | establishment of neuroblast polarity | EN-124k-90-group230.Contig1 | | | | | | | | | | | | | |  |  |  |  |
| M | 0045200 | establishment of neuroblast polarity | EN-124k-90-group462.Contig1 | | | | | | | | | | | | | |  |  |  |  |
| M | 0045200 | establishment of neuroblast polarity | EN-124k-90-group518.Contig2 | | | | | | | | | | | | | |  |  |  |  |
| M | 0045200 | establishment of neuroblast polarity | EN-124k-90-group543.Contig1 | | | | | | | | | | | | | |  |  |  |  |
| M | 0045200 | establishment of neuroblast polarity | EN-124k-90-group550.Contig2 | | | | | | | | | | | | | |  |  |  |  |
| M | 0045200 | establishment of neuroblast polarity | EN-124k-90-group630.Contig2 | | | | | | | | | | | | | |  |  |  |  |
| M | 0045200 | establishment of neuroblast polarity | EN-124k-90-group716.Contig1 | | | | | | | | | | | | | |  |  |  |  |
| M | 0045200 | establishment of neuroblast polarity | EN-124k-90-group735.Contig1 | | | | | | | | | | | | | |  |  |  |  |
| M | 0045200 | establishment of neuroblast polarity | EN-124k-90-group760.Contig4 | | | | | | | | | | | | | |  |  |  |  |
| M | 0045200 | establishment of neuroblast polarity | EN-124k-90-group784.Contig3 | | | | | | | | | | | | | |  |  |  |  |
| M | 0045200 | establishment of neuroblast polarity | EN-124k-90-group849.Contig2 | | | | | | | | | | | | | |  |  |  |  |
| M | 0045200 | establishment of neuroblast polarity | EN-124k-90-group849.Contig3 | | | | | | | | | | | | | |  |  |  |  |
| M | 0045200 | establishment of neuroblast polarity | EN-124k-90-group881.Contig3 | | | | | | | | | | | | | |  |  |  |  |
| M | 0045200 | establishment of neuroblast polarity | EN-124k-90-group1034.Contig1 | | | | | | | | | | | | | |  |  |  |  |
| M | 0045200 | establishment of neuroblast polarity | EN-124k-90-group1106.Contig1 | | | | | | | | | | | | | |  |  |  |  |
| M | 0045200 | establishment of neuroblast polarity | EN-124k-90-group1302.Contig2 | | | | | | | | | | | | | |  |  |  |  |
| M | 0045200 | establishment of neuroblast polarity | EN-124k-90-group1302.Contig3 | | | | | | | | | | | | | |  |  |  |  |
| M | 0045200 | establishment of neuroblast polarity | EN-124k-90-group1302.Contig4 | | | | | | | | | | | | | |  |  |  |  |
| E | 0045200 | establishment of neuroblast polarity | EN-124k-90-group1344.Contig1 | | | | | | | | | | | | | |  |  |  |  |
| M | 0045200 | establishment of neuroblast polarity | EN-124k-90-group1422.Contig1 | | | | | | | | | | | | | |  |  |  |  |
| M | 0045200 | establishment of neuroblast polarity | EN-124k-90-group1554.Contig1 | | | | | | | | | | | | | |  |  |  |  |
| M | 0045200 | establishment of neuroblast polarity | EN-124k-90-group1699.Contig1 | | | | | | | | | | | | | |  |  |  |  |
| M | 0045200 | establishment of neuroblast polarity | EN-124k-90-group1699.Contig3 | | | | | | | | | | | | | |  |  |  |  |
| M | 0045200 | establishment of neuroblast polarity | EN-124k-90-group1726.Contig2 | | | | | | | | | | | | | |  |  |  |  |
| M | 0045200 | establishment of neuroblast polarity | EN-124k-90-group1735.Contig6 | | | | | | | | | | | | | |  |  |  |  |
| M | 0045200 | establishment of neuroblast polarity | EN-124k-90-group1757.Contig1 | | | | | | | | | | | | | |  |  |  |  |
| M | 0045200 | establishment of neuroblast polarity | EN-124k-90-group1782.Contig1 | | | | | | | | | | | | | |  |  |  |  |
| M | 0045200 | establishment of neuroblast polarity | EN-124k-90-group1933.Contig1 | | | | | | | | | | | | | |  |  |  |  |
| M | 0045200 | establishment of neuroblast polarity | EN-124k-90-group1933.Contig2 | | | | | | | | | | | | | |  |  |  |  |
| M | 0045200 | establishment of neuroblast polarity | EN-124k-90-group1960.Contig1 | | | | | | | | | | | | | |  |  |  |  |
| M | 0045200 | establishment of neuroblast polarity | EN-124k-90-group2077.Contig1 | | | | | | | | | | | | | |  |  |  |  |
| M | 0045200 | establishment of neuroblast polarity | EN-124k-90-group2077.Contig3 | | | | | | | | | | | | | |  |  |  |  |
| M | 0045200 | establishment of neuroblast polarity | EN-124k-90-group2081.Contig1 | | | | | | | | | | | | | |  |  |  |  |
| M | 0045200 | establishment of neuroblast polarity | EN-124k-90-group2081.Contig3 | | | | | | | | | | | | | |  |  |  |  |
| M | 0045200 | establishment of neuroblast polarity | EN-124k-90-group2097.Contig1 | | | | | | | | | | | | | |  |  |  |  |
| M | 0045200 | establishment of neuroblast polarity | EN-124k-90-group2145.Contig1 | | | | | | | | | | | | | |  |  |  |  |
| M | 0045200 | establishment of neuroblast polarity | EN-124k-90-group2200.Contig1 | | | | | | | | | | | | | |  |  |  |  |
| M | 0045200 | establishment of neuroblast polarity | EN-124k-90-group2264.Contig2 | | | | | | | | | | | | | |  |  |  |  |
| M | 0045200 | establishment of neuroblast polarity | EN-124k-90-group2264.Contig3 | | | | | | | | | | | | | |  |  |  |  |
| M | 0045200 | establishment of neuroblast polarity | EN-124k-90-group2491.Contig1 | | | | | | | | | | | | | |  |  |  |  |
| M | 0045200 | establishment of neuroblast polarity | EN-124k-90-group2525.Contig3 | | | | | | | | | | | | | |  |  |  |  |
| M | 0045200 | establishment of neuroblast polarity | EN-124k-90-group2525.Contig4 | | | | | | | | | | | | | |  |  |  |  |
| M | 0045200 | establishment of neuroblast polarity | EN-124k-90-group2600.Contig1 | | | | | | | | | | | | | |  |  |  |  |
| M | 0045200 | establishment of neuroblast polarity | EN-124k-90-group2600.Contig2 | | | | | | | | | | | | | |  |  |  |  |
| M | 0045200 | establishment of neuroblast polarity | EN-124k-90-group2642.Contig6 | | | | | | | | | | | | | |  |  |  |  |
| M | 0045200 | establishment of neuroblast polarity | EN-124k-90-group2846.Contig1 | | | | | | | | | | | | | |  |  |  |  |
| M | 0045200 | establishment of neuroblast polarity | EN-124k-90-group2917.Contig1 | | | | | | | | | | | | | |  |  |  |  |
| M | 0045200 | establishment of neuroblast polarity | EN-124k-90-group2917.Contig2 | | | | | | | | | | | | | |  |  |  |  |
| M | 0045200 | establishment of neuroblast polarity | EN-124k-90-group2917.Contig3 | | | | | | | | | | | | | |  |  |  |  |
| M | 0045200 | establishment of neuroblast polarity | EN-124k-90-group2917.Contig4 | | | | | | | | | | | | | |  |  |  |  |
| M | 0045200 | establishment of neuroblast polarity | EN-124k-90-group2975.Contig1 | | | | | | | | | | | | | |  |  |  |  |
| M | 0045200 | establishment of neuroblast polarity | EN-124k-90-group3037.Contig2 | | | | | | | | | | | | | |  |  |  |  |
| M | 0045200 | establishment of neuroblast polarity | EN-124k-90-group3082.Contig1 | | | | | | | | | | | | | |  |  |  |  |
| M | 0045200 | establishment of neuroblast polarity | EN-124k-90-group3082.Contig2 | | | | | | | | | | | | | |  |  |  |  |
| M | 0045200 | establishment of neuroblast polarity | EN-124k-90-group3157.Contig5 | | | | | | | | | | | | | |  |  |  |  |
| M | 0045200 | establishment of neuroblast polarity | EN-124k-90-group3158.Contig2 | | | | | | | | | | | | | |  |  |  |  |
| M | 0045200 | establishment of neuroblast polarity | EN-124k-90-group3223.Contig2 | | | | | | | | | | | | | |  |  |  |  |
| M | 0045200 | establishment of neuroblast polarity | EN-124k-90-group3228.Contig2 | | | | | | | | | | | | | |  |  |  |  |
| M | 0045200 | establishment of neuroblast polarity | EN-124k-90-group3245.Contig2 | | | | | | | | | | | | | |  |  |  |  |
| M | 0045200 | establishment of neuroblast polarity | EN-124k-90-group3313.Contig3 | | | | | | | | | | | | | |  |  |  |  |
| M | 0045200 | establishment of neuroblast polarity | EN-124k-90-group3338.Contig1 | | | | | | | | | | | | | |  |  |  |  |
| M | 0045200 | establishment of neuroblast polarity | EN-124k-90-group3357.Contig1 | | | | | | | | | | | | | |  |  |  |  |
| E | 0045200 | establishment of neuroblast polarity | EN-124k-90-group3377.Contig1 | | | | | | | | | | | | | |  |  |  |  |
| M | 0045200 | establishment of neuroblast polarity | EN-124k-90-group3394.Contig1 | | | | | | | | | | | | | |  |  |  |  |
| M | 0045200 | establishment of neuroblast polarity | EN-124k-90-group3394.Contig2 | | | | | | | | | | | | | |  |  |  |  |
| M | 0045200 | establishment of neuroblast polarity | EN-124k-90-group3394.Contig3 | | | | | | | | | | | | | |  |  |  |  |
| M | 0045200 | establishment of neuroblast polarity | EN-124k-90-group3394.Contig4 | | | | | | | | | | | | | |  |  |  |  |
| M | 0045200 | establishment of neuroblast polarity | EN-124k-90-group3479.Contig1 | | | | | | | | | | | | | |  |  |  |  |
| E | 0045200 | establishment of neuroblast polarity | EN-124k-90-group3518.Contig1 | | | | | | | | | | | | | |  |  |  |  |
| M | 0045200 | establishment of neuroblast polarity | EN-124k-90-group3537.Contig1 | | | | | | | | | | | | | |  |  |  |  |
| M | 0045200 | establishment of neuroblast polarity | EN-124k-90-group3700.Contig1 | | | | | | | | | | | | | |  |  |  |  |
| M | 0045200 | establishment of neuroblast polarity | EN-124k-90-group3849.Contig3 | | | | | | | | | | | | | |  |  |  |  |
| M | 0045200 | establishment of neuroblast polarity | EN-124k-90-group3933.Contig1 | | | | | | | | | | | | | |  |  |  |  |
| M | 0045200 | establishment of neuroblast polarity | EN-124k-90-group4049.Contig1 | | | | | | | | | | | | | |  |  |  |  |
| M | 0045200 | establishment of neuroblast polarity | EN-124k-90-group4168.Contig1 | | | | | | | | | | | | | |  |  |  |  |
| M | 0045200 | establishment of neuroblast polarity | EN-124k-90-group4168.Contig3 | | | | | | | | | | | | | |  |  |  |  |
| M | 0045200 | establishment of neuroblast polarity | EN-124k-90-group4183.Contig1 | | | | | | | | | | | | | |  |  |  |  |
| M | 0045200 | establishment of neuroblast polarity | EN-124k-90-group4290.Contig3 | | | | | | | | | | | | | |  |  |  |  |
| M | 0045200 | establishment of neuroblast polarity | EN-124k-90-group4350.Contig2 | | | | | | | | | | | | | |  |  |  |  |
| M | 0045200 | establishment of neuroblast polarity | EN-124k-90-group4562.Contig1 | | | | | | | | | | | | | |  |  |  |  |
| M | 0045200 | establishment of neuroblast polarity | EN-124k-90-group4715.Contig3 | | | | | | | | | | | | | |  |  |  |  |
| M | 0045200 | establishment of neuroblast polarity | EN-124k-90-group4716.Contig1 | | | | | | | | | | | | | |  |  |  |  |
| M | 0045200 | establishment of neuroblast polarity | EN-124k-90-group4753.Contig1 | | | | | | | | | | | | | |  |  |  |  |
| A | 0045200 | establishment of neuroblast polarity | EN-124k-90-group4925.Contig1 | | | | | | | | | | | | | |  |  |  |  |
| M | 0045200 | establishment of neuroblast polarity | EN-124k-90-group4929.Contig2 | | | | | | | | | | | | | |  |  |  |  |
| M | 0045200 | establishment of neuroblast polarity | EN-124k-90-group4944.Contig2 | | | | | | | | | | | | | |  |  |  |  |
| M | 0045200 | establishment of neuroblast polarity | EN-124k-90-group5051.Contig2 | | | | | | | | | | | | | |  |  |  |  |
| M | 0045200 | establishment of neuroblast polarity | EN-124k-90-group5071.Contig1 | | | | | | | | | | | | | |  |  |  |  |
| M | 0045200 | establishment of neuroblast polarity | EN-124k-90-group5071.Contig2 | | | | | | | | | | | | | |  |  |  |  |
| M | 0045200 | establishment of neuroblast polarity | EN-124k-90-group5071.Contig3 | | | | | | | | | | | | | |  |  |  |  |
| M | 0045200 | establishment of neuroblast polarity | EN-124k-90-group5172.Contig1 | | | | | | | | | | | | | |  |  |  |  |
| M | 0045200 | establishment of neuroblast polarity | EN-124k-90-group5173.Contig1 | | | | | | | | | | | | | |  |  |  |  |
| M | 0045200 | establishment of neuroblast polarity | EN-124k-90-group5273.Contig1 | | | | | | | | | | | | | |  |  |  |  |
| M | 0045200 | establishment of neuroblast polarity | EN-124k-90-group5273.Contig3 | | | | | | | | | | | | | |  |  |  |  |
| M | 0045200 | establishment of neuroblast polarity | EN-124k-90-group5380.Contig1 | | | | | | | | | | | | | |  |  |  |  |
| M | 0045200 | establishment of neuroblast polarity | EN-124k-90-group5607.Contig1 | | | | | | | | | | | | | |  |  |  |  |
| M | 0045200 | establishment of neuroblast polarity | EN-124k-90-group5616.Contig1 | | | | | | | | | | | | | |  |  |  |  |
| M | 0045200 | establishment of neuroblast polarity | EN-124k-90-group5633.Contig1 | | | | | | | | | | | | | |  |  |  |  |
| M | 0045200 | establishment of neuroblast polarity | EN-124k-90-group5710.Contig2 | | | | | | | | | | | | | |  |  |  |  |
| M | 0045200 | establishment of neuroblast polarity | EN-124k-90-group5729.Contig1 | | | | | | | | | | | | | |  |  |  |  |
| M | 0045200 | establishment of neuroblast polarity | EN-124k-90-group5910.Contig1 | | | | | | | | | | | | | |  |  |  |  |
| M | 0045200 | establishment of neuroblast polarity | EN-124k-90-group6038.Contig1 | | | | | | | | | | | | | |  |  |  |  |
| M | 0045200 | establishment of neuroblast polarity | EN-124k-90-group6195.Contig1 | | | | | | | | | | | | | |  |  |  |  |
| M | 0045200 | establishment of neuroblast polarity | EN-124k-90-group6195.Contig2 | | | | | | | | | | | | | |  |  |  |  |
| M | 0045200 | establishment of neuroblast polarity | EN-124k-90-group6358.Contig1 | | | | | | | | | | | | | |  |  |  |  |
| M | 0045200 | establishment of neuroblast polarity | EN-124k-90-group6358.Contig2 | | | | | | | | | | | | | |  |  |  |  |
| M | 0045200 | establishment of neuroblast polarity | EN-124k-90-group6614.Contig1 | | | | | | | | | | | | | |  |  |  |  |
| M | 0045200 | establishment of neuroblast polarity | EN-124k-90-group6883.Contig1 | | | | | | | | | | | | | |  |  |  |  |
| M | 0045200 | establishment of neuroblast polarity | EN-124k-90-group6883.Contig2 | | | | | | | | | | | | | |  |  |  |  |
| M | 0045200 | establishment of neuroblast polarity | EN-124k-90-group6923.Contig1 | | | | | | | | | | | | | |  |  |  |  |
| M | 0045200 | establishment of neuroblast polarity | EN-124k-90-group6923.Contig2 | | | | | | | | | | | | | |  |  |  |  |
| M | 0045200 | establishment of neuroblast polarity | EN-124k-90-group7149.Contig2 | | | | | | | | | | | | | |  |  |  |  |
| M | 0045200 | establishment of neuroblast polarity | EN-124k-90-group7157.Contig1 | | | | | | | | | | | | | |  |  |  |  |
| M | 0045200 | establishment of neuroblast polarity | EN-124k-90-group7157.Contig2 | | | | | | | | | | | | | |  |  |  |  |
| M | 0045200 | establishment of neuroblast polarity | EN-124k-90-group7158.Contig1 | | | | | | | | | | | | | |  |  |  |  |
| M | 0045200 | establishment of neuroblast polarity | EN-124k-90-group7231.Contig1 | | | | | | | | | | | | | |  |  |  |  |
| M | 0045200 | establishment of neuroblast polarity | EN-124k-90-group7343.Contig1 | | | | | | | | | | | | | |  |  |  |  |
| M | 0045200 | establishment of neuroblast polarity | EN-124k-90-group7528.Contig1 | | | | | | | | | | | | | |  |  |  |  |
| M | 0045200 | establishment of neuroblast polarity | EN-124k-90-group7883.Contig1 | | | | | | | | | | | | | |  |  |  |  |
| M | 0045200 | establishment of neuroblast polarity | EN-124k-90-group7964.Contig1 | | | | | | | | | | | | | |  |  |  |  |
| M | 0045200 | establishment of neuroblast polarity | EN-124k-90-group8079.Contig1 | | | | | | | | | | | | | |  |  |  |  |
| M | 0045200 | establishment of neuroblast polarity | EN-124k-90-group8079.Contig2 | | | | | | | | | | | | | |  |  |  |  |
| E | 0045200 | establishment of neuroblast polarity | EN-124k-90-group8101.Contig1 | | | | | | | | | | | | | |  |  |  |  |
| M | 0045200 | establishment of neuroblast polarity | EN-124k-90-group8193.Contig1 | | | | | | | | | | | | | |  |  |  |  |
| M | 0045200 | establishment of neuroblast polarity | EN-124k-90-group8297.Contig1 | | | | | | | | | | | | | |  |  |  |  |
| M | 0045200 | establishment of neuroblast polarity | EN-124k-90-group8589.Contig2 | | | | | | | | | | | | | |  |  |  |  |
| M | 0045200 | establishment of neuroblast polarity | EN-124k-90-group8937.Contig1 | | | | | | | | | | | | | |  |  |  |  |
| M | 0045200 | establishment of neuroblast polarity | EN-124k-90-group8937.Contig2 | | | | | | | | | | | | | |  |  |  |  |
| M | 0045200 | establishment of neuroblast polarity | EN-124k-90-group9170.Contig1 | | | | | | | | | | | | | |  |  |  |  |
| M | 0045200 | establishment of neuroblast polarity | EN-124k-90-group9278.Contig2 | | | | | | | | | | | | | |  |  |  |  |
| M | 0045200 | establishment of neuroblast polarity | EN-124k-90-group9317.Contig1 | | | | | | | | | | | | | |  |  |  |  |
| M | 0045200 | establishment of neuroblast polarity | EN-124k-90-group9446.Contig1 | | | | | | | | | | | | | |  |  |  |  |
| M | 0045200 | establishment of neuroblast polarity | EN-124k-90-group9479.Contig1 | | | | | | | | | | | | | |  |  |  |  |
| M | 0045200 | establishment of neuroblast polarity | EN-124k-90-group9541.Contig1 | | | | | | | | | | | | | |  |  |  |  |
| M | 0045200 | establishment of neuroblast polarity | EN-124k-90-group10450.Contig1 | | | | | | | | | | | | | | |  |  |  |
| M | 0045200 | establishment of neuroblast polarity | EN-124k-90-group10598.Contig1 | | | | | | | | | | | | | | |  |  |  |
| M | 0045200 | establishment of neuroblast polarity | EN-124k-90-group10630.Contig1 | | | | | | | | | | | | | | |  |  |  |
| M | 0045200 | establishment of neuroblast polarity | EN-124k-90-group10630.Contig2 | | | | | | | | | | | | | | |  |  |  |
| E | 0045200 | establishment of neuroblast polarity | EN-124k-90-group10742.Contig1 | | | | | | | | | | | | | | |  |  |  |
| M | 0045200 | establishment of neuroblast polarity | EN-124k-90-group10823.Contig1 | | | | | | | | | | | | | | |  |  |  |
| M | 0045200 | establishment of neuroblast polarity | EN-124k-90-group10838.Contig2 | | | | | | | | | | | | | | |  |  |  |
| M | 0045200 | establishment of neuroblast polarity | EN-124k-90-group10881.Contig1 | | | | | | | | | | | | | | |  |  |  |
| M | 0045200 | establishment of neuroblast polarity | EN-124k-90-group11134.Contig1 | | | | | | | | | | | | | | |  |  |  |
| M | 0045200 | establishment of neuroblast polarity | EN-124k-90-group11408.Contig1 | | | | | | | | | | | | | | |  |  |  |
| M | 0045200 | establishment of neuroblast polarity | EN-124k-90-group12099.Contig1 | | | | | | | | | | | | | | |  |  |  |
| M | 0045200 | establishment of neuroblast polarity | EN-124k-90-group12143.Contig1 | | | | | | | | | | | | | | |  |  |  |
| M | 0045200 | establishment of neuroblast polarity | EN-124k-90-group12337.Contig2 | | | | | | | | | | | | | | |  |  |  |
| E | 0045200 | establishment of neuroblast polarity | EN-124k-90-group12653.Contig1 | | | | | | | | | | | | | | |  |  |  |
| M | 0045200 | establishment of neuroblast polarity | EN-124k-90-group12960.Contig1 | | | | | | | | | | | | | | |  |  |  |
| E | 0045200 | establishment of neuroblast polarity | EN-124k-90-group13342.Contig1 | | | | | | | | | | | | | | |  |  |  |
| A | 0045200 | establishment of neuroblast polarity | EN-124k-90-group65.gs\_37667 | | | | | | | | | | | | | |  |  |  |  |
| A | 0045200 | establishment of neuroblast polarity | EN-124k-90-group230.gs\_25885 | | | | | | | | | | | | | | |  |  |  |
| A | 0045200 | establishment of neuroblast polarity | EN-124k-90-group487.gs\_45542 | | | | | | | | | | | | | | |  |  |  |
| A | 0045200 | establishment of neuroblast polarity | EN-124k-90-group487.gs\_72303 | | | | | | | | | | | | | | |  |  |  |
| A | 0045200 | establishment of neuroblast polarity | EN-124k-90-group487.gs\_40039 | | | | | | | | | | | | | | |  |  |  |
| A | 0045200 | establishment of neuroblast polarity | EN-124k-90-group735.gs\_7849 | | | | | | | | | | | | | |  |  |  |  |
| E | 0045200 | establishment of neuroblast polarity | EN-124k-90-group835.EN\_iowa\_1516 | | | | | | | | | | | | | | |  |  |  |
| E | 0045200 | establishment of neuroblast polarity | EN-124k-90-group1554.jgi\_contig\_JGI\_CBBP18583\_fwd | | | | | | | | | | | | | | | | | |
| E | 0045200 | establishment of neuroblast polarity | EN-124k-90-group1699.jgi\_contig\_JGI\_CBBP7344\_fwd | | | | | | | | | | | | | | | | | |
| A | 0045200 | establishment of neuroblast polarity | EN-124k-90-group1699.gs\_33563 | | | | | | | | | | | | | | |  |  |  |
| A | 0045200 | establishment of neuroblast polarity | EN-124k-90-group1782.gs\_16663 | | | | | | | | | | | | | | |  |  |  |
| E | 0045200 | establishment of neuroblast polarity | EN-124k-90-group2077.jgi\_paired\_JGI\_CBBP13793\_rev | | | | | | | | | | | | | | | | | |
| E | 0045200 | establishment of neuroblast polarity | EN-124k-90-group2525.jgi\_contig\_JGI\_CBBP2745\_fwd | | | | | | | | | | | | | | | | | |
| E | 0045200 | establishment of neuroblast polarity | EN-124k-90-group2525.jgi\_contig\_JGI\_CBBP12002\_fwd | | | | | | | | | | | | | | | | | |
| A | 0045200 | establishment of neuroblast polarity | EN-124k-90-group2525.gs\_26865 | | | | | | | | | | | | | | |  |  |  |
| A | 0045200 | establishment of neuroblast polarity | EN-124k-90-group2525.gs\_28080 | | | | | | | | | | | | | | |  |  |  |
| E | 0045200 | establishment of neuroblast polarity | EN-124k-90-group2525.jgi\_contig\_JGI\_CBBP7217\_fwd | | | | | | | | | | | | | | | | | |
| E | 0045200 | establishment of neuroblast polarity | EN-124k-90-group2600.jgi\_contig\_JGI\_CBBP7087\_fwd | | | | | | | | | | | | | | | | | |
| E | 0045200 | establishment of neuroblast polarity | EN-124k-90-group2600.jgi\_contig\_JGI\_CBBP2795\_fwd | | | | | | | | | | | | | | | | | |
| E | 0045200 | establishment of neuroblast polarity | EN-124k-90-group2600.EN\_iowa\_8689 | | | | | | | | | | | | | | |  |  |  |
| A | 0045200 | establishment of neuroblast polarity | EN-124k-90-group2642.gs\_85577 | | | | | | | | | | | | | | |  |  |  |
| E | 0045200 | establishment of neuroblast polarity | EN-124k-90-group3537.jgi\_paired\_JGI\_CBBP755\_rev | | | | | | | | | | | | | | | | | |
| E | 0045200 | establishment of neuroblast polarity | EN-124k-90-group4049.EN\_iowa\_15523 | | | | | | | | | | | | | | | |  |  |
| A | 0045200 | establishment of neuroblast polarity | EN-124k-90-group5300.gs\_35256 | | | | | | | | | | | | | | |  |  |  |
| E | 0045200 | establishment of neuroblast polarity | EN-124k-90-group5607.jgi\_contig\_JGI\_CBBP445\_fwd | | | | | | | | | | | | | | | | | |
| A | 0045200 | establishment of neuroblast polarity | EN-124k-90-group6883.gs\_22524 | | | | | | | | | | | | | | |  |  |  |
| E | 0045200 | establishment of neuroblast polarity | EN-124k-90-group6923.jgi\_contig\_JGI\_CBBP17789\_fwd | | | | | | | | | | | | | | | | | |
| E | 0045200 | establishment of neuroblast polarity | EN-124k-90-group12653.EN\_iowa\_13251 | | | | | | | | | | | | | | | |  |  |
| A | 0010001 | glial cell differentiation | EN-124k-90-group807.gs\_71281 | | | | | | | | | | | | | | |  |  |  |
| E | 0010001 | glial cell differentiation | EN-124k-90-group1177.jgi\_unpaired\_JGI\_CBBP6661\_fwd | | | | | | | | | | | | | | | | | |
| E | 0010001 | glial cell differentiation | EN-124k-90-group1306.EN\_iowa\_2354 | | | | | | | | | | | | | | |  |  |  |
| E | 0010001 | glial cell differentiation | EN-124k-90-group2106.jgi\_paired\_JGI\_CBBP3443\_fwd | | | | | | | | | | | | | | | | | |
| E | 0010001 | glial cell differentiation | EN-124k-90-group2530.jgi\_paired\_JGI\_CBBP5537\_fwd | | | | | | | | | | | | | | | | | |
| E | 0010001 | glial cell differentiation | EN-124k-90-group2729.EN\_iowa\_12495 | | | | | | | | | | | | | | | |  |  |
| E | 0010001 | glial cell differentiation | EN-124k-90-group3265.jgi\_paired\_JGI\_CBBP17018\_rev | | | | | | | | | | | | | | | | | |
| E | 0010001 | glial cell differentiation | EN-124k-90-group3448.jgi\_paired\_JGI\_CBBP19729\_fwd | | | | | | | | | | | | | | | | | |
| A | 0010001 | glial cell differentiation | EN-124k-90-group3564.gs\_15152 | | | | | | | | | | | | | | |  |  |  |
| E | 0010001 | glial cell differentiation | EN-124k-90-group5117.jgi\_paired\_JGI\_CBBP19153\_fwd | | | | | | | | | | | | | | | | | |
| A | 0010001 | glial cell differentiation | EN-124k-90-group5346.gs\_72008 | | | | | | | | | | | | | | |  |  |  |
| E | 0010001 | glial cell differentiation | EN-124k-90-group5834.jgi\_contig\_JGI\_CBBP12701\_fwd | | | | | | | | | | | | | | | | | |
| E | 0010001 | glial cell differentiation | EN-124k-90-group6737.jgi\_contig\_JGI\_CBBP17820\_fwd | | | | | | | | | | | | | | | | | |
| A | 0010001 | glial cell differentiation | EN-124k-90-group7595.gs\_36261 | | | | | | | | | | | | | | |  |  |  |
| A | 0010001 | glial cell differentiation | EN-124k-90-group8144.gs\_73566 | | | | | | | | | | | | | | |  |  |  |
| A | 0010001 | glial cell differentiation | EN-124k-90-group8310.gs\_24913 | | | | | | | | | | | | | | |  |  |  |
| A | 0010001 | glial cell differentiation | EN-124k-90-group8567.gs\_80390 | | | | | | | | | | | | | | |  |  |  |
| E | 0010001 | glial cell differentiation | EN-124k-90-group8721.jgi\_paired\_JGI\_CBBP11443\_rev | | | | | | | | | | | | | | | | | |
| E | 0010001 | glial cell differentiation | EN-124k-90-group9064.EN\_iowa\_5147 | | | | | | | | | | | | | | |  |  |  |
| E | 0010001 | glial cell differentiation | EN-124k-90-group9268.jgi\_paired\_JGI\_CBBP20285\_fwd | | | | | | | | | | | | | | | | | |
| E | 0010001 | glial cell differentiation | EN-124k-90-group9286.jgi\_paired\_JGI\_CBBP4063\_fwd | | | | | | | | | | | | | | | | | |
| A | 0010001 | glial cell differentiation | EN-124k-90-group9442.gs\_10851 | | | | | | | | | | | | | | |  |  |  |
| A | 0010001 | glial cell differentiation | EN-124k-90-group9467.gs\_16039 | | | | | | | | | | | | | | |  |  |  |
| A | 0010001 | glial cell differentiation | EN-124k-90-group9629.gs\_48808 | | | | | | | | | | | | | | |  |  |  |
| E | 0010001 | glial cell differentiation | EN-124k-90-group9773.jgi\_paired\_JGI\_CBBP6585\_rev | | | | | | | | | | | | | | | | | |
| A | 0010001 | glial cell differentiation | EN-124k-90-group9985.gs\_74394 | | | | | | | | | | | | | | |  |  |  |
| E | 0010001 | glial cell differentiation | EN-124k-90-group10277.jgi\_paired\_JGI\_CBBP15154\_rev | | | | | | | | | | | | | | | | | |
| A | 0010001 | glial cell differentiation | EN-124k-90-group10312.gs\_22624 | | | | | | | | | | | | | | |  |  |  |
| E | 0010001 | glial cell differentiation | EN-124k-90-group10416.jgi\_contig\_JGI\_CBBP3274\_fwd | | | | | | | | | | | | | | | | | |
| E | 0010001 | glial cell differentiation | EN-124k-90-group10538.jgi\_contig\_JGI\_CBBP12785\_fwd | | | | | | | | | | | | | | | | | |
| A | 0010001 | glial cell differentiation | EN-124k-90-group10718.gs\_26673 | | | | | | | | | | | | | | |  |  |  |
| A | 0010001 | glial cell differentiation | EN-124k-90-group10935.gs\_64995 | | | | | | | | | | | | | | |  |  |  |
| A | 0010001 | glial cell differentiation | EN-124k-90-group11738.gs\_53113 | | | | | | | | | | | | | | |  |  |  |
| E | 0010001 | glial cell differentiation | EN-124k-90-group11814.jgi\_paired\_JGI\_CBBP14600\_fwd | | | | | | | | | | | | | | | | | |
| E | 0010001 | glial cell differentiation | EN-124k-90-group11917.jgi\_paired\_JGI\_CBBP3416\_fwd | | | | | | | | | | | | | | | | | |
| E | 0010001 | glial cell differentiation | EN-124k-90-group12012.jgi\_contig\_JGI\_CBBP10480\_fwd | | | | | | | | | | | | | | | | | |
| E | 0010001 | glial cell differentiation | EN-124k-90-group12191.jgi\_paired\_JGI\_CBBP10887\_fwd | | | | | | | | | | | | | | | | | |
| E | 0010001 | glial cell differentiation | EN-124k-90-group12452.EN\_iowa\_9722 | | | | | | | | | | | | | | | |  |  |
| E | 0010001 | glial cell differentiation | EN-124k-90-group12477.jgi\_paired\_JGI\_CBBP10794\_fwd | | | | | | | | | | | | | | | | | |
| A | 0010001 | glial cell differentiation | EN-124k-90-group13006.gs\_33814 | | | | | | | | | | | | | | |  |  |  |
| E | 0010001 | glial cell differentiation | EN-124k-90-group13141.jgi\_paired\_JGI\_CBBP2801\_fwd | | | | | | | | | | | | | | | | | |
| E | 0010001 | glial cell differentiation | EN-124k-90-group13146.jgi\_paired\_JGI\_CBBP17160\_fwd | | | | | | | | | | | | | | | | | |
| E | 0010001 | glial cell differentiation | EN-124k-90-group13346.jgi\_unpaired\_JGI\_CBBP5460\_rev | | | | | | | | | | | | | | | | | |
| E | 0010001 | glial cell differentiation | EN-124k-90-group13384.jgi\_contig\_JGI\_CBBP18717\_fwd | | | | | | | | | | | | | | | | | |
| A | 0010001 | glial cell differentiation | EN-124k-90-group13390.gs\_82388 | | | | | | | | | | | | | | |  |  |  |
| A | 0010001 | glial cell differentiation | EN-124k-90-group13771.gs\_28668 | | | | | | | | | | | | | | |  |  |  |
| A | 0010001 | glial cell differentiation | EN-124k-90-group14128.gs\_38861 | | | | | | | | | | | | | | |  |  |  |
| A | 0010001 | glial cell differentiation | EN-124k-90-group14563.gs\_48486 | | | | | | | | | | | | | | |  |  |  |
| A | 0010001 | glial cell differentiation | EN-124k-90-group14887.gs\_61232 | | | | | | | | | | | | | | |  |  |  |
| A | 0010001 | glial cell differentiation | EN-124k-90-group14948.gs\_26769 | | | | | | | | | | | | | | |  |  |  |
| E | 0010001 | glial cell differentiation | EN-124k-90-group15320.jgi\_paired\_JGI\_CBBP9817\_fwd | | | | | | | | | | | | | | | | | |
| E | 0010001 | glial cell differentiation | EN-124k-90-group15378.jgi\_contig\_JGI\_CBBP10913\_fwd | | | | | | | | | | | | | | | | | |
| A | 0010001 | glial cell differentiation | EN-124k-90-group15397.gs\_22489 | | | | | | | | | | | | | | |  |  |  |
| A | 0010001 | glial cell differentiation | EN-124k-90-group15442.gs\_55801 | | | | | | | | | | | | | | |  |  |  |
| A | 0010001 | glial cell differentiation | EN-124k-90-group15458.gs\_14761 | | | | | | | | | | | | | | |  |  |  |
| E | 0010001 | glial cell differentiation | EN-124k-90-group15532.EN\_iowa\_5883 | | | | | | | | | | | | | | | |  |  |
| E | 0010001 | glial cell differentiation | EN-124k-90-group15588.jgi\_paired\_JGI\_CBBP15154\_fwd | | | | | | | | | | | | | | | | | |
| E | 0010001 | glial cell differentiation | EN-124k-90-group15723.jgi\_paired\_JGI\_CBBP16981\_fwd | | | | | | | | | | | | | | | | | |
| A | 0010001 | glial cell differentiation | EN-124k-90-group15749.gs\_84109 | | | | | | | | | | | | | | |  |  |  |
| A | 0010001 | glial cell differentiation | EN-124k-90-group15770.gs\_45569 | | | | | | | | | | | | | | |  |  |  |
| E | 0010001 | glial cell differentiation | EN-124k-90-group15877.EN\_iowa\_3358 | | | | | | | | | | | | | | | |  |  |
| E | 0010001 | glial cell differentiation | EN-124k-90-group15927.EN\_iowa\_7602 | | | | | | | | | | | | | | | |  |  |
| E | 0010001 | glial cell differentiation | EN-124k-90-group15940.jgi\_contig\_JGI\_CBBP11433\_fwd | | | | | | | | | | | | | | | | | |
| E | 0010001 | glial cell differentiation | EN-124k-90-group15944.jgi\_paired\_JGI\_CBBP5778\_fwd | | | | | | | | | | | | | | | | | |
| A | 0010001 | glial cell differentiation | EN-124k-90-group15950.gs\_36858 | | | | | | | | | | | | | | |  |  |  |
| E | 0010001 | glial cell differentiation | EN-124k-90-group15994.jgi\_paired\_JGI\_CBBP14428\_fwd | | | | | | | | | | | | | | | | | |
| A | 0010001 | glial cell differentiation | EN-124k-90-group16026.gs\_15943 | | | | | | | | | | | | | | |  |  |  |
| E | 0010001 | glial cell differentiation | EN-124k-90-group16134.jgi\_contig\_JGI\_CBBP14519\_fwd | | | | | | | | | | | | | | | | | |
| A | 0010001 | glial cell differentiation | EN-124k-90-group16149.gs\_13439 | | | | | | | | | | | | | | |  |  |  |
| E | 0010001 | glial cell differentiation | EN-124k-90-group16200.jgi\_paired\_JGI\_CBBP10444\_rev | | | | | | | | | | | | | | | | | |
| A | 0010001 | glial cell differentiation | EN-124k-90-group16291.gs\_65588 | | | | | | | | | | | | | | |  |  |  |
| A | 0010001 | glial cell differentiation | EN-124k-90-group16323.gs\_48303 | | | | | | | | | | | | | | |  |  |  |
| A | 0010001 | glial cell differentiation | EN-124k-90-group16412.gs\_64548 | | | | | | | | | | | | | | |  |  |  |
| A | 0010001 | glial cell differentiation | EN-124k-90-group16489.gs\_80464 | | | | | | | | | | | | | | |  |  |  |
| A | 0010001 | glial cell differentiation | EN-124k-90-group16520.gs\_62867 | | | | | | | | | | | | | | |  |  |  |
| A | 0010001 | glial cell differentiation | EN-124k-90-group16522.gs\_78634 | | | | | | | | | | | | | | |  |  |  |
| A | 0010001 | glial cell differentiation | EN-124k-90-group16523.gs\_75670 | | | | | | | | | | | | | | |  |  |  |
| A | 0010001 | glial cell differentiation | EN-124k-90-group16598.gs\_3233 | | | | | | | | | | | | | | |  |  |  |
| A | 0010001 | glial cell differentiation | EN-124k-90-group16640.gs\_7574 | | | | | | | | | | | | | | |  |  |  |
| A | 0010001 | glial cell differentiation | EN-124k-90-group16830.gs\_82512 | | | | | | | | | | | | | | |  |  |  |
| A | 0010001 | glial cell differentiation | EN-124k-90-group16845.gs\_50498 | | | | | | | | | | | | | | |  |  |  |
| A | 0010001 | glial cell differentiation | EN-124k-90-group16851.gs\_64557 | | | | | | | | | | | | | | |  |  |  |
| A | 0010001 | glial cell differentiation | EN-124k-90-group16852.gs\_27372 | | | | | | | | | | | | | | |  |  |  |
| A | 0010001 | glial cell differentiation | EN-124k-90-group16910.gs\_22871 | | | | | | | | | | | | | | |  |  |  |
| A | 0010001 | glial cell differentiation | EN-124k-90-group16929.gs\_43290 | | | | | | | | | | | | | | |  |  |  |
| A | 0010001 | glial cell differentiation | EN-124k-90-group17012.gs\_52263 | | | | | | | | | | | | | | |  |  |  |
| A | 0010001 | glial cell differentiation | EN-124k-90-group17022.gs\_80073 | | | | | | | | | | | | | | |  |  |  |
| A | 0010001 | glial cell differentiation | EN-124k-90-group17117.gs\_84807 | | | | | | | | | | | | | | |  |  |  |
| A | 0010001 | glial cell differentiation | EN-124k-90-group17129.gs\_61289 | | | | | | | | | | | | | | |  |  |  |
| A | 0010001 | glial cell differentiation | EN-124k-90-group17140.gs\_14565 | | | | | | | | | | | | | | |  |  |  |
| A | 0010001 | glial cell differentiation | EN-124k-90-group17193.gs\_13341 | | | | | | | | | | | | | | |  |  |  |
| A | 0010001 | glial cell differentiation | EN-124k-90-group17210.gs\_64350 | | | | | | | | | | | | | | |  |  |  |
| A | 0010001 | glial cell differentiation | EN-124k-90-group17273.gs\_68617 | | | | | | | | | | | | | | |  |  |  |
| A | 0010001 | glial cell differentiation | EN-124k-90-group17301.gs\_75053 | | | | | | | | | | | | | | |  |  |  |
| A | 0010001 | glial cell differentiation | EN-124k-90-group17321.gs\_60379 | | | | | | | | | | | | | | |  |  |  |
| A | 0010001 | glial cell differentiation | EN-124k-90-group17338.gs\_21850 | | | | | | | | | | | | | | |  |  |  |
| A | 0010001 | glial cell differentiation | EN-124k-90-group17386.gs\_68411 | | | | | | | | | | | | | | |  |  |  |
| A | 0010001 | glial cell differentiation | EN-124k-90-group17411.gs\_13940 | | | | | | | | | | | | | | |  |  |  |
| A | 0010001 | glial cell differentiation | EN-124k-90-group17445.gs\_83255 | | | | | | | | | | | | | | |  |  |  |
| A | 0010001 | glial cell differentiation | EN-124k-90-group17471.gs\_85794 | | | | | | | | | | | | | | |  |  |  |
| A | 0010001 | glial cell differentiation | EN-124k-90-group17487.gs\_82041 | | | | | | | | | | | | | | |  |  |  |
| A | 0010001 | glial cell differentiation | EN-124k-90-group17524.gs\_80802 | | | | | | | | | | | | | | |  |  |  |
| A | 0010001 | glial cell differentiation | EN-124k-90-group17556.gs\_87114 | | | | | | | | | | | | | | |  |  |  |
| A | 0010001 | glial cell differentiation | EN-124k-90-group17567.gs\_58610 | | | | | | | | | | | | | | |  |  |  |
| A | 0010001 | glial cell differentiation | EN-124k-90-group17582.gs\_70636 | | | | | | | | | | | | | | |  |  |  |
| A | 0010001 | glial cell differentiation | EN-124k-90-group17702.gs\_18827 | | | | | | | | | | | | | | |  |  |  |
| A | 0010001 | glial cell differentiation | EN-124k-90-group17743.gs\_53689 | | | | | | | | | | | | | | |  |  |  |
| A | 0010001 | glial cell differentiation | EN-124k-90-group17819.gs\_51352 | | | | | | | | | | | | | | |  |  |  |
| A | 0010001 | glial cell differentiation | EN-124k-90-group17864.gs\_35131 | | | | | | | | | | | | | | |  |  |  |
| A | 0010001 | glial cell differentiation | EN-124k-90-group17887.gs\_80072 | | | | | | | | | | | | | | |  |  |  |
| E | 0010001 | glial cell differentiation | EN-124k-90-group17943.EN\_iowa\_18374 | | | | | | | | | | | | | | | |  |  |
| A | 0010001 | glial cell differentiation | EN-124k-90-group17951.gs\_43129 | | | | | | | | | | | | | | |  |  |  |
| E | 0010001 | glial cell differentiation | EN-124k-90-group17979.jgi\_contig\_JGI\_CBBP2421\_fwd | | | | | | | | | | | | | | | | | |
| A | 0010001 | glial cell differentiation | EN-124k-90-group18006.gs\_18909 | | | | | | | | | | | | | | |  |  |  |
| A | 0010001 | glial cell differentiation | EN-124k-90-group18140.gs\_87168 | | | | | | | | | | | | | | |  |  |  |
| A | 0010001 | glial cell differentiation | EN-124k-90-group18238.gs\_39782 | | | | | | | | | | | | | | |  |  |  |
| A | 0010001 | glial cell differentiation | EN-124k-90-group18245.gs\_78006 | | | | | | | | | | | | | | |  |  |  |
| A | 0010001 | glial cell differentiation | EN-124k-90-group18289.gs\_45417 | | | | | | | | | | | | | | |  |  |  |
| A | 0010001 | glial cell differentiation | EN-124k-90-group18324.gs\_57299 | | | | | | | | | | | | | | |  |  |  |
| A | 0010001 | glial cell differentiation | EN-124k-90-group18342.gs\_65612 | | | | | | | | | | | | | | |  |  |  |
| A | 0010001 | glial cell differentiation | EN-124k-90-group18372.gs\_75667 | | | | | | | | | | | | | | |  |  |  |
| A | 0010001 | glial cell differentiation | EN-124k-90-group18447.gs\_40187 | | | | | | | | | | | | | | |  |  |  |
| A | 0010001 | glial cell differentiation | EN-124k-90-group18526.gs\_58285 | | | | | | | | | | | | | | |  |  |  |
| E | 0010001 | glial cell differentiation | EN-124k-90-group18538.jgi\_paired\_JGI\_CBBP19133\_rev | | | | | | | | | | | | | | | | | |
| A | 0010001 | glial cell differentiation | EN-124k-90-group18592.gs\_31083 | | | | | | | | | | | | | | |  |  |  |
| A | 0010001 | glial cell differentiation | EN-124k-90-group18634.gs\_80302 | | | | | | | | | | | | | | |  |  |  |
| A | 0010001 | glial cell differentiation | EN-124k-90-group18774.gs\_57540 | | | | | | | | | | | | | | |  |  |  |
| A | 0010001 | glial cell differentiation | EN-124k-90-group18840.gs\_32731 | | | | | | | | | | | | | | |  |  |  |
| A | 0010001 | glial cell differentiation | EN-124k-90-group18887.gs\_36301 | | | | | | | | | | | | | | |  |  |  |
| A | 0010001 | glial cell differentiation | EN-124k-90-group18929.gs\_13816 | | | | | | | | | | | | | | |  |  |  |
| M | 0010001 | glial cell differentiation | EN-124k-90-group63.Contig2 | | | | | | | | | | | | | |  |  |  |  |
| M | 0010001 | glial cell differentiation | EN-124k-90-group75.Contig2 | | | | | | | | | | | | | |  |  |  |  |
| M | 0010001 | glial cell differentiation | EN-124k-90-group138.Contig1 | | | | | | | | | | | | | |  |  |  |  |
| M | 0010001 | glial cell differentiation | EN-124k-90-group138.Contig62 | | | | | | | | | | | | | |  |  |  |  |
| M | 0010001 | glial cell differentiation | EN-124k-90-group171.Contig2 | | | | | | | | | | | | | |  |  |  |  |
| M | 0010001 | glial cell differentiation | EN-124k-90-group230.Contig1 | | | | | | | | | | | | | |  |  |  |  |
| M | 0010001 | glial cell differentiation | EN-124k-90-group505.Contig1 | | | | | | | | | | | | | |  |  |  |  |
| M | 0010001 | glial cell differentiation | EN-124k-90-group516.Contig2 | | | | | | | | | | | | | |  |  |  |  |
| M | 0010001 | glial cell differentiation | EN-124k-90-group518.Contig2 | | | | | | | | | | | | | |  |  |  |  |
| M | 0010001 | glial cell differentiation | EN-124k-90-group550.Contig2 | | | | | | | | | | | | | |  |  |  |  |
| M | 0010001 | glial cell differentiation | EN-124k-90-group630.Contig2 | | | | | | | | | | | | | |  |  |  |  |
| M | 0010001 | glial cell differentiation | EN-124k-90-group682.Contig1 | | | | | | | | | | | | | |  |  |  |  |
| M | 0010001 | glial cell differentiation | EN-124k-90-group716.Contig1 | | | | | | | | | | | | | |  |  |  |  |
| M | 0010001 | glial cell differentiation | EN-124k-90-group735.Contig1 | | | | | | | | | | | | | |  |  |  |  |
| M | 0010001 | glial cell differentiation | EN-124k-90-group881.Contig2 | | | | | | | | | | | | | |  |  |  |  |
| M | 0010001 | glial cell differentiation | EN-124k-90-group881.Contig3 | | | | | | | | | | | | | |  |  |  |  |
| M | 0010001 | glial cell differentiation | EN-124k-90-group900.Contig1 | | | | | | | | | | | | | |  |  |  |  |
| M | 0010001 | glial cell differentiation | EN-124k-90-group945.Contig1 | | | | | | | | | | | | | |  |  |  |  |
| M | 0010001 | glial cell differentiation | EN-124k-90-group998.Contig1 | | | | | | | | | | | | | |  |  |  |  |
| M | 0010001 | glial cell differentiation | EN-124k-90-group1007.Contig1 | | | | | | | | | | | | | |  |  |  |  |
| E | 0010001 | glial cell differentiation | EN-124k-90-group1092.Contig1 | | | | | | | | | | | | | |  |  |  |  |
| M | 0010001 | glial cell differentiation | EN-124k-90-group1106.Contig1 | | | | | | | | | | | | | |  |  |  |  |
| M | 0010001 | glial cell differentiation | EN-124k-90-group1142.Contig1 | | | | | | | | | | | | | |  |  |  |  |
| M | 0010001 | glial cell differentiation | EN-124k-90-group1142.Contig8 | | | | | | | | | | | | | |  |  |  |  |
| M | 0010001 | glial cell differentiation | EN-124k-90-group1142.Contig10 | | | | | | | | | | | | | | |  |  |  |
| M | 0010001 | glial cell differentiation | EN-124k-90-group1153.Contig1 | | | | | | | | | | | | | |  |  |  |  |
| M | 0010001 | glial cell differentiation | EN-124k-90-group1161.Contig1 | | | | | | | | | | | | | |  |  |  |  |
| M | 0010001 | glial cell differentiation | EN-124k-90-group1161.Contig2 | | | | | | | | | | | | | |  |  |  |  |
| M | 0010001 | glial cell differentiation | EN-124k-90-group1272.Contig1 | | | | | | | | | | | | | |  |  |  |  |
| M | 0010001 | glial cell differentiation | EN-124k-90-group1290.Contig1 | | | | | | | | | | | | | |  |  |  |  |
| M | 0010001 | glial cell differentiation | EN-124k-90-group1290.Contig2 | | | | | | | | | | | | | |  |  |  |  |
| M | 0010001 | glial cell differentiation | EN-124k-90-group1290.Contig3 | | | | | | | | | | | | | |  |  |  |  |
| M | 0010001 | glial cell differentiation | EN-124k-90-group1290.Contig5 | | | | | | | | | | | | | |  |  |  |  |
| M | 0010001 | glial cell differentiation | EN-124k-90-group1290.Contig6 | | | | | | | | | | | | | |  |  |  |  |
| M | 0010001 | glial cell differentiation | EN-124k-90-group1290.Contig8 | | | | | | | | | | | | | |  |  |  |  |
| M | 0010001 | glial cell differentiation | EN-124k-90-group1290.Contig10 | | | | | | | | | | | | | | |  |  |  |
| M | 0010001 | glial cell differentiation | EN-124k-90-group1304.Contig2 | | | | | | | | | | | | | |  |  |  |  |
| M | 0010001 | glial cell differentiation | EN-124k-90-group1304.Contig4 | | | | | | | | | | | | | |  |  |  |  |
| M | 0010001 | glial cell differentiation | EN-124k-90-group1304.Contig8 | | | | | | | | | | | | | |  |  |  |  |
| M | 0010001 | glial cell differentiation | EN-124k-90-group1333.Contig5 | | | | | | | | | | | | | |  |  |  |  |
| M | 0010001 | glial cell differentiation | EN-124k-90-group1351.Contig1 | | | | | | | | | | | | | |  |  |  |  |
| M | 0010001 | glial cell differentiation | EN-124k-90-group1423.Contig14 | | | | | | | | | | | | | | |  |  |  |
| M | 0010001 | glial cell differentiation | EN-124k-90-group1423.Contig20 | | | | | | | | | | | | | | |  |  |  |
| M | 0010001 | glial cell differentiation | EN-124k-90-group1465.Contig1 | | | | | | | | | | | | | |  |  |  |  |
| M | 0010001 | glial cell differentiation | EN-124k-90-group1465.Contig2 | | | | | | | | | | | | | |  |  |  |  |
| M | 0010001 | glial cell differentiation | EN-124k-90-group1493.Contig2 | | | | | | | | | | | | | |  |  |  |  |
| A | 0010001 | glial cell differentiation | EN-124k-90-group1521.Contig1 | | | | | | | | | | | | | |  |  |  |  |
| A | 0010001 | glial cell differentiation | EN-124k-90-group1521.Contig2 | | | | | | | | | | | | | |  |  |  |  |
| A | 0010001 | glial cell differentiation | EN-124k-90-group1521.Contig3 | | | | | | | | | | | | | |  |  |  |  |
| M | 0010001 | glial cell differentiation | EN-124k-90-group1542.Contig1 | | | | | | | | | | | | | |  |  |  |  |
| M | 0010001 | glial cell differentiation | EN-124k-90-group1585.Contig2 | | | | | | | | | | | | | |  |  |  |  |
| M | 0010001 | glial cell differentiation | EN-124k-90-group1728.Contig2 | | | | | | | | | | | | | |  |  |  |  |
| M | 0010001 | glial cell differentiation | EN-124k-90-group1735.Contig6 | | | | | | | | | | | | | |  |  |  |  |
| M | 0010001 | glial cell differentiation | EN-124k-90-group1770.Contig2 | | | | | | | | | | | | | |  |  |  |  |
| M | 0010001 | glial cell differentiation | EN-124k-90-group1782.Contig1 | | | | | | | | | | | | | |  |  |  |  |
| M | 0010001 | glial cell differentiation | EN-124k-90-group1869.Contig1 | | | | | | | | | | | | | |  |  |  |  |
| M | 0010001 | glial cell differentiation | EN-124k-90-group1869.Contig3 | | | | | | | | | | | | | |  |  |  |  |
| M | 0010001 | glial cell differentiation | EN-124k-90-group1909.Contig1 | | | | | | | | | | | | | |  |  |  |  |
| M | 0010001 | glial cell differentiation | EN-124k-90-group1909.Contig2 | | | | | | | | | | | | | |  |  |  |  |
| M | 0010001 | glial cell differentiation | EN-124k-90-group1909.Contig3 | | | | | | | | | | | | | |  |  |  |  |
| M | 0010001 | glial cell differentiation | EN-124k-90-group1919.Contig2 | | | | | | | | | | | | | |  |  |  |  |
| M | 0010001 | glial cell differentiation | EN-124k-90-group1980.Contig1 | | | | | | | | | | | | | |  |  |  |  |
| A | 0010001 | glial cell differentiation | EN-124k-90-group1993.Contig1 | | | | | | | | | | | | | |  |  |  |  |
| M | 0010001 | glial cell differentiation | EN-124k-90-group2069.Contig3 | | | | | | | | | | | | | |  |  |  |  |
| M | 0010001 | glial cell differentiation | EN-124k-90-group2081.Contig1 | | | | | | | | | | | | | |  |  |  |  |
| M | 0010001 | glial cell differentiation | EN-124k-90-group2081.Contig3 | | | | | | | | | | | | | |  |  |  |  |
| M | 0010001 | glial cell differentiation | EN-124k-90-group2097.Contig1 | | | | | | | | | | | | | |  |  |  |  |
| M | 0010001 | glial cell differentiation | EN-124k-90-group2132.Contig1 | | | | | | | | | | | | | |  |  |  |  |
| M | 0010001 | glial cell differentiation | EN-124k-90-group2193.Contig1 | | | | | | | | | | | | | |  |  |  |  |
| M | 0010001 | glial cell differentiation | EN-124k-90-group2200.Contig1 | | | | | | | | | | | | | |  |  |  |  |
| M | 0010001 | glial cell differentiation | EN-124k-90-group2221.Contig2 | | | | | | | | | | | | | |  |  |  |  |
| M | 0010001 | glial cell differentiation | EN-124k-90-group2223.Contig1 | | | | | | | | | | | | | |  |  |  |  |
| M | 0010001 | glial cell differentiation | EN-124k-90-group2268.Contig2 | | | | | | | | | | | | | |  |  |  |  |
| M | 0010001 | glial cell differentiation | EN-124k-90-group2428.Contig1 | | | | | | | | | | | | | |  |  |  |  |
| M | 0010001 | glial cell differentiation | EN-124k-90-group2443.Contig1 | | | | | | | | | | | | | |  |  |  |  |
| M | 0010001 | glial cell differentiation | EN-124k-90-group2498.Contig5 | | | | | | | | | | | | | |  |  |  |  |
| M | 0010001 | glial cell differentiation | EN-124k-90-group2744.Contig1 | | | | | | | | | | | | | |  |  |  |  |
| M | 0010001 | glial cell differentiation | EN-124k-90-group2835.Contig1 | | | | | | | | | | | | | |  |  |  |  |
| M | 0010001 | glial cell differentiation | EN-124k-90-group2835.Contig2 | | | | | | | | | | | | | |  |  |  |  |
| M | 0010001 | glial cell differentiation | EN-124k-90-group2842.Contig1 | | | | | | | | | | | | | |  |  |  |  |
| M | 0010001 | glial cell differentiation | EN-124k-90-group2852.Contig1 | | | | | | | | | | | | | |  |  |  |  |
| M | 0010001 | glial cell differentiation | EN-124k-90-group2879.Contig1 | | | | | | | | | | | | | |  |  |  |  |
| M | 0010001 | glial cell differentiation | EN-124k-90-group2879.Contig3 | | | | | | | | | | | | | |  |  |  |  |
| M | 0010001 | glial cell differentiation | EN-124k-90-group2917.Contig1 | | | | | | | | | | | | | |  |  |  |  |
| M | 0010001 | glial cell differentiation | EN-124k-90-group2917.Contig2 | | | | | | | | | | | | | |  |  |  |  |
| M | 0010001 | glial cell differentiation | EN-124k-90-group2917.Contig3 | | | | | | | | | | | | | |  |  |  |  |
| M | 0010001 | glial cell differentiation | EN-124k-90-group2917.Contig4 | | | | | | | | | | | | | |  |  |  |  |
| M | 0010001 | glial cell differentiation | EN-124k-90-group3082.Contig1 | | | | | | | | | | | | | |  |  |  |  |
| M | 0010001 | glial cell differentiation | EN-124k-90-group3082.Contig2 | | | | | | | | | | | | | |  |  |  |  |
| M | 0010001 | glial cell differentiation | EN-124k-90-group3223.Contig2 | | | | | | | | | | | | | |  |  |  |  |
| M | 0010001 | glial cell differentiation | EN-124k-90-group3313.Contig1 | | | | | | | | | | | | | |  |  |  |  |
| M | 0010001 | glial cell differentiation | EN-124k-90-group3313.Contig3 | | | | | | | | | | | | | |  |  |  |  |
| M | 0010001 | glial cell differentiation | EN-124k-90-group3315.Contig6 | | | | | | | | | | | | | |  |  |  |  |
| M | 0010001 | glial cell differentiation | EN-124k-90-group3343.Contig1 | | | | | | | | | | | | | |  |  |  |  |
| E | 0010001 | glial cell differentiation | EN-124k-90-group3343.Contig2 | | | | | | | | | | | | | |  |  |  |  |
| M | 0010001 | glial cell differentiation | EN-124k-90-group3394.Contig1 | | | | | | | | | | | | | |  |  |  |  |
| M | 0010001 | glial cell differentiation | EN-124k-90-group3394.Contig2 | | | | | | | | | | | | | |  |  |  |  |
| M | 0010001 | glial cell differentiation | EN-124k-90-group3394.Contig3 | | | | | | | | | | | | | |  |  |  |  |
| M | 0010001 | glial cell differentiation | EN-124k-90-group3394.Contig4 | | | | | | | | | | | | | |  |  |  |  |
| M | 0010001 | glial cell differentiation | EN-124k-90-group3408.Contig1 | | | | | | | | | | | | | |  |  |  |  |
| M | 0010001 | glial cell differentiation | EN-124k-90-group3408.Contig2 | | | | | | | | | | | | | |  |  |  |  |
| M | 0010001 | glial cell differentiation | EN-124k-90-group3479.Contig1 | | | | | | | | | | | | | |  |  |  |  |
| M | 0010001 | glial cell differentiation | EN-124k-90-group3516.Contig1 | | | | | | | | | | | | | |  |  |  |  |
| M | 0010001 | glial cell differentiation | EN-124k-90-group3520.Contig1 | | | | | | | | | | | | | |  |  |  |  |
| M | 0010001 | glial cell differentiation | EN-124k-90-group3522.Contig1 | | | | | | | | | | | | | |  |  |  |  |
| M | 0010001 | glial cell differentiation | EN-124k-90-group3586.Contig1 | | | | | | | | | | | | | |  |  |  |  |
| M | 0010001 | glial cell differentiation | EN-124k-90-group3590.Contig1 | | | | | | | | | | | | | |  |  |  |  |
| M | 0010001 | glial cell differentiation | EN-124k-90-group3590.Contig2 | | | | | | | | | | | | | |  |  |  |  |
| E | 0010001 | glial cell differentiation | EN-124k-90-group3611.Contig1 | | | | | | | | | | | | | |  |  |  |  |
| M | 0010001 | glial cell differentiation | EN-124k-90-group3713.Contig1 | | | | | | | | | | | | | |  |  |  |  |
| M | 0010001 | glial cell differentiation | EN-124k-90-group3882.Contig1 | | | | | | | | | | | | | |  |  |  |  |
| M | 0010001 | glial cell differentiation | EN-124k-90-group3882.Contig2 | | | | | | | | | | | | | |  |  |  |  |
| M | 0010001 | glial cell differentiation | EN-124k-90-group3883.Contig1 | | | | | | | | | | | | | |  |  |  |  |
| M | 0010001 | glial cell differentiation | EN-124k-90-group3883.Contig2 | | | | | | | | | | | | | |  |  |  |  |
| M | 0010001 | glial cell differentiation | EN-124k-90-group3883.Contig3 | | | | | | | | | | | | | |  |  |  |  |
| M | 0010001 | glial cell differentiation | EN-124k-90-group3883.Contig4 | | | | | | | | | | | | | |  |  |  |  |
| M | 0010001 | glial cell differentiation | EN-124k-90-group3897.Contig1 | | | | | | | | | | | | | |  |  |  |  |
| M | 0010001 | glial cell differentiation | EN-124k-90-group3933.Contig1 | | | | | | | | | | | | | |  |  |  |  |
| M | 0010001 | glial cell differentiation | EN-124k-90-group3953.Contig1 | | | | | | | | | | | | | |  |  |  |  |
| M | 0010001 | glial cell differentiation | EN-124k-90-group3960.Contig1 | | | | | | | | | | | | | |  |  |  |  |
| M | 0010001 | glial cell differentiation | EN-124k-90-group3960.Contig2 | | | | | | | | | | | | | |  |  |  |  |
| E | 0010001 | glial cell differentiation | EN-124k-90-group4039.Contig1 | | | | | | | | | | | | | |  |  |  |  |
| M | 0010001 | glial cell differentiation | EN-124k-90-group4045.Contig1 | | | | | | | | | | | | | |  |  |  |  |
| M | 0010001 | glial cell differentiation | EN-124k-90-group4109.Contig1 | | | | | | | | | | | | | |  |  |  |  |
| M | 0010001 | glial cell differentiation | EN-124k-90-group4168.Contig1 | | | | | | | | | | | | | |  |  |  |  |
| M | 0010001 | glial cell differentiation | EN-124k-90-group4168.Contig3 | | | | | | | | | | | | | |  |  |  |  |
| M | 0010001 | glial cell differentiation | EN-124k-90-group4183.Contig1 | | | | | | | | | | | | | |  |  |  |  |
| M | 0010001 | glial cell differentiation | EN-124k-90-group4365.Contig1 | | | | | | | | | | | | | |  |  |  |  |
| M | 0010001 | glial cell differentiation | EN-124k-90-group4372.Contig1 | | | | | | | | | | | | | |  |  |  |  |
| M | 0010001 | glial cell differentiation | EN-124k-90-group4410.Contig1 | | | | | | | | | | | | | |  |  |  |  |
| M | 0010001 | glial cell differentiation | EN-124k-90-group4424.Contig1 | | | | | | | | | | | | | |  |  |  |  |
| M | 0010001 | glial cell differentiation | EN-124k-90-group4424.Contig2 | | | | | | | | | | | | | |  |  |  |  |
| M | 0010001 | glial cell differentiation | EN-124k-90-group4562.Contig1 | | | | | | | | | | | | | |  |  |  |  |
| M | 0010001 | glial cell differentiation | EN-124k-90-group4668.Contig1 | | | | | | | | | | | | | |  |  |  |  |
| M | 0010001 | glial cell differentiation | EN-124k-90-group4753.Contig1 | | | | | | | | | | | | | |  |  |  |  |
| M | 0010001 | glial cell differentiation | EN-124k-90-group4763.Contig1 | | | | | | | | | | | | | |  |  |  |  |
| M | 0010001 | glial cell differentiation | EN-124k-90-group4824.Contig4 | | | | | | | | | | | | | |  |  |  |  |
| M | 0010001 | glial cell differentiation | EN-124k-90-group4929.Contig2 | | | | | | | | | | | | | |  |  |  |  |
| M | 0010001 | glial cell differentiation | EN-124k-90-group4944.Contig2 | | | | | | | | | | | | | |  |  |  |  |
| M | 0010001 | glial cell differentiation | EN-124k-90-group5022.Contig1 | | | | | | | | | | | | | |  |  |  |  |
| M | 0010001 | glial cell differentiation | EN-124k-90-group5022.Contig2 | | | | | | | | | | | | | |  |  |  |  |
| M | 0010001 | glial cell differentiation | EN-124k-90-group5038.Contig1 | | | | | | | | | | | | | |  |  |  |  |
| M | 0010001 | glial cell differentiation | EN-124k-90-group5054.Contig1 | | | | | | | | | | | | | |  |  |  |  |
| A | 0010001 | glial cell differentiation | EN-124k-90-group5054.Contig2 | | | | | | | | | | | | | |  |  |  |  |
| M | 0010001 | glial cell differentiation | EN-124k-90-group5054.Contig3 | | | | | | | | | | | | | |  |  |  |  |
| M | 0010001 | glial cell differentiation | EN-124k-90-group5054.Contig4 | | | | | | | | | | | | | |  |  |  |  |
| M | 0010001 | glial cell differentiation | EN-124k-90-group5063.Contig1 | | | | | | | | | | | | | |  |  |  |  |
| M | 0010001 | glial cell differentiation | EN-124k-90-group5172.Contig1 | | | | | | | | | | | | | |  |  |  |  |
| M | 0010001 | glial cell differentiation | EN-124k-90-group5173.Contig1 | | | | | | | | | | | | | |  |  |  |  |
| M | 0010001 | glial cell differentiation | EN-124k-90-group5252.Contig1 | | | | | | | | | | | | | |  |  |  |  |
| M | 0010001 | glial cell differentiation | EN-124k-90-group5273.Contig3 | | | | | | | | | | | | | |  |  |  |  |
| M | 0010001 | glial cell differentiation | EN-124k-90-group5282.Contig1 | | | | | | | | | | | | | |  |  |  |  |
| M | 0010001 | glial cell differentiation | EN-124k-90-group5298.Contig1 | | | | | | | | | | | | | |  |  |  |  |
| M | 0010001 | glial cell differentiation | EN-124k-90-group5383.Contig1 | | | | | | | | | | | | | |  |  |  |  |
| M | 0010001 | glial cell differentiation | EN-124k-90-group5459.Contig2 | | | | | | | | | | | | | |  |  |  |  |
| M | 0010001 | glial cell differentiation | EN-124k-90-group5491.Contig1 | | | | | | | | | | | | | |  |  |  |  |
| M | 0010001 | glial cell differentiation | EN-124k-90-group5491.Contig2 | | | | | | | | | | | | | |  |  |  |  |
| M | 0010001 | glial cell differentiation | EN-124k-90-group5532.Contig1 | | | | | | | | | | | | | |  |  |  |  |
| M | 0010001 | glial cell differentiation | EN-124k-90-group5532.Contig2 | | | | | | | | | | | | | |  |  |  |  |
| M | 0010001 | glial cell differentiation | EN-124k-90-group5967.Contig1 | | | | | | | | | | | | | |  |  |  |  |
| M | 0010001 | glial cell differentiation | EN-124k-90-group5968.Contig1 | | | | | | | | | | | | | |  |  |  |  |
| M | 0010001 | glial cell differentiation | EN-124k-90-group6033.Contig1 | | | | | | | | | | | | | |  |  |  |  |
| M | 0010001 | glial cell differentiation | EN-124k-90-group6033.Contig2 | | | | | | | | | | | | | |  |  |  |  |
| M | 0010001 | glial cell differentiation | EN-124k-90-group6033.Contig3 | | | | | | | | | | | | | |  |  |  |  |
| M | 0010001 | glial cell differentiation | EN-124k-90-group6038.Contig1 | | | | | | | | | | | | | |  |  |  |  |
| M | 0010001 | glial cell differentiation | EN-124k-90-group6229.Contig1 | | | | | | | | | | | | | |  |  |  |  |
| M | 0010001 | glial cell differentiation | EN-124k-90-group6358.Contig1 | | | | | | | | | | | | | |  |  |  |  |
| M | 0010001 | glial cell differentiation | EN-124k-90-group6358.Contig2 | | | | | | | | | | | | | |  |  |  |  |
| M | 0010001 | glial cell differentiation | EN-124k-90-group6360.Contig1 | | | | | | | | | | | | | |  |  |  |  |
| M | 0010001 | glial cell differentiation | EN-124k-90-group6400.Contig1 | | | | | | | | | | | | | |  |  |  |  |
| M | 0010001 | glial cell differentiation | EN-124k-90-group6424.Contig1 | | | | | | | | | | | | | |  |  |  |  |
| M | 0010001 | glial cell differentiation | EN-124k-90-group6457.Contig1 | | | | | | | | | | | | | |  |  |  |  |
| M | 0010001 | glial cell differentiation | EN-124k-90-group6472.Contig1 | | | | | | | | | | | | | |  |  |  |  |
| M | 0010001 | glial cell differentiation | EN-124k-90-group6472.Contig2 | | | | | | | | | | | | | |  |  |  |  |
| M | 0010001 | glial cell differentiation | EN-124k-90-group6694.Contig1 | | | | | | | | | | | | | |  |  |  |  |
| M | 0010001 | glial cell differentiation | EN-124k-90-group6809.Contig1 | | | | | | | | | | | | | |  |  |  |  |
| E | 0010001 | glial cell differentiation | EN-124k-90-group6858.Contig1 | | | | | | | | | | | | | |  |  |  |  |
| M | 0010001 | glial cell differentiation | EN-124k-90-group6904.Contig1 | | | | | | | | | | | | | |  |  |  |  |
| E | 0010001 | glial cell differentiation | EN-124k-90-group6970.Contig1 | | | | | | | | | | | | | |  |  |  |  |
| M | 0010001 | glial cell differentiation | EN-124k-90-group7013.Contig1 | | | | | | | | | | | | | |  |  |  |  |
| M | 0010001 | glial cell differentiation | EN-124k-90-group7067.Contig1 | | | | | | | | | | | | | |  |  |  |  |
| M | 0010001 | glial cell differentiation | EN-124k-90-group7149.Contig2 | | | | | | | | | | | | | |  |  |  |  |
| M | 0010001 | glial cell differentiation | EN-124k-90-group7157.Contig2 | | | | | | | | | | | | | |  |  |  |  |
| M | 0010001 | glial cell differentiation | EN-124k-90-group7165.Contig1 | | | | | | | | | | | | | |  |  |  |  |
| M | 0010001 | glial cell differentiation | EN-124k-90-group7231.Contig1 | | | | | | | | | | | | | |  |  |  |  |
| M | 0010001 | glial cell differentiation | EN-124k-90-group7234.Contig1 | | | | | | | | | | | | | |  |  |  |  |
| M | 0010001 | glial cell differentiation | EN-124k-90-group7343.Contig1 | | | | | | | | | | | | | |  |  |  |  |
| M | 0010001 | glial cell differentiation | EN-124k-90-group7440.Contig1 | | | | | | | | | | | | | |  |  |  |  |
| M | 0010001 | glial cell differentiation | EN-124k-90-group7457.Contig1 | | | | | | | | | | | | | |  |  |  |  |
| A | 0010001 | glial cell differentiation | EN-124k-90-group7650.Contig1 | | | | | | | | | | | | | |  |  |  |  |
| M | 0010001 | glial cell differentiation | EN-124k-90-group7666.Contig2 | | | | | | | | | | | | | |  |  |  |  |
| M | 0010001 | glial cell differentiation | EN-124k-90-group7786.Contig1 | | | | | | | | | | | | | |  |  |  |  |
| M | 0010001 | glial cell differentiation | EN-124k-90-group7915.Contig1 | | | | | | | | | | | | | |  |  |  |  |
| M | 0010001 | glial cell differentiation | EN-124k-90-group8079.Contig1 | | | | | | | | | | | | | |  |  |  |  |
| M | 0010001 | glial cell differentiation | EN-124k-90-group8079.Contig2 | | | | | | | | | | | | | |  |  |  |  |
| E | 0010001 | glial cell differentiation | EN-124k-90-group8101.Contig1 | | | | | | | | | | | | | |  |  |  |  |
| M | 0010001 | glial cell differentiation | EN-124k-90-group8135.Contig2 | | | | | | | | | | | | | |  |  |  |  |
| M | 0010001 | glial cell differentiation | EN-124k-90-group8245.Contig2 | | | | | | | | | | | | | |  |  |  |  |
| M | 0010001 | glial cell differentiation | EN-124k-90-group8245.Contig3 | | | | | | | | | | | | | |  |  |  |  |
| M | 0010001 | glial cell differentiation | EN-124k-90-group8325.Contig1 | | | | | | | | | | | | | |  |  |  |  |
| M | 0010001 | glial cell differentiation | EN-124k-90-group8351.Contig2 | | | | | | | | | | | | | |  |  |  |  |
| M | 0010001 | glial cell differentiation | EN-124k-90-group8484.Contig3 | | | | | | | | | | | | | |  |  |  |  |
| E | 0010001 | glial cell differentiation | EN-124k-90-group8513.Contig1 | | | | | | | | | | | | | |  |  |  |  |
| A | 0010001 | glial cell differentiation | EN-124k-90-group8519.Contig1 | | | | | | | | | | | | | |  |  |  |  |
| M | 0010001 | glial cell differentiation | EN-124k-90-group8568.Contig1 | | | | | | | | | | | | | |  |  |  |  |
| M | 0010001 | glial cell differentiation | EN-124k-90-group8645.Contig1 | | | | | | | | | | | | | |  |  |  |  |
| M | 0010001 | glial cell differentiation | EN-124k-90-group8685.Contig1 | | | | | | | | | | | | | |  |  |  |  |
| M | 0010001 | glial cell differentiation | EN-124k-90-group8775.Contig1 | | | | | | | | | | | | | |  |  |  |  |
| M | 0010001 | glial cell differentiation | EN-124k-90-group8799.Contig2 | | | | | | | | | | | | | |  |  |  |  |
| M | 0010001 | glial cell differentiation | EN-124k-90-group8850.Contig1 | | | | | | | | | | | | | |  |  |  |  |
| M | 0010001 | glial cell differentiation | EN-124k-90-group8897.Contig1 | | | | | | | | | | | | | |  |  |  |  |
| M | 0010001 | glial cell differentiation | EN-124k-90-group8937.Contig1 | | | | | | | | | | | | | |  |  |  |  |
| M | 0010001 | glial cell differentiation | EN-124k-90-group8937.Contig2 | | | | | | | | | | | | | |  |  |  |  |
| A | 0010001 | glial cell differentiation | EN-124k-90-group8957.Contig1 | | | | | | | | | | | | | |  |  |  |  |
| M | 0010001 | glial cell differentiation | EN-124k-90-group9159.Contig1 | | | | | | | | | | | | | |  |  |  |  |
| M | 0010001 | glial cell differentiation | EN-124k-90-group9165.Contig1 | | | | | | | | | | | | | |  |  |  |  |
| A | 0010001 | glial cell differentiation | EN-124k-90-group9245.Contig1 | | | | | | | | | | | | | |  |  |  |  |
| M | 0010001 | glial cell differentiation | EN-124k-90-group9278.Contig2 | | | | | | | | | | | | | |  |  |  |  |
| M | 0010001 | glial cell differentiation | EN-124k-90-group9299.Contig3 | | | | | | | | | | | | | |  |  |  |  |
| M | 0010001 | glial cell differentiation | EN-124k-90-group9317.Contig1 | | | | | | | | | | | | | |  |  |  |  |
| M | 0010001 | glial cell differentiation | EN-124k-90-group9446.Contig1 | | | | | | | | | | | | | |  |  |  |  |
| M | 0010001 | glial cell differentiation | EN-124k-90-group9819.Contig1 | | | | | | | | | | | | | |  |  |  |  |
| M | 0010001 | glial cell differentiation | EN-124k-90-group9931.Contig1 | | | | | | | | | | | | | |  |  |  |  |
| M | 0010001 | glial cell differentiation | EN-124k-90-group10053.Contig1 | | | | | | | | | | | | | | |  |  |  |
| M | 0010001 | glial cell differentiation | EN-124k-90-group10085.Contig1 | | | | | | | | | | | | | | |  |  |  |
| M | 0010001 | glial cell differentiation | EN-124k-90-group10085.Contig2 | | | | | | | | | | | | | | |  |  |  |
| M | 0010001 | glial cell differentiation | EN-124k-90-group10221.Contig1 | | | | | | | | | | | | | | |  |  |  |
| M | 0010001 | glial cell differentiation | EN-124k-90-group10394.Contig1 | | | | | | | | | | | | | | |  |  |  |
| M | 0010001 | glial cell differentiation | EN-124k-90-group10528.Contig1 | | | | | | | | | | | | | | |  |  |  |
| M | 0010001 | glial cell differentiation | EN-124k-90-group10630.Contig1 | | | | | | | | | | | | | | |  |  |  |
| M | 0010001 | glial cell differentiation | EN-124k-90-group10630.Contig2 | | | | | | | | | | | | | | |  |  |  |
| M | 0010001 | glial cell differentiation | EN-124k-90-group10652.Contig1 | | | | | | | | | | | | | | |  |  |  |
| M | 0010001 | glial cell differentiation | EN-124k-90-group10790.Contig1 | | | | | | | | | | | | | | |  |  |  |
| A | 0010001 | glial cell differentiation | EN-124k-90-group10802.Contig1 | | | | | | | | | | | | | | |  |  |  |
| M | 0010001 | glial cell differentiation | EN-124k-90-group10838.Contig2 | | | | | | | | | | | | | | |  |  |  |
| M | 0010001 | glial cell differentiation | EN-124k-90-group10912.Contig1 | | | | | | | | | | | | | | |  |  |  |
| M | 0010001 | glial cell differentiation | EN-124k-90-group10928.Contig1 | | | | | | | | | | | | | | |  |  |  |
| M | 0010001 | glial cell differentiation | EN-124k-90-group11100.Contig1 | | | | | | | | | | | | | | |  |  |  |
| M | 0010001 | glial cell differentiation | EN-124k-90-group11235.Contig1 | | | | | | | | | | | | | | |  |  |  |
| M | 0010001 | glial cell differentiation | EN-124k-90-group11317.Contig1 | | | | | | | | | | | | | | |  |  |  |
| M | 0010001 | glial cell differentiation | EN-124k-90-group11317.Contig2 | | | | | | | | | | | | | | |  |  |  |
| M | 0010001 | glial cell differentiation | EN-124k-90-group11408.Contig1 | | | | | | | | | | | | | | |  |  |  |
| M | 0010001 | glial cell differentiation | EN-124k-90-group11519.Contig1 | | | | | | | | | | | | | | |  |  |  |
| M | 0010001 | glial cell differentiation | EN-124k-90-group12008.Contig1 | | | | | | | | | | | | | | |  |  |  |
| E | 0010001 | glial cell differentiation | EN-124k-90-group12010.Contig1 | | | | | | | | | | | | | | |  |  |  |
| E | 0010001 | glial cell differentiation | EN-124k-90-group12010.Contig2 | | | | | | | | | | | | | | |  |  |  |
| M | 0010001 | glial cell differentiation | EN-124k-90-group12143.Contig1 | | | | | | | | | | | | | | |  |  |  |
| M | 0010001 | glial cell differentiation | EN-124k-90-group12409.Contig2 | | | | | | | | | | | | | | |  |  |  |
| A | 0010001 | glial cell differentiation | EN-124k-90-group12616.Contig1 | | | | | | | | | | | | | | |  |  |  |
| A | 0010001 | glial cell differentiation | EN-124k-90-group12832.Contig1 | | | | | | | | | | | | | | |  |  |  |
| M | 0010001 | glial cell differentiation | EN-124k-90-group13095.Contig1 | | | | | | | | | | | | | | |  |  |  |
| M | 0010001 | glial cell differentiation | EN-124k-90-group13121.Contig1 | | | | | | | | | | | | | | |  |  |  |
| M | 0010001 | glial cell differentiation | EN-124k-90-group13235.Contig1 | | | | | | | | | | | | | | |  |  |  |
| M | 0010001 | glial cell differentiation | EN-124k-90-group13502.Contig2 | | | | | | | | | | | | | | |  |  |  |
| M | 0010001 | glial cell differentiation | EN-124k-90-group13579.Contig1 | | | | | | | | | | | | | | |  |  |  |
| M | 0010001 | glial cell differentiation | EN-124k-90-group13579.Contig2 | | | | | | | | | | | | | | |  |  |  |
| M | 0010001 | glial cell differentiation | EN-124k-90-group13579.Contig3 | | | | | | | | | | | | | | |  |  |  |
| M | 0010001 | glial cell differentiation | EN-124k-90-group14708.Contig1 | | | | | | | | | | | | | | |  |  |  |
| A | 0010001 | glial cell differentiation | EN-124k-90-group230.gs\_25885 | | | | | | | | | | | | | | |  |  |  |
| A | 0010001 | glial cell differentiation | EN-124k-90-group735.gs\_7849 | | | | | | | | | | | | | |  |  |  |  |
| E | 0010001 | glial cell differentiation | EN-124k-90-group900.EN\_iowa\_15436 | | | | | | | | | | | | | | |  |  |  |
| A | 0010001 | glial cell differentiation | EN-124k-90-group900.gs\_31783 | | | | | | | | | | | | | | |  |  |  |
| A | 0010001 | glial cell differentiation | EN-124k-90-group900.gs\_71447 | | | | | | | | | | | | | | |  |  |  |
| A | 0010001 | glial cell differentiation | EN-124k-90-group1153.gs\_635 | | | | | | | | | | | | | |  |  |  |  |
| A | 0010001 | glial cell differentiation | EN-124k-90-group1153.gs\_33239 | | | | | | | | | | | | | | |  |  |  |
| A | 0010001 | glial cell differentiation | EN-124k-90-group1290.gs\_26142 | | | | | | | | | | | | | | |  |  |  |
| A | 0010001 | glial cell differentiation | EN-124k-90-group1290.gs\_22118 | | | | | | | | | | | | | | |  |  |  |
| A | 0010001 | glial cell differentiation | EN-124k-90-group1290.gs\_35147 | | | | | | | | | | | | | | |  |  |  |
| A | 0010001 | glial cell differentiation | EN-124k-90-group1290.gs\_13448 | | | | | | | | | | | | | | |  |  |  |
| A | 0010001 | glial cell differentiation | EN-124k-90-group1290.gs\_16770 | | | | | | | | | | | | | | |  |  |  |
| E | 0010001 | glial cell differentiation | EN-124k-90-group1290.EN\_iowa\_15637 | | | | | | | | | | | | | | | |  |  |
| A | 0010001 | glial cell differentiation | EN-124k-90-group1290.gs\_28522 | | | | | | | | | | | | | | |  |  |  |
| A | 0010001 | glial cell differentiation | EN-124k-90-group1290.gs\_84561 | | | | | | | | | | | | | | |  |  |  |
| A | 0010001 | glial cell differentiation | EN-124k-90-group1423.gs\_16014 | | | | | | | | | | | | | | |  |  |  |
| A | 0010001 | glial cell differentiation | EN-124k-90-group1423.gs\_11172 | | | | | | | | | | | | | | |  |  |  |
| A | 0010001 | glial cell differentiation | EN-124k-90-group1423.gs\_52362 | | | | | | | | | | | | | | |  |  |  |
| A | 0010001 | glial cell differentiation | EN-124k-90-group1423.gs\_75717 | | | | | | | | | | | | | | |  |  |  |
| E | 0010001 | glial cell differentiation | EN-124k-90-group1493.jgi\_contig\_JGI\_CBBP6493\_fwd | | | | | | | | | | | | | | | | | |
| A | 0010001 | glial cell differentiation | EN-124k-90-group1521.gs\_86350 | | | | | | | | | | | | | | |  |  |  |
| A | 0010001 | glial cell differentiation | EN-124k-90-group1782.gs\_16663 | | | | | | | | | | | | | | |  |  |  |
| A | 0010001 | glial cell differentiation | EN-124k-90-group2069.gs\_85543 | | | | | | | | | | | | | | |  |  |  |
| A | 0010001 | glial cell differentiation | EN-124k-90-group2069.gs\_63060 | | | | | | | | | | | | | | |  |  |  |
| A | 0010001 | glial cell differentiation | EN-124k-90-group2069.gs\_69693 | | | | | | | | | | | | | | |  |  |  |
| E | 0010001 | glial cell differentiation | EN-124k-90-group2221.jgi\_contig\_JGI\_CBBP11225\_fwd | | | | | | | | | | | | | | | | | |
| E | 0010001 | glial cell differentiation | EN-124k-90-group2443.EN\_iowa\_8559 | | | | | | | | | | | | | | |  |  |  |
| A | 0010001 | glial cell differentiation | EN-124k-90-group2879.gs\_23381 | | | | | | | | | | | | | | |  |  |  |
| A | 0010001 | glial cell differentiation | EN-124k-90-group3516.gs\_39395 | | | | | | | | | | | | | | |  |  |  |
| A | 0010001 | glial cell differentiation | EN-124k-90-group3522.gs\_9768 | | | | | | | | | | | | | | |  |  |  |
| E | 0010001 | glial cell differentiation | EN-124k-90-group3611.EN\_iowa\_4463 | | | | | | | | | | | | | | |  |  |  |
| A | 0010001 | glial cell differentiation | EN-124k-90-group3713.gs\_7861 | | | | | | | | | | | | | | |  |  |  |
| A | 0010001 | glial cell differentiation | EN-124k-90-group3883.gs\_85360 | | | | | | | | | | | | | | |  |  |  |
| A | 0010001 | glial cell differentiation | EN-124k-90-group5491.gs\_7631 | | | | | | | | | | | | | | |  |  |  |
| A | 0010001 | glial cell differentiation | EN-124k-90-group5491.gs\_75129 | | | | | | | | | | | | | | |  |  |  |
| A | 0010001 | glial cell differentiation | EN-124k-90-group5532.gs\_21595 | | | | | | | | | | | | | | |  |  |  |
| A | 0010001 | glial cell differentiation | EN-124k-90-group5532.gs\_71380 | | | | | | | | | | | | | | |  |  |  |
| A | 0010001 | glial cell differentiation | EN-124k-90-group5532.gs\_38926 | | | | | | | | | | | | | | |  |  |  |
| A | 0010001 | glial cell differentiation | EN-124k-90-group5532.gs\_31235 | | | | | | | | | | | | | | |  |  |  |
| E | 0010001 | glial cell differentiation | EN-124k-90-group5532.EN\_iowa\_9665 | | | | | | | | | | | | | | |  |  |  |
| A | 0010001 | glial cell differentiation | EN-124k-90-group5532.gs\_59967 | | | | | | | | | | | | | | |  |  |  |
| E | 0010001 | glial cell differentiation | EN-124k-90-group5532.EN\_iowa\_13749 | | | | | | | | | | | | | | | |  |  |
| A | 0010001 | glial cell differentiation | EN-124k-90-group5532.gs\_23346 | | | | | | | | | | | | | | |  |  |  |
| E | 0010001 | glial cell differentiation | EN-124k-90-group5532.EN\_iowa\_12774 | | | | | | | | | | | | | | | |  |  |
| A | 0010001 | glial cell differentiation | EN-124k-90-group5532.gs\_2852 | | | | | | | | | | | | | | |  |  |  |
| A | 0010001 | glial cell differentiation | EN-124k-90-group5532.gs\_25188 | | | | | | | | | | | | | | |  |  |  |
| A | 0010001 | glial cell differentiation | EN-124k-90-group5532.gs\_35715 | | | | | | | | | | | | | | |  |  |  |
| A | 0010001 | glial cell differentiation | EN-124k-90-group5532.gs\_7280 | | | | | | | | | | | | | | |  |  |  |
| E | 0010001 | glial cell differentiation | EN-124k-90-group6472.jgi\_contig\_JGI\_CBBP2402\_fwd | | | | | | | | | | | | | | | | | |
| A | 0010001 | glial cell differentiation | EN-124k-90-group7440.gs\_75782 | | | | | | | | | | | | | | |  |  |  |
| A | 0010001 | glial cell differentiation | EN-124k-90-group7650.gs\_30549 | | | | | | | | | | | | | | |  |  |  |
| A | 0010001 | glial cell differentiation | EN-124k-90-group7650.gs\_59944 | | | | | | | | | | | | | | |  |  |  |
| A | 0010001 | glial cell differentiation | EN-124k-90-group8484.gs\_85230 | | | | | | | | | | | | | | |  |  |  |
| A | 0019838 | growth factor binding | EN-124k-90-group10674.gs\_45324 | | | | | | | | | | | | | | |  |  |  |
| E | 0019838 | growth factor binding | EN-124k-90-group343.jgi\_contig\_JGI\_CBBP19299\_fwd | | | | | | | | | | | | | | | | | |
| A | 0019838 | growth factor binding | EN-124k-90-group807.gs\_71281 | | | | | | | | | | | | | | |  |  |  |
| E | 0019838 | growth factor binding | EN-124k-90-group1306.EN\_iowa\_2354 | | | | | | | | | | | | | | |  |  |  |
| E | 0019838 | growth factor binding | EN-124k-90-group1852.jgi\_paired\_JGI\_CBBP10940\_fwd | | | | | | | | | | | | | | | | | |
| E | 0019838 | growth factor binding | EN-124k-90-group2530.jgi\_paired\_JGI\_CBBP5537\_fwd | | | | | | | | | | | | | | | | | |
| E | 0019838 | growth factor binding | EN-124k-90-group3448.jgi\_paired\_JGI\_CBBP19729\_fwd | | | | | | | | | | | | | | | | | |
| E | 0019838 | growth factor binding | EN-124k-90-group5117.jgi\_paired\_JGI\_CBBP19153\_fwd | | | | | | | | | | | | | | | | | |
| E | 0019838 | growth factor binding | EN-124k-90-group5834.jgi\_contig\_JGI\_CBBP12701\_fwd | | | | | | | | | | | | | | | | | |
| E | 0019838 | growth factor binding | EN-124k-90-group6219.EN\_iowa\_9387 | | | | | | | | | | | | | | |  |  |  |
| E | 0019838 | growth factor binding | EN-124k-90-group6737.jgi\_contig\_JGI\_CBBP17820\_fwd | | | | | | | | | | | | | | | | | |
| E | 0019838 | growth factor binding | EN-124k-90-group7598.jgi\_unpaired\_JGI\_CBBP18343\_fwd | | | | | | | | | | | | | | | | | |
| A | 0019838 | growth factor binding | EN-124k-90-group8310.gs\_24913 | | | | | | | | | | | | | | |  |  |  |
| A | 0019838 | growth factor binding | EN-124k-90-group9442.gs\_10851 | | | | | | | | | | | | | | |  |  |  |
| A | 0019838 | growth factor binding | EN-124k-90-group9467.gs\_16039 | | | | | | | | | | | | | | |  |  |  |
| A | 0019838 | growth factor binding | EN-124k-90-group9714.gs\_64750 | | | | | | | | | | | | | | |  |  |  |
| E | 0019838 | growth factor binding | EN-124k-90-group9839.EN\_iowa\_2226 | | | | | | | | | | | | | | |  |  |  |
| E | 0019838 | growth factor binding | EN-124k-90-group10230.jgi\_paired\_JGI\_CBBP3918\_fwd | | | | | | | | | | | | | | | | | |
| A | 0019838 | growth factor binding | EN-124k-90-group10312.gs\_22624 | | | | | | | | | | | | | | |  |  |  |
| E | 0019838 | growth factor binding | EN-124k-90-group10878.jgi\_paired\_JGI\_CBBP952\_rev | | | | | | | | | | | | | | | | | |
| A | 0019838 | growth factor binding | EN-124k-90-group10935.gs\_64995 | | | | | | | | | | | | | | |  |  |  |
| A | 0019838 | growth factor binding | EN-124k-90-group11738.gs\_53113 | | | | | | | | | | | | | | |  |  |  |
| E | 0019838 | growth factor binding | EN-124k-90-group11780.jgi\_paired\_JGI\_CBBP12332\_fwd | | | | | | | | | | | | | | | | | |
| E | 0019838 | growth factor binding | EN-124k-90-group12012.jgi\_contig\_JGI\_CBBP10480\_fwd | | | | | | | | | | | | | | | | | |
| E | 0019838 | growth factor binding | EN-124k-90-group12191.jgi\_paired\_JGI\_CBBP10887\_fwd | | | | | | | | | | | | | | | | | |
| E | 0019838 | growth factor binding | EN-124k-90-group12452.EN\_iowa\_9722 | | | | | | | | | | | | | | | |  |  |
| E | 0019838 | growth factor binding | EN-124k-90-group12595.jgi\_contig\_JGI\_CBBP15955\_fwd | | | | | | | | | | | | | | | | | |
| A | 0019838 | growth factor binding | EN-124k-90-group14563.gs\_48486 | | | | | | | | | | | | | | |  |  |  |
| A | 0019838 | growth factor binding | EN-124k-90-group14582.gs\_17139 | | | | | | | | | | | | | | |  |  |  |
| A | 0019838 | growth factor binding | EN-124k-90-group14948.gs\_26769 | | | | | | | | | | | | | | |  |  |  |
| A | 0019838 | growth factor binding | EN-124k-90-group15044.gs\_60727 | | | | | | | | | | | | | | |  |  |  |
| A | 0019838 | growth factor binding | EN-124k-90-group15056.gs\_73097 | | | | | | | | | | | | | | |  |  |  |
| E | 0019838 | growth factor binding | EN-124k-90-group15200.jgi\_paired\_JGI\_CBBP20190\_fwd | | | | | | | | | | | | | | | | | |
| E | 0019838 | growth factor binding | EN-124k-90-group15320.jgi\_paired\_JGI\_CBBP9817\_fwd | | | | | | | | | | | | | | | | | |
| A | 0019838 | growth factor binding | EN-124k-90-group15770.gs\_45569 | | | | | | | | | | | | | | |  |  |  |
| E | 0019838 | growth factor binding | EN-124k-90-group16200.jgi\_paired\_JGI\_CBBP10444\_rev | | | | | | | | | | | | | | | | | |
| A | 0019838 | growth factor binding | EN-124k-90-group16489.gs\_80464 | | | | | | | | | | | | | | |  |  |  |
| A | 0019838 | growth factor binding | EN-124k-90-group16523.gs\_75670 | | | | | | | | | | | | | | |  |  |  |
| A | 0019838 | growth factor binding | EN-124k-90-group16575.gs\_6 | | | | | | | | | | | | | |  |  |  |  |
| A | 0019838 | growth factor binding | EN-124k-90-group16600.gs\_29512 | | | | | | | | | | | | | | |  |  |  |
| A | 0019838 | growth factor binding | EN-124k-90-group16837.gs\_15971 | | | | | | | | | | | | | | |  |  |  |
| A | 0019838 | growth factor binding | EN-124k-90-group16910.gs\_22871 | | | | | | | | | | | | | | |  |  |  |
| A | 0019838 | growth factor binding | EN-124k-90-group16929.gs\_43290 | | | | | | | | | | | | | | |  |  |  |
| A | 0019838 | growth factor binding | EN-124k-90-group16932.gs\_197 | | | | | | | | | | | | | | |  |  |  |
| A | 0019838 | growth factor binding | EN-124k-90-group17012.gs\_52263 | | | | | | | | | | | | | | |  |  |  |
| A | 0019838 | growth factor binding | EN-124k-90-group17386.gs\_68411 | | | | | | | | | | | | | | |  |  |  |
| A | 0019838 | growth factor binding | EN-124k-90-group17442.gs\_63748 | | | | | | | | | | | | | | |  |  |  |
| A | 0019838 | growth factor binding | EN-124k-90-group17524.gs\_80802 | | | | | | | | | | | | | | |  |  |  |
| A | 0019838 | growth factor binding | EN-124k-90-group17702.gs\_18827 | | | | | | | | | | | | | | |  |  |  |
| A | 0019838 | growth factor binding | EN-124k-90-group17930.gs\_83513 | | | | | | | | | | | | | | |  |  |  |
| E | 0019838 | growth factor binding | EN-124k-90-group17979.jgi\_contig\_JGI\_CBBP2421\_fwd | | | | | | | | | | | | | | | | | |
| E | 0019838 | growth factor binding | EN-124k-90-group18538.jgi\_paired\_JGI\_CBBP19133\_rev | | | | | | | | | | | | | | | | | |
| A | 0019838 | growth factor binding | EN-124k-90-group18634.gs\_80302 | | | | | | | | | | | | | | |  |  |  |
| M | 0019838 | growth factor binding | EN-124k-90-group138.Contig1 | | | | | | | | | | | | | |  |  |  |  |
| M | 0019838 | growth factor binding | EN-124k-90-group138.Contig62 | | | | | | | | | | | | | |  |  |  |  |
| M | 0019838 | growth factor binding | EN-124k-90-group229.Contig1 | | | | | | | | | | | | | |  |  |  |  |
| M | 0019838 | growth factor binding | EN-124k-90-group230.Contig1 | | | | | | | | | | | | | |  |  |  |  |
| M | 0019838 | growth factor binding | EN-124k-90-group251.Contig1 | | | | | | | | | | | | | |  |  |  |  |
| M | 0019838 | growth factor binding | EN-124k-90-group324.Contig1 | | | | | | | | | | | | | |  |  |  |  |
| M | 0019838 | growth factor binding | EN-124k-90-group481.Contig4 | | | | | | | | | | | | | |  |  |  |  |
| M | 0019838 | growth factor binding | EN-124k-90-group518.Contig2 | | | | | | | | | | | | | |  |  |  |  |
| M | 0019838 | growth factor binding | EN-124k-90-group550.Contig2 | | | | | | | | | | | | | |  |  |  |  |
| M | 0019838 | growth factor binding | EN-124k-90-group630.Contig2 | | | | | | | | | | | | | |  |  |  |  |
| M | 0019838 | growth factor binding | EN-124k-90-group658.Contig3 | | | | | | | | | | | | | |  |  |  |  |
| M | 0019838 | growth factor binding | EN-124k-90-group682.Contig1 | | | | | | | | | | | | | |  |  |  |  |
| M | 0019838 | growth factor binding | EN-124k-90-group682.Contig2 | | | | | | | | | | | | | |  |  |  |  |
| M | 0019838 | growth factor binding | EN-124k-90-group689.Contig4 | | | | | | | | | | | | | |  |  |  |  |
| M | 0019838 | growth factor binding | EN-124k-90-group727.Contig5 | | | | | | | | | | | | | |  |  |  |  |
| M | 0019838 | growth factor binding | EN-124k-90-group735.Contig1 | | | | | | | | | | | | | |  |  |  |  |
| M | 0019838 | growth factor binding | EN-124k-90-group881.Contig2 | | | | | | | | | | | | | |  |  |  |  |
| M | 0019838 | growth factor binding | EN-124k-90-group881.Contig3 | | | | | | | | | | | | | |  |  |  |  |
| M | 0019838 | growth factor binding | EN-124k-90-group900.Contig1 | | | | | | | | | | | | | |  |  |  |  |
| M | 0019838 | growth factor binding | EN-124k-90-group945.Contig1 | | | | | | | | | | | | | |  |  |  |  |
| M | 0019838 | growth factor binding | EN-124k-90-group1034.Contig1 | | | | | | | | | | | | | |  |  |  |  |
| M | 0019838 | growth factor binding | EN-124k-90-group1071.Contig3 | | | | | | | | | | | | | |  |  |  |  |
| M | 0019838 | growth factor binding | EN-124k-90-group1106.Contig1 | | | | | | | | | | | | | |  |  |  |  |
| M | 0019838 | growth factor binding | EN-124k-90-group1167.Contig3 | | | | | | | | | | | | | |  |  |  |  |
| M | 0019838 | growth factor binding | EN-124k-90-group1304.Contig2 | | | | | | | | | | | | | |  |  |  |  |
| M | 0019838 | growth factor binding | EN-124k-90-group1304.Contig4 | | | | | | | | | | | | | |  |  |  |  |
| M | 0019838 | growth factor binding | EN-124k-90-group1304.Contig8 | | | | | | | | | | | | | |  |  |  |  |
| M | 0019838 | growth factor binding | EN-124k-90-group1456.Contig8 | | | | | | | | | | | | | |  |  |  |  |
| M | 0019838 | growth factor binding | EN-124k-90-group1728.Contig2 | | | | | | | | | | | | | |  |  |  |  |
| M | 0019838 | growth factor binding | EN-124k-90-group1735.Contig6 | | | | | | | | | | | | | |  |  |  |  |
| M | 0019838 | growth factor binding | EN-124k-90-group1782.Contig1 | | | | | | | | | | | | | |  |  |  |  |
| M | 0019838 | growth factor binding | EN-124k-90-group1909.Contig2 | | | | | | | | | | | | | |  |  |  |  |
| M | 0019838 | growth factor binding | EN-124k-90-group1909.Contig3 | | | | | | | | | | | | | |  |  |  |  |
| M | 0019838 | growth factor binding | EN-124k-90-group1915.Contig1 | | | | | | | | | | | | | |  |  |  |  |
| M | 0019838 | growth factor binding | EN-124k-90-group1960.Contig1 | | | | | | | | | | | | | |  |  |  |  |
| M | 0019838 | growth factor binding | EN-124k-90-group2075.Contig2 | | | | | | | | | | | | | |  |  |  |  |
| M | 0019838 | growth factor binding | EN-124k-90-group2081.Contig1 | | | | | | | | | | | | | |  |  |  |  |
| M | 0019838 | growth factor binding | EN-124k-90-group2081.Contig3 | | | | | | | | | | | | | |  |  |  |  |
| M | 0019838 | growth factor binding | EN-124k-90-group2097.Contig1 | | | | | | | | | | | | | |  |  |  |  |
| M | 0019838 | growth factor binding | EN-124k-90-group2200.Contig1 | | | | | | | | | | | | | |  |  |  |  |
| M | 0019838 | growth factor binding | EN-124k-90-group2428.Contig1 | | | | | | | | | | | | | |  |  |  |  |
| M | 0019838 | growth factor binding | EN-124k-90-group2449.Contig1 | | | | | | | | | | | | | |  |  |  |  |
| M | 0019838 | growth factor binding | EN-124k-90-group2449.Contig2 | | | | | | | | | | | | | |  |  |  |  |
| M | 0019838 | growth factor binding | EN-124k-90-group2491.Contig1 | | | | | | | | | | | | | |  |  |  |  |
| M | 0019838 | growth factor binding | EN-124k-90-group2498.Contig5 | | | | | | | | | | | | | |  |  |  |  |
| M | 0019838 | growth factor binding | EN-124k-90-group2498.Contig7 | | | | | | | | | | | | | |  |  |  |  |
| M | 0019838 | growth factor binding | EN-124k-90-group2606.Contig1 | | | | | | | | | | | | | |  |  |  |  |
| M | 0019838 | growth factor binding | EN-124k-90-group2667.Contig1 | | | | | | | | | | | | | |  |  |  |  |
| M | 0019838 | growth factor binding | EN-124k-90-group2778.Contig1 | | | | | | | | | | | | | |  |  |  |  |
| M | 0019838 | growth factor binding | EN-124k-90-group2845.Contig1 | | | | | | | | | | | | | |  |  |  |  |
| M | 0019838 | growth factor binding | EN-124k-90-group2846.Contig1 | | | | | | | | | | | | | |  |  |  |  |
| M | 0019838 | growth factor binding | EN-124k-90-group2852.Contig1 | | | | | | | | | | | | | |  |  |  |  |
| M | 0019838 | growth factor binding | EN-124k-90-group2917.Contig1 | | | | | | | | | | | | | |  |  |  |  |
| M | 0019838 | growth factor binding | EN-124k-90-group2917.Contig2 | | | | | | | | | | | | | |  |  |  |  |
| M | 0019838 | growth factor binding | EN-124k-90-group2917.Contig3 | | | | | | | | | | | | | |  |  |  |  |
| M | 0019838 | growth factor binding | EN-124k-90-group2917.Contig4 | | | | | | | | | | | | | |  |  |  |  |
| M | 0019838 | growth factor binding | EN-124k-90-group3082.Contig1 | | | | | | | | | | | | | |  |  |  |  |
| M | 0019838 | growth factor binding | EN-124k-90-group3082.Contig2 | | | | | | | | | | | | | |  |  |  |  |
| M | 0019838 | growth factor binding | EN-124k-90-group3223.Contig2 | | | | | | | | | | | | | |  |  |  |  |
| M | 0019838 | growth factor binding | EN-124k-90-group3313.Contig1 | | | | | | | | | | | | | |  |  |  |  |
| M | 0019838 | growth factor binding | EN-124k-90-group3313.Contig3 | | | | | | | | | | | | | |  |  |  |  |
| M | 0019838 | growth factor binding | EN-124k-90-group3315.Contig6 | | | | | | | | | | | | | |  |  |  |  |
| M | 0019838 | growth factor binding | EN-124k-90-group3316.Contig1 | | | | | | | | | | | | | |  |  |  |  |
| M | 0019838 | growth factor binding | EN-124k-90-group3394.Contig1 | | | | | | | | | | | | | |  |  |  |  |
| M | 0019838 | growth factor binding | EN-124k-90-group3394.Contig2 | | | | | | | | | | | | | |  |  |  |  |
| M | 0019838 | growth factor binding | EN-124k-90-group3394.Contig3 | | | | | | | | | | | | | |  |  |  |  |
| M | 0019838 | growth factor binding | EN-124k-90-group3394.Contig4 | | | | | | | | | | | | | |  |  |  |  |
| M | 0019838 | growth factor binding | EN-124k-90-group3479.Contig1 | | | | | | | | | | | | | |  |  |  |  |
| M | 0019838 | growth factor binding | EN-124k-90-group3606.Contig2 | | | | | | | | | | | | | |  |  |  |  |
| M | 0019838 | growth factor binding | EN-124k-90-group3700.Contig1 | | | | | | | | | | | | | |  |  |  |  |
| M | 0019838 | growth factor binding | EN-124k-90-group3830.Contig1 | | | | | | | | | | | | | |  |  |  |  |
| M | 0019838 | growth factor binding | EN-124k-90-group3933.Contig1 | | | | | | | | | | | | | |  |  |  |  |
| M | 0019838 | growth factor binding | EN-124k-90-group4168.Contig1 | | | | | | | | | | | | | |  |  |  |  |
| M | 0019838 | growth factor binding | EN-124k-90-group4168.Contig3 | | | | | | | | | | | | | |  |  |  |  |
| M | 0019838 | growth factor binding | EN-124k-90-group4183.Contig1 | | | | | | | | | | | | | |  |  |  |  |
| M | 0019838 | growth factor binding | EN-124k-90-group4372.Contig1 | | | | | | | | | | | | | |  |  |  |  |
| M | 0019838 | growth factor binding | EN-124k-90-group4410.Contig1 | | | | | | | | | | | | | |  |  |  |  |
| M | 0019838 | growth factor binding | EN-124k-90-group4562.Contig1 | | | | | | | | | | | | | |  |  |  |  |
| M | 0019838 | growth factor binding | EN-124k-90-group4621.Contig2 | | | | | | | | | | | | | |  |  |  |  |
| M | 0019838 | growth factor binding | EN-124k-90-group4753.Contig1 | | | | | | | | | | | | | |  |  |  |  |
| M | 0019838 | growth factor binding | EN-124k-90-group4929.Contig1 | | | | | | | | | | | | | |  |  |  |  |
| M | 0019838 | growth factor binding | EN-124k-90-group4929.Contig2 | | | | | | | | | | | | | |  |  |  |  |
| M | 0019838 | growth factor binding | EN-124k-90-group4944.Contig2 | | | | | | | | | | | | | |  |  |  |  |
| M | 0019838 | growth factor binding | EN-124k-90-group4989.Contig2 | | | | | | | | | | | | | |  |  |  |  |
| M | 0019838 | growth factor binding | EN-124k-90-group5172.Contig1 | | | | | | | | | | | | | |  |  |  |  |
| M | 0019838 | growth factor binding | EN-124k-90-group5173.Contig1 | | | | | | | | | | | | | |  |  |  |  |
| M | 0019838 | growth factor binding | EN-124k-90-group5273.Contig1 | | | | | | | | | | | | | |  |  |  |  |
| M | 0019838 | growth factor binding | EN-124k-90-group5273.Contig2 | | | | | | | | | | | | | |  |  |  |  |
| M | 0019838 | growth factor binding | EN-124k-90-group5273.Contig3 | | | | | | | | | | | | | |  |  |  |  |
| M | 0019838 | growth factor binding | EN-124k-90-group5380.Contig1 | | | | | | | | | | | | | |  |  |  |  |
| M | 0019838 | growth factor binding | EN-124k-90-group5479.Contig1 | | | | | | | | | | | | | |  |  |  |  |
| M | 0019838 | growth factor binding | EN-124k-90-group5607.Contig1 | | | | | | | | | | | | | |  |  |  |  |
| M | 0019838 | growth factor binding | EN-124k-90-group5968.Contig1 | | | | | | | | | | | | | |  |  |  |  |
| M | 0019838 | growth factor binding | EN-124k-90-group6038.Contig1 | | | | | | | | | | | | | |  |  |  |  |
| M | 0019838 | growth factor binding | EN-124k-90-group6204.Contig2 | | | | | | | | | | | | | |  |  |  |  |
| M | 0019838 | growth factor binding | EN-124k-90-group6291.Contig1 | | | | | | | | | | | | | |  |  |  |  |
[truncated: 1,074,960 more chars]
