# Supplementary material for: Construction of a medicinal leech transcriptome database and its application to the identification of leech homologs of neural and innate immune genes
Source: BMC Genomics. 2010 Jun 25;11:407. doi: 10.1186/1471-2164-11-407 (PMC2996935; doi:10.1186/1471-2164-11-407)
Supplement: Additional file 2 — Supplementary Table S2: Complete Set of Neural Transcripts Identified by Gene Ontology Analysis. All transcripts encoding Hirudo proteins with homology to Gene Ontology proteins in neural categories. Transcript IDs are linked to protein sequence alignment summaries provided via the Leechmaster Database http://genomes.ucsd.edu/leechmaster. [file 1471-2164-11-407-S2.HTM]

| Medicinal Leech Transcriptome Database  Supplementary Table 2 | |  |  |  |  |  |  |  |  |  |  |  |  |  |  |  |
|  |  |  |  |  |  |  |  |  |  |  |  |  |  |  |  |  |
| Embryo |  |  |  |  |  |  |  |  |  |  |  |  |  |  |  |  |
| Adult CNS | GO ID | GO Category (neural) | Transcript ID |  |  |  |  |  |  |  |  |  |  |  |  |  |
| Mixed |  |  |  |  |  |  |  |  |  |  |  |  |  |  |  |  |
| E | 0021506 | anterior neuropore closure | EN-124k-90-group11745.EN\_iowa\_9354 | | | | | | | | | | |  |  |  |
| A | 0021506 | anterior neuropore closure | EN-124k-90-group16285.gs\_47129 | | | | | | | | | |  |  |  |  |
| A | 0021506 | anterior neuropore closure | EN-124k-90-group17442.gs\_63748 | | | | | | | | | |  |  |  |  |
| A | 0021506 | anterior neuropore closure | EN-124k-90-group17563.gs\_80410 | | | | | | | | | |  |  |  |  |
| M | 0021506 | anterior neuropore closure | EN-124k-90-group4178.Contig1 | | | | | | | | | |  |  |  |  |
| E | 0055059 | asymmetric neuroblast division | EN-124k-90-group343.jgi\_contig\_JGI\_CBBP19299\_fwd | | | | | | | | | | | | |  |
| E | 0055059 | asymmetric neuroblast division | EN-124k-90-group725.jgi\_paired\_JGI\_CBBP10785\_fwd | | | | | | | | | | | | |  |
| A | 0055059 | asymmetric neuroblast division | EN-124k-90-group807.gs\_71281 | | | | | | | | | |  |  |  |  |
| E | 0055059 | asymmetric neuroblast division | EN-124k-90-group1680.jgi\_contig\_JGI\_CBBP17232\_fwd | | | | | | | | | | | | |  |
| E | 0055059 | asymmetric neuroblast division | EN-124k-90-group1852.jgi\_paired\_JGI\_CBBP10940\_fwd | | | | | | | | | | | | |  |
| E | 0055059 | asymmetric neuroblast division | EN-124k-90-group2530.jgi\_paired\_JGI\_CBBP5537\_fwd | | | | | | | | | | | | |  |
| E | 0055059 | asymmetric neuroblast division | EN-124k-90-group3448.jgi\_paired\_JGI\_CBBP19729\_fwd | | | | | | | | | | | | |  |
| A | 0055059 | asymmetric neuroblast division | EN-124k-90-group3564.gs\_15152 | | | | | | | | | |  |  |  |  |
| A | 0055059 | asymmetric neuroblast division | EN-124k-90-group4315.gs\_20609 | | | | | | | | | |  |  |  |  |
| E | 0055059 | asymmetric neuroblast division | EN-124k-90-group5117.jgi\_paired\_JGI\_CBBP19153\_fwd | | | | | | | | | | | | |  |
| A | 0055059 | asymmetric neuroblast division | EN-124k-90-group5938.gs\_54662 | | | | | | | | | |  |  |  |  |
| E | 0055059 | asymmetric neuroblast division | EN-124k-90-group7594.jgi\_paired\_JGI\_CBBP17768\_fwd | | | | | | | | | | | | |  |
| E | 0055059 | asymmetric neuroblast division | EN-124k-90-group7598.jgi\_unpaired\_JGI\_CBBP18343\_fwd | | | | | | | | | | | | | |
| A | 0055059 | asymmetric neuroblast division | EN-124k-90-group8310.gs\_24913 | | | | | | | | | |  |  |  |  |
| E | 0055059 | asymmetric neuroblast division | EN-124k-90-group8370.jgi\_paired\_JGI\_CBBP17511\_fwd | | | | | | | | | | | | |  |
| A | 0055059 | asymmetric neuroblast division | EN-124k-90-group8977.gs\_49487 | | | | | | | | | |  |  |  |  |
| E | 0055059 | asymmetric neuroblast division | EN-124k-90-group9209.jgi\_paired\_JGI\_CBBP19605\_fwd | | | | | | | | | | | | |  |
| A | 0055059 | asymmetric neuroblast division | EN-124k-90-group9467.gs\_16039 | | | | | | | | | |  |  |  |  |
| A | 0055059 | asymmetric neuroblast division | EN-124k-90-group9714.gs\_64750 | | | | | | | | | |  |  |  |  |
| E | 0055059 | asymmetric neuroblast division | EN-124k-90-group9773.jgi\_paired\_JGI\_CBBP6585\_rev | | | | | | | | | | | | |  |
| A | 0055059 | asymmetric neuroblast division | EN-124k-90-group10312.gs\_22624 | | | | | | | | | |  |  |  |  |
| E | 0055059 | asymmetric neuroblast division | EN-124k-90-group10852.jgi\_paired\_JGI\_CBBP12445\_fwd | | | | | | | | | | | | |  |
| E | 0055059 | asymmetric neuroblast division | EN-124k-90-group11382.jgi\_unpaired\_JGI\_CBBP12542\_fwd | | | | | | | | | | | | | |
| A | 0055059 | asymmetric neuroblast division | EN-124k-90-group11387.gs\_16543 | | | | | | | | | |  |  |  |  |
| E | 0055059 | asymmetric neuroblast division | EN-124k-90-group11559.jgi\_unpaired\_JGI\_CBBP12231\_fwd | | | | | | | | | | | | | |
| E | 0055059 | asymmetric neuroblast division | EN-124k-90-group11653.jgi\_paired\_JGI\_CBBP15394\_fwd | | | | | | | | | | | | |  |
| A | 0055059 | asymmetric neuroblast division | EN-124k-90-group11738.gs\_53113 | | | | | | | | | |  |  |  |  |
| E | 0055059 | asymmetric neuroblast division | EN-124k-90-group12452.EN\_iowa\_9722 | | | | | | | | | | |  |  |  |
| E | 0055059 | asymmetric neuroblast division | EN-124k-90-group12603.jgi\_paired\_JGI\_CBBP6814\_fwd | | | | | | | | | | | | |  |
| E | 0055059 | asymmetric neuroblast division | EN-124k-90-group12657.jgi\_contig\_JGI\_CBBP14885\_fwd | | | | | | | | | | | | |  |
| E | 0055059 | asymmetric neuroblast division | EN-124k-90-group12676.jgi\_paired\_JGI\_CBBP17511\_rev | | | | | | | | | | | | |  |
| E | 0055059 | asymmetric neuroblast division | EN-124k-90-group12744.EN\_iowa\_4256 | | | | | | | | | | |  |  |  |
| E | 0055059 | asymmetric neuroblast division | EN-124k-90-group13261.jgi\_contig\_JGI\_CBBP9652\_fwd | | | | | | | | | | | | |  |
| E | 0055059 | asymmetric neuroblast division | EN-124k-90-group13515.EN\_iowa\_10440 | | | | | | | | | | |  |  |  |
| E | 0055059 | asymmetric neuroblast division | EN-124k-90-group13793.jgi\_paired\_JGI\_CBBP2343\_fwd | | | | | | | | | | | | |  |
| E | 0055059 | asymmetric neuroblast division | EN-124k-90-group13916.EN\_iowa\_14815 | | | | | | | | | | |  |  |  |
| A | 0055059 | asymmetric neuroblast division | EN-124k-90-group13937.gs\_79016 | | | | | | | | | |  |  |  |  |
| A | 0055059 | asymmetric neuroblast division | EN-124k-90-group14128.gs\_38861 | | | | | | | | | |  |  |  |  |
| E | 0055059 | asymmetric neuroblast division | EN-124k-90-group14306.jgi\_paired\_JGI\_CBBP12364\_fwd | | | | | | | | | | | | |  |
| A | 0055059 | asymmetric neuroblast division | EN-124k-90-group14320.gs\_86221 | | | | | | | | | |  |  |  |  |
| A | 0055059 | asymmetric neuroblast division | EN-124k-90-group14351.gs\_85401 | | | | | | | | | |  |  |  |  |
| E | 0055059 | asymmetric neuroblast division | EN-124k-90-group14509.jgi\_paired\_JGI\_CBBP933\_fwd | | | | | | | | | | | | |  |
| A | 0055059 | asymmetric neuroblast division | EN-124k-90-group14558.gs\_28342 | | | | | | | | | |  |  |  |  |
| A | 0055059 | asymmetric neuroblast division | EN-124k-90-group14563.gs\_48486 | | | | | | | | | |  |  |  |  |
| E | 0055059 | asymmetric neuroblast division | EN-124k-90-group14573.jgi\_paired\_JGI\_CBBP20040\_fwd | | | | | | | | | | | | |  |
| A | 0055059 | asymmetric neuroblast division | EN-124k-90-group14609.gs\_33389 | | | | | | | | | |  |  |  |  |
| E | 0055059 | asymmetric neuroblast division | EN-124k-90-group14931.jgi\_paired\_JGI\_CBBP10444\_fwd | | | | | | | | | | | | |  |
| A | 0055059 | asymmetric neuroblast division | EN-124k-90-group15056.gs\_73097 | | | | | | | | | |  |  |  |  |
| A | 0055059 | asymmetric neuroblast division | EN-124k-90-group15229.gs\_75551 | | | | | | | | | |  |  |  |  |
| E | 0055059 | asymmetric neuroblast division | EN-124k-90-group15290.EN\_iowa\_9903 | | | | | | | | | | |  |  |  |
| E | 0055059 | asymmetric neuroblast division | EN-124k-90-group15546.EN\_iowa\_13502 | | | | | | | | | | |  |  |  |
| A | 0055059 | asymmetric neuroblast division | EN-124k-90-group15770.gs\_45569 | | | | | | | | | |  |  |  |  |
| E | 0055059 | asymmetric neuroblast division | EN-124k-90-group15944.jgi\_paired\_JGI\_CBBP5778\_fwd | | | | | | | | | | | | |  |
| E | 0055059 | asymmetric neuroblast division | EN-124k-90-group16200.jgi\_paired\_JGI\_CBBP10444\_rev | | | | | | | | | | | | |  |
| A | 0055059 | asymmetric neuroblast division | EN-124k-90-group16267.gs\_28098 | | | | | | | | | |  |  |  |  |
| A | 0055059 | asymmetric neuroblast division | EN-124k-90-group16416.gs\_28586 | | | | | | | | | |  |  |  |  |
| A | 0055059 | asymmetric neuroblast division | EN-124k-90-group16523.gs\_75670 | | | | | | | | | |  |  |  |  |
| A | 0055059 | asymmetric neuroblast division | EN-124k-90-group16830.gs\_82512 | | | | | | | | | |  |  |  |  |
| A | 0055059 | asymmetric neuroblast division | EN-124k-90-group16971.gs\_60466 | | | | | | | | | |  |  |  |  |
| E | 0055059 | asymmetric neuroblast division | EN-124k-90-group17086.jgi\_paired\_JGI\_CBBP6417\_rev | | | | | | | | | | | | |  |
| E | 0055059 | asymmetric neuroblast division | EN-124k-90-group17091.jgi\_paired\_JGI\_CBBP10179\_rev | | | | | | | | | | | | |  |
| A | 0055059 | asymmetric neuroblast division | EN-124k-90-group17193.gs\_13341 | | | | | | | | | |  |  |  |  |
| A | 0055059 | asymmetric neuroblast division | EN-124k-90-group17442.gs\_63748 | | | | | | | | | |  |  |  |  |
| A | 0055059 | asymmetric neuroblast division | EN-124k-90-group17445.gs\_83255 | | | | | | | | | |  |  |  |  |
| A | 0055059 | asymmetric neuroblast division | EN-124k-90-group17563.gs\_80410 | | | | | | | | | |  |  |  |  |
| A | 0055059 | asymmetric neuroblast division | EN-124k-90-group17582.gs\_70636 | | | | | | | | | |  |  |  |  |
| A | 0055059 | asymmetric neuroblast division | EN-124k-90-group17638.gs\_37176 | | | | | | | | | |  |  |  |  |
| A | 0055059 | asymmetric neuroblast division | EN-124k-90-group17744.gs\_30255 | | | | | | | | | |  |  |  |  |
| A | 0055059 | asymmetric neuroblast division | EN-124k-90-group17864.gs\_35131 | | | | | | | | | |  |  |  |  |
| A | 0055059 | asymmetric neuroblast division | EN-124k-90-group18140.gs\_87168 | | | | | | | | | |  |  |  |  |
| A | 0055059 | asymmetric neuroblast division | EN-124k-90-group18148.gs\_80788 | | | | | | | | | |  |  |  |  |
| A | 0055059 | asymmetric neuroblast division | EN-124k-90-group18480.gs\_449 | | | | | | | | | |  |  |  |  |
| M | 0055059 | asymmetric neuroblast division | EN-124k-90-group10.Contig1 | | | | | | | | |  |  |  |  |  |
| M | 0055059 | asymmetric neuroblast division | EN-124k-90-group21.Contig1 | | | | | | | | |  |  |  |  |  |
| M | 0055059 | asymmetric neuroblast division | EN-124k-90-group163.Contig2 | | | | | | | | | |  |  |  |  |
| M | 0055059 | asymmetric neuroblast division | EN-124k-90-group171.Contig2 | | | | | | | | | |  |  |  |  |
| M | 0055059 | asymmetric neuroblast division | EN-124k-90-group202.Contig2 | | | | | | | | | |  |  |  |  |
| M | 0055059 | asymmetric neuroblast division | EN-124k-90-group229.Contig1 | | | | | | | | | |  |  |  |  |
| M | 0055059 | asymmetric neuroblast division | EN-124k-90-group230.Contig1 | | | | | | | | | |  |  |  |  |
| M | 0055059 | asymmetric neuroblast division | EN-124k-90-group265.Contig1 | | | | | | | | | |  |  |  |  |
| M | 0055059 | asymmetric neuroblast division | EN-124k-90-group324.Contig1 | | | | | | | | | |  |  |  |  |
| M | 0055059 | asymmetric neuroblast division | EN-124k-90-group328.Contig2 | | | | | | | | | |  |  |  |  |
| M | 0055059 | asymmetric neuroblast division | EN-124k-90-group335.Contig1 | | | | | | | | | |  |  |  |  |
| M | 0055059 | asymmetric neuroblast division | EN-124k-90-group335.Contig2 | | | | | | | | | |  |  |  |  |
| A | 0055059 | asymmetric neuroblast division | EN-124k-90-group358.Contig1 | | | | | | | | | |  |  |  |  |
| M | 0055059 | asymmetric neuroblast division | EN-124k-90-group446.Contig4 | | | | | | | | | |  |  |  |  |
| M | 0055059 | asymmetric neuroblast division | EN-124k-90-group462.Contig1 | | | | | | | | | |  |  |  |  |
| M | 0055059 | asymmetric neuroblast division | EN-124k-90-group481.Contig4 | | | | | | | | | |  |  |  |  |
| M | 0055059 | asymmetric neuroblast division | EN-124k-90-group550.Contig1 | | | | | | | | | |  |  |  |  |
| M | 0055059 | asymmetric neuroblast division | EN-124k-90-group550.Contig2 | | | | | | | | | |  |  |  |  |
| M | 0055059 | asymmetric neuroblast division | EN-124k-90-group593.Contig1 | | | | | | | | | |  |  |  |  |
| M | 0055059 | asymmetric neuroblast division | EN-124k-90-group658.Contig1 | | | | | | | | | |  |  |  |  |
| M | 0055059 | asymmetric neuroblast division | EN-124k-90-group658.Contig3 | | | | | | | | | |  |  |  |  |
| M | 0055059 | asymmetric neuroblast division | EN-124k-90-group682.Contig1 | | | | | | | | | |  |  |  |  |
| M | 0055059 | asymmetric neuroblast division | EN-124k-90-group701.Contig1 | | | | | | | | | |  |  |  |  |
| M | 0055059 | asymmetric neuroblast division | EN-124k-90-group701.Contig2 | | | | | | | | | |  |  |  |  |
| M | 0055059 | asymmetric neuroblast division | EN-124k-90-group702.Contig2 | | | | | | | | | |  |  |  |  |
| M | 0055059 | asymmetric neuroblast division | EN-124k-90-group716.Contig1 | | | | | | | | | |  |  |  |  |
| M | 0055059 | asymmetric neuroblast division | EN-124k-90-group820.Contig1 | | | | | | | | | |  |  |  |  |
| M | 0055059 | asymmetric neuroblast division | EN-124k-90-group881.Contig3 | | | | | | | | | |  |  |  |  |
| M | 0055059 | asymmetric neuroblast division | EN-124k-90-group900.Contig1 | | | | | | | | | |  |  |  |  |
| M | 0055059 | asymmetric neuroblast division | EN-124k-90-group945.Contig1 | | | | | | | | | |  |  |  |  |
| M | 0055059 | asymmetric neuroblast division | EN-124k-90-group998.Contig1 | | | | | | | | | |  |  |  |  |
| M | 0055059 | asymmetric neuroblast division | EN-124k-90-group1069.Contig1 | | | | | | | | | |  |  |  |  |
| M | 0055059 | asymmetric neuroblast division | EN-124k-90-group1153.Contig1 | | | | | | | | | |  |  |  |  |
| E | 0055059 | asymmetric neuroblast division | EN-124k-90-group1160.Contig2 | | | | | | | | | |  |  |  |  |
| M | 0055059 | asymmetric neuroblast division | EN-124k-90-group1160.Contig5 | | | | | | | | | |  |  |  |  |
| M | 0055059 | asymmetric neuroblast division | EN-124k-90-group1160.Contig6 | | | | | | | | | |  |  |  |  |
| M | 0055059 | asymmetric neuroblast division | EN-124k-90-group1161.Contig1 | | | | | | | | | |  |  |  |  |
| M | 0055059 | asymmetric neuroblast division | EN-124k-90-group1161.Contig2 | | | | | | | | | |  |  |  |  |
| M | 0055059 | asymmetric neuroblast division | EN-124k-90-group1167.Contig2 | | | | | | | | | |  |  |  |  |
| M | 0055059 | asymmetric neuroblast division | EN-124k-90-group1167.Contig3 | | | | | | | | | |  |  |  |  |
| M | 0055059 | asymmetric neuroblast division | EN-124k-90-group1203.Contig1 | | | | | | | | | |  |  |  |  |
| M | 0055059 | asymmetric neuroblast division | EN-124k-90-group1203.Contig2 | | | | | | | | | |  |  |  |  |
| M | 0055059 | asymmetric neuroblast division | EN-124k-90-group1212.Contig1 | | | | | | | | | |  |  |  |  |
| E | 0055059 | asymmetric neuroblast division | EN-124k-90-group1255.Contig1 | | | | | | | | | |  |  |  |  |
| M | 0055059 | asymmetric neuroblast division | EN-124k-90-group1272.Contig1 | | | | | | | | | |  |  |  |  |
| M | 0055059 | asymmetric neuroblast division | EN-124k-90-group1302.Contig4 | | | | | | | | | |  |  |  |  |
| M | 0055059 | asymmetric neuroblast division | EN-124k-90-group1304.Contig2 | | | | | | | | | |  |  |  |  |
| M | 0055059 | asymmetric neuroblast division | EN-124k-90-group1304.Contig4 | | | | | | | | | |  |  |  |  |
| M | 0055059 | asymmetric neuroblast division | EN-124k-90-group1304.Contig5 | | | | | | | | | |  |  |  |  |
| M | 0055059 | asymmetric neuroblast division | EN-124k-90-group1304.Contig8 | | | | | | | | | |  |  |  |  |
| M | 0055059 | asymmetric neuroblast division | EN-124k-90-group1351.Contig1 | | | | | | | | | |  |  |  |  |
| M | 0055059 | asymmetric neuroblast division | EN-124k-90-group1456.Contig4 | | | | | | | | | |  |  |  |  |
| M | 0055059 | asymmetric neuroblast division | EN-124k-90-group1456.Contig8 | | | | | | | | | |  |  |  |  |
| M | 0055059 | asymmetric neuroblast division | EN-124k-90-group1493.Contig2 | | | | | | | | | |  |  |  |  |
| M | 0055059 | asymmetric neuroblast division | EN-124k-90-group1560.Contig1 | | | | | | | | | |  |  |  |  |
| M | 0055059 | asymmetric neuroblast division | EN-124k-90-group1728.Contig2 | | | | | | | | | |  |  |  |  |
| M | 0055059 | asymmetric neuroblast division | EN-124k-90-group1735.Contig4 | | | | | | | | | |  |  |  |  |
| M | 0055059 | asymmetric neuroblast division | EN-124k-90-group1735.Contig6 | | | | | | | | | |  |  |  |  |
| M | 0055059 | asymmetric neuroblast division | EN-124k-90-group1754.Contig1 | | | | | | | | | |  |  |  |  |
| M | 0055059 | asymmetric neuroblast division | EN-124k-90-group1754.Contig2 | | | | | | | | | |  |  |  |  |
| M | 0055059 | asymmetric neuroblast division | EN-124k-90-group1782.Contig1 | | | | | | | | | |  |  |  |  |
| M | 0055059 | asymmetric neuroblast division | EN-124k-90-group1915.Contig1 | | | | | | | | | |  |  |  |  |
| M | 0055059 | asymmetric neuroblast division | EN-124k-90-group1960.Contig1 | | | | | | | | | |  |  |  |  |
| M | 0055059 | asymmetric neuroblast division | EN-124k-90-group2069.Contig3 | | | | | | | | | |  |  |  |  |
| M | 0055059 | asymmetric neuroblast division | EN-124k-90-group2074.Contig1 | | | | | | | | | |  |  |  |  |
| M | 0055059 | asymmetric neuroblast division | EN-124k-90-group2077.Contig3 | | | | | | | | | |  |  |  |  |
| M | 0055059 | asymmetric neuroblast division | EN-124k-90-group2081.Contig1 | | | | | | | | | |  |  |  |  |
| M | 0055059 | asymmetric neuroblast division | EN-124k-90-group2107.Contig1 | | | | | | | | | |  |  |  |  |
| M | 0055059 | asymmetric neuroblast division | EN-124k-90-group2107.Contig2 | | | | | | | | | |  |  |  |  |
| M | 0055059 | asymmetric neuroblast division | EN-124k-90-group2107.Contig3 | | | | | | | | | |  |  |  |  |
| M | 0055059 | asymmetric neuroblast division | EN-124k-90-group2107.Contig4 | | | | | | | | | |  |  |  |  |
| M | 0055059 | asymmetric neuroblast division | EN-124k-90-group2192.Contig1 | | | | | | | | | |  |  |  |  |
| M | 0055059 | asymmetric neuroblast division | EN-124k-90-group2192.Contig2 | | | | | | | | | |  |  |  |  |
| M | 0055059 | asymmetric neuroblast division | EN-124k-90-group2199.Contig1 | | | | | | | | | |  |  |  |  |
| M | 0055059 | asymmetric neuroblast division | EN-124k-90-group2200.Contig1 | | | | | | | | | |  |  |  |  |
| M | 0055059 | asymmetric neuroblast division | EN-124k-90-group2260.Contig2 | | | | | | | | | |  |  |  |  |
| M | 0055059 | asymmetric neuroblast division | EN-124k-90-group2268.Contig2 | | | | | | | | | |  |  |  |  |
| M | 0055059 | asymmetric neuroblast division | EN-124k-90-group2289.Contig1 | | | | | | | | | |  |  |  |  |
| M | 0055059 | asymmetric neuroblast division | EN-124k-90-group2367.Contig2 | | | | | | | | | |  |  |  |  |
| M | 0055059 | asymmetric neuroblast division | EN-124k-90-group2409.Contig1 | | | | | | | | | |  |  |  |  |
| M | 0055059 | asymmetric neuroblast division | EN-124k-90-group2409.Contig3 | | | | | | | | | |  |  |  |  |
| M | 0055059 | asymmetric neuroblast division | EN-124k-90-group2409.Contig4 | | | | | | | | | |  |  |  |  |
| M | 0055059 | asymmetric neuroblast division | EN-124k-90-group2428.Contig1 | | | | | | | | | |  |  |  |  |
| M | 0055059 | asymmetric neuroblast division | EN-124k-90-group2449.Contig1 | | | | | | | | | |  |  |  |  |
| M | 0055059 | asymmetric neuroblast division | EN-124k-90-group2449.Contig2 | | | | | | | | | |  |  |  |  |
| M | 0055059 | asymmetric neuroblast division | EN-124k-90-group2498.Contig5 | | | | | | | | | |  |  |  |  |
| M | 0055059 | asymmetric neuroblast division | EN-124k-90-group2498.Contig7 | | | | | | | | | |  |  |  |  |
| M | 0055059 | asymmetric neuroblast division | EN-124k-90-group2509.Contig2 | | | | | | | | | |  |  |  |  |
| M | 0055059 | asymmetric neuroblast division | EN-124k-90-group2537.Contig1 | | | | | | | | | |  |  |  |  |
| M | 0055059 | asymmetric neuroblast division | EN-124k-90-group2662.Contig1 | | | | | | | | | |  |  |  |  |
| M | 0055059 | asymmetric neuroblast division | EN-124k-90-group2664.Contig1 | | | | | | | | | |  |  |  |  |
| M | 0055059 | asymmetric neuroblast division | EN-124k-90-group2664.Contig2 | | | | | | | | | |  |  |  |  |
| M | 0055059 | asymmetric neuroblast division | EN-124k-90-group2825.Contig1 | | | | | | | | | |  |  |  |  |
| M | 0055059 | asymmetric neuroblast division | EN-124k-90-group2841.Contig1 | | | | | | | | | |  |  |  |  |
| M | 0055059 | asymmetric neuroblast division | EN-124k-90-group2846.Contig1 | | | | | | | | | |  |  |  |  |
| M | 0055059 | asymmetric neuroblast division | EN-124k-90-group2940.Contig2 | | | | | | | | | |  |  |  |  |
| M | 0055059 | asymmetric neuroblast division | EN-124k-90-group3158.Contig2 | | | | | | | | | |  |  |  |  |
| E | 0055059 | asymmetric neuroblast division | EN-124k-90-group3241.Contig1 | | | | | | | | | |  |  |  |  |
| M | 0055059 | asymmetric neuroblast division | EN-124k-90-group3313.Contig1 | | | | | | | | | |  |  |  |  |
| M | 0055059 | asymmetric neuroblast division | EN-124k-90-group3313.Contig2 | | | | | | | | | |  |  |  |  |
| M | 0055059 | asymmetric neuroblast division | EN-124k-90-group3313.Contig3 | | | | | | | | | |  |  |  |  |
| M | 0055059 | asymmetric neuroblast division | EN-124k-90-group3315.Contig6 | | | | | | | | | |  |  |  |  |
| M | 0055059 | asymmetric neuroblast division | EN-124k-90-group3316.Contig1 | | | | | | | | | |  |  |  |  |
| M | 0055059 | asymmetric neuroblast division | EN-124k-90-group3365.Contig1 | | | | | | | | | |  |  |  |  |
| M | 0055059 | asymmetric neuroblast division | EN-124k-90-group3408.Contig1 | | | | | | | | | |  |  |  |  |
| M | 0055059 | asymmetric neuroblast division | EN-124k-90-group3408.Contig2 | | | | | | | | | |  |  |  |  |
| M | 0055059 | asymmetric neuroblast division | EN-124k-90-group3520.Contig1 | | | | | | | | | |  |  |  |  |
| M | 0055059 | asymmetric neuroblast division | EN-124k-90-group3522.Contig1 | | | | | | | | | |  |  |  |  |
| M | 0055059 | asymmetric neuroblast division | EN-124k-90-group3606.Contig2 | | | | | | | | | |  |  |  |  |
| M | 0055059 | asymmetric neuroblast division | EN-124k-90-group3707.Contig1 | | | | | | | | | |  |  |  |  |
| M | 0055059 | asymmetric neuroblast division | EN-124k-90-group3713.Contig1 | | | | | | | | | |  |  |  |  |
| M | 0055059 | asymmetric neuroblast division | EN-124k-90-group3753.Contig1 | | | | | | | | | |  |  |  |  |
| M | 0055059 | asymmetric neuroblast division | EN-124k-90-group3753.Contig2 | | | | | | | | | |  |  |  |  |
| M | 0055059 | asymmetric neuroblast division | EN-124k-90-group3830.Contig1 | | | | | | | | | |  |  |  |  |
| M | 0055059 | asymmetric neuroblast division | EN-124k-90-group3892.Contig1 | | | | | | | | | |  |  |  |  |
| M | 0055059 | asymmetric neuroblast division | EN-124k-90-group3997.Contig2 | | | | | | | | | |  |  |  |  |
| M | 0055059 | asymmetric neuroblast division | EN-124k-90-group4130.Contig1 | | | | | | | | | |  |  |  |  |
| M | 0055059 | asymmetric neuroblast division | EN-124k-90-group4168.Contig1 | | | | | | | | | |  |  |  |  |
| M | 0055059 | asymmetric neuroblast division | EN-124k-90-group4168.Contig3 | | | | | | | | | |  |  |  |  |
| M | 0055059 | asymmetric neuroblast division | EN-124k-90-group4183.Contig1 | | | | | | | | | |  |  |  |  |
| M | 0055059 | asymmetric neuroblast division | EN-124k-90-group4194.Contig1 | | | | | | | | | |  |  |  |  |
| M | 0055059 | asymmetric neuroblast division | EN-124k-90-group4349.Contig2 | | | | | | | | | |  |  |  |  |
| M | 0055059 | asymmetric neuroblast division | EN-124k-90-group4349.Contig3 | | | | | | | | | |  |  |  |  |
| M | 0055059 | asymmetric neuroblast division | EN-124k-90-group4372.Contig1 | | | | | | | | | |  |  |  |  |
| M | 0055059 | asymmetric neuroblast division | EN-124k-90-group4410.Contig1 | | | | | | | | | |  |  |  |  |
| M | 0055059 | asymmetric neuroblast division | EN-124k-90-group4454.Contig1 | | | | | | | | | |  |  |  |  |
| M | 0055059 | asymmetric neuroblast division | EN-124k-90-group4560.Contig1 | | | | | | | | | |  |  |  |  |
| M | 0055059 | asymmetric neuroblast division | EN-124k-90-group4599.Contig1 | | | | | | | | | |  |  |  |  |
| M | 0055059 | asymmetric neuroblast division | EN-124k-90-group4668.Contig1 | | | | | | | | | |  |  |  |  |
| M | 0055059 | asymmetric neuroblast division | EN-124k-90-group4709.Contig1 | | | | | | | | | |  |  |  |  |
| E | 0055059 | asymmetric neuroblast division | EN-124k-90-group4723.Contig2 | | | | | | | | | |  |  |  |  |
| M | 0055059 | asymmetric neuroblast division | EN-124k-90-group4757.Contig1 | | | | | | | | | |  |  |  |  |
| M | 0055059 | asymmetric neuroblast division | EN-124k-90-group4757.Contig2 | | | | | | | | | |  |  |  |  |
| M | 0055059 | asymmetric neuroblast division | EN-124k-90-group4763.Contig1 | | | | | | | | | |  |  |  |  |
| M | 0055059 | asymmetric neuroblast division | EN-124k-90-group4804.Contig1 | | | | | | | | | |  |  |  |  |
| E | 0055059 | asymmetric neuroblast division | EN-124k-90-group4884.Contig1 | | | | | | | | | |  |  |  |  |
| M | 0055059 | asymmetric neuroblast division | EN-124k-90-group4913.Contig1 | | | | | | | | | |  |  |  |  |
| M | 0055059 | asymmetric neuroblast division | EN-124k-90-group4913.Contig2 | | | | | | | | | |  |  |  |  |
| M | 0055059 | asymmetric neuroblast division | EN-124k-90-group4929.Contig2 | | | | | | | | | |  |  |  |  |
| M | 0055059 | asymmetric neuroblast division | EN-124k-90-group4933.Contig1 | | | | | | | | | |  |  |  |  |
| M | 0055059 | asymmetric neuroblast division | EN-124k-90-group4933.Contig2 | | | | | | | | | |  |  |  |  |
| M | 0055059 | asymmetric neuroblast division | EN-124k-90-group4945.Contig2 | | | | | | | | | |  |  |  |  |
| M | 0055059 | asymmetric neuroblast division | EN-124k-90-group5063.Contig1 | | | | | | | | | |  |  |  |  |
| M | 0055059 | asymmetric neuroblast division | EN-124k-90-group5071.Contig3 | | | | | | | | | |  |  |  |  |
| M | 0055059 | asymmetric neuroblast division | EN-124k-90-group5093.Contig2 | | | | | | | | | |  |  |  |  |
| M | 0055059 | asymmetric neuroblast division | EN-124k-90-group5298.Contig1 | | | | | | | | | |  |  |  |  |
| M | 0055059 | asymmetric neuroblast division | EN-124k-90-group5359.Contig3 | | | | | | | | | |  |  |  |  |
| M | 0055059 | asymmetric neuroblast division | EN-124k-90-group5411.Contig1 | | | | | | | | | |  |  |  |  |
| M | 0055059 | asymmetric neuroblast division | EN-124k-90-group5525.Contig1 | | | | | | | | | |  |  |  |  |
| M | 0055059 | asymmetric neuroblast division | EN-124k-90-group5542.Contig1 | | | | | | | | | |  |  |  |  |
| M | 0055059 | asymmetric neuroblast division | EN-124k-90-group5607.Contig1 | | | | | | | | | |  |  |  |  |
| M | 0055059 | asymmetric neuroblast division | EN-124k-90-group5616.Contig1 | | | | | | | | | |  |  |  |  |
| M | 0055059 | asymmetric neuroblast division | EN-124k-90-group5668.Contig1 | | | | | | | | | |  |  |  |  |
| M | 0055059 | asymmetric neuroblast division | EN-124k-90-group5717.Contig2 | | | | | | | | | |  |  |  |  |
| M | 0055059 | asymmetric neuroblast division | EN-124k-90-group5742.Contig1 | | | | | | | | | |  |  |  |  |
| M | 0055059 | asymmetric neuroblast division | EN-124k-90-group5910.Contig1 | | | | | | | | | |  |  |  |  |
| M | 0055059 | asymmetric neuroblast division | EN-124k-90-group5968.Contig1 | | | | | | | | | |  |  |  |  |
| M | 0055059 | asymmetric neuroblast division | EN-124k-90-group6033.Contig1 | | | | | | | | | |  |  |  |  |
| M | 0055059 | asymmetric neuroblast division | EN-124k-90-group6033.Contig2 | | | | | | | | | |  |  |  |  |
| M | 0055059 | asymmetric neuroblast division | EN-124k-90-group6033.Contig3 | | | | | | | | | |  |  |  |  |
| M | 0055059 | asymmetric neuroblast division | EN-124k-90-group6195.Contig1 | | | | | | | | | |  |  |  |  |
| M | 0055059 | asymmetric neuroblast division | EN-124k-90-group6204.Contig1 | | | | | | | | | |  |  |  |  |
| M | 0055059 | asymmetric neuroblast division | EN-124k-90-group6204.Contig2 | | | | | | | | | |  |  |  |  |
| M | 0055059 | asymmetric neuroblast division | EN-124k-90-group6400.Contig1 | | | | | | | | | |  |  |  |  |
| M | 0055059 | asymmetric neuroblast division | EN-124k-90-group6638.Contig1 | | | | | | | | | |  |  |  |  |
| M | 0055059 | asymmetric neuroblast division | EN-124k-90-group6666.Contig1 | | | | | | | | | |  |  |  |  |
| M | 0055059 | asymmetric neuroblast division | EN-124k-90-group6742.Contig2 | | | | | | | | | |  |  |  |  |
| M | 0055059 | asymmetric neuroblast division | EN-124k-90-group6809.Contig1 | | | | | | | | | |  |  |  |  |
| E | 0055059 | asymmetric neuroblast division | EN-124k-90-group6858.Contig1 | | | | | | | | | |  |  |  |  |
| M | 0055059 | asymmetric neuroblast division | EN-124k-90-group6923.Contig1 | | | | | | | | | |  |  |  |  |
| M | 0055059 | asymmetric neuroblast division | EN-124k-90-group6923.Contig2 | | | | | | | | | |  |  |  |  |
| M | 0055059 | asymmetric neuroblast division | EN-124k-90-group7046.Contig1 | | | | | | | | | |  |  |  |  |
| M | 0055059 | asymmetric neuroblast division | EN-124k-90-group7149.Contig2 | | | | | | | | | |  |  |  |  |
| M | 0055059 | asymmetric neuroblast division | EN-124k-90-group7165.Contig1 | | | | | | | | | |  |  |  |  |
| M | 0055059 | asymmetric neuroblast division | EN-124k-90-group7207.Contig1 | | | | | | | | | |  |  |  |  |
| A | 0055059 | asymmetric neuroblast division | EN-124k-90-group7213.Contig1 | | | | | | | | | |  |  |  |  |
| M | 0055059 | asymmetric neuroblast division | EN-124k-90-group7234.Contig1 | | | | | | | | | |  |  |  |  |
| M | 0055059 | asymmetric neuroblast division | EN-124k-90-group7281.Contig1 | | | | | | | | | |  |  |  |  |
| M | 0055059 | asymmetric neuroblast division | EN-124k-90-group7281.Contig2 | | | | | | | | | |  |  |  |  |
| M | 0055059 | asymmetric neuroblast division | EN-124k-90-group7311.Contig1 | | | | | | | | | |  |  |  |  |
| M | 0055059 | asymmetric neuroblast division | EN-124k-90-group7316.Contig1 | | | | | | | | | |  |  |  |  |
| M | 0055059 | asymmetric neuroblast division | EN-124k-90-group7316.Contig3 | | | | | | | | | |  |  |  |  |
| M | 0055059 | asymmetric neuroblast division | EN-124k-90-group7343.Contig1 | | | | | | | | | |  |  |  |  |
| M | 0055059 | asymmetric neuroblast division | EN-124k-90-group7343.Contig2 | | | | | | | | | |  |  |  |  |
| M | 0055059 | asymmetric neuroblast division | EN-124k-90-group7420.Contig1 | | | | | | | | | |  |  |  |  |
| M | 0055059 | asymmetric neuroblast division | EN-124k-90-group7440.Contig1 | | | | | | | | | |  |  |  |  |
| M | 0055059 | asymmetric neuroblast division | EN-124k-90-group7457.Contig1 | | | | | | | | | |  |  |  |  |
| M | 0055059 | asymmetric neuroblast division | EN-124k-90-group7528.Contig1 | | | | | | | | | |  |  |  |  |
| M | 0055059 | asymmetric neuroblast division | EN-124k-90-group7866.Contig1 | | | | | | | | | |  |  |  |  |
| M | 0055059 | asymmetric neuroblast division | EN-124k-90-group7874.Contig2 | | | | | | | | | |  |  |  |  |
| M | 0055059 | asymmetric neuroblast division | EN-124k-90-group7875.Contig1 | | | | | | | | | |  |  |  |  |
| M | 0055059 | asymmetric neuroblast division | EN-124k-90-group7875.Contig2 | | | | | | | | | |  |  |  |  |
| M | 0055059 | asymmetric neuroblast division | EN-124k-90-group7875.Contig3 | | | | | | | | | |  |  |  |  |
| M | 0055059 | asymmetric neuroblast division | EN-124k-90-group7876.Contig1 | | | | | | | | | |  |  |  |  |
| M | 0055059 | asymmetric neuroblast division | EN-124k-90-group7878.Contig2 | | | | | | | | | |  |  |  |  |
| M | 0055059 | asymmetric neuroblast division | EN-124k-90-group7893.Contig1 | | | | | | | | | |  |  |  |  |
| M | 0055059 | asymmetric neuroblast division | EN-124k-90-group8010.Contig1 | | | | | | | | | |  |  |  |  |
| M | 0055059 | asymmetric neuroblast division | EN-124k-90-group8010.Contig2 | | | | | | | | | |  |  |  |  |
| M | 0055059 | asymmetric neuroblast division | EN-124k-90-group8079.Contig1 | | | | | | | | | |  |  |  |  |
| M | 0055059 | asymmetric neuroblast division | EN-124k-90-group8079.Contig2 | | | | | | | | | |  |  |  |  |
| M | 0055059 | asymmetric neuroblast division | EN-124k-90-group8211.Contig1 | | | | | | | | | |  |  |  |  |
| M | 0055059 | asymmetric neuroblast division | EN-124k-90-group8221.Contig1 | | | | | | | | | |  |  |  |  |
| A | 0055059 | asymmetric neuroblast division | EN-124k-90-group8281.Contig1 | | | | | | | | | |  |  |  |  |
| M | 0055059 | asymmetric neuroblast division | EN-124k-90-group8306.Contig3 | | | | | | | | | |  |  |  |  |
| M | 0055059 | asymmetric neuroblast division | EN-124k-90-group8306.Contig4 | | | | | | | | | |  |  |  |  |
| M | 0055059 | asymmetric neuroblast division | EN-124k-90-group8325.Contig1 | | | | | | | | | |  |  |  |  |
| M | 0055059 | asymmetric neuroblast division | EN-124k-90-group8375.Contig1 | | | | | | | | | |  |  |  |  |
| M | 0055059 | asymmetric neuroblast division | EN-124k-90-group8492.Contig1 | | | | | | | | | |  |  |  |  |
| A | 0055059 | asymmetric neuroblast division | EN-124k-90-group8519.Contig1 | | | | | | | | | |  |  |  |  |
| M | 0055059 | asymmetric neuroblast division | EN-124k-90-group8568.Contig1 | | | | | | | | | |  |  |  |  |
| M | 0055059 | asymmetric neuroblast division | EN-124k-90-group8645.Contig1 | | | | | | | | | |  |  |  |  |
| A | 0055059 | asymmetric neuroblast division | EN-124k-90-group8668.Contig1 | | | | | | | | | |  |  |  |  |
| M | 0055059 | asymmetric neuroblast division | EN-124k-90-group8799.Contig2 | | | | | | | | | |  |  |  |  |
| M | 0055059 | asymmetric neuroblast division | EN-124k-90-group8850.Contig1 | | | | | | | | | |  |  |  |  |
| A | 0055059 | asymmetric neuroblast division | EN-124k-90-group8957.Contig1 | | | | | | | | | |  |  |  |  |
| M | 0055059 | asymmetric neuroblast division | EN-124k-90-group9045.Contig1 | | | | | | | | | |  |  |  |  |
| M | 0055059 | asymmetric neuroblast division | EN-124k-90-group9079.Contig1 | | | | | | | | | |  |  |  |  |
| A | 0055059 | asymmetric neuroblast division | EN-124k-90-group9245.Contig1 | | | | | | | | | |  |  |  |  |
| M | 0055059 | asymmetric neuroblast division | EN-124k-90-group9299.Contig2 | | | | | | | | | |  |  |  |  |
| M | 0055059 | asymmetric neuroblast division | EN-124k-90-group9299.Contig3 | | | | | | | | | |  |  |  |  |
| M | 0055059 | asymmetric neuroblast division | EN-124k-90-group9317.Contig1 | | | | | | | | | |  |  |  |  |
| M | 0055059 | asymmetric neuroblast division | EN-124k-90-group9388.Contig2 | | | | | | | | | |  |  |  |  |
| M | 0055059 | asymmetric neuroblast division | EN-124k-90-group9446.Contig1 | | | | | | | | | |  |  |  |  |
| M | 0055059 | asymmetric neuroblast division | EN-124k-90-group9479.Contig1 | | | | | | | | | |  |  |  |  |
| M | 0055059 | asymmetric neuroblast division | EN-124k-90-group9650.Contig1 | | | | | | | | | |  |  |  |  |
| M | 0055059 | asymmetric neuroblast division | EN-124k-90-group9695.Contig1 | | | | | | | | | |  |  |  |  |
| M | 0055059 | asymmetric neuroblast division | EN-124k-90-group9819.Contig1 | | | | | | | | | |  |  |  |  |
| M | 0055059 | asymmetric neuroblast division | EN-124k-90-group9843.Contig1 | | | | | | | | | |  |  |  |  |
| M | 0055059 | asymmetric neuroblast division | EN-124k-90-group10032.Contig1 | | | | | | | | | |  |  |  |  |
| M | 0055059 | asymmetric neuroblast division | EN-124k-90-group10085.Contig1 | | | | | | | | | |  |  |  |  |
| M | 0055059 | asymmetric neuroblast division | EN-124k-90-group10085.Contig2 | | | | | | | | | |  |  |  |  |
| M | 0055059 | asymmetric neuroblast division | EN-124k-90-group10090.Contig2 | | | | | | | | | |  |  |  |  |
| M | 0055059 | asymmetric neuroblast division | EN-124k-90-group10221.Contig1 | | | | | | | | | |  |  |  |  |
| M | 0055059 | asymmetric neuroblast division | EN-124k-90-group10276.Contig4 | | | | | | | | | |  |  |  |  |
| M | 0055059 | asymmetric neuroblast division | EN-124k-90-group10598.Contig1 | | | | | | | | | |  |  |  |  |
| M | 0055059 | asymmetric neuroblast division | EN-124k-90-group10652.Contig1 | | | | | | | | | |  |  |  |  |
| M | 0055059 | asymmetric neuroblast division | EN-124k-90-group10667.Contig1 | | | | | | | | | |  |  |  |  |
| M | 0055059 | asymmetric neuroblast division | EN-124k-90-group10717.Contig1 | | | | | | | | | |  |  |  |  |
| M | 0055059 | asymmetric neuroblast division | EN-124k-90-group10790.Contig1 | | | | | | | | | |  |  |  |  |
| M | 0055059 | asymmetric neuroblast division | EN-124k-90-group10824.Contig1 | | | | | | | | | |  |  |  |  |
| M | 0055059 | asymmetric neuroblast division | EN-124k-90-group10838.Contig2 | | | | | | | | | |  |  |  |  |
| M | 0055059 | asymmetric neuroblast division | EN-124k-90-group10881.Contig1 | | | | | | | | | |  |  |  |  |
| M | 0055059 | asymmetric neuroblast division | EN-124k-90-group10891.Contig1 | | | | | | | | | |  |  |  |  |
| M | 0055059 | asymmetric neuroblast division | EN-124k-90-group10891.Contig2 | | | | | | | | | |  |  |  |  |
| M | 0055059 | asymmetric neuroblast division | EN-124k-90-group10912.Contig1 | | | | | | | | | |  |  |  |  |
| M | 0055059 | asymmetric neuroblast division | EN-124k-90-group10928.Contig1 | | | | | | | | | |  |  |  |  |
| M | 0055059 | asymmetric neuroblast division | EN-124k-90-group11235.Contig1 | | | | | | | | | |  |  |  |  |
| M | 0055059 | asymmetric neuroblast division | EN-124k-90-group11250.Contig1 | | | | | | | | | |  |  |  |  |
| M | 0055059 | asymmetric neuroblast division | EN-124k-90-group11408.Contig1 | | | | | | | | | |  |  |  |  |
| M | 0055059 | asymmetric neuroblast division | EN-124k-90-group11686.Contig1 | | | | | | | | | |  |  |  |  |
| M | 0055059 | asymmetric neuroblast division | EN-124k-90-group11808.Contig1 | | | | | | | | | |  |  |  |  |
| M | 0055059 | asymmetric neuroblast division | EN-124k-90-group11987.Contig1 | | | | | | | | | |  |  |  |  |
| E | 0055059 | asymmetric neuroblast division | EN-124k-90-group12010.Contig1 | | | | | | | | | |  |  |  |  |
| E | 0055059 | asymmetric neuroblast division | EN-124k-90-group12010.Contig2 | | | | | | | | | |  |  |  |  |
| M | 0055059 | asymmetric neuroblast division | EN-124k-90-group12099.Contig1 | | | | | | | | | |  |  |  |  |
| M | 0055059 | asymmetric neuroblast division | EN-124k-90-group12519.Contig1 | | | | | | | | | |  |  |  |  |
| M | 0055059 | asymmetric neuroblast division | EN-124k-90-group12678.Contig1 | | | | | | | | | |  |  |  |  |
| M | 0055059 | asymmetric neuroblast division | EN-124k-90-group12973.Contig1 | | | | | | | | | |  |  |  |  |
| M | 0055059 | asymmetric neuroblast division | EN-124k-90-group13121.Contig1 | | | | | | | | | |  |  |  |  |
| M | 0055059 | asymmetric neuroblast division | EN-124k-90-group13235.Contig1 | | | | | | | | | |  |  |  |  |
| M | 0055059 | asymmetric neuroblast division | EN-124k-90-group13502.Contig1 | | | | | | | | | |  |  |  |  |
| M | 0055059 | asymmetric neuroblast division | EN-124k-90-group13502.Contig2 | | | | | | | | | |  |  |  |  |
| M | 0055059 | asymmetric neuroblast division | EN-124k-90-group13754.Contig1 | | | | | | | | | |  |  |  |  |
| E | 0055059 | asymmetric neuroblast division | EN-124k-90-group14464.Contig1 | | | | | | | | | |  |  |  |  |
| M | 0055059 | asymmetric neuroblast division | EN-124k-90-group14708.Contig1 | | | | | | | | | |  |  |  |  |
| M | 0055059 | asymmetric neuroblast division | EN-124k-90-group15300.Contig1 | | | | | | | | | |  |  |  |  |
| A | 0055059 | asymmetric neuroblast division | EN-124k-90-group230.gs\_25885 | | | | | | | | | |  |  |  |  |
| A | 0055059 | asymmetric neuroblast division | EN-124k-90-group574.gs\_59096 | | | | | | | | | |  |  |  |  |
| E | 0055059 | asymmetric neuroblast division | EN-124k-90-group701.jgi\_contig\_JGI\_CBBP10955\_fwd | | | | | | | | | | | | |  |
| E | 0055059 | asymmetric neuroblast division | EN-124k-90-group701.jgi\_contig\_JGI\_CBBP5477\_fwd | | | | | | | | | | | | |  |
| E | 0055059 | asymmetric neuroblast division | EN-124k-90-group701.EN\_iowa\_8242 | | | | | | | | | | |  |  |  |
| A | 0055059 | asymmetric neuroblast division | EN-124k-90-group701.gs\_14861 | | | | | | | | | |  |  |  |  |
| E | 0055059 | asymmetric neuroblast division | EN-124k-90-group900.EN\_iowa\_15436 | | | | | | | | | | |  |  |  |
| A | 0055059 | asymmetric neuroblast division | EN-124k-90-group900.gs\_31783 | | | | | | | | | |  |  |  |  |
| A | 0055059 | asymmetric neuroblast division | EN-124k-90-group900.gs\_71447 | | | | | | | | | |  |  |  |  |
| A | 0055059 | asymmetric neuroblast division | EN-124k-90-group1153.gs\_635 | | | | | | | | | |  |  |  |  |
| A | 0055059 | asymmetric neuroblast division | EN-124k-90-group1153.gs\_33239 | | | | | | | | | |  |  |  |  |
| E | 0055059 | asymmetric neuroblast division | EN-124k-90-group1493.jgi\_contig\_JGI\_CBBP6493\_fwd | | | | | | | | | | | | |  |
| E | 0055059 | asymmetric neuroblast division | EN-124k-90-group1560.jgi\_contig\_JGI\_CBBP5129\_fwd | | | | | | | | | | | | |  |
| A | 0055059 | asymmetric neuroblast division | EN-124k-90-group1782.gs\_16663 | | | | | | | | | |  |  |  |  |
| A | 0055059 | asymmetric neuroblast division | EN-124k-90-group2069.gs\_85543 | | | | | | | | | |  |  |  |  |
| A | 0055059 | asymmetric neuroblast division | EN-124k-90-group2069.gs\_63060 | | | | | | | | | |  |  |  |  |
| A | 0055059 | asymmetric neuroblast division | EN-124k-90-group2069.gs\_69693 | | | | | | | | | |  |  |  |  |
| E | 0055059 | asymmetric neuroblast division | EN-124k-90-group2077.jgi\_paired\_JGI\_CBBP13793\_rev | | | | | | | | | | | | |  |
| E | 0055059 | asymmetric neuroblast division | EN-124k-90-group2409.EN\_iowa\_9419 | | | | | | | | | | |  |  |  |
| E | 0055059 | asymmetric neuroblast division | EN-124k-90-group2409.EN\_iowa\_12940 | | | | | | | | | | |  |  |  |
| A | 0055059 | asymmetric neuroblast division | EN-124k-90-group3522.gs\_9768 | | | | | | | | | |  |  |  |  |
| A | 0055059 | asymmetric neuroblast division | EN-124k-90-group3713.gs\_7861 | | | | | | | | | |  |  |  |  |
| A | 0055059 | asymmetric neuroblast division | EN-124k-90-group4349.gs\_42524 | | | | | | | | | |  |  |  |  |
| E | 0055059 | asymmetric neuroblast division | EN-124k-90-group4804.jgi\_contig\_JGI\_CBBP4923\_fwd | | | | | | | | | | | | |  |
| A | 0055059 | asymmetric neuroblast division | EN-124k-90-group4804.gs\_72028 | | | | | | | | | |  |  |  |  |
| E | 0055059 | asymmetric neuroblast division | EN-124k-90-group4945.jgi\_contig\_JGI\_CBBP14472\_fwd | | | | | | | | | | | | |  |
| E | 0055059 | asymmetric neuroblast division | EN-124k-90-group4945.jgi\_contig\_JGI\_CBBP13066\_fwd | | | | | | | | | | | | |  |
| A | 0055059 | asymmetric neuroblast division | EN-124k-90-group4945.gs\_26726 | | | | | | | | | |  |  |  |  |
| E | 0055059 | asymmetric neuroblast division | EN-124k-90-group4945.jgi\_contig\_JGI\_CBBP19558\_fwd | | | | | | | | | | | | |  |
| A | 0055059 | asymmetric neuroblast division | EN-124k-90-group4945.gs\_49527 | | | | | | | | | |  |  |  |  |
| E | 0055059 | asymmetric neuroblast division | EN-124k-90-group5607.jgi\_contig\_JGI\_CBBP445\_fwd | | | | | | | | | | | | |  |
| E | 0055059 | asymmetric neuroblast division | EN-124k-90-group6204.EN\_iowa\_1799 | | | | | | | | | | |  |  |  |
| E | 0055059 | asymmetric neuroblast division | EN-124k-90-group6204.EN\_iowa\_4716 | | | | | | | | | | |  |  |  |
| E | 0055059 | asymmetric neuroblast division | EN-124k-90-group6923.jgi\_contig\_JGI\_CBBP17789\_fwd | | | | | | | | | | | | |  |
| A | 0055059 | asymmetric neuroblast division | EN-124k-90-group7440.gs\_75782 | | | | | | | | | |  |  |  |  |
| E | 0055059 | asymmetric neuroblast division | EN-124k-90-group7875.jgi\_paired\_JGI\_CBBP10776\_rev | | | | | | | | | | | | |  |
| E | 0055059 | asymmetric neuroblast division | EN-124k-90-group8221.jgi\_contig\_JGI\_CBBP18338\_fwd | | | | | | | | | | | | |  |
| A | 0055059 | asymmetric neuroblast division | EN-124k-90-group8306.gs\_61906 | | | | | | | | | |  |  |  |  |
| A | 0055059 | asymmetric neuroblast division | EN-124k-90-group9299.gs\_87064 | | | | | | | | | |  |  |  |  |
| A | 0007414 | axonal defasciculation | EN-124k-90-group13468.gs\_23546 | | | | | | | | | |  |  |  |  |
| E | 0007414 | axonal defasciculation | EN-124k-90-group1177.jgi\_unpaired\_JGI\_CBBP6661\_fwd | | | | | | | | | | | | |  |
| A | 0007414 | axonal defasciculation | EN-124k-90-group3420.gs\_43284 | | | | | | | | | |  |  |  |  |
| A | 0007414 | axonal defasciculation | EN-124k-90-group4707.gs\_46826 | | | | | | | | | |  |  |  |  |
| E | 0007414 | axonal defasciculation | EN-124k-90-group5833.EN\_iowa\_5392 | | | | | | | | | | |  |  |  |
| E | 0007414 | axonal defasciculation | EN-124k-90-group6144.EN\_iowa\_5856 | | | | | | | | | | |  |  |  |
| E | 0007414 | axonal defasciculation | EN-124k-90-group6152.jgi\_paired\_JGI\_CBBP3620\_fwd | | | | | | | | | | | | |  |
| E | 0007414 | axonal defasciculation | EN-124k-90-group7869.jgi\_paired\_JGI\_CBBP11345\_fwd | | | | | | | | | | | | |  |
| E | 0007414 | axonal defasciculation | EN-124k-90-group7940.jgi\_contig\_JGI\_CBBP6088\_fwd | | | | | | | | | | | | |  |
| E | 0007414 | axonal defasciculation | EN-124k-90-group8104.jgi\_paired\_JGI\_CBBP9893\_fwd | | | | | | | | | | | | |  |
| E | 0007414 | axonal defasciculation | EN-124k-90-group8107.jgi\_paired\_JGI\_CBBP6197\_fwd | | | | | | | | | | | | |  |
| A | 0007414 | axonal defasciculation | EN-124k-90-group9059.gs\_31860 | | | | | | | | | |  |  |  |  |
| A | 0007414 | axonal defasciculation | EN-124k-90-group9319.gs\_38649 | | | | | | | | | |  |  |  |  |
| A | 0007414 | axonal defasciculation | EN-124k-90-group9981.gs\_67492 | | | | | | | | | |  |  |  |  |
| E | 0007414 | axonal defasciculation | EN-124k-90-group10538.jgi\_contig\_JGI\_CBBP12785\_fwd | | | | | | | | | | | | |  |
| A | 0007414 | axonal defasciculation | EN-124k-90-group10635.gs\_47150 | | | | | | | | | |  |  |  |  |
| E | 0007414 | axonal defasciculation | EN-124k-90-group10888.jgi\_paired\_JGI\_CBBP10968\_rev | | | | | | | | | | | | |  |
| E | 0007414 | axonal defasciculation | EN-124k-90-group11156.EN\_iowa\_9243 | | | | | | | | | | |  |  |  |
| A | 0007414 | axonal defasciculation | EN-124k-90-group11354.gs\_78099 | | | | | | | | | |  |  |  |  |
| A | 0007414 | axonal defasciculation | EN-124k-90-group12204.gs\_9947 | | | | | | | | | |  |  |  |  |
| E | 0007414 | axonal defasciculation | EN-124k-90-group12537.jgi\_contig\_JGI\_CBBP17605\_fwd | | | | | | | | | | | | |  |
| E | 0007414 | axonal defasciculation | EN-124k-90-group12563.EN\_iowa\_9742 | | | | | | | | | | |  |  |  |
| A | 0007414 | axonal defasciculation | EN-124k-90-group12627.gs\_60273 | | | | | | | | | |  |  |  |  |
| A | 0007414 | axonal defasciculation | EN-124k-90-group12821.gs\_58803 | | | | | | | | | |  |  |  |  |
| A | 0007414 | axonal defasciculation | EN-124k-90-group13006.gs\_33814 | | | | | | | | | |  |  |  |  |
| E | 0007414 | axonal defasciculation | EN-124k-90-group13023.EN\_iowa\_251 | | | | | | | | | | |  |  |  |
| E | 0007414 | axonal defasciculation | EN-124k-90-group13146.jgi\_paired\_JGI\_CBBP17160\_fwd | | | | | | | | | | | | |  |
| E | 0007414 | axonal defasciculation | EN-124k-90-group13346.jgi\_unpaired\_JGI\_CBBP5460\_rev | | | | | | | | | | | | | |
| E | 0007414 | axonal defasciculation | EN-124k-90-group13368.jgi\_unpaired\_JGI\_CBBP5492\_rev | | | | | | | | | | | | | |
| A | 0007414 | axonal defasciculation | EN-124k-90-group13506.gs\_86402 | | | | | | | | | |  |  |  |  |
| A | 0007414 | axonal defasciculation | EN-124k-90-group13823.gs\_32410 | | | | | | | | | |  |  |  |  |
| E | 0007414 | axonal defasciculation | EN-124k-90-group14050.jgi\_paired\_JGI\_CBBP18955\_fwd | | | | | | | | | | | | |  |
| A | 0007414 | axonal defasciculation | EN-124k-90-group14582.gs\_17139 | | | | | | | | | |  |  |  |  |
| E | 0007414 | axonal defasciculation | EN-124k-90-group14826.jgi\_paired\_JGI\_CBBP20200\_fwd | | | | | | | | | | | | |  |
| A | 0007414 | axonal defasciculation | EN-124k-90-group14887.gs\_61232 | | | | | | | | | |  |  |  |  |
| E | 0007414 | axonal defasciculation | EN-124k-90-group14953.EN\_iowa\_3925 | | | | | | | | | | |  |  |  |
| A | 0007414 | axonal defasciculation | EN-124k-90-group15268.gs\_21540 | | | | | | | | | |  |  |  |  |
| E | 0007414 | axonal defasciculation | EN-124k-90-group15671.jgi\_paired\_JGI\_CBBP10266\_fwd | | | | | | | | | | | | |  |
| E | 0007414 | axonal defasciculation | EN-124k-90-group15723.jgi\_paired\_JGI\_CBBP16981\_fwd | | | | | | | | | | | | |  |
| A | 0007414 | axonal defasciculation | EN-124k-90-group15749.gs\_84109 | | | | | | | | | |  |  |  |  |
| A | 0007414 | axonal defasciculation | EN-124k-90-group16026.gs\_15943 | | | | | | | | | |  |  |  |  |
| A | 0007414 | axonal defasciculation | EN-124k-90-group16323.gs\_48303 | | | | | | | | | |  |  |  |  |
| E | 0007414 | axonal defasciculation | EN-124k-90-group16346.jgi\_contig\_JGI\_CBBP6214\_fwd | | | | | | | | | | | | |  |
| A | 0007414 | axonal defasciculation | EN-124k-90-group16412.gs\_64548 | | | | | | | | | |  |  |  |  |
| A | 0007414 | axonal defasciculation | EN-124k-90-group16522.gs\_78634 | | | | | | | | | |  |  |  |  |
| A | 0007414 | axonal defasciculation | EN-124k-90-group16523.gs\_75670 | | | | | | | | | |  |  |  |  |
| A | 0007414 | axonal defasciculation | EN-124k-90-group16598.gs\_3233 | | | | | | | | | |  |  |  |  |
| A | 0007414 | axonal defasciculation | EN-124k-90-group16600.gs\_29512 | | | | | | | | | |  |  |  |  |
| A | 0007414 | axonal defasciculation | EN-124k-90-group16845.gs\_50498 | | | | | | | | | |  |  |  |  |
| A | 0007414 | axonal defasciculation | EN-124k-90-group16851.gs\_64557 | | | | | | | | | |  |  |  |  |
| A | 0007414 | axonal defasciculation | EN-124k-90-group16852.gs\_27372 | | | | | | | | | |  |  |  |  |
| A | 0007414 | axonal defasciculation | EN-124k-90-group17022.gs\_80073 | | | | | | | | | |  |  |  |  |
| A | 0007414 | axonal defasciculation | EN-124k-90-group17117.gs\_84807 | | | | | | | | | |  |  |  |  |
| A | 0007414 | axonal defasciculation | EN-124k-90-group17129.gs\_61289 | | | | | | | | | |  |  |  |  |
| A | 0007414 | axonal defasciculation | EN-124k-90-group17140.gs\_14565 | | | | | | | | | |  |  |  |  |
| A | 0007414 | axonal defasciculation | EN-124k-90-group17210.gs\_64350 | | | | | | | | | |  |  |  |  |
| A | 0007414 | axonal defasciculation | EN-124k-90-group17273.gs\_68617 | | | | | | | | | |  |  |  |  |
| A | 0007414 | axonal defasciculation | EN-124k-90-group17301.gs\_75053 | | | | | | | | | |  |  |  |  |
| A | 0007414 | axonal defasciculation | EN-124k-90-group17321.gs\_60379 | | | | | | | | | |  |  |  |  |
| A | 0007414 | axonal defasciculation | EN-124k-90-group17386.gs\_68411 | | | | | | | | | |  |  |  |  |
| A | 0007414 | axonal defasciculation | EN-124k-90-group17442.gs\_63748 | | | | | | | | | |  |  |  |  |
| A | 0007414 | axonal defasciculation | EN-124k-90-group17567.gs\_58610 | | | | | | | | | |  |  |  |  |
| A | 0007414 | axonal defasciculation | EN-124k-90-group17702.gs\_18827 | | | | | | | | | |  |  |  |  |
| A | 0007414 | axonal defasciculation | EN-124k-90-group17706.gs\_80669 | | | | | | | | | |  |  |  |  |
| A | 0007414 | axonal defasciculation | EN-124k-90-group17743.gs\_53689 | | | | | | | | | |  |  |  |  |
| A | 0007414 | axonal defasciculation | EN-124k-90-group17744.gs\_30255 | | | | | | | | | |  |  |  |  |
| A | 0007414 | axonal defasciculation | EN-124k-90-group17819.gs\_51352 | | | | | | | | | |  |  |  |  |
| A | 0007414 | axonal defasciculation | EN-124k-90-group17887.gs\_80072 | | | | | | | | | |  |  |  |  |
| E | 0007414 | axonal defasciculation | EN-124k-90-group17943.EN\_iowa\_18374 | | | | | | | | | | |  |  |  |
| A | 0007414 | axonal defasciculation | EN-124k-90-group17951.gs\_43129 | | | | | | | | | |  |  |  |  |
| A | 0007414 | axonal defasciculation | EN-124k-90-group18006.gs\_18909 | | | | | | | | | |  |  |  |  |
| A | 0007414 | axonal defasciculation | EN-124k-90-group18025.gs\_40460 | | | | | | | | | |  |  |  |  |
| A | 0007414 | axonal defasciculation | EN-124k-90-group18245.gs\_78006 | | | | | | | | | |  |  |  |  |
| A | 0007414 | axonal defasciculation | EN-124k-90-group18289.gs\_45417 | | | | | | | | | |  |  |  |  |
| A | 0007414 | axonal defasciculation | EN-124k-90-group18372.gs\_75667 | | | | | | | | | |  |  |  |  |
| A | 0007414 | axonal defasciculation | EN-124k-90-group18447.gs\_40187 | | | | | | | | | |  |  |  |  |
| A | 0007414 | axonal defasciculation | EN-124k-90-group18526.gs\_58285 | | | | | | | | | |  |  |  |  |
| A | 0007414 | axonal defasciculation | EN-124k-90-group18634.gs\_80302 | | | | | | | | | |  |  |  |  |
| A | 0007414 | axonal defasciculation | EN-124k-90-group18840.gs\_32731 | | | | | | | | | |  |  |  |  |
| A | 0007414 | axonal defasciculation | EN-124k-90-group18887.gs\_36301 | | | | | | | | | |  |  |  |  |
| A | 0007414 | axonal defasciculation | EN-124k-90-group18929.gs\_13816 | | | | | | | | | |  |  |  |  |
| M | 0007414 | axonal defasciculation | EN-124k-90-group75.Contig2 | | | | | | | | |  |  |  |  |  |
| M | 0007414 | axonal defasciculation | EN-124k-90-group253.Contig1 | | | | | | | | | |  |  |  |  |
| M | 0007414 | axonal defasciculation | EN-124k-90-group583.Contig1 | | | | | | | | | |  |  |  |  |
| M | 0007414 | axonal defasciculation | EN-124k-90-group586.Contig2 | | | | | | | | | |  |  |  |  |
| M | 0007414 | axonal defasciculation | EN-124k-90-group745.Contig1 | | | | | | | | | |  |  |  |  |
| M | 0007414 | axonal defasciculation | EN-124k-90-group745.Contig2 | | | | | | | | | |  |  |  |  |
| M | 0007414 | axonal defasciculation | EN-124k-90-group925.Contig1 | | | | | | | | | |  |  |  |  |
| M | 0007414 | axonal defasciculation | EN-124k-90-group925.Contig2 | | | | | | | | | |  |  |  |  |
| M | 0007414 | axonal defasciculation | EN-124k-90-group925.Contig3 | | | | | | | | | |  |  |  |  |
| M | 0007414 | axonal defasciculation | EN-124k-90-group944.Contig4 | | | | | | | | | |  |  |  |  |
| M | 0007414 | axonal defasciculation | EN-124k-90-group1001.Contig1 | | | | | | | | | |  |  |  |  |
| M | 0007414 | axonal defasciculation | EN-124k-90-group1001.Contig4 | | | | | | | | | |  |  |  |  |
| M | 0007414 | axonal defasciculation | EN-124k-90-group1076.Contig1 | | | | | | | | | |  |  |  |  |
| M | 0007414 | axonal defasciculation | EN-124k-90-group1142.Contig7 | | | | | | | | | |  |  |  |  |
| M | 0007414 | axonal defasciculation | EN-124k-90-group1290.Contig1 | | | | | | | | | |  |  |  |  |
| M | 0007414 | axonal defasciculation | EN-124k-90-group1290.Contig2 | | | | | | | | | |  |  |  |  |
| M | 0007414 | axonal defasciculation | EN-124k-90-group1290.Contig3 | | | | | | | | | |  |  |  |  |
| M | 0007414 | axonal defasciculation | EN-124k-90-group1290.Contig5 | | | | | | | | | |  |  |  |  |
| M | 0007414 | axonal defasciculation | EN-124k-90-group1290.Contig6 | | | | | | | | | |  |  |  |  |
| M | 0007414 | axonal defasciculation | EN-124k-90-group1290.Contig8 | | | | | | | | | |  |  |  |  |
| M | 0007414 | axonal defasciculation | EN-124k-90-group1290.Contig10 | | | | | | | | | |  |  |  |  |
| M | 0007414 | axonal defasciculation | EN-124k-90-group1408.Contig1 | | | | | | | | | |  |  |  |  |
| M | 0007414 | axonal defasciculation | EN-124k-90-group1408.Contig2 | | | | | | | | | |  |  |  |  |
| A | 0007414 | axonal defasciculation | EN-124k-90-group1482.Contig1 | | | | | | | | | |  |  |  |  |
| A | 0007414 | axonal defasciculation | EN-124k-90-group1521.Contig1 | | | | | | | | | |  |  |  |  |
| A | 0007414 | axonal defasciculation | EN-124k-90-group1521.Contig2 | | | | | | | | | |  |  |  |  |
| M | 0007414 | axonal defasciculation | EN-124k-90-group1834.Contig1 | | | | | | | | | |  |  |  |  |
| M | 0007414 | axonal defasciculation | EN-124k-90-group1871.Contig2 | | | | | | | | | |  |  |  |  |
| M | 0007414 | axonal defasciculation | EN-124k-90-group2397.Contig1 | | | | | | | | | |  |  |  |  |
| M | 0007414 | axonal defasciculation | EN-124k-90-group2397.Contig2 | | | | | | | | | |  |  |  |  |
| M | 0007414 | axonal defasciculation | EN-124k-90-group2455.Contig1 | | | | | | | | | |  |  |  |  |
| M | 0007414 | axonal defasciculation | EN-124k-90-group2681.Contig1 | | | | | | | | | |  |  |  |  |
| M | 0007414 | axonal defasciculation | EN-124k-90-group2811.Contig2 | | | | | | | | | |  |  |  |  |
| M | 0007414 | axonal defasciculation | EN-124k-90-group2835.Contig1 | | | | | | | | | |  |  |  |  |
| M | 0007414 | axonal defasciculation | EN-124k-90-group2835.Contig2 | | | | | | | | | |  |  |  |  |
| M | 0007414 | axonal defasciculation | EN-124k-90-group3092.Contig2 | | | | | | | | | |  |  |  |  |
| M | 0007414 | axonal defasciculation | EN-124k-90-group3222.Contig1 | | | | | | | | | |  |  |  |  |
| M | 0007414 | axonal defasciculation | EN-124k-90-group3299.Contig1 | | | | | | | | | |  |  |  |  |
| E | 0007414 | axonal defasciculation | EN-124k-90-group3611.Contig1 | | | | | | | | | |  |  |  |  |
| M | 0007414 | axonal defasciculation | EN-124k-90-group3876.Contig1 | | | | | | | | | |  |  |  |  |
| M | 0007414 | axonal defasciculation | EN-124k-90-group3883.Contig1 | | | | | | | | | |  |  |  |  |
| M | 0007414 | axonal defasciculation | EN-124k-90-group3883.Contig2 | | | | | | | | | |  |  |  |  |
| M | 0007414 | axonal defasciculation | EN-124k-90-group3883.Contig3 | | | | | | | | | |  |  |  |  |
| M | 0007414 | axonal defasciculation | EN-124k-90-group3883.Contig4 | | | | | | | | | |  |  |  |  |
| M | 0007414 | axonal defasciculation | EN-124k-90-group4045.Contig1 | | | | | | | | | |  |  |  |  |
| M | 0007414 | axonal defasciculation | EN-124k-90-group4436.Contig1 | | | | | | | | | |  |  |  |  |
| M | 0007414 | axonal defasciculation | EN-124k-90-group4827.Contig2 | | | | | | | | | |  |  |  |  |
| M | 0007414 | axonal defasciculation | EN-124k-90-group4827.Contig3 | | | | | | | | | |  |  |  |  |
| M | 0007414 | axonal defasciculation | EN-124k-90-group4890.Contig1 | | | | | | | | | |  |  |  |  |
| M | 0007414 | axonal defasciculation | EN-124k-90-group5022.Contig2 | | | | | | | | | |  |  |  |  |
| M | 0007414 | axonal defasciculation | EN-124k-90-group5054.Contig3 | | | | | | | | | |  |  |  |  |
| M | 0007414 | axonal defasciculation | EN-124k-90-group5282.Contig1 | | | | | | | | | |  |  |  |  |
| A | 0007414 | axonal defasciculation | EN-124k-90-group5543.Contig1 | | | | | | | | | |  |  |  |  |
| M | 0007414 | axonal defasciculation | EN-124k-90-group5709.Contig1 | | | | | | | | | |  |  |  |  |
| E | 0007414 | axonal defasciculation | EN-124k-90-group6326.Contig1 | | | | | | | | | |  |  |  |  |
| A | 0007414 | axonal defasciculation | EN-124k-90-group7650.Contig1 | | | | | | | | | |  |  |  |  |
| M | 0007414 | axonal defasciculation | EN-124k-90-group7660.Contig1 | | | | | | | | | |  |  |  |  |
| M | 0007414 | axonal defasciculation | EN-124k-90-group7743.Contig1 | | | | | | | | | |  |  |  |  |
| M | 0007414 | axonal defasciculation | EN-124k-90-group7770.Contig1 | | | | | | | | | |  |  |  |  |
| M | 0007414 | axonal defasciculation | EN-124k-90-group7770.Contig2 | | | | | | | | | |  |  |  |  |
| M | 0007414 | axonal defasciculation | EN-124k-90-group7770.Contig3 | | | | | | | | | |  |  |  |  |
| M | 0007414 | axonal defasciculation | EN-124k-90-group7859.Contig3 | | | | | | | | | |  |  |  |  |
| M | 0007414 | axonal defasciculation | EN-124k-90-group8103.Contig4 | | | | | | | | | |  |  |  |  |
| M | 0007414 | axonal defasciculation | EN-124k-90-group8897.Contig1 | | | | | | | | | |  |  |  |  |
| M | 0007414 | axonal defasciculation | EN-124k-90-group8988.Contig1 | | | | | | | | | |  |  |  |  |
| M | 0007414 | axonal defasciculation | EN-124k-90-group9165.Contig1 | | | | | | | | | |  |  |  |  |
| M | 0007414 | axonal defasciculation | EN-124k-90-group9887.Contig1 | | | | | | | | | |  |  |  |  |
| A | 0007414 | axonal defasciculation | EN-124k-90-group10298.Contig1 | | | | | | | | | |  |  |  |  |
| A | 0007414 | axonal defasciculation | EN-124k-90-group10370.Contig1 | | | | | | | | | |  |  |  |  |
| M | 0007414 | axonal defasciculation | EN-124k-90-group10486.Contig1 | | | | | | | | | |  |  |  |  |
| M | 0007414 | axonal defasciculation | EN-124k-90-group11317.Contig2 | | | | | | | | | |  |  |  |  |
| E | 0007414 | axonal defasciculation | EN-124k-90-group12535.Contig1 | | | | | | | | | |  |  |  |  |
| M | 0007414 | axonal defasciculation | EN-124k-90-group14580.Contig1 | | | | | | | | | |  |  |  |  |
| A | 0007414 | axonal defasciculation | EN-124k-90-group1290.gs\_26142 | | | | | | | | | |  |  |  |  |
| A | 0007414 | axonal defasciculation | EN-124k-90-group1290.gs\_22118 | | | | | | | | | |  |  |  |  |
| A | 0007414 | axonal defasciculation | EN-124k-90-group1290.gs\_35147 | | | | | | | | | |  |  |  |  |
| A | 0007414 | axonal defasciculation | EN-124k-90-group1290.gs\_13448 | | | | | | | | | |  |  |  |  |
| A | 0007414 | axonal defasciculation | EN-124k-90-group1290.gs\_16770 | | | | | | | | | |  |  |  |  |
| E | 0007414 | axonal defasciculation | EN-124k-90-group1290.EN\_iowa\_15637 | | | | | | | | | | |  |  |  |
| A | 0007414 | axonal defasciculation | EN-124k-90-group1290.gs\_28522 | | | | | | | | | |  |  |  |  |
| A | 0007414 | axonal defasciculation | EN-124k-90-group1290.gs\_84561 | | | | | | | | | |  |  |  |  |
| A | 0007414 | axonal defasciculation | EN-124k-90-group1408.gs\_20773 | | | | | | | | | |  |  |  |  |
| A | 0007414 | axonal defasciculation | EN-124k-90-group1521.gs\_86350 | | | | | | | | | |  |  |  |  |
| A | 0007414 | axonal defasciculation | EN-124k-90-group1871.gs\_37528 | | | | | | | | | |  |  |  |  |
| A | 0007414 | axonal defasciculation | EN-124k-90-group2811.gs\_23030 | | | | | | | | | |  |  |  |  |
| A | 0007414 | axonal defasciculation | EN-124k-90-group2811.gs\_38224 | | | | | | | | | |  |  |  |  |
| A | 0007414 | axonal defasciculation | EN-124k-90-group2811.gs\_15146 | | | | | | | | | |  |  |  |  |
| E | 0007414 | axonal defasciculation | EN-124k-90-group3611.EN\_iowa\_4463 | | | | | | | | | | |  |  |  |
| A | 0007414 | axonal defasciculation | EN-124k-90-group3883.gs\_85360 | | | | | | | | | |  |  |  |  |
| A | 0007414 | axonal defasciculation | EN-124k-90-group7650.gs\_30549 | | | | | | | | | |  |  |  |  |
| A | 0007414 | axonal defasciculation | EN-124k-90-group7650.gs\_59944 | | | | | | | | | |  |  |  |  |
| A | 0007413 | axonal fasciculation | EN-124k-90-group10674.gs\_45324 | | | | | | | | | |  |  |  |  |
| A | 0007413 | axonal fasciculation | EN-124k-90-group807.gs\_71281 | | | | | | | | | |  |  |  |  |
| A | 0007413 | axonal fasciculation | EN-124k-90-group1020.gs\_69762 | | | | | | | | | |  |  |  |  |
| E | 0007413 | axonal fasciculation | EN-124k-90-group1177.jgi\_unpaired\_JGI\_CBBP6661\_fwd | | | | | | | | | | | | |  |
| E | 0007413 | axonal fasciculation | EN-124k-90-group1306.EN\_iowa\_2354 | | | | | | | | | | |  |  |  |
| E | 0007413 | axonal fasciculation | EN-124k-90-group1825.jgi\_contig\_JGI\_CBBP14395\_fwd | | | | | | | | | | | | |  |
| E | 0007413 | axonal fasciculation | EN-124k-90-group2916.jgi\_paired\_JGI\_CBBP2065\_fwd | | | | | | | | | | | | |  |
| A | 0007413 | axonal fasciculation | EN-124k-90-group2932.gs\_32728 | | | | | | | | | |  |  |  |  |
| A | 0007413 | axonal fasciculation | EN-124k-90-group3420.gs\_43284 | | | | | | | | | |  |  |  |  |
| E | 0007413 | axonal fasciculation | EN-124k-90-group3448.jgi\_paired\_JGI\_CBBP19729\_fwd | | | | | | | | | | | | |  |
| A | 0007413 | axonal fasciculation | EN-124k-90-group3807.gs\_52991 | | | | | | | | | |  |  |  |  |
| E | 0007413 | axonal fasciculation | EN-124k-90-group5117.jgi\_paired\_JGI\_CBBP19153\_fwd | | | | | | | | | | | | |  |
| A | 0007413 | axonal fasciculation | EN-124k-90-group5423.gs\_20381 | | | | | | | | | |  |  |  |  |
| E | 0007413 | axonal fasciculation | EN-124k-90-group5834.jgi\_contig\_JGI\_CBBP12701\_fwd | | | | | | | | | | | | |  |
| E | 0007413 | axonal fasciculation | EN-124k-90-group6116.EN\_iowa\_7333 | | | | | | | | | | |  |  |  |
| E | 0007413 | axonal fasciculation | EN-124k-90-group6144.EN\_iowa\_5856 | | | | | | | | | | |  |  |  |
| E | 0007413 | axonal fasciculation | EN-124k-90-group6737.jgi\_contig\_JGI\_CBBP17820\_fwd | | | | | | | | | | | | |  |
| E | 0007413 | axonal fasciculation | EN-124k-90-group6849.EN\_iowa\_8480 | | | | | | | | | | |  |  |  |
| A | 0007413 | axonal fasciculation | EN-124k-90-group7595.gs\_36261 | | | | | | | | | |  |  |  |  |
| A | 0007413 | axonal fasciculation | EN-124k-90-group7953.gs\_11284 | | | | | | | | | |  |  |  |  |
| E | 0007413 | axonal fasciculation | EN-124k-90-group8104.jgi\_paired\_JGI\_CBBP9893\_fwd | | | | | | | | | | | | |  |
| E | 0007413 | axonal fasciculation | EN-124k-90-group8107.jgi\_paired\_JGI\_CBBP6197\_fwd | | | | | | | | | | | | |  |
| A | 0007413 | axonal fasciculation | EN-124k-90-group8310.gs\_24913 | | | | | | | | | |  |  |  |  |
| A | 0007413 | axonal fasciculation | EN-124k-90-group8658.gs\_22315 | | | | | | | | | |  |  |  |  |
| E | 0007413 | axonal fasciculation | EN-124k-90-group8721.jgi\_paired\_JGI\_CBBP11443\_rev | | | | | | | | | | | | |  |
| E | 0007413 | axonal fasciculation | EN-124k-90-group9286.jgi\_paired\_JGI\_CBBP4063\_fwd | | | | | | | | | | | | |  |
| A | 0007413 | axonal fasciculation | EN-124k-90-group9442.gs\_10851 | | | | | | | | | |  |  |  |  |
| E | 0007413 | axonal fasciculation | EN-124k-90-group9448.jgi\_paired\_JGI\_CBBP4366\_rev | | | | | | | | | | | | |  |
| A | 0007413 | axonal fasciculation | EN-124k-90-group9467.gs\_16039 | | | | | | | | | |  |  |  |  |
| A | 0007413 | axonal fasciculation | EN-124k-90-group9657.gs\_43696 | | | | | | | | | |  |  |  |  |
| E | 0007413 | axonal fasciculation | EN-124k-90-group9773.jgi\_paired\_JGI\_CBBP6585\_rev | | | | | | | | | | | | |  |
| E | 0007413 | axonal fasciculation | EN-124k-90-group9900.jgi\_paired\_JGI\_CBBP17759\_fwd | | | | | | | | | | | | |  |
| E | 0007413 | axonal fasciculation | EN-124k-90-group10230.jgi\_paired\_JGI\_CBBP3918\_fwd | | | | | | | | | | | | |  |
| A | 0007413 | axonal fasciculation | EN-124k-90-group10312.gs\_22624 | | | | | | | | | |  |  |  |  |
| E | 0007413 | axonal fasciculation | EN-124k-90-group10416.jgi\_contig\_JGI\_CBBP3274\_fwd | | | | | | | | | | | | |  |
| A | 0007413 | axonal fasciculation | EN-124k-90-group10635.gs\_47150 | | | | | | | | | |  |  |  |  |
| E | 0007413 | axonal fasciculation | EN-124k-90-group10878.jgi\_paired\_JGI\_CBBP952\_rev | | | | | | | | | | | | |  |
| A | 0007413 | axonal fasciculation | EN-124k-90-group10935.gs\_64995 | | | | | | | | | |  |  |  |  |
| E | 0007413 | axonal fasciculation | EN-124k-90-group11108.jgi\_contig\_JGI\_CBBP10361\_fwd | | | | | | | | | | | | |  |
| E | 0007413 | axonal fasciculation | EN-124k-90-group11156.EN\_iowa\_9243 | | | | | | | | | | |  |  |  |
| A | 0007413 | axonal fasciculation | EN-124k-90-group11354.gs\_78099 | | | | | | | | | |  |  |  |  |
| A | 0007413 | axonal fasciculation | EN-124k-90-group11387.gs\_16543 | | | | | | | | | |  |  |  |  |
| E | 0007413 | axonal fasciculation | EN-124k-90-group11469.jgi\_paired\_JGI\_CBBP7559\_fwd | | | | | | | | | | | | |  |
| E | 0007413 | axonal fasciculation | EN-124k-90-group11545.jgi\_paired\_JGI\_CBBP2808\_fwd | | | | | | | | | | | | |  |
| E | 0007413 | axonal fasciculation | EN-124k-90-group11647.EN\_iowa\_13292 | | | | | | | | | | |  |  |  |
| A | 0007413 | axonal fasciculation | EN-124k-90-group11738.gs\_53113 | | | | | | | | | |  |  |  |  |
| E | 0007413 | axonal fasciculation | EN-124k-90-group11780.jgi\_paired\_JGI\_CBBP12332\_fwd | | | | | | | | | | | | |  |
| E | 0007413 | axonal fasciculation | EN-124k-90-group12012.jgi\_contig\_JGI\_CBBP10480\_fwd | | | | | | | | | | | | |  |
| E | 0007413 | axonal fasciculation | EN-124k-90-group12452.EN\_iowa\_9722 | | | | | | | | | | |  |  |  |
| E | 0007413 | axonal fasciculation | EN-124k-90-group12523.jgi\_unpaired\_JGI\_CBBP6872\_fwd | | | | | | | | | | | | | |
| E | 0007413 | axonal fasciculation | EN-124k-90-group12537.jgi\_contig\_JGI\_CBBP17605\_fwd | | | | | | | | | | | | |  |
| E | 0007413 | axonal fasciculation | EN-124k-90-group12595.jgi\_contig\_JGI\_CBBP15955\_fwd | | | | | | | | | | | | |  |
| A | 0007413 | axonal fasciculation | EN-124k-90-group12740.gs\_56121 | | | | | | | | | |  |  |  |  |
| A | 0007413 | axonal fasciculation | EN-124k-90-group12946.gs\_86900 | | | | | | | | | |  |  |  |  |
| A | 0007413 | axonal fasciculation | EN-124k-90-group13006.gs\_33814 | | | | | | | | | |  |  |  |  |
| E | 0007413 | axonal fasciculation | EN-124k-90-group13146.jgi\_paired\_JGI\_CBBP17160\_fwd | | | | | | | | | | | | |  |
| A | 0007413 | axonal fasciculation | EN-124k-90-group13187.gs\_23330 | | | | | | | | | |  |  |  |  |
| E | 0007413 | axonal fasciculation | EN-124k-90-group13466.jgi\_paired\_JGI\_CBBP5821\_fwd | | | | | | | | | | | | |  |
| A | 0007413 | axonal fasciculation | EN-124k-90-group13506.gs\_86402 | | | | | | | | | |  |  |  |  |
| A | 0007413 | axonal fasciculation | EN-124k-90-group13537.gs\_73717 | | | | | | | | | |  |  |  |  |
| E | 0007413 | axonal fasciculation | EN-124k-90-group13736.jgi\_paired\_JGI\_CBBP15489\_rev | | | | | | | | | | | | |  |
| E | 0007413 | axonal fasciculation | EN-124k-90-group13770.jgi\_paired\_JGI\_CBBP10036\_fwd | | | | | | | | | | | | |  |
| A | 0007413 | axonal fasciculation | EN-124k-90-group13777.gs\_40847 | | | | | | | | | |  |  |  |  |
| A | 0007413 | axonal fasciculation | EN-124k-90-group13823.gs\_32410 | | | | | | | | | |  |  |  |  |
| A | 0007413 | axonal fasciculation | EN-124k-90-group13900.gs\_16949 | | | | | | | | | |  |  |  |  |
| A | 0007413 | axonal fasciculation | EN-124k-90-group14128.gs\_38861 | | | | | | | | | |  |  |  |  |
| E | 0007413 | axonal fasciculation | EN-124k-90-group14335.jgi\_paired\_JGI\_CBBP12938\_fwd | | | | | | | | | | | | |  |
| A | 0007413 | axonal fasciculation | EN-124k-90-group14336.gs\_71984 | | | | | | | | | |  |  |  |  |
| A | 0007413 | axonal fasciculation | EN-124k-90-group14498.gs\_52406 | | | | | | | | | |  |  |  |  |
| A | 0007413 | axonal fasciculation | EN-124k-90-group14558.gs\_28342 | | | | | | | | | |  |  |  |  |
| A | 0007413 | axonal fasciculation | EN-124k-90-group14563.gs\_48486 | | | | | | | | | |  |  |  |  |
| A | 0007413 | axonal fasciculation | EN-124k-90-group14582.gs\_17139 | | | | | | | | | |  |  |  |  |
| E | 0007413 | axonal fasciculation | EN-124k-90-group14826.jgi\_paired\_JGI\_CBBP20200\_fwd | | | | | | | | | | | | |  |
| A | 0007413 | axonal fasciculation | EN-124k-90-group14891.gs\_33740 | | | | | | | | | |  |  |  |  |
| A | 0007413 | axonal fasciculation | EN-124k-90-group14896.gs\_55224 | | | | | | | | | |  |  |  |  |
| A | 0007413 | axonal fasciculation | EN-124k-90-group14948.gs\_26769 | | | | | | | | | |  |  |  |  |
| E | 0007413 | axonal fasciculation | EN-124k-90-group14953.EN\_iowa\_3925 | | | | | | | | | | |  |  |  |
| E | 0007413 | axonal fasciculation | EN-124k-90-group14960.jgi\_paired\_JGI\_CBBP2642\_fwd | | | | | | | | | | | | |  |
| A | 0007413 | axonal fasciculation | EN-124k-90-group15062.gs\_40000 | | | | | | | | | |  |  |  |  |
| A | 0007413 | axonal fasciculation | EN-124k-90-group15124.gs\_82644 | | | | | | | | | |  |  |  |  |
| A | 0007413 | axonal fasciculation | EN-124k-90-group15268.gs\_21540 | | | | | | | | | |  |  |  |  |
| E | 0007413 | axonal fasciculation | EN-124k-90-group15315.EN\_iowa\_6410 | | | | | | | | | | |  |  |  |
| E | 0007413 | axonal fasciculation | EN-124k-90-group15320.jgi\_paired\_JGI\_CBBP9817\_fwd | | | | | | | | | | | | |  |
| E | 0007413 | axonal fasciculation | EN-124k-90-group15486.jgi\_paired\_JGI\_CBBP15354\_fwd | | | | | | | | | | | | |  |
| E | 0007413 | axonal fasciculation | EN-124k-90-group15532.EN\_iowa\_5883 | | | | | | | | | | |  |  |  |
| A | 0007413 | axonal fasciculation | EN-124k-90-group15589.gs\_17337 | | | | | | | | | |  |  |  |  |
| E | 0007413 | axonal fasciculation | EN-124k-90-group15692.jgi\_contig\_JGI\_CBBP12510\_fwd | | | | | | | | | | | | |  |
| E | 0007413 | axonal fasciculation | EN-124k-90-group15723.jgi\_paired\_JGI\_CBBP16981\_fwd | | | | | | | | | | | | |  |
| A | 0007413 | axonal fasciculation | EN-124k-90-group15770.gs\_45569 | | | | | | | | | |  |  |  |  |
| A | 0007413 | axonal fasciculation | EN-124k-90-group15856.gs\_15241 | | | | | | | | | |  |  |  |  |
| E | 0007413 | axonal fasciculation | EN-124k-90-group15940.jgi\_contig\_JGI\_CBBP11433\_fwd | | | | | | | | | | | | |  |
| A | 0007413 | axonal fasciculation | EN-124k-90-group16026.gs\_15943 | | | | | | | | | |  |  |  |  |
| A | 0007413 | axonal fasciculation | EN-124k-90-group16087.gs\_81197 | | | | | | | | | |  |  |  |  |
| E | 0007413 | axonal fasciculation | EN-124k-90-group16134.jgi\_contig\_JGI\_CBBP14519\_fwd | | | | | | | | | | | | |  |
| A | 0007413 | axonal fasciculation | EN-124k-90-group16149.gs\_13439 | | | | | | | | | |  |  |  |  |
| A | 0007413 | axonal fasciculation | EN-124k-90-group16189.gs\_70159 | | | | | | | | | |  |  |  |  |
| E | 0007413 | axonal fasciculation | EN-124k-90-group16200.jgi\_paired\_JGI\_CBBP10444\_rev | | | | | | | | | | | | |  |
| E | 0007413 | axonal fasciculation | EN-124k-90-group16264.jgi\_paired\_JGI\_CBBP10036\_rev | | | | | | | | | | | | |  |
| A | 0007413 | axonal fasciculation | EN-124k-90-group16285.gs\_47129 | | | | | | | | | |  |  |  |  |
| A | 0007413 | axonal fasciculation | EN-124k-90-group16291.gs\_65588 | | | | | | | | | |  |  |  |  |
| E | 0007413 | axonal fasciculation | EN-124k-90-group16346.jgi\_contig\_JGI\_CBBP6214\_fwd | | | | | | | | | | | | |  |
| A | 0007413 | axonal fasciculation | EN-124k-90-group16416.gs\_28586 | | | | | | | | | |  |  |  |  |
| A | 0007413 | axonal fasciculation | EN-124k-90-group16423.gs\_20626 | | | | | | | | | |  |  |  |  |
| A | 0007413 | axonal fasciculation | EN-124k-90-group16443.gs\_57863 | | | | | | | | | |  |  |  |  |
| A | 0007413 | axonal fasciculation | EN-124k-90-group16489.gs\_80464 | | | | | | | | | |  |  |  |  |
| A | 0007413 | axonal fasciculation | EN-124k-90-group16520.gs\_62867 | | | | | | | | | |  |  |  |  |
| A | 0007413 | axonal fasciculation | EN-124k-90-group16523.gs\_75670 | | | | | | | | | |  |  |  |  |
| A | 0007413 | axonal fasciculation | EN-124k-90-group16636.gs\_429 | | | | | | | | | |  |  |  |  |
| E | 0007413 | axonal fasciculation | EN-124k-90-group16800.EN\_iowa\_4640 | | | | | | | | | | |  |  |  |
| A | 0007413 | axonal fasciculation | EN-124k-90-group16830.gs\_82512 | | | | | | | | | |  |  |  |  |
| A | 0007413 | axonal fasciculation | EN-124k-90-group16837.gs\_15971 | | | | | | | | | |  |  |  |  |
| A | 0007413 | axonal fasciculation | EN-124k-90-group16910.gs\_22871 | | | | | | | | | |  |  |  |  |
| A | 0007413 | axonal fasciculation | EN-124k-90-group17193.gs\_13341 | | | | | | | | | |  |  |  |  |
| A | 0007413 | axonal fasciculation | EN-124k-90-group17338.gs\_21850 | | | | | | | | | |  |  |  |  |
| A | 0007413 | axonal fasciculation | EN-124k-90-group17386.gs\_68411 | | | | | | | | | |  |  |  |  |
| A | 0007413 | axonal fasciculation | EN-124k-90-group17411.gs\_13940 | | | | | | | | | |  |  |  |  |
| A | 0007413 | axonal fasciculation | EN-124k-90-group17442.gs\_63748 | | | | | | | | | |  |  |  |  |
| A | 0007413 | axonal fasciculation | EN-124k-90-group17445.gs\_83255 | | | | | | | | | |  |  |  |  |
| A | 0007413 | axonal fasciculation | EN-124k-90-group17487.gs\_82041 | | | | | | | | | |  |  |  |  |
| A | 0007413 | axonal fasciculation | EN-124k-90-group17524.gs\_80802 | | | | | | | | | |  |  |  |  |
| A | 0007413 | axonal fasciculation | EN-124k-90-group17563.gs\_80410 | | | | | | | | | |  |  |  |  |
| A | 0007413 | axonal fasciculation | EN-124k-90-group17572.gs\_7187 | | | | | | | | | |  |  |  |  |
| A | 0007413 | axonal fasciculation | EN-124k-90-group17582.gs\_70636 | | | | | | | | | |  |  |  |  |
| A | 0007413 | axonal fasciculation | EN-124k-90-group17650.gs\_59359 | | | | | | | | | |  |  |  |  |
| A | 0007413 | axonal fasciculation | EN-124k-90-group17702.gs\_18827 | | | | | | | | | |  |  |  |  |
| A | 0007413 | axonal fasciculation | EN-124k-90-group17706.gs\_80669 | | | | | | | | | |  |  |  |  |
| A | 0007413 | axonal fasciculation | EN-124k-90-group17930.gs\_83513 | | | | | | | | | |  |  |  |  |
| E | 0007413 | axonal fasciculation | EN-124k-90-group17979.jgi\_contig\_JGI\_CBBP2421\_fwd | | | | | | | | | | | | |  |
| A | 0007413 | axonal fasciculation | EN-124k-90-group18038.gs\_30262 | | | | | | | | | |  |  |  |  |
| A | 0007413 | axonal fasciculation | EN-124k-90-group18140.gs\_87168 | | | | | | | | | |  |  |  |  |
| A | 0007413 | axonal fasciculation | EN-124k-90-group18238.gs\_39782 | | | | | | | | | |  |  |  |  |
| A | 0007413 | axonal fasciculation | EN-124k-90-group18289.gs\_45417 | | | | | | | | | |  |  |  |  |
| A | 0007413 | axonal fasciculation | EN-124k-90-group18324.gs\_57299 | | | | | | | | | |  |  |  |  |
| A | 0007413 | axonal fasciculation | EN-124k-90-group18342.gs\_65612 | | | | | | | | | |  |  |  |  |
| E | 0007413 | axonal fasciculation | EN-124k-90-group18538.jgi\_paired\_JGI\_CBBP19133\_rev | | | | | | | | | | | | |  |
| A | 0007413 | axonal fasciculation | EN-124k-90-group18592.gs\_31083 | | | | | | | | | |  |  |  |  |
| A | 0007413 | axonal fasciculation | EN-124k-90-group18618.gs\_55759 | | | | | | | | | |  |  |  |  |
| A | 0007413 | axonal fasciculation | EN-124k-90-group18774.gs\_57540 | | | | | | | | | |  |  |  |  |
| A | 0007413 | axonal fasciculation | EN-124k-90-group18929.gs\_13816 | | | | | | | | | |  |  |  |  |
| M | 0007413 | axonal fasciculation | EN-124k-90-group59.Contig1 | | | | | | | | |  |  |  |  |  |
| M | 0007413 | axonal fasciculation | EN-124k-90-group115.Contig1 | | | | | | | | | |  |  |  |  |
| M | 0007413 | axonal fasciculation | EN-124k-90-group115.Contig2 | | | | | | | | | |  |  |  |  |
| M | 0007413 | axonal fasciculation | EN-124k-90-group138.Contig1 | | | | | | | | | |  |  |  |  |
| M | 0007413 | axonal fasciculation | EN-124k-90-group138.Contig62 | | | | | | | | | |  |  |  |  |
| M | 0007413 | axonal fasciculation | EN-124k-90-group229.Contig1 | | | | | | | | | |  |  |  |  |
| M | 0007413 | axonal fasciculation | EN-124k-90-group230.Contig1 | | | | | | | | | |  |  |  |  |
| M | 0007413 | axonal fasciculation | EN-124k-90-group232.Contig1 | | | | | | | | | |  |  |  |  |
| M | 0007413 | axonal fasciculation | EN-124k-90-group232.Contig2 | | | | | | | | | |  |  |  |  |
| M | 0007413 | axonal fasciculation | EN-124k-90-group232.Contig3 | | | | | | | | | |  |  |  |  |
| M | 0007413 | axonal fasciculation | EN-124k-90-group232.Contig5 | | | | | | | | | |  |  |  |  |
| M | 0007413 | axonal fasciculation | EN-124k-90-group253.Contig1 | | | | | | | | | |  |  |  |  |
| M | 0007413 | axonal fasciculation | EN-124k-90-group270.Contig2 | | | | | | | | | |  |  |  |  |
| M | 0007413 | axonal fasciculation | EN-124k-90-group290.Contig5 | | | | | | | | | |  |  |  |  |
| M | 0007413 | axonal fasciculation | EN-124k-90-group290.Contig6 | | | | | | | | | |  |  |  |  |
| M | 0007413 | axonal fasciculation | EN-124k-90-group414.Contig1 | | | | | | | | | |  |  |  |  |
| M | 0007413 | axonal fasciculation | EN-124k-90-group414.Contig2 | | | | | | | | | |  |  |  |  |
| M | 0007413 | axonal fasciculation | EN-124k-90-group414.Contig3 | | | | | | | | | |  |  |  |  |
| M | 0007413 | axonal fasciculation | EN-124k-90-group430.Contig1 | | | | | | | | | |  |  |  |  |
| M | 0007413 | axonal fasciculation | EN-124k-90-group506.Contig1 | | | | | | | | | |  |  |  |  |
| M | 0007413 | axonal fasciculation | EN-124k-90-group506.Contig3 | | | | | | | | | |  |  |  |  |
| M | 0007413 | axonal fasciculation | EN-124k-90-group506.Contig4 | | | | | | | | | |  |  |  |  |
| M | 0007413 | axonal fasciculation | EN-124k-90-group518.Contig2 | | | | | | | | | |  |  |  |  |
| M | 0007413 | axonal fasciculation | EN-124k-90-group524.Contig3 | | | | | | | | | |  |  |  |  |
| M | 0007413 | axonal fasciculation | EN-124k-90-group529.Contig2 | | | | | | | | | |  |  |  |  |
| M | 0007413 | axonal fasciculation | EN-124k-90-group550.Contig2 | | | | | | | | | |  |  |  |  |
| M | 0007413 | axonal fasciculation | EN-124k-90-group564.Contig5 | | | | | | | | | |  |  |  |  |
| M | 0007413 | axonal fasciculation | EN-124k-90-group586.Contig2 | | | | | | | | | |  |  |  |  |
| M | 0007413 | axonal fasciculation | EN-124k-90-group630.Contig2 | | | | | | | | | |  |  |  |  |
| M | 0007413 | axonal fasciculation | EN-124k-90-group682.Contig1 | | | | | | | | | |  |  |  |  |
| M | 0007413 | axonal fasciculation | EN-124k-90-group689.Contig4 | | | | | | | | | |  |  |  |  |
| M | 0007413 | axonal fasciculation | EN-124k-90-group727.Contig5 | | | | | | | | | |  |  |  |  |
| M | 0007413 | axonal fasciculation | EN-124k-90-group735.Contig1 | | | | | | | | | |  |  |  |  |
| M | 0007413 | axonal fasciculation | EN-124k-90-group750.Contig1 | | | | | | | | | |  |  |  |  |
| M | 0007413 | axonal fasciculation | EN-124k-90-group750.Contig4 | | | | | | | | | |  |  |  |  |
| M | 0007413 | axonal fasciculation | EN-124k-90-group750.Contig6 | | | | | | | | | |  |  |  |  |
| M | 0007413 | axonal fasciculation | EN-124k-90-group750.Contig7 | | | | | | | | | |  |  |  |  |
| M | 0007413 | axonal fasciculation | EN-124k-90-group850.Contig1 | | | | | | | | | |  |  |  |  |
| M | 0007413 | axonal fasciculation | EN-124k-90-group881.Contig2 | | | | | | | | | |  |  |  |  |
| M | 0007413 | axonal fasciculation | EN-124k-90-group881.Contig3 | | | | | | | | | |  |  |  |  |
| M | 0007413 | axonal fasciculation | EN-124k-90-group882.Contig2 | | | | | | | | | |  |  |  |  |
| M | 0007413 | axonal fasciculation | EN-124k-90-group900.Contig1 | | | | | | | | | |  |  |  |  |
| M | 0007413 | axonal fasciculation | EN-124k-90-group908.Contig6 | | | | | | | | | |  |  |  |  |
| M | 0007413 | axonal fasciculation | EN-124k-90-group944.Contig1 | | | | | | | | | |  |  |  |  |
| M | 0007413 | axonal fasciculation | EN-124k-90-group944.Contig2 | | | | | | | | | |  |  |  |  |
| M | 0007413 | axonal fasciculation | EN-124k-90-group944.Contig4 | | | | | | | | | |  |  |  |  |
| M | 0007413 | axonal fasciculation | EN-124k-90-group944.Contig5 | | | | | | | | | |  |  |  |  |
| M | 0007413 | axonal fasciculation | EN-124k-90-group944.Contig6 | | | | | | | | | |  |  |  |  |
| M | 0007413 | axonal fasciculation | EN-124k-90-group944.Contig7 | | | | | | | | | |  |  |  |  |
| M | 0007413 | axonal fasciculation | EN-124k-90-group944.Contig8 | | | | | | | | | |  |  |  |  |
| M | 0007413 | axonal fasciculation | EN-124k-90-group944.Contig9 | | | | | | | | | |  |  |  |  |
| M | 0007413 | axonal fasciculation | EN-124k-90-group944.Contig11 | | | | | | | | | |  |  |  |  |
| M | 0007413 | axonal fasciculation | EN-124k-90-group944.Contig13 | | | | | | | | | |  |  |  |  |
| M | 0007413 | axonal fasciculation | EN-124k-90-group944.Contig14 | | | | | | | | | |  |  |  |  |
| M | 0007413 | axonal fasciculation | EN-124k-90-group944.Contig15 | | | | | | | | | |  |  |  |  |
| M | 0007413 | axonal fasciculation | EN-124k-90-group945.Contig1 | | | | | | | | | |  |  |  |  |
| M | 0007413 | axonal fasciculation | EN-124k-90-group994.Contig1 | | | | | | | | | |  |  |  |  |
| M | 0007413 | axonal fasciculation | EN-124k-90-group998.Contig1 | | | | | | | | | |  |  |  |  |
| M | 0007413 | axonal fasciculation | EN-124k-90-group1106.Contig1 | | | | | | | | | |  |  |  |  |
| M | 0007413 | axonal fasciculation | EN-124k-90-group1135.Contig1 | | | | | | | | | |  |  |  |  |
| M | 0007413 | axonal fasciculation | EN-124k-90-group1135.Contig4 | | | | | | | | | |  |  |  |  |
| M | 0007413 | axonal fasciculation | EN-124k-90-group1135.Contig5 | | | | | | | | | |  |  |  |  |
| M | 0007413 | axonal fasciculation | EN-124k-90-group1135.Contig7 | | | | | | | | | |  |  |  |  |
| M | 0007413 | axonal fasciculation | EN-124k-90-group1161.Contig1 | | | | | | | | | |  |  |  |  |
| M | 0007413 | axonal fasciculation | EN-124k-90-group1161.Contig2 | | | | | | | | | |  |  |  |  |
| M | 0007413 | axonal fasciculation | EN-124k-90-group1272.Contig1 | | | | | | | | | |  |  |  |  |
| M | 0007413 | axonal fasciculation | EN-124k-90-group1294.Contig1 | | | | | | | | | |  |  |  |  |
| M | 0007413 | axonal fasciculation | EN-124k-90-group1302.Contig5 | | | | | | | | | |  |  |  |  |
| M | 0007413 | axonal fasciculation | EN-124k-90-group1304.Contig2 | | | | | | | | | |  |  |  |  |
| M | 0007413 | axonal fasciculation | EN-124k-90-group1304.Contig4 | | | | | | | | | |  |  |  |  |
| M | 0007413 | axonal fasciculation | EN-124k-90-group1304.Contig8 | | | | | | | | | |  |  |  |  |
| M | 0007413 | axonal fasciculation | EN-124k-90-group1351.Contig1 | | | | | | | | | |  |  |  |  |
| M | 0007413 | axonal fasciculation | EN-124k-90-group1423.Contig14 | | | | | | | | | |  |  |  |  |
| M | 0007413 | axonal fasciculation | EN-124k-90-group1423.Contig22 | | | | | | | | | |  |  |  |  |
| M | 0007413 | axonal fasciculation | EN-124k-90-group1456.Contig7 | | | | | | | | | |  |  |  |  |
| M | 0007413 | axonal fasciculation | EN-124k-90-group1462.Contig1 | | | | | | | | | |  |  |  |  |
| A | 0007413 | axonal fasciculation | EN-124k-90-group1482.Contig1 | | | | | | | | | |  |  |  |  |
| A | 0007413 | axonal fasciculation | EN-124k-90-group1521.Contig1 | | | | | | | | | |  |  |  |  |
| A | 0007413 | axonal fasciculation | EN-124k-90-group1521.Contig3 | | | | | | | | | |  |  |  |  |
| M | 0007413 | axonal fasciculation | EN-124k-90-group1567.Contig1 | | | | | | | | | |  |  |  |  |
| M | 0007413 | axonal fasciculation | EN-124k-90-group1578.Contig1 | | | | | | | | | |  |  |  |  |
| M | 0007413 | axonal fasciculation | EN-124k-90-group1578.Contig2 | | | | | | | | | |  |  |  |  |
| M | 0007413 | axonal fasciculation | EN-124k-90-group1578.Contig3 | | | | | | | | | |  |  |  |  |
| M | 0007413 | axonal fasciculation | EN-124k-90-group1626.Contig1 | | | | | | | | | |  |  |  |  |
| M | 0007413 | axonal fasciculation | EN-124k-90-group1654.Contig1 | | | | | | | | | |  |  |  |  |
| M | 0007413 | axonal fasciculation | EN-124k-90-group1654.Contig2 | | | | | | | | | |  |  |  |  |
| M | 0007413 | axonal fasciculation | EN-124k-90-group1657.Contig1 | | | | | | | | | |  |  |  |  |
| M | 0007413 | axonal fasciculation | EN-124k-90-group1735.Contig6 | | | | | | | | | |  |  |  |  |
| M | 0007413 | axonal fasciculation | EN-124k-90-group1762.Contig1 | | | | | | | | | |  |  |  |  |
| M | 0007413 | axonal fasciculation | EN-124k-90-group1770.Contig2 | | | | | | | | | |  |  |  |  |
| M | 0007413 | axonal fasciculation | EN-124k-90-group1782.Contig1 | | | | | | | | | |  |  |  |  |
| M | 0007413 | axonal fasciculation | EN-124k-90-group1798.Contig2 | | | | | | | | | |  |  |  |  |
| M | 0007413 | axonal fasciculation | EN-124k-90-group1798.Contig3 | | | | | | | | | |  |  |  |  |
| M | 0007413 | axonal fasciculation | EN-124k-90-group1799.Contig1 | | | | | | | | | |  |  |  |  |
| M | 0007413 | axonal fasciculation | EN-124k-90-group1799.Contig2 | | | | | | | | | |  |  |  |  |
| M | 0007413 | axonal fasciculation | EN-124k-90-group1799.Contig3 | | | | | | | | | |  |  |  |  |
| M | 0007413 | axonal fasciculation | EN-124k-90-group1799.Contig5 | | | | | | | | | |  |  |  |  |
| M | 0007413 | axonal fasciculation | EN-124k-90-group1799.Contig7 | | | | | | | | | |  |  |  |  |
| M | 0007413 | axonal fasciculation | EN-124k-90-group1799.Contig8 | | | | | | | | | |  |  |  |  |
| M | 0007413 | axonal fasciculation | EN-124k-90-group1799.Contig9 | | | | | | | | | |  |  |  |  |
| M | 0007413 | axonal fasciculation | EN-124k-90-group1819.Contig3 | | | | | | | | | |  |  |  |  |
| M | 0007413 | axonal fasciculation | EN-124k-90-group1834.Contig1 | | | | | | | | | |  |  |  |  |
| M | 0007413 | axonal fasciculation | EN-124k-90-group1861.Contig1 | | | | | | | | | |  |  |  |  |
| M | 0007413 | axonal fasciculation | EN-124k-90-group1861.Contig2 | | | | | | | | | |  |  |  |  |
| M | 0007413 | axonal fasciculation | EN-124k-90-group1867.Contig1 | | | | | | | | | |  |  |  |  |
| M | 0007413 | axonal fasciculation | EN-124k-90-group1907.Contig1 | | | | | | | | | |  |  |  |  |
| M | 0007413 | axonal fasciculation | EN-124k-90-group1909.Contig3 | | | | | | | | | |  |  |  |  |
| M | 0007413 | axonal fasciculation | EN-124k-90-group1915.Contig1 | | | | | | | | | |  |  |  |  |
| M | 0007413 | axonal fasciculation | EN-124k-90-group1958.Contig1 | | | | | | | | | |  |  |  |  |
| M | 0007413 | axonal fasciculation | EN-124k-90-group1960.Contig1 | | | | | | | | | |  |  |  |  |
| M | 0007413 | axonal fasciculation | EN-124k-90-group2021.Contig2 | | | | | | | | | |  |  |  |  |
| E | 0007413 | axonal fasciculation | EN-124k-90-group2041.Contig1 | | | | | | | | | |  |  |  |  |
| M | 0007413 | axonal fasciculation | EN-124k-90-group2076.Contig1 | | | | | | | | | |  |  |  |  |
| M | 0007413 | axonal fasciculation | EN-124k-90-group2081.Contig1 | | | | | | | | | |  |  |  |  |
| M | 0007413 | axonal fasciculation | EN-124k-90-group2081.Contig3 | | | | | | | | | |  |  |  |  |
| M | 0007413 | axonal fasciculation | EN-124k-90-group2097.Contig1 | | | | | | | | | |  |  |  |  |
| M | 0007413 | axonal fasciculation | EN-124k-90-group2200.Contig1 | | | | | | | | | |  |  |  |  |
| M | 0007413 | axonal fasciculation | EN-124k-90-group2428.Contig1 | | | | | | | | | |  |  |  |  |
| M | 0007413 | axonal fasciculation | EN-124k-90-group2449.Contig1 | | | | | | | | | |  |  |  |  |
| M | 0007413 | axonal fasciculation | EN-124k-90-group2449.Contig2 | | | | | | | | | |  |  |  |  |
| M | 0007413 | axonal fasciculation | EN-124k-90-group2451.Contig1 | | | | | | | | | |  |  |  |  |
| M | 0007413 | axonal fasciculation | EN-124k-90-group2455.Contig1 | | | | | | | | | |  |  |  |  |
| M | 0007413 | axonal fasciculation | EN-124k-90-group2472.Contig2 | | | | | | | | | |  |  |  |  |
| M | 0007413 | axonal fasciculation | EN-124k-90-group2472.Contig3 | | | | | | | | | |  |  |  |  |
| M | 0007413 | axonal fasciculation | EN-124k-90-group2495.Contig1 | | | | | | | | | |  |  |  |  |
| M | 0007413 | axonal fasciculation | EN-124k-90-group2495.Contig2 | | | | | | | | | |  |  |  |  |
| M | 0007413 | axonal fasciculation | EN-124k-90-group2498.Contig5 | | | | | | | | | |  |  |  |  |
| M | 0007413 | axonal fasciculation | EN-124k-90-group2537.Contig1 | | | | | | | | | |  |  |  |  |
| M | 0007413 | axonal fasciculation | EN-124k-90-group2540.Contig1 | | | | | | | | | |  |  |  |  |
| M | 0007413 | axonal fasciculation | EN-124k-90-group2540.Contig2 | | | | | | | | | |  |  |  |  |
| M | 0007413 | axonal fasciculation | EN-124k-90-group2582.Contig1 | | | | | | | | | |  |  |  |  |
| M | 0007413 | axonal fasciculation | EN-124k-90-group2584.Contig2 | | | | | | | | | |  |  |  |  |
| A | 0007413 | axonal fasciculation | EN-124k-90-group2585.Contig1 | | | | | | | | | |  |  |  |  |
| M | 0007413 | axonal fasciculation | EN-124k-90-group2606.Contig1 | | | | | | | | | |  |  |  |  |
| M | 0007413 | axonal fasciculation | EN-124k-90-group2681.Contig1 | | | | | | | | | |  |  |  |  |
| M | 0007413 | axonal fasciculation | EN-124k-90-group2686.Contig1 | | | | | | | | | |  |  |  |  |
| M | 0007413 | axonal fasciculation | EN-124k-90-group2834.Contig2 | | | | | | | | | |  |  |  |  |
| M | 0007413 | axonal fasciculation | EN-124k-90-group2835.Contig2 | | | | | | | | | |  |  |  |  |
| M | 0007413 | axonal fasciculation | EN-124k-90-group2852.Contig1 | | | | | | | | | |  |  |  |  |
| M | 0007413 | axonal fasciculation | EN-124k-90-group2903.Contig1 | | | | | | | | | |  |  |  |  |
| M | 0007413 | axonal fasciculation | EN-124k-90-group2917.Contig1 | | | | | | | | | |  |  |  |  |
| M | 0007413 | axonal fasciculation | EN-124k-90-group2917.Contig2 | | | | | | | | | |  |  |  |  |
| M | 0007413 | axonal fasciculation | EN-124k-90-group2917.Contig3 | | | | | | | | | |  |  |  |  |
| M | 0007413 | axonal fasciculation | EN-124k-90-group2917.Contig4 | | | | | | | | | |  |  |  |  |
| M | 0007413 | axonal fasciculation | EN-124k-90-group2940.Contig2 | | | | | | | | | |  |  |  |  |
| M | 0007413 | axonal fasciculation | EN-124k-90-group2959.Contig1 | | | | | | | | | |  |  |  |  |
| M | 0007413 | axonal fasciculation | EN-124k-90-group2959.Contig3 | | | | | | | | | |  |  |  |  |
| M | 0007413 | axonal fasciculation | EN-124k-90-group2975.Contig1 | | | | | | | | | |  |  |  |  |
| M | 0007413 | axonal fasciculation | EN-124k-90-group3082.Contig1 | | | | | | | | | |  |  |  |  |
| M | 0007413 | axonal fasciculation | EN-124k-90-group3082.Contig2 | | | | | | | | | |  |  |  |  |
| M | 0007413 | axonal fasciculation | EN-124k-90-group3135.Contig1 | | | | | | | | | |  |  |  |  |
| M | 0007413 | axonal fasciculation | EN-124k-90-group3135.Contig2 | | | | | | | | | |  |  |  |  |
| M | 0007413 | axonal fasciculation | EN-124k-90-group3223.Contig2 | | | | | | | | | |  |  |  |  |
| M | 0007413 | axonal fasciculation | EN-124k-90-group3224.Contig4 | | | | | | | | | |  |  |  |  |
| M | 0007413 | axonal fasciculation | EN-124k-90-group3224.Contig5 | | | | | | | | | |  |  |  |  |
| M | 0007413 | axonal fasciculation | EN-124k-90-group3292.Contig1 | | | | | | | | | |  |  |  |  |
| M | 0007413 | axonal fasciculation | EN-124k-90-group3299.Contig1 | | | | | | | | | |  |  |  |  |
| M | 0007413 | axonal fasciculation | EN-124k-90-group3313.Contig1 | | | | | | | | | |  |  |  |  |
| M | 0007413 | axonal fasciculation | EN-124k-90-group3313.Contig3 | | | | | | | | | |  |  |  |  |
| M | 0007413 | axonal fasciculation | EN-124k-90-group3315.Contig6 | | | | | | | | | |  |  |  |  |
| M | 0007413 | axonal fasciculation | EN-124k-90-group3343.Contig1 | | | | | | | | | |  |  |  |  |
| M | 0007413 | axonal fasciculation | EN-124k-90-group3394.Contig1 | | | | | | | | | |  |  |  |  |
| M | 0007413 | axonal fasciculation | EN-124k-90-group3394.Contig2 | | | | | | | | | |  |  |  |  |
| M | 0007413 | axonal fasciculation | EN-124k-90-group3394.Contig3 | | | | | | | | | |  |  |  |  |
| M | 0007413 | axonal fasciculation | EN-124k-90-group3394.Contig4 | | | | | | | | | |  |  |  |  |
| M | 0007413 | axonal fasciculation | EN-124k-90-group3401.Contig2 | | | | | | | | | |  |  |  |  |
| M | 0007413 | axonal fasciculation | EN-124k-90-group3477.Contig1 | | | | | | | | | |  |  |  |  |
| M | 0007413 | axonal fasciculation | EN-124k-90-group3479.Contig1 | | | | | | | | | |  |  |  |  |
| M | 0007413 | axonal fasciculation | EN-124k-90-group3511.Contig1 | | | | | | | | | |  |  |  |  |
| M | 0007413 | axonal fasciculation | EN-124k-90-group3511.Contig2 | | | | | | | | | |  |  |  |  |
| M | 0007413 | axonal fasciculation | EN-124k-90-group3522.Contig1 | | | | | | | | | |  |  |  |  |
| M | 0007413 | axonal fasciculation | EN-124k-90-group3567.Contig1 | | | | | | | | | |  |  |  |  |
| M | 0007413 | axonal fasciculation | EN-124k-90-group3567.Contig2 | | | | | | | | | |  |  |  |  |
| M | 0007413 | axonal fasciculation | EN-124k-90-group3567.Contig3 | | | | | | | | | |  |  |  |  |
| M | 0007413 | axonal fasciculation | EN-124k-90-group3582.Contig2 | | | | | | | | | |  |  |  |  |
| M | 0007413 | axonal fasciculation | EN-124k-90-group3586.Contig1 | | | | | | | | | |  |  |  |  |
| M | 0007413 | axonal fasciculation | EN-124k-90-group3594.Contig4 | | | | | | | | | |  |  |  |  |
| E | 0007413 | axonal fasciculation | EN-124k-90-group3611.Contig1 | | | | | | | | | |  |  |  |  |
| M | 0007413 | axonal fasciculation | EN-124k-90-group3630.Contig2 | | | | | | | | | |  |  |  |  |
| M | 0007413 | axonal fasciculation | EN-124k-90-group3700.Contig1 | | | | | | | | | |  |  |  |  |
| M | 0007413 | axonal fasciculation | EN-124k-90-group3713.Contig1 | | | | | | | | | |  |  |  |  |
| A | 0007413 | axonal fasciculation | EN-124k-90-group3737.Contig1 | | | | | | | | | |  |  |  |  |
| M | 0007413 | axonal fasciculation | EN-124k-90-group3816.Contig1 | | | | | | | | | |  |  |  |  |
| M | 0007413 | axonal fasciculation | EN-124k-90-group3882.Contig1 | | | | | | | | | |  |  |  |  |
| M | 0007413 | axonal fasciculation | EN-124k-90-group3897.Contig1 | | | | | | | | | |  |  |  |  |
| M | 0007413 | axonal fasciculation | EN-124k-90-group3916.Contig1 | | | | | | | | | |  |  |  |  |
| M | 0007413 | axonal fasciculation | EN-124k-90-group3933.Contig1 | | | | | | | | | |  |  |  |  |
| M | 0007413 | axonal fasciculation | EN-124k-90-group4009.Contig1 | | | | | | | | | |  |  |  |  |
| M | 0007413 | axonal fasciculation | EN-124k-90-group4045.Contig1 | | | | | | | | | |  |  |  |  |
| M | 0007413 | axonal fasciculation | EN-124k-90-group4046.Contig1 | | | | | | | | | |  |  |  |  |
| M | 0007413 | axonal fasciculation | EN-124k-90-group4049.Contig1 | | | | | | | | | |  |  |  |  |
| M | 0007413 | axonal fasciculation | EN-124k-90-group4168.Contig1 | | | | | | | | | |  |  |  |  |
| M | 0007413 | axonal fasciculation | EN-124k-90-group4168.Contig3 | | | | | | | | | |  |  |  |  |
| M | 0007413 | axonal fasciculation | EN-124k-90-group4183.Contig1 | | | | | | | | | |  |  |  |  |
| M | 0007413 | axonal fasciculation | EN-124k-90-group4194.Contig1 | | | | | | | | | |  |  |  |  |
| M | 0007413 | axonal fasciculation | EN-124k-90-group4216.Contig1 | | | | | | | | | |  |  |  |  |
| M | 0007413 | axonal fasciculation | EN-124k-90-group4349.Contig4 | | | | | | | | | |  |  |  |  |
| M | 0007413 | axonal fasciculation | EN-124k-90-group4351.Contig1 | | | | | | | | | |  |  |  |  |
| M | 0007413 | axonal fasciculation | EN-124k-90-group4372.Contig1 | | | | | | | | | |  |  |  |  |
| M | 0007413 | axonal fasciculation | EN-124k-90-group4410.Contig1 | | | | | | | | | |  |  |  |  |
| M | 0007413 | axonal fasciculation | EN-124k-90-group4432.Contig4 | | | | | | | | | |  |  |  |  |
| M | 0007413 | axonal fasciculation | EN-124k-90-group4477.Contig4 | | | | | | | | | |  |  |  |  |
| M | 0007413 | axonal fasciculation | EN-124k-90-group4562.Contig1 | | | | | | | | | |  |  |  |  |
| M | 0007413 | axonal fasciculation | EN-124k-90-group4600.Contig1 | | | | | | | | | |  |  |  |  |
| M | 0007413 | axonal fasciculation | EN-124k-90-group4616.Contig1 | | | | | | | | | |  |  |  |  |
| M | 0007413 | axonal fasciculation | EN-124k-90-group4753.Contig1 | | | | | | | | | |  |  |  |  |
| M | 0007413 | axonal fasciculation | EN-124k-90-group4759.Contig3 | | | | | | | | | |  |  |  |  |
| M | 0007413 | axonal fasciculation | EN-124k-90-group4763.Contig1 | | | | | | | | | |  |  |  |  |
| M | 0007413 | axonal fasciculation | EN-124k-90-group4827.Contig2 | | | | | | | | | |  |  |  |  |
| M | 0007413 | axonal fasciculation | EN-124k-90-group4929.Contig2 | | | | | | | | | |  |  |  |  |
| M | 0007413 | axonal fasciculation | EN-124k-90-group4944.Contig2 | | | | | | | | | |  |  |  |  |
| M | 0007413 | axonal fasciculation | EN-124k-90-group5036.Contig2 | | | | | | | | | |  |  |  |  |
| M | 0007413 | axonal fasciculation | EN-124k-90-group5040.Contig1 | | | | | | | | | |  |  |  |  |
| M | 0007413 | axonal fasciculation | EN-124k-90-group5040.Contig2 | | | | | | | | | |  |  |  |  |
| M | 0007413 | axonal fasciculation | EN-124k-90-group5055.Contig1 | | | | | | | | | |  |  |  |  |
| M | 0007413 | axonal fasciculation | EN-124k-90-group5055.Contig3 | | | | | | | | | |  |  |  |  |
| M | 0007413 | axonal fasciculation | EN-124k-90-group5063.Contig1 | | | | | | | | | |  |  |  |  |
| A | 0007413 | axonal fasciculation | EN-124k-90-group5155.Contig1 | | | | | | | | | |  |  |  |  |
| M | 0007413 | axonal fasciculation | EN-124k-90-group5170.Contig1 | | | | | | | | | |  |  |  |  |
| M | 0007413 | axonal fasciculation | EN-124k-90-group5170.Contig2 | | | | | | | | | |  |  |  |  |
| M | 0007413 | axonal fasciculation | EN-124k-90-group5170.Contig3 | | | | | | | | | |  |  |  |  |
| M | 0007413 | axonal fasciculation | EN-124k-90-group5172.Contig1 | | | | | | | | | |  |  |  |  |
| M | 0007413 | axonal fasciculation | EN-124k-90-group5173.Contig1 | | | | | | | | | |  |  |  |  |
| M | 0007413 | axonal fasciculation | EN-124k-90-group5203.Contig2 | | | | | | | | | |  |  |  |  |
| M | 0007413 | axonal fasciculation | EN-124k-90-group5215.Contig3 | | | | | | | | | |  |  |  |  |
| M | 0007413 | axonal fasciculation | EN-124k-90-group5252.Contig1 | | | | | | | | | |  |  |  |  |
| M | 0007413 | axonal fasciculation | EN-124k-90-group5273.Contig1 | | | | | | | | | |  |  |  |  |
| M | 0007413 | axonal fasciculation | EN-124k-90-group5273.Contig3 | | | | | | | | | |  |  |  |  |
| M | 0007413 | axonal fasciculation | EN-124k-90-group5298.Contig1 | | | | | | | | | |  |  |  |  |
| M | 0007413 | axonal fasciculation | EN-124k-90-group5329.Contig1 | | | | | | | | | |  |  |  |  |
| M | 0007413 | axonal fasciculation | EN-124k-90-group5398.Contig1 | | | | | | | | | |  |  |  |  |
| M | 0007413 | axonal fasciculation | EN-124k-90-group5479.Contig1 | | | | | | | | | |  |  |  |  |
| M | 0007413 | axonal fasciculation | EN-124k-90-group5491.Contig1 | | | | | | | | | |  |  |  |  |
| M | 0007413 | axonal fasciculation | EN-124k-90-group5491.Contig2 | | | | | | | | | |  |  |  |  |
| M | 0007413 | axonal fasciculation | EN-124k-90-group5532.Contig1 | | | | | | | | | |  |  |  |  |
| M | 0007413 | axonal fasciculation | EN-124k-90-group5532.Contig2 | | | | | | | | | |  |  |  |  |
| M | 0007413 | axonal fasciculation | EN-124k-90-group5541.Contig1 | | | | | | | | | |  |  |  |  |
| M | 0007413 | axonal fasciculation | EN-124k-90-group5567.Contig2 | | | | | | | | | |  |  |  |  |
| M | 0007413 | axonal fasciculation | EN-124k-90-group5567.Contig3 | | | | | | | | | |  |  |  |  |
| M | 0007413 | axonal fasciculation | EN-124k-90-group5607.Contig1 | | | | | | | | | |  |  |  |  |
| M | 0007413 | axonal fasciculation | EN-124k-90-group5616.Contig2 | | | | | | | | | |  |  |  |  |
| M | 0007413 | axonal fasciculation | EN-124k-90-group5616.Contig3 | | | | | | | | | |  |  |  |  |
| M | 0007413 | axonal fasciculation | EN-124k-90-group5709.Contig1 | | | | | | | | | |  |  |  |  |
| M | 0007413 | axonal fasciculation | EN-124k-90-group5719.Contig1 | | | | | | | | | |  |  |  |  |
| M | 0007413 | axonal fasciculation | EN-124k-90-group5817.Contig1 | | | | | | | | | |  |  |  |  |
| M | 0007413 | axonal fasciculation | EN-124k-90-group5968.Contig1 | | | | | | | | | |  |  |  |  |
| M | 0007413 | axonal fasciculation | EN-124k-90-group6033.Contig1 | | | | | | | | | |  |  |  |  |
| M | 0007413 | axonal fasciculation | EN-124k-90-group6033.Contig3 | | | | | | | | | |  |  |  |  |
| M | 0007413 | axonal fasciculation | EN-124k-90-group6038.Contig1 | | | | | | | | | |  |  |  |  |
| M | 0007413 | axonal fasciculation | EN-124k-90-group6104.Contig1 | | | | | | | | | |  |  |  |  |
| M | 0007413 | axonal fasciculation | EN-124k-90-group6104.Contig2 | | | | | | | | | |  |  |  |  |
| M | 0007413 | axonal fasciculation | EN-124k-90-group6147.Contig1 | | | | | | | | | |  |  |  |  |
| E | 0007413 | axonal fasciculation | EN-124k-90-group6238.Contig1 | | | | | | | | | |  |  |  |  |
| E | 0007413 | axonal fasciculation | EN-124k-90-group6326.Contig1 | | | | | | | | | |  |  |  |  |
| M | 0007413 | axonal fasciculation | EN-124k-90-group6358.Contig1 | | | | | | | | | |  |  |  |  |
| M | 0007413 | axonal fasciculation | EN-124k-90-group6358.Contig2 | | | | | | | | | |  |  |  |  |
| M | 0007413 | axonal fasciculation | EN-124k-90-group6400.Contig1 | | | | | | | | | |  |  |  |  |
| M | 0007413 | axonal fasciculation | EN-124k-90-group6472.Contig1 | | | | | | | | | |  |  |  |  |
| M | 0007413 | axonal fasciculation | EN-124k-90-group6472.Contig2 | | | | | | | | | |  |  |  |  |
| M | 0007413 | axonal fasciculation | EN-124k-90-group6591.Contig1 | | | | | | | | | |  |  |  |  |
| M | 0007413 | axonal fasciculation | EN-124k-90-group6647.Contig1 | | | | | | | | | |  |  |  |  |
| M | 0007413 | axonal fasciculation | EN-124k-90-group6809.Contig1 | | | | | | | | | |  |  |  |  |
| E | 0007413 | axonal fasciculation | EN-124k-90-group6858.Contig1 | | | | | | | | | |  |  |  |  |
| M | 0007413 | axonal fasciculation | EN-124k-90-group6923.Contig1 | | | | | | | | | |  |  |  |  |
| M | 0007413 | axonal fasciculation | EN-124k-90-group6923.Contig2 | | | | | | | | | |  |  |  |  |
| M | 0007413 | axonal fasciculation | EN-124k-90-group7010.Contig1 | | | | | | | | | |  |  |  |  |
| M | 0007413 | axonal fasciculation | EN-124k-90-group7013.Contig1 | | | | | | | | | |  |  |  |  |
| M | 0007413 | axonal fasciculation | EN-124k-90-group7017.Contig1 | | | | | | | | | |  |  |  |  |
| M | 0007413 | axonal fasciculation | EN-124k-90-group7107.Contig2 | | | | | | | | | |  |  |  |  |
| M | 0007413 | axonal fasciculation | EN-124k-90-group7149.Contig2 | | | | | | | | | |  |  |  |  |
| M | 0007413 | axonal fasciculation | EN-124k-90-group7157.Contig2 | | | | | | | | | |  |  |  |  |
| M | 0007413 | axonal fasciculation | EN-124k-90-group7165.Contig1 | | | | | | | | | |  |  |  |  |
| M | 0007413 | axonal fasciculation | EN-124k-90-group7231.Contig1 | | | | | | | | | |  |  |  |  |
| M | 0007413 | axonal fasciculation | EN-124k-90-group7234.Contig1 | | | | | | | | | |  |  |  |  |
| M | 0007413 | axonal fasciculation | EN-124k-90-group7343.Contig1 | | | | | | | | | |  |  |  |  |
| M | 0007413 | axonal fasciculation | EN-124k-90-group7440.Contig1 | | | | | | | | | |  |  |  |  |
| M | 0007413 | axonal fasciculation | EN-124k-90-group7457.Contig1 | | | | | | | | | |  |  |  |  |
| M | 0007413 | axonal fasciculation | EN-124k-90-group7460.Contig2 | | | | | | | | | |  |  |  |  |
| M | 0007413 | axonal fasciculation | EN-124k-90-group7463.Contig2 | | | | | | | | | |  |  |  |  |
| A | 0007413 | axonal fasciculation | EN-124k-90-group7650.Contig1 | | | | | | | | | |  |  |  |  |
| M | 0007413 | axonal fasciculation | EN-124k-90-group7740.Contig1 | | | | | | | | | |  |  |  |  |
| M | 0007413 | axonal fasciculation | EN-124k-90-group7740.Contig3 | | | | | | | | | |  |  |  |  |
| M | 0007413 | axonal fasciculation | EN-124k-90-group7871.Contig1 | | | | | | | | | |  |  |  |  |
| M | 0007413 | axonal fasciculation | EN-124k-90-group7964.Contig1 | | | | | | | | | |  |  |  |  |
| M | 0007413 | axonal fasciculation | EN-124k-90-group8079.Contig1 | | | | | | | | | |  |  |  |  |
| M | 0007413 | axonal fasciculation | EN-124k-90-group8079.Contig2 | | | | | | | | | |  |  |  |  |
| E | 0007413 | axonal fasciculation | EN-124k-90-group8101.Contig1 | | | | | | | | | |  |  |  |  |
| M | 0007413 | axonal fasciculation | EN-124k-90-group8131.Contig1 | | | | | | | | | |  |  |  |  |
| M | 0007413 | axonal fasciculation | EN-124k-90-group8174.Contig1 | | | | | | | | | |  |  |  |  |
| M | 0007413 | axonal fasciculation | EN-124k-90-group8174.Contig2 | | | | | | | | | |  |  |  |  |
| M | 0007413 | axonal fasciculation | EN-124k-90-group8211.Contig1 | | | | | | | | | |  |  |  |  |
| M | 0007413 | axonal fasciculation | EN-124k-90-group8253.Contig1 | | | | | | | | | |  |  |  |  |
| A | 0007413 | axonal fasciculation | EN-124k-90-group8519.Contig1 | | | | | | | | | |  |  |  |  |
| M | 0007413 | axonal fasciculation | EN-124k-90-group8524.Contig1 | | | | | | | | | |  |  |  |  |
| M | 0007413 | axonal fasciculation | EN-124k-90-group8568.Contig1 | | | | | | | | | |  |  |  |  |
| M | 0007413 | axonal fasciculation | EN-124k-90-group8645.Contig1 | | | | | | | | | |  |  |  |  |
| M | 0007413 | axonal fasciculation | EN-124k-90-group8696.Contig1 | | | | | | | | | |  |  |  |  |
| M | 0007413 | axonal fasciculation | EN-124k-90-group8799.Contig2 | | | | | | | | | |  |  |  |  |
| M | 0007413 | axonal fasciculation | EN-124k-90-group8850.Contig1 | | | | | | | | | |  |  |  |  |
| M | 0007413 | axonal fasciculation | EN-124k-90-group8897.Contig1 | | | | | | | | | |  |  |  |  |
| M | 0007413 | axonal fasciculation | EN-124k-90-group8937.Contig1 | | | | | | | | | |  |  |  |  |
| M | 0007413 | axonal fasciculation | EN-124k-90-group8937.Contig2 | | | | | | | | | |  |  |  |  |
| A | 0007413 | axonal fasciculation | EN-124k-90-group8957.Contig1 | | | | | | | | | |  |  |  |  |
| M | 0007413 | axonal fasciculation | EN-124k-90-group9165.Contig1 | | | | | | | | | |  |  |  |  |
| M | 0007413 | axonal fasciculation | EN-124k-90-group9168.Contig1 | | | | | | | | | |  |  |  |  |
| A | 0007413 | axonal fasciculation | EN-124k-90-group9245.Contig1 | | | | | | | | | |  |  |  |  |
| M | 0007413 | axonal fasciculation | EN-124k-90-group9278.Contig2 | | | | | | | | | |  |  |  |  |
| M | 0007413 | axonal fasciculation | EN-124k-90-group9299.Contig3 | | | | | | | | | |  |  |  |  |
| M | 0007413 | axonal fasciculation | EN-124k-90-group9317.Contig1 | | | | | | | | | |  |  |  |  |
| M | 0007413 | axonal fasciculation | EN-124k-90-group9388.Contig2 | | | | | | | | | |  |  |  |  |
| M | 0007413 | axonal fasciculation | EN-124k-90-group9446.Contig1 | | | | | | | | | |  |  |  |  |
| M | 0007413 | axonal fasciculation | EN-124k-90-group9485.Contig4 | | | | | | | | | |  |  |  |  |
| M | 0007413 | axonal fasciculation | EN-124k-90-group9506.Contig1 | | | | | | | | | |  |  |  |  |
| M | 0007413 | axonal fasciculation | EN-124k-90-group9517.Contig2 | | | | | | | | | |  |  |  |  |
| M | 0007413 | axonal fasciculation | EN-124k-90-group9771.Contig1 | | | | | | | | | |  |  |  |  |
| M | 0007413 | axonal fasciculation | EN-124k-90-group9771.Contig2 | | | | | | | | | |  |  |  |  |
| M | 0007413 | axonal fasciculation | EN-124k-90-group9819.Contig1 | | | | | | | | | |  |  |  |  |
| M | 0007413 | axonal fasciculation | EN-124k-90-group9887.Contig1 | | | | | | | | | |  |  |  |  |
| M | 0007413 | axonal fasciculation | EN-124k-90-group10085.Contig1 | | | | | | | | | |  |  |  |  |
| M | 0007413 | axonal fasciculation | EN-124k-90-group10085.Contig2 | | | | | | | | | |  |  |  |  |
| A | 0007413 | axonal fasciculation | EN-124k-90-group10370.Contig1 | | | | | | | | | |  |  |  |  |
| M | 0007413 | axonal fasciculation | EN-124k-90-group10467.Contig1 | | | | | | | | | |  |  |  |  |
| M | 0007413 | axonal fasciculation | EN-124k-90-group10630.Contig1 | | | | | | | | | |  |  |  |  |
| M | 0007413 | axonal fasciculation | EN-124k-90-group10630.Contig2 | | | | | | | | | |  |  |  |  |
| M | 0007413 | axonal fasciculation | EN-124k-90-group10652.Contig1 | | | | | | | | | |  |  |  |  |
| E | 0007413 | axonal fasciculation | EN-124k-90-group10722.Contig1 | | | | | | | | | |  |  |  |  |
| M | 0007413 | axonal fasciculation | EN-124k-90-group10759.Contig1 | | | | | | | | | |  |  |  |  |
| M | 0007413 | axonal fasciculation | EN-124k-90-group10790.Contig1 | | | | | | | | | |  |  |  |  |
| M | 0007413 | axonal fasciculation | EN-124k-90-group10838.Contig2 | | | | | | | | | |  |  |  |  |
| M | 0007413 | axonal fasciculation | EN-124k-90-group10912.Contig1 | | | | | | | | | |  |  |  |  |
| M | 0007413 | axonal fasciculation | EN-124k-90-group10928.Contig1 | | | | | | | | | |  |  |  |  |
| M | 0007413 | axonal fasciculation | EN-124k-90-group10963.Contig1 | | | | | | | | | |  |  |  |  |
| M | 0007413 | axonal fasciculation | EN-124k-90-group10990.Contig1 | | | | | | | | | |  |  |  |  |
| E | 0007413 | axonal fasciculation | EN-124k-90-group11029.Contig1 | | | | | | | | | |  |  |  |  |
| M | 0007413 | axonal fasciculation | EN-124k-90-group11131.Contig1 | | | | | | | | | |  |  |  |  |
| M | 0007413 | axonal fasciculation | EN-124k-90-group11134.Contig1 | | | | | | | | | |  |  |  |  |
| M | 0007413 | axonal fasciculation | EN-124k-90-group11235.Contig1 | | | | | | | | | |  |  |  |  |
| M | 0007413 | axonal fasciculation | EN-124k-90-group11392.Contig1 | | | | | | | | | |  |  |  |  |
| M | 0007413 | axonal fasciculation | EN-124k-90-group11392.Contig2 | | | | | | | | | |  |  |  |  |
| M | 0007413 | axonal fasciculation | EN-124k-90-group11408.Contig1 | | | | | | | | | |  |  |  |  |
| M | 0007413 | axonal fasciculation | EN-124k-90-group11707.Contig1 | | | | | | | | | |  |  |  |  |
| M | 0007413 | axonal fasciculation | EN-124k-90-group11808.Contig1 | | | | | | | | | |  |  |  |  |
| M | 0007413 | axonal fasciculation | EN-124k-90-group11810.Contig1 | | | | | | | | | |  |  |  |  |
| M | 0007413 | axonal fasciculation | EN-124k-90-group12003.Contig1 | | | | | | | | | |  |  |  |  |
| M | 0007413 | axonal fasciculation | EN-124k-90-group12099.Contig1 | | | | | | | | | |  |  |  |  |
| M | 0007413 | axonal fasciculation | EN-124k-90-group12143.Contig1 | | | | | | | | | |  |  |  |  |
| M | 0007413 | axonal fasciculation | EN-124k-90-group12337.Contig2 | | | | | | | | | |  |  |  |  |
| M | 0007413 | axonal fasciculation | EN-124k-90-group12409.Contig1 | | | | | | | | | |  |  |  |  |
| M | 0007413 | axonal fasciculation | EN-124k-90-group12409.Contig2 | | | | | | | | | |  |  |  |  |
| A | 0007413 | axonal fasciculation | EN-124k-90-group12616.Contig1 | | | | | | | | | |  |  |  |  |
| E | 0007413 | axonal fasciculation | EN-124k-90-group12653.Contig1 | | | | | | | | | |  |  |  |  |
| M | 0007413 | axonal fasciculation | EN-124k-90-group13121.Contig1 | | | | | | | | | |  |  |  |  |
| M | 0007413 | axonal fasciculation | EN-124k-90-group13235.Contig1 | | | | | | | | | |  |  |  |  |
| M | 0007413 | axonal fasciculation | EN-124k-90-group13502.Contig2 | | | | | | | | | |  |  |  |  |
| M | 0007413 | axonal fasciculation | EN-124k-90-group13646.Contig1 | | | | | | | | | |  |  |  |  |
| M | 0007413 | axonal fasciculation | EN-124k-90-group13754.Contig1 | | | | | | | | | |  |  |  |  |
| E | 0007413 | axonal fasciculation | EN-124k-90-group14241.Contig2 | | | | | | | | | |  |  |  |  |
| M | 0007413 | axonal fasciculation | EN-124k-90-group14390.Contig2 | | | | | | | | | |  |  |  |  |
| M | 0007413 | axonal fasciculation | EN-124k-90-group14708.Contig1 | | | | | | | | | |  |  |  |  |
| E | 0007413 | axonal fasciculation | EN-124k-90-group15137.Contig1 | | | | | | | | | |  |  |  |  |
| M | 0007413 | axonal fasciculation | EN-124k-90-group15757.Contig1 | | | | | | | | | |  |  |  |  |
| E | 0007413 | axonal fasciculation | EN-124k-90-group15764.Contig1 | | | | | | | | | |  |  |  |  |
| E | 0007413 | axonal fasciculation | EN-124k-90-group115.EN\_iowa\_11188 | | | | | | | | | | |  |  |  |
| A | 0007413 | axonal fasciculation | EN-124k-90-group230.gs\_25885 | | | | | | | | | |  |  |  |  |
| E | 0007413 | axonal fasciculation | EN-124k-90-group232.jgi\_contig\_JGI\_CBBP16338\_fwd | | | | | | | | | | | | |  |
| E | 0007413 | axonal fasciculation | EN-124k-90-group232.jgi\_contig\_JGI\_CBBP9823\_fwd | | | | | | | | | | | | |  |
| E | 0007413 | axonal fasciculation | EN-124k-90-group232.jgi\_contig\_JGI\_CBBP10596\_fwd | | | | | | | | | | | | |  |
| E | 0007413 | axonal fasciculation | EN-124k-90-group290.EN\_iowa\_14805 | | | | | | | | | | |  |  |  |
| E | 0007413 | axonal fasciculation | EN-124k-90-group414.EN\_iowa\_11198 | | | | | | | | | | |  |  |  |
| A | 0007413 | axonal fasciculation | EN-124k-90-group735.gs\_7849 | | | | | | | | | |  |  |  |  |
| E | 0007413 | axonal fasciculation | EN-124k-90-group850.EN\_iowa\_1547 | | | | | | | | | | |  |  |  |
| E | 0007413 | axonal fasciculation | EN-124k-90-group900.EN\_iowa\_15436 | | | | | | | | | | |  |  |  |
| A | 0007413 | axonal fasciculation | EN-124k-90-group900.gs\_31783 | | | | | | | | | |  |  |  |  |
| A | 0007413 | axonal fasciculation | EN-124k-90-group900.gs\_71447 | | | | | | | | | |  |  |  |  |
| E | 0007413 | axonal fasciculation | EN-124k-90-group944.EN\_iowa\_1330 | | | | | | | | | | |  |  |  |
| E | 0007413 | axonal fasciculation | EN-124k-90-group1135.jgi\_unpaired\_JGI\_CBBP19047\_fwd | | | | | | | | | | | | | |
| E | 0007413 | axonal fasciculation | EN-124k-90-group1135.jgi\_contig\_JGI\_CBBP19919\_fwd | | | | | | | | | | | | |  |
| A | 0007413 | axonal fasciculation | EN-124k-90-group1135.gs\_2733 | | | | | | | | | |  |  |  |  |
| A | 0007413 | axonal fasciculation | EN-124k-90-group1302.gs\_68057 | | | | | | | | | |  |  |  |  |
| A | 0007413 | axonal fasciculation | EN-124k-90-group1351.gs\_80275 | | | | | | | | | |  |  |  |  |
| A | 0007413 | axonal fasciculation | EN-124k-90-group1423.gs\_16014 | | | | | | | | | |  |  |  |  |
| A | 0007413 | axonal fasciculation | EN-124k-90-group1423.gs\_11172 | | | | | | | | | |  |  |  |  |
| A | 0007413 | axonal fasciculation | EN-124k-90-group1423.gs\_52362 | | | | | | | | | |  |  |  |  |
| A | 0007413 | axonal fasciculation | EN-124k-90-group1423.gs\_75717 | | | | | | | | | |  |  |  |  |
| E | 0007413 | axonal fasciculation | EN-124k-90-group1456.jgi\_contig\_JGI\_CBBP4193\_fwd | | | | | | | | | | | | |  |
| A | 0007413 | axonal fasciculation | EN-124k-90-group1521.gs\_86350 | | | | | | | | | |  |  |  |  |
| E | 0007413 | axonal fasciculation | EN-124k-90-group1626.jgi\_contig\_JGI\_CBBP20097\_fwd | | | | | | | | | | | | |  |
| A | 0007413 | axonal fasciculation | EN-124k-90-group1626.gs\_84009 | | | | | | | | | |  |  |  |  |
| E | 0007413 | axonal fasciculation | EN-124k-90-group1626.jgi\_contig\_JGI\_CBBP16438\_fwd | | | | | | | | | | | | |  |
| A | 0007413 | axonal fasciculation | EN-124k-90-group1654.gs\_12044 | | | | | | | | | |  |  |  |  |
| A | 0007413 | axonal fasciculation | EN-124k-90-group1782.gs\_16663 | | | | | | | | | |  |  |  |  |
| A | 0007413 | axonal fasciculation | EN-124k-90-group1798.gs\_54312 | | | | | | | | | |  |  |  |  |
| A | 0007413 | axonal fasciculation | EN-124k-90-group1798.gs\_39367 | | | | | | | | | |  |  |  |  |
| E | 0007413 | axonal fasciculation | EN-124k-90-group1798.EN\_iowa\_16312 | | | | | | | | | | |  |  |  |
| A | 0007413 | axonal fasciculation | EN-124k-90-group1798.gs\_29292 | | | | | | | | | |  |  |  |  |
| A | 0007413 | axonal fasciculation | EN-124k-90-group1798.gs\_11661 | | | | | | | | | |  |  |  |  |
| A | 0007413 | axonal fasciculation | EN-124k-90-group1798.gs\_36996 | | | | | | | | | |  |  |  |  |
| A | 0007413 | axonal fasciculation | EN-124k-90-group1798.gs\_46615 | | | | | | | | | |  |  |  |  |
| E | 0007413 | axonal fasciculation | EN-124k-90-group1798.EN\_iowa\_6796 | | | | | | | | | | |  |  |  |
| A | 0007413 | axonal fasciculation | EN-124k-90-group1798.gs\_56703 | | | | | | | | | |  |  |  |  |
| A | 0007413 | axonal fasciculation | EN-124k-90-group1798.gs\_77511 | | | | | | | | | |  |  |  |  |
| A | 0007413 | axonal fasciculation | EN-124k-90-group1798.gs\_54963 | | | | | | | | | |  |  |  |  |
| A | 0007413 | axonal fasciculation | EN-124k-90-group1798.gs\_79284 | | | | | | | | | |  |  |  |  |
| A | 0007413 | axonal fasciculation | EN-124k-90-group1798.gs\_4498 | | | | | | | | | |  |  |  |  |
| A | 0007413 | axonal fasciculation | EN-124k-90-group1798.gs\_7178 | | | | | | | | | |  |  |  |  |
| A | 0007413 | axonal fasciculation | EN-124k-90-group1798.gs\_7576 | | | | | | | | | |  |  |  |  |
| A | 0007413 | axonal fasciculation | EN-124k-90-group1798.gs\_11790 | | | | | | | | | |  |  |  |  |
| A | 0007413 | axonal fasciculation | EN-124k-90-group1798.gs\_50785 | | | | | | | | | |  |  |  |  |
| A | 0007413 | axonal fasciculation | EN-124k-90-group1798.gs\_55765 | | | | | | | | | |  |  |  |  |
| E | 0007413 | axonal fasciculation | EN-124k-90-group1799.EN\_iowa\_15365 | | | | | | | | | | |  |  |  |
| A | 0007413 | axonal fasciculation | EN-124k-90-group1799.gs\_13071 | | | | | | | | | |  |  |  |  |
| A | 0007413 | axonal fasciculation | EN-124k-90-group1799.gs\_20064 | | | | | | | | | |  |  |  |  |
| A | 0007413 | axonal fasciculation | EN-124k-90-group1799.gs\_76593 | | | | | | | | | |  |  |  |  |
| A | 0007413 | axonal fasciculation | EN-124k-90-group1799.gs\_77643 | | | | | | | | | |  |  |  |  |
| A | 0007413 | axonal fasciculation | EN-124k-90-group1799.gs\_38929 | | | | | | | | | |  |  |  |  |
| E | 0007413 | axonal fasciculation | EN-124k-90-group1799.EN\_iowa\_9582 | | | | | | | | | | |  |  |  |
| A | 0007413 | axonal fasciculation | EN-124k-90-group1799.gs\_3745 | | | | | | | | | |  |  |  |  |
| E | 0007413 | axonal fasciculation | EN-124k-90-group1799.EN\_iowa\_16390 | | | | | | | | | | |  |  |  |
| E | 0007413 | axonal fasciculation | EN-124k-90-group1819.jgi\_contig\_JGI\_CBBP4948\_fwd | | | | | | | | | | | | |  |
| A | 0007413 | axonal fasciculation | EN-124k-90-group2021.gs\_23180 | | | | | | | | | |  |  |  |  |
| E | 0007413 | axonal fasciculation | EN-124k-90-group2076.jgi\_contig\_JGI\_CBBP929\_fwd | | | | | | | | | | | | |  |
| A | 0007413 | axonal fasciculation | EN-124k-90-group2076.gs\_5951 | | | | | | | | | |  |  |  |  |
| A | 0007413 | axonal fasciculation | EN-124k-90-group2076.gs\_13876 | | | | | | | | | |  |  |  |  |
| A | 0007413 | axonal fasciculation | EN-124k-90-group2076.gs\_6163 | | | | | | | | | |  |  |  |  |
| A | 0007413 | axonal fasciculation | EN-124k-90-group2449.gs\_35369 | | | | | | | | | |  |  |  |  |
| E | 0007413 | axonal fasciculation | EN-124k-90-group2451.jgi\_contig\_JGI\_CBBP13284\_fwd | | | | | | | | | | | | |  |
| A | 0007413 | axonal fasciculation | EN-124k-90-group2903.gs\_16952 | | | | | | | | | |  |  |  |  |
| E | 0007413 | axonal fasciculation | EN-124k-90-group2959.jgi\_contig\_JGI\_CBBP3203\_fwd | | | | | | | | | | | | |  |
| E | 0007413 | axonal fasciculation | EN-124k-90-group2959.jgi\_contig\_JGI\_CBBP16696\_fwd | | | | | | | | | | | | |  |
| E | 0007413 | axonal fasciculation | EN-124k-90-group2959.jgi\_contig\_JGI\_CBBP5581\_fwd | | | | | | | | | | | | |  |
| A | 0007413 | axonal fasciculation | EN-124k-90-group3394.gs\_42372 | | | | | | | | | |  |  |  |  |
| A | 0007413 | axonal fasciculation | EN-124k-90-group3522.gs\_9768 | | | | | | | | | |  |  |  |  |
| E | 0007413 | axonal fasciculation | EN-124k-90-group3582.jgi\_contig\_JGI\_CBBP13041\_fwd | | | | | | | | | | | | |  |
| A | 0007413 | axonal fasciculation | EN-124k-90-group3594.gs\_29852 | | | | | | | | | |  |  |  |  |
| E | 0007413 | axonal fasciculation | EN-124k-90-group3611.EN\_iowa\_4463 | | | | | | | | | | |  |  |  |
| A | 0007413 | axonal fasciculation | EN-124k-90-group3630.gs\_19342 | | | | | | | | | |  |  |  |  |
| A | 0007413 | axonal fasciculation | EN-124k-90-group3630.gs\_19317 | | | | | | | | | |  |  |  |  |
| A | 0007413 | axonal fasciculation | EN-124k-90-group3713.gs\_7861 | | | | | | | | | |  |  |  |  |
| E | 0007413 | axonal fasciculation | EN-124k-90-group4049.EN\_iowa\_15523 | | | | | | | | | | |  |  |  |
| A | 0007413 | axonal fasciculation | EN-124k-90-group4349.gs\_17809 | | | | | | | | | |  |  |  |  |
| E | 0007413 | axonal fasciculation | EN-124k-90-group4600.jgi\_contig\_JGI\_CBBP12800\_fwd | | | | | | | | | | | | |  |
| A | 0007413 | axonal fasciculation | EN-124k-90-group5203.gs\_53868 | | | | | | | | | |  |  |  |  |
| A | 0007413 | axonal fasciculation | EN-124k-90-group5203.gs\_41715 | | | | | | | | | |  |  |  |  |
| A | 0007413 | axonal fasciculation | EN-124k-90-group5203.gs\_19246 | | | | | | | | | |  |  |  |  |
| A | 0007413 | axonal fasciculation | EN-124k-90-group5215.gs\_74365 | | | | | | | | | |  |  |  |  |
| A | 0007413 | axonal fasciculation | EN-124k-90-group5491.gs\_7631 | | | | | | | | | |  |  |  |  |
| A | 0007413 | axonal fasciculation | EN-124k-90-group5491.gs\_75129 | | | | | | | | | |  |  |  |  |
| A | 0007413 | axonal fasciculation | EN-124k-90-group5532.gs\_21595 | | | | | | | | | |  |  |  |  |
| A | 0007413 | axonal fasciculation | EN-124k-90-group5532.gs\_71380 | | | | | | | | | |  |  |  |  |
| A | 0007413 | axonal fasciculation | EN-124k-90-group5532.gs\_38926 | | | | | | | | | |  |  |  |  |
| A | 0007413 | axonal fasciculation | EN-124k-90-group5532.gs\_31235 | | | | | | | | | |  |  |  |  |
| E | 0007413 | axonal fasciculation | EN-124k-90-group5532.EN\_iowa\_9665 | | | | | | | | | | |  |  |  |
| A | 0007413 | axonal fasciculation | EN-124k-90-group5532.gs\_59967 | | | | | | | | | |  |  |  |  |
| E | 0007413 | axonal fasciculation | EN-124k-90-group5532.EN\_iowa\_13749 | | | | | | | | | | |  |  |  |
| A | 0007413 | axonal fasciculation | EN-124k-90-group5532.gs\_23346 | | | | | | | | | |  |  |  |  |
| E | 0007413 | axonal fasciculation | EN-124k-90-group5532.EN\_iowa\_12774 | | | | | | | | | | |  |  |  |
| A | 0007413 | axonal fasciculation | EN-124k-90-group5532.gs\_2852 | | | | | | | | | |  |  |  |  |
| A | 0007413 | axonal fasciculation | EN-124k-90-group5532.gs\_25188 | | | | | | | | | |  |  |  |  |
| A | 0007413 | axonal fasciculation | EN-124k-90-group5532.gs\_35715 | | | | | | | | | |  |  |  |  |
| A | 0007413 | axonal fasciculation | EN-124k-90-group5532.gs\_7280 | | | | | | | | | |  |  |  |  |
| E | 0007413 | axonal fasciculation | EN-124k-90-group5607.jgi\_contig\_JGI\_CBBP445\_fwd | | | | | | | | | | | | |  |
| E | 0007413 | axonal fasciculation | EN-124k-90-group6472.jgi\_contig\_JGI\_CBBP2402\_fwd | | | | | | | | | | | | |  |
| E | 0007413 | axonal fasciculation | EN-124k-90-group6923.jgi\_contig\_JGI\_CBBP17789\_fwd | | | | | | | | | | | | |  |
| A | 0007413 | axonal fasciculation | EN-124k-90-group7440.gs\_75782 | | | | | | | | | |  |  |  |  |
| A | 0007413 | axonal fasciculation | EN-124k-90-group10963.gs\_69412 | | | | | | | | | |  |  |  |  |
| E | 0007413 | axonal fasciculation | EN-124k-90-group12653.EN\_iowa\_13251 | | | | | | | | | | |  |  |  |
| A | 0007409 | axonogenesis | EN-124k-90-group10674.gs\_45324 | | | | | | | | | |  |  |  |  |
| E | 0007409 | axonogenesis | EN-124k-90-group343.jgi\_contig\_JGI\_CBBP19299\_fwd | | | | | | | | | | | | |  |
| A | 0007409 | axonogenesis | EN-124k-90-group807.gs\_71281 | | | | | | | | | |  |  |  |  |
| E | 0007409 | axonogenesis | EN-124k-90-group1177.jgi\_unpaired\_JGI\_CBBP6661\_fwd | | | | | | | | | | | | |  |
| E | 0007409 | axonogenesis | EN-124k-90-group1306.EN\_iowa\_2354 | | | | | | | | | | |  |  |  |
| E | 0007409 | axonogenesis | EN-124k-90-group1680.jgi\_contig\_JGI\_CBBP17232\_fwd | | | | | | | | | | | | |  |
| E | 0007409 | axonogenesis | EN-124k-90-group1825.jgi\_contig\_JGI\_CBBP14395\_fwd | | | | | | | | | | | | |  |
| E | 0007409 | axonogenesis | EN-124k-90-group1852.jgi\_paired\_JGI\_CBBP10940\_fwd | | | | | | | | | | | | |  |
| A | 0007409 | axonogenesis | EN-124k-90-group1944.gs\_55531 | | | | | | | | | |  |  |  |  |
| E | 0007409 | axonogenesis | EN-124k-90-group2106.jgi\_paired\_JGI\_CBBP3443\_fwd | | | | | | | | | | | | |  |
| E | 0007409 | axonogenesis | EN-124k-90-group2530.jgi\_paired\_JGI\_CBBP5537\_fwd | | | | | | | | | | | | |  |
| E | 0007409 | axonogenesis | EN-124k-90-group2916.jgi\_paired\_JGI\_CBBP2065\_fwd | | | | | | | | | | | | |  |
| A | 0007409 | axonogenesis | EN-124k-90-group2932.gs\_32728 | | | | | | | | | |  |  |  |  |
| E | 0007409 | axonogenesis | EN-124k-90-group3265.jgi\_paired\_JGI\_CBBP17018\_rev | | | | | | | | | | | | |  |
| A | 0007409 | axonogenesis | EN-124k-90-group3420.gs\_43284 | | | | | | | | | |  |  |  |  |
| E | 0007409 | axonogenesis | EN-124k-90-group3448.jgi\_paired\_JGI\_CBBP19729\_fwd | | | | | | | | | | | | |  |
| A | 0007409 | axonogenesis | EN-124k-90-group3564.gs\_15152 | | | | | | | | | |  |  |  |  |
| E | 0007409 | axonogenesis | EN-124k-90-group3588.jgi\_paired\_JGI\_CBBP13551\_rev | | | | | | | | | | | | |  |
| A | 0007409 | axonogenesis | EN-124k-90-group3807.gs\_52991 | | | | | | | | | |  |  |  |  |
| E | 0007409 | axonogenesis | EN-124k-90-group4197.jgi\_paired\_JGI\_CBBP4981\_fwd | | | | | | | | | | | | |  |
| E | 0007409 | axonogenesis | EN-124k-90-group4248.jgi\_contig\_JGI\_CBBP19155\_fwd | | | | | | | | | | | | |  |
| E | 0007409 | axonogenesis | EN-124k-90-group5117.jgi\_paired\_JGI\_CBBP19153\_fwd | | | | | | | | | | | | |  |
| A | 0007409 | axonogenesis | EN-124k-90-group5330.gs\_86287 | | | | | | | | | |  |  |  |  |
| A | 0007409 | axonogenesis | EN-124k-90-group5346.gs\_72008 | | | | | | | | | |  |  |  |  |
| A | 0007409 | axonogenesis | EN-124k-90-group5384.gs\_17980 | | | | | | | | | |  |  |  |  |
| A | 0007409 | axonogenesis | EN-124k-90-group5423.gs\_20381 | | | | | | | | | |  |  |  |  |
| A | 0007409 | axonogenesis | EN-124k-90-group5604.gs\_26346 | | | | | | | | | |  |  |  |  |
| E | 0007409 | axonogenesis | EN-124k-90-group5741.jgi\_paired\_JGI\_CBBP19133\_fwd | | | | | | | | | | | | |  |
| E | 0007409 | axonogenesis | EN-124k-90-group5833.EN\_iowa\_5392 | | | | | | | | | | |  |  |  |
| E | 0007409 | axonogenesis | EN-124k-90-group5834.jgi\_contig\_JGI\_CBBP12701\_fwd | | | | | | | | | | | | |  |
| E | 0007409 | axonogenesis | EN-124k-90-group6144.EN\_iowa\_5856 | | | | | | | | | | |  |  |  |
| E | 0007409 | axonogenesis | EN-124k-90-group6152.jgi\_paired\_JGI\_CBBP3620\_fwd | | | | | | | | | | | | |  |
| E | 0007409 | axonogenesis | EN-124k-90-group6737.jgi\_contig\_JGI\_CBBP17820\_fwd | | | | | | | | | | | | |  |
| E | 0007409 | axonogenesis | EN-124k-90-group7523.jgi\_paired\_JGI\_CBBP3627\_rev | | | | | | | | | | | | |  |
| E | 0007409 | axonogenesis | EN-124k-90-group7594.jgi\_paired\_JGI\_CBBP17768\_fwd | | | | | | | | | | | | |  |
| A | 0007409 | axonogenesis | EN-124k-90-group7595.gs\_36261 | | | | | | | | | |  |  |  |  |
| E | 0007409 | axonogenesis | EN-124k-90-group7598.jgi\_unpaired\_JGI\_CBBP18343\_fwd | | | | | | | | | | | | | |
| E | 0007409 | axonogenesis | EN-124k-90-group7869.jgi\_paired\_JGI\_CBBP11345\_fwd | | | | | | | | | | | | |  |
| E | 0007409 | axonogenesis | EN-124k-90-group7940.jgi\_contig\_JGI\_CBBP6088\_fwd | | | | | | | | | | | | |  |
| A | 0007409 | axonogenesis | EN-124k-90-group7953.gs\_11284 | | | | | | | | | |  |  |  |  |
| E | 0007409 | axonogenesis | EN-124k-90-group8037.jgi\_paired\_JGI\_CBBP11201\_fwd | | | | | | | | | | | | |  |
| E | 0007409 | axonogenesis | EN-124k-90-group8104.jgi\_paired\_JGI\_CBBP9893\_fwd | | | | | | | | | | | | |  |
| E | 0007409 | axonogenesis | EN-124k-90-group8107.jgi\_paired\_JGI\_CBBP6197\_fwd | | | | | | | | | | | | |  |
| A | 0007409 | axonogenesis | EN-124k-90-group8144.gs\_73566 | | | | | | | | | |  |  |  |  |
| A | 0007409 | axonogenesis | EN-124k-90-group8310.gs\_24913 | | | | | | | | | |  |  |  |  |
| E | 0007409 | axonogenesis | EN-124k-90-group8370.jgi\_paired\_JGI\_CBBP17511\_fwd | | | | | | | | | | | | |  |
| E | 0007409 | axonogenesis | EN-124k-90-group8443.jgi\_paired\_JGI\_CBBP6445\_fwd | | | | | | | | | | | | |  |
| A | 0007409 | axonogenesis | EN-124k-90-group8567.gs\_80390 | | | | | | | | | |  |  |  |  |
| A | 0007409 | axonogenesis | EN-124k-90-group8658.gs\_22315 | | | | | | | | | |  |  |  |  |
| E | 0007409 | axonogenesis | EN-124k-90-group8721.jgi\_paired\_JGI\_CBBP11443\_rev | | | | | | | | | | | | |  |
| E | 0007409 | axonogenesis | EN-124k-90-group9037.jgi\_paired\_JGI\_CBBP9664\_rev | | | | | | | | | | | | |  |
| E | 0007409 | axonogenesis | EN-124k-90-group9064.EN\_iowa\_5147 | | | | | | | | | | |  |  |  |
| E | 0007409 | axonogenesis | EN-124k-90-group9263.jgi\_paired\_JGI\_CBBP3642\_fwd | | | | | | | | | | | | |  |
| E | 0007409 | axonogenesis | EN-124k-90-group9286.jgi\_paired\_JGI\_CBBP4063\_fwd | | | | | | | | | | | | |  |
| A | 0007409 | axonogenesis | EN-124k-90-group9467.gs\_16039 | | | | | | | | | |  |  |  |  |
| A | 0007409 | axonogenesis | EN-124k-90-group9534.gs\_76749 | | | | | | | | | |  |  |  |  |
| A | 0007409 | axonogenesis | EN-124k-90-group9629.gs\_48808 | | | | | | | | | |  |  |  |  |
| A | 0007409 | axonogenesis | EN-124k-90-group9714.gs\_64750 | | | | | | | | | |  |  |  |  |
| A | 0007409 | axonogenesis | EN-124k-90-group9739.gs\_17794 | | | | | | | | | |  |  |  |  |
| E | 0007409 | axonogenesis | EN-124k-90-group9773.jgi\_paired\_JGI\_CBBP6585\_rev | | | | | | | | | | | | |  |
| A | 0007409 | axonogenesis | EN-124k-90-group9881.gs\_79019 | | | | | | | | | |  |  |  |  |
| A | 0007409 | axonogenesis | EN-124k-90-group9910.gs\_20549 | | | | | | | | | |  |  |  |  |
| A | 0007409 | axonogenesis | EN-124k-90-group9923.gs\_69535 | | | | | | | | | |  |  |  |  |
| E | 0007409 | axonogenesis | EN-124k-90-group9989.EN\_iowa\_3399 | | | | | | | | | | |  |  |  |
| E | 0007409 | axonogenesis | EN-124k-90-group10110.jgi\_paired\_JGI\_CBBP10219\_fwd | | | | | | | | | | | | |  |
| E | 0007409 | axonogenesis | EN-124k-90-group10277.jgi\_paired\_JGI\_CBBP15154\_rev | | | | | | | | | | | | |  |
| E | 0007409 | axonogenesis | EN-124k-90-group10304.jgi\_paired\_JGI\_CBBP11045\_fwd | | | | | | | | | | | | |  |
| A | 0007409 | axonogenesis | EN-124k-90-group10312.gs\_22624 | | | | | | | | | |  |  |  |  |
| E | 0007409 | axonogenesis | EN-124k-90-group10416.jgi\_contig\_JGI\_CBBP3274\_fwd | | | | | | | | | | | | |  |
| E | 0007409 | axonogenesis | EN-124k-90-group10458.jgi\_contig\_JGI\_CBBP9840\_fwd | | | | | | | | | | | | |  |
| E | 0007409 | axonogenesis | EN-124k-90-group10538.jgi\_contig\_JGI\_CBBP12785\_fwd | | | | | | | | | | | | |  |
| A | 0007409 | axonogenesis | EN-124k-90-group10635.gs\_47150 | | | | | | | | | |  |  |  |  |
| A | 0007409 | axonogenesis | EN-124k-90-group10841.gs\_43135 | | | | | | | | | |  |  |  |  |
| E | 0007409 | axonogenesis | EN-124k-90-group10878.jgi\_paired\_JGI\_CBBP952\_rev | | | | | | | | | | | | |  |
| E | 0007409 | axonogenesis | EN-124k-90-group10888.jgi\_paired\_JGI\_CBBP10968\_rev | | | | | | | | | | | | |  |
| A | 0007409 | axonogenesis | EN-124k-90-group10935.gs\_64995 | | | | | | | | | |  |  |  |  |
| E | 0007409 | axonogenesis | EN-124k-90-group10968.EN\_iowa\_5471 | | | | | | | | | | |  |  |  |
| A | 0007409 | axonogenesis | EN-124k-90-group11067.gs\_59652 | | | | | | | | | |  |  |  |  |
| A | 0007409 | axonogenesis | EN-124k-90-group11147.gs\_84591 | | | | | | | | | |  |  |  |  |
| E | 0007409 | axonogenesis | EN-124k-90-group11156.EN\_iowa\_9243 | | | | | | | | | | |  |  |  |
| E | 0007409 | axonogenesis | EN-124k-90-group11184.EN\_iowa\_18337 | | | | | | | | | | |  |  |  |
| E | 0007409 | axonogenesis | EN-124k-90-group11237.jgi\_paired\_JGI\_CBBP10968\_fwd | | | | | | | | | | | | |  |
| A | 0007409 | axonogenesis | EN-124k-90-group11354.gs\_78099 | | | | | | | | | |  |  |  |  |
| A | 0007409 | axonogenesis | EN-124k-90-group11434.gs\_43503 | | | | | | | | | |  |  |  |  |
| E | 0007409 | axonogenesis | EN-124k-90-group11438.jgi\_paired\_JGI\_CBBP9709\_fwd | | | | | | | | | | | | |  |
| A | 0007409 | axonogenesis | EN-124k-90-group11441.gs\_30577 | | | | | | | | | |  |  |  |  |
| E | 0007409 | axonogenesis | EN-124k-90-group11503.jgi\_paired\_JGI\_CBBP14517\_fwd | | | | | | | | | | | | |  |
| E | 0007409 | axonogenesis | EN-124k-90-group11545.jgi\_paired\_JGI\_CBBP2808\_fwd | | | | | | | | | | | | |  |
| E | 0007409 | axonogenesis | EN-124k-90-group11559.jgi\_unpaired\_JGI\_CBBP12231\_fwd | | | | | | | | | | | | | |
| E | 0007409 | axonogenesis | EN-124k-90-group11586.jgi\_paired\_JGI\_CBBP13551\_fwd | | | | | | | | | | | | |  |
| E | 0007409 | axonogenesis | EN-124k-90-group11708.jgi\_contig\_JGI\_CBBP516\_fwd | | | | | | | | | | | | |  |
| A | 0007409 | axonogenesis | EN-124k-90-group11738.gs\_53113 | | | | | | | | | |  |  |  |  |
| E | 0007409 | axonogenesis | EN-124k-90-group11780.jgi\_paired\_JGI\_CBBP12332\_fwd | | | | | | | | | | | | |  |
| A | 0007409 | axonogenesis | EN-124k-90-group11821.gs\_86440 | | | | | | | | | |  |  |  |  |
| A | 0007409 | axonogenesis | EN-124k-90-group11865.gs\_82011 | | | | | | | | | |  |  |  |  |
| E | 0007409 | axonogenesis | EN-124k-90-group11878.EN\_iowa\_18095 | | | | | | | | | | |  |  |  |
| E | 0007409 | axonogenesis | EN-124k-90-group11882.EN\_iowa\_9262 | | | | | | | | | | |  |  |  |
| E | 0007409 | axonogenesis | EN-124k-90-group11917.jgi\_paired\_JGI\_CBBP3416\_fwd | | | | | | | | | | | | |  |
| E | 0007409 | axonogenesis | EN-124k-90-group12007.jgi\_paired\_JGI\_CBBP14791\_fwd | | | | | | | | | | | | |  |
| E | 0007409 | axonogenesis | EN-124k-90-group12012.jgi\_contig\_JGI\_CBBP10480\_fwd | | | | | | | | | | | | |  |
| A | 0007409 | axonogenesis | EN-124k-90-group12032.gs\_85627 | | | | | | | | | |  |  |  |  |
| A | 0007409 | axonogenesis | EN-124k-90-group12066.gs\_43214 | | | | | | | | | |  |  |  |  |
| A | 0007409 | axonogenesis | EN-124k-90-group12082.gs\_71967 | | | | | | | | | |  |  |  |  |
| E | 0007409 | axonogenesis | EN-124k-90-group12191.jgi\_paired\_JGI\_CBBP10887\_fwd | | | | | | | | | | | | |  |
| A | 0007409 | axonogenesis | EN-124k-90-group12204.gs\_9947 | | | | | | | | | |  |  |  |  |
| E | 0007409 | axonogenesis | EN-124k-90-group12452.EN\_iowa\_9722 | | | | | | | | | | |  |  |  |
| E | 0007409 | axonogenesis | EN-124k-90-group12477.jgi\_paired\_JGI\_CBBP10794\_fwd | | | | | | | | | | | | |  |
| E | 0007409 | axonogenesis | EN-124k-90-group12537.jgi\_contig\_JGI\_CBBP17605\_fwd | | | | | | | | | | | | |  |
| E | 0007409 | axonogenesis | EN-124k-90-group12563.EN\_iowa\_9742 | | | | | | | | | | |  |  |  |
| E | 0007409 | axonogenesis | EN-124k-90-group12595.jgi\_contig\_JGI\_CBBP15955\_fwd | | | | | | | | | | | | |  |
| E | 0007409 | axonogenesis | EN-124k-90-group12676.jgi\_paired\_JGI\_CBBP17511\_rev | | | | | | | | | | | | |  |
| E | 0007409 | axonogenesis | EN-124k-90-group12683.jgi\_paired\_JGI\_CBBP11245\_fwd | | | | | | | | | | | | |  |
| E | 0007409 | axonogenesis | EN-124k-90-group12721.EN\_iowa\_5331 | | | | | | | | | | |  |  |  |
| A | 0007409 | axonogenesis | EN-124k-90-group12740.gs\_56121 | | | | | | | | | |  |  |  |  |
| E | 0007409 | axonogenesis | EN-124k-90-group12744.EN\_iowa\_4256 | | | | | | | | | | |  |  |  |
| A | 0007409 | axonogenesis | EN-124k-90-group12821.gs\_58803 | | | | | | | | | |  |  |  |  |
| A | 0007409 | axonogenesis | EN-124k-90-group12946.gs\_86900 | | | | | | | | | |  |  |  |  |
| A | 0007409 | axonogenesis | EN-124k-90-group13006.gs\_33814 | | | | | | | | | |  |  |  |  |
| E | 0007409 | axonogenesis | EN-124k-90-group13011.jgi\_paired\_JGI\_CBBP19322\_fwd | | | | | | | | | | | | |  |
| E | 0007409 | axonogenesis | EN-124k-90-group13023.EN\_iowa\_251 | | | | | | | | | | |  |  |  |
| E | 0007409 | axonogenesis | EN-124k-90-group13146.jgi\_paired\_JGI\_CBBP17160\_fwd | | | | | | | | | | | | |  |
| E | 0007409 | axonogenesis | EN-124k-90-group13346.jgi\_unpaired\_JGI\_CBBP5460\_rev | | | | | | | | | | | | | |
| E | 0007409 | axonogenesis | EN-124k-90-group13368.jgi\_unpaired\_JGI\_CBBP5492\_rev | | | | | | | | | | | | | |
| E | 0007409 | axonogenesis | EN-124k-90-group13382.jgi\_contig\_JGI\_CBBP6648\_fwd | | | | | | | | | | | | |  |
| A | 0007409 | axonogenesis | EN-124k-90-group13390.gs\_82388 | | | | | | | | | |  |  |  |  |
| E | 0007409 | axonogenesis | EN-124k-90-group13505.EN\_iowa\_4386 | | | | | | | | | | |  |  |  |
| A | 0007409 | axonogenesis | EN-124k-90-group13506.gs\_86402 | | | | | | | | | |  |  |  |  |
| E | 0007409 | axonogenesis | EN-124k-90-group13508.EN\_iowa\_12581 | | | | | | | | | | |  |  |  |
| E | 0007409 | axonogenesis | EN-124k-90-group13515.EN\_iowa\_10440 | | | | | | | | | | |  |  |  |
| E | 0007409 | axonogenesis | EN-124k-90-group13547.jgi\_contig\_JGI\_CBBP6263\_fwd | | | | | | | | | | | | |  |
| A | 0007409 | axonogenesis | EN-124k-90-group13721.gs\_13610 | | | | | | | | | |  |  |  |  |
| A | 0007409 | axonogenesis | EN-124k-90-group13771.gs\_28668 | | | | | | | | | |  |  |  |  |
| A | 0007409 | axonogenesis | EN-124k-90-group13823.gs\_32410 | | | | | | | | | |  |  |  |  |
| E | 0007409 | axonogenesis | EN-124k-90-group13906.jgi\_paired\_JGI\_CBBP12965\_fwd | | | | | | | | | | | | |  |
| E | 0007409 | axonogenesis | EN-124k-90-group13916.EN\_iowa\_14815 | | | | | | | | | | |  |  |  |
| A | 0007409 | axonogenesis | EN-124k-90-group13938.gs\_32571 | | | | | | | | | |  |  |  |  |
| E | 0007409 | axonogenesis | EN-124k-90-group13951.jgi\_paired\_JGI\_CBBP12336\_fwd | | | | | | | | | | | | |  |
| A | 0007409 | axonogenesis | EN-124k-90-group14040.gs\_63341 | | | | | | | | | |  |  |  |  |
| E | 0007409 | axonogenesis | EN-124k-90-group14050.jgi\_paired\_JGI\_CBBP18955\_fwd | | | | | | | | | | | | |  |
| E | 0007409 | axonogenesis | EN-124k-90-group14114.jgi\_paired\_JGI\_CBBP9776\_fwd | | | | | | | | | | | | |  |
| A | 0007409 | axonogenesis | EN-124k-90-group14128.gs\_38861 | | | | | | | | | |  |  |  |  |
| E | 0007409 | axonogenesis | EN-124k-90-group14311.jgi\_paired\_JGI\_CBBP682\_fwd | | | | | | | | | | | | |  |
| A | 0007409 | axonogenesis | EN-124k-90-group14320.gs\_86221 | | | | | | | | | |  |  |  |  |
| E | 0007409 | axonogenesis | EN-124k-90-group14335.jgi\_paired\_JGI\_CBBP12938\_fwd | | | | | | | | | | | | |  |
| A | 0007409 | axonogenesis | EN-124k-90-group14351.gs\_85401 | | | | | | | | | |  |  |  |  |
| E | 0007409 | axonogenesis | EN-124k-90-group14452.jgi\_paired\_JGI\_CBBP10500\_fwd | | | | | | | | | | | | |  |
| E | 0007409 | axonogenesis | EN-124k-90-group14475.jgi\_paired\_JGI\_CBBP15870\_rev | | | | | | | | | | | | |  |
| E | 0007409 | axonogenesis | EN-124k-90-group14509.jgi\_paired\_JGI\_CBBP933\_fwd | | | | | | | | | | | | |  |
| E | 0007409 | axonogenesis | EN-124k-90-group14544.jgi\_contig\_JGI\_CBBP14322\_fwd | | | | | | | | | | | | |  |
| A | 0007409 | axonogenesis | EN-124k-90-group14563.gs\_48486 | | | | | | | | | |  |  |  |  |
| E | 0007409 | axonogenesis | EN-124k-90-group14573.jgi\_paired\_JGI\_CBBP20040\_fwd | | | | | | | | | | | | |  |
| A | 0007409 | axonogenesis | EN-124k-90-group14582.gs\_17139 | | | | | | | | | |  |  |  |  |
| A | 0007409 | axonogenesis | EN-124k-90-group14609.gs\_33389 | | | | | | | | | |  |  |  |  |
| A | 0007409 | axonogenesis | EN-124k-90-group14734.gs\_78905 | | | | | | | | | |  |  |  |  |
| A | 0007409 | axonogenesis | EN-124k-90-group14813.gs\_84560 | | | | | | | | | |  |  |  |  |
| E | 0007409 | axonogenesis | EN-124k-90-group14826.jgi\_paired\_JGI\_CBBP20200\_fwd | | | | | | | | | | | | |  |
| A | 0007409 | axonogenesis | EN-124k-90-group14887.gs\_61232 | | | | | | | | | |  |  |  |  |
| E | 0007409 | axonogenesis | EN-124k-90-group14931.jgi\_paired\_JGI\_CBBP10444\_fwd | | | | | | | | | | | | |  |
| E | 0007409 | axonogenesis | EN-124k-90-group14935.jgi\_contig\_JGI\_CBBP17959\_fwd | | | | | | | | | | | | |  |
| A | 0007409 | axonogenesis | EN-124k-90-group14948.gs\_26769 | | | | | | | | | |  |  |  |  |
| A | 0007409 | axonogenesis | EN-124k-90-group15056.gs\_73097 | | | | | | | | | |  |  |  |  |
| A | 0007409 | axonogenesis | EN-124k-90-group15062.gs\_40000 | | | | | | | | | |  |  |  |  |
| A | 0007409 | axonogenesis | EN-124k-90-group15124.gs\_82644 | | | | | | | | | |  |  |  |  |
| E | 0007409 | axonogenesis | EN-124k-90-group15223.EN\_iowa\_18438 | | | | | | | | | | |  |  |  |
| A | 0007409 | axonogenesis | EN-124k-90-group15229.gs\_75551 | | | | | | | | | |  |  |  |  |
| A | 0007409 | axonogenesis | EN-124k-90-group15268.gs\_21540 | | | | | | | | | |  |  |  |  |
| E | 0007409 | axonogenesis | EN-124k-90-group15289.jgi\_paired\_JGI\_CBBP6226\_fwd | | | | | | | | | | | | |  |
| E | 0007409 | axonogenesis | EN-124k-90-group15290.EN\_iowa\_9903 | | | | | | | | | | |  |  |  |
| E | 0007409 | axonogenesis | EN-124k-90-group15320.jgi\_paired\_JGI\_CBBP9817\_fwd | | | | | | | | | | | | |  |
| E | 0007409 | axonogenesis | EN-124k-90-group15378.jgi\_contig\_JGI\_CBBP10913\_fwd | | | | | | | | | | | | |  |
| A | 0007409 | axonogenesis | EN-124k-90-group15397.gs\_22489 | | | | | | | | | |  |  |  |  |
| A | 0007409 | axonogenesis | EN-124k-90-group15399.gs\_80457 | | | | | | | | | |  |  |  |  |
| A | 0007409 | axonogenesis | EN-124k-90-group15413.gs\_80915 | | | | | | | | | |  |  |  |  |
| E | 0007409 | axonogenesis | EN-124k-90-group15532.EN\_iowa\_5883 | | | | | | | | | | |  |  |  |
| E | 0007409 | axonogenesis | EN-124k-90-group15546.EN\_iowa\_13502 | | | | | | | | | | |  |  |  |
| E | 0007409 | axonogenesis | EN-124k-90-group15553.EN\_iowa\_14509 | | | | | | | | | | |  |  |  |
| A | 0007409 | axonogenesis | EN-124k-90-group15556.gs\_43579 | | | | | | | | | |  |  |  |  |
| E | 0007409 | axonogenesis | EN-124k-90-group15588.jgi\_paired\_JGI\_CBBP15154\_fwd | | | | | | | | | | | | |  |
| E | 0007409 | axonogenesis | EN-124k-90-group15601.jgi\_paired\_JGI\_CBBP13413\_fwd | | | | | | | | | | | | |  |
| E | 0007409 | axonogenesis | EN-124k-90-group15671.jgi\_paired\_JGI\_CBBP10266\_fwd | | | | | | | | | | | | |  |
| E | 0007409 | axonogenesis | EN-124k-90-group15692.jgi\_contig\_JGI\_CBBP12510\_fwd | | | | | | | | | | | | |  |
| E | 0007409 | axonogenesis | EN-124k-90-group15723.jgi\_paired\_JGI\_CBBP16981\_fwd | | | | | | | | | | | | |  |
| A | 0007409 | axonogenesis | EN-124k-90-group15749.gs\_84109 | | | | | | | | | |  |  |  |  |
| A | 0007409 | axonogenesis | EN-124k-90-group15770.gs\_45569 | | | | | | | | | |  |  |  |  |
| E | 0007409 | axonogenesis | EN-124k-90-group15776.EN\_iowa\_1971 | | | | | | | | | | |  |  |  |
| E | 0007409 | axonogenesis | EN-124k-90-group15789.jgi\_paired\_JGI\_CBBP2907\_fwd | | | | | | | | | | | | |  |
| A | 0007409 | axonogenesis | EN-124k-90-group15856.gs\_15241 | | | | | | | | | |  |  |  |  |
| E | 0007409 | axonogenesis | EN-124k-90-group15877.EN\_iowa\_3358 | | | | | | | | | | |  |  |  |
| E | 0007409 | axonogenesis | EN-124k-90-group15927.EN\_iowa\_7602 | | | | | | | | | | |  |  |  |
| E | 0007409 | axonogenesis | EN-124k-90-group15944.jgi\_paired\_JGI\_CBBP5778\_fwd | | | | | | | | | | | | |  |
| E | 0007409 | axonogenesis | EN-124k-90-group15994.jgi\_paired\_JGI\_CBBP14428\_fwd | | | | | | | | | | | | |  |
| A | 0007409 | axonogenesis | EN-124k-90-group16026.gs\_15943 | | | | | | | | | |  |  |  |  |
| A | 0007409 | axonogenesis | EN-124k-90-group16067.gs\_19008 | | | | | | | | | |  |  |  |  |
| E | 0007409 | axonogenesis | EN-124k-90-group16134.jgi\_contig\_JGI\_CBBP14519\_fwd | | | | | | | | | | | | |  |
| E | 0007409 | axonogenesis | EN-124k-90-group16143.jgi\_paired\_JGI\_CBBP4408\_fwd | | | | | | | | | | | | |  |
| A | 0007409 | axonogenesis | EN-124k-90-group16149.gs\_13439 | | | | | | | | | |  |  |  |  |
| A | 0007409 | axonogenesis | EN-124k-90-group16189.gs\_70159 | | | | | | | | | |  |  |  |  |
| E | 0007409 | axonogenesis | EN-124k-90-group16200.jgi\_paired\_JGI\_CBBP10444\_rev | | | | | | | | | | | | |  |
| A | 0007409 | axonogenesis | EN-124k-90-group16285.gs\_47129 | | | | | | | | | |  |  |  |  |
| A | 0007409 | axonogenesis | EN-124k-90-group16291.gs\_65588 | | | | | | | | | |  |  |  |  |
| A | 0007409 | axonogenesis | EN-124k-90-group16323.gs\_48303 | | | | | | | | | |  |  |  |  |
| E | 0007409 | axonogenesis | EN-124k-90-group16346.jgi\_contig\_JGI\_CBBP6214\_fwd | | | | | | | | | | | | |  |
| A | 0007409 | axonogenesis | EN-124k-90-group16399.gs\_80314 | | | | | | | | | |  |  |  |  |
| A | 0007409 | axonogenesis | EN-124k-90-group16412.gs\_64548 | | | | | | | | | |  |  |  |  |
| A | 0007409 | axonogenesis | EN-124k-90-group16416.gs\_28586 | | | | | | | | | |  |  |  |  |
| A | 0007409 | axonogenesis | EN-124k-90-group16439.gs\_54729 | | | | | | | | | |  |  |  |  |
| A | 0007409 | axonogenesis | EN-124k-90-group16443.gs\_57863 | | | | | | | | | |  |  |  |  |
| A | 0007409 | axonogenesis | EN-124k-90-group16489.gs\_80464 | | | | | | | | | |  |  |  |  |
| A | 0007409 | axonogenesis | EN-124k-90-group16520.gs\_62867 | | | | | | | | | |  |  |  |  |
| A | 0007409 | axonogenesis | EN-124k-90-group16522.gs\_78634 | | | | | | | | | |  |  |  |  |
| A | 0007409 | axonogenesis | EN-124k-90-group16523.gs\_75670 | | | | | | | | | |  |  |  |  |
| A | 0007409 | axonogenesis | EN-124k-90-group16598.gs\_3233 | | | | | | | | | |  |  |  |  |
| A | 0007409 | axonogenesis | EN-124k-90-group16636.gs\_429 | | | | | | | | | |  |  |  |  |
| A | 0007409 | axonogenesis | EN-124k-90-group16714.gs\_320 | | | | | | | | | |  |  |  |  |
| A | 0007409 | axonogenesis | EN-124k-90-group16826.gs\_5886 | | | | | | | | | |  |  |  |  |
| A | 0007409 | axonogenesis | EN-124k-90-group16830.gs\_82512 | | | | | | | | | |  |  |  |  |
| A | 0007409 | axonogenesis | EN-124k-90-group16845.gs\_50498 | | | | | | | | | |  |  |  |  |
| A | 0007409 | axonogenesis | EN-124k-90-group16851.gs\_64557 | | | | | | | | | |  |  |  |  |
| A | 0007409 | axonogenesis | EN-124k-90-group16852.gs\_27372 | | | | | | | | | |  |  |  |  |
| A | 0007409 | axonogenesis | EN-124k-90-group16857.gs\_13788 | | | | | | | | | |  |  |  |  |
| A | 0007409 | axonogenesis | EN-124k-90-group16872.gs\_213 | | | | | | | | | |  |  |  |  |
| A | 0007409 | axonogenesis | EN-124k-90-group16910.gs\_22871 | | | | | | | | | |  |  |  |  |
| A | 0007409 | axonogenesis | EN-124k-90-group16929.gs\_43290 | | | | | | | | | |  |  |  |  |
| A | 0007409 | axonogenesis | EN-124k-90-group16932.gs\_197 | | | | | | | | | |  |  |  |  |
| A | 0007409 | axonogenesis | EN-124k-90-group17012.gs\_52263 | | | | | | | | | |  |  |  |  |
| A | 0007409 | axonogenesis | EN-124k-90-group17022.gs\_80073 | | | | | | | | | |  |  |  |  |
| E | 0007409 | axonogenesis | EN-124k-90-group17086.jgi\_paired\_JGI\_CBBP6417\_rev | | | | | | | | | | | | |  |
| E | 0007409 | axonogenesis | EN-124k-90-group17091.jgi\_paired\_JGI\_CBBP10179\_rev | | | | | | | | | | | | |  |
| A | 0007409 | axonogenesis | EN-124k-90-group17117.gs\_84807 | | | | | | | | | |  |  |  |  |
| A | 0007409 | axonogenesis | EN-124k-90-group17129.gs\_61289 | | | | | | | | | |  |  |  |  |
| A | 0007409 | axonogenesis | EN-124k-90-group17140.gs\_14565 | | | | | | | | | |  |  |  |  |
| A | 0007409 | axonogenesis | EN-124k-90-group17161.gs\_56414 | | | | | | | | | |  |  |  |  |
| A | 0007409 | axonogenesis | EN-124k-90-group17210.gs\_64350 | | | | | | | | | |  |  |  |  |
| A | 0007409 | axonogenesis | EN-124k-90-group17273.gs\_68617 | | | | | | | | | |  |  |  |  |
| A | 0007409 | axonogenesis | EN-124k-90-group17276.gs\_80679 | | | | | | | | | |  |  |  |  |
| A | 0007409 | axonogenesis | EN-124k-90-group17301.gs\_75053 | | | | | | | | | |  |  |  |  |
| A | 0007409 | axonogenesis | EN-124k-90-group17321.gs\_60379 | | | | | | | | | |  |  |  |  |
| A | 0007409 | axonogenesis | EN-124k-90-group17360.gs\_72469 | | | | | | | | | |  |  |  |  |
| A | 0007409 | axonogenesis | EN-124k-90-group17386.gs\_68411 | | | | | | | | | |  |  |  |  |
| A | 0007409 | axonogenesis | EN-124k-90-group17411.gs\_13940 | | | | | | | | | |  |  |  |  |
| A | 0007409 | axonogenesis | EN-124k-90-group17433.gs\_222 | | | | | | | | | |  |  |  |  |
| A | 0007409 | axonogenesis | EN-124k-90-group17442.gs\_63748 | | | | | | | | | |  |  |  |  |
| A | 0007409 | axonogenesis | EN-124k-90-group17445.gs\_83255 | | | | | | | | | |  |  |  |  |
| A | 0007409 | axonogenesis | EN-124k-90-group17471.gs\_85794 | | | | | | | | | |  |  |  |  |
| A | 0007409 | axonogenesis | EN-124k-90-group17487.gs\_82041 | | | | | | | | | |  |  |  |  |
| A | 0007409 | axonogenesis | EN-124k-90-group17524.gs\_80802 | | | | | | | | | |  |  |  |  |
| A | 0007409 | axonogenesis | EN-124k-90-group17556.gs\_87114 | | | | | | | | | |  |  |  |  |
| A | 0007409 | axonogenesis | EN-124k-90-group17563.gs\_80410 | | | | | | | | | |  |  |  |  |
| A | 0007409 | axonogenesis | EN-124k-90-group17567.gs\_58610 | | | | | | | | | |  |  |  |  |
| A | 0007409 | axonogenesis | EN-124k-90-group17572.gs\_7187 | | | | | | | | | |  |  |  |  |
| A | 0007409 | axonogenesis | EN-124k-90-group17582.gs\_70636 | | | | | | | | | |  |  |  |  |
| A | 0007409 | axonogenesis | EN-124k-90-group17650.gs\_59359 | | | | | | | | | |  |  |  |  |
| A | 0007409 | axonogenesis | EN-124k-90-group17657.gs\_68076 | | | | | | | | | |  |  |  |  |
| A | 0007409 | axonogenesis | EN-124k-90-group17679.gs\_71618 | | | | | | | | | |  |  |  |  |
| A | 0007409 | axonogenesis | EN-124k-90-group17702.gs\_18827 | | | | | | | | | |  |  |  |  |
| A | 0007409 | axonogenesis | EN-124k-90-group17743.gs\_53689 | | | | | | | | | |  |  |  |  |
| A | 0007409 | axonogenesis | EN-124k-90-group17744.gs\_30255 | | | | | | | | | |  |  |  |  |
| A | 0007409 | axonogenesis | EN-124k-90-group17776.gs\_56285 | | | | | | | | | |  |  |  |  |
| A | 0007409 | axonogenesis | EN-124k-90-group17819.gs\_51352 | | | | | | | | | |  |  |  |  |
| A | 0007409 | axonogenesis | EN-124k-90-group17864.gs\_35131 | | | | | | | | | |  |  |  |  |
| A | 0007409 | axonogenesis | EN-124k-90-group17887.gs\_80072 | | | | | | | | | |  |  |  |  |
| A | 0007409 | axonogenesis | EN-124k-90-group17892.gs\_49260 | | | | | | | | | |  |  |  |  |
| A | 0007409 | axonogenesis | EN-124k-90-group17930.gs\_83513 | | | | | | | | | |  |  |  |  |
| E | 0007409 | axonogenesis | EN-124k-90-group17943.EN\_iowa\_18374 | | | | | | | | | | |  |  |  |
| A | 0007409 | axonogenesis | EN-124k-90-group17951.gs\_43129 | | | | | | | | | |  |  |  |  |
| E | 0007409 | axonogenesis | EN-124k-90-group17979.jgi\_contig\_JGI\_CBBP2421\_fwd | | | | | | | | | | | | |  |
| A | 0007409 | axonogenesis | EN-124k-90-group18006.gs\_18909 | | | | | | | | | |  |  |  |  |
| A | 0007409 | axonogenesis | EN-124k-90-group18033.gs\_11467 | | | | | | | | | |  |  |  |  |
| A | 0007409 | axonogenesis | EN-124k-90-group18038.gs\_30262 | | | | | | | | | |  |  |  |  |
| A | 0007409 | axonogenesis | EN-124k-90-group18140.gs\_87168 | | | | | | | | | |  |  |  |  |
| A | 0007409 | axonogenesis | EN-124k-90-group18148.gs\_80788 | | | | | | | | | |  |  |  |  |
| A | 0007409 | axonogenesis | EN-124k-90-group18205.gs\_57120 | | | | | | | | | |  |  |  |  |
| A | 0007409 | axonogenesis | EN-124k-90-group18238.gs\_39782 | | | | | | | | | |  |  |  |  |
| A | 0007409 | axonogenesis | EN-124k-90-group18245.gs\_78006 | | | | | | | | | |  |  |  |  |
| A | 0007409 | axonogenesis | EN-124k-90-group18289.gs\_45417 | | | | | | | | | |  |  |  |  |
| A | 0007409 | axonogenesis | EN-124k-90-group18372.gs\_75667 | | | | | | | | | |  |  |  |  |
| E | 0007409 | axonogenesis | EN-124k-90-group18375.EN\_iowa\_3673 | | | | | | | | | | |  |  |  |
| A | 0007409 | axonogenesis | EN-124k-90-group18409.gs\_84438 | | | | | | | | | |  |  |  |  |
| A | 0007409 | axonogenesis | EN-124k-90-group18447.gs\_40187 | | | | | | | | | |  |  |  |  |
| A | 0007409 | axonogenesis | EN-124k-90-group18480.gs\_449 | | | | | | | | | |  |  |  |  |
| A | 0007409 | axonogenesis | EN-124k-90-group18526.gs\_58285 | | | | | | | | | |  |  |  |  |
| E | 0007409 | axonogenesis | EN-124k-90-group18538.jgi\_paired\_JGI\_CBBP19133\_rev | | | | | | | | | | | | |  |
| A | 0007409 | axonogenesis | EN-124k-90-group18592.gs\_31083 | | | | | | | | | |  |  |  |  |
| A | 0007409 | axonogenesis | EN-124k-90-group18634.gs\_80302 | | | | | | | | | |  |  |  |  |
| A | 0007409 | axonogenesis | EN-124k-90-group18774.gs\_57540 | | | | | | | | | |  |  |  |  |
| A | 0007409 | axonogenesis | EN-124k-90-group18840.gs\_32731 | | | | | | | | | |  |  |  |  |
| A | 0007409 | axonogenesis | EN-124k-90-group18887.gs\_36301 | | | | | | | | | |  |  |  |  |
| A | 0007409 | axonogenesis | EN-124k-90-group18929.gs\_13816 | | | | | | | | | |  |  |  |  |
| A | 0007409 | axonogenesis | EN-124k-90-group18948.gs\_56541 | | | | | | | | | |  |  |  |  |
| M | 0007409 | axonogenesis | EN-124k-90-group10.Contig1 | | | | | | | | |  |  |  |  |  |
| M | 0007409 | axonogenesis | EN-124k-90-group29.Contig2 | | | | | | | | |  |  |  |  |  |
| M | 0007409 | axonogenesis | EN-124k-90-group29.Contig3 | | | | | | | | |  |  |  |  |  |
| M | 0007409 | axonogenesis | EN-124k-90-group29.Contig7 | | | | | | | | |  |  |  |  |  |
| M | 0007409 | axonogenesis | EN-124k-90-group29.Contig9 | | | | | | | | |  |  |  |  |  |
| M | 0007409 | axonogenesis | EN-124k-90-group29.Contig13 | | | | | | | | | |  |  |  |  |
| M | 0007409 | axonogenesis | EN-124k-90-group29.Contig15 | | | | | | | | | |  |  |  |  |
| M | 0007409 | axonogenesis | EN-124k-90-group43.Contig1 | | | | | | | | |  |  |  |  |  |
| M | 0007409 | axonogenesis | EN-124k-90-group63.Contig2 | | | | | | | | |  |  |  |  |  |
| M | 0007409 | axonogenesis | EN-124k-90-group70.Contig3 | | | | | | | | |  |  |  |  |  |
| M | 0007409 | axonogenesis | EN-124k-90-group70.Contig17 | | | | | | | | | |  |  |  |  |
| M | 0007409 | axonogenesis | EN-124k-90-group75.Contig2 | | | | | | | | |  |  |  |  |  |
| M | 0007409 | axonogenesis | EN-124k-90-group138.Contig1 | | | | | | | | | |  |  |  |  |
| M | 0007409 | axonogenesis | EN-124k-90-group138.Contig35 | | | | | | | | | |  |  |  |  |
| M | 0007409 | axonogenesis | EN-124k-90-group138.Contig62 | | | | | | | | | |  |  |  |  |
| M | 0007409 | axonogenesis | EN-124k-90-group186.Contig2 | | | | | | | | | |  |  |  |  |
| M | 0007409 | axonogenesis | EN-124k-90-group229.Contig1 | | | | | | | | | |  |  |  |  |
| M | 0007409 | axonogenesis | EN-124k-90-group230.Contig1 | | | | | | | | | |  |  |  |  |
| M | 0007409 | axonogenesis | EN-124k-90-group232.Contig1 | | | | | | | | | |  |  |  |  |
| M | 0007409 | axonogenesis | EN-124k-90-group232.Contig2 | | | | | | | | | |  |  |  |  |
| M | 0007409 | axonogenesis | EN-124k-90-group232.Contig3 | | | | | | | | | |  |  |  |  |
| M | 0007409 | axonogenesis | EN-124k-90-group232.Contig5 | | | | | | | | | |  |  |  |  |
| M | 0007409 | axonogenesis | EN-124k-90-group245.Contig3 | | | | | | | | | |  |  |  |  |
| M | 0007409 | axonogenesis | EN-124k-90-group245.Contig28 | | | | | | | | | |  |  |  |  |
| M | 0007409 | axonogenesis | EN-124k-90-group253.Contig1 | | | | | | | | | |  |  |  |  |
| M | 0007409 | axonogenesis | EN-124k-90-group265.Contig1 | | | | | | | | | |  |  |  |  |
| M | 0007409 | axonogenesis | EN-124k-90-group270.Contig2 | | | | | | | | | |  |  |  |  |
| M | 0007409 | axonogenesis | EN-124k-90-group294.Contig1 | | | | | | | | | |  |  |  |  |
| M | 0007409 | axonogenesis | EN-124k-90-group294.Contig3 | | | | | | | | | |  |  |  |  |
| M | 0007409 | axonogenesis | EN-124k-90-group304.Contig1 | | | | | | | | | |  |  |  |  |
| M | 0007409 | axonogenesis | EN-124k-90-group324.Contig1 | | | | | | | | | |  |  |  |  |
| M | 0007409 | axonogenesis | EN-124k-90-group328.Contig2 | | | | | | | | | |  |  |  |  |
| M | 0007409 | axonogenesis | EN-124k-90-group382.Contig1 | | | | | | | | | |  |  |  |  |
| M | 0007409 | axonogenesis | EN-124k-90-group382.Contig2 | | | | | | | | | |  |  |  |  |
| M | 0007409 | axonogenesis | EN-124k-90-group430.Contig1 | | | | | | | | | |  |  |  |  |
| M | 0007409 | axonogenesis | EN-124k-90-group462.Contig1 | | | | | | | | | |  |  |  |  |
| M | 0007409 | axonogenesis | EN-124k-90-group481.Contig4 | | | | | | | | | |  |  |  |  |
| M | 0007409 | axonogenesis | EN-124k-90-group506.Contig1 | | | | | | | | | |  |  |  |  |
| M | 0007409 | axonogenesis | EN-124k-90-group506.Contig3 | | | | | | | | | |  |  |  |  |
| M | 0007409 | axonogenesis | EN-124k-90-group506.Contig4 | | | | | | | | | |  |  |  |  |
| M | 0007409 | axonogenesis | EN-124k-90-group509.Contig1 | | | | | | | | | |  |  |  |  |
| M | 0007409 | axonogenesis | EN-124k-90-group509.Contig3 | | | | | | | | | |  |  |  |  |
| M | 0007409 | axonogenesis | EN-124k-90-group513.Contig1 | | | | | | | | | |  |  |  |  |
| M | 0007409 | axonogenesis | EN-124k-90-group513.Contig2 | | | | | | | | | |  |  |  |  |
| M | 0007409 | axonogenesis | EN-124k-90-group516.Contig2 | | | | | | | | | |  |  |  |  |
| M | 0007409 | axonogenesis | EN-124k-90-group518.Contig2 | | | | | | | | | |  |  |  |  |
| M | 0007409 | axonogenesis | EN-124k-90-group524.Contig1 | | | | | | | | | |  |  |  |  |
| M | 0007409 | axonogenesis | EN-124k-90-group524.Contig2 | | | | | | | | | |  |  |  |  |
| M | 0007409 | axonogenesis | EN-124k-90-group524.Contig3 | | | | | | | | | |  |  |  |  |
| M | 0007409 | axonogenesis | EN-124k-90-group530.Contig2 | | | | | | | | | |  |  |  |  |
| M | 0007409 | axonogenesis | EN-124k-90-group543.Contig1 | | | | | | | | | |  |  |  |  |
| M | 0007409 | axonogenesis | EN-124k-90-group550.Contig1 | | | | | | | | | |  |  |  |  |
| M | 0007409 | axonogenesis | EN-124k-90-group550.Contig2 | | | | | | | | | |  |  |  |  |
| M | 0007409 | axonogenesis | EN-124k-90-group564.Contig5 | | | | | | | | | |  |  |  |  |
| M | 0007409 | axonogenesis | EN-124k-90-group583.Contig1 | | | | | | | | | |  |  |  |  |
| M | 0007409 | axonogenesis | EN-124k-90-group586.Contig1 | | | | | | | | | |  |  |  |  |
| M | 0007409 | axonogenesis | EN-124k-90-group586.Contig2 | | | | | | | | | |  |  |  |  |
| M | 0007409 | axonogenesis | EN-124k-90-group593.Contig1 | | | | | | | | | |  |  |  |  |
| M | 0007409 | axonogenesis | EN-124k-90-group630.Contig1 | | | | | | | | | |  |  |  |  |
| M | 0007409 | axonogenesis | EN-124k-90-group630.Contig2 | | | | | | | | | |  |  |  |  |
| M | 0007409 | axonogenesis | EN-124k-90-group658.Contig1 | | | | | | | | | |  |  |  |  |
| M | 0007409 | axonogenesis | EN-124k-90-group658.Contig3 | | | | | | | | | |  |  |  |  |
| M | 0007409 | axonogenesis | EN-124k-90-group674.Contig1 | | | | | | | | | |  |  |  |  |
| M | 0007409 | axonogenesis | EN-124k-90-group674.Contig2 | | | | | | | | | |  |  |  |  |
| M | 0007409 | axonogenesis | EN-124k-90-group678.Contig2 | | | | | | | | | |  |  |  |  |
| M | 0007409 | axonogenesis | EN-124k-90-group682.Contig1 | | | | | | | | | |  |  |  |  |
| M | 0007409 | axonogenesis | EN-124k-90-group689.Contig4 | | | | | | | | | |  |  |  |  |
| M | 0007409 | axonogenesis | EN-124k-90-group702.Contig2 | | | | | | | | | |  |  |  |  |
| M | 0007409 | axonogenesis | EN-124k-90-group716.Contig1 | | | | | | | | | |  |  |  |  |
| M | 0007409 | axonogenesis | EN-124k-90-group735.Contig1 | | | | | | | | | |  |  |  |  |
| M | 0007409 | axonogenesis | EN-124k-90-group745.Contig1 | | | | | | | | | |  |  |  |  |
| M | 0007409 | axonogenesis | EN-124k-90-group745.Contig2 | | | | | | | | | |  |  |  |  |
| M | 0007409 | axonogenesis | EN-124k-90-group760.Contig4 | | | | | | | | | |  |  |  |  |
| E | 0007409 | axonogenesis | EN-124k-90-group792.Contig1 | | | | | | | | | |  |  |  |  |
| M | 0007409 | axonogenesis | EN-124k-90-group849.Contig2 | | | | | | | | | |  |  |  |  |
| M | 0007409 | axonogenesis | EN-124k-90-group849.Contig3 | | | | | | | | | |  |  |  |  |
| M | 0007409 | axonogenesis | EN-124k-90-group850.Contig1 | | | | | | | | | |  |  |  |  |
| M | 0007409 | axonogenesis | EN-124k-90-group868.Contig1 | | | | | | | | | |  |  |  |  |
| M | 0007409 | axonogenesis | EN-124k-90-group881.Contig1 | | | | | | | | | |  |  |  |  |
| M | 0007409 | axonogenesis | EN-124k-90-group881.Contig2 | | | | | | | | | |  |  |  |  |
| M | 0007409 | axonogenesis | EN-124k-90-group881.Contig3 | | | | | | | | | |  |  |  |  |
| M | 0007409 | axonogenesis | EN-124k-90-group882.Contig2 | | | | | | | | | |  |  |  |  |
| M | 0007409 | axonogenesis | EN-124k-90-group900.Contig1 | | | | | | | | | |  |  |  |  |
| M | 0007409 | axonogenesis | EN-124k-90-group908.Contig6 | | | | | | | | | |  |  |  |  |
| M | 0007409 | axonogenesis | EN-124k-90-group925.Contig1 | | | | | | | | | |  |  |  |  |
| M | 0007409 | axonogenesis | EN-124k-90-group925.Contig2 | | | | | | | | | |  |  |  |  |
| M | 0007409 | axonogenesis | EN-124k-90-group925.Contig3 | | | | | | | | | |  |  |  |  |
| M | 0007409 | axonogenesis | EN-124k-90-group939.Contig1 | | | | | | | | | |  |  |  |  |
| M | 0007409 | axonogenesis | EN-124k-90-group944.Contig4 | | | | | | | | | |  |  |  |  |
| M | 0007409 | axonogenesis | EN-124k-90-group945.Contig1 | | | | | | | | | |  |  |  |  |
| M | 0007409 | axonogenesis | EN-124k-90-group986.Contig2 | | | | | | | | | |  |  |  |  |
| M | 0007409 | axonogenesis | EN-124k-90-group994.Contig1 | | | | | | | | | |  |  |  |  |
| M | 0007409 | axonogenesis | EN-124k-90-group998.Contig1 | | | | | | | | | |  |  |  |  |
| M | 0007409 | axonogenesis | EN-124k-90-group1001.Contig1 | | | | | | | | | |  |  |  |  |
| M | 0007409 | axonogenesis | EN-124k-90-group1001.Contig4 | | | | | | | | | |  |  |  |  |
| M | 0007409 | axonogenesis | EN-124k-90-group1007.Contig1 | | | | | | | | | |  |  |  |  |
| M | 0007409 | axonogenesis | EN-124k-90-group1021.Contig1 | | | | | | | | | |  |  |  |  |
| M | 0007409 | axonogenesis | EN-124k-90-group1021.Contig2 | | | | | | | | | |  |  |  |  |
| M | 0007409 | axonogenesis | EN-124k-90-group1071.Contig1 | | | | | | | | | |  |  |  |  |
| M | 0007409 | axonogenesis | EN-124k-90-group1071.Contig2 | | | | | | | | | |  |  |  |  |
| M | 0007409 | axonogenesis | EN-124k-90-group1071.Contig3 | | | | | | | | | |  |  |  |  |
| M | 0007409 | axonogenesis | EN-124k-90-group1076.Contig1 | | | | | | | | | |  |  |  |  |
| M | 0007409 | axonogenesis | EN-124k-90-group1091.Contig1 | | | | | | | | | |  |  |  |  |
| M | 0007409 | axonogenesis | EN-124k-90-group1091.Contig3 | | | | | | | | | |  |  |  |  |
| E | 0007409 | axonogenesis | EN-124k-90-group1092.Contig1 | | | | | | | | | |  |  |  |  |
| M | 0007409 | axonogenesis | EN-124k-90-group1106.Contig1 | | | | | | | | | |  |  |  |  |
| M | 0007409 | axonogenesis | EN-124k-90-group1135.Contig1 | | | | | | | | | |  |  |  |  |
| M | 0007409 | axonogenesis | EN-124k-90-group1135.Contig3 | | | | | | | | | |  |  |  |  |
| M | 0007409 | axonogenesis | EN-124k-90-group1135.Contig4 | | | | | | | | | |  |  |  |  |
| M | 0007409 | axonogenesis | EN-124k-90-group1135.Contig5 | | | | | | | | | |  |  |  |  |
| M | 0007409 | axonogenesis | EN-124k-90-group1135.Contig7 | | | | | | | | | |  |  |  |  |
| M | 0007409 | axonogenesis | EN-124k-90-group1142.Contig8 | | | | | | | | | |  |  |  |  |
| M | 0007409 | axonogenesis | EN-124k-90-group1142.Contig10 | | | | | | | | | |  |  |  |  |
| M | 0007409 | axonogenesis | EN-124k-90-group1153.Contig1 | | | | | | | | | |  |  |  |  |
| M | 0007409 | axonogenesis | EN-124k-90-group1161.Contig1 | | | | | | | | | |  |  |  |  |
| M | 0007409 | axonogenesis | EN-124k-90-group1161.Contig2 | | | | | | | | | |  |  |  |  |
| M | 0007409 | axonogenesis | EN-124k-90-group1167.Contig2 | | | | | | | | | |  |  |  |  |
| M | 0007409 | axonogenesis | EN-124k-90-group1167.Contig3 | | | | | | | | | |  |  |  |  |
| M | 0007409 | axonogenesis | EN-124k-90-group1182.Contig1 | | | | | | | | | |  |  |  |  |
| M | 0007409 | axonogenesis | EN-124k-90-group1182.Contig2 | | | | | | | | | |  |  |  |  |
| M | 0007409 | axonogenesis | EN-124k-90-group1182.Contig3 | | | | | | | | | |  |  |  |  |
| M | 0007409 | axonogenesis | EN-124k-90-group1182.Contig4 | | | | | | | | | |  |  |  |  |
| M | 0007409 | axonogenesis | EN-124k-90-group1212.Contig1 | | | | | | | | | |  |  |  |  |
| M | 0007409 | axonogenesis | EN-124k-90-group1215.Contig3 | | | | | | | | | |  |  |  |  |
| M | 0007409 | axonogenesis | EN-124k-90-group1215.Contig4 | | | | | | | | | |  |  |  |  |
| M | 0007409 | axonogenesis | EN-124k-90-group1272.Contig1 | | | | | | | | | |  |  |  |  |
| M | 0007409 | axonogenesis | EN-124k-90-group1290.Contig1 | | | | | | | | | |  |  |  |  |
| M | 0007409 | axonogenesis | EN-124k-90-group1290.Contig2 | | | | | | | | | |  |  |  |  |
| M | 0007409 | axonogenesis | EN-124k-90-group1290.Contig3 | | | | | | | | | |  |  |  |  |
| M | 0007409 | axonogenesis | EN-124k-90-group1290.Contig5 | | | | | | | | | |  |  |  |  |
| M | 0007409 | axonogenesis | EN-124k-90-group1290.Contig6 | | | | | | | | | |  |  |  |  |
| M | 0007409 | axonogenesis | EN-124k-90-group1290.Contig8 | | | | | | | | | |  |  |  |  |
| M | 0007409 | axonogenesis | EN-124k-90-group1290.Contig10 | | | | | | | | | |  |  |  |  |
| M | 0007409 | axonogenesis | EN-124k-90-group1294.Contig1 | | | | | | | | | |  |  |  |  |
| M | 0007409 | axonogenesis | EN-124k-90-group1302.Contig2 | | | | | | | | | |  |  |  |  |
| M | 0007409 | axonogenesis | EN-124k-90-group1302.Contig3 | | | | | | | | | |  |  |  |  |
| M | 0007409 | axonogenesis | EN-124k-90-group1302.Contig4 | | | | | | | | | |  |  |  |  |
| M | 0007409 | axonogenesis | EN-124k-90-group1302.Contig6 | | | | | | | | | |  |  |  |  |
| M | 0007409 | axonogenesis | EN-124k-90-group1304.Contig2 | | | | | | | | | |  |  |  |  |
| M | 0007409 | axonogenesis | EN-124k-90-group1304.Contig4 | | | | | | | | | |  |  |  |  |
| M | 0007409 | axonogenesis | EN-124k-90-group1304.Contig8 | | | | | | | | | |  |  |  |  |
| E | 0007409 | axonogenesis | EN-124k-90-group1344.Contig1 | | | | | | | | | |  |  |  |  |
| M | 0007409 | axonogenesis | EN-124k-90-group1351.Contig1 | | | | | | | | | |  |  |  |  |
| M | 0007409 | axonogenesis | EN-124k-90-group1373.Contig1 | | | | | | | | | |  |  |  |  |
| M | 0007409 | axonogenesis | EN-124k-90-group1408.Contig1 | | | | | | | | | |  |  |  |  |
| M | 0007409 | axonogenesis | EN-124k-90-group1408.Contig2 | | | | | | | | | |  |  |  |  |
| M | 0007409 | axonogenesis | EN-124k-90-group1423.Contig14 | | | | | | | | | |  |  |  |  |
| M | 0007409 | axonogenesis | EN-124k-90-group1423.Contig20 | | | | | | | | | |  |  |  |  |
| M | 0007409 | axonogenesis | EN-124k-90-group1423.Contig22 | | | | | | | | | |  |  |  |  |
| M | 0007409 | axonogenesis | EN-124k-90-group1456.Contig4 | | | | | | | | | |  |  |  |  |
| M | 0007409 | axonogenesis | EN-124k-90-group1456.Contig7 | | | | | | | | | |  |  |  |  |
| M | 0007409 | axonogenesis | EN-124k-90-group1456.Contig8 | | | | | | | | | |  |  |  |  |
| M | 0007409 | axonogenesis | EN-124k-90-group1462.Contig1 | | | | | | | | | |  |  |  |  |
| M | 0007409 | axonogenesis | EN-124k-90-group1465.Contig1 | | | | | | | | | |  |  |  |  |
| M | 0007409 | axonogenesis | EN-124k-90-group1465.Contig2 | | | | | | | | | |  |  |  |  |
| A | 0007409 | axonogenesis | EN-124k-90-group1482.Contig1 | | | | | | | | | |  |  |  |  |
| M | 0007409 | axonogenesis | EN-124k-90-group1493.Contig2 | | | | | | | | | |  |  |  |  |
| A | 0007409 | axonogenesis | EN-124k-90-group1521.Contig1 | | | | | | | | | |  |  |  |  |
| A | 0007409 | axonogenesis | EN-124k-90-group1521.Contig2 | | | | | | | | | |  |  |  |  |
| A | 0007409 | axonogenesis | EN-124k-90-group1521.Contig3 | | | | | | | | | |  |  |  |  |
| M | 0007409 | axonogenesis | EN-124k-90-group1542.Contig1 | | | | | | | | | |  |  |  |  |
| M | 0007409 | axonogenesis | EN-124k-90-group1549.Contig1 | | | | | | | | | |  |  |  |  |
| M | 0007409 | axonogenesis | EN-124k-90-group1554.Contig1 | | | | | | | | | |  |  |  |  |
| M | 0007409 | axonogenesis | EN-124k-90-group1585.Contig2 | | | | | | | | | |  |  |  |  |
| M | 0007409 | axonogenesis | EN-124k-90-group1642.Contig5 | | | | | | | | | |  |  |  |  |
| M | 0007409 | axonogenesis | EN-124k-90-group1654.Contig1 | | | | | | | | | |  |  |  |  |
| M | 0007409 | axonogenesis | EN-124k-90-group1654.Contig2 | | | | | | | | | |  |  |  |  |
| M | 0007409 | axonogenesis | EN-124k-90-group1699.Contig1 | | | | | | | | | |  |  |  |  |
| M | 0007409 | axonogenesis | EN-124k-90-group1699.Contig3 | | | | | | | | | |  |  |  |  |
| M | 0007409 | axonogenesis | EN-124k-90-group1726.Contig2 | | | | | | | | | |  |  |  |  |
| M | 0007409 | axonogenesis | EN-124k-90-group1728.Contig2 | | | | | | | | | |  |  |  |  |
| M | 0007409 | axonogenesis | EN-124k-90-group1735.Contig4 | | | | | | | | | |  |  |  |  |
| M | 0007409 | axonogenesis | EN-124k-90-group1735.Contig6 | | | | | | | | | |  |  |  |  |
| M | 0007409 | axonogenesis | EN-124k-90-group1752.Contig1 | | | | | | | | | |  |  |  |  |
| M | 0007409 | axonogenesis | EN-124k-90-group1752.Contig2 | | | | | | | | | |  |  |  |  |
| M | 0007409 | axonogenesis | EN-124k-90-group1752.Contig3 | | | | | | | | | |  |  |  |  |
| M | 0007409 | axonogenesis | EN-124k-90-group1752.Contig4 | | | | | | | | | |  |  |  |  |
| M | 0007409 | axonogenesis | EN-124k-90-group1752.Contig5 | | | | | | | | | |  |  |  |  |
| M | 0007409 | axonogenesis | EN-124k-90-group1752.Contig6 | | | | | | | | | |  |  |  |  |
| M | 0007409 | axonogenesis | EN-124k-90-group1757.Contig1 | | | | | | | | | |  |  |  |  |
| M | 0007409 | axonogenesis | EN-124k-90-group1770.Contig2 | | | | | | | | | |  |  |  |  |
| M | 0007409 | axonogenesis | EN-124k-90-group1772.Contig1 | | | | | | | | | |  |  |  |  |
| M | 0007409 | axonogenesis | EN-124k-90-group1781.Contig1 | | | | | | | | | |  |  |  |  |
| M | 0007409 | axonogenesis | EN-124k-90-group1782.Contig1 | | | | | | | | | |  |  |  |  |
| M | 0007409 | axonogenesis | EN-124k-90-group1798.Contig2 | | | | | | | | | |  |  |  |  |
| M | 0007409 | axonogenesis | EN-124k-90-group1798.Contig3 | | | | | | | | | |  |  |  |  |
| M | 0007409 | axonogenesis | EN-124k-90-group1819.Contig1 | | | | | | | | | |  |  |  |  |
| M | 0007409 | axonogenesis | EN-124k-90-group1819.Contig3 | | | | | | | | | |  |  |  |  |
| M | 0007409 | axonogenesis | EN-124k-90-group1834.Contig1 | | | | | | | | | |  |  |  |  |
| M | 0007409 | axonogenesis | EN-124k-90-group1861.Contig1 | | | | | | | | | |  |  |  |  |
| M | 0007409 | axonogenesis | EN-124k-90-group1861.Contig2 | | | | | | | | | |  |  |  |  |
| M | 0007409 | axonogenesis | EN-124k-90-group1867.Contig1 | | | | | | | | | |  |  |  |  |
| M | 0007409 | axonogenesis | EN-124k-90-group1869.Contig3 | | | | | | | | | |  |  |  |  |
| M | 0007409 | axonogenesis | EN-124k-90-group1873.Contig1 | | | | | | | | | |  |  |  |  |
| M | 0007409 | axonogenesis | EN-124k-90-group1907.Contig1 | | | | | | | | | |  |  |  |  |
| M | 0007409 | axonogenesis | EN-124k-90-group1909.Contig1 | | | | | | | | | |  |  |  |  |
| M | 0007409 | axonogenesis | EN-124k-90-group1909.Contig2 | | | | | | | | | |  |  |  |  |
| M | 0007409 | axonogenesis | EN-124k-90-group1915.Contig1 | | | | | | | | | |  |  |  |  |
| M | 0007409 | axonogenesis | EN-124k-90-group1933.Contig1 | | | | | | | | | |  |  |  |  |
| M | 0007409 | axonogenesis | EN-124k-90-group1933.Contig2 | | | | | | | | | |  |  |  |  |
| M | 0007409 | axonogenesis | EN-124k-90-group1938.Contig1 | | | | | | | | | |  |  |  |  |
| M | 0007409 | axonogenesis | EN-124k-90-group1958.Contig1 | | | | | | | | | |  |  |  |  |
| M | 0007409 | axonogenesis | EN-124k-90-group1960.Contig1 | | | | | | | | | |  |  |  |  |
| M | 0007409 | axonogenesis | EN-124k-90-group1980.Contig1 | | | | | | | | | |  |  |  |  |
| A | 0007409 | axonogenesis | EN-124k-90-group1993.Contig1 | | | | | | | | | |  |  |  |  |
| M | 0007409 | axonogenesis | EN-124k-90-group2035.Contig1 | | | | | | | | | |  |  |  |  |
| E | 0007409 | axonogenesis | EN-124k-90-group2041.Contig1 | | | | | | | | | |  |  |  |  |
| M | 0007409 | axonogenesis | EN-124k-90-group2069.Contig3 | | | | | | | | | |  |  |  |  |
| M | 0007409 | axonogenesis | EN-124k-90-group2074.Contig1 | | | | | | | | | |  |  |  |  |
| M | 0007409 | axonogenesis | EN-124k-90-group2075.Contig2 | | | | | | | | | |  |  |  |  |
| M | 0007409 | axonogenesis | EN-124k-90-group2077.Contig2 | | | | | | | | | |  |  |  |  |
| M | 0007409 | axonogenesis | EN-124k-90-group2077.Contig3 | | | | | | | | | |  |  |  |  |
| M | 0007409 | axonogenesis | EN-124k-90-group2081.Contig1 | | | | | | | | | |  |  |  |  |
| M | 0007409 | axonogenesis | EN-124k-90-group2081.Contig3 | | | | | | | | | |  |  |  |  |
| M | 0007409 | axonogenesis | EN-124k-90-group2097.Contig1 | | | | | | | | | |  |  |  |  |
| M | 0007409 | axonogenesis | EN-124k-90-group2099.Contig1 | | | | | | | | | |  |  |  |  |
| M | 0007409 | axonogenesis | EN-124k-90-group2127.Contig1 | | | | | | | | | |  |  |  |  |
| M | 0007409 | axonogenesis | EN-124k-90-group2132.Contig1 | | | | | | | | | |  |  |  |  |
| M | 0007409 | axonogenesis | EN-124k-90-group2145.Contig1 | | | | | | | | | |  |  |  |  |
| M | 0007409 | axonogenesis | EN-124k-90-group2163.Contig1 | | | | | | | | | |  |  |  |  |
| M | 0007409 | axonogenesis | EN-124k-90-group2200.Contig1 | | | | | | | | | |  |  |  |  |
| M | 0007409 | axonogenesis | EN-124k-90-group2221.Contig2 | | | | | | | | | |  |  |  |  |
| M | 0007409 | axonogenesis | EN-124k-90-group2223.Contig1 | | | | | | | | | |  |  |  |  |
| M | 0007409 | axonogenesis | EN-124k-90-group2239.Contig1 | | | | | | | | | |  |  |  |  |
| M | 0007409 | axonogenesis | EN-124k-90-group2264.Contig1 | | | | | | | | | |  |  |  |  |
| M | 0007409 | axonogenesis | EN-124k-90-group2264.Contig2 | | | | | | | | | |  |  |  |  |
| M | 0007409 | axonogenesis | EN-124k-90-group2264.Contig3 | | | | | | | | | |  |  |  |  |
| M | 0007409 | axonogenesis | EN-124k-90-group2268.Contig2 | | | | | | | | | |  |  |  |  |
| M | 0007409 | axonogenesis | EN-124k-90-group2287.Contig2 | | | | | | | | | |  |  |  |  |
| M | 0007409 | axonogenesis | EN-124k-90-group2289.Contig1 | | | | | | | | | |  |  |  |  |
| A | 0007409 | axonogenesis | EN-124k-90-group2305.Contig1 | | | | | | | | | |  |  |  |  |
| M | 0007409 | axonogenesis | EN-124k-90-group2327.Contig2 | | | | | | | | | |  |  |  |  |
| M | 0007409 | axonogenesis | EN-124k-90-group2397.Contig1 | | | | | | | | | |  |  |  |  |
| M | 0007409 | axonogenesis | EN-124k-90-group2397.Contig2 | | | | | | | | | |  |  |  |  |
| M | 0007409 | axonogenesis | EN-124k-90-group2409.Contig1 | | | | | | | | | |  |  |  |  |
| M | 0007409 | axonogenesis | EN-124k-90-group2409.Contig3 | | | | | | | | | |  |  |  |  |
| M | 0007409 | axonogenesis | EN-124k-90-group2409.Contig4 | | | | | | | | | |  |  |  |  |
| M | 0007409 | axonogenesis | EN-124k-90-group2425.Contig1 | | | | | | | | | |  |  |  |  |
| M | 0007409 | axonogenesis | EN-124k-90-group2425.Contig7 | | | | | | | | | |  |  |  |  |
| M | 0007409 | axonogenesis | EN-124k-90-group2425.Contig8 | | | | | | | | | |  |  |  |  |
| M | 0007409 | axonogenesis | EN-124k-90-group2428.Contig1 | | | | | | | | | |  |  |  |  |
| M | 0007409 | axonogenesis | EN-124k-90-group2430.Contig1 | | | | | | | | | |  |  |  |  |
| M | 0007409 | axonogenesis | EN-124k-90-group2430.Contig2 | | | | | | | | | |  |  |  |  |
| M | 0007409 | axonogenesis | EN-124k-90-group2438.Contig1 | | | | | | | | | |  |  |  |  |
| M | 0007409 | axonogenesis | EN-124k-90-group2443.Contig1 | | | | | | | | | |  |  |  |  |
| M | 0007409 | axonogenesis | EN-124k-90-group2449.Contig1 | | | | | | | | | |  |  |  |  |
| M | 0007409 | axonogenesis | EN-124k-90-group2449.Contig2 | | | | | | | | | |  |  |  |  |
| M | 0007409 | axonogenesis | EN-124k-90-group2451.Contig1 | | | | | | | | | |  |  |  |  |
| M | 0007409 | axonogenesis | EN-124k-90-group2455.Contig1 | | | | | | | | | |  |  |  |  |
| M | 0007409 | axonogenesis | EN-124k-90-group2472.Contig2 | | | | | | | | | |  |  |  |  |
| M | 0007409 | axonogenesis | EN-124k-90-group2472.Contig3 | | | | | | | | | |  |  |  |  |
| M | 0007409 | axonogenesis | EN-124k-90-group2489.Contig1 | | | | | | | | | |  |  |  |  |
| M | 0007409 | axonogenesis | EN-124k-90-group2491.Contig1 | | | | | | | | | |  |  |  |  |
| M | 0007409 | axonogenesis | EN-124k-90-group2495.Contig1 | | | | | | | | | |  |  |  |  |
| M | 0007409 | axonogenesis | EN-124k-90-group2495.Contig2 | | | | | | | | | |  |  |  |  |
| M | 0007409 | axonogenesis | EN-124k-90-group2498.Contig5 | | | | | | | | | |  |  |  |  |
| M | 0007409 | axonogenesis | EN-124k-90-group2498.Contig7 | | | | | | | | | |  |  |  |  |
| M | 0007409 | axonogenesis | EN-124k-90-group2509.Contig2 | | | | | | | | | |  |  |  |  |
| M | 0007409 | axonogenesis | EN-124k-90-group2525.Contig1 | | | | | | | | | |  |  |  |  |
| M | 0007409 | axonogenesis | EN-124k-90-group2525.Contig2 | | | | | | | | | |  |  |  |  |
| M | 0007409 | axonogenesis | EN-124k-90-group2525.Contig3 | | | | | | | | | |  |  |  |  |
| M | 0007409 | axonogenesis | EN-124k-90-group2525.Contig4 | | | | | | | | | |  |  |  |  |
| M | 0007409 | axonogenesis | EN-124k-90-group2537.Contig1 | | | | | | | | | |  |  |  |  |
| M | 0007409 | axonogenesis | EN-124k-90-group2582.Contig1 | | | | | | | | | |  |  |  |  |
| M | 0007409 | axonogenesis | EN-124k-90-group2584.Contig2 | | | | | | | | | |  |  |  |  |
| M | 0007409 | axonogenesis | EN-124k-90-group2600.Contig2 | | | | | | | | | |  |  |  |  |
| M | 0007409 | axonogenesis | EN-124k-90-group2606.Contig1 | | | | | | | | | |  |  |  |  |
| M | 0007409 | axonogenesis | EN-124k-90-group2619.Contig1 | | | | | | | | | |  |  |  |  |
| M | 0007409 | axonogenesis | EN-124k-90-group2619.Contig2 | | | | | | | | | |  |  |  |  |
| M | 0007409 | axonogenesis | EN-124k-90-group2651.Contig1 | | | | | | | | | |  |  |  |  |
| M | 0007409 | axonogenesis | EN-124k-90-group2681.Contig1 | | | | | | | | | |  |  |  |  |
| M | 0007409 | axonogenesis | EN-124k-90-group2686.Contig1 | | | | | | | | | |  |  |  |  |
| M | 0007409 | axonogenesis | EN-124k-90-group2806.Contig1 | | | | | | | | | |  |  |  |  |
| M | 0007409 | axonogenesis | EN-124k-90-group2811.Contig1 | | | | | | | | | |  |  |  |  |
| M | 0007409 | axonogenesis | EN-124k-90-group2811.Contig2 | | | | | | | | | |  |  |  |  |
| M | 0007409 | axonogenesis | EN-124k-90-group2830.Contig1 | | | | | | | | | |  |  |  |  |
| M | 0007409 | axonogenesis | EN-124k-90-group2834.Contig2 | | | | | | | | | |  |  |  |  |
| M | 0007409 | axonogenesis | EN-124k-90-group2835.Contig1 | | | | | | | | | |  |  |  |  |
| M | 0007409 | axonogenesis | EN-124k-90-group2835.Contig2 | | | | | | | | | |  |  |  |  |
| M | 0007409 | axonogenesis | EN-124k-90-group2841.Contig1 | | | | | | | | | |  |  |  |  |
| M | 0007409 | axonogenesis | EN-124k-90-group2846.Contig1 | | | | | | | | | |  |  |  |  |
| M | 0007409 | axonogenesis | EN-124k-90-group2850.Contig2 | | | | | | | | | |  |  |  |  |
| M | 0007409 | axonogenesis | EN-124k-90-group2903.Contig1 | | | | | | | | | |  |  |  |  |
| M | 0007409 | axonogenesis | EN-124k-90-group2917.Contig1 | | | | | | | | | |  |  |  |  |
| M | 0007409 | axonogenesis | EN-124k-90-group2917.Contig2 | | | | | | | | | |  |  |  |  |
| M | 0007409 | axonogenesis | EN-124k-90-group2917.Contig3 | | | | | | | | | |  |  |  |  |
| M | 0007409 | axonogenesis | EN-124k-90-group2917.Contig4 | | | | | | | | | |  |  |  |  |
| M | 0007409 | axonogenesis | EN-124k-90-group2930.Contig1 | | | | | | | | | |  |  |  |  |
| M | 0007409 | axonogenesis | EN-124k-90-group2940.Contig2 | | | | | | | | | |  |  |  |  |
| M | 0007409 | axonogenesis | EN-124k-90-group3018.Contig1 | | | | | | | | | |  |  |  |  |
| M | 0007409 | axonogenesis | EN-124k-90-group3018.Contig2 | | | | | | | | | |  |  |  |  |
| M | 0007409 | axonogenesis | EN-124k-90-group3033.Contig1 | | | | | | | | | |  |  |  |  |
| M | 0007409 | axonogenesis | EN-124k-90-group3037.Contig1 | | | | | | | | | |  |  |  |  |
| M | 0007409 | axonogenesis | EN-124k-90-group3037.Contig2 | | | | | | | | | |  |  |  |  |
| M | 0007409 | axonogenesis | EN-124k-90-group3067.Contig1 | | | | | | | | | |  |  |  |  |
| M | 0007409 | axonogenesis | EN-124k-90-group3082.Contig2 | | | | | | | | | |  |  |  |  |
| M | 0007409 | axonogenesis | EN-124k-90-group3092.Contig2 | | | | | | | | | |  |  |  |  |
| M | 0007409 | axonogenesis | EN-124k-90-group3114.Contig1 | | | | | | | | | |  |  |  |  |
| M | 0007409 | axonogenesis | EN-124k-90-group3114.Contig3 | | | | | | | | | |  |  |  |  |
| M | 0007409 | axonogenesis | EN-124k-90-group3157.Contig1 | | | | | | | | | |  |  |  |  |
| M | 0007409 | axonogenesis | EN-124k-90-group3157.Contig5 | | | | | | | | | |  |  |  |  |
| M | 0007409 | axonogenesis | EN-124k-90-group3158.Contig2 | | | | | | | | | |  |  |  |  |
| M | 0007409 | axonogenesis | EN-124k-90-group3202.Contig1 | | | | | | | | | |  |  |  |  |
| M | 0007409 | axonogenesis | EN-124k-90-group3202.Contig2 | | | | | | | | | |  |  |  |  |
| M | 0007409 | axonogenesis | EN-124k-90-group3202.Contig3 | | | | | | | | | |  |  |  |  |
| M | 0007409 | axonogenesis | EN-124k-90-group3222.Contig1 | | | | | | | | | |  |  |  |  |
| M | 0007409 | axonogenesis | EN-124k-90-group3223.Contig2 | | | | | | | | | |  |  |  |  |
| M | 0007409 | axonogenesis | EN-124k-90-group3238.Contig1 | | | | | | | | | |  |  |  |  |
| E | 0007409 | axonogenesis | EN-124k-90-group3241.Contig1 | | | | | | | | | |  |  |  |  |
| M | 0007409 | axonogenesis | EN-124k-90-group3242.Contig1 | | | | | | | | | |  |  |  |  |
| M | 0007409 | axonogenesis | EN-124k-90-group3245.Contig2 | | | | | | | | | |  |  |  |  |
| M | 0007409 | axonogenesis | EN-124k-90-group3257.Contig1 | | | | | | | | | |  |  |  |  |
| M | 0007409 | axonogenesis | EN-124k-90-group3292.Contig1 | | | | | | | | | |  |  |  |  |
| M | 0007409 | axonogenesis | EN-124k-90-group3299.Contig1 | | | | | | | | | |  |  |  |  |
| M | 0007409 | axonogenesis | EN-124k-90-group3313.Contig1 | | | | | | | | | |  |  |  |  |
| M | 0007409 | axonogenesis | EN-124k-90-group3313.Contig2 | | | | | | | | | |  |  |  |  |
| M | 0007409 | axonogenesis | EN-124k-90-group3313.Contig3 | | | | | | | | | |  |  |  |  |
| M | 0007409 | axonogenesis | EN-124k-90-group3315.Contig2 | | | | | | | | | |  |  |  |  |
| M | 0007409 | axonogenesis | EN-124k-90-group3315.Contig5 | | | | | | | | | |  |  |  |  |
| M | 0007409 | axonogenesis | EN-124k-90-group3315.Contig6 | | | | | | | | | |  |  |  |  |
| M | 0007409 | axonogenesis | EN-124k-90-group3316.Contig1 | | | | | | | | | |  |  |  |  |
| M | 0007409 | axonogenesis | EN-124k-90-group3343.Contig1 | | | | | | | | | |  |  |  |  |
| E | 0007409 | axonogenesis | EN-124k-90-group3343.Contig2 | | | | | | | | | |  |  |  |  |
| M | 0007409 | axonogenesis | EN-124k-90-group3357.Contig1 | | | | | | | | | |  |  |  |  |
| M | 0007409 | axonogenesis | EN-124k-90-group3365.Contig1 | | | | | | | | | |  |  |  |  |
| M | 0007409 | axonogenesis | EN-124k-90-group3394.Contig1 | | | | | | | | | |  |  |  |  |
| M | 0007409 | axonogenesis | EN-124k-90-group3394.Contig2 | | | | | | | | | |  |  |  |  |
| M | 0007409 | axonogenesis | EN-124k-90-group3394.Contig3 | | | | | | | | | |  |  |  |  |
| M | 0007409 | axonogenesis | EN-124k-90-group3394.Contig4 | | | | | | | | | |  |  |  |  |
| M | 0007409 | axonogenesis | EN-124k-90-group3398.Contig1 | | | | | | | | | |  |  |  |  |
| M | 0007409 | axonogenesis | EN-124k-90-group3408.Contig1 | | | | | | | | | |  |  |  |  |
| M | 0007409 | axonogenesis | EN-124k-90-group3408.Contig2 | | | | | | | | | |  |  |  |  |
| M | 0007409 | axonogenesis | EN-124k-90-group3477.Contig1 | | | | | | | | | |  |  |  |  |
| M | 0007409 | axonogenesis | EN-124k-90-group3479.Contig1 | | | | | | | | | |  |  |  |  |
| M | 0007409 | axonogenesis | EN-124k-90-group3509.Contig1 | | | | | | | | | |  |  |  |  |
| M | 0007409 | axonogenesis | EN-124k-90-group3520.Contig1 | | | | | | | | | |  |  |  |  |
| M | 0007409 | axonogenesis | EN-124k-90-group3522.Contig1 | | | | | | | | | |  |  |  |  |
| M | 0007409 | axonogenesis | EN-124k-90-group3537.Contig1 | | | | | | | | | |  |  |  |  |
| M | 0007409 | axonogenesis | EN-124k-90-group3577.Contig1 | | | | | | | | | |  |  |  |  |
| M | 0007409 | axonogenesis | EN-124k-90-group3577.Contig2 | | | | | | | | | |  |  |  |  |
| M | 0007409 | axonogenesis | EN-124k-90-group3584.Contig1 | | | | | | | | | |  |  |  |  |
| M | 0007409 | axonogenesis | EN-124k-90-group3586.Contig1 | | | | | | | | | |  |  |  |  |
| M | 0007409 | axonogenesis | EN-124k-90-group3590.Contig1 | | | | | | | | | |  |  |  |  |
| M | 0007409 | axonogenesis | EN-124k-90-group3594.Contig1 | | | | | | | | | |  |  |  |  |
| M | 0007409 | axonogenesis | EN-124k-90-group3594.Contig4 | | | | | | | | | |  |  |  |  |
| M | 0007409 | axonogenesis | EN-124k-90-group3606.Contig2 | | | | | | | | | |  |  |  |  |
| E | 0007409 | axonogenesis | EN-124k-90-group3611.Contig1 | | | | | | | | | |  |  |  |  |
| M | 0007409 | axonogenesis | EN-124k-90-group3660.Contig1 | | | | | | | | | |  |  |  |  |
| M | 0007409 | axonogenesis | EN-124k-90-group3670.Contig1 | | | | | | | | | |  |  |  |  |
| M | 0007409 | axonogenesis | EN-124k-90-group3700.Contig1 | | | | | | | | | |  |  |  |  |
| E | 0007409 | axonogenesis | EN-124k-90-group3702.Contig1 | | | | | | | | | |  |  |  |  |
| M | 0007409 | axonogenesis | EN-124k-90-group3713.Contig1 | | | | | | | | | |  |  |  |  |
| M | 0007409 | axonogenesis | EN-124k-90-group3753.Contig1 | | | | | | | | | |  |  |  |  |
| M | 0007409 | axonogenesis | EN-124k-90-group3753.Contig2 | | | | | | | | | |  |  |  |  |
| M | 0007409 | axonogenesis | EN-124k-90-group3810.Contig1 | | | | | | | | | |  |  |  |  |
| M | 0007409 | axonogenesis | EN-124k-90-group3816.Contig1 | | | | | | | | | |  |  |  |  |
| M | 0007409 | axonogenesis | EN-124k-90-group3830.Contig1 | | | | | | | | | |  |  |  |  |
| M | 0007409 | axonogenesis | EN-124k-90-group3849.Contig3 | | | | | | | | | |  |  |  |  |
| M | 0007409 | axonogenesis | EN-124k-90-group3872.Contig3 | | | | | | | | | |  |  |  |  |
| M | 0007409 | axonogenesis | EN-124k-90-group3872.Contig4 | | | | | | | | | |  |  |  |  |
| M | 0007409 | axonogenesis | EN-124k-90-group3876.Contig1 | | | | | | | | | |  |  |  |  |
| M | 0007409 | axonogenesis | EN-124k-90-group3882.Contig1 | | | | | | | | | |  |  |  |  |
| M | 0007409 | axonogenesis | EN-124k-90-group3882.Contig2 | | | | | | | | | |  |  |  |  |
| M | 0007409 | axonogenesis | EN-124k-90-group3883.Contig1 | | | | | | | | | |  |  |  |  |
| M | 0007409 | axonogenesis | EN-124k-90-group3883.Contig2 | | | | | | | | | |  |  |  |  |
| M | 0007409 | axonogenesis | EN-124k-90-group3883.Contig3 | | | | | | | | | |  |  |  |  |
| M | 0007409 | axonogenesis | EN-124k-90-group3883.Contig4 | | | | | | | | | |  |  |  |  |
| M | 0007409 | axonogenesis | EN-124k-90-group3892.Contig1 | | | | | | | | | |  |  |  |  |
| M | 0007409 | axonogenesis | EN-124k-90-group3911.Contig2 | | | | | | | | | |  |  |  |  |
| M | 0007409 | axonogenesis | EN-124k-90-group3916.Contig1 | | | | | | | | | |  |  |  |  |
| M | 0007409 | axonogenesis | EN-124k-90-group3933.Contig1 | | | | | | | | | |  |  |  |  |
| M | 0007409 | axonogenesis | EN-124k-90-group3947.Contig1 | | | | | | | | | |  |  |  |  |
| M | 0007409 | axonogenesis | EN-124k-90-group3947.Contig2 | | | | | | | | | |  |  |  |  |
| M | 0007409 | axonogenesis | EN-124k-90-group3966.Contig1 | | | | | | | | | |  |  |  |  |
| M | 0007409 | axonogenesis | EN-124k-90-group3966.Contig2 | | | | | | | | | |  |  |  |  |
| M | 0007409 | axonogenesis | EN-124k-90-group3991.Contig1 | | | | | | | | | |  |  |  |  |
| M | 0007409 | axonogenesis | EN-124k-90-group3997.Contig2 | | | | | | | | | |  |  |  |  |
| M | 0007409 | axonogenesis | EN-124k-90-group4009.Contig1 | | | | | | | | | |  |  |  |  |
| E | 0007409 | axonogenesis | EN-124k-90-group4039.Contig1 | | | | | | | | | |  |  |  |  |
| M | 0007409 | axonogenesis | EN-124k-90-group4045.Contig1 | | | | | | | | | |  |  |  |  |
| M | 0007409 | axonogenesis | EN-124k-90-group4046.Contig1 | | | | | | | | | |  |  |  |  |
| M | 0007409 | axonogenesis | EN-124k-90-group4049.Contig1 | | | | | | | | | |  |  |  |  |
| M | 0007409 | axonogenesis | EN-124k-90-group4086.Contig1 | | | | | | | | | |  |  |  |  |
| M | 0007409 | axonogenesis | EN-124k-90-group4090.Contig1 | | | | | | | | | |  |  |  |  |
| M | 0007409 | axonogenesis | EN-124k-90-group4106.Contig1 | | | | | | | | | |  |  |  |  |
| M | 0007409 | axonogenesis | EN-124k-90-group4115.Contig2 | | | | | | | | | |  |  |  |  |
| M | 0007409 | axonogenesis | EN-124k-90-group4128.Contig1 | | | | | | | | | |  |  |  |  |
| M | 0007409 | axonogenesis | EN-124k-90-group4154.Contig1 | | | | | | | | | |  |  |  |  |
| M | 0007409 | axonogenesis | EN-124k-90-group4168.Contig1 | | | | | | | | | |  |  |  |  |
| M | 0007409 | axonogenesis | EN-124k-90-group4168.Contig2 | | | | | | | | | |  |  |  |  |
| M | 0007409 | axonogenesis | EN-124k-90-group4168.Contig3 | | | | | | | | | |  |  |  |  |
| M | 0007409 | axonogenesis | EN-124k-90-group4183.Contig1 | | | | | | | | | |  |  |  |  |
| M | 0007409 | axonogenesis | EN-124k-90-group4216.Contig1 | | | | | | | | | |  |  |  |  |
| M | 0007409 | axonogenesis | EN-124k-90-group4225.Contig2 | | | | | | | | | |  |  |  |  |
| M | 0007409 | axonogenesis | EN-124k-90-group4290.Contig3 | | | | | | | | | |  |  |  |  |
| M | 0007409 | axonogenesis | EN-124k-90-group4339.Contig1 | | | | | | | | | |  |  |  |  |
| M | 0007409 | axonogenesis | EN-124k-90-group4349.Contig1 | | | | | | | | | |  |  |  |  |
| M | 0007409 | axonogenesis | EN-124k-90-group4349.Contig3 | | | | | | | | | |  |  |  |  |
| M | 0007409 | axonogenesis | EN-124k-90-group4349.Contig4 | | | | | | | | | |  |  |  |  |
| M | 0007409 | axonogenesis | EN-124k-90-group4350.Contig2 | | | | | | | | | |  |  |  |  |
| M | 0007409 | axonogenesis | EN-124k-90-group4372.Contig1 | | | | | | | | | |  |  |  |  |
| M | 0007409 | axonogenesis | EN-124k-90-group4410.Contig1 | | | | | | | | | |  |  |  |  |
| M | 0007409 | axonogenesis | EN-124k-90-group4432.Contig4 | | | | | | | | | |  |  |  |  |
| M | 0007409 | axonogenesis | EN-124k-90-group4440.Contig1 | | | | | | | | | |  |  |  |  |
| M | 0007409 | axonogenesis | EN-124k-90-group4440.Contig2 | | | | | | | | | |  |  |  |  |
| M | 0007409 | axonogenesis | EN-124k-90-group4440.Contig3 | | | | | | | | | |  |  |  |  |
| M | 0007409 | axonogenesis | EN-124k-90-group4440.Contig4 | | | | | | | | | |  |  |  |  |
| M | 0007409 | axonogenesis | EN-124k-90-group4529.Contig1 | | | | | | | | | |  |  |  |  |
| M | 0007409 | axonogenesis | EN-124k-90-group4558.Contig2 | | | | | | | | | |  |  |  |  |
| M | 0007409 | axonogenesis | EN-124k-90-group4560.Contig1 | | | | | | | | | |  |  |  |  |
| M | 0007409 | axonogenesis | EN-124k-90-group4562.Contig1 | | | | | | | | | |  |  |  |  |
| M | 0007409 | axonogenesis | EN-124k-90-group4599.Contig1 | | | | | | | | | |  |  |  |  |
| M | 0007409 | axonogenesis | EN-124k-90-group4600.Contig1 | | | | | | | | | |  |  |  |  |
| M | 0007409 | axonogenesis | EN-124k-90-group4616.Contig1 | | | | | | | | | |  |  |  |  |
| M | 0007409 | axonogenesis | EN-124k-90-group4621.Contig2 | | | | | | | | | |  |  |  |  |
| M | 0007409 | axonogenesis | EN-124k-90-group4668.Contig1 | | | | | | | | | |  |  |  |  |
| M | 0007409 | axonogenesis | EN-124k-90-group4701.Contig1 | | | | | | | | | |  |  |  |  |
| M | 0007409 | axonogenesis | EN-124k-90-group4701.Contig2 | | | | | | | | | |  |  |  |  |
| M | 0007409 | axonogenesis | EN-124k-90-group4701.Contig3 | | | | | | | | | |  |  |  |  |
| M | 0007409 | axonogenesis | EN-124k-90-group4716.Contig1 | | | | | | | | | |  |  |  |  |
| M | 0007409 | axonogenesis | EN-124k-90-group4753.Contig1 | | | | | | | | | |  |  |  |  |
| M | 0007409 | axonogenesis | EN-124k-90-group4759.Contig2 | | | | | | | | | |  |  |  |  |
| M | 0007409 | axonogenesis | EN-124k-90-group4759.Contig3 | | | | | | | | | |  |  |  |  |
| M | 0007409 | axonogenesis | EN-124k-90-group4763.Contig1 | | | | | | | | | |  |  |  |  |
| M | 0007409 | axonogenesis | EN-124k-90-group4781.Contig1 | | | | | | | | | |  |  |  |  |
| M | 0007409 | axonogenesis | EN-124k-90-group4781.Contig2 | | | | | | | | | |  |  |  |  |
| M | 0007409 | axonogenesis | EN-124k-90-group4784.Contig1 | | | | | | | | | |  |  |  |  |
| M | 0007409 | axonogenesis | EN-124k-90-group4788.Contig1 | | | | | | | | | |  |  |  |  |
| M | 0007409 | axonogenesis | EN-124k-90-group4793.Contig2 | | | | | | | | | |  |  |  |  |
| M | 0007409 | axonogenesis | EN-124k-90-group4803.Contig1 | | | | | | | | | |  |  |  |  |
| M | 0007409 | axonogenesis | EN-124k-90-group4803.Contig2 | | | | | | | | | |  |  |  |  |
| M | 0007409 | axonogenesis | EN-124k-90-group4808.Contig1 | | | | | | | | | |  |  |  |  |
| M | 0007409 | axonogenesis | EN-124k-90-group4808.Contig2 | | | | | | | | | |  |  |  |  |
| M | 0007409 | axonogenesis | EN-124k-90-group4824.Contig3 | | | | | | | | | |  |  |  |  |
| M | 0007409 | axonogenesis | EN-124k-90-group4824.Contig4 | | | | | | | | | |  |  |  |  |
| M | 0007409 | axonogenesis | EN-124k-90-group4827.Contig2 | | | | | | | | | |  |  |  |  |
| M | 0007409 | axonogenesis | EN-124k-90-group4827.Contig3 | | | | | | | | | |  |  |  |  |
| M | 0007409 | axonogenesis | EN-124k-90-group4829.Contig3 | | | | | | | | | |  |  |  |  |
| E | 0007409 | axonogenesis | EN-124k-90-group4884.Contig1 | | | | | | | | | |  |  |  |  |
| M | 0007409 | axonogenesis | EN-124k-90-group4890.Contig1 | | | | | | | | | |  |  |  |  |
| M | 0007409 | axonogenesis | EN-124k-90-group4929.Contig1 | | | | | | | | | |  |  |  |  |
| M | 0007409 | axonogenesis | EN-124k-90-group4929.Contig2 | | | | | | | | | |  |  |  |  |
| A | 0007409 | axonogenesis | EN-124k-90-group4960.Contig1 | | | | | | | | | |  |  |  |  |
| M | 0007409 | axonogenesis | EN-124k-90-group4989.Contig2 | | | | | | | | | |  |  |  |  |
| M | 0007409 | axonogenesis | EN-124k-90-group5022.Contig2 | | | | | | | | | |  |  |  |  |
| M | 0007409 | axonogenesis | EN-124k-90-group5036.Contig2 | | | | | | | | | |  |  |  |  |
| M | 0007409 | axonogenesis | EN-124k-90-group5040.Contig1 | | | | | | | | | |  |  |  |  |
| M | 0007409 | axonogenesis | EN-124k-90-group5040.Contig2 | | | | | | | | | |  |  |  |  |
| M | 0007409 | axonogenesis | EN-124k-90-group5051.Contig2 | | | | | | | | | |  |  |  |  |
| M | 0007409 | axonogenesis | EN-124k-90-group5054.Contig1 | | | | | | | | | |  |  |  |  |
| A | 0007409 | axonogenesis | EN-124k-90-group5054.Contig2 | | | | | | | | | |  |  |  |  |
| M | 0007409 | axonogenesis | EN-124k-90-group5054.Contig3 | | | | | | | | | |  |  |  |  |
| M | 0007409 | axonogenesis | EN-124k-90-group5054.Contig4 | | | | | | | | | |  |  |  |  |
| M | 0007409 | axonogenesis | EN-124k-90-group5055.Contig1 | | | | | | | | | |  |  |  |  |
| M | 0007409 | axonogenesis | EN-124k-90-group5055.Contig3 | | | | | | | | | |  |  |  |  |
| M | 0007409 | axonogenesis | EN-124k-90-group5063.Contig1 | | | | | | | | | |  |  |  |  |
| M | 0007409 | axonogenesis | EN-124k-90-group5071.Contig3 | | | | | | | | | |  |  |  |  |
| M | 0007409 | axonogenesis | EN-124k-90-group5093.Contig2 | | | | | | | | | |  |  |  |  |
| M | 0007409 | axonogenesis | EN-124k-90-group5134.Contig1 | | | | | | | | | |  |  |  |  |
| M | 0007409 | axonogenesis | EN-124k-90-group5148.Contig1 | | | | | | | | | |  |  |  |  |
| M | 0007409 | axonogenesis | EN-124k-90-group5148.Contig3 | | | | | | | | | |  |  |  |  |
| M | 0007409 | axonogenesis | EN-124k-90-group5170.Contig1 | | | | | | | | | |  |  |  |  |
| M | 0007409 | axonogenesis | EN-124k-90-group5170.Contig2 | | | | | | | | | |  |  |  |  |
| M | 0007409 | axonogenesis | EN-124k-90-group5170.Contig3 | | | | | | | | | |  |  |  |  |
| M | 0007409 | axonogenesis | EN-124k-90-group5172.Contig1 | | | | | | | | | |  |  |  |  |
| M | 0007409 | axonogenesis | EN-124k-90-group5173.Contig1 | | | | | | | | | |  |  |  |  |
| M | 0007409 | axonogenesis | EN-124k-90-group5184.Contig1 | | | | | | | | | |  |  |  |  |
| M | 0007409 | axonogenesis | EN-124k-90-group5215.Contig3 | | | | | | | | | |  |  |  |  |
| M | 0007409 | axonogenesis | EN-124k-90-group5252.Contig1 | | | | | | | | | |  |  |  |  |
| M | 0007409 | axonogenesis | EN-124k-90-group5273.Contig2 | | | | | | | | | |  |  |  |  |
| M | 0007409 | axonogenesis | EN-124k-90-group5273.Contig3 | | | | | | | | | |  |  |  |  |
| M | 0007409 | axonogenesis | EN-124k-90-group5282.Contig1 | | | | | | | | | |  |  |  |  |
| M | 0007409 | axonogenesis | EN-124k-90-group5293.Contig1 | | | | | | | | | |  |  |  |  |
| M | 0007409 | axonogenesis | EN-124k-90-group5298.Contig1 | | | | | | | | | |  |  |  |  |
| M | 0007409 | axonogenesis | EN-124k-90-group5300.Contig5 | | | | | | | | | |  |  |  |  |
| M | 0007409 | axonogenesis | EN-124k-90-group5329.Contig1 | | | | | | | | | |  |  |  |  |
| M | 0007409 | axonogenesis | EN-124k-90-group5341.Contig4 | | | | | | | | | |  |  |  |  |
| M | 0007409 | axonogenesis | EN-124k-90-group5359.Contig3 | | | | | | | | | |  |  |  |  |
| M | 0007409 | axonogenesis | EN-124k-90-group5365.Contig1 | | | | | | | | | |  |  |  |  |
| M | 0007409 | axonogenesis | EN-124k-90-group5380.Contig1 | | | | | | | | | |  |  |  |  |
| M | 0007409 | axonogenesis | EN-124k-90-group5383.Contig1 | | | | | | | | | |  |  |  |  |
| M | 0007409 | axonogenesis | EN-124k-90-group5398.Contig1 | | | | | | | | | |  |  |  |  |
| M | 0007409 | axonogenesis | EN-124k-90-group5411.Contig1 | | | | | | | | | |  |  |  |  |
| M | 0007409 | axonogenesis | EN-124k-90-group5459.Contig2 | | | | | | | | | |  |  |  |  |
| M | 0007409 | axonogenesis | EN-124k-90-group5471.Contig1 | | | | | | | | | |  |  |  |  |
| M | 0007409 | axonogenesis | EN-124k-90-group5471.Contig3 | | | | | | | | | |  |  |  |  |
| M | 0007409 | axonogenesis | EN-124k-90-group5479.Contig1 | | | | | | | | | |  |  |  |  |
| M | 0007409 | axonogenesis | EN-124k-90-group5491.Contig1 | | | | | | | | | |  |  |  |  |
| M | 0007409 | axonogenesis | EN-124k-90-group5491.Contig2 | | | | | | | | | |  |  |  |  |
| M | 0007409 | axonogenesis | EN-124k-90-group5527.Contig2 | | | | | | | | | |  |  |  |  |
| M | 0007409 | axonogenesis | EN-124k-90-group5541.Contig1 | | | | | | | | | |  |  |  |  |
| M | 0007409 | axonogenesis | EN-124k-90-group5542.Contig1 | | | | | | | | | |  |  |  |  |
| A | 0007409 | axonogenesis | EN-124k-90-group5543.Contig1 | | | | | | | | | |  |  |  |  |
| M | 0007409 | axonogenesis | EN-124k-90-group5563.Contig1 | | | | | | | | | |  |  |  |  |
| M | 0007409 | axonogenesis | EN-124k-90-group5567.Contig2 | | | | | | | | | |  |  |  |  |
| M | 0007409 | axonogenesis | EN-124k-90-group5567.Contig3 | | | | | | | | | |  |  |  |  |
| M | 0007409 | axonogenesis | EN-124k-90-group5597.Contig2 | | | | | | | | | |  |  |  |  |
| M | 0007409 | axonogenesis | EN-124k-90-group5607.Contig1 | | | | | | | | | |  |  |  |  |
| M | 0007409 | axonogenesis | EN-124k-90-group5616.Contig1 | | | | | | | | | |  |  |  |  |
| M | 0007409 | axonogenesis | EN-124k-90-group5633.Contig1 | | | | | | | | | |  |  |  |  |
| M | 0007409 | axonogenesis | EN-124k-90-group5633.Contig2 | | | | | | | | | |  |  |  |  |
| M | 0007409 | axonogenesis | EN-124k-90-group5709.Contig1 | | | | | | | | | |  |  |  |  |
| M | 0007409 | axonogenesis | EN-124k-90-group5710.Contig2 | | | | | | | | | |  |  |  |  |
| M | 0007409 | axonogenesis | EN-124k-90-group5717.Contig2 | | | | | | | | | |  |  |  |  |
| M | 0007409 | axonogenesis | EN-124k-90-group5729.Contig1 | | | | | | | | | |  |  |  |  |
| M | 0007409 | axonogenesis | EN-124k-90-group5729.Contig2 | | | | | | | | | |  |  |  |  |
| M | 0007409 | axonogenesis | EN-124k-90-group5746.Contig1 | | | | | | | | | |  |  |  |  |
| M | 0007409 | axonogenesis | EN-124k-90-group5910.Contig1 | | | | | | | | | |  |  |  |  |
| M | 0007409 | axonogenesis | EN-124k-90-group5967.Contig1 | | | | | | | | | |  |  |  |  |
| M | 0007409 | axonogenesis | EN-124k-90-group5968.Contig1 | | | | | | | | | |  |  |  |  |
| M | 0007409 | axonogenesis | EN-124k-90-group5996.Contig1 | | | | | | | | | |  |  |  |  |
| M | 0007409 | axonogenesis | EN-124k-90-group5996.Contig2 | | | | | | | | | |  |  |  |  |
| M | 0007409 | axonogenesis | EN-124k-90-group6033.Contig1 | | | | | | | | | |  |  |  |  |
| M | 0007409 | axonogenesis | EN-124k-90-group6033.Contig2 | | | | | | | | | |  |  |  |  |
| M | 0007409 | axonogenesis | EN-124k-90-group6033.Contig3 | | | | | | | | | |  |  |  |  |
| M | 0007409 | axonogenesis | EN-124k-90-group6039.Contig2 | | | | | | | | | |  |  |  |  |
| M | 0007409 | axonogenesis | EN-124k-90-group6039.Contig3 | | | | | | | | | |  |  |  |  |
| M | 0007409 | axonogenesis | EN-124k-90-group6045.Contig1 | | | | | | | | | |  |  |  |  |
| M | 0007409 | axonogenesis | EN-124k-90-group6109.Contig1 | | | | | | | | | |  |  |  |  |
| M | 0007409 | axonogenesis | EN-124k-90-group6112.Contig1 | | | | | | | | | |  |  |  |  |
| M | 0007409 | axonogenesis | EN-124k-90-group6157.Contig1 | | | | | | | | | |  |  |  |  |
| M | 0007409 | axonogenesis | EN-124k-90-group6172.Contig1 | | | | | | | | | |  |  |  |  |
| M | 0007409 | axonogenesis | EN-124k-90-group6172.Contig2 | | | | | | | | | |  |  |  |  |
| M | 0007409 | axonogenesis | EN-124k-90-group6172.Contig3 | | | | | | | | | |  |  |  |  |
| M | 0007409 | axonogenesis | EN-124k-90-group6183.Contig1 | | | | | | | | | |  |  |  |  |
| M | 0007409 | axonogenesis | EN-124k-90-group6195.Contig1 | | | | | | | | | |  |  |  |  |
| M | 0007409 | axonogenesis | EN-124k-90-group6195.Contig2 | | | | | | | | | |  |  |  |  |
| M | 0007409 | axonogenesis | EN-124k-90-group6203.Contig1 | | | | | | | | | |  |  |  |  |
| M | 0007409 | axonogenesis | EN-124k-90-group6204.Contig1 | | | | | | | | | |  |  |  |  |
| M | 0007409 | axonogenesis | EN-124k-90-group6204.Contig2 | | | | | | | | | |  |  |  |  |
| E | 0007409 | axonogenesis | EN-124k-90-group6238.Contig1 | | | | | | | | | |  |  |  |  |
| M | 0007409 | axonogenesis | EN-124k-90-group6259.Contig1 | | | | | | | | | |  |  |  |  |
| M | 0007409 | axonogenesis | EN-124k-90-group6259.Contig2 | | | | | | | | | |  |  |  |  |
| M | 0007409 | axonogenesis | EN-124k-90-group6291.Contig2 | | | | | | | | | |  |  |  |  |
| E | 0007409 | axonogenesis | EN-124k-90-group6326.Contig1 | | | | | | | | | |  |  |  |  |
| M | 0007409 | axonogenesis | EN-124k-90-group6358.Contig2 | | | | | | | | | |  |  |  |  |
| M | 0007409 | axonogenesis | EN-124k-90-group6360.Contig1 | | | | | | | | | |  |  |  |  |
| M | 0007409 | axonogenesis | EN-124k-90-group6400.Contig1 | | | | | | | | | |  |  |  |  |
| M | 0007409 | axonogenesis | EN-124k-90-group6457.Contig1 | | | | | | | | | |  |  |  |  |
| M | 0007409 | axonogenesis | EN-124k-90-group6591.Contig1 | | | | | | | | | |  |  |  |  |
| M | 0007409 | axonogenesis | EN-124k-90-group6614.Contig1 | | | | | | | | | |  |  |  |  |
| M | 0007409 | axonogenesis | EN-124k-90-group6620.Contig1 | | | | | | | | | |  |  |  |  |
| M | 0007409 | axonogenesis | EN-124k-90-group6644.Contig1 | | | | | | | | | |  |  |  |  |
| M | 0007409 | axonogenesis | EN-124k-90-group6647.Contig1 | | | | | | | | | |  |  |  |  |
| M | 0007409 | axonogenesis | EN-124k-90-group6649.Contig2 | | | | | | | | | |  |  |  |  |
| M | 0007409 | axonogenesis | EN-124k-90-group6649.Contig3 | | | | | | | | | |  |  |  |  |
| M | 0007409 | axonogenesis | EN-124k-90-group6652.Contig1 | | | | | | | | | |  |  |  |  |
| M | 0007409 | axonogenesis | EN-124k-90-group6666.Contig1 | | | | | | | | | |  |  |  |  |
| M | 0007409 | axonogenesis | EN-124k-90-group6667.Contig1 | | | | | | | | | |  |  |  |  |
| M | 0007409 | axonogenesis | EN-124k-90-group6680.Contig1 | | | | | | | | | |  |  |  |  |
| M | 0007409 | axonogenesis | EN-124k-90-group6687.Contig2 | | | | | | | | | |  |  |  |  |
| M | 0007409 | axonogenesis | EN-124k-90-group6694.Contig1 | | | | | | | | | |  |  |  |  |
| E | 0007409 | axonogenesis | EN-124k-90-group6714.Contig1 | | | | | | | | | |  |  |  |  |
| M | 0007409 | axonogenesis | EN-124k-90-group6719.Contig2 | | | | | | | | | |  |  |  |  |
| M | 0007409 | axonogenesis | EN-124k-90-group6742.Contig2 | | | | | | | | | |  |  |  |  |
| M | 0007409 | axonogenesis | EN-124k-90-group6809.Contig1 | | | | | | | | | |  |  |  |  |
| M | 0007409 | axonogenesis | EN-124k-90-group6883.Contig2 | | | | | | | | | |  |  |  |  |
| M | 0007409 | axonogenesis | EN-124k-90-group6895.Contig1 | | | | | | | | | |  |  |  |  |
| M | 0007409 | axonogenesis | EN-124k-90-group6904.Contig1 | | | | | | | | | |  |  |  |  |
| M | 0007409 | axonogenesis | EN-124k-90-group6923.Contig1 | | | | | | | | | |  |  |  |  |
| M | 0007409 | axonogenesis | EN-124k-90-group6923.Contig2 | | | | | | | | | |  |  |  |  |
| A | 0007409 | axonogenesis | EN-124k-90-group6966.Contig1 | | | | | | | | | |  |  |  |  |
| M | 0007409 | axonogenesis | EN-124k-90-group6983.Contig2 | | | | | | | | | |  |  |  |  |
| M | 0007409 | axonogenesis | EN-124k-90-group7017.Contig1 | | | | | | | | | |  |  |  |  |
| M | 0007409 | axonogenesis | EN-124k-90-group7018.Contig1 | | | | | | | | | |  |  |  |  |
| M | 0007409 | axonogenesis | EN-124k-90-group7046.Contig1 | | | | | | | | | |  |  |  |  |
| M | 0007409 | axonogenesis | EN-124k-90-group7065.Contig2 | | | | | | | | | |  |  |  |  |
| M | 0007409 | axonogenesis | EN-124k-90-group7102.Contig1 | | | | | | | | | |  |  |  |  |
| M | 0007409 | axonogenesis | EN-124k-90-group7107.Contig2 | | | | | | | | | |  |  |  |  |
| M | 0007409 | axonogenesis | EN-124k-90-group7145.Contig1 | | | | | | | | | |  |  |  |  |
| M | 0007409 | axonogenesis | EN-124k-90-group7149.Contig2 | | | | | | | | | |  |  |  |  |
| M | 0007409 | axonogenesis | EN-124k-90-group7157.Contig1 | | | | | | | | | |  |  |  |  |
| M | 0007409 | axonogenesis | EN-124k-90-group7157.Contig2 | | | | | | | | | |  |  |  |  |
| M | 0007409 | axonogenesis | EN-124k-90-group7165.Contig1 | | | | | | | | | |  |  |  |  |
| M | 0007409 | axonogenesis | EN-124k-90-group7214.Contig1 | | | | | | | | | |  |  |  |  |
| M | 0007409 | axonogenesis | EN-124k-90-group7214.Contig2 | | | | | | | | | |  |  |  |  |
| M | 0007409 | axonogenesis | EN-124k-90-group7231.Contig1 | | | | | | | | | |  |  |  |  |
| M | 0007409 | axonogenesis | EN-124k-90-group7234.Contig1 | | | | | | | | | |  |  |  |  |
| M | 0007409 | axonogenesis | EN-124k-90-group7280.Contig1 | | | | | | | | | |  |  |  |  |
| M | 0007409 | axonogenesis | EN-124k-90-group7281.Contig1 | | | | | | | | | |  |  |  |  |
| M | 0007409 | axonogenesis | EN-124k-90-group7281.Contig2 | | | | | | | | | |  |  |  |  |
| M | 0007409 | axonogenesis | EN-124k-90-group7332.Contig1 | | | | | | | | | |  |  |  |  |
| M | 0007409 | axonogenesis | EN-124k-90-group7343.Contig1 | | | | | | | | | |  |  |  |  |
| M | 0007409 | axonogenesis | EN-124k-90-group7382.Contig1 | | | | | | | | | |  |  |  |  |
| M | 0007409 | axonogenesis | EN-124k-90-group7382.Contig2 | | | | | | | | | |  |  |  |  |
| M | 0007409 | axonogenesis | EN-124k-90-group7440.Contig1 | | | | | | | | | |  |  |  |  |
| M | 0007409 | axonogenesis | EN-124k-90-group7457.Contig1 | | | | | | | | | |  |  |  |  |
| M | 0007409 | axonogenesis | EN-124k-90-group7489.Contig1 | | | | | | | | | |  |  |  |  |
| M | 0007409 | axonogenesis | EN-124k-90-group7514.Contig1 | | | | | | | | | |  |  |  |  |
| M | 0007409 | axonogenesis | EN-124k-90-group7528.Contig1 | | | | | | | | | |  |  |  |  |
| M | 0007409 | axonogenesis | EN-124k-90-group7579.Contig1 | | | | | | | | | |  |  |  |  |
| M | 0007409 | axonogenesis | EN-124k-90-group7612.Contig2 | | | | | | | | | |  |  |  |  |
| A | 0007409 | axonogenesis | EN-124k-90-group7650.Contig1 | | | | | | | | | |  |  |  |  |
| M | 0007409 | axonogenesis | EN-124k-90-group7660.Contig1 | | | | | | | | | |  |  |  |  |
| M | 0007409 | axonogenesis | EN-124k-90-group7666.Contig2 | | | | | | | | | |  |  |  |  |
| M | 0007409 | axonogenesis | EN-124k-90-group7743.Contig1 | | | | | | | | | |  |  |  |  |
| M | 0007409 | axonogenesis | EN-124k-90-group7749.Contig1 | | | | | | | | | |  |  |  |  |
| M | 0007409 | axonogenesis | EN-124k-90-group7770.Contig1 | | | | | | | | | |  |  |  |  |
| M | 0007409 | axonogenesis | EN-124k-90-group7770.Contig2 | | | | | | | | | |  |  |  |  |
| M | 0007409 | axonogenesis | EN-124k-90-group7770.Contig3 | | | | | | | | | |  |  |  |  |
| M | 0007409 | axonogenesis | EN-124k-90-group7785.Contig1 | | | | | | | | | |  |  |  |  |
| M | 0007409 | axonogenesis | EN-124k-90-group7786.Contig1 | | | | | | | | | |  |  |  |  |
| M | 0007409 | axonogenesis | EN-124k-90-group7859.Contig3 | | | | | | | | | |  |  |  |  |
| M | 0007409 | axonogenesis | EN-124k-90-group7866.Contig1 | | | | | | | | | |  |  |  |  |
| M | 0007409 | axonogenesis | EN-124k-90-group7876.Contig1 | | | | | | | | | |  |  |  |  |
| M | 0007409 | axonogenesis | EN-124k-90-group7883.Contig1 | | | | | | | | | |  |  |  |  |
| E | 0007409 | axonogenesis | EN-124k-90-group7888.Contig1 | | | | | | | | | |  |  |  |  |
| M | 0007409 | axonogenesis | EN-124k-90-group7915.Contig1 | | | | | | | | | |  |  |  |  |
| M | 0007409 | axonogenesis | EN-124k-90-group7948.Contig1 | | | | | | | | | |  |  |  |  |
| M | 0007409 | axonogenesis | EN-124k-90-group8079.Contig1 | | | | | | | | | |  |  |  |  |
| M | 0007409 | axonogenesis | EN-124k-90-group8079.Contig2 | | | | | | | | | |  |  |  |  |
| E | 0007409 | axonogenesis | EN-124k-90-group8101.Contig1 | | | | | | | | | |  |  |  |  |
| M | 0007409 | axonogenesis | EN-124k-90-group8103.Contig4 | | | | | | | | | |  |  |  |  |
| M | 0007409 | axonogenesis | EN-124k-90-group8174.Contig1 | | | | | | | | | |  |  |  |  |
| M | 0007409 | axonogenesis | EN-124k-90-group8174.Contig2 | | | | | | | | | |  |  |  |  |
| M | 0007409 | axonogenesis | EN-124k-90-group8193.Contig1 | | | | | | | | | |  |  |  |  |
| M | 0007409 | axonogenesis | EN-124k-90-group8211.Contig1 | | | | | | | | | |  |  |  |  |
| M | 0007409 | axonogenesis | EN-124k-90-group8221.Contig1 | | | | | | | | | |  |  |  |  |
| M | 0007409 | axonogenesis | EN-124k-90-group8245.Contig2 | | | | | | | | | |  |  |  |  |
| M | 0007409 | axonogenesis | EN-124k-90-group8245.Contig3 | | | | | | | | | |  |  |  |  |
| M | 0007409 | axonogenesis | EN-124k-90-group8253.Contig1 | | | | | | | | | |  |  |  |  |
| M | 0007409 | axonogenesis | EN-124k-90-group8297.Contig1 | | | | | | | | | |  |  |  |  |
| M | 0007409 | axonogenesis | EN-124k-90-group8306.Contig1 | | | | | | | | | |  |  |  |  |
| M | 0007409 | axonogenesis | EN-124k-90-group8306.Contig4 | | | | | | | | | |  |  |  |  |
| M | 0007409 | axonogenesis | EN-124k-90-group8325.Contig1 | | | | | | | | | |  |  |  |  |
| M | 0007409 | axonogenesis | EN-124k-90-group8351.Contig2 | | | | | | | | | |  |  |  |  |
| M | 0007409 | axonogenesis | EN-124k-90-group8375.Contig1 | | | | | | | | | |  |  |  |  |
| E | 0007409 | axonogenesis | EN-124k-90-group8412.Contig1 | | | | | | | | | |  |  |  |  |
| M | 0007409 | axonogenesis | EN-124k-90-group8418.Contig1 | | | | | | | | | |  |  |  |  |
| M | 0007409 | axonogenesis | EN-124k-90-group8434.Contig2 | | | | | | | | | |  |  |  |  |
| M | 0007409 | axonogenesis | EN-124k-90-group8440.Contig1 | | | | | | | | | |  |  |  |  |
| M | 0007409 | axonogenesis | EN-124k-90-group8452.Contig1 | | | | | | | | | |  |  |  |  |
| M | 0007409 | axonogenesis | EN-124k-90-group8484.Contig3 | | | | | | | | | |  |  |  |  |
| M | 0007409 | axonogenesis | EN-124k-90-group8492.Contig1 | | | | | | | | | |  |  |  |  |
| E | 0007409 | axonogenesis | EN-124k-90-group8513.Contig1 | | | | | | | | | |  |  |  |  |
| A | 0007409 | axonogenesis | EN-124k-90-group8519.Contig1 | | | | | | | | | |  |  |  |  |
| M | 0007409 | axonogenesis | EN-124k-90-group8568.Contig1 | | | | | | | | | |  |  |  |  |
| M | 0007409 | axonogenesis | EN-124k-90-group8589.Contig2 | | | | | | | | | |  |  |  |  |
| M | 0007409 | axonogenesis | EN-124k-90-group8645.Contig1 | | | | | | | | | |  |  |  |  |
| M | 0007409 | axonogenesis | EN-124k-90-group8685.Contig1 | | | | | | | | | |  |  |  |  |
| M | 0007409 | axonogenesis | EN-124k-90-group8696.Contig1 | | | | | | | | | |  |  |  |  |
| A | 0007409 | axonogenesis | EN-124k-90-group8725.Contig1 | | | | | | | | | |  |  |  |  |
| A | 0007409 | axonogenesis | EN-124k-90-group8766.Contig1 | | | | | | | | | |  |  |  |  |
| M | 0007409 | axonogenesis | EN-124k-90-group8775.Contig1 | | | | | | | | | |  |  |  |  |
| M | 0007409 | axonogenesis | EN-124k-90-group8796.Contig2 | | | | | | | | | |  |  |  |  |
| M | 0007409 | axonogenesis | EN-124k-90-group8799.Contig2 | | | | | | | | | |  |  |  |  |
| M | 0007409 | axonogenesis | EN-124k-90-group8850.Contig1 | | | | | | | | | |  |  |  |  |
| M | 0007409 | axonogenesis | EN-124k-90-group8897.Contig1 | | | | | | | | | |  |  |  |  |
| M | 0007409 | axonogenesis | EN-124k-90-group8905.Contig1 | | | | | | | | | |  |  |  |  |
| M | 0007409 | axonogenesis | EN-124k-90-group8905.Contig2 | | | | | | | | | |  |  |  |  |
| M | 0007409 | axonogenesis | EN-124k-90-group8908.Contig2 | | | | | | | | | |  |  |  |  |
| M | 0007409 | axonogenesis | EN-124k-90-group8937.Contig1 | | | | | | | | | |  |  |  |  |
| M | 0007409 | axonogenesis | EN-124k-90-group8937.Contig2 | | | | | | | | | |  |  |  |  |
| A | 0007409 | axonogenesis | EN-124k-90-group8957.Contig1 | | | | | | | | | |  |  |  |  |
| M | 0007409 | axonogenesis | EN-124k-90-group8988.Contig1 | | | | | | | | | |  |  |  |  |
| A | 0007409 | axonogenesis | EN-124k-90-group9012.Contig1 | | | | | | | | | |  |  |  |  |
| M | 0007409 | axonogenesis | EN-124k-90-group9079.Contig1 | | | | | | | | | |  |  |  |  |
| M | 0007409 | axonogenesis | EN-124k-90-group9165.Contig1 | | | | | | | | | |  |  |  |  |
| M | 0007409 | axonogenesis | EN-124k-90-group9168.Contig1 | | | | | | | | | |  |  |  |  |
| M | 0007409 | axonogenesis | EN-124k-90-group9238.Contig1 | | | | | | | | | |  |  |  |  |
| A | 0007409 | axonogenesis | EN-124k-90-group9245.Contig1 | | | | | | | | | |  |  |  |  |
| M | 0007409 | axonogenesis | EN-124k-90-group9249.Contig1 | | | | | | | | | |  |  |  |  |
| M | 0007409 | axonogenesis | EN-124k-90-group9278.Contig2 | | | | | | | | | |  |  |  |  |
| M | 0007409 | axonogenesis | EN-124k-90-group9299.Contig3 | | | | | | | | | |  |  |  |  |
| M | 0007409 | axonogenesis | EN-124k-90-group9317.Contig1 | | | | | | | | | |  |  |  |  |
| M | 0007409 | axonogenesis | EN-124k-90-group9341.Contig1 | | | | | | | | | |  |  |  |  |
| M | 0007409 | axonogenesis | EN-124k-90-group9388.Contig1 | | | | | | | | | |  |  |  |  |
| M | 0007409 | axonogenesis | EN-124k-90-group9388.Contig2 | | | | | | | | | |  |  |  |  |
| M | 0007409 | axonogenesis | EN-124k-90-group9420.Contig1 | | | | | | | | | |  |  |  |  |
| M | 0007409 | axonogenesis | EN-124k-90-group9446.Contig1 | | | | | | | | | |  |  |  |  |
| M | 0007409 | axonogenesis | EN-124k-90-group9479.Contig1 | | | | | | | | | |  |  |  |  |
| M | 0007409 | axonogenesis | EN-124k-90-group9487.Contig2 | | | | | | | | | |  |  |  |  |
| M | 0007409 | axonogenesis | EN-124k-90-group9541.Contig1 | | | | | | | | | |  |  |  |  |
| M | 0007409 | axonogenesis | EN-124k-90-group9586.Contig1 | | | | | | | | | |  |  |  |  |
| E | 0007409 | axonogenesis | EN-124k-90-group9586.Contig2 | | | | | | | | | |  |  |  |  |
| M | 0007409 | axonogenesis | EN-124k-90-group9620.Contig1 | | | | | | | | | |  |  |  |  |
| M | 0007409 | axonogenesis | EN-124k-90-group9650.Contig1 | | | | | | | | | |  |  |  |  |
| M | 0007409 | axonogenesis | EN-124k-90-group9692.Contig1 | | | | | | | | | |  |  |  |  |
| A | 0007409 | axonogenesis | EN-124k-90-group9767.Contig1 | | | | | | | | | |  |  |  |  |
| M | 0007409 | axonogenesis | EN-124k-90-group9771.Contig1 | | | | | | | | | |  |  |  |  |
| M | 0007409 | axonogenesis | EN-124k-90-group9771.Contig2 | | | | | | | | | |  |  |  |  |
| M | 0007409 | axonogenesis | EN-124k-90-group9794.Contig1 | | | | | | | | | |  |  |  |  |
| M | 0007409 | axonogenesis | EN-124k-90-group9794.Contig2 | | | | | | | | | |  |  |  |  |
| M | 0007409 | axonogenesis | EN-124k-90-group9819.Contig1 | | | | | | | | | |  |  |  |  |
| M | 0007409 | axonogenesis | EN-124k-90-group9843.Contig1 | | | | | | | | | |  |  |  |  |
| M | 0007409 | axonogenesis | EN-124k-90-group9849.Contig1 | | | | | | | | | |  |  |  |  |
| M | 0007409 | axonogenesis | EN-124k-90-group9953.Contig1 | | | | | | | | | |  |  |  |  |
| M | 0007409 | axonogenesis | EN-124k-90-group10046.Contig1 | | | | | | | | | |  |  |  |  |
| M | 0007409 | axonogenesis | EN-124k-90-group10053.Contig1 | | | | | | | | | |  |  |  |  |
| M | 0007409 | axonogenesis | EN-124k-90-group10056.Contig1 | | | | | | | | | |  |  |  |  |
| A | 0007409 | axonogenesis | EN-124k-90-group10065.Contig1 | | | | | | | | | |  |  |  |  |
| M | 0007409 | axonogenesis | EN-124k-90-group10085.Contig1 | | | | | | | | | |  |  |  |  |
| M | 0007409 | axonogenesis | EN-124k-90-group10085.Contig2 | | | | | | | | | |  |  |  |  |
| M | 0007409 | axonogenesis | EN-124k-90-group10221.Contig1 | | | | | | | | | |  |  |  |  |
| M | 0007409 | axonogenesis | EN-124k-90-group10290.Contig2 | | | | | | | | | |  |  |  |  |
| A | 0007409 | axonogenesis | EN-124k-90-group10370.Contig1 | | | | | | | | | |  |  |  |  |
| M | 0007409 | axonogenesis | EN-124k-90-group10394.Contig1 | | | | | | | | | |  |  |  |  |
| M | 0007409 | axonogenesis | EN-124k-90-group10450.Contig1 | | | | | | | | | |  |  |  |  |
| M | 0007409 | axonogenesis | EN-124k-90-group10467.Contig1 | | | | | | | | | |  |  |  |  |
| M | 0007409 | axonogenesis | EN-124k-90-group10486.Contig1 | | | | | | | | | |  |  |  |  |
| M | 0007409 | axonogenesis | EN-124k-90-group10528.Contig1 | | | | | | | | | |  |  |  |  |
| E | 0007409 | axonogenesis | EN-124k-90-group10565.Contig1 | | | | | | | | | |  |  |  |  |
| M | 0007409 | axonogenesis | EN-124k-90-group10598.Contig1 | | | | | | | | | |  |  |  |  |
| M | 0007409 | axonogenesis | EN-124k-90-group10630.Contig2 | | | | | | | | | |  |  |  |  |
| M | 0007409 | axonogenesis | EN-124k-90-group10637.Contig1 | | | | | | | | | |  |  |  |  |
| M | 0007409 | axonogenesis | EN-124k-90-group10650.Contig1 | | | | | | | | | |  |  |  |  |
| M | 0007409 | axonogenesis | EN-124k-90-group10652.Contig1 | | | | | | | | | |  |  |  |  |
| M | 0007409 | axonogenesis | EN-124k-90-group10717.Contig1 | | | | | | | | | |  |  |  |  |
| E | 0007409 | axonogenesis | EN-124k-90-group10742.Contig1 | | | | | | | | | |  |  |  |  |
| M | 0007409 | axonogenesis | EN-124k-90-group10750.Contig1 | | | | | | | | | |  |  |  |  |
| M | 0007409 | axonogenesis | EN-124k-90-group10790.Contig1 | | | | | | | | | |  |  |  |  |
| A | 0007409 | axonogenesis | EN-124k-90-group10802.Contig1 | | | | | | | | | |  |  |  |  |
| M | 0007409 | axonogenesis | EN-124k-90-group10823.Contig1 | | | | | | | | | |  |  |  |  |
| M | 0007409 | axonogenesis | EN-124k-90-group10824.Contig1 | | | | | | | | | |  |  |  |  |
| M | 0007409 | axonogenesis | EN-124k-90-group10838.Contig1 | | | | | | | | | |  |  |  |  |
| M | 0007409 | axonogenesis | EN-124k-90-group10838.Contig2 | | | | | | | | | |  |  |  |  |
| M | 0007409 | axonogenesis | EN-124k-90-group10843.Contig1 | | | | | | | | | |  |  |  |  |
| A | 0007409 | axonogenesis | EN-124k-90-group10847.Contig1 | | | | | | | | | |  |  |  |  |
| M | 0007409 | axonogenesis | EN-124k-90-group10875.Contig1 | | | | | | | | | |  |  |  |  |
| M | 0007409 | axonogenesis | EN-124k-90-group10877.Contig1 | | | | | | | | | |  |  |  |  |
| M | 0007409 | axonogenesis | EN-124k-90-group10881.Contig1 | | | | | | | | | |  |  |  |  |
| M | 0007409 | axonogenesis | EN-124k-90-group10907.Contig1 | | | | | | | | | |  |  |  |  |
| M | 0007409 | axonogenesis | EN-124k-90-group10912.Contig1 | | | | | | | | | |  |  |  |  |
| M | 0007409 | axonogenesis | EN-124k-90-group10928.Contig1 | | | | | | | | | |  |  |  |  |
| M | 0007409 | axonogenesis | EN-124k-90-group10950.Contig1 | | | | | | | | | |  |  |  |  |
| E | 0007409 | axonogenesis | EN-124k-90-group10984.Contig2 | | | | | | | | | |  |  |  |  |
| M | 0007409 | axonogenesis | EN-124k-90-group10990.Contig1 | | | | | | | | | |  |  |  |  |
| M | 0007409 | axonogenesis | EN-124k-90-group11082.Contig1 | | | | | | | | | |  |  |  |  |
| M | 0007409 | axonogenesis | EN-124k-90-group11100.Contig1 | | | | | | | | | |  |  |  |  |
| M | 0007409 | axonogenesis | EN-124k-90-group11223.Contig1 | | | | | | | | | |  |  |  |  |
| M | 0007409 | axonogenesis | EN-124k-90-group11228.Contig1 | | | | | | | | | |  |  |  |  |
| M | 0007409 | axonogenesis | EN-124k-90-group11235.Contig1 | | | | | | | | | |  |  |  |  |
| M | 0007409 | axonogenesis | EN-124k-90-group11250.Contig1 | | | | | | | | | |  |  |  |  |
| M | 0007409 | axonogenesis | EN-124k-90-group11317.Contig1 | | | | | | | | | |  |  |  |  |
| M | 0007409 | axonogenesis | EN-124k-90-group11317.Contig2 | | | | | | | | | |  |  |  |  |
| M | 0007409 | axonogenesis | EN-124k-90-group11399.Contig2 | | | | | | | | | |  |  |  |  |
| M | 0007409 | axonogenesis | EN-124k-90-group11408.Contig1 | | | | | | | | | |  |  |  |  |
| M | 0007409 | axonogenesis | EN-124k-90-group11413.Contig1 | | | | | | | | | |  |  |  |  |
| M | 0007409 | axonogenesis | EN-124k-90-group11508.Contig1 | | | | | | | | | |  |  |  |  |
| M | 0007409 | axonogenesis | EN-124k-90-group11519.Contig1 | | | | | | | | | |  |  |  |  |
| M | 0007409 | axonogenesis | EN-124k-90-group11686.Contig1 | | | | | | | | | |  |  |  |  |
| M | 0007409 | axonogenesis | EN-124k-90-group11723.Contig1 | | | | | | | | | |  |  |  |  |
| M | 0007409 | axonogenesis | EN-124k-90-group11737.Contig1 | | | | | | | | | |  |  |  |  |
| M | 0007409 | axonogenesis | EN-124k-90-group11808.Contig1 | | | | | | | | | |  |  |  |  |
| M | 0007409 | axonogenesis | EN-124k-90-group12003.Contig1 | | | | | | | | | |  |  |  |  |
| M | 0007409 | axonogenesis | EN-124k-90-group12008.Contig1 | | | | | | | | | |  |  |  |  |
| M | 0007409 | axonogenesis | EN-124k-90-group12099.Contig1 | | | | | | | | | |  |  |  |  |
| M | 0007409 | axonogenesis | EN-124k-90-group12337.Contig1 | | | | | | | | | |  |  |  |  |
| M | 0007409 | axonogenesis | EN-124k-90-group12337.Contig2 | | | | | | | | | |  |  |  |  |
| M | 0007409 | axonogenesis | EN-124k-90-group12470.Contig1 | | | | | | | | | |  |  |  |  |
| M | 0007409 | axonogenesis | EN-124k-90-group12519.Contig1 | | | | | | | | | |  |  |  |  |
| E | 0007409 | axonogenesis | EN-124k-90-group12524.Contig1 | | | | | | | | | |  |  |  |  |
| M | 0007409 | axonogenesis | EN-124k-90-group12530.Contig1 | | | | | | | | | |  |  |  |  |
| M | 0007409 | axonogenesis | EN-124k-90-group12530.Contig2 | | | | | | | | | |  |  |  |  |
| E | 0007409 | axonogenesis | EN-124k-90-group12535.Contig1 | | | | | | | | | |  |  |  |  |
| M | 0007409 | axonogenesis | EN-124k-90-group12594.Contig1 | | | | | | | | | |  |  |  |  |
| A | 0007409 | axonogenesis | EN-124k-90-group12616.Contig1 | | | | | | | | | |  |  |  |  |
| M | 0007409 | axonogenesis | EN-124k-90-group12638.Contig1 | | | | | | | | | |  |  |  |  |
| E | 0007409 | axonogenesis | EN-124k-90-group12653.Contig1 | | | | | | | | | |  |  |  |  |
| M | 0007409 | axonogenesis | EN-124k-90-group12654.Contig1 | | | | | | | | | |  |  |  |  |
| M | 0007409 | axonogenesis | EN-124k-90-group12751.Contig1 | | | | | | | | | |  |  |  |  |
| M | 0007409 | axonogenesis | EN-124k-90-group12751.Contig2 | | | | | | | | | |  |  |  |  |
| M | 0007409 | axonogenesis | EN-124k-90-group12751.Contig3 | | | | | | | | | |  |  |  |  |
| M | 0007409 | axonogenesis | EN-124k-90-group12755.Contig1 | | | | | | | | | |  |  |  |  |
| A | 0007409 | axonogenesis | EN-124k-90-group12774.Contig1 | | | | | | | | | |  |  |  |  |
| A | 0007409 | axonogenesis | EN-124k-90-group12832.Contig1 | | | | | | | | | |  |  |  |  |
| M | 0007409 | axonogenesis | EN-124k-90-group12836.Contig1 | | | | | | | | | |  |  |  |  |
| M | 0007409 | axonogenesis | EN-124k-90-group12881.Contig1 | | | | | | | | | |  |  |  |  |
| M | 0007409 | axonogenesis | EN-124k-90-group12960.Contig1 | | | | | | | | | |  |  |  |  |
| M | 0007409 | axonogenesis | EN-124k-90-group12973.Contig1 | | | | | | | | | |  |  |  |  |
| M | 0007409 | axonogenesis | EN-124k-90-group13025.Contig1 | | | | | | | | | |  |  |  |  |
| M | 0007409 | axonogenesis | EN-124k-90-group13095.Contig1 | | | | | | | | | |  |  |  |  |
| M | 0007409 | axonogenesis | EN-124k-90-group13107.Contig1 | | | | | | | | | |  |  |  |  |
| M | 0007409 | axonogenesis | EN-124k-90-group13121.Contig1 | | | | | | | | | |  |  |  |  |
| M | 0007409 | axonogenesis | EN-124k-90-group13235.Contig1 | | | | | | | | | |  |  |  |  |
| M | 0007409 | axonogenesis | EN-124k-90-group13235.Contig2 | | | | | | | | | |  |  |  |  |
| E | 0007409 | axonogenesis | EN-124k-90-group13342.Contig1 | | | | | | | | | |  |  |  |  |
| M | 0007409 | axonogenesis | EN-124k-90-group13502.Contig2 | | | | | | | | | |  |  |  |  |
| M | 0007409 | axonogenesis | EN-124k-90-group13579.Contig3 | | | | | | | | | |  |  |  |  |
| M | 0007409 | axonogenesis | EN-124k-90-group13646.Contig1 | | | | | | | | | |  |  |  |  |
| M | 0007409 | axonogenesis | EN-124k-90-group14027.Contig1 | | | | | | | | | |  |  |  |  |
| E | 0007409 | axonogenesis | EN-124k-90-group14073.Contig1 | | | | | | | | | |  |  |  |  |
| A | 0007409 | axonogenesis | EN-124k-90-group14199.Contig1 | | | | | | | | | |  |  |  |  |
| M | 0007409 | axonogenesis | EN-124k-90-group14390.Contig2 | | | | | | | | | |  |  |  |  |
| M | 0007409 | axonogenesis | EN-124k-90-group14577.Contig1 | | | | | | | | | |  |  |  |  |
| M | 0007409 | axonogenesis | EN-124k-90-group14580.Contig1 | | | | | | | | | |  |  |  |  |
| M | 0007409 | axonogenesis | EN-124k-90-group14708.Contig1 | | | | | | | | | |  |  |  |  |
| M | 0007409 | axonogenesis | EN-124k-90-group14794.Contig1 | | | | | | | | | |  |  |  |  |
| M | 0007409 | axonogenesis | EN-124k-90-group15757.Contig1 | | | | | | | | | |  |  |  |  |
| A | 0007409 | axonogenesis | EN-124k-90-group29.gs\_82128 | | | | | | | | | |  |  |  |  |
| A | 0007409 | axonogenesis | EN-124k-90-group65.gs\_37667 | | | | | | | | | |  |  |  |  |
| E | 0007409 | axonogenesis | EN-124k-90-group70.jgi\_contig\_JGI\_CBBP17523\_fwd | | | | | | | | | | | | |  |
| A | 0007409 | axonogenesis | EN-124k-90-group230.gs\_25885 | | | | | | | | | |  |  |  |  |
| E | 0007409 | axonogenesis | EN-124k-90-group232.jgi\_contig\_JGI\_CBBP16338\_fwd | | | | | | | | | | | | |  |
| E | 0007409 | axonogenesis | EN-124k-90-group232.jgi\_contig\_JGI\_CBBP9823\_fwd | | | | | | | | | | | | |  |
| E | 0007409 | axonogenesis | EN-124k-90-group232.jgi\_contig\_JGI\_CBBP10596\_fwd | | | | | | | | | | | | |  |
| A | 0007409 | axonogenesis | EN-124k-90-group294.gs\_52577 | | | | | | | | | |  |  |  |  |
| A | 0007409 | axonogenesis | EN-124k-90-group735.gs\_7849 | | | | | | | | | |  |  |  |  |
| E | 0007409 | axonogenesis | EN-124k-90-group760.jgi\_contig\_JGI\_CBBP4536\_fwd | | | | | | | | | | | | |  |
| E | 0007409 | axonogenesis | EN-124k-90-group850.EN\_iowa\_1547 | | | | | | | | | | |  |  |  |
| E | 0007409 | axonogenesis | EN-124k-90-group868.jgi\_contig\_JGI\_CBBP6965\_fwd | | | | | | | | | | | | |  |
| A | 0007409 | axonogenesis | EN-124k-90-group868.gs\_12961 | | | | | | | | | |  |  |  |  |
| E | 0007409 | axonogenesis | EN-124k-90-group900.EN\_iowa\_15436 | | | | | | | | | | |  |  |  |
| A | 0007409 | axonogenesis | EN-124k-90-group900.gs\_31783 | | | | | | | | | |  |  |  |  |
| A | 0007409 | axonogenesis | EN-124k-90-group900.gs\_71447 | | | | | | | | | |  |  |  |  |
| E | 0007409 | axonogenesis | EN-124k-90-group939.jgi\_contig\_JGI\_CBBP15138\_fwd | | | | | | | | | | | | |  |
| A | 0007409 | axonogenesis | EN-124k-90-group986.gs\_75970 | | | | | | | | | |  |  |  |  |
| A | 0007409 | axonogenesis | EN-124k-90-group986.gs\_7329 | | | | | | | | | |  |  |  |  |
| A | 0007409 | axonogenesis | EN-124k-90-group986.gs\_9698 | | | | | | | | | |  |  |  |  |
| E | 0007409 | axonogenesis | EN-124k-90-group1135.jgi\_unpaired\_JGI\_CBBP19047\_fwd | | | | | | | | | | | | | |
| E | 0007409 | axonogenesis | EN-124k-90-group1135.jgi\_contig\_JGI\_CBBP19919\_fwd | | | | | | | | | | | | |  |
| A | 0007409 | axonogenesis | EN-124k-90-group1135.gs\_2733 | | | | | | | | | |  |  |  |  |
| E | 0007409 | axonogenesis | EN-124k-90-group1135.EN\_iowa\_9861 | | | | | | | | | | |  |  |  |
| A | 0007409 | axonogenesis | EN-124k-90-group1135.gs\_17825 | | | | | | | | | |  |  |  |  |
| A | 0007409 | axonogenesis | EN-124k-90-group1153.gs\_635 | | | | | | | | | |  |  |  |  |
| A | 0007409 | axonogenesis | EN-124k-90-group1153.gs\_33239 | | | | | | | | | |  |  |  |  |
| A | 0007409 | axonogenesis | EN-124k-90-group1290.gs\_26142 | | | | | | | | | |  |  |  |  |
| A | 0007409 | axonogenesis | EN-124k-90-group1290.gs\_22118 | | | | | | | | | |  |  |  |  |
| A | 0007409 | axonogenesis | EN-124k-90-group1290.gs\_35147 | | | | | | | | | |  |  |  |  |
| A | 0007409 | axonogenesis | EN-124k-90-group1290.gs\_13448 | | | | | | | | | |  |  |  |  |
| A | 0007409 | axonogenesis | EN-124k-90-group1290.gs\_16770 | | | | | | | | | |  |  |  |  |
| E | 0007409 | axonogenesis | EN-124k-90-group1290.EN\_iowa\_15637 | | | | | | | | | | |  |  |  |
| A | 0007409 | axonogenesis | EN-124k-90-group1290.gs\_28522 | | | | | | | | | |  |  |  |  |
| A | 0007409 | axonogenesis | EN-124k-90-group1290.gs\_84561 | | | | | | | | | |  |  |  |  |
| A | 0007409 | axonogenesis | EN-124k-90-group1408.gs\_20773 | | | | | | | | | |  |  |  |  |
| A | 0007409 | axonogenesis | EN-124k-90-group1423.gs\_16014 | | | | | | | | | |  |  |  |  |
| A | 0007409 | axonogenesis | EN-124k-90-group1423.gs\_11172 | | | | | | | | | |  |  |  |  |
| A | 0007409 | axonogenesis | EN-124k-90-group1423.gs\_52362 | | | | | | | | | |  |  |  |  |
| A | 0007409 | axonogenesis | EN-124k-90-group1423.gs\_75717 | | | | | | | | | |  |  |  |  |
| E | 0007409 | axonogenesis | EN-124k-90-group1456.jgi\_contig\_JGI\_CBBP4193\_fwd | | | | | | | | | | | | |  |
| E | 0007409 | axonogenesis | EN-124k-90-group1493.jgi\_contig\_JGI\_CBBP6493\_fwd | | | | | | | | | | | | |  |
| A | 0007409 | axonogenesis | EN-124k-90-group1521.gs\_86350 | | | | | | | | | |  |  |  |  |
| E | 0007409 | axonogenesis | EN-124k-90-group1554.jgi\_contig\_JGI\_CBBP18583\_fwd | | | | | | | | | | | | |  |
| A | 0007409 | axonogenesis | EN-124k-90-group1654.gs\_12044 | | | | | | | | | |  |  |  |  |
| E | 0007409 | axonogenesis | EN-124k-90-group1699.jgi\_contig\_JGI\_CBBP7344\_fwd | | | | | | | | | | | | |  |
| A | 0007409 | axonogenesis | EN-124k-90-group1699.gs\_33563 | | | | | | | | | |  |  |  |  |
| A | 0007409 | axonogenesis | EN-124k-90-group1782.gs\_16663 | | | | | | | | | |  |  |  |  |
| A | 0007409 | axonogenesis | EN-124k-90-group1798.gs\_54312 | | | | | | | | | |  |  |  |  |
| A | 0007409 | axonogenesis | EN-124k-90-group1798.gs\_39367 | | | | | | | | | |  |  |  |  |
| E | 0007409 | axonogenesis | EN-124k-90-group1798.EN\_iowa\_16312 | | | | | | | | | | |  |  |  |
| A | 0007409 | axonogenesis | EN-124k-90-group1798.gs\_29292 | | | | | | | | | |  |  |  |  |
| A | 0007409 | axonogenesis | EN-124k-90-group1798.gs\_11661 | | | | | | | | | |  |  |  |  |
| A | 0007409 | axonogenesis | EN-124k-90-group1798.gs\_36996 | | | | | | | | | |  |  |  |  |
| A | 0007409 | axonogenesis | EN-124k-90-group1798.gs\_46615 | | | | | | | | | |  |  |  |  |
| A | 0007409 | axonogenesis | EN-124k-90-group1798.gs\_54963 | | | | | | | | | |  |  |  |  |
| A | 0007409 | axonogenesis | EN-124k-90-group1798.gs\_4498 | | | | | | | | | |  |  |  |  |
| A | 0007409 | axonogenesis | EN-124k-90-group1798.gs\_7178 | | | | | | | | | |  |  |  |  |
| A | 0007409 | axonogenesis | EN-124k-90-group1798.gs\_7576 | | | | | | | | | |  |  |  |  |
| A | 0007409 | axonogenesis | EN-124k-90-group1798.gs\_11790 | | | | | | | | | |  |  |  |  |
| E | 0007409 | axonogenesis | EN-124k-90-group1819.jgi\_contig\_JGI\_CBBP4948\_fwd | | | | | | | | | | | | |  |
| E | 0007409 | axonogenesis | EN-124k-90-group2077.jgi\_paired\_JGI\_CBBP13793\_rev | | | | | | | | | | | | |  |
| E | 0007409 | axonogenesis | EN-124k-90-group2163.jgi\_contig\_JGI\_CBBP7370\_fwd | | | | | | | | | | | | |  |
| E | 0007409 | axonogenesis | EN-124k-90-group2221.jgi\_contig\_JGI\_CBBP11225\_fwd | | | | | | | | | | | | |  |
| E | 0007409 | axonogenesis | EN-124k-90-group2409.EN\_iowa\_9419 | | | | | | | | | | |  |  |  |
| E | 0007409 | axonogenesis | EN-124k-90-group2409.EN\_iowa\_12940 | | | | | | | | | | |  |  |  |
| E | 0007409 | axonogenesis | EN-124k-90-group2443.EN\_iowa\_8559 | | | | | | | | | | |  |  |  |
| A | 0007409 | axonogenesis | EN-124k-90-group2449.gs\_35369 | | | | | | | | | |  |  |  |  |
| E | 0007409 | axonogenesis | EN-124k-90-group2451.jgi\_contig\_JGI\_CBBP13284\_fwd | | | | | | | | | | | | |  |
| E | 0007409 | axonogenesis | EN-124k-90-group2525.jgi\_contig\_JGI\_CBBP2745\_fwd | | | | | | | | | | | | |  |
| E | 0007409 | axonogenesis | EN-124k-90-group2525.jgi\_contig\_JGI\_CBBP12002\_fwd | | | | | | | | | | | | |  |
| A | 0007409 | axonogenesis | EN-124k-90-group2525.gs\_26865 | | | | | | | | | |  |  |  |  |
| A | 0007409 | axonogenesis | EN-124k-90-group2525.gs\_28080 | | | | | | | | | |  |  |  |  |
| E | 0007409 | axonogenesis | EN-124k-90-group2525.jgi\_contig\_JGI\_CBBP7217\_fwd | | | | | | | | | | | | |  |
| E | 0007409 | axonogenesis | EN-124k-90-group2600.jgi\_contig\_JGI\_CBBP7087\_fwd | | | | | | | | | | | | |  |
| E | 0007409 | axonogenesis | EN-124k-90-group2600.jgi\_contig\_JGI\_CBBP2795\_fwd | | | | | | | | | | | | |  |
| A | 0007409 | axonogenesis | EN-124k-90-group2619.gs\_45204 | | | | | | | | | |  |  |  |  |
| A | 0007409 | axonogenesis | EN-124k-90-group2811.gs\_23030 | | | | | | | | | |  |  |  |  |
| A | 0007409 | axonogenesis | EN-124k-90-group2811.gs\_38224 | | | | | | | | | |  |  |  |  |
| A | 0007409 | axonogenesis | EN-124k-90-group2811.gs\_15146 | | | | | | | | | |  |  |  |  |
| A | 0007409 | axonogenesis | EN-124k-90-group2903.gs\_16952 | | | | | | | | | |  |  |  |  |
| A | 0007409 | axonogenesis | EN-124k-90-group3067.gs\_19498 | | | | | | | | | |  |  |  |  |
| E | 0007409 | axonogenesis | EN-124k-90-group3114.jgi\_paired\_JGI\_CBBP6377\_fwd | | | | | | | | | | | | |  |
| A | 0007409 | axonogenesis | EN-124k-90-group3202.gs\_15566 | | | | | | | | | |  |  |  |  |
| A | 0007409 | axonogenesis | EN-124k-90-group3238.gs\_87325 | | | | | | | | | |  |  |  |  |
| A | 0007409 | axonogenesis | EN-124k-90-group3357.gs\_11871 | | | | | | | | | |  |  |  |  |
| A | 0007409 | axonogenesis | EN-124k-90-group3522.gs\_9768 | | | | | | | | | |  |  |  |  |
| E | 0007409 | axonogenesis | EN-124k-90-group3584.jgi\_paired\_JGI\_CBBP15905\_fwd | | | | | | | | | | | | |  |
| A | 0007409 | axonogenesis | EN-124k-90-group3594.gs\_29852 | | | | | | | | | |  |  |  |  |
| E | 0007409 | axonogenesis | EN-124k-90-group3611.EN\_iowa\_4463 | | | | | | | | | | |  |  |  |
| A | 0007409 | axonogenesis | EN-124k-90-group3713.gs\_7861 | | | | | | | | | |  |  |  |  |
| E | 0007409 | axonogenesis | EN-124k-90-group3872.jgi\_contig\_JGI\_CBBP10436\_fwd | | | | | | | | | | | | |  |
| E | 0007409 | axonogenesis | EN-124k-90-group3872.EN\_iowa\_18089 | | | | | | | | | | |  |  |  |
| A | 0007409 | axonogenesis | EN-124k-90-group3883.gs\_85360 | | | | | | | | | |  |  |  |  |
| E | 0007409 | axonogenesis | EN-124k-90-group3905.jgi\_contig\_JGI\_CBBP12601\_fwd | | | | | | | | | | | | |  |
| E | 0007409 | axonogenesis | EN-124k-90-group3947.EN\_iowa\_3123 | | | | | | | | | | |  |  |  |
| E | 0007409 | axonogenesis | EN-124k-90-group3966.jgi\_contig\_JGI\_CBBP12693\_fwd | | | | | | | | | | | | |  |
| E | 0007409 | axonogenesis | EN-124k-90-group3966.jgi\_contig\_JGI\_CBBP7455\_fwd | | | | | | | | | | | | |  |
| E | 0007409 | axonogenesis | EN-124k-90-group3966.EN\_iowa\_15266 | | | | | | | | | | |  |  |  |
| E | 0007409 | axonogenesis | EN-124k-90-group4049.EN\_iowa\_15523 | | | | | | | | | | |  |  |  |
| E | 0007409 | axonogenesis | EN-124k-90-group4154.EN\_iowa\_7753 | | | | | | | | | | |  |  |  |
| E | 0007409 | axonogenesis | EN-124k-90-group4154.EN\_iowa\_1478 | | | | | | | | | | |  |  |  |
| A | 0007409 | axonogenesis | EN-124k-90-group4349.gs\_17809 | | | | | | | | | |  |  |  |  |
| E | 0007409 | axonogenesis | EN-124k-90-group4600.jgi\_contig\_JGI\_CBBP12800\_fwd | | | | | | | | | | | | |  |
| E | 0007409 | axonogenesis | EN-124k-90-group4808.jgi\_contig\_JGI\_CBBP12941\_fwd | | | | | | | | | | | | |  |
| E | 0007409 | axonogenesis | EN-124k-90-group4808.jgi\_contig\_JGI\_CBBP16155\_fwd | | | | | | | | | | | | |  |
| A | 0007409 | axonogenesis | EN-124k-90-group4824.gs\_12585 | | | | | | | | | |  |  |  |  |
| E | 0007409 | axonogenesis | EN-124k-90-group5184.EN\_iowa\_8658 | | | | | | | | | | |  |  |  |
| A | 0007409 | axonogenesis | EN-124k-90-group5215.gs\_74365 | | | | | | | | | |  |  |  |  |
| A | 0007409 | axonogenesis | EN-124k-90-group5300.gs\_35256 | | | | | | | | | |  |  |  |  |
| A | 0007409 | axonogenesis | EN-124k-90-group5491.gs\_7631 | | | | | | | | | |  |  |  |  |
| A | 0007409 | axonogenesis | EN-124k-90-group5491.gs\_75129 | | | | | | | | | |  |  |  |  |
| A | 0007409 | axonogenesis | EN-124k-90-group5563.gs\_3008 | | | | | | | | | |  |  |  |  |
| E | 0007409 | axonogenesis | EN-124k-90-group5567.jgi\_paired\_JGI\_CBBP16996\_fwd | | | | | | | | | | | | |  |
| E | 0007409 | axonogenesis | EN-124k-90-group5607.jgi\_contig\_JGI\_CBBP445\_fwd | | | | | | | | | | | | |  |
| E | 0007409 | axonogenesis | EN-124k-90-group6204.EN\_iowa\_1799 | | | | | | | | | | |  |  |  |
| E | 0007409 | axonogenesis | EN-124k-90-group6204.EN\_iowa\_4716 | | | | | | | | | | |  |  |  |
| E | 0007409 | axonogenesis | EN-124k-90-group6687.EN\_iowa\_2719 | | | | | | | | | | |  |  |  |
| A | 0007409 | axonogenesis | EN-124k-90-group6883.gs\_22524 | | | | | | | | | |  |  |  |  |
| E | 0007409 | axonogenesis | EN-124k-90-group6923.jgi\_contig\_JGI\_CBBP17789\_fwd | | | | | | | | | | | | |  |
| A | 0007409 | axonogenesis | EN-124k-90-group7440.gs\_75782 | | | | | | | | | |  |  |  |  |
| A | 0007409 | axonogenesis | EN-124k-90-group7650.gs\_30549 | | | | | | | | | |  |  |  |  |
| A | 0007409 | axonogenesis | EN-124k-90-group7650.gs\_59944 | | | | | | | | | |  |  |  |  |
| E | 0007409 | axonogenesis | EN-124k-90-group8221.jgi\_contig\_JGI\_CBBP18338\_fwd | | | | | | | | | | | | |  |
| A | 0007409 | axonogenesis | EN-124k-90-group8484.gs\_85230 | | | | | | | | | |  |  |  |  |
| A | 0007409 | axonogenesis | EN-124k-90-group9299.gs\_87064 | | | | | | | | | |  |  |  |  |
| E | 0007409 | axonogenesis | EN-124k-90-group11408.jgi\_contig\_JGI\_CBBP16915\_fwd | | | | | | | | | | | | |  |
| E | 0007409 | axonogenesis | EN-124k-90-group12653.EN\_iowa\_13251 | | | | | | | | | | |  |  |  |
| A | 0021785 | branchiomotor neuron axon guidance | EN-124k-90-group16254.gs\_29060 | | | | | | | | | |  |  |  |  |
| A | 0021785 | branchiomotor neuron axon guidance | EN-124k-90-group16439.gs\_54729 | | | | | | | | | |  |  |  |  |
| A | 0021785 | branchiomotor neuron axon guidance | EN-124k-90-group16489.gs\_80464 | | | | | | | | | |  |  |  |  |
| A | 0021785 | branchiomotor neuron axon guidance | EN-124k-90-group16600.gs\_29512 | | | | | | | | | |  |  |  |  |
| A | 0021785 | branchiomotor neuron axon guidance | EN-124k-90-group16830.gs\_82512 | | | | | | | | | |  |  |  |  |
| A | 0021785 | branchiomotor neuron axon guidance | EN-124k-90-group16837.gs\_15971 | | | | | | | | | |  |  |  |  |
| A | 0021785 | branchiomotor neuron axon guidance | EN-124k-90-group16872.gs\_213 | | | | | | | | | |  |  |  |  |
| A | 0021785 | branchiomotor neuron axon guidance | EN-124k-90-group16929.gs\_43290 | | | | | | | | | |  |  |  |  |
| A | 0021785 | branchiomotor neuron axon guidance | EN-124k-90-group17360.gs\_72469 | | | | | | | | | |  |  |  |  |
| A | 0021785 | branchiomotor neuron axon guidance | EN-124k-90-group17487.gs\_82041 | | | | | | | | | |  |  |  |  |
| A | 0021785 | branchiomotor neuron axon guidance | EN-124k-90-group17572.gs\_7187 | | | | | | | | | |  |  |  |  |
| A | 0021785 | branchiomotor neuron axon guidance | EN-124k-90-group17582.gs\_70636 | | | | | | | | | |  |  |  |  |
| A | 0021785 | branchiomotor neuron axon guidance | EN-124k-90-group17776.gs\_56285 | | | | | | | | | |  |  |  |  |
| A | 0021785 | branchiomotor neuron axon guidance | EN-124k-90-group17892.gs\_49260 | | | | | | | | | |  |  |  |  |
| M | 0021785 | branchiomotor neuron axon guidance | EN-124k-90-group362.Contig2 | | | | | | | | | |  |  |  |  |
| M | 0021785 | branchiomotor neuron axon guidance | EN-124k-90-group2155.Contig1 | | | | | | | | | |  |  |  |  |
| M | 0021785 | branchiomotor neuron axon guidance | EN-124k-90-group5337.Contig1 | | | | | | | | | |  |  |  |  |
| M | 0021785 | branchiomotor neuron axon guidance | EN-124k-90-group8034.Contig1 | | | | | | | | | |  |  |  |  |
| M | 0021785 | branchiomotor neuron axon guidance | EN-124k-90-group9712.Contig1 | | | | | | | | | |  |  |  |  |
| M | 0021785 | branchiomotor neuron axon guidance | EN-124k-90-group14134.Contig1 | | | | | | | | | |  |  |  |  |
| A | 0048791 | calcium ion-dependent exocytosis of neurotransmitter | EN-124k-90-group16439.gs\_54729 | | | | | | | | | |  |  |  |  |
| A | 0048791 | calcium ion-dependent exocytosis of neurotransmitter | EN-124k-90-group16523.gs\_75670 | | | | | | | | | |  |  |  |  |
| A | 0048791 | calcium ion-dependent exocytosis of neurotransmitter | EN-124k-90-group16575.gs\_6 | | | | | | | | | |  |  |  |  |
| A | 0048791 | calcium ion-dependent exocytosis of neurotransmitter | EN-124k-90-group17471.gs\_85794 | | | | | | | | | |  |  |  |  |
| A | 0048791 | calcium ion-dependent exocytosis of neurotransmitter | EN-124k-90-group17572.gs\_7187 | | | | | | | | | |  |  |  |  |
| A | 0048791 | calcium ion-dependent exocytosis of neurotransmitter | EN-124k-90-group17702.gs\_18827 | | | | | | | | | |  |  |  |  |
| A | 0048791 | calcium ion-dependent exocytosis of neurotransmitter | EN-124k-90-group18342.gs\_65612 | | | | | | | | | |  |  |  |  |
| A | 0048791 | calcium ion-dependent exocytosis of neurotransmitter | EN-124k-90-group18480.gs\_449 | | | | | | | | | |  |  |  |  |
| M | 0048791 | calcium ion-dependent exocytosis of neurotransmitter | EN-124k-90-group4462.Contig1 | | | | | | | | | |  |  |  |  |
| M | 0048791 | calcium ion-dependent exocytosis of neurotransmitter | EN-124k-90-group5563.Contig1 | | | | | | | | | |  |  |  |  |
| M | 0048791 | calcium ion-dependent exocytosis of neurotransmitter | EN-124k-90-group5597.Contig2 | | | | | | | | | |  |  |  |  |
| A | 0048791 | calcium ion-dependent exocytosis of neurotransmitter | EN-124k-90-group5563.gs\_3008 | | | | | | | | | |  |  |  |  |
| A | 0048667 | cell morphogenesis involved in neuron differentiation | EN-124k-90-group86.gs\_20715 | | | | | | | | | |  |  |  |  |
| A | 0048667 | cell morphogenesis involved in neuron differentiation | EN-124k-90-group1020.gs\_69762 | | | | | | | | | |  |  |  |  |
| E | 0048667 | cell morphogenesis involved in neuron differentiation | EN-124k-90-group1825.jgi\_contig\_JGI\_CBBP14395\_fwd | | | | | | | | | | | | |  |
| E | 0048667 | cell morphogenesis involved in neuron differentiation | EN-124k-90-group6048.EN\_iowa\_911 | | | | | | | | | | |  |  |  |
| A | 0048667 | cell morphogenesis involved in neuron differentiation | EN-124k-90-group8023.gs\_82870 | | | | | | | | | |  |  |  |  |
| E | 0048667 | cell morphogenesis involved in neuron differentiation | EN-124k-90-group10878.jgi\_paired\_JGI\_CBBP952\_rev | | | | | | | | | | | | |  |
| A | 0048667 | cell morphogenesis involved in neuron differentiation | EN-124k-90-group11433.gs\_55747 | | | | | | | | | |  |  |  |  |
| E | 0048667 | cell morphogenesis involved in neuron differentiation | EN-124k-90-group13911.jgi\_contig\_JGI\_CBBP10628\_fwd | | | | | | | | | | | | |  |
| E | 0048667 | cell morphogenesis involved in neuron differentiation | EN-124k-90-group15895.jgi\_contig\_JGI\_CBBP12118\_fwd | | | | | | | | | | | | |  |
| A | 0048667 | cell morphogenesis involved in neuron differentiation | EN-124k-90-group16254.gs\_29060 | | | | | | | | | |  |  |  |  |
| A | 0048667 | cell morphogenesis involved in neuron differentiation | EN-124k-90-group16439.gs\_54729 | | | | | | | | | |  |  |  |  |
| A | 0048667 | cell morphogenesis involved in neuron differentiation | EN-124k-90-group16489.gs\_80464 | | | | | | | | | |  |  |  |  |
| A | 0048667 | cell morphogenesis involved in neuron differentiation | EN-124k-90-group16600.gs\_29512 | | | | | | | | | |  |  |  |  |
| A | 0048667 | cell morphogenesis involved in neuron differentiation | EN-124k-90-group16830.gs\_82512 | | | | | | | | | |  |  |  |  |
| A | 0048667 | cell morphogenesis involved in neuron differentiation | EN-124k-90-group16837.gs\_15971 | | | | | | | | | |  |  |  |  |
| A | 0048667 | cell morphogenesis involved in neuron differentiation | EN-124k-90-group16872.gs\_213 | | | | | | | | | |  |  |  |  |
| A | 0048667 | cell morphogenesis involved in neuron differentiation | EN-124k-90-group16929.gs\_43290 | | | | | | | | | |  |  |  |  |
| A | 0048667 | cell morphogenesis involved in neuron differentiation | EN-124k-90-group17360.gs\_72469 | | | | | | | | | |  |  |  |  |
| A | 0048667 | cell morphogenesis involved in neuron differentiation | EN-124k-90-group17487.gs\_82041 | | | | | | | | | |  |  |  |  |
| A | 0048667 | cell morphogenesis involved in neuron differentiation | EN-124k-90-group17563.gs\_80410 | | | | | | | | | |  |  |  |  |
| A | 0048667 | cell morphogenesis involved in neuron differentiation | EN-124k-90-group17572.gs\_7187 | | | | | | | | | |  |  |  |  |
| A | 0048667 | cell morphogenesis involved in neuron differentiation | EN-124k-90-group17582.gs\_70636 | | | | | | | | | |  |  |  |  |
| A | 0048667 | cell morphogenesis involved in neuron differentiation | EN-124k-90-group17776.gs\_56285 | | | | | | | | | |  |  |  |  |
| A | 0048667 | cell morphogenesis involved in neuron differentiation | EN-124k-90-group17892.gs\_49260 | | | | | | | | | |  |  |  |  |
| A | 0048667 | cell morphogenesis involved in neuron differentiation | EN-124k-90-group18038.gs\_30262 | | | | | | | | | |  |  |  |  |
| M | 0048667 | cell morphogenesis involved in neuron differentiation | EN-124k-90-group362.Contig2 | | | | | | | | | |  |  |  |  |
| M | 0048667 | cell morphogenesis involved in neuron differentiation | EN-124k-90-group2155.Contig1 | | | | | | | | | |  |  |  |  |
| M | 0048667 | cell morphogenesis involved in neuron differentiation | EN-124k-90-group2219.Contig1 | | | | | | | | | |  |  |  |  |
| M | 0048667 | cell morphogenesis involved in neuron differentiation | EN-124k-90-group2866.Contig1 | | | | | | | | | |  |  |  |  |
| M | 0048667 | cell morphogenesis involved in neuron differentiation | EN-124k-90-group2975.Contig1 | | | | | | | | | |  |  |  |  |
| M | 0048667 | cell morphogenesis involved in neuron differentiation | EN-124k-90-group4049.Contig1 | | | | | | | | | |  |  |  |  |
| M | 0048667 | cell morphogenesis involved in neuron differentiation | EN-124k-90-group5337.Contig1 | | | | | | | | | |  |  |  |  |
| M | 0048667 | cell morphogenesis involved in neuron differentiation | EN-124k-90-group7017.Contig1 | | | | | | | | | |  |  |  |  |
| M | 0048667 | cell morphogenesis involved in neuron differentiation | EN-124k-90-group7632.Contig1 | | | | | | | | | |  |  |  |  |
| M | 0048667 | cell morphogenesis involved in neuron differentiation | EN-124k-90-group7787.Contig1 | | | | | | | | | |  |  |  |  |
| M | 0048667 | cell morphogenesis involved in neuron differentiation | EN-124k-90-group7787.Contig2 | | | | | | | | | |  |  |  |  |
| M | 0048667 | cell morphogenesis involved in neuron differentiation | EN-124k-90-group7787.Contig3 | | | | | | | | | |  |  |  |  |
| M | 0048667 | cell morphogenesis involved in neuron differentiation | EN-124k-90-group8034.Contig1 | | | | | | | | | |  |  |  |  |
| M | 0048667 | cell morphogenesis involved in neuron differentiation | EN-124k-90-group9485.Contig4 | | | | | | | | | |  |  |  |  |
| M | 0048667 | cell morphogenesis involved in neuron differentiation | EN-124k-90-group9712.Contig1 | | | | | | | | | |  |  |  |  |
| E | 0048667 | cell morphogenesis involved in neuron differentiation | EN-124k-90-group12653.Contig1 | | | | | | | | | |  |  |  |  |
| M | 0048667 | cell morphogenesis involved in neuron differentiation | EN-124k-90-group14134.Contig1 | | | | | | | | | |  |  |  |  |
| E | 0048667 | cell morphogenesis involved in neuron differentiation | EN-124k-90-group15137.Contig1 | | | | | | | | | |  |  |  |  |
| E | 0048667 | cell morphogenesis involved in neuron differentiation | EN-124k-90-group4049.EN\_iowa\_15523 | | | | | | | | | | |  |  |  |
| E | 0048667 | cell morphogenesis involved in neuron differentiation | EN-124k-90-group12653.EN\_iowa\_13251 | | | | | | | | | | |  |  |  |
| M | 0021813 | cell-cell adhesion involved in neuronal-glial interactions involved in cerebral cortex radial glia guided migration | EN-124k-90-group7318.Contig2 | | | | | | | | | |  |  |  |  |
| A | 0021956 | central nervous system interneuron axonogenesis | EN-124k-90-group16830.gs\_82512 | | | | | | | | | |  |  |  |  |
| E | 0021955 | central nervous system neuron axonogenesis | EN-124k-90-group343.jgi\_contig\_JGI\_CBBP19299\_fwd | | | | | | | | | | | | |  |
| A | 0021955 | central nervous system neuron axonogenesis | EN-124k-90-group807.gs\_71281 | | | | | | | | | |  |  |  |  |
| E | 0021955 | central nervous system neuron axonogenesis | EN-124k-90-group5117.jgi\_paired\_JGI\_CBBP19153\_fwd | | | | | | | | | | | | |  |
| E | 0021955 | central nervous system neuron axonogenesis | EN-124k-90-group5834.jgi\_contig\_JGI\_CBBP12701\_fwd | | | | | | | | | | | | |  |
| E | 0021955 | central nervous system neuron axonogenesis | EN-124k-90-group5954.jgi\_paired\_JGI\_CBBP7330\_fwd | | | | | | | | | | | | |  |
| E | 0021955 | central nervous system neuron axonogenesis | EN-124k-90-group6737.jgi\_contig\_JGI\_CBBP17820\_fwd | | | | | | | | | | | | |  |
| E | 0021955 | central nervous system neuron axonogenesis | EN-124k-90-group7867.EN\_iowa\_18229 | | | | | | | | | | |  |  |  |
| A | 0021955 | central nervous system neuron axonogenesis | EN-124k-90-group9467.gs\_16039 | | | | | | | | | |  |  |  |  |
| E | 0021955 | central nervous system neuron axonogenesis | EN-124k-90-group9942.jgi\_paired\_JGI\_CBBP11089\_fwd | | | | | | | | | | | | |  |
| A | 0021955 | central nervous system neuron axonogenesis | EN-124k-90-group10935.gs\_64995 | | | | | | | | | |  |  |  |  |
| E | 0021955 | central nervous system neuron axonogenesis | EN-124k-90-group11500.EN\_iowa\_7830 | | | | | | | | | | |  |  |  |
| A | 0021955 | central nervous system neuron axonogenesis | EN-124k-90-group11738.gs\_53113 | | | | | | | | | |  |  |  |  |
| A | 0021955 | central nervous system neuron axonogenesis | EN-124k-90-group11821.gs\_86440 | | | | | | | | | |  |  |  |  |
| E | 0021955 | central nervous system neuron axonogenesis | EN-124k-90-group11882.EN\_iowa\_9262 | | | | | | | | | | |  |  |  |
| E | 0021955 | central nervous system neuron axonogenesis | EN-124k-90-group12012.jgi\_contig\_JGI\_CBBP10480\_fwd | | | | | | | | | | | | |  |
| E | 0021955 | central nervous system neuron axonogenesis | EN-124k-90-group13011.jgi\_paired\_JGI\_CBBP19322\_fwd | | | | | | | | | | | | |  |
| E | 0021955 | central nervous system neuron axonogenesis | EN-124k-90-group13366.jgi\_paired\_JGI\_CBBP16258\_fwd | | | | | | | | | | | | |  |
| E | 0021955 | central nervous system neuron axonogenesis | EN-124k-90-group13491.jgi\_paired\_JGI\_CBBP11089\_rev | | | | | | | | | | | | |  |
| E | 0021955 | central nervous system neuron axonogenesis | EN-124k-90-group14142.jgi\_contig\_JGI\_CBBP18747\_fwd | | | | | | | | | | | | |  |
| E | 0021955 | central nervous system neuron axonogenesis | EN-124k-90-group14311.jgi\_paired\_JGI\_CBBP682\_fwd | | | | | | | | | | | | |  |
| A | 0021955 | central nervous system neuron axonogenesis | EN-124k-90-group14545.gs\_14842 | | | | | | | | | |  |  |  |  |
| A | 0021955 | central nervous system neuron axonogenesis | EN-124k-90-group14563.gs\_48486 | | | | | | | | | |  |  |  |  |
| A | 0021955 | central nervous system neuron axonogenesis | EN-124k-90-group14948.gs\_26769 | | | | | | | | | |  |  |  |  |
| A | 0021955 | central nervous system neuron axonogenesis | EN-124k-90-group15198.gs\_65271 | | | | | | | | | |  |  |  |  |
| E | 0021955 | central nervous system neuron axonogenesis | EN-124k-90-group15290.EN\_iowa\_9903 | | | | | | | | | | |  |  |  |
| E | 0021955 | central nervous system neuron axonogenesis | EN-124k-90-group15320.jgi\_paired\_JGI\_CBBP9817\_fwd | | | | | | | | | | | | |  |
| E | 0021955 | central nervous system neuron axonogenesis | EN-124k-90-group15988.jgi\_unpaired\_JGI\_CBBP2255\_fwd | | | | | | | | | | | | | |
| A | 0021955 | central nervous system neuron axonogenesis | EN-124k-90-group16929.gs\_43290 | | | | | | | | | |  |  |  |  |
| A | 0021955 | central nervous system neuron axonogenesis | EN-124k-90-group17360.gs\_72469 | | | | | | | | | |  |  |  |  |
| A | 0021955 | central nervous system neuron axonogenesis | EN-124k-90-group17744.gs\_30255 | | | | | | | | | |  |  |  |  |
| A | 0021955 | central nervous system neuron axonogenesis | EN-124k-90-group17930.gs\_83513 | | | | | | | | | |  |  |  |  |
| E | 0021955 | central nervous system neuron axonogenesis | EN-124k-90-group17979.jgi\_contig\_JGI\_CBBP2421\_fwd | | | | | | | | | | | | |  |
| A | 0021955 | central nervous system neuron axonogenesis | EN-124k-90-group18148.gs\_80788 | | | | | | | | | |  |  |  |  |
| E | 0021955 | central nervous system neuron axonogenesis | EN-124k-90-group18538.jgi\_paired\_JGI\_CBBP19133\_rev | | | | | | | | | | | | |  |
| A | 0021955 | central nervous system neuron axonogenesis | EN-124k-90-group78.Contig1 | | | | | | | | |  |  |  |  |  |
| A | 0021955 | central nervous system neuron axonogenesis | EN-124k-90-group78.Contig2 | | | | | | | | |  |  |  |  |  |
| M | 0021955 | central nervous system neuron axonogenesis | EN-124k-90-group229.Contig1 | | | | | | | | | |  |  |  |  |
| M | 0021955 | central nervous system neuron axonogenesis | EN-124k-90-group230.Contig1 | | | | | | | | | |  |  |  |  |
| M | 0021955 | central nervous system neuron axonogenesis | EN-124k-90-group359.Contig1 | | | | | | | | | |  |  |  |  |
| M | 0021955 | central nervous system neuron axonogenesis | EN-124k-90-group359.Contig2 | | | | | | | | | |  |  |  |  |
| M | 0021955 | central nervous system neuron axonogenesis | EN-124k-90-group359.Contig3 | | | | | | | | | |  |  |  |  |
| M | 0021955 | central nervous system neuron axonogenesis | EN-124k-90-group359.Contig4 | | | | | | | | | |  |  |  |  |
| M | 0021955 | central nervous system neuron axonogenesis | EN-124k-90-group359.Contig5 | | | | | | | | | |  |  |  |  |
| M | 0021955 | central nervous system neuron axonogenesis | EN-124k-90-group481.Contig4 | | | | | | | | | |  |  |  |  |
| M | 0021955 | central nervous system neuron axonogenesis | EN-124k-90-group518.Contig2 | | | | | | | | | |  |  |  |  |
| M | 0021955 | central nervous system neuron axonogenesis | EN-124k-90-group593.Contig1 | | | | | | | | | |  |  |  |  |
| M | 0021955 | central nervous system neuron axonogenesis | EN-124k-90-group630.Contig2 | | | | | | | | | |  |  |  |  |
| M | 0021955 | central nervous system neuron axonogenesis | EN-124k-90-group658.Contig3 | | | | | | | | | |  |  |  |  |
| M | 0021955 | central nervous system neuron axonogenesis | EN-124k-90-group682.Contig1 | | | | | | | | | |  |  |  |  |
| M | 0021955 | central nervous system neuron axonogenesis | EN-124k-90-group881.Contig3 | | | | | | | | | |  |  |  |  |
| M | 0021955 | central nervous system neuron axonogenesis | EN-124k-90-group900.Contig1 | | | | | | | | | |  |  |  |  |
| M | 0021955 | central nervous system neuron axonogenesis | EN-124k-90-group939.Contig1 | | | | | | | | | |  |  |  |  |
| M | 0021955 | central nervous system neuron axonogenesis | EN-124k-90-group1091.Contig3 | | | | | | | | | |  |  |  |  |
| M | 0021955 | central nervous system neuron axonogenesis | EN-124k-90-group1106.Contig1 | | | | | | | | | |  |  |  |  |
| M | 0021955 | central nervous system neuron axonogenesis | EN-124k-90-group1302.Contig4 | | | | | | | | | |  |  |  |  |
| M | 0021955 | central nervous system neuron axonogenesis | EN-124k-90-group1401.Contig2 | | | | | | | | | |  |  |  |  |
| M | 0021955 | central nervous system neuron axonogenesis | EN-124k-90-group1642.Contig1 | | | | | | | | | |  |  |  |  |
| M | 0021955 | central nervous system neuron axonogenesis | EN-124k-90-group1642.Contig2 | | | | | | | | | |  |  |  |  |
| M | 0021955 | central nervous system neuron axonogenesis | EN-124k-90-group1642.Contig6 | | | | | | | | | |  |  |  |  |
| M | 0021955 | central nervous system neuron axonogenesis | EN-124k-90-group1642.Contig7 | | | | | | | | | |  |  |  |  |
| M | 0021955 | central nervous system neuron axonogenesis | EN-124k-90-group1669.Contig1 | | | | | | | | | |  |  |  |  |
| M | 0021955 | central nervous system neuron axonogenesis | EN-124k-90-group1728.Contig2 | | | | | | | | | |  |  |  |  |
| M | 0021955 | central nervous system neuron axonogenesis | EN-124k-90-group1735.Contig6 | | | | | | | | | |  |  |  |  |
| M | 0021955 | central nervous system neuron axonogenesis | EN-124k-90-group1752.Contig1 | | | | | | | | | |  |  |  |  |
| M | 0021955 | central nervous system neuron axonogenesis | EN-124k-90-group1752.Contig2 | | | | | | | | | |  |  |  |  |
| M | 0021955 | central nervous system neuron axonogenesis | EN-124k-90-group1752.Contig3 | | | | | | | | | |  |  |  |  |
| M | 0021955 | central nervous system neuron axonogenesis | EN-124k-90-group1752.Contig4 | | | | | | | | | |  |  |  |  |
| M | 0021955 | central nervous system neuron axonogenesis | EN-124k-90-group1752.Contig5 | | | | | | | | | |  |  |  |  |
| M | 0021955 | central nervous system neuron axonogenesis | EN-124k-90-group1752.Contig6 | | | | | | | | | |  |  |  |  |
| M | 0021955 | central nervous system neuron axonogenesis | EN-124k-90-group1782.Contig1 | | | | | | | | | |  |  |  |  |
| M | 0021955 | central nervous system neuron axonogenesis | EN-124k-90-group1873.Contig1 | | | | | | | | | |  |  |  |  |
| M | 0021955 | central nervous system neuron axonogenesis | EN-124k-90-group1960.Contig1 | | | | | | | | | |  |  |  |  |
| M | 0021955 | central nervous system neuron axonogenesis | EN-124k-90-group2075.Contig2 | | | | | | | | | |  |  |  |  |
| M | 0021955 | central nervous system neuron axonogenesis | EN-124k-90-group2081.Contig1 | | | | | | | | | |  |  |  |  |
| M | 0021955 | central nervous system neuron axonogenesis | EN-124k-90-group2081.Contig3 | | | | | | | | | |  |  |  |  |
| M | 0021955 | central nervous system neuron axonogenesis | EN-124k-90-group2097.Contig1 | | | | | | | | | |  |  |  |  |
| M | 0021955 | central nervous system neuron axonogenesis | EN-124k-90-group2163.Contig1 | | | | | | | | | |  |  |  |  |
| M | 0021955 | central nervous system neuron axonogenesis | EN-124k-90-group2200.Contig1 | | | | | | | | | |  |  |  |  |
| M | 0021955 | central nervous system neuron axonogenesis | EN-124k-90-group2264.Contig1 | | | | | | | | | |  |  |  |  |
| M | 0021955 | central nervous system neuron axonogenesis | EN-124k-90-group2491.Contig1 | | | | | | | | | |  |  |  |  |
| M | 0021955 | central nervous system neuron axonogenesis | EN-124k-90-group2806.Contig1 | | | | | | | | | |  |  |  |  |
| M | 0021955 | central nervous system neuron axonogenesis | EN-124k-90-group2917.Contig1 | | | | | | | | | |  |  |  |  |
| M | 0021955 | central nervous system neuron axonogenesis | EN-124k-90-group2917.Contig2 | | | | | | | | | |  |  |  |  |
| M | 0021955 | central nervous system neuron axonogenesis | EN-124k-90-group2917.Contig3 | | | | | | | | | |  |  |  |  |
| M | 0021955 | central nervous system neuron axonogenesis | EN-124k-90-group2917.Contig4 | | | | | | | | | |  |  |  |  |
| M | 0021955 | central nervous system neuron axonogenesis | EN-124k-90-group3082.Contig2 | | | | | | | | | |  |  |  |  |
| M | 0021955 | central nervous system neuron axonogenesis | EN-124k-90-group3223.Contig2 | | | | | | | | | |  |  |  |  |
| M | 0021955 | central nervous system neuron axonogenesis | EN-124k-90-group3394.Contig1 | | | | | | | | | |  |  |  |  |
| M | 0021955 | central nervous system neuron axonogenesis | EN-124k-90-group3394.Contig2 | | | | | | | | | |  |  |  |  |
| M | 0021955 | central nervous system neuron axonogenesis | EN-124k-90-group3394.Contig3 | | | | | | | | | |  |  |  |  |
| M | 0021955 | central nervous system neuron axonogenesis | EN-124k-90-group3394.Contig4 | | | | | | | | | |  |  |  |  |
| M | 0021955 | central nervous system neuron axonogenesis | EN-124k-90-group3479.Contig1 | | | | | | | | | |  |  |  |  |
| M | 0021955 | central nervous system neuron axonogenesis | EN-124k-90-group3503.Contig2 | | | | | | | | | |  |  |  |  |
| M | 0021955 | central nervous system neuron axonogenesis | EN-124k-90-group3503.Contig5 | | | | | | | | | |  |  |  |  |
| M | 0021955 | central nervous system neuron axonogenesis | EN-124k-90-group3509.Contig1 | | | | | | | | | |  |  |  |  |
| M | 0021955 | central nervous system neuron axonogenesis | EN-124k-90-group3606.Contig2 | | | | | | | | | |  |  |  |  |
| M | 0021955 | central nervous system neuron axonogenesis | EN-124k-90-group3700.Contig1 | | | | | | | | | |  |  |  |  |
| M | 0021955 | central nervous system neuron axonogenesis | EN-124k-90-group3849.Contig3 | | | | | | | | | |  |  |  |  |
| M | 0021955 | central nervous system neuron axonogenesis | EN-124k-90-group3933.Contig1 | | | | | | | | | |  |  |  |  |
| M | 0021955 | central nervous system neuron axonogenesis | EN-124k-90-group3966.Contig1 | | | | | | | | | |  |  |  |  |
| M | 0021955 | central nervous system neuron axonogenesis | EN-124k-90-group3966.Contig2 | | | | | | | | | |  |  |  |  |
| M | 0021955 | central nervous system neuron axonogenesis | EN-124k-90-group4090.Contig1 | | | | | | | | | |  |  |  |  |
| M | 0021955 | central nervous system neuron axonogenesis | EN-124k-90-group4410.Contig1 | | | | | | | | | |  |  |  |  |
| M | 0021955 | central nervous system neuron axonogenesis | EN-124k-90-group4562.Contig1 | | | | | | | | | |  |  |  |  |
| M | 0021955 | central nervous system neuron axonogenesis | EN-124k-90-group4701.Contig1 | | | | | | | | | |  |  |  |  |
| M | 0021955 | central nervous system neuron axonogenesis | EN-124k-90-group4701.Contig2 | | | | | | | | | |  |  |  |  |
| M | 0021955 | central nervous system neuron axonogenesis | EN-124k-90-group4701.Contig3 | | | | | | | | | |  |  |  |  |
| M | 0021955 | central nervous system neuron axonogenesis | EN-124k-90-group4753.Contig1 | | | | | | | | | |  |  |  |  |
| M | 0021955 | central nervous system neuron axonogenesis | EN-124k-90-group4929.Contig2 | | | | | | | | | |  |  |  |  |
| M | 0021955 | central nervous system neuron axonogenesis | EN-124k-90-group4944.Contig2 | | | | | | | | | |  |  |  |  |
| M | 0021955 | central nervous system neuron axonogenesis | EN-124k-90-group4989.Contig2 | | | | | | | | | |  |  |  |  |
| M | 0021955 | central nervous system neuron axonogenesis | EN-124k-90-group5172.Contig1 | | | | | | | | | |  |  |  |  |
| M | 0021955 | central nervous system neuron axonogenesis | EN-124k-90-group5173.Contig1 | | | | | | | | | |  |  |  |  |
| M | 0021955 | central nervous system neuron axonogenesis | EN-124k-90-group5184.Contig1 | | | | | | | | | |  |  |  |  |
| M | 0021955 | central nervous system neuron axonogenesis | EN-124k-90-group5273.Contig3 | | | | | | | | | |  |  |  |  |
| M | 0021955 | central nervous system neuron axonogenesis | EN-124k-90-group5293.Contig1 | | | | | | | | | |  |  |  |  |
| M | 0021955 | central nervous system neuron axonogenesis | EN-124k-90-group5380.Contig1 | | | | | | | | | |  |  |  |  |
| M | 0021955 | central nervous system neuron axonogenesis | EN-124k-90-group5479.Contig1 | | | | | | | | | |  |  |  |  |
| M | 0021955 | central nervous system neuron axonogenesis | EN-124k-90-group5524.Contig1 | | | | | | | | | |  |  |  |  |
| M | 0021955 | central nervous system neuron axonogenesis | EN-124k-90-group5996.Contig1 | | | | | | | | | |  |  |  |  |
| M | 0021955 | central nervous system neuron axonogenesis | EN-124k-90-group5996.Contig2 | | | | | | | | | |  |  |  |  |
| M | 0021955 | central nervous system neuron axonogenesis | EN-124k-90-group6038.Contig1 | | | | | | | | | |  |  |  |  |
| M | 0021955 | central nervous system neuron axonogenesis | EN-124k-90-group6112.Contig1 | | | | | | | | | |  |  |  |  |
| M | 0021955 | central nervous system neuron axonogenesis | EN-124k-90-group6204.Contig2 | | | | | | | | | |  |  |  |  |
| M | 0021955 | central nervous system neuron axonogenesis | EN-124k-90-group6358.Contig2 | | | | | | | | | |  |  |  |  |
| M | 0021955 | central nervous system neuron axonogenesis | EN-124k-90-group6666.Contig1 | | | | | | | | | |  |  |  |  |
| M | 0021955 | central nervous system neuron axonogenesis | EN-124k-90-group6719.Contig2 | | | | | | | | | |  |  |  |  |
| M | 0021955 | central nervous system neuron axonogenesis | EN-124k-90-group6923.Contig1 | | | | | | | | | |  |  |  |  |
| M | 0021955 | central nervous system neuron axonogenesis | EN-124k-90-group6923.Contig2 | | | | | | | | | |  |  |  |  |
| M | 0021955 | central nervous system neuron axonogenesis | EN-124k-90-group7149.Contig2 | | | | | | | | | |  |  |  |  |
| M | 0021955 | central nervous system neuron axonogenesis | EN-124k-90-group7157.Contig2 | | | | | | | | | |  |  |  |  |
| M | 0021955 | central nervous system neuron axonogenesis | EN-124k-90-group7158.Contig1 | | | | | | | | | |  |  |  |  |
| M | 0021955 | central nervous system neuron axonogenesis | EN-124k-90-group7231.Contig1 | | | | | | | | | |  |  |  |  |
| M | 0021955 | central nervous system neuron axonogenesis | EN-124k-90-group7343.Contig1 | | | | | | | | | |  |  |  |  |
| M | 0021955 | central nervous system neuron axonogenesis | EN-124k-90-group7528.Contig1 | | | | | | | | | |  |  |  |  |
| M | 0021955 | central nervous system neuron axonogenesis | EN-124k-90-group7749.Contig1 | | | | | | | | | |  |  |  |  |
| M | 0021955 | central nervous system neuron axonogenesis | EN-124k-90-group7785.Contig1 | | | | | | | | | |  |  |  |  |
| M | 0021955 | central nervous system neuron axonogenesis | EN-124k-90-group8079.Contig1 | | | | | | | | | |  |  |  |  |
| M | 0021955 | central nervous system neuron axonogenesis | EN-124k-90-group8079.Contig2 | | | | | | | | | |  |  |  |  |
| E | 0021955 | central nervous system neuron axonogenesis | EN-124k-90-group8101.Contig1 | | | | | | | | | |  |  |  |  |
| M | 0021955 | central nervous system neuron axonogenesis | EN-124k-90-group8850.Contig1 | | | | | | | | | |  |  |  |  |
| M | 0021955 | central nervous system neuron axonogenesis | EN-124k-90-group8937.Contig2 | | | | | | | | | |  |  |  |  |
| M | 0021955 | central nervous system neuron axonogenesis | EN-124k-90-group9295.Contig1 | | | | | | | | | |  |  |  |  |
| A | 0021955 | central nervous system neuron axonogenesis | EN-124k-90-group9295.Contig2 | | | | | | | | | |  |  |  |  |
| M | 0021955 | central nervous system neuron axonogenesis | EN-124k-90-group9317.Contig1 | | | | | | | | | |  |  |  |  |
| M | 0021955 | central nervous system neuron axonogenesis | EN-124k-90-group9446.Contig1 | | | | | | | | | |  |  |  |  |
| M | 0021955 | central nervous system neuron axonogenesis | EN-124k-90-group9479.Contig1 | | | | | | | | | |  |  |  |  |
| M | 0021955 | central nervous system neuron axonogenesis | EN-124k-90-group9747.Contig1 | | | | | | | | | |  |  |  |  |
| M | 0021955 | central nervous system neuron axonogenesis | EN-124k-90-group9747.Contig2 | | | | | | | | | |  |  |  |  |
| M | 0021955 | central nervous system neuron axonogenesis | EN-124k-90-group9794.Contig2 | | | | | | | | | |  |  |  |  |
| M | 0021955 | central nervous system neuron axonogenesis | EN-124k-90-group10085.Contig1 | | | | | | | | | |  |  |  |  |
| M | 0021955 | central nervous system neuron axonogenesis | EN-124k-90-group10085.Contig2 | | | | | | | | | |  |  |  |  |
| M | 0021955 | central nervous system neuron axonogenesis | EN-124k-90-group10221.Contig1 | | | | | | | | | |  |  |  |  |
| M | 0021955 | central nervous system neuron axonogenesis | EN-124k-90-group10598.Contig1 | | | | | | | | | |  |  |  |  |
| M | 0021955 | central nervous system neuron axonogenesis | EN-124k-90-group10630.Contig1 | | | | | | | | | |  |  |  |  |
| M | 0021955 | central nervous system neuron axonogenesis | EN-124k-90-group10630.Contig2 | | | | | | | | | |  |  |  |  |
| M | 0021955 | central nervous system neuron axonogenesis | EN-124k-90-group10650.Contig1 | | | | | | | | | |  |  |  |  |
| M | 0021955 | central nervous system neuron axonogenesis | EN-124k-90-group10772.Contig3 | | | | | | | | | |  |  |  |  |
| M | 0021955 | central nervous system neuron axonogenesis | EN-124k-90-group10838.Contig2 | | | | | | | | | |  |  |  |  |
| M | 0021955 | central nervous system neuron axonogenesis | EN-124k-90-group10912.Contig1 | | | | | | | | | |  |  |  |  |
| M | 0021955 | central nervous system neuron axonogenesis | EN-124k-90-group10928.Contig1 | | | | | | | | | |  |  |  |  |
| M | 0021955 | central nervous system neuron axonogenesis | EN-124k-90-group11228.Contig1 | | | | | | | | | |  |  |  |  |
| M | 0021955 | central nervous system neuron axonogenesis | EN-124k-90-group11235.Contig1 | | | | | | | | | |  |  |  |  |
| M | 0021955 | central nervous system neuron axonogenesis | EN-124k-90-group11408.Contig1 | | | | | | | | | |  |  |  |  |
| M | 0021955 | central nervous system neuron axonogenesis | EN-124k-90-group11516.Contig1 | | | | | | | | | |  |  |  |  |
| M | 0021955 | central nervous system neuron axonogenesis | EN-124k-90-group11516.Contig2 | | | | | | | | | |  |  |  |  |
| M | 0021955 | central nervous system neuron axonogenesis | EN-124k-90-group11808.Contig1 | | | | | | | | | |  |  |  |  |
| M | 0021955 | central nervous system neuron axonogenesis | EN-124k-90-group12099.Contig1 | | | | | | | | | |  |  |  |  |
| M | 0021955 | central nervous system neuron axonogenesis | EN-124k-90-group13235.Contig1 | | | | | | | | | |  |  |  |  |
| E | 0021955 | central nervous system neuron axonogenesis | EN-124k-90-group13456.Contig1 | | | | | | | | | |  |  |  |  |
| M | 0021955 | central nervous system neuron axonogenesis | EN-124k-90-group14027.Contig1 | | | | | | | | | |  |  |  |  |
| M | 0021955 | central nervous system neuron axonogenesis | EN-124k-90-group14708.Contig1 | | | | | | | | | |  |  |  |  |
| M | 0021955 | central nervous system neuron axonogenesis | EN-124k-90-group14975.Contig1 | | | | | | | | | |  |  |  |  |
| M | 0021955 | central nervous system neuron axonogenesis | EN-124k-90-group14975.Contig2 | | | | | | | | | |  |  |  |  |
| A | 0021955 | central nervous system neuron axonogenesis | EN-124k-90-group230.gs\_25885 | | | | | | | | | |  |  |  |  |
| E | 0021955 | central nervous system neuron axonogenesis | EN-124k-90-group359.jgi\_contig\_JGI\_CBBP16242\_fwd | | | | | | | | | | | | |  |
| E | 0021955 | central nervous system neuron axonogenesis | EN-124k-90-group359.EN\_iowa\_14996 | | | | | | | | | | |  |  |  |
| E | 0021955 | central nervous system neuron axonogenesis | EN-124k-90-group359.EN\_iowa\_1876 | | | | | | | | | | |  |  |  |
| E | 0021955 | central nervous system neuron axonogenesis | EN-124k-90-group359.EN\_iowa\_13183 | | | | | | | | | | |  |  |  |
| A | 0021955 | central nervous system neuron axonogenesis | EN-124k-90-group359.gs\_11387 | | | | | | | | | |  |  |  |  |
| A | 0021955 | central nervous system neuron axonogenesis | EN-124k-90-group359.gs\_45042 | | | | | | | | | |  |  |  |  |
| A | 0021955 | central nervous system neuron axonogenesis | EN-124k-90-group359.gs\_60113 | | | | | | | | | |  |  |  |  |
| E | 0021955 | central nervous system neuron axonogenesis | EN-124k-90-group900.EN\_iowa\_15436 | | | | | | | | | | |  |  |  |
| A | 0021955 | central nervous system neuron axonogenesis | EN-124k-90-group900.gs\_31783 | | | | | | | | | |  |  |  |  |
| A | 0021955 | central nervous system neuron axonogenesis | EN-124k-90-group900.gs\_71447 | | | | | | | | | |  |  |  |  |
| E | 0021955 | central nervous system neuron axonogenesis | EN-124k-90-group939.jgi\_contig\_JGI\_CBBP15138\_fwd | | | | | | | | | | | | |  |
| A | 0021955 | central nervous system neuron axonogenesis | EN-124k-90-group1642.gs\_68829 | | | | | | | | | |  |  |  |  |
| A | 0021955 | central nervous system neuron axonogenesis | EN-124k-90-group1642.gs\_49728 | | | | | | | | | |  |  |  |  |
| E | 0021955 | central nervous system neuron axonogenesis | EN-124k-90-group1642.jgi\_contig\_JGI\_CBBP16332\_fwd | | | | | | | | | | | | |  |
| A | 0021955 | central nervous system neuron axonogenesis | EN-124k-90-group1642.gs\_63459 | | | | | | | | | |  |  |  |  |
| E | 0021955 | central nervous system neuron axonogenesis | EN-124k-90-group1642.jgi\_contig\_JGI\_CBBP16017\_fwd | | | | | | | | | | | | |  |
| A | 0021955 | central nervous system neuron axonogenesis | EN-124k-90-group1642.gs\_32797 | | | | | | | | | |  |  |  |  |
| E | 0021955 | central nervous system neuron axonogenesis | EN-124k-90-group1642.EN\_iowa\_1848 | | | | | | | | | | |  |  |  |
| A | 0021955 | central nervous system neuron axonogenesis | EN-124k-90-group1782.gs\_16663 | | | | | | | | | |  |  |  |  |
| E | 0021955 | central nervous system neuron axonogenesis | EN-124k-90-group2163.jgi\_contig\_JGI\_CBBP7370\_fwd | | | | | | | | | | | | |  |
| A | 0021955 | central nervous system neuron axonogenesis | EN-124k-90-group3394.gs\_42372 | | | | | | | | | |  |  |  |  |
| E | 0021955 | central nervous system neuron axonogenesis | EN-124k-90-group3966.jgi\_contig\_JGI\_CBBP12693\_fwd | | | | | | | | | | | | |  |
| E | 0021955 | central nervous system neuron axonogenesis | EN-124k-90-group3966.jgi\_contig\_JGI\_CBBP7455\_fwd | | | | | | | | | | | | |  |
| E | 0021955 | central nervous system neuron axonogenesis | EN-124k-90-group3966.EN\_iowa\_15266 | | | | | | | | | | |  |  |  |
| E | 0021955 | central nervous system neuron axonogenesis | EN-124k-90-group6923.jgi\_contig\_JGI\_CBBP17789\_fwd | | | | | | | | | | | | |  |
| E | 0021954 | central nervous system neuron development | EN-124k-90-group343.jgi\_contig\_JGI\_CBBP19299\_fwd | | | | | | | | | | | | |  |
| A | 0021954 | central nervous system neuron development | EN-124k-90-group807.gs\_71281 | | | | | | | | | |  |  |  |  |
| E | 0021954 | central nervous system neuron development | EN-124k-90-group1680.jgi\_contig\_JGI\_CBBP17232\_fwd | | | | | | | | | | | | |  |
| E | 0021954 | central nervous system neuron development | EN-124k-90-group1852.jgi\_paired\_JGI\_CBBP10940\_fwd | | | | | | | | | | | | |  |
| E | 0021954 | central nervous system neuron development | EN-124k-90-group2530.jgi\_paired\_JGI\_CBBP5537\_fwd | | | | | | | | | | | | |  |
| A | 0021954 | central nervous system neuron development | EN-124k-90-group3420.gs\_43284 | | | | | | | | | |  |  |  |  |
| E | 0021954 | central nervous system neuron development | EN-124k-90-group5117.jgi\_paired\_JGI\_CBBP19153\_fwd | | | | | | | | | | | | |  |
| A | 0021954 | central nervous system neuron development | EN-124k-90-group5346.gs\_72008 | | | | | | | | | |  |  |  |  |
| E | 0021954 | central nervous system neuron development | EN-124k-90-group5558.jgi\_contig\_JGI\_CBBP4325\_fwd | | | | | | | | | | | | |  |
| E | 0021954 | central nervous system neuron development | EN-124k-90-group5834.jgi\_contig\_JGI\_CBBP12701\_fwd | | | | | | | | | | | | |  |
| E | 0021954 | central nervous system neuron development | EN-124k-90-group6048.EN\_iowa\_911 | | | | | | | | | | |  |  |  |
| E | 0021954 | central nervous system neuron development | EN-124k-90-group7594.jgi\_paired\_JGI\_CBBP17768\_fwd | | | | | | | | | | | | |  |
| A | 0021954 | central nervous system neuron development | EN-124k-90-group7595.gs\_36261 | | | | | | | | | |  |  |  |  |
| E | 0021954 | central nervous system neuron development | EN-124k-90-group7598.jgi\_unpaired\_JGI\_CBBP18343\_fwd | | | | | | | | | | | | | |
| A | 0021954 | central nervous system neuron development | EN-124k-90-group8023.gs\_82870 | | | | | | | | | |  |  |  |  |
| E | 0021954 | central nervous system neuron development | EN-124k-90-group8107.jgi\_paired\_JGI\_CBBP6197\_fwd | | | | | | | | | | | | |  |
| A | 0021954 | central nervous system neuron development | EN-124k-90-group8310.gs\_24913 | | | | | | | | | |  |  |  |  |
| A | 0021954 | central nervous system neuron development | EN-124k-90-group9714.gs\_64750 | | | | | | | | | |  |  |  |  |
| E | 0021954 | central nervous system neuron development | EN-124k-90-group9989.EN\_iowa\_3399 | | | | | | | | | | |  |  |  |
| E | 0021954 | central nervous system neuron development | EN-124k-90-group10304.jgi\_paired\_JGI\_CBBP11045\_fwd | | | | | | | | | | | | |  |
| A | 0021954 | central nervous system neuron development | EN-124k-90-group10312.gs\_22624 | | | | | | | | | |  |  |  |  |
| E | 0021954 | central nervous system neuron development | EN-124k-90-group10416.jgi\_contig\_JGI\_CBBP3274\_fwd | | | | | | | | | | | | |  |
| A | 0021954 | central nervous system neuron development | EN-124k-90-group10692.gs\_44993 | | | | | | | | | |  |  |  |  |
| A | 0021954 | central nervous system neuron development | EN-124k-90-group10718.gs\_26673 | | | | | | | | | |  |  |  |  |
| A | 0021954 | central nervous system neuron development | EN-124k-90-group11354.gs\_78099 | | | | | | | | | |  |  |  |  |
| E | 0021954 | central nervous system neuron development | EN-124k-90-group11438.jgi\_paired\_JGI\_CBBP9709\_fwd | | | | | | | | | | | | |  |
| E | 0021954 | central nervous system neuron development | EN-124k-90-group11605.jgi\_paired\_JGI\_CBBP17453\_rev | | | | | | | | | | | | |  |
| A | 0021954 | central nervous system neuron development | EN-124k-90-group11738.gs\_53113 | | | | | | | | | |  |  |  |  |
| E | 0021954 | central nervous system neuron development | EN-124k-90-group12452.EN\_iowa\_9722 | | | | | | | | | | |  |  |  |
| E | 0021954 | central nervous system neuron development | EN-124k-90-group12595.jgi\_contig\_JGI\_CBBP15955\_fwd | | | | | | | | | | | | |  |
| E | 0021954 | central nervous system neuron development | EN-124k-90-group13011.jgi\_paired\_JGI\_CBBP19322\_fwd | | | | | | | | | | | | |  |
| A | 0021954 | central nervous system neuron development | EN-124k-90-group13823.gs\_32410 | | | | | | | | | |  |  |  |  |
| E | 0021954 | central nervous system neuron development | EN-124k-90-group13911.jgi\_contig\_JGI\_CBBP10628\_fwd | | | | | | | | | | | | |  |
| A | 0021954 | central nervous system neuron development | EN-124k-90-group13938.gs\_32571 | | | | | | | | | |  |  |  |  |
| A | 0021954 | central nervous system neuron development | EN-124k-90-group14563.gs\_48486 | | | | | | | | | |  |  |  |  |
| E | 0021954 | central nervous system neuron development | EN-124k-90-group14931.jgi\_paired\_JGI\_CBBP10444\_fwd | | | | | | | | | | | | |  |
| A | 0021954 | central nervous system neuron development | EN-124k-90-group15056.gs\_73097 | | | | | | | | | |  |  |  |  |
| A | 0021954 | central nervous system neuron development | EN-124k-90-group15229.gs\_75551 | | | | | | | | | |  |  |  |  |
| E | 0021954 | central nervous system neuron development | EN-124k-90-group15289.jgi\_paired\_JGI\_CBBP6226\_fwd | | | | | | | | | | | | |  |
| E | 0021954 | central nervous system neuron development | EN-124k-90-group15290.EN\_iowa\_9903 | | | | | | | | | | |  |  |  |
| E | 0021954 | central nervous system neuron development | EN-124k-90-group15532.EN\_iowa\_5883 | | | | | | | | | | |  |  |  |
| A | 0021954 | central nervous system neuron development | EN-124k-90-group15770.gs\_45569 | | | | | | | | | |  |  |  |  |
| A | 0021954 | central nervous system neuron development | EN-124k-90-group15856.gs\_15241 | | | | | | | | | |  |  |  |  |
| E | 0021954 | central nervous system neuron development | EN-124k-90-group16034.jgi\_paired\_JGI\_CBBP10733\_fwd | | | | | | | | | | | | |  |
| E | 0021954 | central nervous system neuron development | EN-124k-90-group16143.jgi\_paired\_JGI\_CBBP4408\_fwd | | | | | | | | | | | | |  |
| E | 0021954 | central nervous system neuron development | EN-124k-90-group16262.jgi\_paired\_JGI\_CBBP4191\_fwd | | | | | | | | | | | | |  |
| A | 0021954 | central nervous system neuron development | EN-124k-90-group16285.gs\_47129 | | | | | | | | | |  |  |  |  |
| A | 0021954 | central nervous system neuron development | EN-124k-90-group16439.gs\_54729 | | | | | | | | | |  |  |  |  |
| E | 0021954 | central nervous system neuron development | EN-124k-90-group16705.jgi\_paired\_JGI\_CBBP19935\_rev | | | | | | | | | | | | |  |
| A | 0021954 | central nervous system neuron development | EN-124k-90-group16837.gs\_15971 | | | | | | | | | |  |  |  |  |
| A | 0021954 | central nervous system neuron development | EN-124k-90-group16910.gs\_22871 | | | | | | | | | |  |  |  |  |
| A | 0021954 | central nervous system neuron development | EN-124k-90-group16929.gs\_43290 | | | | | | | | | |  |  |  |  |
| E | 0021954 | central nervous system neuron development | EN-124k-90-group17086.jgi\_paired\_JGI\_CBBP6417\_rev | | | | | | | | | | | | |  |
| E | 0021954 | central nervous system neuron development | EN-124k-90-group17091.jgi\_paired\_JGI\_CBBP10179\_rev | | | | | | | | | | | | |  |
| A | 0021954 | central nervous system neuron development | EN-124k-90-group17442.gs\_63748 | | | | | | | | | |  |  |  |  |
| A | 0021954 | central nervous system neuron development | EN-124k-90-group17524.gs\_80802 | | | | | | | | | |  |  |  |  |
| A | 0021954 | central nervous system neuron development | EN-124k-90-group17556.gs\_87114 | | | | | | | | | |  |  |  |  |
| A | 0021954 | central nervous system neuron development | EN-124k-90-group17563.gs\_80410 | | | | | | | | | |  |  |  |  |
| A | 0021954 | central nervous system neuron development | EN-124k-90-group17930.gs\_83513 | | | | | | | | | |  |  |  |  |
| A | 0021954 | central nervous system neuron development | EN-124k-90-group18038.gs\_30262 | | | | | | | | | |  |  |  |  |
| M | 0021954 | central nervous system neuron development | EN-124k-90-group13.Contig4 | | | | | | | | |  |  |  |  |  |
| M | 0021954 | central nervous system neuron development | EN-124k-90-group70.Contig6 | | | | | | | | |  |  |  |  |  |
| M | 0021954 | central nervous system neuron development | EN-124k-90-group138.Contig1 | | | | | | | | | |  |  |  |  |
| M | 0021954 | central nervous system neuron development | EN-124k-90-group138.Contig62 | | | | | | | | | |  |  |  |  |
| M | 0021954 | central nervous system neuron development | EN-124k-90-group229.Contig1 | | | | | | | | | |  |  |  |  |
| M | 0021954 | central nervous system neuron development | EN-124k-90-group230.Contig1 | | | | | | | | | |  |  |  |  |
| M | 0021954 | central nervous system neuron development | EN-124k-90-group324.Contig1 | | | | | | | | | |  |  |  |  |
| M | 0021954 | central nervous system neuron development | EN-124k-90-group478.Contig1 | | | | | | | | | |  |  |  |  |
| M | 0021954 | central nervous system neuron development | EN-124k-90-group481.Contig4 | | | | | | | | | |  |  |  |  |
| M | 0021954 | central nervous system neuron development | EN-124k-90-group550.Contig1 | | | | | | | | | |  |  |  |  |
| M | 0021954 | central nervous system neuron development | EN-124k-90-group550.Contig2 | | | | | | | | | |  |  |  |  |
| M | 0021954 | central nervous system neuron development | EN-124k-90-group593.Contig1 | | | | | | | | | |  |  |  |  |
| M | 0021954 | central nervous system neuron development | EN-124k-90-group630.Contig2 | | | | | | | | | |  |  |  |  |
| M | 0021954 | central nervous system neuron development | EN-124k-90-group658.Contig1 | | | | | | | | | |  |  |  |  |
| M | 0021954 | central nervous system neuron development | EN-124k-90-group658.Contig3 | | | | | | | | | |  |  |  |  |
| M | 0021954 | central nervous system neuron development | EN-124k-90-group682.Contig1 | | | | | | | | | |  |  |  |  |
| M | 0021954 | central nervous system neuron development | EN-124k-90-group900.Contig1 | | | | | | | | | |  |  |  |  |
| M | 0021954 | central nervous system neuron development | EN-124k-90-group1167.Contig3 | | | | | | | | | |  |  |  |  |
| M | 0021954 | central nervous system neuron development | EN-124k-90-group1266.Contig1 | | | | | | | | | |  |  |  |  |
| M | 0021954 | central nervous system neuron development | EN-124k-90-group1266.Contig2 | | | | | | | | | |  |  |  |  |
| M | 0021954 | central nervous system neuron development | EN-124k-90-group1266.Contig3 | | | | | | | | | |  |  |  |  |
| M | 0021954 | central nervous system neuron development | EN-124k-90-group1297.Contig1 | | | | | | | | | |  |  |  |  |
| M | 0021954 | central nervous system neuron development | EN-124k-90-group1304.Contig2 | | | | | | | | | |  |  |  |  |
| M | 0021954 | central nervous system neuron development | EN-124k-90-group1304.Contig4 | | | | | | | | | |  |  |  |  |
| M | 0021954 | central nervous system neuron development | EN-124k-90-group1304.Contig8 | | | | | | | | | |  |  |  |  |
| M | 0021954 | central nervous system neuron development | EN-124k-90-group1423.Contig14 | | | | | | | | | |  |  |  |  |
| M | 0021954 | central nervous system neuron development | EN-124k-90-group1456.Contig4 | | | | | | | | | |  |  |  |  |
| M | 0021954 | central nervous system neuron development | EN-124k-90-group1456.Contig8 | | | | | | | | | |  |  |  |  |
| A | 0021954 | central nervous system neuron development | EN-124k-90-group1482.Contig1 | | | | | | | | | |  |  |  |  |
| M | 0021954 | central nervous system neuron development | EN-124k-90-group1735.Contig6 | | | | | | | | | |  |  |  |  |
| M | 0021954 | central nervous system neuron development | EN-124k-90-group1915.Contig1 | | | | | | | | | |  |  |  |  |
| M | 0021954 | central nervous system neuron development | EN-124k-90-group1960.Contig1 | | | | | | | | | |  |  |  |  |
| M | 0021954 | central nervous system neuron development | EN-124k-90-group2004.Contig3 | | | | | | | | | |  |  |  |  |
| M | 0021954 | central nervous system neuron development | EN-124k-90-group2074.Contig1 | | | | | | | | | |  |  |  |  |
| M | 0021954 | central nervous system neuron development | EN-124k-90-group2081.Contig1 | | | | | | | | | |  |  |  |  |
| M | 0021954 | central nervous system neuron development | EN-124k-90-group2081.Contig3 | | | | | | | | | |  |  |  |  |
| M | 0021954 | central nervous system neuron development | EN-124k-90-group2409.Contig3 | | | | | | | | | |  |  |  |  |
| M | 0021954 | central nervous system neuron development | EN-124k-90-group2428.Contig1 | | | | | | | | | |  |  |  |  |
| M | 0021954 | central nervous system neuron development | EN-124k-90-group2455.Contig1 | | | | | | | | | |  |  |  |  |
| M | 0021954 | central nervous system neuron development | EN-124k-90-group2582.Contig1 | | | | | | | | | |  |  |  |  |
| M | 0021954 | central nervous system neuron development | EN-124k-90-group2607.Contig1 | | | | | | | | | |  |  |  |  |
| M | 0021954 | central nervous system neuron development | EN-124k-90-group2607.Contig2 | | | | | | | | | |  |  |  |  |
| M | 0021954 | central nervous system neuron development | EN-124k-90-group2607.Contig7 | | | | | | | | | |  |  |  |  |
| M | 0021954 | central nervous system neuron development | EN-124k-90-group2681.Contig1 | | | | | | | | | |  |  |  |  |
| M | 0021954 | central nervous system neuron development | EN-124k-90-group2811.Contig1 | | | | | | | | | |  |  |  |  |
| M | 0021954 | central nervous system neuron development | EN-124k-90-group2811.Contig2 | | | | | | | | | |  |  |  |  |
| M | 0021954 | central nervous system neuron development | EN-124k-90-group2829.Contig2 | | | | | | | | | |  |  |  |  |
| M | 0021954 | central nervous system neuron development | EN-124k-90-group2846.Contig1 | | | | | | | | | |  |  |  |  |
| M | 0021954 | central nervous system neuron development | EN-124k-90-group2866.Contig1 | | | | | | | | | |  |  |  |  |
| M | 0021954 | central nervous system neuron development | EN-124k-90-group2940.Contig2 | | | | | | | | | |  |  |  |  |
| M | 0021954 | central nervous system neuron development | EN-124k-90-group3058.Contig1 | | | | | | | | | |  |  |  |  |
| M | 0021954 | central nervous system neuron development | EN-124k-90-group3082.Contig2 | | | | | | | | | |  |  |  |  |
| M | 0021954 | central nervous system neuron development | EN-124k-90-group3299.Contig1 | | | | | | | | | |  |  |  |  |
| M | 0021954 | central nervous system neuron development | EN-124k-90-group3313.Contig1 | | | | | | | | | |  |  |  |  |
| M | 0021954 | central nervous system neuron development | EN-124k-90-group3313.Contig2 | | | | | | | | | |  |  |  |  |
| M | 0021954 | central nervous system neuron development | EN-124k-90-group3313.Contig3 | | | | | | | | | |  |  |  |  |
| M | 0021954 | central nervous system neuron development | EN-124k-90-group3315.Contig6 | | | | | | | | | |  |  |  |  |
| M | 0021954 | central nervous system neuron development | EN-124k-90-group3316.Contig1 | | | | | | | | | |  |  |  |  |
| M | 0021954 | central nervous system neuron development | EN-124k-90-group3479.Contig1 | | | | | | | | | |  |  |  |  |
| M | 0021954 | central nervous system neuron development | EN-124k-90-group3586.Contig1 | | | | | | | | | |  |  |  |  |
| M | 0021954 | central nervous system neuron development | EN-124k-90-group3606.Contig2 | | | | | | | | | |  |  |  |  |
| M | 0021954 | central nervous system neuron development | EN-124k-90-group3615.Contig4 | | | | | | | | | |  |  |  |  |
| M | 0021954 | central nervous system neuron development | EN-124k-90-group3620.Contig3 | | | | | | | | | |  |  |  |  |
| M | 0021954 | central nervous system neuron development | EN-124k-90-group3661.Contig1 | | | | | | | | | |  |  |  |  |
| M | 0021954 | central nervous system neuron development | EN-124k-90-group3897.Contig1 | | | | | | | | | |  |  |  |  |
| M | 0021954 | central nervous system neuron development | EN-124k-90-group4183.Contig1 | | | | | | | | | |  |  |  |  |
| M | 0021954 | central nervous system neuron development | EN-124k-90-group4339.Contig1 | | | | | | | | | |  |  |  |  |
| M | 0021954 | central nervous system neuron development | EN-124k-90-group4374.Contig1 | | | | | | | | | |  |  |  |  |
| M | 0021954 | central nervous system neuron development | EN-124k-90-group4583.Contig4 | | | | | | | | | |  |  |  |  |
| M | 0021954 | central nervous system neuron development | EN-124k-90-group4753.Contig1 | | | | | | | | | |  |  |  |  |
| M | 0021954 | central nervous system neuron development | EN-124k-90-group4803.Contig1 | | | | | | | | | |  |  |  |  |
| M | 0021954 | central nervous system neuron development | EN-124k-90-group4803.Contig2 | | | | | | | | | |  |  |  |  |
| M | 0021954 | central nervous system neuron development | EN-124k-90-group4929.Contig2 | | | | | | | | | |  |  |  |  |
| M | 0021954 | central nervous system neuron development | EN-124k-90-group5173.Contig1 | | | | | | | | | |  |  |  |  |
| M | 0021954 | central nervous system neuron development | EN-124k-90-group5203.Contig2 | | | | | | | | | |  |  |  |  |
| M | 0021954 | central nervous system neuron development | EN-124k-90-group5252.Contig1 | | | | | | | | | |  |  |  |  |
| M | 0021954 | central nervous system neuron development | EN-124k-90-group5273.Contig2 | | | | | | | | | |  |  |  |  |
| M | 0021954 | central nervous system neuron development | EN-124k-90-group5411.Contig1 | | | | | | | | | |  |  |  |  |
| M | 0021954 | central nervous system neuron development | EN-124k-90-group5471.Contig3 | | | | | | | | | |  |  |  |  |
| M | 0021954 | central nervous system neuron development | EN-124k-90-group5491.Contig1 | | | | | | | | | |  |  |  |  |
| M | 0021954 | central nervous system neuron development | EN-124k-90-group5491.Contig2 | | | | | | | | | |  |  |  |  |
| M | 0021954 | central nervous system neuron development | EN-124k-90-group5527.Contig2 | | | | | | | | | |  |  |  |  |
| M | 0021954 | central nervous system neuron development | EN-124k-90-group5542.Contig1 | | | | | | | | | |  |  |  |  |
| A | 0021954 | central nervous system neuron development | EN-124k-90-group5543.Contig1 | | | | | | | | | |  |  |  |  |
| M | 0021954 | central nervous system neuron development | EN-124k-90-group5607.Contig1 | | | | | | | | | |  |  |  |  |
| M | 0021954 | central nervous system neuron development | EN-124k-90-group6044.Contig2 | | | | | | | | | |  |  |  |  |
| M | 0021954 | central nervous system neuron development | EN-124k-90-group6204.Contig1 | | | | | | | | | |  |  |  |  |
| M | 0021954 | central nervous system neuron development | EN-124k-90-group6204.Contig2 | | | | | | | | | |  |  |  |  |
| M | 0021954 | central nervous system neuron development | EN-124k-90-group6400.Contig1 | | | | | | | | | |  |  |  |  |
| M | 0021954 | central nervous system neuron development | EN-124k-90-group6472.Contig1 | | | | | | | | | |  |  |  |  |
| M | 0021954 | central nervous system neuron development | EN-124k-90-group6472.Contig2 | | | | | | | | | |  |  |  |  |
| M | 0021954 | central nervous system neuron development | EN-124k-90-group6666.Contig1 | | | | | | | | | |  |  |  |  |
| M | 0021954 | central nervous system neuron development | EN-124k-90-group6923.Contig1 | | | | | | | | | |  |  |  |  |
| M | 0021954 | central nervous system neuron development | EN-124k-90-group6923.Contig2 | | | | | | | | | |  |  |  |  |
| M | 0021954 | central nervous system neuron development | EN-124k-90-group7149.Contig2 | | | | | | | | | |  |  |  |  |
| M | 0021954 | central nervous system neuron development | EN-124k-90-group7231.Contig1 | | | | | | | | | |  |  |  |  |
| M | 0021954 | central nervous system neuron development | EN-124k-90-group7343.Contig1 | | | | | | | | | |  |  |  |  |
| M | 0021954 | central nervous system neuron development | EN-124k-90-group7382.Contig2 | | | | | | | | | |  |  |  |  |
| M | 0021954 | central nervous system neuron development | EN-124k-90-group7528.Contig1 | | | | | | | | | |  |  |  |  |
| M | 0021954 | central nervous system neuron development | EN-124k-90-group7787.Contig1 | | | | | | | | | |  |  |  |  |
| M | 0021954 | central nervous system neuron development | EN-124k-90-group7787.Contig2 | | | | | | | | | |  |  |  |  |
| M | 0021954 | central nervous system neuron development | EN-124k-90-group7787.Contig3 | | | | | | | | | |  |  |  |  |
| M | 0021954 | central nervous system neuron development | EN-124k-90-group7866.Contig1 | | | | | | | | | |  |  |  |  |
| M | 0021954 | central nervous system neuron development | EN-124k-90-group7915.Contig1 | | | | | | | | | |  |  |  |  |
| M | 0021954 | central nervous system neuron development | EN-124k-90-group8079.Contig1 | | | | | | | | | |  |  |  |  |
| M | 0021954 | central nervous system neuron development | EN-124k-90-group8079.Contig2 | | | | | | | | | |  |  |  |  |
| M | 0021954 | central nervous system neuron development | EN-124k-90-group8418.Contig1 | | | | | | | | | |  |  |  |  |
| M | 0021954 | central nervous system neuron development | EN-124k-90-group8492.Contig1 | | | | | | | | | |  |  |  |  |
| A | 0021954 | central nervous system neuron development | EN-124k-90-group8565.Contig1 | | | | | | | | | |  |  |  |  |
| M | 0021954 | central nervous system neuron development | EN-124k-90-group8799.Contig2 | | | | | | | | | |  |  |  |  |
| M | 0021954 | central nervous system neuron development | EN-124k-90-group8850.Contig1 | | | | | | | | | |  |  |  |  |
| M | 0021954 | central nervous system neuron development | EN-124k-90-group9079.Contig1 | | | | | | | | | |  |  |  |  |
| M | 0021954 | central nervous system neuron development | EN-124k-90-group9299.Contig3 | | | | | | | | | |  |  |  |  |
| M | 0021954 | central nervous system neuron development | EN-124k-90-group9388.Contig1 | | | | | | | | | |  |  |  |  |
| M | 0021954 | central nervous system neuron development | EN-124k-90-group9388.Contig2 | | | | | | | | | |  |  |  |  |
| M | 0021954 | central nervous system neuron development | EN-124k-90-group9446.Contig1 | | | | | | | | | |  |  |  |  |
| M | 0021954 | central nervous system neuron development | EN-124k-90-group9819.Contig1 | | | | | | | | | |  |  |  |  |
| M | 0021954 | central nervous system neuron development | EN-124k-90-group9843.Contig1 | | | | | | | | | |  |  |  |  |
| M | 0021954 | central nervous system neuron development | EN-124k-90-group10085.Contig1 | | | | | | | | | |  |  |  |  |
| M | 0021954 | central nervous system neuron development | EN-124k-90-group10085.Contig2 | | | | | | | | | |  |  |  |  |
| M | 0021954 | central nervous system neuron development | EN-124k-90-group10221.Contig1 | | | | | | | | | |  |  |  |  |
| M | 0021954 | central nervous system neuron development | EN-124k-90-group10467.Contig1 | | | | | | | | | |  |  |  |  |
| M | 0021954 | central nervous system neuron development | EN-124k-90-group10637.Contig1 | | | | | | | | | |  |  |  |  |
| M | 0021954 | central nervous system neuron development | EN-124k-90-group10877.Contig1 | | | | | | | | | |  |  |  |  |
| M | 0021954 | central nervous system neuron development | EN-124k-90-group10912.Contig1 | | | | | | | | | |  |  |  |  |
| M | 0021954 | central nervous system neuron development | EN-124k-90-group10928.Contig1 | | | | | | | | | |  |  |  |  |
| M | 0021954 | central nervous system neuron development | EN-124k-90-group11235.Contig1 | | | | | | | | | |  |  |  |  |
| M | 0021954 | central nervous system neuron development | EN-124k-90-group11399.Contig2 | | | | | | | | | |  |  |  |  |
| M | 0021954 | central nervous system neuron development | EN-124k-90-group12099.Contig1 | | | | | | | | | |  |  |  |  |
| M | 0021954 | central nervous system neuron development | EN-124k-90-group12115.Contig1 | | | | | | | | | |  |  |  |  |
| M | 0021954 | central nervous system neuron development | EN-124k-90-group12519.Contig1 | | | | | | | | | |  |  |  |  |
| M | 0021954 | central nervous system neuron development | EN-124k-90-group12638.Contig1 | | | | | | | | | |  |  |  |  |
| M | 0021954 | central nervous system neuron development | EN-124k-90-group12973.Contig1 | | | | | | | | | |  |  |  |  |
| E | 0021954 | central nervous system neuron development | EN-124k-90-group13069.Contig2 | | | | | | | | | |  |  |  |  |
| M | 0021954 | central nervous system neuron development | EN-124k-90-group13235.Contig1 | | | | | | | | | |  |  |  |  |
| A | 0021954 | central nervous system neuron development | EN-124k-90-group14199.Contig1 | | | | | | | | | |  |  |  |  |
| M | 0021954 | central nervous system neuron development | EN-124k-90-group14514.Contig1 | | | | | | | | | |  |  |  |  |
| M | 0021954 | central nervous system neuron development | EN-124k-90-group14708.Contig1 | | | | | | | | | |  |  |  |  |
| A | 0021954 | central nervous system neuron development | EN-124k-90-group230.gs\_25885 | | | | | | | | | |  |  |  |  |
| E | 0021954 | central nervous system neuron development | EN-124k-90-group478.jgi\_contig\_JGI\_CBBP10824\_fwd | | | | | | | | | | | | |  |
| A | 0021954 | central nervous system neuron development | EN-124k-90-group1423.gs\_11172 | | | | | | | | | |  |  |  |  |
| A | 0021954 | central nervous system neuron development | EN-124k-90-group1423.gs\_52362 | | | | | | | | | |  |  |  |  |
| A | 0021954 | central nervous system neuron development | EN-124k-90-group1423.gs\_75717 | | | | | | | | | |  |  |  |  |
| E | 0021954 | central nervous system neuron development | EN-124k-90-group3058.jgi\_contig\_JGI\_CBBP9922\_fwd | | | | | | | | | | | | |  |
| A | 0021954 | central nervous system neuron development | EN-124k-90-group3615.gs\_46165 | | | | | | | | | |  |  |  |  |
| A | 0021954 | central nervous system neuron development | EN-124k-90-group3615.gs\_63719 | | | | | | | | | |  |  |  |  |
| A | 0021954 | central nervous system neuron development | EN-124k-90-group5203.gs\_53868 | | | | | | | | | |  |  |  |  |
| A | 0021954 | central nervous system neuron development | EN-124k-90-group5203.gs\_41715 | | | | | | | | | |  |  |  |  |
| A | 0021954 | central nervous system neuron development | EN-124k-90-group5203.gs\_19246 | | | | | | | | | |  |  |  |  |
| A | 0021954 | central nervous system neuron development | EN-124k-90-group5491.gs\_7631 | | | | | | | | | |  |  |  |  |
| A | 0021954 | central nervous system neuron development | EN-124k-90-group5491.gs\_75129 | | | | | | | | | |  |  |  |  |
| E | 0021954 | central nervous system neuron development | EN-124k-90-group5607.jgi\_contig\_JGI\_CBBP445\_fwd | | | | | | | | | | | | |  |
| E | 0021954 | central nervous system neuron development | EN-124k-90-group6204.EN\_iowa\_1799 | | | | | | | | | | |  |  |  |
| E | 0021954 | central nervous system neuron development | EN-124k-90-group6204.EN\_iowa\_4716 | | | | | | | | | | |  |  |  |
| E | 0021954 | central nervous system neuron development | EN-124k-90-group6472.jgi\_contig\_JGI\_CBBP2402\_fwd | | | | | | | | | | | | |  |
| E | 0021954 | central nervous system neuron development | EN-124k-90-group6923.jgi\_contig\_JGI\_CBBP17789\_fwd | | | | | | | | | | | | |  |
| E | 0021953 | central nervous system neuron differentiation | EN-124k-90-group3265.jgi\_paired\_JGI\_CBBP17018\_rev | | | | | | | | | | | | |  |
| A | 0021953 | central nervous system neuron differentiation | EN-124k-90-group3564.gs\_15152 | | | | | | | | | |  |  |  |  |
| A | 0021953 | central nervous system neuron differentiation | EN-124k-90-group7595.gs\_36261 | | | | | | | | | |  |  |  |  |
| A | 0021953 | central nervous system neuron differentiation | EN-124k-90-group7985.gs\_22681 | | | | | | | | | |  |  |  |  |
| E | 0021953 | central nervous system neuron differentiation | EN-124k-90-group9064.EN\_iowa\_5147 | | | | | | | | | | |  |  |  |
| E | 0021953 | central nervous system neuron differentiation | EN-124k-90-group9773.jgi\_paired\_JGI\_CBBP6585\_rev | | | | | | | | | | | | |  |
| E | 0021953 | central nervous system neuron differentiation | EN-124k-90-group10304.jgi\_paired\_JGI\_CBBP11045\_fwd | | | | | | | | | | | | |  |
| E | 0021953 | central nervous system neuron differentiation | EN-124k-90-group10416.jgi\_contig\_JGI\_CBBP3274\_fwd | | | | | | | | | | | | |  |
| E | 0021953 | central nervous system neuron differentiation | EN-124k-90-group11043.EN\_iowa\_1645 | | | | | | | | | | |  |  |  |
| E | 0021953 | central nervous system neuron differentiation | EN-124k-90-group11438.jgi\_paired\_JGI\_CBBP9709\_fwd | | | | | | | | | | | | |  |
| E | 0021953 | central nervous system neuron differentiation | EN-124k-90-group11545.jgi\_paired\_JGI\_CBBP2808\_fwd | | | | | | | | | | | | |  |
| A | 0021953 | central nervous system neuron differentiation | EN-124k-90-group13938.gs\_32571 | | | | | | | | | |  |  |  |  |
| A | 0021953 | central nervous system neuron differentiation | EN-124k-90-group14128.gs\_38861 | | | | | | | | | |  |  |  |  |
| E | 0021953 | central nervous system neuron differentiation | EN-124k-90-group14544.jgi\_contig\_JGI\_CBBP14322\_fwd | | | | | | | | | | | | |  |
| A | 0021953 | central nervous system neuron differentiation | EN-124k-90-group14558.gs\_28342 | | | | | | | | | |  |  |  |  |
| E | 0021953 | central nervous system neuron differentiation | EN-124k-90-group15289.jgi\_paired\_JGI\_CBBP6226\_fwd | | | | | | | | | | | | |  |
| E | 0021953 | central nervous system neuron differentiation | EN-124k-90-group15532.EN\_iowa\_5883 | | | | | | | | | | |  |  |  |
| A | 0021953 | central nervous system neuron differentiation | EN-124k-90-group15856.gs\_15241 | | | | | | | | | |  |  |  |  |
| E | 0021953 | central nervous system neuron differentiation | EN-124k-90-group15944.jgi\_paired\_JGI\_CBBP5778\_fwd | | | | | | | | | | | | |  |
| A | 0021953 | central nervous system neuron differentiation | EN-124k-90-group16439.gs\_54729 | | | | | | | | | |  |  |  |  |
| A | 0021953 | central nervous system neuron differentiation | EN-124k-90-group16443.gs\_57863 | | | | | | | | | |  |  |  |  |
| A | 0021953 | central nervous system neuron differentiation | EN-124k-90-group16489.gs\_80464 | | | | | | | | | |  |  |  |  |
| A | 0021953 | central nervous system neuron differentiation | EN-124k-90-group16523.gs\_75670 | | | | | | | | | |  |  |  |  |
| A | 0021953 | central nervous system neuron differentiation | EN-124k-90-group16575.gs\_6 | | | | | | | | | |  |  |  |  |
| A | 0021953 | central nervous system neuron differentiation | EN-124k-90-group16636.gs\_429 | | | | | | | | | |  |  |  |  |
| A | 0021953 | central nervous system neuron differentiation | EN-124k-90-group16714.gs\_320 | | | | | | | | | |  |  |  |  |
| A | 0021953 | central nervous system neuron differentiation | EN-124k-90-group16830.gs\_82512 | | | | | | | | | |  |  |  |  |
| A | 0021953 | central nervous system neuron differentiation | EN-124k-90-group16872.gs\_213 | | | | | | | | | |  |  |  |  |
| A | 0021953 | central nervous system neuron differentiation | EN-124k-90-group16910.gs\_22871 | | | | | | | | | |  |  |  |  |
| A | 0021953 | central nervous system neuron differentiation | EN-124k-90-group16929.gs\_43290 | | | | | | | | | |  |  |  |  |
| A | 0021953 | central nervous system neuron differentiation | EN-124k-90-group17276.gs\_80679 | | | | | | | | | |  |  |  |  |
| A | 0021953 | central nervous system neuron differentiation | EN-124k-90-group17471.gs\_85794 | | | | | | | | | |  |  |  |  |
| A | 0021953 | central nervous system neuron differentiation | EN-124k-90-group17572.gs\_7187 | | | | | | | | | |  |  |  |  |
| A | 0021953 | central nervous system neuron differentiation | EN-124k-90-group17582.gs\_70636 | | | | | | | | | |  |  |  |  |
| A | 0021953 | central nervous system neuron differentiation | EN-124k-90-group17650.gs\_59359 | | | | | | | | | |  |  |  |  |
| A | 0021953 | central nervous system neuron differentiation | EN-124k-90-group17702.gs\_18827 | | | | | | | | | |  |  |  |  |
| A | 0021953 | central nervous system neuron differentiation | EN-124k-90-group17744.gs\_30255 | | | | | | | | | |  |  |  |  |
| A | 0021953 | central nervous system neuron differentiation | EN-124k-90-group18140.gs\_87168 | | | | | | | | | |  |  |  |  |
| A | 0021953 | central nervous system neuron differentiation | EN-124k-90-group18342.gs\_65612 | | | | | | | | | |  |  |  |  |
| A | 0021953 | central nervous system neuron differentiation | EN-124k-90-group18480.gs\_449 | | | | | | | | | |  |  |  |  |
| A | 0021953 | central nervous system neuron differentiation | EN-124k-90-group18774.gs\_57540 | | | | | | | | | |  |  |  |  |
| M | 0021953 | central nervous system neuron differentiation | EN-124k-90-group16.Contig1 | | | | | | | | |  |  |  |  |  |
| M | 0021953 | central nervous system neuron differentiation | EN-124k-90-group16.Contig2 | | | | | | | | |  |  |  |  |  |
| M | 0021953 | central nervous system neuron differentiation | EN-124k-90-group478.Contig1 | | | | | | | | | |  |  |  |  |
| M | 0021953 | central nervous system neuron differentiation | EN-124k-90-group727.Contig29 | | | | | | | | | |  |  |  |  |
| M | 0021953 | central nervous system neuron differentiation | EN-124k-90-group945.Contig1 | | | | | | | | | |  |  |  |  |
| M | 0021953 | central nervous system neuron differentiation | EN-124k-90-group998.Contig1 | | | | | | | | | |  |  |  |  |
| M | 0021953 | central nervous system neuron differentiation | EN-124k-90-group1153.Contig1 | | | | | | | | | |  |  |  |  |
| M | 0021953 | central nervous system neuron differentiation | EN-124k-90-group1161.Contig1 | | | | | | | | | |  |  |  |  |
| M | 0021953 | central nervous system neuron differentiation | EN-124k-90-group1161.Contig2 | | | | | | | | | |  |  |  |  |
| M | 0021953 | central nervous system neuron differentiation | EN-124k-90-group1272.Contig1 | | | | | | | | | |  |  |  |  |
| M | 0021953 | central nervous system neuron differentiation | EN-124k-90-group1348.Contig1 | | | | | | | | | |  |  |  |  |
| M | 0021953 | central nervous system neuron differentiation | EN-124k-90-group1351.Contig1 | | | | | | | | | |  |  |  |  |
| M | 0021953 | central nervous system neuron differentiation | EN-124k-90-group1423.Contig14 | | | | | | | | | |  |  |  |  |
| M | 0021953 | central nervous system neuron differentiation | EN-124k-90-group1423.Contig20 | | | | | | | | | |  |  |  |  |
| M | 0021953 | central nervous system neuron differentiation | EN-124k-90-group2537.Contig1 | | | | | | | | | |  |  |  |  |
| M | 0021953 | central nervous system neuron differentiation | EN-124k-90-group3058.Contig1 | | | | | | | | | |  |  |  |  |
| M | 0021953 | central nervous system neuron differentiation | EN-124k-90-group3176.Contig2 | | | | | | | | | |  |  |  |  |
| M | 0021953 | central nervous system neuron differentiation | EN-124k-90-group3238.Contig1 | | | | | | | | | |  |  |  |  |
| M | 0021953 | central nervous system neuron differentiation | EN-124k-90-group3408.Contig1 | | | | | | | | | |  |  |  |  |
| M | 0021953 | central nervous system neuron differentiation | EN-124k-90-group3408.Contig2 | | | | | | | | | |  |  |  |  |
| M | 0021953 | central nervous system neuron differentiation | EN-124k-90-group3520.Contig1 | | | | | | | | | |  |  |  |  |
| M | 0021953 | central nervous system neuron differentiation | EN-124k-90-group3522.Contig1 | | | | | | | | | |  |  |  |  |
| M | 0021953 | central nervous system neuron differentiation | EN-124k-90-group3577.Contig1 | | | | | | | | | |  |  |  |  |
| M | 0021953 | central nervous system neuron differentiation | EN-124k-90-group3577.Contig2 | | | | | | | | | |  |  |  |  |
| M | 0021953 | central nervous system neuron differentiation | EN-124k-90-group3586.Contig1 | | | | | | | | | |  |  |  |  |
| M | 0021953 | central nervous system neuron differentiation | EN-124k-90-group3713.Contig1 | | | | | | | | | |  |  |  |  |
| M | 0021953 | central nervous system neuron differentiation | EN-124k-90-group4462.Contig1 | | | | | | | | | |  |  |  |  |
| M | 0021953 | central nervous system neuron differentiation | EN-124k-90-group4668.Contig1 | | | | | | | | | |  |  |  |  |
| M | 0021953 | central nervous system neuron differentiation | EN-124k-90-group4763.Contig1 | | | | | | | | | |  |  |  |  |
| M | 0021953 | central nervous system neuron differentiation | EN-124k-90-group5063.Contig1 | | | | | | | | | |  |  |  |  |
| A | 0021953 | central nervous system neuron differentiation | EN-124k-90-group5155.Contig1 | | | | | | | | | |  |  |  |  |
| M | 0021953 | central nervous system neuron differentiation | EN-124k-90-group5252.Contig1 | | | | | | | | | |  |  |  |  |
| M | 0021953 | central nervous system neuron differentiation | EN-124k-90-group5298.Contig1 | | | | | | | | | |  |  |  |  |
| M | 0021953 | central nervous system neuron differentiation | EN-124k-90-group5471.Contig3 | | | | | | | | | |  |  |  |  |
| M | 0021953 | central nervous system neuron differentiation | EN-124k-90-group5491.Contig1 | | | | | | | | | |  |  |  |  |
| M | 0021953 | central nervous system neuron differentiation | EN-124k-90-group5491.Contig2 | | | | | | | | | |  |  |  |  |
| M | 0021953 | central nervous system neuron differentiation | EN-124k-90-group5527.Contig2 | | | | | | | | | |  |  |  |  |
| M | 0021953 | central nervous system neuron differentiation | EN-124k-90-group6033.Contig1 | | | | | | | | | |  |  |  |  |
| M | 0021953 | central nervous system neuron differentiation | EN-124k-90-group6033.Contig2 | | | | | | | | | |  |  |  |  |
| M | 0021953 | central nervous system neuron differentiation | EN-124k-90-group6033.Contig3 | | | | | | | | | |  |  |  |  |
| M | 0021953 | central nervous system neuron differentiation | EN-124k-90-group6809.Contig1 | | | | | | | | | |  |  |  |  |
| M | 0021953 | central nervous system neuron differentiation | EN-124k-90-group7165.Contig1 | | | | | | | | | |  |  |  |  |
| M | 0021953 | central nervous system neuron differentiation | EN-124k-90-group7234.Contig1 | | | | | | | | | |  |  |  |  |
| M | 0021953 | central nervous system neuron differentiation | EN-124k-90-group7440.Contig1 | | | | | | | | | |  |  |  |  |
| M | 0021953 | central nervous system neuron differentiation | EN-124k-90-group7457.Contig1 | | | | | | | | | |  |  |  |  |
| M | 0021953 | central nervous system neuron differentiation | EN-124k-90-group7915.Contig1 | | | | | | | | | |  |  |  |  |
| M | 0021953 | central nervous system neuron differentiation | EN-124k-90-group8325.Contig1 | | | | | | | | | |  |  |  |  |
| M | 0021953 | central nervous system neuron differentiation | EN-124k-90-group8418.Contig1 | | | | | | | | | |  |  |  |  |
| A | 0021953 | central nervous system neuron differentiation | EN-124k-90-group8519.Contig1 | | | | | | | | | |  |  |  |  |
| A | 0021953 | central nervous system neuron differentiation | EN-124k-90-group8565.Contig1 | | | | | | | | | |  |  |  |  |
| M | 0021953 | central nervous system neuron differentiation | EN-124k-90-group8568.Contig1 | | | | | | | | | |  |  |  |  |
| M | 0021953 | central nervous system neuron differentiation | EN-124k-90-group8645.Contig1 | | | | | | | | | |  |  |  |  |
| A | 0021953 | central nervous system neuron differentiation | EN-124k-90-group8957.Contig1 | | | | | | | | | |  |  |  |  |
| A | 0021953 | central nervous system neuron differentiation | EN-124k-90-group9245.Contig1 | | | | | | | | | |  |  |  |  |
| M | 0021953 | central nervous system neuron differentiation | EN-124k-90-group9661.Contig1 | | | | | | | | | |  |  |  |  |
| M | 0021953 | central nervous system neuron differentiation | EN-124k-90-group10652.Contig1 | | | | | | | | | |  |  |  |  |
| M | 0021953 | central nervous system neuron differentiation | EN-124k-90-group10790.Contig1 | | | | | | | | | |  |  |  |  |
| M | 0021953 | central nervous system neuron differentiation | EN-124k-90-group10843.Contig1 | | | | | | | | | |  |  |  |  |
| M | 0021953 | central nervous system neuron differentiation | EN-124k-90-group11209.Contig1 | | | | | | | | | |  |  |  |  |
| M | 0021953 | central nervous system neuron differentiation | EN-124k-90-group11399.Contig2 | | | | | | | | | |  |  |  |  |
| A | 0021953 | central nervous system neuron differentiation | EN-124k-90-group12616.Contig1 | | | | | | | | | |  |  |  |  |
| M | 0021953 | central nervous system neuron differentiation | EN-124k-90-group12638.Contig1 | | | | | | | | | |  |  |  |  |
| E | 0021953 | central nervous system neuron differentiation | EN-124k-90-group13069.Contig2 | | | | | | | | | |  |  |  |  |
| M | 0021953 | central nervous system neuron differentiation | EN-124k-90-group13121.Contig1 | | | | | | | | | |  |  |  |  |
| M | 0021953 | central nervous system neuron differentiation | EN-124k-90-group13502.Contig1 | | | | | | | | | |  |  |  |  |
| M | 0021953 | central nervous system neuron differentiation | EN-124k-90-group13502.Contig2 | | | | | | | | | |  |  |  |  |
| A | 0021953 | central nervous system neuron differentiation | EN-124k-90-group14199.Contig1 | | | | | | | | | |  |  |  |  |
| E | 0021953 | central nervous system neuron differentiation | EN-124k-90-group478.jgi\_contig\_JGI\_CBBP10824\_fwd | | | | | | | | | | | | |  |
| A | 0021953 | central nervous system neuron differentiation | EN-124k-90-group1153.gs\_635 | | | | | | | | | |  |  |  |  |
| A | 0021953 | central nervous system neuron differentiation | EN-124k-90-group1153.gs\_33239 | | | | | | | | | |  |  |  |  |
| A | 0021953 | central nervous system neuron differentiation | EN-124k-90-group1423.gs\_16014 | | | | | | | | | |  |  |  |  |
| A | 0021953 | central nervous system neuron differentiation | EN-124k-90-group1423.gs\_11172 | | | | | | | | | |  |  |  |  |
| A | 0021953 | central nervous system neuron differentiation | EN-124k-90-group1423.gs\_52362 | | | | | | | | | |  |  |  |  |
| A | 0021953 | central nervous system neuron differentiation | EN-124k-90-group1423.gs\_75717 | | | | | | | | | |  |  |  |  |
| E | 0021953 | central nervous system neuron differentiation | EN-124k-90-group3058.jgi\_contig\_JGI\_CBBP9922\_fwd | | | | | | | | | | | | |  |
| A | 0021953 | central nervous system neuron differentiation | EN-124k-90-group3238.gs\_87325 | | | | | | | | | |  |  |  |  |
| A | 0021953 | central nervous system neuron differentiation | EN-124k-90-group3522.gs\_9768 | | | | | | | | | |  |  |  |  |
| A | 0021953 | central nervous system neuron differentiation | EN-124k-90-group3713.gs\_7861 | | | | | | | | | |  |  |  |  |
| A | 0021953 | central nervous system neuron differentiation | EN-124k-90-group5491.gs\_7631 | | | | | | | | | |  |  |  |  |
| A | 0021953 | central nervous system neuron differentiation | EN-124k-90-group5491.gs\_75129 | | | | | | | | | |  |  |  |  |
| A | 0021953 | central nervous system neuron differentiation | EN-124k-90-group7440.gs\_75782 | | | | | | | | | |  |  |  |  |
| A | 0021952 | central nervous system projection neuron axonogenesis | EN-124k-90-group807.gs\_71281 | | | | | | | | | |  |  |  |  |
| E | 0021952 | central nervous system projection neuron axonogenesis | EN-124k-90-group1306.EN\_iowa\_2354 | | | | | | | | | | |  |  |  |
| E | 0021952 | central nervous system projection neuron axonogenesis | EN-124k-90-group2530.jgi\_paired\_JGI\_CBBP5537\_fwd | | | | | | | | | | | | |  |
| E | 0021952 | central nervous system projection neuron axonogenesis | EN-124k-90-group5117.jgi\_paired\_JGI\_CBBP19153\_fwd | | | | | | | | | | | | |  |
| E | 0021952 | central nervous system projection neuron axonogenesis | EN-124k-90-group5741.jgi\_paired\_JGI\_CBBP19133\_fwd | | | | | | | | | | | | |  |
| E | 0021952 | central nervous system projection neuron axonogenesis | EN-124k-90-group5834.jgi\_contig\_JGI\_CBBP12701\_fwd | | | | | | | | | | | | |  |
| E | 0021952 | central nervous system projection neuron axonogenesis | EN-124k-90-group5954.jgi\_paired\_JGI\_CBBP7330\_fwd | | | | | | | | | | | | |  |
| E | 0021952 | central nervous system projection neuron axonogenesis | EN-124k-90-group6737.jgi\_contig\_JGI\_CBBP17820\_fwd | | | | | | | | | | | | |  |
| E | 0021952 | central nervous system projection neuron axonogenesis | EN-124k-90-group7594.jgi\_paired\_JGI\_CBBP17768\_fwd | | | | | | | | | | | | |  |
| A | 0021952 | central nervous system projection neuron axonogenesis | EN-124k-90-group8310.gs\_24913 | | | | | | | | | |  |  |  |  |
| A | 0021952 | central nervous system projection neuron axonogenesis | EN-124k-90-group8658.gs\_22315 | | | | | | | | | |  |  |  |  |
| A | 0021952 | central nervous system projection neuron axonogenesis | EN-124k-90-group9467.gs\_16039 | | | | | | | | | |  |  |  |  |
| A | 0021952 | central nervous system projection neuron axonogenesis | EN-124k-90-group9714.gs\_64750 | | | | | | | | | |  |  |  |  |
| E | 0021952 | central nervous system projection neuron axonogenesis | EN-124k-90-group9942.jgi\_paired\_JGI\_CBBP11089\_fwd | | | | | | | | | | | | |  |
| E | 0021952 | central nervous system projection neuron axonogenesis | EN-124k-90-group9989.EN\_iowa\_3399 | | | | | | | | | | |  |  |  |
| E | 0021952 | central nervous system projection neuron axonogenesis | EN-124k-90-group10304.jgi\_paired\_JGI\_CBBP11045\_fwd | | | | | | | | | | | | |  |
| A | 0021952 | central nervous system projection neuron axonogenesis | EN-124k-90-group10312.gs\_22624 | | | | | | | | | |  |  |  |  |
| A | 0021952 | central nervous system projection neuron axonogenesis | EN-124k-90-group10935.gs\_64995 | | | | | | | | | |  |  |  |  |
| E | 0021952 | central nervous system projection neuron axonogenesis | EN-124k-90-group11438.jgi\_paired\_JGI\_CBBP9709\_fwd | | | | | | | | | | | | |  |
| E | 0021952 | central nervous system projection neuron axonogenesis | EN-124k-90-group11500.EN\_iowa\_7830 | | | | | | | | | | |  |  |  |
| A | 0021952 | central nervous system projection neuron axonogenesis | EN-124k-90-group11738.gs\_53113 | | | | | | | | | |  |  |  |  |
| E | 0021952 | central nervous system projection neuron axonogenesis | EN-124k-90-group12012.jgi\_contig\_JGI\_CBBP10480\_fwd | | | | | | | | | | | | |  |
| E | 0021952 | central nervous system projection neuron axonogenesis | EN-124k-90-group12191.jgi\_paired\_JGI\_CBBP10887\_fwd | | | | | | | | | | | | |  |
| E | 0021952 | central nervous system projection neuron axonogenesis | EN-124k-90-group12452.EN\_iowa\_9722 | | | | | | | | | | |  |  |  |
| E | 0021952 | central nervous system projection neuron axonogenesis | EN-124k-90-group13011.jgi\_paired\_JGI\_CBBP19322\_fwd | | | | | | | | | | | | |  |
| E | 0021952 | central nervous system projection neuron axonogenesis | EN-124k-90-group13366.jgi\_paired\_JGI\_CBBP16258\_fwd | | | | | | | | | | | | |  |
| E | 0021952 | central nervous system projection neuron axonogenesis | EN-124k-90-group13491.jgi\_paired\_JGI\_CBBP11089\_rev | | | | | | | | | | | | |  |
| A | 0021952 | central nervous system projection neuron axonogenesis | EN-124k-90-group13938.gs\_32571 | | | | | | | | | |  |  |  |  |
| E | 0021952 | central nervous system projection neuron axonogenesis | EN-124k-90-group14335.jgi\_paired\_JGI\_CBBP12938\_fwd | | | | | | | | | | | | |  |
| A | 0021952 | central nervous system projection neuron axonogenesis | EN-124k-90-group14545.gs\_14842 | | | | | | | | | |  |  |  |  |
| A | 0021952 | central nervous system projection neuron axonogenesis | EN-124k-90-group14563.gs\_48486 | | | | | | | | | |  |  |  |  |
| A | 0021952 | central nervous system projection neuron axonogenesis | EN-124k-90-group14948.gs\_26769 | | | | | | | | | |  |  |  |  |
| A | 0021952 | central nervous system projection neuron axonogenesis | EN-124k-90-group15056.gs\_73097 | | | | | | | | | |  |  |  |  |
| A | 0021952 | central nervous system projection neuron axonogenesis | EN-124k-90-group15198.gs\_65271 | | | | | | | | | |  |  |  |  |
| E | 0021952 | central nervous system projection neuron axonogenesis | EN-124k-90-group15223.EN\_iowa\_18438 | | | | | | | | | | |  |  |  |
| E | 0021952 | central nervous system projection neuron axonogenesis | EN-124k-90-group15290.EN\_iowa\_9903 | | | | | | | | | | |  |  |  |
| E | 0021952 | central nervous system projection neuron axonogenesis | EN-124k-90-group15320.jgi\_paired\_JGI\_CBBP9817\_fwd | | | | | | | | | | | | |  |
| A | 0021952 | central nervous system projection neuron axonogenesis | EN-124k-90-group15399.gs\_80457 | | | | | | | | | |  |  |  |  |
| A | 0021952 | central nervous system projection neuron axonogenesis | EN-124k-90-group15770.gs\_45569 | | | | | | | | | |  |  |  |  |
| E | 0021952 | central nervous system projection neuron axonogenesis | EN-124k-90-group15988.jgi\_unpaired\_JGI\_CBBP2255\_fwd | | | | | | | | | | | | | |
| A | 0021952 | central nervous system projection neuron axonogenesis | EN-124k-90-group16067.gs\_19008 | | | | | | | | | |  |  |  |  |
| E | 0021952 | central nervous system projection neuron axonogenesis | EN-124k-90-group16200.jgi\_paired\_JGI\_CBBP10444\_rev | | | | | | | | | | | | |  |
| A | 0021952 | central nervous system projection neuron axonogenesis | EN-124k-90-group16254.gs\_29060 | | | | | | | | | |  |  |  |  |
| A | 0021952 | central nervous system projection neuron axonogenesis | EN-124k-90-group16439.gs\_54729 | | | | | | | | | |  |  |  |  |
| A | 0021952 | central nervous system projection neuron axonogenesis | EN-124k-90-group16489.gs\_80464 | | | | | | | | | |  |  |  |  |
| A | 0021952 | central nervous system projection neuron axonogenesis | EN-124k-90-group16600.gs\_29512 | | | | | | | | | |  |  |  |  |
| A | 0021952 | central nervous system projection neuron axonogenesis | EN-124k-90-group16830.gs\_82512 | | | | | | | | | |  |  |  |  |
| A | 0021952 | central nervous system projection neuron axonogenesis | EN-124k-90-group16837.gs\_15971 | | | | | | | | | |  |  |  |  |
| A | 0021952 | central nervous system projection neuron axonogenesis | EN-124k-90-group16872.gs\_213 | | | | | | | | | |  |  |  |  |
| A | 0021952 | central nervous system projection neuron axonogenesis | EN-124k-90-group16929.gs\_43290 | | | | | | | | | |  |  |  |  |
| A | 0021952 | central nervous system projection neuron axonogenesis | EN-124k-90-group17276.gs\_80679 | | | | | | | | | |  |  |  |  |
| A | 0021952 | central nervous system projection neuron axonogenesis | EN-124k-90-group17360.gs\_72469 | | | | | | | | | |  |  |  |  |
| A | 0021952 | central nervous system projection neuron axonogenesis | EN-124k-90-group17487.gs\_82041 | | | | | | | | | |  |  |  |  |
| A | 0021952 | central nervous system projection neuron axonogenesis | EN-124k-90-group17572.gs\_7187 | | | | | | | | | |  |  |  |  |
| A | 0021952 | central nervous system projection neuron axonogenesis | EN-124k-90-group17582.gs\_70636 | | | | | | | | | |  |  |  |  |
| A | 0021952 | central nervous system projection neuron axonogenesis | EN-124k-90-group17706.gs\_80669 | | | | | | | | | |  |  |  |  |
| A | 0021952 | central nervous system projection neuron axonogenesis | EN-124k-90-group17776.gs\_56285 | | | | | | | | | |  |  |  |  |
| A | 0021952 | central nervous system projection neuron axonogenesis | EN-124k-90-group17892.gs\_49260 | | | | | | | | | |  |  |  |  |
| E | 0021952 | central nervous system projection neuron axonogenesis | EN-124k-90-group17979.jgi\_contig\_JGI\_CBBP2421\_fwd | | | | | | | | | | | | |  |
| A | 0021952 | central nervous system projection neuron axonogenesis | EN-124k-90-group18140.gs\_87168 | | | | | | | | | |  |  |  |  |
| E | 0021952 | central nervous system projection neuron axonogenesis | EN-124k-90-group18538.jgi\_paired\_JGI\_CBBP19133\_rev | | | | | | | | | | | | |  |
| A | 0021952 | central nervous system projection neuron axonogenesis | EN-124k-90-group18774.gs\_57540 | | | | | | | | | |  |  |  |  |
| A | 0021952 | central nervous system projection neuron axonogenesis | EN-124k-90-group78.Contig1 | | | | | | | | |  |  |  |  |  |
| A | 0021952 | central nervous system projection neuron axonogenesis | EN-124k-90-group78.Contig2 | | | | | | | | |  |  |  |  |  |
| M | 0021952 | central nervous system projection neuron axonogenesis | EN-124k-90-group230.Contig1 | | | | | | | | | |  |  |  |  |
| M | 0021952 | central nervous system projection neuron axonogenesis | EN-124k-90-group328.Contig2 | | | | | | | | | |  |  |  |  |
| M | 0021952 | central nervous system projection neuron axonogenesis | EN-124k-90-group359.Contig1 | | | | | | | | | |  |  |  |  |
| M | 0021952 | central nervous system projection neuron axonogenesis | EN-124k-90-group359.Contig2 | | | | | | | | | |  |  |  |  |
| M | 0021952 | central nervous system projection neuron axonogenesis | EN-124k-90-group359.Contig3 | | | | | | | | | |  |  |  |  |
| M | 0021952 | central nervous system projection neuron axonogenesis | EN-124k-90-group359.Contig4 | | | | | | | | | |  |  |  |  |
| M | 0021952 | central nervous system projection neuron axonogenesis | EN-124k-90-group359.Contig5 | | | | | | | | | |  |  |  |  |
| M | 0021952 | central nervous system projection neuron axonogenesis | EN-124k-90-group362.Contig2 | | | | | | | | | |  |  |  |  |
| M | 0021952 | central nervous system projection neuron axonogenesis | EN-124k-90-group481.Contig4 | | | | | | | | | |  |  |  |  |
| M | 0021952 | central nervous system projection neuron axonogenesis | EN-124k-90-group518.Contig2 | | | | | | | | | |  |  |  |  |
| M | 0021952 | central nervous system projection neuron axonogenesis | EN-124k-90-group550.Contig2 | | | | | | | | | |  |  |  |  |
| M | 0021952 | central nervous system projection neuron axonogenesis | EN-124k-90-group593.Contig1 | | | | | | | | | |  |  |  |  |
| M | 0021952 | central nervous system projection neuron axonogenesis | EN-124k-90-group630.Contig2 | | | | | | | | | |  |  |  |  |
| M | 0021952 | central nervous system projection neuron axonogenesis | EN-124k-90-group658.Contig1 | | | | | | | | | |  |  |  |  |
| M | 0021952 | central nervous system projection neuron axonogenesis | EN-124k-90-group658.Contig3 | | | | | | | | | |  |  |  |  |
| M | 0021952 | central nervous system projection neuron axonogenesis | EN-124k-90-group682.Contig1 | | | | | | | | | |  |  |  |  |
| M | 0021952 | central nervous system projection neuron axonogenesis | EN-124k-90-group735.Contig1 | | | | | | | | | |  |  |  |  |
| M | 0021952 | central nervous system projection neuron axonogenesis | EN-124k-90-group881.Contig1 | | | | | | | | | |  |  |  |  |
| M | 0021952 | central nervous system projection neuron axonogenesis | EN-124k-90-group881.Contig2 | | | | | | | | | |  |  |  |  |
| M | 0021952 | central nervous system projection neuron axonogenesis | EN-124k-90-group881.Contig3 | | | | | | | | | |  |  |  |  |
| M | 0021952 | central nervous system projection neuron axonogenesis | EN-124k-90-group900.Contig1 | | | | | | | | | |  |  |  |  |
| M | 0021952 | central nervous system projection neuron axonogenesis | EN-124k-90-group1034.Contig1 | | | | | | | | | |  |  |  |  |
| M | 0021952 | central nervous system projection neuron axonogenesis | EN-124k-90-group1071.Contig1 | | | | | | | | | |  |  |  |  |
| M | 0021952 | central nervous system projection neuron axonogenesis | EN-124k-90-group1071.Contig2 | | | | | | | | | |  |  |  |  |
| M | 0021952 | central nervous system projection neuron axonogenesis | EN-124k-90-group1071.Contig3 | | | | | | | | | |  |  |  |  |
| M | 0021952 | central nervous system projection neuron axonogenesis | EN-124k-90-group1106.Contig1 | | | | | | | | | |  |  |  |  |
| M | 0021952 | central nervous system projection neuron axonogenesis | EN-124k-90-group1167.Contig2 | | | | | | | | | |  |  |  |  |
| M | 0021952 | central nervous system projection neuron axonogenesis | EN-124k-90-group1167.Contig3 | | | | | | | | | |  |  |  |  |
| M | 0021952 | central nervous system projection neuron axonogenesis | EN-124k-90-group1304.Contig2 | | | | | | | | | |  |  |  |  |
| M | 0021952 | central nervous system projection neuron axonogenesis | EN-124k-90-group1304.Contig4 | | | | | | | | | |  |  |  |  |
| M | 0021952 | central nervous system projection neuron axonogenesis | EN-124k-90-group1304.Contig8 | | | | | | | | | |  |  |  |  |
| M | 0021952 | central nervous system projection neuron axonogenesis | EN-124k-90-group1401.Contig2 | | | | | | | | | |  |  |  |  |
| M | 0021952 | central nervous system projection neuron axonogenesis | EN-124k-90-group1456.Contig4 | | | | | | | | | |  |  |  |  |
| M | 0021952 | central nervous system projection neuron axonogenesis | EN-124k-90-group1456.Contig8 | | | | | | | | | |  |  |  |  |
| M | 0021952 | central nervous system projection neuron axonogenesis | EN-124k-90-group1619.Contig1 | | | | | | | | | |  |  |  |  |
| M | 0021952 | central nervous system projection neuron axonogenesis | EN-124k-90-group1642.Contig1 | | | | | | | | | |  |  |  |  |
| M | 0021952 | central nervous system projection neuron axonogenesis | EN-124k-90-group1642.Contig2 | | | | | | | | | |  |  |  |  |
| M | 0021952 | central nervous system projection neuron axonogenesis | EN-124k-90-group1642.Contig6 | | | | | | | | | |  |  |  |  |
| M | 0021952 | central nervous system projection neuron axonogenesis | EN-124k-90-group1642.Contig7 | | | | | | | | | |  |  |  |  |
| M | 0021952 | central nervous system projection neuron axonogenesis | EN-124k-90-group1728.Contig2 | | | | | | | | | |  |  |  |  |
| M | 0021952 | central nervous system projection neuron axonogenesis | EN-124k-90-group1735.Contig4 | | | | | | | | | |  |  |  |  |
| M | 0021952 | central nervous system projection neuron axonogenesis | EN-124k-90-group1735.Contig6 | | | | | | | | | |  |  |  |  |
| M | 0021952 | central nervous system projection neuron axonogenesis | EN-124k-90-group1782.Contig1 | | | | | | | | | |  |  |  |  |
| M | 0021952 | central nervous system projection neuron axonogenesis | EN-124k-90-group1915.Contig1 | | | | | | | | | |  |  |  |  |
| M | 0021952 | central nervous system projection neuron axonogenesis | EN-124k-90-group1960.Contig1 | | | | | | | | | |  |  |  |  |
| M | 0021952 | central nervous system projection neuron axonogenesis | EN-124k-90-group2075.Contig2 | | | | | | | | | |  |  |  |  |
| M | 0021952 | central nervous system projection neuron axonogenesis | EN-124k-90-group2081.Contig1 | | | | | | | | | |  |  |  |  |
| M | 0021952 | central nervous system projection neuron axonogenesis | EN-124k-90-group2081.Contig3 | | | | | | | | | |  |  |  |  |
| M | 0021952 | central nervous system projection neuron axonogenesis | EN-124k-90-group2097.Contig1 | | | | | | | | | |  |  |  |  |
| M | 0021952 | central nervous system projection neuron axonogenesis | EN-124k-90-group2155.Contig1 | | | | | | | | | |  |  |  |  |
| M | 0021952 | central nervous system projection neuron axonogenesis | EN-124k-90-group2200.Contig1 | | | | | | | | | |  |  |  |  |
| M | 0021952 | central nervous system projection neuron axonogenesis | EN-124k-90-group2449.Contig1 | | | | | | | | | |  |  |  |  |
| M | 0021952 | central nervous system projection neuron axonogenesis | EN-124k-90-group2449.Contig2 | | | | | | | | | |  |  |  |  |
| M | 0021952 | central nervous system projection neuron axonogenesis | EN-124k-90-group2498.Contig5 | | | | | | | | | |  |  |  |  |
| M | 0021952 | central nervous system projection neuron axonogenesis | EN-124k-90-group2498.Contig7 | | | | | | | | | |  |  |  |  |
| M | 0021952 | central nervous system projection neuron axonogenesis | EN-124k-90-group2606.Contig1 | | | | | | | | | |  |  |  |  |
| M | 0021952 | central nervous system projection neuron axonogenesis | EN-124k-90-group2778.Contig1 | | | | | | | | | |  |  |  |  |
| M | 0021952 | central nervous system projection neuron axonogenesis | EN-124k-90-group2846.Contig1 | | | | | | | | | |  |  |  |  |
| M | 0021952 | central nervous system projection neuron axonogenesis | EN-124k-90-group2917.Contig1 | | | | | | | | | |  |  |  |  |
| M | 0021952 | central nervous system projection neuron axonogenesis | EN-124k-90-group2917.Contig2 | | | | | | | | | |  |  |  |  |
| M | 0021952 | central nervous system projection neuron axonogenesis | EN-124k-90-group2917.Contig3 | | | | | | | | | |  |  |  |  |
| M | 0021952 | central nervous system projection neuron axonogenesis | EN-124k-90-group2917.Contig4 | | | | | | | | | |  |  |  |  |
| M | 0021952 | central nervous system projection neuron axonogenesis | EN-124k-90-group3082.Contig1 | | | | | | | | | |  |  |  |  |
| M | 0021952 | central nervous system projection neuron axonogenesis | EN-124k-90-group3082.Contig2 | | | | | | | | | |  |  |  |  |
| M | 0021952 | central nervous system projection neuron axonogenesis | EN-124k-90-group3223.Contig2 | | | | | | | | | |  |  |  |  |
| M | 0021952 | central nervous system projection neuron axonogenesis | EN-124k-90-group3313.Contig3 | | | | | | | | | |  |  |  |  |
| M | 0021952 | central nervous system projection neuron axonogenesis | EN-124k-90-group3315.Contig2 | | | | | | | | | |  |  |  |  |
| M | 0021952 | central nervous system projection neuron axonogenesis | EN-124k-90-group3315.Contig5 | | | | | | | | | |  |  |  |  |
| M | 0021952 | central nervous system projection neuron axonogenesis | EN-124k-90-group3315.Contig6 | | | | | | | | | |  |  |  |  |
| M | 0021952 | central nervous system projection neuron axonogenesis | EN-124k-90-group3365.Contig1 | | | | | | | | | |  |  |  |  |
| M | 0021952 | central nervous system projection neuron axonogenesis | EN-124k-90-group3394.Contig1 | | | | | | | | | |  |  |  |  |
| M | 0021952 | central nervous system projection neuron axonogenesis | EN-124k-90-group3394.Contig2 | | | | | | | | | |  |  |  |  |
| M | 0021952 | central nervous system projection neuron axonogenesis | EN-124k-90-group3394.Contig3 | | | | | | | | | |  |  |  |  |
| M | 0021952 | central nervous system projection neuron axonogenesis | EN-124k-90-group3394.Contig4 | | | | | | | | | |  |  |  |  |
| M | 0021952 | central nervous system projection neuron axonogenesis | EN-124k-90-group3479.Contig1 | | | | | | | | | |  |  |  |  |
| M | 0021952 | central nervous system projection neuron axonogenesis | EN-124k-90-group3503.Contig2 | | | | | | | | | |  |  |  |  |
| M | 0021952 | central nervous system projection neuron axonogenesis | EN-124k-90-group3503.Contig5 | | | | | | | | | |  |  |  |  |
| M | 0021952 | central nervous system projection neuron axonogenesis | EN-124k-90-group3700.Contig1 | | | | | | | | | |  |  |  |  |
| M | 0021952 | central nervous system projection neuron axonogenesis | EN-124k-90-group3830.Contig1 | | | | | | | | | |  |  |  |  |
| M | 0021952 | central nervous system projection neuron axonogenesis | EN-124k-90-group3933.Contig1 | | | | | | | | | |  |  |  |  |
| M | 0021952 | central nervous system projection neuron axonogenesis | EN-124k-90-group3997.Contig2 | | | | | | | | | |  |  |  |  |
| M | 0021952 | central nervous system projection neuron axonogenesis | EN-124k-90-group4183.Contig1 | | | | | | | | | |  |  |  |  |
| M | 0021952 | central nervous system projection neuron axonogenesis | EN-124k-90-group4372.Contig1 | | | | | | | | | |  |  |  |  |
| M | 0021952 | central nervous system projection neuron axonogenesis | EN-124k-90-group4410.Contig1 | | | | | | | | | |  |  |  |  |
| M | 0021952 | central nervous system projection neuron axonogenesis | EN-124k-90-group4562.Contig1 | | | | | | | | | |  |  |  |  |
| M | 0021952 | central nervous system projection neuron axonogenesis | EN-124k-90-group4753.Contig1 | | | | | | | | | |  |  |  |  |
| M | 0021952 | central nervous system projection neuron axonogenesis | EN-124k-90-group4929.Contig2 | | | | | | | | | |  |  |  |  |
| M | 0021952 | central nervous system projection neuron axonogenesis | EN-124k-90-group5172.Contig1 | | | | | | | | | |  |  |  |  |
| M | 0021952 | central nervous system projection neuron axonogenesis | EN-124k-90-group5173.Contig1 | | | | | | | | | |  |  |  |  |
| M | 0021952 | central nervous system projection neuron axonogenesis | EN-124k-90-group5273.Contig3 | | | | | | | | | |  |  |  |  |
| M | 0021952 | central nervous system projection neuron axonogenesis | EN-124k-90-group5337.Contig1 | | | | | | | | | |  |  |  |  |
| M | 0021952 | central nervous system projection neuron axonogenesis | EN-124k-90-group5380.Contig1 | | | | | | | | | |  |  |  |  |
| M | 0021952 | central nervous system projection neuron axonogenesis | EN-124k-90-group5411.Contig1 | | | | | | | | | |  |  |  |  |
| M | 0021952 | central nervous system projection neuron axonogenesis | EN-124k-90-group5471.Contig3 | | | | | | | | | |  |  |  |  |
| M | 0021952 | central nervous system projection neuron axonogenesis | EN-124k-90-group5479.Contig1 | | | | | | | | | |  |  |  |  |
| M | 0021952 | central nervous system projection neuron axonogenesis | EN-124k-90-group5527.Contig2 | | | | | | | | | |  |  |  |  |
| M | 0021952 | central nervous system projection neuron axonogenesis | EN-124k-90-group5542.Contig1 | | | | | | | | | |  |  |  |  |
| M | 0021952 | central nervous system projection neuron axonogenesis | EN-124k-90-group5607.Contig1 | | | | | | | | | |  |  |  |  |
| M | 0021952 | central nervous system projection neuron axonogenesis | EN-124k-90-group5717.Contig2 | | | | | | | | | |  |  |  |  |
| M | 0021952 | central nervous system projection neuron axonogenesis | EN-124k-90-group5968.Contig1 | | | | | | | | | |  |  |  |  |
| E | 0021952 | central nervous system projection neuron axonogenesis | EN-124k-90-group6238.Contig1 | | | | | | | | | |  |  |  |  |
| M | 0021952 | central nervous system projection neuron axonogenesis | EN-124k-90-group6291.Contig2 | | | | | | | | | |  |  |  |  |
| M | 0021952 | central nervous system projection neuron axonogenesis | EN-124k-90-group6358.Contig1 | | | | | | | | | |  |  |  |  |
| M | 0021952 | central nervous system projection neuron axonogenesis | EN-124k-90-group6358.Contig2 | | | | | | | | | |  |  |  |  |
| M | 0021952 | central nervous system projection neuron axonogenesis | EN-124k-90-group6400.Contig1 | | | | | | | | | |  |  |  |  |
| M | 0021952 | central nervous system projection neuron axonogenesis | EN-124k-90-group6666.Contig1 | | | | | | | | | |  |  |  |  |
| M | 0021952 | central nervous system projection neuron axonogenesis | EN-124k-90-group6923.Contig1 | | | | | | | | | |  |  |  |  |
| M | 0021952 | central nervous system projection neuron axonogenesis | EN-124k-90-group6923.Contig2 | | | | | | | | | |  |  |  |  |
| M | 0021952 | central nervous system projection neuron axonogenesis | EN-124k-90-group7149.Contig2 | | | | | | | | | |  |  |  |  |
| M | 0021952 | central nervous system projection neuron axonogenesis | EN-124k-90-group7157.Contig2 | | | | | | | | | |  |  |  |  |
| M | 0021952 | central nervous system projection neuron axonogenesis | EN-124k-90-group7231.Contig1 | | | | | | | | | |  |  |  |  |
| M | 0021952 | central nervous system projection neuron axonogenesis | EN-124k-90-group7343.Contig1 | | | | | | | | | |  |  |  |  |
| M | 0021952 | central nervous system projection neuron axonogenesis | EN-124k-90-group7528.Contig1 | | | | | | | | | |  |  |  |  |
| M | 0021952 | central nervous system projection neuron axonogenesis | EN-124k-90-group7612.Contig2 | | | | | | | | | |  |  |  |  |
| M | 0021952 | central nervous system projection neuron axonogenesis | EN-124k-90-group7866.Contig1 | | | | | | | | | |  |  |  |  |
| M | 0021952 | central nervous system projection neuron axonogenesis | EN-124k-90-group8034.Contig1 | | | | | | | | | |  |  |  |  |
| M | 0021952 | central nervous system projection neuron axonogenesis | EN-124k-90-group8079.Contig1 | | | | | | | | | |  |  |  |  |
| M | 0021952 | central nervous system projection neuron axonogenesis | EN-124k-90-group8079.Contig2 | | | | | | | | | |  |  |  |  |
| E | 0021952 | central nervous system projection neuron axonogenesis | EN-124k-90-group8101.Contig1 | | | | | | | | | |  |  |  |  |
| M | 0021952 | central nervous system projection neuron axonogenesis | EN-124k-90-group8211.Contig1 | | | | | | | | | |  |  |  |  |
| M | 0021952 | central nervous system projection neuron axonogenesis | EN-124k-90-group8297.Contig1 | | | | | | | | | |  |  |  |  |
| M | 0021952 | central nervous system projection neuron axonogenesis | EN-124k-90-group8375.Contig1 | | | | | | | | | |  |  |  |  |
| A | 0021952 | central nervous system projection neuron axonogenesis | EN-124k-90-group8565.Contig1 | | | | | | | | | |  |  |  |  |
| M | 0021952 | central nervous system projection neuron axonogenesis | EN-124k-90-group8799.Contig2 | | | | | | | | | |  |  |  |  |
| M | 0021952 | central nervous system projection neuron axonogenesis | EN-124k-90-group8937.Contig1 | | | | | | | | | |  |  |  |  |
| M | 0021952 | central nervous system projection neuron axonogenesis | EN-124k-90-group8937.Contig2 | | | | | | | | | |  |  |  |  |
| M | 0021952 | central nervous system projection neuron axonogenesis | EN-124k-90-group9278.Contig2 | | | | | | | | | |  |  |  |  |
| M | 0021952 | central nervous system projection neuron axonogenesis | EN-124k-90-group9295.Contig1 | | | | | | | | | |  |  |  |  |
| A | 0021952 | central nervous system projection neuron axonogenesis | EN-124k-90-group9295.Contig2 | | | | | | | | | |  |  |  |  |
| M | 0021952 | central nervous system projection neuron axonogenesis | EN-124k-90-group9299.Contig3 | | | | | | | | | |  |  |  |  |
| M | 0021952 | central nervous system projection neuron axonogenesis | EN-124k-90-group9317.Contig1 | | | | | | | | | |  |  |  |  |
| M | 0021952 | central nervous system projection neuron axonogenesis | EN-124k-90-group9446.Contig1 | | | | | | | | | |  |  |  |  |
| M | 0021952 | central nervous system projection neuron axonogenesis | EN-124k-90-group9620.Contig1 | | | | | | | | | |  |  |  |  |
| M | 0021952 | central nervous system projection neuron axonogenesis | EN-124k-90-group9712.Contig1 | | | | | | | | | |  |  |  |  |
| M | 0021952 | central nervous system projection neuron axonogenesis | EN-124k-90-group9747.Contig1 | | | | | | | | | |  |  |  |  |
| M | 0021952 | central nervous system projection neuron axonogenesis | EN-124k-90-group9747.Contig2 | | | | | | | | | |  |  |  |  |
| M | 0021952 | central nervous system projection neuron axonogenesis | EN-124k-90-group9819.Contig1 | | | | | | | | | |  |  |  |  |
| M | 0021952 | central nervous system projection neuron axonogenesis | EN-124k-90-group10221.Contig1 | | | | | | | | | |  |  |  |  |
| M | 0021952 | central nervous system projection neuron axonogenesis | EN-124k-90-group10598.Contig1 | | | | | | | | | |  |  |  |  |
| M | 0021952 | central nervous system projection neuron axonogenesis | EN-124k-90-group10630.Contig1 | | | | | | | | | |  |  |  |  |
| M | 0021952 | central nervous system projection neuron axonogenesis | EN-124k-90-group10630.Contig2 | | | | | | | | | |  |  |  |  |
| M | 0021952 | central nervous system projection neuron axonogenesis | EN-124k-90-group10712.Contig1 | | | | | | | | | |  |  |  |  |
| M | 0021952 | central nervous system projection neuron axonogenesis | EN-124k-90-group10838.Contig2 | | | | | | | | | |  |  |  |  |
| M | 0021952 | central nervous system projection neuron axonogenesis | EN-124k-90-group10877.Contig1 | | | | | | | | | |  |  |  |  |
| M | 0021952 | central nervous system projection neuron axonogenesis | EN-124k-90-group10912.Contig1 | | | | | | | | | |  |  |  |  |
| M | 0021952 | central nervous system projection neuron axonogenesis | EN-124k-90-group10928.Contig1 | | | | | | | | | |  |  |  |  |
| M | 0021952 | central nervous system projection neuron axonogenesis | EN-124k-90-group10950.Contig1 | | | | | | | | | |  |  |  |  |
| M | 0021952 | central nervous system projection neuron axonogenesis | EN-124k-90-group11134.Contig1 | | | | | | | | | |  |  |  |  |
| M | 0021952 | central nervous system projection neuron axonogenesis | EN-124k-90-group11228.Contig1 | | | | | | | | | |  |  |  |  |
| M | 0021952 | central nervous system projection neuron axonogenesis | EN-124k-90-group11235.Contig1 | | | | | | | | | |  |  |  |  |
| M | 0021952 | central nervous system projection neuron axonogenesis | EN-124k-90-group11399.Contig2 | | | | | | | | | |  |  |  |  |
| M | 0021952 | central nervous system projection neuron axonogenesis | EN-124k-90-group11408.Contig1 | | | | | | | | | |  |  |  |  |
| M | 0021952 | central nervous system projection neuron axonogenesis | EN-124k-90-group11516.Contig1 | | | | | | | | | |  |  |  |  |
| M | 0021952 | central nervous system projection neuron axonogenesis | EN-124k-90-group11516.Contig2 | | | | | | | | | |  |  |  |  |
| M | 0021952 | central nervous system projection neuron axonogenesis | EN-124k-90-group11686.Contig1 | | | | | | | | | |  |  |  |  |
| M | 0021952 | central nervous system projection neuron axonogenesis | EN-124k-90-group11808.Contig1 | | | | | | | | | |  |  |  |  |
| M | 0021952 | central nervous system projection neuron axonogenesis | EN-124k-90-group12099.Contig1 | | | | | | | | | |  |  |  |  |
| M | 0021952 | central nervous system projection neuron axonogenesis | EN-124k-90-group12143.Contig1 | | | | | | | | | |  |  |  |  |
| M | 0021952 | central nervous system projection neuron axonogenesis | EN-124k-90-group12519.Contig1 | | | | | | | | | |  |  |  |  |
| E | 0021952 | central nervous system projection neuron axonogenesis | EN-124k-90-group13069.Contig2 | | | | | | | | | |  |  |  |  |
| M | 0021952 | central nervous system projection neuron axonogenesis | EN-124k-90-group13235.Contig1 | | | | | | | | | |  |  |  |  |
| M | 0021952 | central nervous system projection neuron axonogenesis | EN-124k-90-group14134.Contig1 | | | | | | | | | |  |  |  |  |
| A | 0021952 | central nervous system projection neuron axonogenesis | EN-124k-90-group14199.Contig1 | | | | | | | | | |  |  |  |  |
| M | 0021952 | central nervous system projection neuron axonogenesis | EN-124k-90-group14708.Contig1 | | | | | | | | | |  |  |  |  |
| M | 0021952 | central nervous system projection neuron axonogenesis | EN-124k-90-group14975.Contig1 | | | | | | | | | |  |  |  |  |
| M | 0021952 | central nervous system projection neuron axonogenesis | EN-124k-90-group14975.Contig2 | | | | | | | | | |  |  |  |  |
| A | 0021952 | central nervous system projection neuron axonogenesis | EN-124k-90-group230.gs\_25885 | | | | | | | | | |  |  |  |  |
| E | 0021952 | central nervous system projection neuron axonogenesis | EN-124k-90-group359.jgi\_contig\_JGI\_CBBP16242\_fwd | | | | | | | | | | | | |  |
| E | 0021952 | central nervous system projection neuron axonogenesis | EN-124k-90-group359.EN\_iowa\_14996 | | | | | | | | | | |  |  |  |
| E | 0021952 | central nervous system projection neuron axonogenesis | EN-124k-90-group359.EN\_iowa\_1876 | | | | | | | | | | |  |  |  |
| E | 0021952 | central nervous system projection neuron axonogenesis | EN-124k-90-group359.EN\_iowa\_13183 | | | | | | | | | | |  |  |  |
| A | 0021952 | central nervous system projection neuron axonogenesis | EN-124k-90-group359.gs\_11387 | | | | | | | | | |  |  |  |  |
| A | 0021952 | central nervous system projection neuron axonogenesis | EN-124k-90-group359.gs\_45042 | | | | | | | | | |  |  |  |  |
| A | 0021952 | central nervous system projection neuron axonogenesis | EN-124k-90-group359.gs\_60113 | | | | | | | | | |  |  |  |  |
| A | 0021952 | central nervous system projection neuron axonogenesis | EN-124k-90-group735.gs\_7849 | | | | | | | | | |  |  |  |  |
| E | 0021952 | central nervous system projection neuron axonogenesis | EN-124k-90-group900.EN\_iowa\_15436 | | | | | | | | | | |  |  |  |
| A | 0021952 | central nervous system projection neuron axonogenesis | EN-124k-90-group900.gs\_31783 | | | | | | | | | |  |  |  |  |
| A | 0021952 | central nervous system projection neuron axonogenesis | EN-124k-90-group900.gs\_71447 | | | | | | | | | |  |  |  |  |
| A | 0021952 | central nervous system projection neuron axonogenesis | EN-124k-90-group1642.gs\_68829 | | | | | | | | | |  |  |  |  |
| A | 0021952 | central nervous system projection neuron axonogenesis | EN-124k-90-group1642.gs\_49728 | | | | | | | | | |  |  |  |  |
| E | 0021952 | central nervous system projection neuron axonogenesis | EN-124k-90-group1642.jgi\_contig\_JGI\_CBBP16332\_fwd | | | | | | | | | | | | |  |
| A | 0021952 | central nervous system projection neuron axonogenesis | EN-124k-90-group1642.gs\_63459 | | | | | | | | | |  |  |  |  |
| E | 0021952 | central nervous system projection neuron axonogenesis | EN-124k-90-group1642.jgi\_contig\_JGI\_CBBP16017\_fwd | | | | | | | | | | | | |  |
| A | 0021952 | central nervous system projection neuron axonogenesis | EN-124k-90-group1642.gs\_32797 | | | | | | | | | |  |  |  |  |
| E | 0021952 | central nervous system projection neuron axonogenesis | EN-124k-90-group1642.EN\_iowa\_1848 | | | | | | | | | | |  |  |  |
| A | 0021952 | central nervous system projection neuron axonogenesis | EN-124k-90-group1782.gs\_16663 | | | | | | | | | |  |  |  |  |
| A | 0021952 | central nervous system projection neuron axonogenesis | EN-124k-90-group2449.gs\_35369 | | | | | | | | | |  |  |  |  |
| E | 0021952 | central nervous system projection neuron axonogenesis | EN-124k-90-group2778.jgi\_contig\_JGI\_CBBP17773\_fwd | | | | | | | | | | | | |  |
| E | 0021952 | central nervous system projection neuron axonogenesis | EN-124k-90-group5607.jgi\_contig\_JGI\_CBBP445\_fwd | | | | | | | | | | | | |  |
| E | 0021952 | central nervous system projection neuron axonogenesis | EN-124k-90-group6923.jgi\_contig\_JGI\_CBBP17789\_fwd | | | | | | | | | | | | |  |
| A | 0021952 | central nervous system projection neuron axonogenesis | EN-124k-90-group9299.gs\_87064 | | | | | | | | | |  |  |  |  |
| E | 0021893 | cerebral cortex GABAergic interneuron fate commitment | EN-124k-90-group3265.jgi\_paired\_JGI\_CBBP17018\_rev | | | | | | | | | | | | |  |
| A | 0021893 | cerebral cortex GABAergic interneuron fate commitment | EN-124k-90-group7595.gs\_36261 | | | | | | | | | |  |  |  |  |
| E | 0021893 | cerebral cortex GABAergic interneuron fate commitment | EN-124k-90-group9064.EN\_iowa\_5147 | | | | | | | | | | |  |  |  |
| E | 0021893 | cerebral cortex GABAergic interneuron fate commitment | EN-124k-90-group10416.jgi\_contig\_JGI\_CBBP3274\_fwd | | | | | | | | | | | | |  |
| E | 0021893 | cerebral cortex GABAergic interneuron fate commitment | EN-124k-90-group15532.EN\_iowa\_5883 | | | | | | | | | | |  |  |  |
| A | 0021893 | cerebral cortex GABAergic interneuron fate commitment | EN-124k-90-group16489.gs\_80464 | | | | | | | | | |  |  |  |  |
| A | 0021893 | cerebral cortex GABAergic interneuron fate commitment | EN-124k-90-group16830.gs\_82512 | | | | | | | | | |  |  |  |  |
| M | 0021893 | cerebral cortex GABAergic interneuron fate commitment | EN-124k-90-group1423.Contig14 | | | | | | | | | |  |  |  |  |
| M | 0021893 | cerebral cortex GABAergic interneuron fate commitment | EN-124k-90-group1423.Contig20 | | | | | | | | | |  |  |  |  |
| M | 0021893 | cerebral cortex GABAergic interneuron fate commitment | EN-124k-90-group2223.Contig1 | | | | | | | | | |  |  |  |  |
| M | 0021893 | cerebral cortex GABAergic interneuron fate commitment | EN-124k-90-group3586.Contig1 | | | | | | | | | |  |  |  |  |
| M | 0021893 | cerebral cortex GABAergic interneuron fate commitment | EN-124k-90-group5252.Contig1 | | | | | | | | | |  |  |  |  |
| M | 0021893 | cerebral cortex GABAergic interneuron fate commitment | EN-124k-90-group5491.Contig1 | | | | | | | | | |  |  |  |  |
| M | 0021893 | cerebral cortex GABAergic interneuron fate commitment | EN-124k-90-group5491.Contig2 | | | | | | | | | |  |  |  |  |
| M | 0021893 | cerebral cortex GABAergic interneuron fate commitment | EN-124k-90-group7915.Contig1 | | | | | | | | | |  |  |  |  |
| A | 0021893 | cerebral cortex GABAergic interneuron fate commitment | EN-124k-90-group1423.gs\_16014 | | | | | | | | | |  |  |  |  |
| A | 0021893 | cerebral cortex GABAergic interneuron fate commitment | EN-124k-90-group1423.gs\_11172 | | | | | | | | | |  |  |  |  |
| A | 0021893 | cerebral cortex GABAergic interneuron fate commitment | EN-124k-90-group1423.gs\_52362 | | | | | | | | | |  |  |  |  |
| A | 0021893 | cerebral cortex GABAergic interneuron fate commitment | EN-124k-90-group1423.gs\_75717 | | | | | | | | | |  |  |  |  |
| A | 0021893 | cerebral cortex GABAergic interneuron fate commitment | EN-124k-90-group5491.gs\_7631 | | | | | | | | | |  |  |  |  |
| A | 0021893 | cerebral cortex GABAergic interneuron fate commitment | EN-124k-90-group5491.gs\_75129 | | | | | | | | | |  |  |  |  |
| A | 0021853 | cerebral cortex GABAergic interneuron migration | EN-124k-90-group5346.gs\_72008 | | | | | | | | | |  |  |  |  |
| A | 0021853 | cerebral cortex GABAergic interneuron migration | EN-124k-90-group7595.gs\_36261 | | | | | | | | | |  |  |  |  |
| E | 0021853 | cerebral cortex GABAergic interneuron migration | EN-124k-90-group9064.EN\_iowa\_5147 | | | | | | | | | | |  |  |  |
| A | 0021853 | cerebral cortex GABAergic interneuron migration | EN-124k-90-group9910.gs\_20549 | | | | | | | | | |  |  |  |  |
| E | 0021853 | cerebral cortex GABAergic interneuron migration | EN-124k-90-group10110.jgi\_paired\_JGI\_CBBP10219\_fwd | | | | | | | | | | | | |  |
| E | 0021853 | cerebral cortex GABAergic interneuron migration | EN-124k-90-group10416.jgi\_contig\_JGI\_CBBP3274\_fwd | | | | | | | | | | | | |  |
| A | 0021853 | cerebral cortex GABAergic interneuron migration | EN-124k-90-group11441.gs\_30577 | | | | | | | | | |  |  |  |  |
| E | 0021853 | cerebral cortex GABAergic interneuron migration | EN-124k-90-group14544.jgi\_contig\_JGI\_CBBP14322\_fwd | | | | | | | | | | | | |  |
| A | 0021853 | cerebral cortex GABAergic interneuron migration | EN-124k-90-group14813.gs\_84560 | | | | | | | | | |  |  |  |  |
| A | 0021853 | cerebral cortex GABAergic interneuron migration | EN-124k-90-group15413.gs\_80915 | | | | | | | | | |  |  |  |  |
| E | 0021853 | cerebral cortex GABAergic interneuron migration | EN-124k-90-group15532.EN\_iowa\_5883 | | | | | | | | | | |  |  |  |
| A | 0021853 | cerebral cortex GABAergic interneuron migration | EN-124k-90-group15856.gs\_15241 | | | | | | | | | |  |  |  |  |
| A | 0021853 | cerebral cortex GABAergic interneuron migration | EN-124k-90-group16523.gs\_75670 | | | | | | | | | |  |  |  |  |
| M | 0021853 | cerebral cortex GABAergic interneuron migration | EN-124k-90-group1117.Contig4 | | | | | | | | | |  |  |  |  |
| M | 0021853 | cerebral cortex GABAergic interneuron migration | EN-124k-90-group1302.Contig7 | | | | | | | | | |  |  |  |  |
| M | 0021853 | cerebral cortex GABAergic interneuron migration | EN-124k-90-group1423.Contig14 | | | | | | | | | |  |  |  |  |
| M | 0021853 | cerebral cortex GABAergic interneuron migration | EN-124k-90-group1423.Contig20 | | | | | | | | | |  |  |  |  |
| M | 0021853 | cerebral cortex GABAergic interneuron migration | EN-124k-90-group2223.Contig1 | | | | | | | | | |  |  |  |  |
| M | 0021853 | cerebral cortex GABAergic interneuron migration | EN-124k-90-group3018.Contig2 | | | | | | | | | |  |  |  |  |
| M | 0021853 | cerebral cortex GABAergic interneuron migration | EN-124k-90-group3586.Contig1 | | | | | | | | | |  |  |  |  |
| M | 0021853 | cerebral cortex GABAergic interneuron migration | EN-124k-90-group3816.Contig1 | | | | | | | | | |  |  |  |  |
| M | 0021853 | cerebral cortex GABAergic interneuron migration | EN-124k-90-group3859.Contig1 | | | | | | | | | |  |  |  |  |
| M | 0021853 | cerebral cortex GABAergic interneuron migration | EN-124k-90-group5203.Contig1 | | | | | | | | | |  |  |  |  |
| M | 0021853 | cerebral cortex GABAergic interneuron migration | EN-124k-90-group5203.Contig2 | | | | | | | | | |  |  |  |  |
| M | 0021853 | cerebral cortex GABAergic interneuron migration | EN-124k-90-group5252.Contig1 | | | | | | | | | |  |  |  |  |
| M | 0021853 | cerebral cortex GABAergic interneuron migration | EN-124k-90-group5491.Contig1 | | | | | | | | | |  |  |  |  |
| M | 0021853 | cerebral cortex GABAergic interneuron migration | EN-124k-90-group5491.Contig2 | | | | | | | | | |  |  |  |  |
| M | 0021853 | cerebral cortex GABAergic interneuron migration | EN-124k-90-group6307.Contig2 | | | | | | | | | |  |  |  |  |
| M | 0021853 | cerebral cortex GABAergic interneuron migration | EN-124k-90-group6307.Contig3 | | | | | | | | | |  |  |  |  |
| M | 0021853 | cerebral cortex GABAergic interneuron migration | EN-124k-90-group7915.Contig1 | | | | | | | | | |  |  |  |  |
| M | 0021853 | cerebral cortex GABAergic interneuron migration | EN-124k-90-group8418.Contig1 | | | | | | | | | |  |  |  |  |
| M | 0021853 | cerebral cortex GABAergic interneuron migration | EN-124k-90-group10750.Contig1 | | | | | | | | | |  |  |  |  |
| A | 0021853 | cerebral cortex GABAergic interneuron migration | EN-124k-90-group1423.gs\_16014 | | | | | | | | | |  |  |  |  |
| A | 0021853 | cerebral cortex GABAergic interneuron migration | EN-124k-90-group1423.gs\_11172 | | | | | | | | | |  |  |  |  |
| A | 0021853 | cerebral cortex GABAergic interneuron migration | EN-124k-90-group1423.gs\_52362 | | | | | | | | | |  |  |  |  |
| A | 0021853 | cerebral cortex GABAergic interneuron migration | EN-124k-90-group1423.gs\_75717 | | | | | | | | | |  |  |  |  |
| A | 0021853 | cerebral cortex GABAergic interneuron migration | EN-124k-90-group5203.gs\_53868 | | | | | | | | | |  |  |  |  |
| A | 0021853 | cerebral cortex GABAergic interneuron migration | EN-124k-90-group5203.gs\_41715 | | | | | | | | | |  |  |  |  |
| A | 0021853 | cerebral cortex GABAergic interneuron migration | EN-124k-90-group5491.gs\_7631 | | | | | | | | | |  |  |  |  |
| A | 0021853 | cerebral cortex GABAergic interneuron migration | EN-124k-90-group5491.gs\_75129 | | | | | | | | | |  |  |  |  |
| A | 0021853 | cerebral cortex GABAergic interneuron migration | EN-124k-90-group6307.gs\_50382 | | | | | | | | | |  |  |  |  |
| A | 0021853 | cerebral cortex GABAergic interneuron migration | EN-124k-90-group6307.gs\_86786 | | | | | | | | | |  |  |  |  |
| A | 0021895 | cerebral cortex neuron differentiation | EN-124k-90-group8404.gs\_39024 | | | | | | | | | |  |  |  |  |
| E | 0021895 | cerebral cortex neuron differentiation | EN-124k-90-group10406.jgi\_contig\_JGI\_CBBP11462\_fwd | | | | | | | | | | | | |  |
| E | 0021895 | cerebral cortex neuron differentiation | EN-124k-90-group13814.jgi\_paired\_JGI\_CBBP18956\_fwd | | | | | | | | | | | | |  |
| A | 0021895 | cerebral cortex neuron differentiation | EN-124k-90-group16439.gs\_54729 | | | | | | | | | |  |  |  |  |
| A | 0021895 | cerebral cortex neuron differentiation | EN-124k-90-group16830.gs\_82512 | | | | | | | | | |  |  |  |  |
| A | 0021895 | cerebral cortex neuron differentiation | EN-124k-90-group17012.gs\_52263 | | | | | | | | | |  |  |  |  |
| A | 0021895 | cerebral cortex neuron differentiation | EN-124k-90-group17411.gs\_13940 | | | | | | | | | |  |  |  |  |
| A | 0021895 | cerebral cortex neuron differentiation | EN-124k-90-group18038.gs\_30262 | | | | | | | | | |  |  |  |  |
| M | 0021895 | cerebral cortex neuron differentiation | EN-124k-90-group1297.Contig1 | | | | | | | | | |  |  |  |  |
| M | 0021895 | cerebral cortex neuron differentiation | EN-124k-90-group2418.Contig1 | | | | | | | | | |  |  |  |  |
| M | 0021895 | cerebral cortex neuron differentiation | EN-124k-90-group4500.Contig1 | | | | | | | | | |  |  |  |  |
| M | 0021895 | cerebral cortex neuron differentiation | EN-124k-90-group4761.Contig1 | | | | | | | | | |  |  |  |  |
| M | 0021895 | cerebral cortex neuron differentiation | EN-124k-90-group4761.Contig2 | | | | | | | | | |  |  |  |  |
| M | 0021895 | cerebral cortex neuron differentiation | EN-124k-90-group5178.Contig1 | | | | | | | | | |  |  |  |  |
| M | 0021895 | cerebral cortex neuron differentiation | EN-124k-90-group10147.Contig2 | | | | | | | | | |  |  |  |  |
| E | 0021895 | cerebral cortex neuron differentiation | EN-124k-90-group5178.EN\_iowa\_6303 | | | | | | | | | | |  |  |  |
| A | 0021895 | cerebral cortex neuron differentiation | EN-124k-90-group5178.gs\_4377 | | | | | | | | | |  |  |  |  |
| A | 0005127 | ciliary neurotrophic factor receptor binding | EN-124k-90-group18774.gs\_57540 | | | | | | | | | |  |  |  |  |
| E | 0021902 | commitment of a neuronal cell to a specific type of neuron in the forebrain | EN-124k-90-group10416.jgi\_contig\_JGI\_CBBP3274\_fwd | | | | | | | | | | | | |  |
| E | 0021902 | commitment of a neuronal cell to a specific type of neuron in the forebrain | EN-124k-90-group14544.jgi\_contig\_JGI\_CBBP14322\_fwd | | | | | | | | | | | | |  |
| E | 0021902 | commitment of a neuronal cell to a specific type of neuron in the forebrain | EN-124k-90-group15532.EN\_iowa\_5883 | | | | | | | | | | |  |  |  |
| M | 0021902 | commitment of a neuronal cell to a specific type of neuron in the forebrain | EN-124k-90-group1423.Contig14 | | | | | | | | | |  |  |  |  |
| M | 0021902 | commitment of a neuronal cell to a specific type of neuron in the forebrain | EN-124k-90-group1423.Contig20 | | | | | | | | | |  |  |  |  |
| M | 0021902 | commitment of a neuronal cell to a specific type of neuron in the forebrain | EN-124k-90-group3586.Contig1 | | | | | | | | | |  |  |  |  |
| M | 0021902 | commitment of a neuronal cell to a specific type of neuron in the forebrain | EN-124k-90-group5252.Contig1 | | | | | | | | | |  |  |  |  |
| M | 0021902 | commitment of a neuronal cell to a specific type of neuron in the forebrain | EN-124k-90-group5491.Contig1 | | | | | | | | | |  |  |  |  |
| M | 0021902 | commitment of a neuronal cell to a specific type of neuron in the forebrain | EN-124k-90-group5491.Contig2 | | | | | | | | | |  |  |  |  |
| M | 0021902 | commitment of a neuronal cell to a specific type of neuron in the forebrain | EN-124k-90-group7915.Contig1 | | | | | | | | | |  |  |  |  |
| M | 0021902 | commitment of a neuronal cell to a specific type of neuron in the forebrain | EN-124k-90-group12638.Contig1 | | | | | | | | | |  |  |  |  |
| M | 0021902 | commitment of a neuronal cell to a specific type of neuron in the forebrain | EN-124k-90-group14577.Contig1 | | | | | | | | | |  |  |  |  |
| A | 0021902 | commitment of a neuronal cell to a specific type of neuron in the forebrain | EN-124k-90-group1423.gs\_16014 | | | | | | | | | |  |  |  |  |
| A | 0021902 | commitment of a neuronal cell to a specific type of neuron in the forebrain | EN-124k-90-group1423.gs\_11172 | | | | | | | | | |  |  |  |  |
| A | 0021902 | commitment of a neuronal cell to a specific type of neuron in the forebrain | EN-124k-90-group1423.gs\_52362 | | | | | | | | | |  |  |  |  |
| A | 0021902 | commitment of a neuronal cell to a specific type of neuron in the forebrain | EN-124k-90-group1423.gs\_75717 | | | | | | | | | |  |  |  |  |
| A | 0021902 | commitment of a neuronal cell to a specific type of neuron in the forebrain | EN-124k-90-group5491.gs\_7631 | | | | | | | | | |  |  |  |  |
| A | 0021902 | commitment of a neuronal cell to a specific type of neuron in the forebrain | EN-124k-90-group5491.gs\_75129 | | | | | | | | | |  |  |  |  |
| E | 0004693 | cyclin-dependent protein kinase activity | EN-124k-90-group343.jgi\_contig\_JGI\_CBBP19299\_fwd | | | | | | | | | | | | |  |
| A | 0004693 | cyclin-dependent protein kinase activity | EN-124k-90-group807.gs\_71281 | | | | | | | | | |  |  |  |  |
| E | 0004693 | cyclin-dependent protein kinase activity | EN-124k-90-group1306.EN\_iowa\_2354 | | | | | | | | | | |  |  |  |
| E | 0004693 | cyclin-dependent protein kinase activity | EN-124k-90-group1680.jgi\_contig\_JGI\_CBBP17232\_fwd | | | | | | | | | | | | |  |
| E | 0004693 | cyclin-dependent protein kinase activity | EN-124k-90-group1852.jgi\_paired\_JGI\_CBBP10940\_fwd | | | | | | | | | | | | |  |
| E | 0004693 | cyclin-dependent protein kinase activity | EN-124k-90-group2530.jgi\_paired\_JGI\_CBBP5537\_fwd | | | | | | | | | | | | |  |
| E | 0004693 | cyclin-dependent protein kinase activity | EN-124k-90-group3448.jgi\_paired\_JGI\_CBBP19729\_fwd | | | | | | | | | | | | |  |
| A | 0004693 | cyclin-dependent protein kinase activity | EN-124k-90-group4315.gs\_20609 | | | | | | | | | |  |  |  |  |
| E | 0004693 | cyclin-dependent protein kinase activity | EN-124k-90-group5117.jgi\_paired\_JGI\_CBBP19153\_fwd | | | | | | | | | | | | |  |
| E | 0004693 | cyclin-dependent protein kinase activity | EN-124k-90-group5741.jgi\_paired\_JGI\_CBBP19133\_fwd | | | | | | | | | | | | |  |
| E | 0004693 | cyclin-dependent protein kinase activity | EN-124k-90-group5834.jgi\_contig\_JGI\_CBBP12701\_fwd | | | | | | | | | | | | |  |
| E | 0004693 | cyclin-dependent protein kinase activity | EN-124k-90-group6737.jgi\_contig\_JGI\_CBBP17820\_fwd | | | | | | | | | | | | |  |
| E | 0004693 | cyclin-dependent protein kinase activity | EN-124k-90-group7594.jgi\_paired\_JGI\_CBBP17768\_fwd | | | | | | | | | | | | |  |
| E | 0004693 | cyclin-dependent protein kinase activity | EN-124k-90-group7598.jgi\_unpaired\_JGI\_CBBP18343\_fwd | | | | | | | | | | | | | |
| A | 0004693 | cyclin-dependent protein kinase activity | EN-124k-90-group8310.gs\_24913 | | | | | | | | | |  |  |  |  |
| A | 0004693 | cyclin-dependent protein kinase activity | EN-124k-90-group9467.gs\_16039 | | | | | | | | | |  |  |  |  |
| A | 0004693 | cyclin-dependent protein kinase activity | EN-124k-90-group9714.gs\_64750 | | | | | | | | | |  |  |  |  |
| E | 0004693 | cyclin-dependent protein kinase activity | EN-124k-90-group9839.EN\_iowa\_2226 | | | | | | | | | | |  |  |  |
| E | 0004693 | cyclin-dependent protein kinase activity | EN-124k-90-group9989.EN\_iowa\_3399 | | | | | | | | | | |  |  |  |
| A | 0004693 | cyclin-dependent protein kinase activity | EN-124k-90-group10312.gs\_22624 | | | | | | | | | |  |  |  |  |
| A | 0004693 | cyclin-dependent protein kinase activity | EN-124k-90-group11738.gs\_53113 | | | | | | | | | |  |  |  |  |
| E | 0004693 | cyclin-dependent protein kinase activity | EN-124k-90-group12191.jgi\_paired\_JGI\_CBBP10887\_fwd | | | | | | | | | | | | |  |
| E | 0004693 | cyclin-dependent protein kinase activity | EN-124k-90-group12452.EN\_iowa\_9722 | | | | | | | | | | |  |  |  |
| E | 0004693 | cyclin-dependent protein kinase activity | EN-124k-90-group13011.jgi\_paired\_JGI\_CBBP19322\_fwd | | | | | | | | | | | | |  |
| E | 0004693 | cyclin-dependent protein kinase activity | EN-124k-90-group13261.jgi\_contig\_JGI\_CBBP9652\_fwd | | | | | | | | | | | | |  |
| E | 0004693 | cyclin-dependent protein kinase activity | EN-124k-90-group13951.jgi\_paired\_JGI\_CBBP12336\_fwd | | | | | | | | | | | | |  |
| E | 0004693 | cyclin-dependent protein kinase activity | EN-124k-90-group14335.jgi\_paired\_JGI\_CBBP12938\_fwd | | | | | | | | | | | | |  |
| A | 0004693 | cyclin-dependent protein kinase activity | EN-124k-90-group14563.gs\_48486 | | | | | | | | | |  |  |  |  |
| E | 0004693 | cyclin-dependent protein kinase activity | EN-124k-90-group14931.jgi\_paired\_JGI\_CBBP10444\_fwd | | | | | | | | | | | | |  |
| A | 0004693 | cyclin-dependent protein kinase activity | EN-124k-90-group15056.gs\_73097 | | | | | | | | | |  |  |  |  |
| E | 0004693 | cyclin-dependent protein kinase activity | EN-124k-90-group15223.EN\_iowa\_18438 | | | | | | | | | | |  |  |  |
| A | 0004693 | cyclin-dependent protein kinase activity | EN-124k-90-group15229.gs\_75551 | | | | | | | | | |  |  |  |  |
| E | 0004693 | cyclin-dependent protein kinase activity | EN-124k-90-group15290.EN\_iowa\_9903 | | | | | | | | | | |  |  |  |
| E | 0004693 | cyclin-dependent protein kinase activity | EN-124k-90-group15320.jgi\_paired\_JGI\_CBBP9817\_fwd | | | | | | | | | | | | |  |
| A | 0004693 | cyclin-dependent protein kinase activity | EN-124k-90-group15770.gs\_45569 | | | | | | | | | |  |  |  |  |
| E | 0004693 | cyclin-dependent protein kinase activity | EN-124k-90-group16143.jgi\_paired\_JGI\_CBBP4408\_fwd | | | | | | | | | | | | |  |
| E | 0004693 | cyclin-dependent protein kinase activity | EN-124k-90-group16200.jgi\_paired\_JGI\_CBBP10444\_rev | | | | | | | | | | | | |  |
| A | 0004693 | cyclin-dependent protein kinase activity | EN-124k-90-group16285.gs\_47129 | | | | | | | | | |  |  |  |  |
| A | 0004693 | cyclin-dependent protein kinase activity | EN-124k-90-group16416.gs\_28586 | | | | | | | | | |  |  |  |  |
| A | 0004693 | cyclin-dependent protein kinase activity | EN-124k-90-group16489.gs\_80464 | | | | | | | | | |  |  |  |  |
| E | 0004693 | cyclin-dependent protein kinase activity | EN-124k-90-group17086.jgi\_paired\_JGI\_CBBP6417\_rev | | | | | | | | | | | | |  |
| E | 0004693 | cyclin-dependent protein kinase activity | EN-124k-90-group17091.jgi\_paired\_JGI\_CBBP10179\_rev | | | | | | | | | | | | |  |
| A | 0004693 | cyclin-dependent protein kinase activity | EN-124k-90-group17386.gs\_68411 | | | | | | | | | |  |  |  |  |
| A | 0004693 | cyclin-dependent protein kinase activity | EN-124k-90-group17411.gs\_13940 | | | | | | | | | |  |  |  |  |
| A | 0004693 | cyclin-dependent protein kinase activity | EN-124k-90-group17442.gs\_63748 | | | | | | | | | |  |  |  |  |
| A | 0004693 | cyclin-dependent protein kinase activity | EN-124k-90-group17556.gs\_87114 | | | | | | | | | |  |  |  |  |
| A | 0004693 | cyclin-dependent protein kinase activity | EN-124k-90-group17563.gs\_80410 | | | | | | | | | |  |  |  |  |
| A | 0004693 | cyclin-dependent protein kinase activity | EN-124k-90-group17582.gs\_70636 | | | | | | | | | |  |  |  |  |
| A | 0004693 | cyclin-dependent protein kinase activity | EN-124k-90-group17657.gs\_68076 | | | | | | | | | |  |  |  |  |
| A | 0004693 | cyclin-dependent protein kinase activity | EN-124k-90-group17702.gs\_18827 | | | | | | | | | |  |  |  |  |
| A | 0004693 | cyclin-dependent protein kinase activity | EN-124k-90-group17930.gs\_83513 | | | | | | | | | |  |  |  |  |
| A | 0004693 | cyclin-dependent protein kinase activity | EN-124k-90-group18140.gs\_87168 | | | | | | | | | |  |  |  |  |
| E | 0004693 | cyclin-dependent protein kinase activity | EN-124k-90-group18538.jgi\_paired\_JGI\_CBBP19133\_rev | | | | | | | | | | | | |  |
| A | 0004693 | cyclin-dependent protein kinase activity | EN-124k-90-group18929.gs\_13816 | | | | | | | | | |  |  |  |  |
| A | 0004693 | cyclin-dependent protein kinase activity | EN-124k-90-group18948.gs\_56541 | | | | | | | | | |  |  |  |  |
| M | 0004693 | cyclin-dependent protein kinase activity | EN-124k-90-group138.Contig1 | | | | | | | | | |  |  |  |  |
| M | 0004693 | cyclin-dependent protein kinase activity | EN-124k-90-group138.Contig35 | | | | | | | | | |  |  |  |  |
| M | 0004693 | cyclin-dependent protein kinase activity | EN-124k-90-group138.Contig62 | | | | | | | | | |  |  |  |  |
| M | 0004693 | cyclin-dependent protein kinase activity | EN-124k-90-group163.Contig2 | | | | | | | | | |  |  |  |  |
| M | 0004693 | cyclin-dependent protein kinase activity | EN-124k-90-group229.Contig1 | | | | | | | | | |  |  |  |  |
| M | 0004693 | cyclin-dependent protein kinase activity | EN-124k-90-group230.Contig1 | | | | | | | | | |  |  |  |  |
| M | 0004693 | cyclin-dependent protein kinase activity | EN-124k-90-group324.Contig1 | | | | | | | | | |  |  |  |  |
| M | 0004693 | cyclin-dependent protein kinase activity | EN-124k-90-group328.Contig2 | | | | | | | | | |  |  |  |  |
| M | 0004693 | cyclin-dependent protein kinase activity | EN-124k-90-group481.Contig4 | | | | | | | | | |  |  |  |  |
| M | 0004693 | cyclin-dependent protein kinase activity | EN-124k-90-group550.Contig1 | | | | | | | | | |  |  |  |  |
| M | 0004693 | cyclin-dependent protein kinase activity | EN-124k-90-group550.Contig2 | | | | | | | | | |  |  |  |  |
| M | 0004693 | cyclin-dependent protein kinase activity | EN-124k-90-group593.Contig1 | | | | | | | | | |  |  |  |  |
| M | 0004693 | cyclin-dependent protein kinase activity | EN-124k-90-group630.Contig2 | | | | | | | | | |  |  |  |  |
| M | 0004693 | cyclin-dependent protein kinase activity | EN-124k-90-group658.Contig1 | | | | | | | | | |  |  |  |  |
| M | 0004693 | cyclin-dependent protein kinase activity | EN-124k-90-group658.Contig3 | | | | | | | | | |  |  |  |  |
| M | 0004693 | cyclin-dependent protein kinase activity | EN-124k-90-group682.Contig1 | | | | | | | | | |  |  |  |  |
| M | 0004693 | cyclin-dependent protein kinase activity | EN-124k-90-group702.Contig2 | | | | | | | | | |  |  |  |  |
| M | 0004693 | cyclin-dependent protein kinase activity | EN-124k-90-group735.Contig1 | | | | | | | | | |  |  |  |  |
| M | 0004693 | cyclin-dependent protein kinase activity | EN-124k-90-group881.Contig2 | | | | | | | | | |  |  |  |  |
| M | 0004693 | cyclin-dependent protein kinase activity | EN-124k-90-group881.Contig3 | | | | | | | | | |  |  |  |  |
| M | 0004693 | cyclin-dependent protein kinase activity | EN-124k-90-group900.Contig1 | | | | | | | | | |  |  |  |  |
| M | 0004693 | cyclin-dependent protein kinase activity | EN-124k-90-group1071.Contig1 | | | | | | | | | |  |  |  |  |
| M | 0004693 | cyclin-dependent protein kinase activity | EN-124k-90-group1071.Contig2 | | | | | | | | | |  |  |  |  |
| M | 0004693 | cyclin-dependent protein kinase activity | EN-124k-90-group1071.Contig3 | | | | | | | | | |  |  |  |  |
| M | 0004693 | cyclin-dependent protein kinase activity | EN-124k-90-group1167.Contig2 | | | | | | | | | |  |  |  |  |
| M | 0004693 | cyclin-dependent protein kinase activity | EN-124k-90-group1167.Contig3 | | | | | | | | | |  |  |  |  |
| M | 0004693 | cyclin-dependent protein kinase activity | EN-124k-90-group1262.Contig1 | | | | | | | | | |  |  |  |  |
| M | 0004693 | cyclin-dependent protein kinase activity | EN-124k-90-group1304.Contig2 | | | | | | | | | |  |  |  |  |
| M | 0004693 | cyclin-dependent protein kinase activity | EN-124k-90-group1304.Contig4 | | | | | | | | | |  |  |  |  |
| M | 0004693 | cyclin-dependent protein kinase activity | EN-124k-90-group1304.Contig8 | | | | | | | | | |  |  |  |  |
| M | 0004693 | cyclin-dependent protein kinase activity | EN-124k-90-group1357.Contig2 | | | | | | | | | |  |  |  |  |
| M | 0004693 | cyclin-dependent protein kinase activity | EN-124k-90-group1357.Contig3 | | | | | | | | | |  |  |  |  |
| M | 0004693 | cyclin-dependent protein kinase activity | EN-124k-90-group1456.Contig1 | | | | | | | | | |  |  |  |  |
| M | 0004693 | cyclin-dependent protein kinase activity | EN-124k-90-group1456.Contig4 | | | | | | | | | |  |  |  |  |
| M | 0004693 | cyclin-dependent protein kinase activity | EN-124k-90-group1456.Contig8 | | | | | | | | | |  |  |  |  |
| M | 0004693 | cyclin-dependent protein kinase activity | EN-124k-90-group1728.Contig2 | | | | | | | | | |  |  |  |  |
| M | 0004693 | cyclin-dependent protein kinase activity | EN-124k-90-group1735.Contig4 | | | | | | | | | |  |  |  |  |
| M | 0004693 | cyclin-dependent protein kinase activity | EN-124k-90-group1735.Contig6 | | | | | | | | | |  |  |  |  |
| M | 0004693 | cyclin-dependent protein kinase activity | EN-124k-90-group1782.Contig1 | | | | | | | | | |  |  |  |  |
| M | 0004693 | cyclin-dependent protein kinase activity | EN-124k-90-group1915.Contig1 | | | | | | | | | |  |  |  |  |
| M | 0004693 | cyclin-dependent protein kinase activity | EN-124k-90-group1960.Contig1 | | | | | | | | | |  |  |  |  |
| M | 0004693 | cyclin-dependent protein kinase activity | EN-124k-90-group2074.Contig1 | | | | | | | | | |  |  |  |  |
| M | 0004693 | cyclin-dependent protein kinase activity | EN-124k-90-group2075.Contig2 | | | | | | | | | |  |  |  |  |
| M | 0004693 | cyclin-dependent protein kinase activity | EN-124k-90-group2081.Contig1 | | | | | | | | | |  |  |  |  |
| M | 0004693 | cyclin-dependent protein kinase activity | EN-124k-90-group2081.Contig3 | | | | | | | | | |  |  |  |  |
| M | 0004693 | cyclin-dependent protein kinase activity | EN-124k-90-group2097.Contig1 | | | | | | | | | |  |  |  |  |
| M | 0004693 | cyclin-dependent protein kinase activity | EN-124k-90-group2200.Contig1 | | | | | | | | | |  |  |  |  |
| M | 0004693 | cyclin-dependent protein kinase activity | EN-124k-90-group2409.Contig1 | | | | | | | | | |  |  |  |  |
| M | 0004693 | cyclin-dependent protein kinase activity | EN-124k-90-group2409.Contig3 | | | | | | | | | |  |  |  |  |
| M | 0004693 | cyclin-dependent protein kinase activity | EN-124k-90-group2409.Contig4 | | | | | | | | | |  |  |  |  |
| M | 0004693 | cyclin-dependent protein kinase activity | EN-124k-90-group2428.Contig1 | | | | | | | | | |  |  |  |  |
| M | 0004693 | cyclin-dependent protein kinase activity | EN-124k-90-group2449.Contig1 | | | | | | | | | |  |  |  |  |
| M | 0004693 | cyclin-dependent protein kinase activity | EN-124k-90-group2449.Contig2 | | | | | | | | | |  |  |  |  |
| M | 0004693 | cyclin-dependent protein kinase activity | EN-124k-90-group2491.Contig1 | | | | | | | | | |  |  |  |  |
| M | 0004693 | cyclin-dependent protein kinase activity | EN-124k-90-group2498.Contig5 | | | | | | | | | |  |  |  |  |
| M | 0004693 | cyclin-dependent protein kinase activity | EN-124k-90-group2498.Contig7 | | | | | | | | | |  |  |  |  |
| A | 0004693 | cyclin-dependent protein kinase activity | EN-124k-90-group2585.Contig1 | | | | | | | | | |  |  |  |  |
| M | 0004693 | cyclin-dependent protein kinase activity | EN-124k-90-group2651.Contig1 | | | | | | | | | |  |  |  |  |
| M | 0004693 | cyclin-dependent protein kinase activity | EN-124k-90-group2778.Contig1 | | | | | | | | | |  |  |  |  |
| M | 0004693 | cyclin-dependent protein kinase activity | EN-124k-90-group2846.Contig1 | | | | | | | | | |  |  |  |  |
| M | 0004693 | cyclin-dependent protein kinase activity | EN-124k-90-group2917.Contig1 | | | | | | | | | |  |  |  |  |
| M | 0004693 | cyclin-dependent protein kinase activity | EN-124k-90-group2917.Contig2 | | | | | | | | | |  |  |  |  |
| M | 0004693 | cyclin-dependent protein kinase activity | EN-124k-90-group2917.Contig4 | | | | | | | | | |  |  |  |  |
| M | 0004693 | cyclin-dependent protein kinase activity | EN-124k-90-group2940.Contig2 | | | | | | | | | |  |  |  |  |
| M | 0004693 | cyclin-dependent protein kinase activity | EN-124k-90-group3082.Contig2 | | | | | | | | | |  |  |  |  |
| M | 0004693 | cyclin-dependent protein kinase activity | EN-124k-90-group3313.Contig1 | | | | | | | | | |  |  |  |  |
| M | 0004693 | cyclin-dependent protein kinase activity | EN-124k-90-group3313.Contig2 | | | | | | | | | |  |  |  |  |
| M | 0004693 | cyclin-dependent protein kinase activity | EN-124k-90-group3313.Contig3 | | | | | | | | | |  |  |  |  |
| M | 0004693 | cyclin-dependent protein kinase activity | EN-124k-90-group3315.Contig2 | | | | | | | | | |  |  |  |  |
| M | 0004693 | cyclin-dependent protein kinase activity | EN-124k-90-group3315.Contig5 | | | | | | | | | |  |  |  |  |
| M | 0004693 | cyclin-dependent protein kinase activity | EN-124k-90-group3315.Contig6 | | | | | | | | | |  |  |  |  |
| M | 0004693 | cyclin-dependent protein kinase activity | EN-124k-90-group3316.Contig1 | | | | | | | | | |  |  |  |  |
| M | 0004693 | cyclin-dependent protein kinase activity | EN-124k-90-group3365.Contig1 | | | | | | | | | |  |  |  |  |
| M | 0004693 | cyclin-dependent protein kinase activity | EN-124k-90-group3394.Contig1 | | | | | | | | | |  |  |  |  |
| M | 0004693 | cyclin-dependent protein kinase activity | EN-124k-90-group3394.Contig3 | | | | | | | | | |  |  |  |  |
| M | 0004693 | cyclin-dependent protein kinase activity | EN-124k-90-group3394.Contig4 | | | | | | | | | |  |  |  |  |
| M | 0004693 | cyclin-dependent protein kinase activity | EN-124k-90-group3479.Contig1 | | | | | | | | | |  |  |  |  |
| M | 0004693 | cyclin-dependent protein kinase activity | EN-124k-90-group3606.Contig2 | | | | | | | | | |  |  |  |  |
| M | 0004693 | cyclin-dependent protein kinase activity | EN-124k-90-group3700.Contig1 | | | | | | | | | |  |  |  |  |
| M | 0004693 | cyclin-dependent protein kinase activity | EN-124k-90-group3830.Contig1 | | | | | | | | | |  |  |  |  |
| M | 0004693 | cyclin-dependent protein kinase activity | EN-124k-90-group4168.Contig1 | | | | | | | | | |  |  |  |  |
| M | 0004693 | cyclin-dependent protein kinase activity | EN-124k-90-group4168.Contig3 | | | | | | | | | |  |  |  |  |
| M | 0004693 | cyclin-dependent protein kinase activity | EN-124k-90-group4183.Contig1 | | | | | | | | | |  |  |  |  |
| M | 0004693 | cyclin-dependent protein kinase activity | EN-124k-90-group4339.Contig1 | | | | | | | | | |  |  |  |  |
| M | 0004693 | cyclin-dependent protein kinase activity | EN-124k-90-group4349.Contig3 | | | | | | | | | |  |  |  |  |
| M | 0004693 | cyclin-dependent protein kinase activity | EN-124k-90-group4372.Contig1 | | | | | | | | | |  |  |  |  |
| M | 0004693 | cyclin-dependent protein kinase activity | EN-124k-90-group4410.Contig1 | | | | | | | | | |  |  |  |  |
| M | 0004693 | cyclin-dependent protein kinase activity | EN-124k-90-group4753.Contig1 | | | | | | | | | |  |  |  |  |
| M | 0004693 | cyclin-dependent protein kinase activity | EN-124k-90-group4757.Contig2 | | | | | | | | | |  |  |  |  |
| M | 0004693 | cyclin-dependent protein kinase activity | EN-124k-90-group4803.Contig1 | | | | | | | | | |  |  |  |  |
| M | 0004693 | cyclin-dependent protein kinase activity | EN-124k-90-group4803.Contig2 | | | | | | | | | |  |  |  |  |
| M | 0004693 | cyclin-dependent protein kinase activity | EN-124k-90-group4929.Contig1 | | | | | | | | | |  |  |  |  |
| M | 0004693 | cyclin-dependent protein kinase activity | EN-124k-90-group4929.Contig2 | | | | | | | | | |  |  |  |  |
| M | 0004693 | cyclin-dependent protein kinase activity | EN-124k-90-group4989.Contig2 | | | | | | | | | |  |  |  |  |
| M | 0004693 | cyclin-dependent protein kinase activity | EN-124k-90-group5102.Contig1 | | | | | | | | | |  |  |  |  |
| M | 0004693 | cyclin-dependent protein kinase activity | EN-124k-90-group5102.Contig2 | | | | | | | | | |  |  |  |  |
| M | 0004693 | cyclin-dependent protein kinase activity | EN-124k-90-group5102.Contig3 | | | | | | | | | |  |  |  |  |
| M | 0004693 | cyclin-dependent protein kinase activity | EN-124k-90-group5173.Contig1 | | | | | | | | | |  |  |  |  |
| M | 0004693 | cyclin-dependent protein kinase activity | EN-124k-90-group5273.Contig2 | | | | | | | | | |  |  |  |  |
| M | 0004693 | cyclin-dependent protein kinase activity | EN-124k-90-group5273.Contig3 | | | | | | | | | |  |  |  |  |
| M | 0004693 | cyclin-dependent protein kinase activity | EN-124k-90-group5411.Contig1 | | | | | | | | | |  |  |  |  |
| M | 0004693 | cyclin-dependent protein kinase activity | EN-124k-90-group5479.Contig1 | | | | | | | | | |  |  |  |  |
| M | 0004693 | cyclin-dependent protein kinase activity | EN-124k-90-group5542.Contig1 | | | | | | | | | |  |  |  |  |
| M | 0004693 | cyclin-dependent protein kinase activity | EN-124k-90-group5607.Contig1 | | | | | | | | | |  |  |  |  |
| M | 0004693 | cyclin-dependent protein kinase activity | EN-124k-90-group5668.Contig1 | | | | | | | | | |  |  |  |  |
| M | 0004693 | cyclin-dependent protein kinase activity | EN-124k-90-group5717.Contig2 | | | | | | | | | |  |  |  |  |
| M | 0004693 | cyclin-dependent protein kinase activity | EN-124k-90-group5968.Contig1 | | | | | | | | | |  |  |  |  |
| M | 0004693 | cyclin-dependent protein kinase activity | EN-124k-90-group6038.Contig1 | | | | | | | | | |  |  |  |  |
| M | 0004693 | cyclin-dependent protein kinase activity | EN-124k-90-group6204.Contig1 | | | | | | | | | |  |  |  |  |
| M | 0004693 | cyclin-dependent protein kinase activity | EN-124k-90-group6204.Contig2 | | | | | | | | | |  |  |  |  |
| M | 0004693 | cyclin-dependent protein kinase activity | EN-124k-90-group6291.Contig1 | | | | | | | | | |  |  |  |  |
| M | 0004693 | cyclin-dependent protein kinase activity | EN-124k-90-group6291.Contig2 | | | | | | | | | |  |  |  |  |
| M | 0004693 | cyclin-dependent protein kinase activity | EN-124k-90-group6358.Contig2 | | | | | | | | | |  |  |  |  |
| M | 0004693 | cyclin-dependent protein kinase activity | EN-124k-90-group6400.Contig1 | | | | | | | | | |  |  |  |  |
| M | 0004693 | cyclin-dependent protein kinase activity | EN-124k-90-group6666.Contig1 | | | | | | | | | |  |  |  |  |
| M | 0004693 | cyclin-dependent protein kinase activity | EN-124k-90-group6923.Contig1 | | | | | | | | | |  |  |  |  |
| M | 0004693 | cyclin-dependent protein kinase activity | EN-124k-90-group6923.Contig2 | | | | | | | | | |  |  |  |  |
| M | 0004693 | cyclin-dependent protein kinase activity | EN-124k-90-group7046.Contig1 | | | | | | | | | |  |  |  |  |
| M | 0004693 | cyclin-dependent protein kinase activity | EN-124k-90-group7149.Contig2 | | | | | | | | | |  |  |  |  |
| M | 0004693 | cyclin-dependent protein kinase activity | EN-124k-90-group7157.Contig2 | | | | | | | | | |  |  |  |  |
| M | 0004693 | cyclin-dependent protein kinase activity | EN-124k-90-group7231.Contig1 | | | | | | | | | |  |  |  |  |
| M | 0004693 | cyclin-dependent protein kinase activity | EN-124k-90-group7343.Contig1 | | | | | | | | | |  |  |  |  |
| M | 0004693 | cyclin-dependent protein kinase activity | EN-124k-90-group7528.Contig1 | | | | | | | | | |  |  |  |  |
| M | 0004693 | cyclin-dependent protein kinase activity | EN-124k-90-group7612.Contig2 | | | | | | | | | |  |  |  |  |
| M | 0004693 | cyclin-dependent protein kinase activity | EN-124k-90-group7866.Contig1 | | | | | | | | | |  |  |  |  |
| M | 0004693 | cyclin-dependent protein kinase activity | EN-124k-90-group7876.Contig1 | | | | | | | | | |  |  |  |  |
| M | 0004693 | cyclin-dependent protein kinase activity | EN-124k-90-group8079.Contig1 | | | | | | | | | |  |  |  |  |
| M | 0004693 | cyclin-dependent protein kinase activity | EN-124k-90-group8079.Contig2 | | | | | | | | | |  |  |  |  |
| E | 0004693 | cyclin-dependent protein kinase activity | EN-124k-90-group8101.Contig1 | | | | | | | | | |  |  |  |  |
| M | 0004693 | cyclin-dependent protein kinase activity | EN-124k-90-group8211.Contig1 | | | | | | | | | |  |  |  |  |
| M | 0004693 | cyclin-dependent protein kinase activity | EN-124k-90-group8306.Contig1 | | | | | | | | | |  |  |  |  |
| M | 0004693 | cyclin-dependent protein kinase activity | EN-124k-90-group8306.Contig3 | | | | | | | | | |  |  |  |  |
| M | 0004693 | cyclin-dependent protein kinase activity | EN-124k-90-group8306.Contig4 | | | | | | | | | |  |  |  |  |
| M | 0004693 | cyclin-dependent protein kinase activity | EN-124k-90-group8492.Contig1 | | | | | | | | | |  |  |  |  |
| M | 0004693 | cyclin-dependent protein kinase activity | EN-124k-90-group8524.Contig1 | | | | | | | | | |  |  |  |  |
| M | 0004693 | cyclin-dependent protein kinase activity | EN-124k-90-group8652.Contig1 | | | | | | | | | |  |  |  |  |
| M | 0004693 | cyclin-dependent protein kinase activity | EN-124k-90-group8652.Contig2 | | | | | | | | | |  |  |  |  |
| M | 0004693 | cyclin-dependent protein kinase activity | EN-124k-90-group8652.Contig3 | | | | | | | | | |  |  |  |  |
| A | 0004693 | cyclin-dependent protein kinase activity | EN-124k-90-group8668.Contig1 | | | | | | | | | |  |  |  |  |
| M | 0004693 | cyclin-dependent protein kinase activity | EN-124k-90-group8764.Contig1 | | | | | | | | | |  |  |  |  |
| M | 0004693 | cyclin-dependent protein kinase activity | EN-124k-90-group8799.Contig2 | | | | | | | | | |  |  |  |  |
| M | 0004693 | cyclin-dependent protein kinase activity | EN-124k-90-group8850.Contig1 | | | | | | | | | |  |  |  |  |
| M | 0004693 | cyclin-dependent protein kinase activity | EN-124k-90-group8937.Contig2 | | | | | | | | | |  |  |  |  |
| M | 0004693 | cyclin-dependent protein kinase activity | EN-124k-90-group9079.Contig1 | | | | | | | | | |  |  |  |  |
| M | 0004693 | cyclin-dependent protein kinase activity | EN-124k-90-group9299.Contig3 | | | | | | | | | |  |  |  |  |
| M | 0004693 | cyclin-dependent protein kinase activity | EN-124k-90-group9317.Contig1 | | | | | | | | | |  |  |  |  |
| M | 0004693 | cyclin-dependent protein kinase activity | EN-124k-90-group9388.Contig1 | | | | | | | | | |  |  |  |  |
| M | 0004693 | cyclin-dependent protein kinase activity | EN-124k-90-group9388.Contig2 | | | | | | | | | |  |  |  |  |
| M | 0004693 | cyclin-dependent protein kinase activity | EN-124k-90-group9446.Contig1 | | | | | | | | | |  |  |  |  |
| M | 0004693 | cyclin-dependent protein kinase activity | EN-124k-90-group9819.Contig1 | | | | | | | | | |  |  |  |  |
| M | 0004693 | cyclin-dependent protein kinase activity | EN-124k-90-group9843.Contig1 | | | | | | | | | |  |  |  |  |
| M | 0004693 | cyclin-dependent protein kinase activity | EN-124k-90-group10085.Contig1 | | | | | | | | | |  |  |  |  |
| M | 0004693 | cyclin-dependent protein kinase activity | EN-124k-90-group10085.Contig2 | | | | | | | | | |  |  |  |  |
| M | 0004693 | cyclin-dependent protein kinase activity | EN-124k-90-group10090.Contig2 | | | | | | | | | |  |  |  |  |
| M | 0004693 | cyclin-dependent protein kinase activity | EN-124k-90-group10221.Contig1 | | | | | | | | | |  |  |  |  |
| M | 0004693 | cyclin-dependent protein kinase activity | EN-124k-90-group10598.Contig1 | | | | | | | | | |  |  |  |  |
| M | 0004693 | cyclin-dependent protein kinase activity | EN-124k-90-group10630.Contig2 | | | | | | | | | |  |  |  |  |
| M | 0004693 | cyclin-dependent protein kinase activity | EN-124k-90-group10637.Contig1 | | | | | | | | | |  |  |  |  |
| M | 0004693 | cyclin-dependent protein kinase activity | EN-124k-90-group10712.Contig1 | | | | | | | | | |  |  |  |  |
| M | 0004693 | cyclin-dependent protein kinase activity | EN-124k-90-group10772.Contig3 | | | | | | | | | |  |  |  |  |
| M | 0004693 | cyclin-dependent protein kinase activity | EN-124k-90-group10824.Contig1 | | | | | | | | | |  |  |  |  |
| M | 0004693 | cyclin-dependent protein kinase activity | EN-124k-90-group10838.Contig1 | | | | | | | | | |  |  |  |  |
| M | 0004693 | cyclin-dependent protein kinase activity | EN-124k-90-group10838.Contig2 | | | | | | | | | |  |  |  |  |
| M | 0004693 | cyclin-dependent protein kinase activity | EN-124k-90-group10877.Contig1 | | | | | | | | | |  |  |  |  |
| M | 0004693 | cyclin-dependent protein kinase activity | EN-124k-90-group10912.Contig1 | | | | | | | | | |  |  |  |  |
| M | 0004693 | cyclin-dependent protein kinase activity | EN-124k-90-group10928.Contig1 | | | | | | | | | |  |  |  |  |
| M | 0004693 | cyclin-dependent protein kinase activity | EN-124k-90-group11228.Contig1 | | | | | | | | | |  |  |  |  |
| M | 0004693 | cyclin-dependent protein kinase activity | EN-124k-90-group11235.Contig1 | | | | | | | | | |  |  |  |  |
| M | 0004693 | cyclin-dependent protein kinase activity | EN-124k-90-group11408.Contig1 | | | | | | | | | |  |  |  |  |
| M | 0004693 | cyclin-dependent protein kinase activity | EN-124k-90-group11808.Contig1 | | | | | | | | | |  |  |  |  |
| M | 0004693 | cyclin-dependent protein kinase activity | EN-124k-90-group12099.Contig1 | | | | | | | | | |  |  |  |  |
| M | 0004693 | cyclin-dependent protein kinase activity | EN-124k-90-group12519.Contig1 | | | | | | | | | |  |  |  |  |
| M | 0004693 | cyclin-dependent protein kinase activity | EN-124k-90-group12973.Contig1 | | | | | | | | | |  |  |  |  |
| M | 0004693 | cyclin-dependent protein kinase activity | EN-124k-90-group13235.Contig1 | | | | | | | | | |  |  |  |  |
| M | 0004693 | cyclin-dependent protein kinase activity | EN-124k-90-group13235.Contig2 | | | | | | | | | |  |  |  |  |
| M | 0004693 | cyclin-dependent protein kinase activity | EN-124k-90-group14708.Contig1 | | | | | | | | | |  |  |  |  |
| A | 0004693 | cyclin-dependent protein kinase activity | EN-124k-90-group230.gs\_25885 | | | | | | | | | |  |  |  |  |
| E | 0004693 | cyclin-dependent protein kinase activity | EN-124k-90-group900.EN\_iowa\_15436 | | | | | | | | | | |  |  |  |
| A | 0004693 | cyclin-dependent protein kinase activity | EN-124k-90-group900.gs\_31783 | | | | | | | | | |  |  |  |  |
| A | 0004693 | cyclin-dependent protein kinase activity | EN-124k-90-group900.gs\_71447 | | | | | | | | | |  |  |  |  |
| A | 0004693 | cyclin-dependent protein kinase activity | EN-124k-90-group1782.gs\_16663 | | | | | | | | | |  |  |  |  |
| E | 0004693 | cyclin-dependent protein kinase activity | EN-124k-90-group2409.EN\_iowa\_9419 | | | | | | | | | | |  |  |  |
| E | 0004693 | cyclin-dependent protein kinase activity | EN-124k-90-group2409.EN\_iowa\_12940 | | | | | | | | | | |  |  |  |
| A | 0004693 | cyclin-dependent protein kinase activity | EN-124k-90-group2449.gs\_35369 | | | | | | | | | |  |  |  |  |
| E | 0004693 | cyclin-dependent protein kinase activity | EN-124k-90-group2778.jgi\_contig\_JGI\_CBBP17773\_fwd | | | | | | | | | | | | |  |
| A | 0004693 | cyclin-dependent protein kinase activity | EN-124k-90-group4349.gs\_42524 | | | | | | | | | |  |  |  |  |
| E | 0004693 | cyclin-dependent protein kinase activity | EN-124k-90-group5607.jgi\_contig\_JGI\_CBBP445\_fwd | | | | | | | | | | | | |  |
| E | 0004693 | cyclin-dependent protein kinase activity | EN-124k-90-group6204.EN\_iowa\_1799 | | | | | | | | | | |  |  |  |
| E | 0004693 | cyclin-dependent protein kinase activity | EN-124k-90-group6204.EN\_iowa\_4716 | | | | | | | | | | |  |  |  |
| E | 0004693 | cyclin-dependent protein kinase activity | EN-124k-90-group6291.EN\_iowa\_6413 | | | | | | | | | | |  |  |  |
| E | 0004693 | cyclin-dependent protein kinase activity | EN-124k-90-group6923.jgi\_contig\_JGI\_CBBP17789\_fwd | | | | | | | | | | | | |  |
| A | 0004693 | cyclin-dependent protein kinase activity | EN-124k-90-group8306.gs\_61906 | | | | | | | | | |  |  |  |  |
| A | 0007415 | defasciculation of motor neuron axon | EN-124k-90-group13468.gs\_23546 | | | | | | | | | |  |  |  |  |
| A | 0007415 | defasciculation of motor neuron axon | EN-124k-90-group4707.gs\_46826 | | | | | | | | | |  |  |  |  |
| E | 0007415 | defasciculation of motor neuron axon | EN-124k-90-group5333.jgi\_paired\_JGI\_CBBP5749\_fwd | | | | | | | | | | | | |  |
| E | 0007415 | defasciculation of motor neuron axon | EN-124k-90-group6152.jgi\_paired\_JGI\_CBBP3620\_fwd | | | | | | | | | | | | |  |
| E | 0007415 | defasciculation of motor neuron axon | EN-124k-90-group7869.jgi\_paired\_JGI\_CBBP11345\_fwd | | | | | | | | | | | | |  |
| E | 0007415 | defasciculation of motor neuron axon | EN-124k-90-group7940.jgi\_contig\_JGI\_CBBP6088\_fwd | | | | | | | | | | | | |  |
| E | 0007415 | defasciculation of motor neuron axon | EN-124k-90-group8721.jgi\_paired\_JGI\_CBBP11443\_rev | | | | | | | | | | | | |  |
| A | 0007415 | defasciculation of motor neuron axon | EN-124k-90-group9981.gs\_67492 | | | | | | | | | |  |  |  |  |
| A | 0007415 | defasciculation of motor neuron axon | EN-124k-90-group10635.gs\_47150 | | | | | | | | | |  |  |  |  |
| E | 0007415 | defasciculation of motor neuron axon | EN-124k-90-group10888.jgi\_paired\_JGI\_CBBP10968\_rev | | | | | | | | | | | | |  |
| E | 0007415 | defasciculation of motor neuron axon | EN-124k-90-group11394.jgi\_paired\_JGI\_CBBP16404\_fwd | | | | | | | | | | | | |  |
| E | 0007415 | defasciculation of motor neuron axon | EN-124k-90-group12072.jgi\_paired\_JGI\_CBBP11443\_fwd | | | | | | | | | | | | |  |
| A | 0007415 | defasciculation of motor neuron axon | EN-124k-90-group12204.gs\_9947 | | | | | | | | | |  |  |  |  |
| E | 0007415 | defasciculation of motor neuron axon | EN-124k-90-group13023.EN\_iowa\_251 | | | | | | | | | | |  |  |  |
| E | 0007415 | defasciculation of motor neuron axon | EN-124k-90-group14050.jgi\_paired\_JGI\_CBBP18955\_fwd | | | | | | | | | | | | |  |
| A | 0007415 | defasciculation of motor neuron axon | EN-124k-90-group14582.gs\_17139 | | | | | | | | | |  |  |  |  |
| E | 0007415 | defasciculation of motor neuron axon | EN-124k-90-group15671.jgi\_paired\_JGI\_CBBP10266\_fwd | | | | | | | | | | | | |  |
| A | 0007415 | defasciculation of motor neuron axon | EN-124k-90-group16026.gs\_15943 | | | | | | | | | |  |  |  |  |
| E | 0007415 | defasciculation of motor neuron axon | EN-124k-90-group16134.jgi\_contig\_JGI\_CBBP14519\_fwd | | | | | | | | | | | | |  |
| A | 0007415 | defasciculation of motor neuron axon | EN-124k-90-group16149.gs\_13439 | | | | | | | | | |  |  |  |  |
| A | 0007415 | defasciculation of motor neuron axon | EN-124k-90-group16523.gs\_75670 | | | | | | | | | |  |  |  |  |
| A | 0007415 | defasciculation of motor neuron axon | EN-124k-90-group16600.gs\_29512 | | | | | | | | | |  |  |  |  |
| A | 0007415 | defasciculation of motor neuron axon | EN-124k-90-group17702.gs\_18827 | | | | | | | | | |  |  |  |  |
| A | 0007415 | defasciculation of motor neuron axon | EN-124k-90-group17706.gs\_80669 | | | | | | | | | |  |  |  |  |
| A | 0007415 | defasciculation of motor neuron axon | EN-124k-90-group17930.gs\_83513 | | | | | | | | | |  |  |  |  |
| A | 0007415 | defasciculation of motor neuron axon | EN-124k-90-group18592.gs\_31083 | | | | | | | | | |  |  |  |  |
| A | 0007415 | defasciculation of motor neuron axon | EN-124k-90-group18929.gs\_13816 | | | | | | | | | |  |  |  |  |
| M | 0007415 | defasciculation of motor neuron axon | EN-124k-90-group115.Contig1 | | | | | | | | | |  |  |  |  |
| M | 0007415 | defasciculation of motor neuron axon | EN-124k-90-group115.Contig2 | | | | | | | | | |  |  |  |  |
| M | 0007415 | defasciculation of motor neuron axon | EN-124k-90-group583.Contig1 | | | | | | | | | |  |  |  |  |
| M | 0007415 | defasciculation of motor neuron axon | EN-124k-90-group586.Contig2 | | | | | | | | | |  |  |  |  |
| M | 0007415 | defasciculation of motor neuron axon | EN-124k-90-group745.Contig1 | | | | | | | | | |  |  |  |  |
| M | 0007415 | defasciculation of motor neuron axon | EN-124k-90-group745.Contig2 | | | | | | | | | |  |  |  |  |
| M | 0007415 | defasciculation of motor neuron axon | EN-124k-90-group925.Contig1 | | | | | | | | | |  |  |  |  |
| M | 0007415 | defasciculation of motor neuron axon | EN-124k-90-group925.Contig2 | | | | | | | | | |  |  |  |  |
| M | 0007415 | defasciculation of motor neuron axon | EN-124k-90-group925.Contig3 | | | | | | | | | |  |  |  |  |
| M | 0007415 | defasciculation of motor neuron axon | EN-124k-90-group1001.Contig1 | | | | | | | | | |  |  |  |  |
[truncated: 2,168,582 more chars]
