# Supplementary material for: Construction of a medicinal leech transcriptome database and its application to the identification of leech homologs of neural and innate immune genes
Source: BMC Genomics. 2010 Jun 25;11:407. doi: 10.1186/1471-2164-11-407 (PMC2996935; doi:10.1186/1471-2164-11-407)
Supplement: Additional file 3 — Supplementary Table S3: Immune System Transcripts. Transcripts encoding Hirudo proteins with homology to immune factors, listed by functional groups. Transcript IDs are linked to protein sequence alignment summaries provided via the Leechmaster Database http://genomes.ucsd.edu/leechmaster. [file 1471-2164-11-407-S3.HTM]

| Medicinal Leech Transcriptome Database  Supplementary Table 3 |  |  |  |  |  |  |  |  |  |  |  |  |  |  |  |  |  |  |  |
|  |  |  |  |  |  |  |  |  |  |  |  |  |  |  |  |  |  |  |  |
|  |  |  |  |  |  |  |  |  |  |  |  |  |  |  |  |  |  |  |  |
|  | Embryo |  |  |  |  |  |  |  |  |  |  |  |  |  |  |  |  |  |  |
| Immune Factors | Adult CNS | Transcript ID |  |  |  |  |  |  |  |  |  |  |  |  |  |  |  |  |  |
|  | Mixed |  |  |  |  |  |  |  |  |  |  |  |  |  |  |  |  |  |  |
| PRR pathway proteins |  |  |  |  |  |  |  |  |  |  |  |  |  |  |  |  |  |  |  |
| TLR | A | EN-124k-90-group9059.gs\_31860 | | | | | | | | | | | | | | |  |  |  |
| LBP | M | EN-124k-90-group1854.Contig2 | | | | | | | | | | | | | | |  |  |  |
| Myeloid differentiation factor 88 (MyD88) | M | EN-124k-90-group4501.Contig1 | | | | | | | | | | | | | | |  |  |  |
| Sterile alpha and TIR motif-containing protein (SARM) | M | EN-124k-90-group12447.Contig1 | | | | | | | | | | | | | | |  |  |  |
| Interleukin-1 receptor-associated kinase 4 (IRAK-4) | M | EN-124k-90-group10838.Contig2 | | | | | | | | | | | | | | |  |  |  |
| Toll-interacting protein (TOLLIP) | M | EN-124k-90-group2577.Contig1 | | | | | | | | | | | | | | |  |  |  |
| TNF receptor associated factor 6 (TRAF-6) | M | EN-124k-90-group11548.Contig1 | | | | | | | | | | | | | | |  |  |  |
| MEKK | M | EN-124k-90-group9317.Contig1 | | | | | | | | | | | | | | |  |  |  |
| IKK-like protein | E | EN-124k-90-group7594.jgi\_paired\_JGI\_CBBP17768\_fwd | | | | | | | | | | | | | | | | | |
| TAK1 | M | EN-124k-90-group5380.Contig1 | | | | | | | | | | | | | | |  |  |  |
| MKK3/6 | M | EN-124k-90-group324.Contig1 | | | | | | | | | | | | | |  |  |  |  |
| MKK4/7 | M | EN-124k-90-group324.Contig1 | | | | | | | | | | | | | |  |  |  |  |
| P38 mitogen activated protein kinase (MAPK | M | EN-124k-90-group3313.Contig3 | | | | | | | | | | | | | | |  |  |  |
| JNK | M | EN-124k-90-group14563.gs\_48486 | | | | | | | | | | | | | | |  |  |  |
| NF-kappa-B p105 subunit (Contains: NFkB p50 subunit) | M | EN-124k-90-group2116.Contig3 | | | | | | | | | | | | | | |  |  |  |
| I\_B | M | EN-124k-90-group4424.Contig1 | | | | | | | | | | | | | | |  |  |  |
| Interferon Regulatory Factor (IRF) | M | EN-124k-90-group9768.Contig2 | | | | | | | | | | | | | | |  |  |  |
| Activator protein 1 (AP1) | M | EN-124k-90-group883.Contig1 | | | | | | | | | | | | | |  |  |  |  |
| Tumor necrosis factor (TNF) receptor-associated factor 3 (TRAF-3) | M | EN-124k-90-group116.Contig1 | | | | | | | | | | | | | |  |  |  |  |
| Gene associated with Retinoid-IFN-induced Mortality (GRIM) -19 | M | EN-124k-90-group960.Contig2 | | | | | | | | | | | | | |  |  |  |  |
| Fas Associated via Death Domain (FADD) | M | EN-124k-90-group7037.Contig2 | | | | | | | | | | | | | | |  |  |  |
| Caspase 3 | M | EN-124k-90-group3438.Contig2 | | | | | | | | | | | | | | |  |  |  |
| Caspase 7 | M | EN-124k-90-group8152.Contig2 | | | | | | | | | | | | | | |  |  |  |
| Evolutionarily conserved signalling intermediate in toll pathways (ECSIT) | A | EN-124k-90-group1535.gs\_78653 | | | | | | | | | | | | | | |  |  |  |
|  |  |  |  |  |  |  |  |  |  |  |  |  |  |  |  |  |  |  |  |
| Pattern recognition Receptors (PRRs) |  |  |  |  |  |  |  |  |  |  |  |  |  |  |  |  |  |  |  |
| related to Toll-like receptor 13 precursor | M | EN-124k-90-group5788.Contig1 | | | | | | | | | | | | | | |  |  |  |
| related to Toll receptor - Chlamys farreri | M | EN-124k-90-group5461.Contig1 | | | | | | | | | | | | | | |  |  |  |
| similar to toll-like receptor 3, partial [Strongylocentrotus purpuratus] | A | EN-124k-90-group2932.gs\_32728 | | | | | | | | | | | | | | |  |  |  |
| related to Toll - Aedes aegypti | M | EN-124k-90-group8103.Contig4 | | | | | | | | | | | | | | |  |  |  |
| similar to NOD3 protein [Rattus norvegicus] | E | EN-124k-90-group14222.EN\_iowa\_13328 | | | | | | | | | | | | | | | |  |  |
| Caspase recruitment domain (CARD)-containing protein 10 (Bimp1) | M | EN-124k-90-group574.Contig1 | | | | | | | | | | | | | |  |  |  |  |
| RIG-1 like Receptors (RLRs) | A | EN-124k-90-group1633.Contig1 | | | | | | | | | | | | | | |  |  |  |
| Immunoglobulin superfamily receptors | A | EN-124k-90-group10601.gs\_47217 | | | | | | | | | | | | | | |  |  |  |
| Genes containing Leucine Rich Repeats (LRRs) | M | EN-124k-90-group12504.Contig1 | | | | | | | | | | | | | | |  |  |  |
|  | M | EN-124k-90-group2607.Contig7 | | | | | | | | | | | | | | |  |  |  |
|  | A | EN-124k-90-group10370.Contig1 | | | | | | | | | | | | | | |  |  |  |
|  | M | EN-124k-90-group2811.Contig2 | | | | | | | | | | | | | | |  |  |  |
|  |  |  |  |  |  |  |  |  |  |  |  |  |  |  |  |  |  |  |  |
| Antimicrobial response factors (Effectors) |  |  |  |  |  |  |  |  |  |  |  |  |  |  |  |  |  |  |  |
| Destabilase 1 (lysozyme) | M | EN-124k-90-group5917.Contig1 | | | | | | | | | | | | | | |  |  |  |
| Theromacin (antimicrobial peptide ) | A | EN-124k-90-group213.gs\_71118 | | | | | | | | | | | | | | |  |  |  |
| Bactericidal permeability-increasing protein | M | EN-124k-90-group7476.Contig1 | | | | | | | | | | | | | | |  |  |  |
| Theromyzin | M | EN-124k-90-group2417.Contig1 | | | | | | | | | | | | | | |  |  |  |
| Tryptase inhibitor (LDTI) | A | EN-124k-90-group5993.gs\_76110 | | | | | | | | | | | | | | |  |  |  |
| Eglin-C | M | EN-124k-90-group2924.Contig1 | | | | | | | | | | | | | | |  |  |  |
| LPS binding protein/ Bactericidal Permeability Increasing protein (LBP/BPI) | M | EN-124k-90-group1854.Contig2 | | | | | | | | | | | | | | |  |  |  |
|  | M | EN-124k-90-group1134.Contig2 | | | | | | | | | | | | | | |  |  |  |
| Dicer | M | EN-124k-90-group8614.Contig1 | | | | | | | | | | | | | | |  |  |  |
| Drosha | M | EN-124k-90-group389.Contig3 | | | | | | | | | | | | | |  |  |  |  |
| Argonaute-like protein | M | EN-124k-90-group3359.Contig1 | | | | | | | | | | | | | | |  |  |  |
|  |  |  |  |  |  |  |  |  |  |  |  |  |  |  |  |  |  |  |  |
| Complement system |  |  |  |  |  |  |  |  |  |  |  |  |  |  |  |  |  |  |  |
| Ficolins | M | EN-124k-90-group8905.Contig1 | | | | | | | | | | | | | | |  |  |  |
|  | M | EN-124k-90-group8905.Contig2 | | | | | | | | | | | | | | |  |  |  |
| Galectin | M | EN-124k-90-group6136.Contig1 | | | | | | | | | | | | | | |  |  |  |
|  | A | EN-124k-90-group10956.Contig1 | | | | | | | | | | | | | | |  |  |  |
| Techylectin 5A | M | EN-124k-90-group29.Contig15 | | | | | | | | | | | | | |  |  |  |  |
| C-type lectin 1 | M | EN-124k-90-group2719.Contig2 | | | | | | | | | | | | | | |  |  |  |
|  | M | EN-124k-90-group4856.Contig1 | | | | | | | | | | | | | | |  |  |  |
| Mannose binding lectin | A | EN-124k-90-group9776.Contig1 | | | | | | | | | | | | | | |  |  |  |
| Pantraxin | M | EN-124k-90-group2896.Contig1 | | | | | | | | | | | | | | |  |  |  |
| Fucolectin-5 | M | EN-124k-90-group2896.Contig1 | | | | | | | | | | | | | | |  |  |  |
| C1q | M | EN-124k-90-group7146.Contig1 | | | | | | | | | | | | | | |  |  |  |
| C1q receptor | M | EN-124k-90-group3343.Contig1 | | | | | | | | | | | | | | |  |  |  |
| Similar to C3 and PDZ domain | A | EN-124k-90-group9011.gs\_72608 | | | | | | | | | | | | | | |  |  |  |
| \_2 macroglobulin receptor | M | EN-124k-90-group1421.Contig1 | | | | | | | | | | | | | | |  |  |  |
| Thioester containing protein | M | EN-124k-90-group455.Contig9 | | | | | | | | | | | | | |  |  |  |  |
| \_2 macroglobulin | M | EN-124k-90-group3534.Contig2 | | | | | | | | | | | | | | |  |  |  |
| MASP | M | EN-124k-90-group25.Contig4 | | | | | | | | | | | | | |  |  |  |  |
| Phenoloxidase | A | EN-124k-90-group13562.gs\_23887 | | | | | | | | | | | | | | |  |  |  |
| Serpin B1 (leukocyte elastase inhibitor) | M | EN-124k-90-group3562.Contig2 | | | | | | | | | | | | | | |  |  |  |
|  |  |  |  |  |  |  |  |  |  |  |  |  |  |  |  |  |  |  |  |
| Clotting and fibrinolytic cascades |  |  |  |  |  |  |  |  |  |  |  |  |  |  |  |  |  |  |  |
| Angiopoietin precursor | A | EN-124k-90-group10607.Contig1 | | | | | | | | | | | | | | |  |  |  |
| Fibrinogen and fibronectin | M | EN-124k-90-group29.Contig15 | | | | | | | | | | | | | |  |  |  |  |
| Kallikrein precursor | E | EN-124k-90-group8575.Contig1 | | | | | | | | | | | | | | |  |  |  |
| Antistasin | A | EN-124k-90-group721.EN\_iowa\_16324 | | | | | | | | | | | | | | |  |  |  |
|  |  |  |  |  |  |  |  |  |  |  |  |  |  |  |  |  |  |  |  |
| Cytokines |  |  |  |  |  |  |  |  |  |  |  |  |  |  |  |  |  |  |  |
| p43/Endothelial monocyte-activating polypeptide 2 (EMAP2) | M | EN-124k-90-group3932.Contig1 | | | | | | | | | | | | | | |  |  |  |
| TNF alpha | M | EN-124k-90-group5457.Contig1 | | | | | | | | | | | | | | |  |  |  |
| Granulins | M | EN-124k-90-group4406.Contig1 | | | | | | | | | | | | | | |  |  |  |
| TDP43 | M | EN-124k-90-group9372.Contig2 | | | | | | | | | | | | | | |  |  |  |
| Interleukin-16 | M | EN-124k-90-group1525.Contig1 | | | | | | | | | | | | | | |  |  |  |
|  |  |  |  |  |  |  |  |  |  |  |  |  |  |  |  |  |  |  |  |
| Cluster of differentiation related molecules |  |  |  |  |  |  |  |  |  |  |  |  |  |  |  |  |  |  |  |
| CD45 related protein | M | EN-124k-90-group745.Contig1 | | | | | | | | | | | | | |  |  |  |  |
| CD61 related protein | M | EN-124k-90-group2132.Contig1 | | | | | | | | | | | | | | |  |  |  |
| CD20 related protein (membrane-spanning 4A/chandra) | M | EN-124k-90-group1075.gs\_7681 | | | | | | | | | | | | | | |  |  |  |
| CD19 related protein | M | EN-124k-90-group11960.Contig1 | | | | | | | | | | | | | | |  |  |  |
| CD38 related protein | M | EN-124k-90-group4537.Contig1 | | | | | | | | | | | | | | |  |  |  |
| CD69 related protein | M | EN-124k-90-group9470.Contig2 | | | | | | | | | | | | | | |  |  |  |
| CD13 related protein | M | EN-124k-90-group8605.Contig1 | | | | | | | | | | | | | | |  |  |  |
| CD206 antigen | A | EN-124k-90-group5008.Contig1 | | | | | | | | | | | | | | |  |  |  |
|  |  |  |  |  |  |  |  |  |  |  |  |  |  |  |  |  |  |  |  |
| Genes related to vertebrate type adaptive immune system |  |  |  |  |  |  |  |  |  |  |  |  |  |  |  |  |  |  |  |
| Recombination activating gene 1 (RAG1) activation protein | M | EN-124k-90-group3472.Contig1 | | | | | | | | | | | | | | |  |  |  |
| Calreticulin | M | EN-124k-90-group2770.Contig6 | | | | | | | | | | | | | | |  |  |  |
| Calnexin | M | EN-124k-90-group2770.Contig6 | | | | | | | | | | | | | | |  |  |  |
| Cathepsin B | M | EN-124k-90-group5112.Contig3 | | | | | | | | | | | | | | |  |  |  |
| Cathepsin I | M | EN-124k-90-group9873.Contig2 | | | | | | | | | | | | | | |  |  |  |
| Cathepsin Y | M | EN-124k-90-group2952.Contig1 | | | | | | | | | | | | | | |  |  |  |
| Cathepsin L | M | EN-124k-90-group8510.Contig1 | | | | | | | | | | | | | | |  |  |  |
| Cathepsin Z | M | EN-124k-90-group2952.Contig1 | | | | | | | | | | | | | | |  |  |  |
| Cathepsin C | M | EN-124k-90-group4705.Contig1 | | | | | | | | | | | | | | |  |  |  |
| Cystatin B | M | EN-124k-90-group5885.Contig1 | | | | | | | | | | | | | | |  |  |  |
| MHC class I antigen Cw\*2 | A | EN-124k-90-group97.gs\_16455 | | | | | | | | | | | | | |  |  |  |  |
| HLA-DR-associated protein II | A | EN-124k-90-group2033.gs\_13434 | | | | | | | | | | | | | | |  |  |  |
